# Integrative Bioinformatics Approaches to Map Potential Novel Genes and Pathways Involved in Ovarian Cancer

**DOI:** 10.3389/fbioe.2019.00391

**Published:** 2019-12-17

**Authors:** S. Udhaya Kumar, D. Thirumal Kumar, R. Siva, C. George Priya Doss, Hatem Zayed

**Affiliations:** ^1^School of Biosciences and Technology, Vellore Institute of Technology, Vellore, India; ^2^Department of Biomedical Sciences, College of Health and Sciences, Qatar University, Doha, Qatar

**Keywords:** ovarian cancer, protein–protein interactions, Metacore, biomarkers, functional enrichment analysis, expression profiling data, microarray

## Abstract

**Background and aims:** Ovarian cancer (OC) is the seventh most commonly detected cancer among women. This study aimed to map the hub and core genes and potential pathways that might be involved in the molecular pathogenesis of OC.

**Methods:** In the present work, we analyzed a microarray dataset (GSE126519) from the Gene Expression Omnibus (GEO) database and used the GEO2R tool to screen OC cells and ovarian SINE-resistant cancer cells for differentially expressed genes (DEGs). For the functional annotation of the DEGs, we conducted Gene Ontology (GO) and pathway enrichment analyses (KEGG) using the DAVID v6.8 online server and GenoGo Metacore™, Cortellis Solution software. Protein–protein interaction (PPI) networks were constructed using the STRING database, and Cytoscape software was used for visualization. The survival analysis was performed using the online platform GEPIA2 to determine the prognostic value of the expression of hub genes in cell lines from OC patients.

**Results:** We identified a total of 809 upregulated and 700 downregulated DEGs. GO analysis revealed that the genes with statistically significant differences in expression were mainly associated with biological processes involved in the cell cycle, the mitotic cell cycle, mitotic nuclear division, organ morphogenesis, cell development, and cell morphogenesis. By using the Analyze Networks (AN) algorithm in GeneGo, we identified the most relevant biological networks involving DEGs that were mainly enriched in the cell cycle (in metaphase checkpoints) and revealed the role of APC in cell cycle regulation pathways. We found 10 hub genes and four core genes (*FZD6, FZD8, CDK2*, and *RBBP8*) that are strongly linked to OC.

**Conclusion:** This study sheds light on the molecular pathogenesis of OC and is expected to provide potential molecular biomarkers that are beneficial for the treatment and clinical molecular diagnosis of OC.

## Introduction

Ovarian cancer (OC) is the seventh most frequently detected cancer among women worldwide (Reid et al., 2017). Epithelial cancers represent ~90% of OC in patients with different ailments (Cancer Genome Atlas Research Network, 2011) comprising five distinct histological subtypes that have various distinguishable complications, sources of cells, molecular compositions, clinical signs, and symptoms and treatments (Matulonis et al., 2016). Matulonis et al. (2016) reported that OC is typically detected at the late stage, and no successful screening approaches have been found thus far. However, patients with an increased risk of OC with germline mutations in *BRCA1, BRCA2*, or additional genes can be identified (Pennington and Swisher, 2012; Younes and Zayed, 2019).

Proteins transported by exportin 1 (XPO1 or CMR1), such as IkB, p53, pRb, p21, p27, and FOXO, play significant roles as tumor suppressors. When restricted to the nucleus, they inhibit the growth of cells and cell survival unless they are transferred to the cytoplasm (Senapedis et al., 2014). A selective inhibitor of nuclear export (SINE) acts along with CMR1 to block its interaction with nuclear proteins intended to be exported to the cytoplasm; inhibitors of CMR1 are known as SINE compounds (Gerecitano, 2014). Recent work has also revealed that SINE compounds enhance the proteasomal deterioration of CMR1, increase the nuclear retention of FOXO and p53, and contribute to enhanced apoptosis in prostate cancer cell lines (Mendonca et al., 2014). As an outcome of resistance to treatment, the elevated annual mortality rate is due to a variety of diagnoses at advanced phases of the disease and recurrence of the disease. Additionally, OC comprises several subtypes with distinct etiologies and molecular profiles that result in considerable variations in the inherent sensitivity to treatment (Zyl et al., 2018). To overcome treatment resistance, there is a need to understand the complete set of molecular mechanisms underlying SINE resistance in OC cell lines. Therefore, the development of OC and associated phenomena need to be investigated, and there is an urgent need to find candidate early diagnostic biomarkers.

Microarray-based gene expression assessment is the most commonly used high-throughput and successful technique used to study complicated disease pathogenesis. However, studies performed that utilize human OC gene expression profiling are very uncommon. In the current research, we tried to explore the differentially expressed genes (DEGs), gene network, pathways, and protein interactions that are unique to OC. To detect the DEGs between OC and SINE-resistant OC cell lines (GSE126519), we utilized a bioinformatics approach to analyze DEG data retrieved from the Gene Expression Omnibus (GEO) database. For the screened DEGs, functional annotation assessment with Gene Ontology (GO) and pathway enrichment assessment with the Kyoto Encyclopedia of Genes and Genomes (KEGG) were carried out using the Database for Annotation, Visualization, and Integrated Discovery and GeneGo Metacore™ software. Ultimately, we found 10 potential hub genes and four core genes that were strongly linked to OC.

## Materials and Methods

### Data Preprocessing and Screening of DEGs

The expression profiling was performed on the OC gene dataset GSE126519, which was retrieved from GEO (Gene Expression Omnibus database, https://www.ncbi.nlm.nih.gov/geo/) and includes gene expression datasets from RNA-seq, high-throughput hybridization array, DNAseq, ChIPs, and microarray (Barrett et al., 2013). “Ovarian cancer” AND “Homo sapiens” were the keywords used to search OC-related expression profiles within the GEO datasets. The GSE126519 expression profiling was conducted in arrays that included three human OC cell lines and three SINE (selective inhibitors of nuclear export)-resistant human OC cell lines. We utilized the GEO2R (http://www.ncbi.nlm.nih.gov/geo/geo2r/) statistical tool to recalculate and assess the genes that were expressed differently between the human OC cell lines and the SINE-resistant human OC cell lines (Ritchie et al., 2015). The Benjamini and Hochberg (false discovery rate) and *t*-test methods were utilized with the GEO2R tool to calculate the FDR and *p*-values, respectively, to identify the DEGs. We considered *p* < 0.05 and a | log (fold change) | > 1 to be statistically significant for the DEGs, and logFC ≥ 1 and logFC ≤ −1 were considered to indicate upregulated and downregulated DEGs, respectively (Aubert et al., 2004).

By using all of the DEGs identified in the OC cell lines, we constructed a volcano plot by using the Volcano Plot (https://paolo.shinyapps.io/ShinyVolcanoPlot/) online server, which is hosted on shinyapps.io by RStudio. The resultant DEG dataset was collected and used for further analysis. The initial ontology of gene (GO) and KEGG pathway enrichment analyses of the DEGs was annotated (*p* < 0.05) using the online bioinformatics tool DAVID v6.8 (https://david.ncifcrf.gov/) (Huang et al., 2009a,b).

### PPI Network Construction

The online database STRING (v11.0, http://www.string-db.org/) was used to visualize the PPIs between the statistically significant DEG-encoded proteins in the resultant dataset (Szklarczyk et al., 2015). The dataset contained more than 10,000 DEGs. To avoid an inaccurate PPI network, we used a cutoff ≥ 0.9 (high-confidence interaction score) to obtain the significant PPIs. We used Cytoscape software v3.7.1 (http://www.cytoscape.org/) to visualize the PPI network obtained from the STRING database (Shannon et al., 2003). Based on the log fold change values, the PPI network was plotted for both the upregulated and downregulated DEGs. The interrelation analysis of the identified genes was performed by using the GeneMANIA online tool (Franz et al., 2018).

### Analyzing the Backbone Network

The NetworkAnalyzer app in Cytoscape was utilized to explore the networks of both the upregulated and downregulated DEGs (Saito et al., 2012). NetworkAnalyzer computes the topological parameters and centrality measures such as the distribution of the node degree, the betweenness centrality, the topological coefficients, the shortest path length, and the closeness centrality for directed and undirected networks (Assenov et al., 2008). The distribution of the node degree indicates the number of nodes with a certain degree and is a comparative measure of the degree to which a node parameter shares neighbors with other nodes in terms of the topological coefficient. NetworkAnalyzer calculates the topological coefficients for all network nodes with more than one neighbor (Stelzl et al., 2005). The networks that do not have multiple edges have been determined according to the betweenness centrality, whereas the closeness centrality computes this for all nodes and plots it against the number of neighbors in terms of the closeness centrality (Brandes, 2001; Newman, 2005).

### GeneGo Analysis

The statistically significant DEGs were further analyzed in Metacore and Cortellis Solution software (https://clarivate.com/products/metacore/, Clarivate Analytics, London, UK) to perform the GO function and pathway enrichment analyses. GeneGo enables the fast analysis of protein networks, metabolic pathways, and maps for the list of genes and proteins obtained from experimental high-throughput data (MetaCore Login|Clarivate Analytics^1^). We used the pathway maps tool to identify the enriched pathways involving DEGs in terms of the hypergeometric distribution, and the *p*-values were calculated by using the default database as the background (based on an FDR *p* < 0.005). Based on a significant *p*-value < 0.05, graphical depictions of the molecular interactions between the DEGs were generated.

### Hub Gene Survival Analysis

A comprehensive online platform called Gene Expression Profiling Interactive Analysis (GEPIA2, http://gepia2.cancer-pku.cn/#index) provides fast and customized delivery of functionalities based on TCGA (The Cancer Genome Atlas) and genotype-tissue expression (GTEx) data. GEPIA2 evaluates the survival effect of differentially expressed genes in a given cancer sample. The overall survival effect of hub genes in OC was estimated by calculating the log-rank *p*-value and the HR (hazard ratio-95% confidence interval) using GEPIA2 Single Gene Analysis. Then, the validation of the expression of the core hub genes in OC and normal tissues was performed and visualized in a boxplot (Tang et al., 2017).

## Results

### DEG Identification

**Figure 1:**
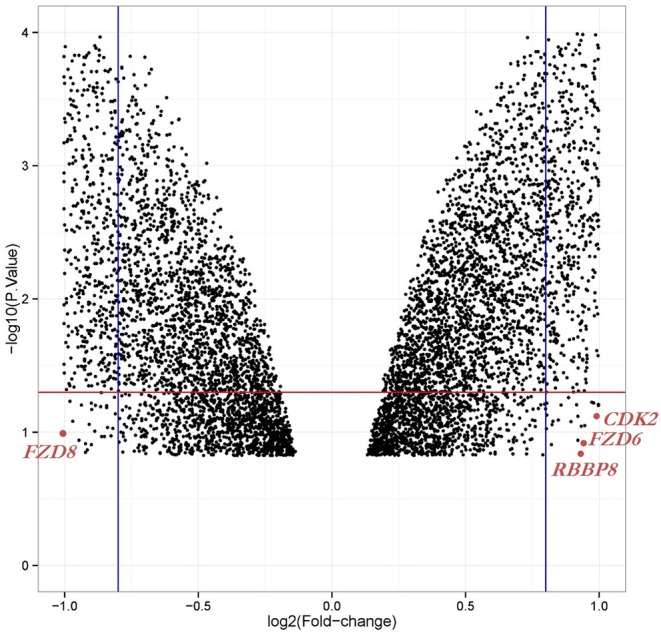
Volcano plot of the DEGs in OC compared with those in SINE-resistant OC from the GSE126519 dataset. *x*-axis: log2FC, large-scale fold changes; *y*-axis: –log10 of the *p*-value showing the statistical significance. Each black point corresponds to one gene. The black points above the red line and beside the blue line (left side and right side) represent log2FC ≥ 1 and *p*-value < 0.05; all DEGs that were OC-related are shown below the red line, which represents log2FC < 1 or *p*-value > 0.05. The position of the core genes are named and marked in scarlet color.

We obtained the gene expression profiles for GSE126519, “Analysis of RNA profiles in parent and selective inhibitors of nuclear export (SINE)-resistant OC cells” from the GEO datasets. (Miyake and Sood, 2019) submitted the GSE126519 dataset, which was generated on the GPL10558 platform (Illumina HumanHT-12 V4.0 expression bead chip). The GSE126519 dataset was obtained from three patient cell lines that comprised six samples, including three OC cell lines and three SINE-resistant OC cell lines (Table 1). To identify the DEGs from these two groups (OC and SINE-resistant OC), we conducted GEO2R web-server analysis (https://www.ncbi.nlm.nih.gov/geo/geo2r/?acc=GSE126519) to calculate the *p*-values and |log2FC| values. The resulting genes that met the cutoff criteria (|log2FC| ≥ 1.0 and *p* < 0.05) were considered DEGs. Overall, 8,855 genes were identified from the GEO dataset (GSE126519) with *p* > 0.05 and *p* < 0.05 using the GEO2R tool and are shown in Supplementary Table 1. We constructed a volcano plot using the Shiny Volcano Plot online server by Rstudio to compare the two groups; a total of 2708 DEGs were identified from the GSE126519 dataset (Figure 1). Among them, 809 and 700 genes were upregulated and downregulated, respectively, between two groups according to their log2FC and *p*-values (Supplementary Table 2).

**Table 1:** Patients' information in GSE126519 derived from the GEO database.

**Group**	**Accession**	**Patient no**.	**Organism**	**Disease state**	**Type**
OC	GSM3602932	Patient 1	*Homo sapiens*	Ovarian cancer	Human ovarian cancer cell
	GSM3602933	Patient 2	*Homo sapiens*	Ovarian cancer	Human ovarian cancer cell
	GSM3602934	Patient 3	*Homo sapiens*	Ovarian cancer	Human ovarian cancer cell
SINE resistance OC	GSM3602935	Patient 4	*Homo sapiens*	Ovarian cancer	Human ovarian cancer cell
	GSM3602936	Patient 5	*Homo sapiens*	Ovarian cancer	Human ovarian cancer cell
	GSM3602937	Patient 6	*Homo sapiens*	Ovarian cancer	Human ovarian cancer cell

*GEO, Gene Expression Omnibus; OC, ovarian cancer; SINE, selective inhibitors of nuclear export*.

### Construction of the PPI Network

**Figure 2:**
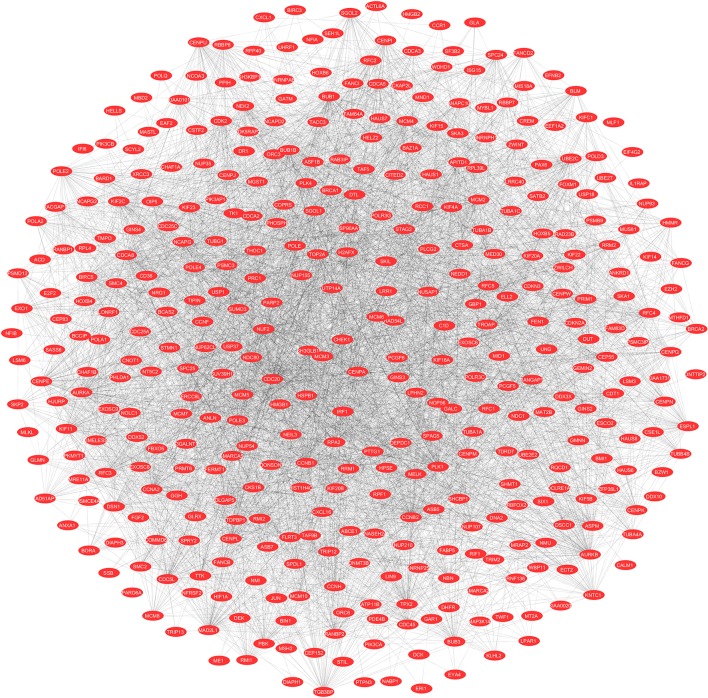
The protein networks of the upregulated DEGs determined using Cytoscape are shown. The representation is as follows: spheres represent the nodes, and the edges are shown as lines.

**Figure 3:**
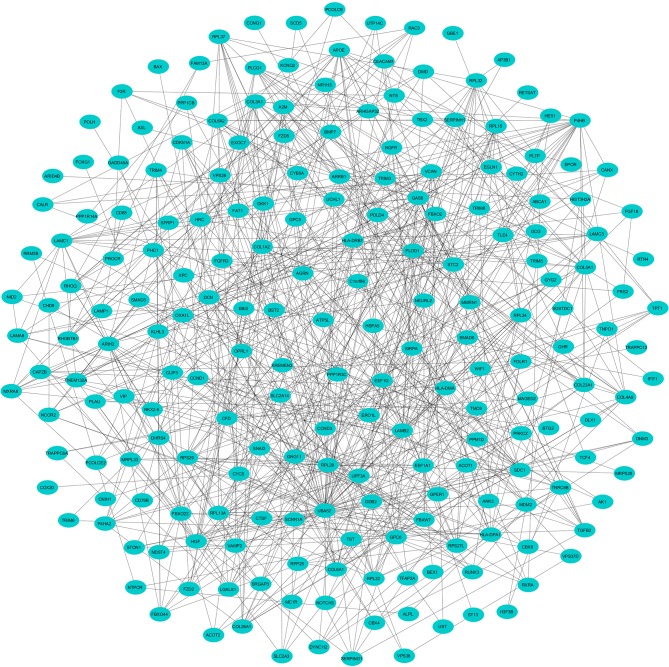
The protein networks of the downregulated DEGs determined using Cytoscape are shown. The representation is as follows: spheres represent the nodes, and the edges are shown as lines.

**Figure 4:**
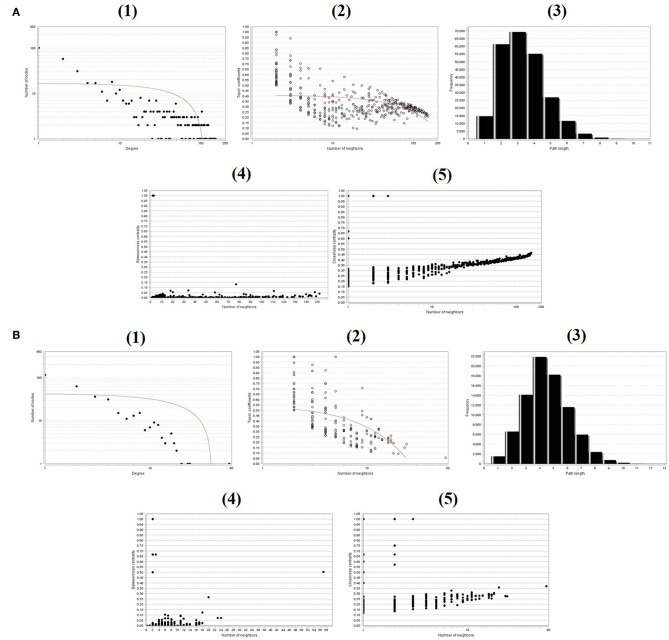
Backbone network topology parameters. **(A)** The network of upregulated DEGs. **(B)** The network of downregulated DEGs. (1) Distribution of the node degree. (2) Topological coefficients. (3) Shortest path length distribution. (4) Betweenness centrality. (5) Closeness centrality.

To evaluate the PPIs between the DEGs, we used the STRING tool to identify the PPI networks for both the up- and downregulated genes. A combined score of ≥0.9 for the nodes was considered to indicate a significant PPI interaction. Then, we exported the resulting PPI network as a “.txt” file and imported it as a.csv file into Cytoscape v3.7.1 software for visualization. The graphical representations of the PPI networks of the up- and downregulated DEGs are shown in Figures 2, 3, respectively. The backbone network of the up- and downregulated genes consist of 794 nodes and 676 nodes with estimated clustering coefficients of 0.321 and 0.192, respectively. The Cytoscape plug-in Network Analyzer was used to analyze the networks for both the up- and downregulated DEGs. Table 2 shows the topological parameters of the up- and downregulated PPI networks, and the topological components, including the distribution of the node degree, the topological coefficient, the shortest path length distribution, the betweenness centrality, and the closeness centrality for the individual PPI networks are shown in Figures 4A,B.

**Table 2:** Topological parameters for the upregulated and downregulated PPI network.

**S. no**.	**Topological parameters**	**Comprehended values**	
		**Upregulated genes**	**Downregulated genes**
(1)	Number of nodes	794	676
(2)	Clustering co-efficient	0.321	0.192
(3)	Network density	0.023	0.003
(4)	Network centralization	0.169	0.081
(5)	Network heterogeneity	1.748	1.893
(6)	Characteristic path length	3.295	4.509
(7)	Average number of neighbors	18.544	2.257

### GO and Enrichment Analysis

To determine the potential GO classifications and KEGG pathway-enriched genes from the dataset, we imported all target DEGs into the online analysis tool DAVID to conduct the annotation process (Supplementary Table 3). The annotated results for the GO terms were divided according to the MF (molecular function), BP (biological process), and CC (cell component) ontologies (*p* < 0.05, FDR < 0.05). The results of the GO biological process (BP) analysis revealed that the upregulated DEGs were mainly enriched in the cell cycle, mitotic cell cycle process, and mitotic nuclear division; the downregulated DEGs were mainly elevated in pathways related to organ morphogenesis, cell development, and cell morphogenesis, which are involved in differentiation, mesenchymal development, and cellular responses to UV. For the GO molecular function analysis, the upregulated DEGs were significantly enriched in nucleoside-triphosphatase activity and hydrolase activity, which acts on acid anhydrides and phosphorus-containing anhydrides, DNA-dependent ATPase activity, and pyrophosphatase activity, whereas the downregulated DEGs were largely enriched in beta-amyloid binding, carbonyl reductase (NADPH) activity, and collagen, amide, and calcium ion binding. Concerning the GO cell component analysis, the upregulated DEGs were mostly enriched in the chromosome and condensed chromosome, while the downregulated DEGs were enriched in membrane-bound vesicles, extracellular vesicles, and the extracellular region and organelles (Supplementary Table 3). Moreover, we used the DAVID online tool to categorize the DEGs involved in various signaling pathways according to the KEGG reference pathways (*p* < 0.05, FDR < 0.05). By examining the KEGG pathways, we noticed that the upregulated DEGs were enriched in DNA replication, the cell cycle, the nucleotide excision repair mechanism, the Fanconi anemia pathway, and DNA mismatch repair; the downregulated DEGs were mostly enriched in the ECM–receptor interaction, the PI3K–Akt signaling pathway, arginine and proline metabolism, Oligodontia-colorectal cancer syndrome, and Nevoid basal cell carcinoma syndrome (Supplementary Table 4).

### Enrichment Analysis Using Metacore™ Software

**Figure 5:**
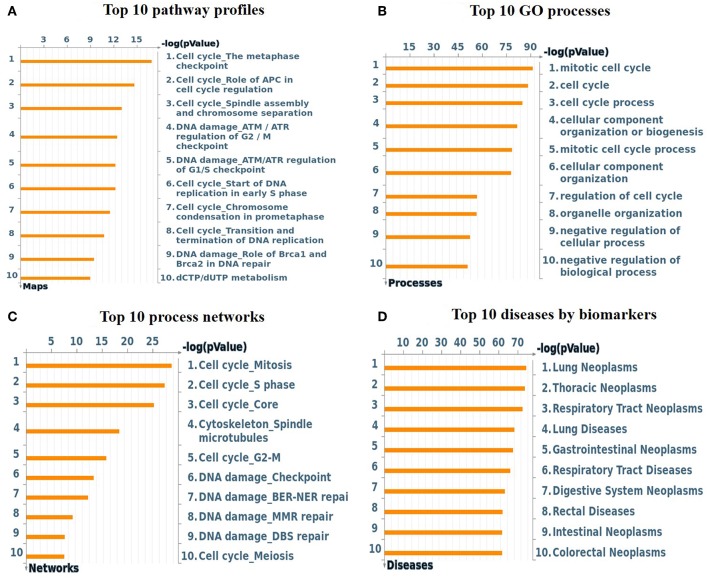
**(A)** Top 10 pathway profiles; **(B)** top 10 GO processes; **(C)** top 10 process networks; **(D)** top 10 diseases according to biomarkers. GeneGo annotation of the top 10 pathway profiles, GO cellular processes, process networks, and diseases according to biomarkers for the DEG datasets. **(A)** The canonical pathway maps represent a set of signaling and metabolic maps comprehensively covering the relevant pathways in humans. **(B)** Gene Ontology (GO) cellular processes. As most GO processes have no gene/protein content, the “empty terms” were excluded from the *p*-value calculations. **(C)** The cellular and molecular processes were defined and annotated; each process represents a preset network of protein interactions characteristic of the process. **(D)** Disease folders (by biomarkers) were organized into a hierarchical tree.

**Figure 6:**
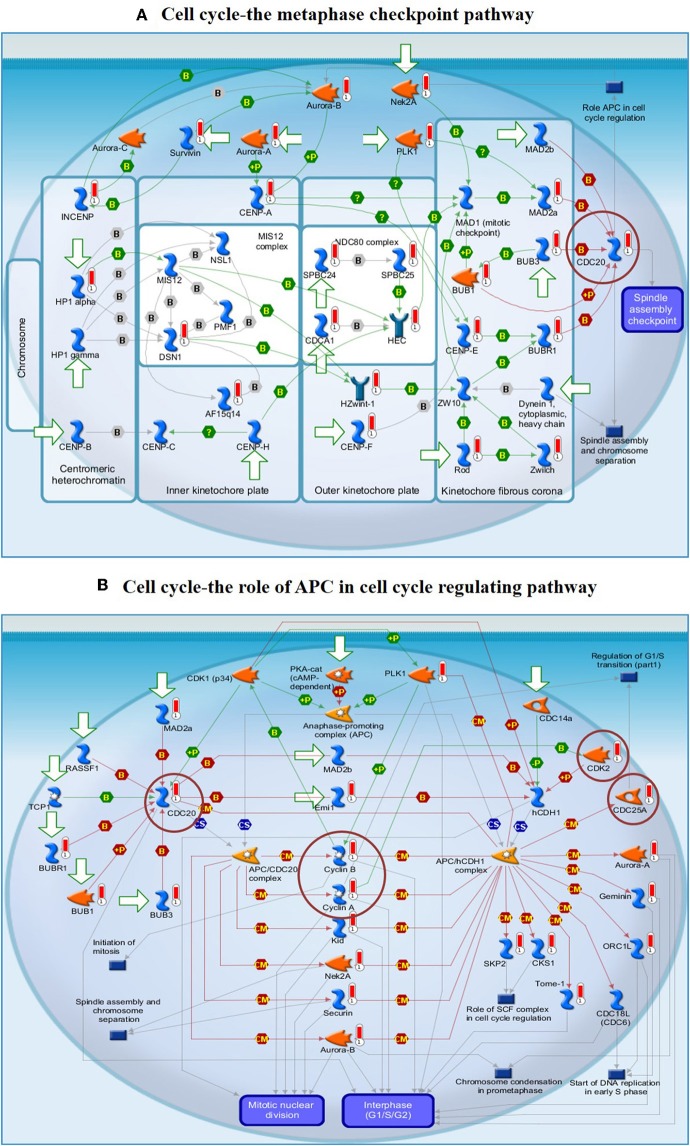
**(A)** The cell cycle metaphase checkpoint pathway. **(B)** The APC cell cycle regulation pathway. The two top-scored regulated pathways were activated in the OC cell lines. The pathway images were generated by GeneGo Metacore™ enrichment analysis. Well-characterized proteins or protein complexes are shown as individual symbols within the image; experimental data from all the records are connected and depicted as thermometer-like figures on the maps. Upward-facing thermometers are shown in red and indicate upregulated gene transcripts. The linkage of proteins by arrows depicts the stimulatory and inhibitory effects or interaction of the encoded protein on the desired protein. The hub genes (protein families) that were involved in the canonical signaling pathways are marked in a circle (scarlet). Further explanations are provided at https://portal.genego.com/help/MC_legend.pdf.

To understand the map pathways and the genes that were differentially expressed in the OC patient cell lines, we used Metacore™ software (Calrivate Analytics) to perform enrichment analysis (EA) using a widely known database for protein–protein signaling. EA identified the gene IDs of the potential targets from the DEG sets with gene IDs via the functional ontology function in MetaCore. The possibility of a random intersection of a gene set and the corresponding ontological entities was determined according to the hypergeometric intersection *p*-value. A reduced *p*-value suggested that the object was more relevant to the dataset, which indicated that it had a higher rating. Comparative enrichment analysis of the DEG dataset identified the top 10 enriched pathways, and the maps, GO cellular processes, networks, and biomarkers (by disease) are shown in Figures 5A–D. These are the most statistically significant data for each category based on a very low *p*-value. The pictorial representation of the top-scored pathway map (lowest *p*-value) is based on the distribution of gene enrichment, as shown in Figure 6A; similarly, the second scored map (second-lowest *p*-value) is shown in Figure 6B. In Figures 6A,B, the well-characterized proteins or protein complexes are displayed as individual symbols; the data from all experiments are shown and linked on the maps as thermometer-like symbols. A red upward-facing thermometer indicates the upregulated genes, and a blue thermometer indicates the expression level of a downregulated gene. The AN algorithm in GeneGo was used to identify the most relevant biological networks by prioritizing the number of fragments of the canonical pathways in the network, as shown in Table 3. The top regulated network processes are presented in Supplementary Figures 1A,B, illustrating the two major pathways involving DEGs that were commonly affected in both OC groups. We identified several crucial hub genes, including *TCF4*, frizzled family proteins (*FZD2, FZD8*, and *FZD6*), *RUNX2*, CDC25 family protein (*CDC25A*), protein kinase family proteins (*CDK2*), *BRCA1, ATM*, and *RBBP8*. The selected hub genes were mainly involved in the regulation of the canonical Wnt signaling pathway, cell–cell signaling mediated by Wnt, cell cycle phase transition, and the positive regulation of the cell cycle (Figure 6, Supplementary Figures 1A,B).

**Table 3:** Most relevant biological networks were generated using GeneGo Analyze Networks (AN) algorithm.

**S. no**.	**Network name**	**Processes**	**Size**	**Target**	**Pathways**	***p*-value**	***z* score**	***g* score**
1	TCF7L2 (TCF4), Tcf(Lef), Frizzled, RUNX2, p21	Canonical Wnt signaling pathway (50.0%), regulation of Wnt signaling pathway (58.0%), cell-cell signaling by Wnt (56.0%), Wnt signaling pathway (56.0%), regulation of canonical Wnt signaling pathway (50.0%)	50	17	262	1.12e-20	21.47	348.97
2	CDC25A, CDK2, Brca1, ATM, RBBP8 (CtIP)	Mitotic cell cycle (57.1%), mitotic cell cycle process (53.1%), mitotic cell cycle phase transition (42.9%), cell cycle phase transition (42.9%), positive regulation of cell cycle (46.9%)	50	32	24	2.61e-49	41.10	71.10
3	KRMP1, FAM83D, LMO1, CBWD1, PSF1	cGMP catabolic process (6.4%), response to macrophage colony-stimulating factor (6.4%), cellular response to macrophage colony-stimulating factor stimulus (6.4%), purine ribonucleotide catabolic process (8.5%), ribonucleotide catabolic process (8.5%)	50	35	0	5.36e-56	45.03	45.03

*In this workflow, the networks were prioritized based on the number of fragments of canonical pathways on the network*.

### Survival Analysis and Expression Levels of Hub Genes

**Figure 7:**
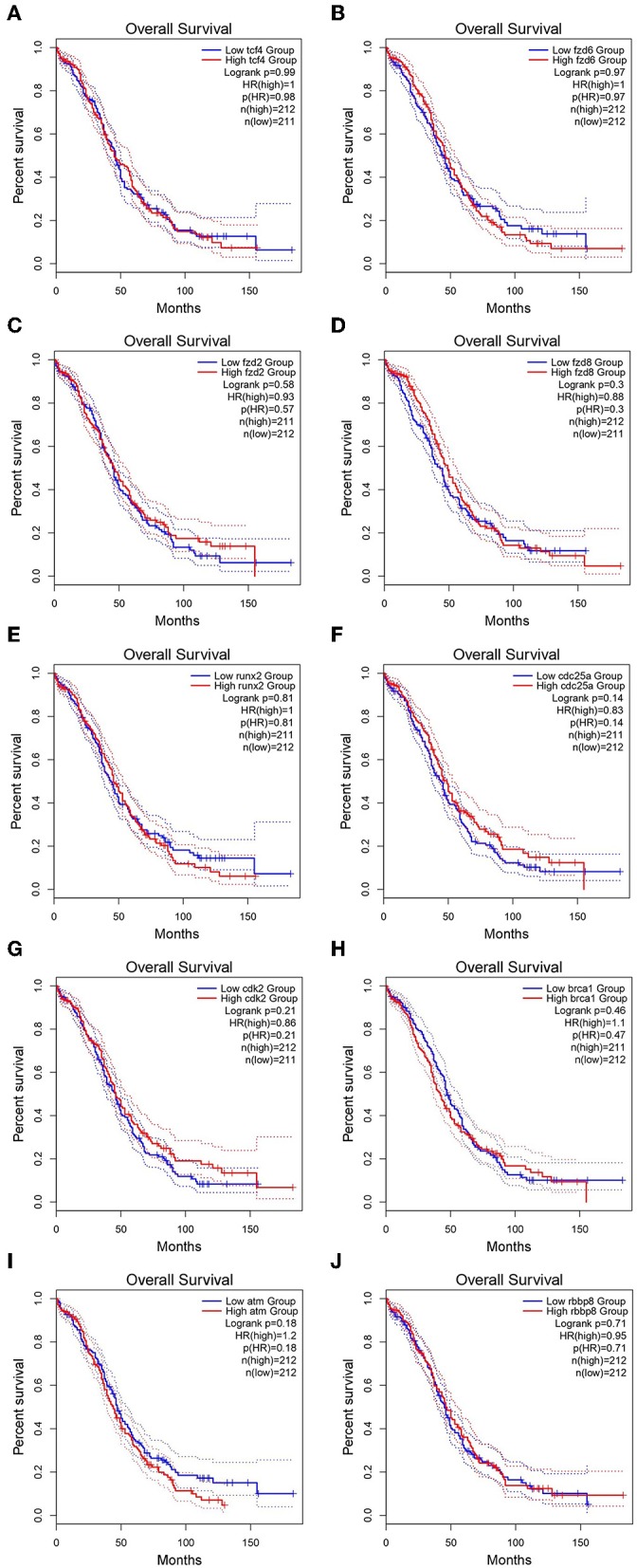
Kaplan–Meier overall survival analysis of the hub genes expressed in OC and SINE-resistant OC cell lines. **(A)** TCF4 (Transcription factor 4), **(B)** *FZD6* (Frizzled class receptor 6), **(C)** *FZD2* (Frizzled class receptor 2), **(D)** *FZD8* (Frizzled class receptor 8), **(E)** *RUNX2* (Runt related transcription factor 2), **(F)** *CDC25A* (Cell division cycle 25A), **(G)** *CDK2* (Cyclin-dependent kinase 2), **(H)** *BRCA1* (Breast cancer type 1 susceptibility protein), **(I)** *ATM* (ATM serine/threonine kinase), and **(J)** *RBBP8* (Retinoblastoma-binding protein 8). The survival curves were plotted using the GEPIA2 web server. The genes with high expression in the cohorts are shown in red, and the blue line indicates the low-expression cohort. The survival curves are represented as dotted lines, and the solid line represents the 95% confidence interval. HR stands for hazard ratio; patient number (*n*) = 212. The *p*-values were calculated using log-rank statistics.

**Figure 8:**
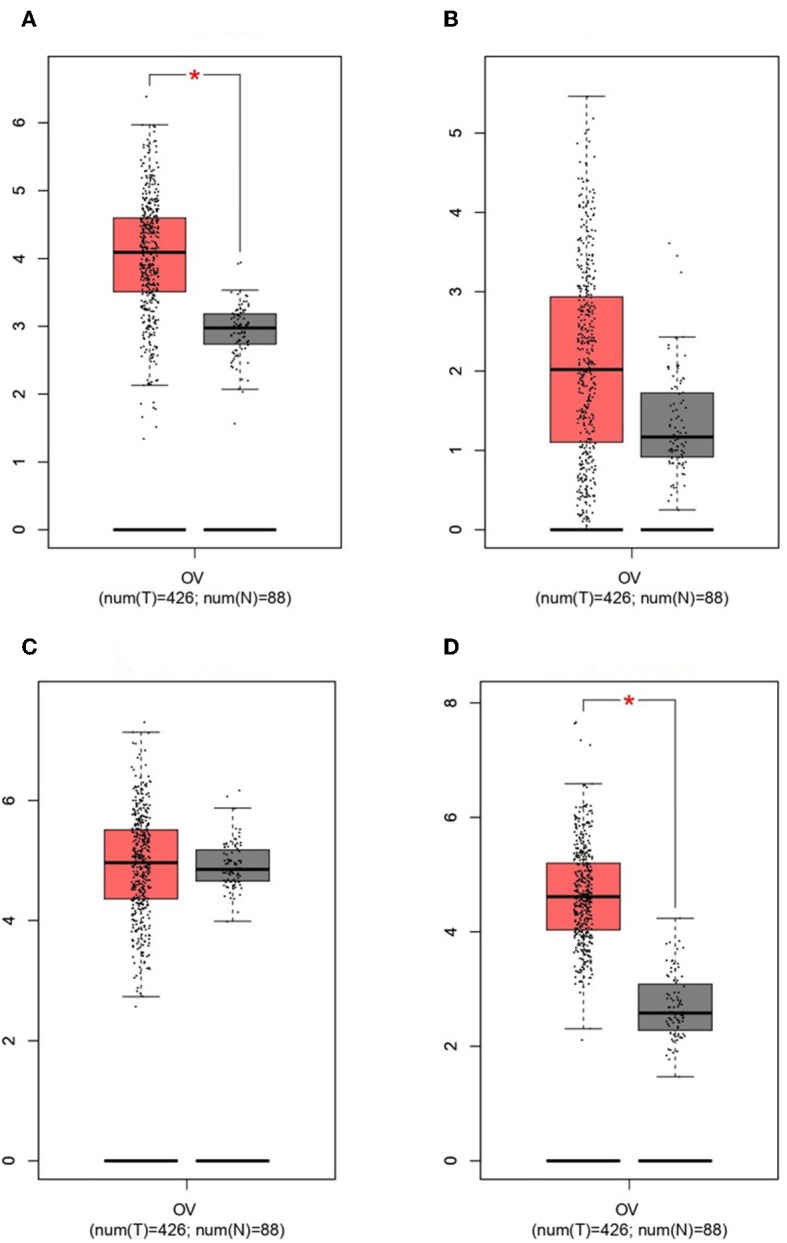
Based on TCGA and GTEx data in GEPIA, we validated the expression levels of the four core genes in ovarian cancer (*n* = 426) and normal tissues (*n* = 88). **(A)** *FZD6*. **(B)** *FZD8*. **(C)** *CDK2*. **(D)** *RBBP8*.

GEPIA survival assessment was used to investigate the overall association with survival of 10 hub genes from both the low- and high-expression OC groups. As a result, we noticed that the high expression of FZD2 (HR = 0.93) (Figure 7C), FZD8 (HR = 0.88) (Figure 7D), CDC25A (HR = 0.83) (Figure 7F), CDK2 (HR = 0.86) (Figure 7G), and RBBP8 (HR = 0.95) (Figure 7J) were associated with improved overall survival in the OC cell line. However, the high expression of TCF4 (HR = 1) (Figure 7A), FZD6 (HR = 1) (Figure 7B), RUNX2 (HR = 1) (Figure 7E), BRCA1 (HR = 1.1) (Figure 7H), and ATM (HR = 1.2) (Figure 7I) were linked with worse overall survival in the OC cell line. Taken together, the results show that *FZD6, FZD8, CDK2*, and *RBBP8* function as core genes that have a close relationship with OC. Furthermore, the GEPIA box plot analysis investigated the level of expression of the core genes in 426 OC tissue samples and 88 normal tissue samples. The boxplot in Figures 8A–D shows a considerable increase in the level of core gene expression (*FZD6, FZD8, CDK2*, and *RBBP8*) in the OC cell line.

## Discussion

Microarray technology is one of the most important approaches used by many researchers worldwide to explore the expression levels of genes involved in complex disorders (Russo et al., 2003; Babu, 2004; Perez-Diez et al., 2013). Therefore, studying the expression profiles of DEGs and predicting the target genes of OC is of the utmost importance. In this study, data from a total of three OC cell lines and three OC cell lines with SINE resistance were obtained from the GEO database (GSE126519). A total of 2708 DEGs were screened, including 809 upregulated and 700 downregulated genes. *In silico* methods have typically shown good efficiency, and networks have been demonstrated to be a reliable way to depict genomic data. The topological interpretation of upregulated and downregulated genes is required for large PPI networks and is thus substantially based on integrated local components, such as the distribution node of the degree, the topological coefficient, the shortest path length distribution, and the betweenness and closeness centralities (Assenov et al., 2008). These parameters were used to analyze the nodes in individual PPI networks of the DEG dataset to deduce their significance in networks with different characteristics. Furthermore, we implemented GO and KEGG pathway analyses to determine MF, BP, CC, and pathways involving the DEGs using the DAVID online tool. The GO BP terms and KEGG assessment indicated that the upregulated DEGs were enriched primarily in the cell cycle, mitotic cell cycle process, mitotic nuclear division, DNA replication, cell cycle, nucleotide excision repair, DNA mismatch repair, and Fanconi anemia pathways. Interestingly, mutations in mismatch repair (MMR) and Fanconi anemia pathway-related genes in women have been shown to be one of the primary causes of hereditary OC (Norquist et al., 2016). Therefore, our observed results are consistent with the role of upregulated genes in pathways that cause OC. Similarly, the downregulated DEGs were mainly enriched in organ morphogenesis, cell development, cell morphogenesis, mesenchymal development and the interaction of the ECM receptor, PI3K-Akt signaling, and arginine and proline metabolism pathways. In line with this, a significant cause of cancer would appear to be the abnormal functioning of the cell cycle and mitosis (Kastan and Bartek, 2004; Malumbres and Barbacid, 2009).

The findings from the STRING, Cytoscape, GO, and KEGG analyses indicated that many pathways were primarily affected in OC. Several studies have used Cytoscape plugins such as MCODE, cytoHubba, CytoCluster, CytoKegg, and CytoNCA to elucidate the core interactions in PPI networks (Lan et al., 2015; Villaveces et al., 2015; Sriroopreddy and Sudandiradoss, 2018; Zhang et al., 2019). To delineate the molecular interactions and pathways identified from the STRING, GO, and KEGG analyses, we utilized GeneGo Metacore, which has a massive amount of information about regulatory and metabolic pathways and contains precisely curated biological networks. To obtain a comprehensive picture of the DEGs involved in OC, we used GeneGo Metacore™ software to identify the most significant genes and signaling pathways based on the calculated *p*-values. Among the top 10 enriched pathways, the cell cycle metaphase checkpoint, APC in cell cycle regulation, chromosome separation, spindle assembly, and DNA damage/ATR regulation of G2/M phase checkpoint/ATM were highly significant in the DEG datasets from both groups (Figure 5A). The GO cellular processes showed that the DEGs were enriched in a variety of cellular processes (Figure 5B), and these processes are mainly utilized in the enrichment analysis and to prioritize the genes in the constructed networks. The GO process networks were enriched in various groups. Among the top 10 process networks, we selected the four that were the most significant based on the calculated *p*-values, which included the mitotic cell cycle, the S phase in the cell cycle, and cytoskeleton-spindle microtubules (Figure 5C). The biomarkers of the diseases distinctly showed that the DEGs with the highest representation in the dataset were also known to contribute to other cancer types (Figure 5D). Additionally, there were two top-scored regulated pathways that were activated in the OC cell line that were involved in the cell cycle: the metaphase checkpoint and APC cell cycle regulation pathways (Figure 6). Furthermore, we analyzed the biological network of the upregulated DEGs in the signaling pathways by utilizing the AN algorithm in GeneGo. As a result, we determined the two most significant networks that were commonly affected in both of the OC groups. The components of these networks included several crucial hub genes, including *TCF4*, the frizzled family proteins (*FZD2, FZD8*, and *FZD6*), *RUNX2*, a CDC25 family protein (*CDC25A*), the protein kinase family proteins (*CDK2*), *BRCA1, ATM*, and *RBBP8*. Among them, the *TCF4, frizzled*, and *RUNX2* genes are primarily involved in the regulation of the canonical WNT signaling pathway (58%) and cell–cell signaling mediated by WNT (56%). Genes such as *CDC25A, CDK2, BRCA1, ATM*, and *RBBP8* are mostly involved in the mitotic cell cycle process (53.1%), mitotic cell cycle phase transition (42.9%), positive regulation of the cell cycle (46.9%), and cell cycle phase transition (42.9%). Finally, the GEPIA web server was used to assess the association between hub gene expression and OC prognosis. The overall survival analysis indicated that high expression of *FZD2, FZD8, CDC25A, CDK2*, and *RBBP8* were associated with better survival, and high expression of *TCF4, FZD6, RUNX2, BRCA1*, and *ATM* were associated with decreased survival in the OC cell line. Collectively, *FZD6, FZD8, CDK2*, and *RBBP8* were identified as core genes that were strongly associated with overall survival in OC. Therefore, these four genes could contribute to OC metastasis.

Supplementary Figure 1A shows that the frizzled family of proteins is involved in canonical Wnt signaling pathway regulation. FZD6, also known as frizzled class receptor 6, is a member of the “frizzled” gene family, which consists of 7-transmembrane domain proteins that are Wnt signaling protein receptors. Many studies have observed through mutagenesis experiments that several residues in the intracellular loops and the C-terminus of FZD play a prominent role in signaling (Cong et al., 2004; Wallingford and Habas, 2005). Kim et al. (2015) found that the expression of FZD6 was increased in colorectal cancer (CRC) patients when compared to that in nontumor tissues. Furthermore, they discovered that FZD6 expression in CRC cells was negatively regulated by miR199a-5p (Kim et al., 2015). In recent research, Corda et al. (2017) observed that the Wnt receptor-encoding gene *FZD6* is often duplicated in breast cancer and confers a higher risk of triple-negative breast cancer. For the assembly of the fibronectin matrix, FZD6 signaling is intrinsically required and interferes with actin cytoskeletal organization. The researchers concluded that in highly metastatic forms of breast cancer, such as TNBC, the FZD6-fibronectin actin axis could be targeted for drug treatment (Corda et al., 2017). In our study, we observed the overexpression of frizzled class receptor 6 in OC cell lines, and the overexpression of FZD6, which acts as an adverse prognostic factor, was associated with decreased survival in OC patients.

Frizzled class receptor 8 is also a “frizzled” gene family member that serves as a Wnt signaling protein receptor (Bhanot et al., 1996). Most frizzled receptors are also associated with the canonical signaling pathway of beta-catenin (Dann et al., 2001). Li et al. (2017) reported a higher level of FZD8 expression in bone metastases in prostate cancer (PCa), which is frequently diagnosed among men. This research group also found that the silencing of FZD8 suppressed the migration and invasion of cells and the occurrence of PCa bone metastasis *in vitro* and *in vivo* by activating the canonical β-catenin/Wnt signaling pathway, and the data suggest that FZD8 could be a potential therapeutic target for the treatment of bone metastasis in PCa (Li et al., 2017). Chakravarthi et al. (2018) reported that ETS-related gene (ERS) specifically targets and activates FZD8 directly by binding to its promoter region rather than ETV1 and suggested that the overexpression of ERG in PCa leads to FZD8 induction and the activation of the Wnt pathway (Chakravarthi et al., 2018). The research group led by He et al. recently found that miR-520b overexpression results in the inhibition of cell proliferation, migration, and invasion in human spinal osteosarcoma (OS) tissues and cell lines by inactivating the Wnt/β-catenin signaling pathway through the downregulation of FZD8 and thus provides a new spinal OS therapeutic target (Wang et al., 2017). Similarly, Liu et al. (2019) reported a reduced level of miR-99b-5p in non-small cell lung cancer (NSCLC) cell lines. They validated FZD8 as a specific target of miR-99b-5p and found that increased expression of miR-99b-5p inhibited NSCLC proliferation, migration, and invasion *in vitro* (Liu et al., 2019). The findings of our study suggest that the overexpression of FZDs in OC results in the anomalous activation of the canonical Wnt signaling pathway and may increase their function during the development of OC.

As seen in Figure 6B, CDK2 is mainly involved in the APC cell cycle regulation pathway, and the overexpression of CDK2 results in the upregulation of the G1/S phase transition, resulting in cancer cell proliferation. CDK2 and other relevant genes that are upregulated in OC (red circles in Supplementary Figure 1B) are shown in the closed network of the APC cell cycle regulation pathway; because of the increase in CDKs, APC failed to inactivate the CDK complexes by inducing their degradation. Liu et al. (2011) revealed that CDK2 expression was significantly higher in laryngeal squamous cell cancer tissues when compared to that in paired adjacent normal laryngeal tissues (Liu et al., 2011). Duong et al. (2012) reported that low-molecular-weight cyclin E (LMW-E) required kinase activity associated with CDK2 to induce the formation of mammary tumors by disrupting the growth of acinar cells. They used a combination of therapy with a CDK inhibitor (roscovitine) plus a b-Raf-targeting pan-kinase inhibitor (sorafenib) or an mTOR inhibitor (rapamycin) to arrest the G1/S cell cycle in breast cancer cells; thus, the b-Raf-ERK1/2-mTOR signaling pathway could be suppressed (Duong et al., 2012). Kanwal et al. (2016) found that the expression of CDK2 is significantly increased in OC tissues when compared to that in normal ovarian tissues (Kanwal et al., 2016). The pathways involving cyclin-dependent kinase (CDK) are significant and well-established cancer treatment targets. The role of CDK2 remains controversial in several cancer types (McCurdy et al., 2017). Many studies have suggested that CDK2 could be a crucial factor in the progression of cancer by regulating several pathways and might be a prospective biomarker and indicator of prognosis. Therefore, CDK2 and its cyclin binding partners are possible therapeutic targets for future cancer treatments (Yin et al., 2018; Zhang et al., 2018; Wood et al., 2019).

RBBP8, also known as retinoblastoma-binding protein 8, aids in regulating cell proliferation and DNA repair by homologous recombination (Fusco et al., 1998). Soria-Bretones et al. (2013) observed decreased or no expression of *RBBP8* in paraffin-embedded breast cancer biopsy tissues from high-grade breast cancer and nodal metastases that were acquired during tumor removal surgery (Soria-Bretones et al., 2013). The research group led by Rose et al. suggested that *RBBP8* was significantly hypermethylated in bladder cancer (BLCA) and was associated with more prolonged overall survival, and they indicated that it may be used as a complementary marker for the detection of BLCA in urine (Mijnes et al., 2018). Miao et al. (2019) reported that the downregulation of the long noncoding RNA (lncRNA) cancer susceptibility candidate 2 (CASC2, enhanced tumor development, increased miR-18a-5p levels, and reduced the expression of *RBBP8* in nasopharyngeal carcinoma (NPC). The upregulation of CASC2 resulted in decreased proliferation and increased apoptotic cell death *in vivo* (Miao et al., 2019). Our data clearly showed that *RBBP8* was differentially expressed and involved in cell cycle regulation (Supplementary Figure 1B). Additionally, it contributes to the development of OC in both groups. However, the role of *RBBP8* in OC is unclear and requires further research.

**Figure 9:**
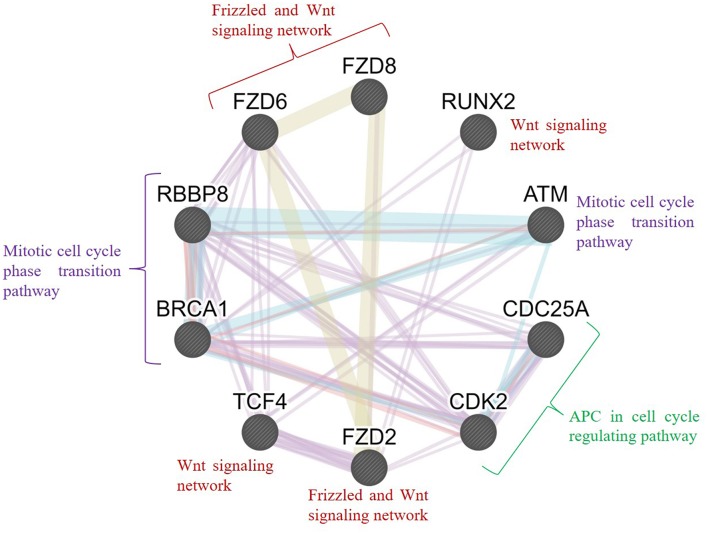
Interrelation analysis of the hub genes identified from different pathways. GeneMANIA was used to plot the network, which was visualized in Cytoscape. Color code: physical interaction shown in red, coexpression shown in violet, predicted interaction shown in orange, common pathway shown in cyan, and colocalization shown in blue. The genes *FZD2, FZD6, FZD8, RUNX2*, and *TCF4* were involved in the frizzled and Wnt signaling network; *BRCA1, RBBP8*, and *ATM* were involved in the mitotic cell cycle phase transition pathway; and *CDC25A* and *CDK2* were involved in the APC cell cycle regulation pathway.

Furthermore, we conducted an interrelation analysis of the identified hub genes to elucidate the interactions between them, primarily among the genes that interacted with one another directly or indirectly. As shown in Figure 9, the mitotic cell cycle phase transition pathway interacts with the regulation of G1/S phase transition and APC in the cell cycle regulating pathway via the essential genes *RBBP8, BRCA1, CDC25A, ATM*, and *CDK2* (D'Andrilli et al., 2004; Soria-Bretones et al., 2013; Xiao et al., 2019). In contrast, frizzled family proteins (*FZD2, FZD6*, and *FZD8*) are directly involved in the Wnt signaling pathways because they are receptors of Wnt proteins (Janda et al., 2012). The *TCF4* and *RUNX2* genes are involved directly or indirectly in the Wnt signaling network, resulting in tumorigenesis (Gaur et al., 2005; Hrckulak et al., 2018; Komori, 2019). Taken together, these findings showed that node genes involved in the development of OC could be significant factors in cell cycle regulation and the Wnt signaling pathway.

Overall, our systematic bioinformatics assessment demonstrated that DEGs might play a pivotal role in the incidence, prognosis, growth, and development of OC. In this study, a total of 2708 DEGs and 10 hub genes were identified, and *FZD6, FZD8, CDK2*, and *RBBP8* could be the core genes involved in OC and SINE-resistant OC. Expression analysis and the correlation of the multiple genes will undoubtedly aid in the understanding of the roles of such genes in the growth and development of OC. Several research groups have demonstrated that preclinical models have showed some success in reducing tumor growth and decreasing the side effects of existing chemotherapy drugs (Cicenas et al., 2015; Whittaker et al., 2017; Xia et al., 2018). We need to conduct a series of experimental studies to prove this hypothesis to obtain more precise correlation reports. However, the findings from this study could enhance the understanding of the molecular pathogenesis of OC. Furthermore, the core genes and pathways might be potential biomarkers that could be used for the detection and targeting of OC and SINE-resistant OC cells for therapy.

## Data Availability Statement

All datasets generated for this study are included in the article/Supplementary Material.

## Author Contributions

SK, DK, and CD were involved in the design of the study and the acquisition, analysis, and interpretation of the data. SK, DK, CD, and HZ were involved in the interpretation of the data and drafting the manuscript. CD, RS, and HZ supervised the entire study and were involved in study design, the acquisition, analysis, interpretation of the data, and drafting the manuscript. The manuscript was reviewed and approved by all the authors.

### Conflict of Interest

The authors declare that the research was conducted in the absence of any commercial or financial relationships that could be construed as a potential conflict of interest.

## Footnotes

^1^MetaCore Login | Clarivate Analytics Available at: https://portal.genego.com/ (accessed June 22, 2019).
