# Supplementary material for: Integrative Bioinformatics Approaches to Map Potential Novel Genes and Pathways Involved in Ovarian Cancer
Source: Front Bioeng Biotechnol. 2019 Dec 17;7:391. doi: 10.3389/fbioe.2019.00391 (PMC6927934; doi:10.3389/fbioe.2019.00391)
Supplement: Supplementary file 1 [file Data_Sheet_1.pdf]

Supplementary Material

Supplementary Figure 1A & B

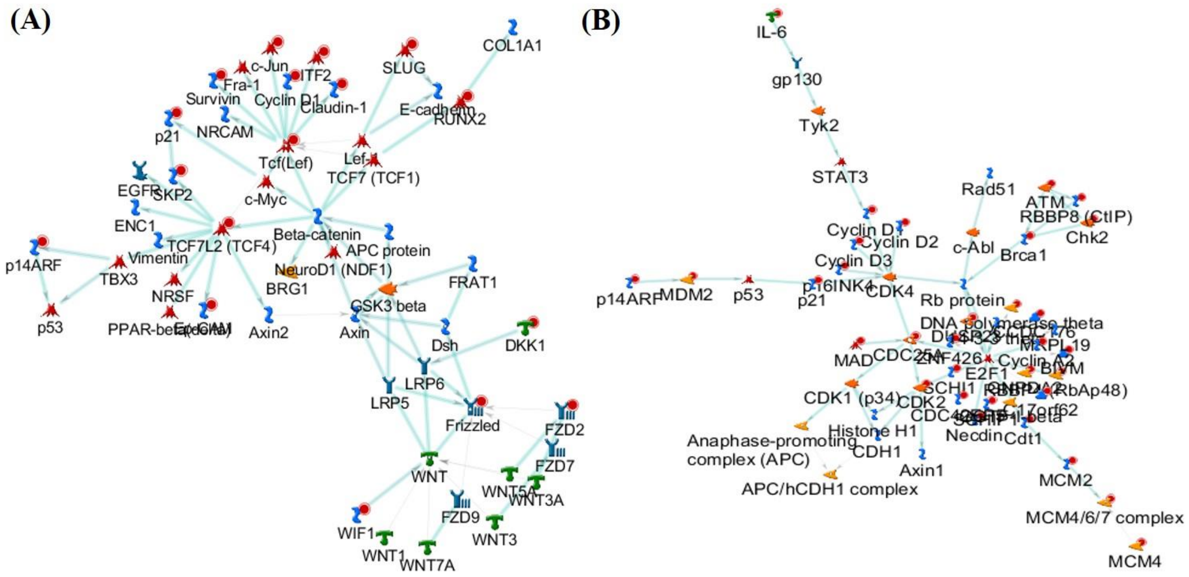

Supplementary Figure 1. Identification of the top regulated network processes using the Analyze Networks (AN) algorithm in GeneGo. Comparative enrichment analysis of the disease groups revealed two commonly affected pathways: (A) the top-scored biological network of OC and (B) the second most-highly scored biological network of OC. The fragments of the canonical pathways are shown as thick cyan lines. Upregulated genes are labeled with red circles; blue circles label downregulated genes. A checkerboard pattern indicates the mixed expression of the gene in different files or according to multiple tags for the same gene.

Supplementary Table 1. The table displays all the differentially expressed genes from two groups of GSE126519 dataset using GEO2R tool.

Gene Symbol	Gene Title	P-Value	logFC
GDF15	growth differentiation factor 15	5.20E-12	-5.01
SDPR	serum deprivation response	6.05E-12	6.74
ITM2A	integral membrane protein 2A	2.09E-11	-5.61
S100A4	S100 calcium binding protein A4	3.24E-11	-5.53
DKK1	dickkopf WNT signaling pathway inhibitor 1	3.27E-11	-6.57
NCAPG	non-SMC condensin I complex subunit G	3.31E-11	4.03
NABP1	nucleic acid binding protein 1	3.49E-11	4.41
HAPLN1	hyaluronan and proteoglycan link protein 1	4.18E-11	-4.6
NEK2	NIMA related kinase 2	4.53E-11	4.44
EPDR1	ependymin related 1	5.89E-11	3.49
HAPLN1	hyaluronan and proteoglycan link protein 1	6.04E-11	-4.32
NUP62CL	nucleoporin 62 C-terminal like	7.29E-11	3.63
TCEAL8	transcription elongation factor A like 8	9.78E-11	-4.44
PBK	PDZ binding kinase	1.04E-10	3.77
CDKN1A	cyclin dependent kinase inhibitor 1A	1.14E-10	-6.08
CLDN1	claudin 1	1.19E-10	5.33
IGFBP3	insulin like growth factor binding protein 3	1.21E-10	6.44
DLGAP5	DLG associated protein 5	1.87E-10	3.69
ATP1B1	ATPase Na+/K+ transporting subunit beta 1	1.89E-10	5.5
MAP1LC3A	microtubule associated protein 1 light chain 3 alpha	2.01E-10	-4.45
EPCAM	epithelial cell adhesion molecule	2.04E-10	3.95
VIP	vasoactive intestinal peptide	2.09E-10	-4.61
FAM9C	family with sequence similarity 9 member C	2.25E-10	3.8
GALC	galactosylceramidase	2.26E-10	2.74
PLBD1	phospholipase B domain containing 1	2.39E-10	-3.41

TM4SF1	transmembrane 4 L six family member 1	2.42E-10	4.88
TMEM30B	transmembrane protein 30B	2.64E-10	3.64
CDCA5	cell division cycle associated 5	2.64E-10	3.57
GBP1	guanylate binding protein 1	2.72E-10	4.16
OIP5	Opa interacting protein 5	2.89E-10	3.46
ACTG2	actin, gamma 2, smooth muscle, enteric	2.95E-10	3.1
LHX8	LIM homeobox 8	2.96E-10	-3.32
SLC16A14	solute carrier family 16 member 14	2.97E-10	3.24
RGS16	regulator of G-protein signaling 16	3.10E-10	-3.98
RAB3IP	RAB3A interacting protein	3.19E-10	3.05
CDC45	cell division cycle 45	3.19E-10	3.77
MCM10	minichromosome maintenance 10 replication initiation factor	3.28E-10	3.71
DTNA	dystrobrevin alpha	3.38E-10	2.96
RBM24	RNA binding motif protein 24	3.40E-10	3.32
NTS	neurotensin	3.98E-10	-5.74
ISL1	ISL LIM homeobox 1	3.99E-10	5.72
HMMR	hyaluronan mediated motility receptor	4.08E-10	3.18
MELK	maternal embryonic leucine zipper kinase	4.34E-10	3.16
POSTN	periostin	4.36E-10	5.47
CEP55	centrosomal protein 55	4.52E-10	4.1
KERA	keratocan	4.82E-10	-2.84
QPCT	glutaminyl-peptide cyclotransferase	5.53E-10	-2.97
TPD52L1	tumor protein D52-like 1	5.54E-10	3.32
EPCAM	epithelial cell adhesion molecule	5.78E-10	4.08
AXL	AXL receptor tyrosine kinase	5.81E-10	-3.21
CD36	CD36 molecule	5.95E-10	3.27
S100A4	S100 calcium binding protein A4	5.98E-10	-5.64
IGFBP3	insulin like growth factor binding protein 3	6.16E-10	5.36
EFHD1	EF-hand domain family member D1	6.23E-10	-2.46

MID1IP1	MID1 interacting protein 1	6.28E-10	-2.93
LPAR1	lysophosphatidic acid receptor 1	6.73E-10	2.63
ARHGAP28	Rho GTPase activating protein 28	7.06E-10	2.67
C8orf4	chromosome 8 open reading frame 4	7.20E-10	-4.36
MID1	midline 1	7.26E-10	4.72
CCNO	cyclin O	7.63E-10	3.02
PTPRD	protein tyrosine phosphatase, receptor type D	8.01E-10	-3.05
SNAI2	snail family transcriptional repressor 2	8.01E-10	-2.46
NRG1	neuregulin 1	8.02E-10	4.1
NUSAP1	nucleolar and spindle associated protein 1	8.19E-10	4.02
TMEFF2	transmembrane protein with EGF like and two follistatin like domains 2	8.22E-10	3.44
P3H2	prolyl 3-hydroxylase 2	8.52E-10	4.76
DDIAS	DNA damage induced apoptosis suppressor	8.69E-10	2.44
POSTN	periostin	9.36E-10	5.21
TMEM98	transmembrane protein 98	9.61E-10	2.65
B3GALNT1	beta-1,3-N-acetylgalactosaminyltransferase 1 (globoside blood group)	1.00E-09	2.21
PRTFDC1	phosphoribosyl transferase domain containing 1	1.02E-09	2.54
ALDH2	aldehyde dehydrogenase 2 family (mitochondrial)	1.07E-09	-2.46
FLRT3	fibronectin leucine rich transmembrane protein 3	1.12E-09	3.79
EXO1	exonuclease 1	1.14E-09	3.17
MMP3	matrix metallopeptidase 3	1.25E-09	4.21
DLGAP5	DLG associated protein 5	1.29E-09	3.58
CITED2	Cbp/p300 interacting transactivator with Glu/Asp rich carboxy-terminal domain 2	1.32E-09	2.91
PDE4C	phosphodiesterase 4C	1.43E-09	-3.65
ARHGAP28	Rho GTPase activating protein 28	1.53E-09	2.96

TWIST1	twist family bHLH transcription factor 1	1.53E-09	-4.01
CCNA2	cyclin A2	1.62E-09	3.66
RAD51AP1	RAD51 associated protein 1	1.62E-09	3.41
SYNE4	spectrin repeat containing nuclear envelope family member 4	1.66E-09	2.63
MDK	midkine (neurite growth-promoting factor 2)	1.67E-09	4.37
UCHL1	ubiquitin C-terminal hydrolase L1	1.69E-09	-4.05
LIN28A	lin-28 homolog A	1.85E-09	-3.21
ANKRD1	ankyrin repeat domain 1	1.91E-09	4.48
SGCE	sarcoglycan epsilon	1.91E-09	2.87
MARCKS	myristoylated alanine rich protein kinase C substrate	1.95E-09	2.6
DKK3	dickkopf WNT signaling pathway inhibitor 3	1.96E-09	-2.2
RGS20	regulator of G-protein signaling 20	2.11E-09	3.3
PCDH20	protocadherin 20	2.27E-09	-4.09
FANCD2	Fanconi anemia complementation group D2	2.29E-09	2.88
ITM2A	integral membrane protein 2A	2.33E-09	-4
TYRP1	tyrosinase related protein 1	2.39E-09	-4.73
SKA3	spindle and kinetochore associated complex subunit 3	2.39E-09	2.27
HMMR	hyaluronan mediated motility receptor	2.53E-09	3.06
AUNIP	aurora kinase A and ninein interacting protein	2.78E-09	2.18
ALX1	ALX homeobox 1	2.79E-09	-2.71
PKIA	protein kinase (cAMP-dependent, catalytic) inhibitor alpha	2.79E-09	2.18
KIF14	kinesin family member 14	2.81E-09	2.18
CGNL1	cingulin like 1	2.91E-09	2.28
EOMES	eomesodermin	3.03E-09	-3.23
ALX1	ALX homeobox 1	3.04E-09	-2.96
SCHIP1	schwannomin interacting protein 1	3.21E-09	2.29
KIF4A	kinesin family member 4A	3.24E-09	2.14

KCNJ16	potassium voltage-gated channel subfamily J member 16	3.28E-09	3.01
C9orf3	chromosome 9 open reading frame 3	3.36E-09	2.33
POLE2	DNA polymerase epsilon 2, accessory subunit	3.41E-09	2.38
PRIM1	primase (DNA) subunit 1	3.50E-09	2.73
HJURP	Holliday junction recognition protein	3.60E-09	2.32
LRRTM4	leucine rich repeat transmembrane neuronal 4	3.65E-09	2.9
SGO1	shugoshin 1	3.92E-09	2.33
MMP1	matrix metallopeptidase 1	4.00E-09	4.26
CHODL	chondrolectin	4.01E-09	2.52
FAM110D	family with sequence similarity 110 member D	4.19E-09	-3.04
SLF1	SMC5-SMC6 complex localization factor 1	4.24E-09	3.14
AP1S2	adaptor related protein complex 1 sigma 2 subunit	4.31E-09	2.51
TNFRSF19	TNF receptor superfamily member 19	4.36E-09	4.17
SLC16A14	solute carrier family 16 member 14	4.73E-09	2.4
LRRN3	leucine rich repeat neuronal 3	4.85E-09	2.71
PCOLCE	procollagen C-endopeptidase enhancer	4.91E-09	-3.67
CCDC138	coiled-coil domain containing 138	5.03E-09	3.08
PLPPR4	phospholipid phosphatase related 4	5.05E-09	-3.72
UBE2E2	ubiquitin conjugating enzyme E2	5.07E-09	3.27
TPD52L1	tumor protein D52-like 1	5.15E-09	3.44
FXYD5	FXYD domain containing ion transport regulator 5	5.27E-09	3.55
MT1F	metallothionein 1F	5.28E-09	2.31
PLPP7	phospholipid phosphatase 7 (inactive)	5.39E-09	-1.89
CKB	creatine kinase B	5.53E-09	-2.34
RNLS	renalase, FAD dependent amine oxidase	5.56E-09	3.02
ERI1	exoribonuclease 1	5.74E-09	2.69
KIF20B	kinesin family member 20B	5.79E-09	2.07

KCNJ2	potassium voltage-gated channel subfamily J member 2	6.37E-09	3.49
SPOCK2	sparc/osteonectin, cwcv and kazal-like domains proteoglycan (testican) 2	6.61E-09	4.46
NAP1L3	nucleosome assembly protein 1 like 3	6.68E-09	-4.1
LGALS8	galectin 8	6.72E-09	1.98
SCN9A	sodium voltage-gated channel alpha subunit 9	6.92E-09	2.55
CDC25C	cell division cycle 25C	7.02E-09	1.8
OGFRL1	opioid growth factor receptor like 1	7.27E-09	2.51
BIRC3	baculoviral IAP repeat containing 3	7.43E-09	2.11
ATP1B1	ATPase Na+/K+ transporting subunit beta 1	7.79E-09	3.19
MGST1	microsomal glutathione S-transferase 1	8.07E-09	3.35
PKIA	protein kinase (cAMP-dependent, catalytic) inhibitor alpha	8.09E-09	1.91
MELK	maternal embryonic leucine zipper kinase	8.17E-09	2.68
NOTCH3	notch 3	8.60E-09	-2.71
DSN1	DSN1 homolog, MIS12 kinetochore complex component	8.77E-09	2
NDC80	NDC80, kinetochore complex component	8.88E-09	3.1
FAM72D	family with sequence similarity 72 member D	8.91E-09	2.46
MYO1B	myosin IB	8.94E-09	3.83
MCM8	minichromosome maintenance 8 homologous recombination repair factor	9.10E-09	1.66
SH3KBP1	SH3 domain containing kinase binding protein 1	9.26E-09	1.73
ASB5	ankyrin repeat and SOCS box containing 5	9.54E-09	2.9
RNLS	renalase, FAD dependent amine oxidase	9.62E-09	1.87
KIF11	kinesin family member 11	9.79E-09	2.42
MYOC	myocilin	1.01E-08	-2.36
GSTM2	glutathione S-transferase mu 2 (muscle)	1.03E-08	-2.53
CPQ	carboxypeptidase Q	1.08E-08	1.62

WDR63	WD repeat domain 63	1.13E-08	-2.9
GPER1	G protein-coupled estrogen receptor 1	1.14E-08	-1.99
CCNB1	cyclin B1	1.14E-08	2.09
PLPPR5	phospholipid phosphatase related 5	1.17E-08	-2.67
AURKA	aurora kinase A	1.19E-08	1.83
TP53I3	tumor protein p53 inducible protein 3	1.24E-08	-3.01
PNCK	pregnancy up-regulated nonubiquitous CaM kinase	1.29E-08	-2.85
FAM133A	family with sequence similarity 133 member A	1.30E-08	-1.95
ATP11B	ATPase phospholipid transporting 11B (putative)	1.30E-08	2.66
ZNF204P	zinc finger protein 204, pseudogene	1.35E-08	2.44
DLX1	distal-less homeobox 1	1.37E-08	-1.72
GLT8D2	glycosyltransferase 8 domain containing 2	1.39E-08	-1.63
CYP1B1	cytochrome P450 family 1 subfamily B member 1	1.39E-08	-4.9
TIGAR	TP53 induced glycolysis regulatory phosphatase	1.40E-08	-2.26
NMU	neuromedin U	1.45E-08	3.44
FEN1	flap structure-specific endonuclease 1	1.46E-08	2.73
GCG	glucagon	1.47E-08	-3.05
SAMD5	sterile alpha motif domain containing 5	1.48E-08	-2.07
RERGL	RERG like	1.51E-08	-3.82
RUNDC3A	RUN domain containing 3A	1.59E-08	-1.68
MESP1	mesoderm posterior bHLH transcription factor 1	1.62E-08	1.95
SLF1	SMC5-SMC6 complex localization factor 1	1.62E-08	2.84
CKAP2L	cytoskeleton associated protein 2 like	1.63E-08	1.47
ANXA3	annexin A3	1.63E-08	2.9
NUSAP1	nucleolar and spindle associated protein 1	1.66E-08	3.3

CDH10	cadherin 10	1.67E-08	-3.86
ERI1	exoribonuclease 1	1.73E-08	3.2
PDE4B	phosphodiesterase 4B	1.77E-08	1.82
SSBP2	single stranded DNA binding protein 2	1.79E-08	-2.18
MOCOS	molybdenum cofactor sulfurase	1.80E-08	1.81
DBNDD2	dysbindin domain containing 2	1.81E-08	3.96
UBE2C	ubiquitin conjugating enzyme E2 C	1.82E-08	4.01
CRISPLD1	cysteine rich secretory protein LCCL domain containing 1	1.82E-08	2.21
MEOX1	mesenchyme homeobox 1	1.83E-08	1.86
PHLDA3	pleckstrin homology like domain family A member 3	1.84E-08	-2.6
ESCO2	establishment of sister chromatid cohesion N-acetyltransferase 2	1.86E-08	2.33
ERCC6L	ERCC excision repair 6 like, spindle assembly checkpoint helicase	1.86E-08	2.02
LGALS8	galectin 8	1.91E-08	2.13
AP1S2	adaptor related protein complex 1 sigma 2 subunit	1.91E-08	2.77
GATM	glycine amidinotransferase	1.92E-08	2.41
LRRN3	leucine rich repeat neuronal 3	1.94E-08	2.72
TTK	TTK protein kinase	1.94E-08	3.44
DEPDC1B	DEP domain containing 1B	1.97E-08	2.7
MCM5	minichromosome maintenance complex component 5	1.97E-08	2.09
LAMC3	laminin subunit gamma 3	1.97E-08	-2.38
ZNF20	zinc finger protein 20	1.98E-08	-2.41
DYNLT3	dynein light chain Tctex-type 3	1.99E-08	3.48
TMEM47	transmembrane protein 47	2.02E-08	-2.99
MYOC	myocilin	2.04E-08	-2.19
CENPA	centromere protein A	2.07E-08	2.1

EPCAM	epithelial cell adhesion molecule	2.14E-08	3.8
CDC42EP5	CDC42 effector protein 5	2.23E-08	-1.77
ITM2B	integral membrane protein 2B	2.31E-08	-3.52
NMI	N-myc and STAT interactor	2.35E-08	2.35
FXYD5	FXYD domain containing ion transport regulator 5	2.36E-08	3.95
ISOC1	isochorismatase domain containing 1	2.36E-08	2.32
PLTP	phospholipid transfer protein	2.37E-08	-2.99
CDCA8	cell division cycle associated 8	2.39E-08	2.48
CCNB2	cyclin B2	2.40E-08	2.97
CA2	carbonic anhydrase 2	2.41E-08	-2.3
DEPDC1	DEP domain containing 1	2.44E-08	3.16
FBXO5	F-box protein 5	2.57E-08	2.21
FAM64A	family with sequence similarity 64 member A	2.62E-08	2.23
RACGAP1	Rac GTPase activating protein 1	2.65E-08	2.08
TLX3	T-cell leukemia homeobox 3	2.66E-08	-2.52
CXCL1	C-X-C motif chemokine ligand 1	2.70E-08	3.92
DKK1	dickkopf WNT signaling pathway inhibitor 1	2.75E-08	-5.76
SEPP1	selenoprotein P, plasma, 1	2.82E-08	-2.29
GBP1P1	guanylate binding protein 1 pseudogene 1	3.00E-08	2
SPATS2L	spermatogenesis associated serine rich 2 like	3.01E-08	1.87
ANXA1	annexin A1	3.11E-08	3.47
TFPI	tissue factor pathway inhibitor	3.22E-08	-2.69
PLK4	polo like kinase 4	3.23E-08	2.54
MRAP2	melanocortin 2 receptor accessory protein 2	3.29E-08	1.87
PAX6	paired box 6	3.29E-08	1.87
SSPO	SCO-spondin	3.33E-08	-2.42
S100A11	S100 calcium binding protein A11	3.33E-08	-3.69
INA	internexin neuronal intermediate filament protein alpha	3.50E-08	2.52

KIF2C	kinesin family member 2C	3.51E-08	2.37
PTPRU	protein tyrosine phosphatase, receptor type U	3.54E-08	-1.57
TFPI	tissue factor pathway inhibitor	3.54E-08	-3.21
CD248	CD248 molecule	3.58E-08	-3.38
ZWINT	ZW10 interacting kinetochore protein	3.63E-08	2.56
FAM9C	family with sequence similarity 9 member C	3.63E-08	2.38
SNAI2	snail family transcriptional repressor 2	3.66E-08	-3.9
CDK2	cyclin dependent kinase 2	3.66E-08	1.8
MBP	myelin basic protein	3.71E-08	2.3
RBM24	RNA binding motif protein 24	3.71E-08	2.52
AURKB	aurora kinase B	3.75E-08	3.17
ZWINT	ZW10 interacting kinetochore protein	3.86E-08	2.77
STC2	stanniocalcin 2	4.00E-08	-1.65
FAM111A	family with sequence similarity 111 member A	4.03E-08	2.54
WLS	wntless Wnt ligand secretion mediator	4.04E-08	1.73
SKIDA1	SKI/DACH domain containing 1	4.09E-08	2.18
BUB1	BUB1 mitotic checkpoint serine/threonine kinase	4.13E-08	2.77
ZNF816	zinc finger protein 816	4.24E-08	1.35
SERPINB1	serpin family B member 1	4.29E-08	2.73
HSPA5	heat shock protein family A (Hsp70) member 5	4.37E-08	-2.05
GBP1	guanylate binding protein 1	4.42E-08	3.54
TAGLN	transgelin	4.44E-08	2.58
RIPPLY3	ripply transcriptional repressor 3	4.54E-08	2.03
MOCS1	molybdenum cofactor synthesis 1	4.55E-08	1.47
NUP107	nucleoporin 107	4.55E-08	1.78
TCEAL8	transcription elongation factor A like 8	4.55E-08	-3.83
MBNL2	muscleblind like splicing regulator 2	4.66E-08	-2.6

IL1RAP	interleukin 1 receptor accessory protein	4.69E-08	2.8
VIP	vasoactive intestinal peptide	4.71E-08	-2.5
PSMC3IP	PSMC3 interacting protein	4.74E-08	1.43
DDX54	DEAD-box helicase 54	4.81E-08	-1.92
ADAMTS1	ADAM metallopeptidase with thrombospondin type 1 motif 1	4.82E-08	2.89
AP1M2	adaptor related protein complex 1 mu 2 subunit	4.85E-08	1.96
TOP2A	topoisomerase (DNA) II alpha	4.87E-08	3.85
HPSE	heparanase	4.91E-08	1.45
HOXC13	homeobox C13	4.94E-08	-1.44
SHCBP1	SHC binding and spindle associated 1	4.96E-08	2.32
CDCA3	cell division cycle associated 3	5.12E-08	2.98
TSPAN12	tetraspanin 12	5.12E-08	1.4
OIP5	Opa interacting protein 5	5.18E-08	2.77
ADHFE1	alcohol dehydrogenase, iron containing 1	5.18E-08	-1.46
MCM4	minichromosome maintenance complex component 4	5.22E-08	2.32
PSD3	pleckstrin and Sec7 domain containing 3	5.27E-08	2.51
SKA1	spindle and kinetochore associated complex subunit 1	5.31E-08	1.85
PRIM1	primase (DNA) subunit 1	5.51E-08	2.7
DHRS2	dehydrogenase/reductase 2	5.64E-08	-2.41
NFIB	nuclear factor I B	5.64E-08	2.21
BEX1	brain expressed X-linked 1	5.71E-08	-4.48
GRB14	growth factor receptor bound protein 14	5.85E-08	1.89
TACC3	transforming acidic coiled-coil containing protein 3	5.86E-08	2.42
CEP128	centrosomal protein 128	5.98E-08	1.65
LHX2	LIM homeobox 2	6.02E-08	1.27
MND1	meiotic nuclear divisions 1	6.04E-08	2.42

ENO2	enolase 2	6.20E-08	2.01
RTN2	reticulon 2	6.41E-08	-1.9
IL12A	interleukin 12A	6.57E-08	1.32
TFAP2A	transcription factor AP-2 alpha	6.60E-08	-3.44
CRISPLD1	cysteine rich secretory protein LCCL domain containing 1	6.75E-08	1.7
AURKA	aurora kinase A	6.76E-08	1.66
FANCI	Fanconi anemia complementation group I	6.88E-08	2.83
NRG1	neuregulin 1	6.96E-08	2.06
MAD2L1	MAD2 mitotic arrest deficient-like 1 (yeast)	7.02E-08	2.89
SLC38A4	solute carrier family 38 member 4	7.07E-08	1.32
CLYBL	citrate lyase beta like	7.12E-08	-2.49
HAUS8	HAUS augmin like complex subunit 8	7.12E-08	1.43
B3GNT5	UDP-GlcNAc:betaGal beta-1,3-N-acetylglucosaminyltransferase 5	7.32E-08	1.62
SPAG6	sperm associated antigen 6	7.61E-08	2
SPC25	SPC25, NDC80 kinetochore complex component	7.65E-08	2.42
BIRC5	baculoviral IAP repeat containing 5	7.72E-08	3.12
GPC5	glypican 5	7.98E-08	-1.62
MAD2L1	MAD2 mitotic arrest deficient-like 1 (yeast)	8.09E-08	2.71
AXL	AXL receptor tyrosine kinase	8.13E-08	-3.13
OCA2	OCA2 melanosomal transmembrane protein	8.22E-08	-1.88
BMP7	bone morphogenetic protein 7	8.35E-08	-3.21
MCM4	minichromosome maintenance complex component 4	8.36E-08	2.07
PTPRK	protein tyrosine phosphatase, receptor type K	8.38E-08	2.29
TDRD7	tudor domain containing 7	8.44E-08	2.01
BIN1	bridging integrator 1	8.47E-08	1.74
MAP7D2	MAP7 domain containing 2	8.54E-08	1.73
PHPT1	phosphohistidine phosphatase 1	8.56E-08	-1.83

ANK3	ankyrin 3, node of Ranvier (ankyrin G)	8.58E-08	-2.19
KIF20A	kinesin family member 20A	8.58E-08	2.25
ARL6	ADP ribosylation factor like GTPase 6	8.65E-08	1.57
FCGRT	Fc fragment of IgG receptor and transporter	8.68E-08	-1.26
DKFZP564C152	DKFZP564C152 protein	8.69E-08	-1.3
LRRCC1	leucine rich repeat and coiled-coil centrosomal protein 1	8.79E-08	2.56
QPRT	quinolinate phosphoribosyltransferase	8.79E-08	-2.63
RETSAT	retinol saturase	8.80E-08	-1.17
IFRD1	interferon related developmental regulator 1	8.85E-08	1.76
MXRA8	matrix remodeling associated 8	8.85E-08	-1.54
SERPING1	serpin family G member 1	9.03E-08	-2.08
NCAPG2	non-SMC condensin II complex subunit G2	9.04E-08	1.85
PPP1R14A	protein phosphatase 1 regulatory inhibitor subunit 14A	9.32E-08	-3.07
CXXC4	CXXC finger protein 4	9.51E-08	-1.65
GCLM	glutamate-cysteine ligase modifier subunit	9.56E-08	1.37
CMBL	carboxymethylenebutenolidase homolog	9.59E-08	-1.85
PRKCH	protein kinase C eta	9.61E-08	2.17
HS1BP3	HCLS1 binding protein 3	9.68E-08	-2.07
KIFC1	kinesin family member C1	9.81E-08	2.04
EPHX4	epoxide hydrolase 4	9.86E-08	1.74
TK1	thymidine kinase 1	9.89E-08	2.31
MLKL	mixed lineage kinase domain like	9.93E-08	1.96
GALNT14	polypeptide N-acetylgalactosaminyltransferase 14	1.01E-07	1.12
RIMKLB	ribosomal modification protein rimK like family member B	1.04E-07	-2.12
NXT2	nuclear transport factor 2 like export factor 2	1.05E-07	1.78
FBXO2	F-box protein 2	1.05E-07	-1.53
FOXM1	forkhead box M1	1.05E-07	1.81

MBNL2	muscleblind like splicing regulator 2	1.06E-07	-2.29
ZAK	sterile alpha motif and leucine zipper containing kinase AZK	1.06E-07	1.64
BLM	Bloom syndrome RecQ like helicase	1.07E-07	1.34
PDE4B	phosphodiesterase 4B	1.08E-07	1.2
ZMYND8	zinc finger MYND-type containing 8	1.08E-07	-1.57
PAX6	paired box 6	1.08E-07	2.23
TUBA1A	tubulin alpha 1a	1.10E-07	1.43
TOM1L2	target of myb1 like 2 membrane trafficking protein	1.11E-07	2.19
NADSYN1	NAD synthetase 1	1.12E-07	-1.52
DCN	decorin	1.16E-07	-1.85
NEIL3	nei like DNA glycosylase 3	1.18E-07	1.64
DTL	denticleless E3 ubiquitin protein ligase homolog	1.18E-07	3.41
VGLL3	vestigial like family member 3	1.19E-07	2.32
HOXB5	homeobox B5	1.20E-07	1.26
PANK1	pantothenate kinase 1	1.21E-07	-1.66
SESTD1	SEC14 and spectrin domain containing 1	1.21E-07	1.42
ZFP36L1	ZFP36 ring finger protein like 1	1.22E-07	1.18
NKD2	naked cuticle homolog 2	1.22E-07	-1.91
TFAP2A	transcription factor AP-2 alpha	1.23E-07	-2.27
NR2F2	nuclear receptor subfamily 2 group F member 2	1.24E-07	2.92
PDGFC	platelet derived growth factor C	1.24E-07	2.33
TYRO3	TYRO3 protein tyrosine kinase	1.28E-07	1.09
NEBL	nebulette	1.28E-07	1.83
RGS2	regulator of G-protein signaling 2	1.28E-07	-1.85
RNF175	ring finger protein 175	1.30E-07	1.43
CASC10	cancer susceptibility candidate 10	1.30E-07	1.36
MID1IP1	MID1 interacting protein 1	1.31E-07	-1.8

RPL13A	ribosomal protein L13a	1.31E-07	-2.17
MCM10	minichromosome maintenance 10 replication initiation factor	1.33E-07	1.71
PAWR	pro-apoptotic WT1 regulator	1.33E-07	1.96
FAM83D	family with sequence similarity 83 member D	1.35E-07	1.72
NPR2	natriuretic peptide receptor 2	1.35E-07	1.45
RFC4	replication factor C subunit 4	1.37E-07	2.6
ENO3	enolase 3	1.38E-07	-1.81
COL26A1	collagen type XXVI alpha 1 chain	1.39E-07	-1.12
SOX8	SRY-box 8	1.41E-07	-2.29
E2F2	E2F transcription factor 2	1.41E-07	2.85
ZNF79	zinc finger protein 79	1.45E-07	-1.18
ABCA1	ATP binding cassette subfamily A member 1	1.46E-07	-2.5
APOE	apolipoprotein E	1.47E-07	-2.21
FZD6	frizzled class receptor 6	1.47E-07	2.23
SGO1	shugoshin 1	1.48E-07	1.68
DKK3	dickkopf WNT signaling pathway inhibitor 3	1.49E-07	-2.07
TNFSF9	tumor necrosis factor superfamily member 9	1.51E-07	-1.86
POLQ	DNA polymerase theta	1.51E-07	1.84
ADAMTS5	ADAM metallopeptidase with thrombospondin type 1 motif 5	1.53E-07	1.59
GPC6	glypican 6	1.55E-07	-1.51
ANTXR1	anthrax toxin receptor 1	1.56E-07	-3.25
FRY	FRY microtubule binding protein	1.56E-07	1.11
ERRFI1	ERBB receptor feedback inhibitor 1	1.57E-07	1.84
SPACA6	sperm acrosome associated 6	1.57E-07	-1.88
DDX10	DEAD-box helicase 10	1.57E-07	2.26
GRHL3	grainyhead like transcription factor 3	1.58E-07	-2.13
CKMT1A	creatine kinase, mitochondrial 1A	1.59E-07	-1.34
TM4SF18	transmembrane 4 L six family member 18	1.62E-07	1.63

RAD54L	RAD54-like (S. cerevisiae)	1.63E-07	1.74
PCCA	propionyl-CoA carboxylase alpha subunit	1.65E-07	-1.79
OGFRL1	opioid growth factor receptor like 1	1.66E-07	2.78
ASPM	abnormal spindle microtubule assembly	1.67E-07	2.22
MTFR2	mitochondrial fission regulator 2	1.74E-07	2.01
CXXC5	CXXC finger protein 5	1.78E-07	-1.77
PARPBP	PARP1 binding protein	1.82E-07	1.73
HRC	histidine rich calcium binding protein	1.82E-07	-3.6
SATB2	SATB homeobox 2	1.82E-07	2.17
PRC1	protein regulator of cytokinesis 1	1.83E-07	3.17
SCN9A	sodium voltage-gated channel alpha subunit 9	1.84E-07	2.42
ZDHHC13	zinc finger DHHC-type containing 13	1.84E-07	1.51
KIF18A	kinesin family member 18A	1.87E-07	1.45
BIN1	bridging integrator 1	1.88E-07	1.43
PCDHB16	protocadherin beta 16	1.89E-07	-1.74
NFIB	nuclear factor I B	1.89E-07	1.93
C9orf40	chromosome 9 open reading frame 40	1.90E-07	1.27
TCEAL3	transcription elongation factor A like 3	1.91E-07	-3.34
ASB5	ankyrin repeat and SOCS box containing 5	1.92E-07	1.83
PLCD1	phospholipase C delta 1	1.94E-07	-1.42
VRK1	vaccinia related kinase 1	1.94E-07	2.23
SMARCA2	SWI/SNF related, matrix associated, actin dependent regulator of chromatin, subfamily a, member 2	1.95E-07	1.3
PARD6G	par-6 family cell polarity regulator gamma	1.99E-07	-2.31
SUV39H1	suppressor of variegation 3-9 homolog 1	1.99E-07	1.83
TRAPPC3L	trafficking protein particle complex 3 like	2.03E-07	2.92
MASTL	microtubule associated serine/threonine kinase like	2.05E-07	1.82
HS6ST2	heparan sulfate 6-O-sulfotransferase 2	2.11E-07	2.45

CLIP3	CAP-Gly domain containing linker protein 3	2.12E-07	-2.39
SH2D4A	SH2 domain containing 4A	2.12E-07	2.41
PCGF5	polycomb group ring finger 5	2.12E-07	1.44
FBXO5	F-box protein 5	2.13E-07	2.11
PEG10	paternally expressed 10	2.16E-07	1.73
CNPY4	canopy FGF signaling regulator 4	2.21E-07	-1.15
CD36	CD36 molecule	2.23E-07	1.32
TMSB15A	thymosin beta 15a	2.24E-07	2.41
BRCA1	BRCA1, DNA repair associated	2.24E-07	1.94
CT45A3	cancer/testis antigen family 45, member A3	2.25E-07	1.19
SLC39A8	solute carrier family 39 member 8	2.27E-07	2.38
PMAIP1	phorbol-12-myristate-13-acetate-induced protein 1	2.31E-07	1.87
SLC39A8	solute carrier family 39 member 8	2.35E-07	2.37
CDCA2	cell division cycle associated 2	2.36E-07	1.64
GINS2	GINS complex subunit 2	2.43E-07	2.75
IQCK	IQ motif containing K	2.44E-07	1.52
NKX2-5	NK2 homeobox 5	2.45E-07	-2.53
TROAP	trophinin associated protein	2.46E-07	2.1
MLIP	muscular LMNA-interacting protein	2.48E-07	1.41
PPM1D	protein phosphatase, Mg2+/Mn2+ dependent 1D	2.52E-07	-2.17
CDO1	cysteine dioxygenase type 1	2.59E-07	-1.21
VCAN	versican	2.59E-07	-4.08
RIMKLB	ribosomal modification protein rimK like family member B	2.60E-07	-1.24
STIL	SCL/TAL1 interrupting locus	2.64E-07	2.06
CANX	calnexin	2.68E-07	-1.45
UCP2	uncoupling protein 2	2.71E-07	2.11
SOSTDC1	sclerostin domain containing 1	2.73E-07	-1.38

TRPC4	transient receptor potential cation channel subfamily C member 4	2.77E-07	1.39
ANLN	anillin actin binding protein	2.77E-07	2.95
FILIP1	filamin A interacting protein 1	2.77E-07	1.7
NUDT10	nudix hydrolase 10	2.78E-07	-1.28
C1orf52	chromosome 1 open reading frame 52	2.83E-07	-1.41
C14orf169	chromosome 14 open reading frame 169	2.83E-07	-2.57
KCNMB4	potassium calcium-activated channel subfamily M regulatory beta subunit 4	2.84E-07	1.47
EXOSC8	exosome component 8	2.87E-07	1.94
TMEM261	transmembrane protein 261	2.90E-07	-1.11
MIS18A	MIS18 kinetochore protein A	2.92E-07	1.44
SEMA6A	semaphorin 6A	2.98E-07	-1.56
SEMA6B	semaphorin 6B	3.01E-07	-1.56
CD99	CD99 molecule	3.01E-07	-2.37
FRAT2	frequently rearranged in advanced T-cell lymphomas 2	3.02E-07	-1.2
CKMT1B	creatine kinase, mitochondrial 1B	3.02E-07	-1.42
TRIP13	thyroid hormone receptor interactor 13	3.04E-07	1.38
FANCB	Fanconi anemia complementation group B	3.05E-07	9.71E-01
SYTL1	synaptotagmin like 1	3.06E-07	-1.25
C1orf112	chromosome 1 open reading frame 112	3.07E-07	1.69
APOBEC3F	apolipoprotein B mRNA editing enzyme catalytic subunit 3F	3.09E-07	-1.16
KERA	keratocan	3.12E-07	-2.67
LOC729860	uncharacterized LOC729860	3.13E-07	9.99E-01
CA2	carbonic anhydrase 2	3.14E-07	-2
COL23A1	collagen type XXIII alpha 1 chain	3.14E-07	-2.14
SLC27A6	solute carrier family 27 member 6	3.20E-07	1.38

DNAJC9	DnaJ heat shock protein family (Hsp40) member C9	3.21E-07	1.5
ISPD	isoprenoid synthase domain containing	3.24E-07	1.02
MND1	meiotic nuclear divisions 1	3.24E-07	3
JUN	Jun proto-oncogene, AP-1 transcription factor subunit	3.25E-07	1.72
GYG2	glycogenin 2	3.27E-07	-1.99
BMP5	bone morphogenetic protein 5	3.27E-07	-9.52E-01
TMEM237	transmembrane protein 237	3.28E-07	1.26
DUT	deoxyuridine triphosphatase	3.35E-07	1.3
HEY1	hes related family bHLH transcription factor with YRPW motif 1	3.36E-07	1.92
RFC3	replication factor C subunit 3	3.36E-07	2.18
LPCAT3	lysophosphatidylcholine acyltransferase 3	3.39E-07	-1.74
MAT2B	methionine adenosyltransferase 2B	3.46E-07	1.23
ERCC6L	ERCC excision repair 6 like, spindle assembly checkpoint helicase	3.47E-07	1.17
SPIN3	spindlin family member 3	3.47E-07	-1.12
JPH3	junctophilin 3	3.53E-07	-2.05
BBOF1	basal body orientation factor 1	3.56E-07	1.43
BCAS3	BCAS3, microtubule associated cell migration factor	3.59E-07	-1.23
FGF18	fibroblast growth factor 18	3.64E-07	-2.41
MTFR2	mitochondrial fission regulator 2	3.65E-07	1.42
FOXG1	forkhead box G1	3.71E-07	-2.03
PCOLCE2	procollagen C-endopeptidase enhancer 2	3.71E-07	-1.05
KIAA0101	KIAA0101	3.73E-07	1.18
MCUB	mitochondrial calcium uniporter dominant negative beta subunit	3.74E-07	1.41
RPP40	ribonuclease P/MRP subunit p40	3.75E-07	1.61

SLC25A6	solute carrier family 25 member 6	3.76E-07	-1.64
KAZALD1	Kazal type serine peptidase inhibitor domain 1	3.82E-07	1.81
RMI1	RecQ mediated genome instability 1	3.82E-07	1.47
AGR2	anterior gradient 2, protein disulphide isomerase family member	3.82E-07	1.37
PIR	pirin	3.83E-07	1.65
KIF23	kinesin family member 23	3.84E-07	2.23
THNSL2	threonine synthase like 2	3.84E-07	2.61
HMGB1	high mobility group box 1	3.89E-07	2.51
KIAA1147	KIAA1147	3.90E-07	2.41
RPL15	ribosomal protein L15	3.90E-07	-1.29
MAGED2	MAGE family member D2	3.94E-07	-1.68
TCEAL8	transcription elongation factor A like 8	3.96E-07	-3.16
ZNF426	zinc finger protein 426	3.99E-07	1.08
FAXDC2	fatty acid hydroxylase domain containing 2	3.99E-07	-1.31
MCM2	minichromosome maintenance complex component 2	4.05E-07	2.3
DNAJC15	DnaJ heat shock protein family (Hsp40) member C15	4.05E-07	1.58
CEACAM1	carcinoembryonic antigen related cell adhesion molecule 1	4.14E-07	-1.5
ELL2	elongation factor for RNA polymerase II 2	4.15E-07	1.87
TM4SF18	transmembrane 4 L six family member 18	4.17E-07	1.67
HS6ST2	heparan sulfate 6-O-sulfotransferase 2	4.20E-07	1.9
TUBG1	tubulin gamma 1	4.25E-07	1.39
TMPO	thymopoietin	4.27E-07	1.2
LRR1	leucine rich repeat protein 1	4.27E-07	1.7
BUB3	BUB3, mitotic checkpoint protein	4.27E-07	1.42
OXR1	oxidation resistance 1	4.28E-07	-1.85
SIRPA	signal regulatory protein alpha	4.35E-07	-1.88
NEK2	NIMA related kinase 2	4.40E-07	1.7

PAQR7	progestin and adipoQ receptor family member 7	4.43E-07	1.23
FGFR3	fibroblast growth factor receptor 3	4.44E-07	-2.23
STMN1	stathmin 1	4.44E-07	1.73
PLAU	plasminogen activator, urokinase	4.45E-07	-1.23
FMN2	formin 2	4.47E-07	1.29
SPRY2	sprouty RTK signaling antagonist 2	4.49E-07	1.4
HAUS8	HAUS augmin like complex subunit 8	4.49E-07	1.8
PRMT6	protein arginine methyltransferase 6	4.51E-07	1.47
RPP25	ribonuclease P/MRP subunit p25	4.54E-07	-1.2
PDLIM3	PDZ and LIM domain 3	4.59E-07	1.54
GGH	gamma-glutamyl hydrolase	4.61E-07	1.82
HELLS	helicase, lymphoid-specific	4.61E-07	1.74
TRAPPC12	trafficking protein particle complex 12	4.63E-07	-1.13
PRKAB1	protein kinase AMP-activated non-catalytic subunit beta 1	4.63E-07	-1.13
GPC6	glypican 6	4.67E-07	-2.9
ZNF583	zinc finger protein 583	4.70E-07	1.22
TMC6	transmembrane channel like 6	4.71E-07	-1.35
FZD8	frizzled class receptor 8	4.73E-07	-1.69
CHEK1	checkpoint kinase 1	4.75E-07	1.12
UBE2T	ubiquitin conjugating enzyme E2 T	4.75E-07	2.01
RFC5	replication factor C subunit 5	4.82E-07	2.16
COL1A2	collagen type I alpha 2 chain	4.89E-07	-3.87
CACNA1H	calcium voltage-gated channel subunit alpha1 H	4.92E-07	-2.35
PLCL2	phospholipase C like 2	4.92E-07	-2.42
SIX1	SIX homeobox 1	4.93E-07	1.93
CENPV	centromere protein V	4.93E-07	1.95
SCG2	secretogranin II	4.95E-07	1.19

PIK3AP1	phosphoinositide-3-kinase adaptor protein 1	4.97E-07	1.2
HOTAIR	HOX transcript antisense RNA	5.03E-07	-1.66
ECT2	epithelial cell transforming 2	5.04E-07	1.5
ZFP64	ZFP64 zinc finger protein	5.05E-07	-1.11
SH3BP4	SH3 domain binding protein 4	5.05E-07	-2.69
NUF2	NUF2, NDC80 kinetochore complex component	5.13E-07	2.62
MUC15	mucin 15, cell surface associated	5.16E-07	2.46
GAS6	growth arrest specific 6	5.17E-07	-2.08
CDKN3	cyclin dependent kinase inhibitor 3	5.26E-07	2.72
NES	nestin	5.27E-07	-2.02
H19	H19, imprinted maternally expressed transcript (non-protein coding)	5.28E-07	-2.23
SCD5	stearoyl-CoA desaturase 5	5.28E-07	-1.49
STRA6	stimulated by retinoic acid 6	5.44E-07	-1.91
USP1	ubiquitin specific peptidase 1	5.46E-07	2.12
NETO2	neuropilin and tolloid like 2	5.48E-07	1.79
CDO1	cysteine dioxygenase type 1	5.49E-07	-2.61
RNASEH2A	ribonuclease H2 subunit A	5.51E-07	2.26
PLCG2	phospholipase C gamma 2	5.57E-07	1.26
Mar-02	mitochondrial amidoxime reducing component 2	5.58E-07	1.11
GINS3	GINS complex subunit 3	5.60E-07	1.67
CT45A3	cancer/testis antigen family 45, member A3	5.61E-07	1.33
SKAP1	src kinase associated phosphoprotein 1	5.64E-07	1.72
SEH1L	SEH1 like nucleoporin	5.67E-07	1.3
BACE2	beta-site APP-cleaving enzyme 2	5.76E-07	1.48
CORO2A	coronin 2A	5.81E-07	-9.84E-01
ARL6	ADP ribosylation factor like GTPase 6	5.81E-07	1.84

MLH1	mutL homolog 1	5.86E-07	-1.26
CLYBL	citrate lyase beta like	5.92E-07	-2.02
PLCD4	phospholipase C delta 4	5.93E-07	-1.23
MCTP1	multiple C2 and transmembrane domain containing 1	5.93E-07	1.15
SLC2A3	solute carrier family 2 member 3	6.00E-07	-1.96
ELOVL4	ELOVL fatty acid elongase 4	6.00E-07	1.63
DNA2	DNA replication helicase/nuclease 2	6.05E-07	1.44
SLTM	SAFB like transcription modulator	6.07E-07	-1.45
TNNI3	troponin I3, cardiac type	6.08E-07	1.19
CENPU	centromere protein U	6.18E-07	1.55
DACT3	dishevelled binding antagonist of beta catenin 3	6.20E-07	-1.99
LRRC1	leucine rich repeat containing 1	6.22E-07	1.1
C3orf14	chromosome 3 open reading frame 14	6.30E-07	1.92
CLEC11A	C-type lectin domain family 11 member A	6.31E-07	-2.36
PUS7L	pseudouridylate synthase 7 like	6.35E-07	1.19
NUP54	nucleoporin 54	6.37E-07	1.38
PTPRE	protein tyrosine phosphatase, receptor type E	6.41E-07	1.68
DLX1	distal-less homeobox 1	6.42E-07	-2.06
DDX52	DEAD-box helicase 52	6.46E-07	1.16
PPP1R3C	protein phosphatase 1 regulatory subunit 3C	6.48E-07	-1.57
JMJD8	jumonji domain containing 8	6.51E-07	-3
CXXC5	CXXC finger protein 5	6.52E-07	-2.51
VWCE	von Willebrand factor C and EGF domains	6.52E-07	-1.63
PTGES	prostaglandin E synthase	6.57E-07	-1.41
CDH7	cadherin 7	6.59E-07	1.16
KNTC1	kinetochore associated 1	6.66E-07	1.39
DNAH7	dynein axonemal heavy chain 7	6.72E-07	-1.41
MRPS25	mitochondrial ribosomal protein S25	6.75E-07	-1.02

ACSF2	acyl-CoA synthetase family member 2	6.75E-07	-2.07
EEF1A1	eukaryotic translation elongation factor 1 alpha 1	6.78E-07	-1.75
CHFR	checkpoint with forkhead and ring finger domains, E3 ubiquitin protein ligase	6.88E-07	-1.99
CCR1	C-C motif chemokine receptor 1	6.91E-07	1.25
GPER1	G protein-coupled estrogen receptor 1	6.91E-07	-2.05
ATL3	atlastin GTPase 3	6.99E-07	1.19
FEZ1	fasciculation and elongation protein zeta 1	7.04E-07	-1.77
SIX4	SIX homeobox 4	7.09E-07	1.58
TESK1	testis-specific kinase 1	7.09E-07	1.08
ACTA2	actin, alpha 2, smooth muscle, aorta	7.09E-07	1.92
PMAIP1	phorbol-12-myristate-13-acetate-induced protein 1	7.14E-07	1.06
HAUS6	HAUS augmin like complex subunit 6	7.19E-07	1.43
DSCC1	DNA replication and sister chromatid cohesion 1	7.22E-07	2.36
GAS6	growth arrest specific 6	7.23E-07	-2.05
DMKN	dermokine	7.24E-07	1.29
CLIC3	chloride intracellular channel 3	7.30E-07	1.14
TMCO3	transmembrane and coiled-coil domains 3	7.33E-07	-1.13
SPAG1	sperm associated antigen 1	7.35E-07	2.37
CFLAR	CASP8 and FADD like apoptosis regulator	7.36E-07	-1.41
CHRNA3	cholinergic receptor nicotinic alpha 3 subunit	7.43E-07	-1.52
NCAPH2	non-SMC condensin II complex subunit H2	7.57E-07	9.54E-01
TCF4	transcription factor 4	7.59E-07	-1.51
LRRC4C	leucine rich repeat containing 4C	7.61E-07	-2.28
ASS1	argininosuccinate synthase 1	7.62E-07	-1.2
SPDL1	spindle apparatus coiled-coil protein 1	7.63E-07	1.93
ERO1A	endoplasmic reticulum oxidoreductase 1 alpha	7.66E-07	-1.76

CD70	CD70 molecule	7.76E-07	-3.35
TMSB15B	thymosin beta 15B	7.82E-07	1.78
LCN15	lipocalin 15	7.82E-07	-2.59
B3GALNT1	beta-1,3-N-acetylgalactosaminyltransferase 1 (globoside blood group)	7.91E-07	1.15
SPAG6	sperm associated antigen 6	8.02E-07	1.07
TOX	thymocyte selection associated high mobility group box	8.05E-07	1.25
PROCR	protein C receptor	8.11E-07	-1.95
NDRG4	NDRG family member 4	8.14E-07	-1.5
GYG2	glycogenin 2	8.17E-07	-2.49
MGST1	microsomal glutathione S-transferase 1	8.21E-07	1.06
GUCY1A3	guanylate cyclase 1 soluble subunit alpha	8.34E-07	1.26
MYBL1	MYB proto-oncogene like 1	8.34E-07	1.01
GHR	growth hormone receptor	8.35E-07	-1.31
MMP16	matrix metallopeptidase 16	8.55E-07	2.05
LRRC75B	leucine rich repeat containing 75B	8.63E-07	-8.28E-01
DGKA	diacylglycerol kinase alpha	8.63E-07	-1.13
HOTAIR	HOX transcript antisense RNA	8.65E-07	-1.11
SARS	seryl-tRNA synthetase	8.67E-07	-1.35
RAD54B	RAD54 homolog B (S. cerevisiae)	8.69E-07	1.39
SYT4	synaptotagmin 4	8.69E-07	9.99E-01
ACY1	aminoacylase 1	8.70E-07	-1.75
MTBP	MDM2 binding protein	8.75E-07	1.64
ANKRD10	ankyrin repeat domain 10	8.82E-07	-1.61
FZD2	frizzled class receptor 2	8.82E-07	-1.68
CCDC77	coiled-coil domain containing 77	8.84E-07	1.95
RCAN1	regulator of calcineurin 1	9.03E-07	-2.05

ATP11B	ATPase phospholipid transporting 11B (putative)	9.10E-07	2.25
CYB5A	cytochrome b5 type A	9.11E-07	-1.91
SLC8A2	solute carrier family 8 member A2	9.17E-07	-1.77
EXOSC9	exosome component 9	9.27E-07	1.62
DGKA	diacylglycerol kinase alpha	9.34E-07	-1.24
DDB2	damage specific DNA binding protein 2	9.39E-07	-1.29
RBPMS	RNA binding protein with multiple splicing	9.69E-07	-1.54
RAB40B	RAB40B, member RAS oncogene family	9.70E-07	-1.04
TMEM106C	transmembrane protein 106C	9.75E-07	1.08
CLN5	ceroid-lipofuscinosis, neuronal 5	9.85E-07	-1.5
ANGPT1	angiopoietin 1	1.00E-06	-1.13
ESPL1	extra spindle pole bodies like 1, separase	1.00E-06	1.4
INTU	inturned planar cell polarity protein	1.01E-06	8.00E-01
MMP10	matrix metallopeptidase 10	1.01E-06	1.43
NFIA	nuclear factor I A	1.02E-06	2.35
IDO2	indoleamine 2,3-dioxygenase 2	1.03E-06	-1.07
MCM3	minichromosome maintenance complex component 3	1.03E-06	1.87
ZDHHC11	zinc finger DHHC-type containing 11	1.03E-06	-1.2
CLDN16	claudin 16	1.03E-06	2
KLF2	Kruppel like factor 2	1.03E-06	1.7
CHMP4C	charged multivesicular body protein 4C	1.04E-06	1.41
XK	X-linked Kx blood group	1.04E-06	1.1
DDIT4	DNA damage inducible transcript 4	1.05E-06	-1.5
CSTF2	cleavage stimulation factor subunit 2	1.06E-06	1.64
SNORD57	small nucleolar RNA, C/D box 57	1.07E-06	1.01
EXOSC9	exosome component 9	1.08E-06	1.65
INPP1	inositol polyphosphate-1-phosphatase	1.08E-06	-2.54

TCEAL3	transcription elongation factor A like 3	1.08E-06	-2.38
MRI1	methylthioribose-1-phosphate isomerase 1	1.08E-06	-9.35E-01
TTC26	tetratricopeptide repeat domain 26	1.10E-06	1.43
ZNF684	zinc finger protein 684	1.10E-06	1.15
IFIT1	interferon induced protein with tetratricopeptide repeats 1	1.10E-06	-1.33
RPL22	ribosomal protein L22	1.11E-06	-1.16
RRM2B	ribonucleotide reductase regulatory TP53 inducible subunit M2B	1.12E-06	-1.63
EZH2	enhancer of zeste 2 polycomb repressive complex 2 subunit	1.13E-06	1.41
AATF	apoptosis antagonizing transcription factor	1.13E-06	8.13E-01
DHRS2	dehydrogenase/reductase 2	1.14E-06	-1.55
ZWILCH	zwilch kinetochore protein	1.14E-06	1.82
SHTN1	shootin 1	1.14E-06	2.1
PARP2	poly(ADP-ribose) polymerase 2	1.15E-06	1.11
WIF1	WNT inhibitory factor 1	1.15E-06	-1.21
FOXM1	forkhead box M1	1.15E-06	9.98E-01
LAPTM4A	lysosomal protein transmembrane 4 alpha	1.15E-06	-9.05E-01
BRCA1	BRCA1, DNA repair associated	1.16E-06	1.91
FOXR1	forkhead box R1	1.16E-06	1.42
BTG2	BTG anti-proliferation factor 2	1.16E-06	-2.16
SH3GLB1	SH3 domain containing GRB2 like endophilin B1	1.16E-06	1.81
TRIM4	tripartite motif containing 4	1.16E-06	-1.19
SLC2A1	solute carrier family 2 member 1	1.17E-06	-1.4
TMEM56	transmembrane protein 56	1.17E-06	1.59

CERS5	ceramide synthase 5	1.20E-06	-1.68
RFC3	replication factor C subunit 3	1.20E-06	2.34
ARMCX6	armadillo repeat containing, X-linked 6	1.21E-06	1.95
PEG10	paternally expressed 10	1.22E-06	2.66
BCHE	butyrylcholinesterase	1.22E-06	-1.59
LETMD1	LETM1 domain containing 1	1.23E-06	-9.00E-01
RBBP8	RB binding protein 8, endonuclease	1.23E-06	2.53
BTG1	BTG anti-proliferation factor 1	1.24E-06	-1.07
EFNA1	ephrin A1	1.24E-06	-1.14
MYO1B	myosin IB	1.25E-06	1.41
SCPEP1	serine carboxypeptidase 1	1.25E-06	-1.22
TIPIN	TIMELESS interacting protein	1.26E-06	2.03
ZMYND8	zinc finger MYND-type containing 8	1.27E-06	-1.59
HOXB6	homeobox B6	1.27E-06	1.7
ASB7	ankyrin repeat and SOCS box containing 7	1.28E-06	1.19
ARHGAP44	Rho GTPase activating protein 44	1.28E-06	1.59
CES2	carboxylesterase 2	1.29E-06	-1.57
IDO1	indoleamine 2,3-dioxygenase 1	1.29E-06	-1.17
ADGRV1	adhesion G protein-coupled receptor V1	1.30E-06	1.36
NT5C2	5'-nucleotidase, cytosolic II	1.30E-06	1.82
CXorf57	chromosome X open reading frame 57	1.30E-06	1.54
CENPQ	centromere protein Q	1.30E-06	1.95
FAM110D	family with sequence similarity 110 member D	1.31E-06	-1.21
HAUS8	HAUS augmin like complex subunit 8	1.32E-06	1.14
ZNF114	zinc finger protein 114	1.33E-06	-2.27
TMPRSS15	transmembrane protease, serine 15	1.34E-06	9.62E-01
BTG3	BTG anti-proliferation factor 3	1.35E-06	1.83

MIR199A2	microRNA 199a-2	1.36E-06	-2.74
Mar-01	mitochondrial amidoxime reducing component 1	1.36E-06	1.3
GPI	glucose-6-phosphate isomerase	1.36E-06	1.12
PDLIM3	PDZ and LIM domain 3	1.37E-06	9.18E-01
LYAR	Ly1 antibody reactive	1.37E-06	1.52
FANCG	Fanconi anemia complementation group G	1.38E-06	1.87
GUCY1A3	guanylate cyclase 1 soluble subunit alpha	1.39E-06	8.39E-01
CHAF1A	chromatin assembly factor 1 subunit A	1.40E-06	1.35
SESN1	sestrin 1	1.40E-06	-2.74
TRMT5	tRNA methyltransferase 5	1.41E-06	1.22
PERP	PERP, TP53 apoptosis effector	1.41E-06	-2.43
ORC1	origin recognition complex subunit 1	1.42E-06	9.95E-01
PCDH11Y	protocadherin 11 Y-linked	1.43E-06	-9.91E-01
PHLDA1	pleckstrin homology like domain family A member 1	1.43E-06	1.7
IL17RB	interleukin 17 receptor B	1.44E-06	1.23
TRIM51	tripartite motif-containing 51	1.45E-06	-1.99
TMEM50B	transmembrane protein 50B	1.46E-06	-7.97E-01
CORO1B	coronin 1B	1.46E-06	-1.17
HSD17B12	hydroxysteroid 17-beta dehydrogenase 12	1.48E-06	-1.73
APITD1	apoptosis-inducing, TAF9-like domain 1	1.48E-06	1.31
CHDH	choline dehydrogenase	1.53E-06	1.46
KBTBD11	kelch repeat and BTB domain containing 11	1.53E-06	1.25
GUSBP2	glucuronidase, beta pseudogene 2	1.53E-06	-1.07
MYOF	myoferlin	1.54E-06	2.5

SLC22A18	solute carrier family 22 member 18	1.55E-06	-1.41
TMEM80	transmembrane protein 80	1.55E-06	-7.38E-01
CT45A3	cancer/testis antigen family 45, member A3	1.56E-06	1.4
DGKB	diacylglycerol kinase beta	1.58E-06	-1.07
ZNF215	zinc finger protein 215	1.59E-06	1.29
PLD6	phospholipase D family member 6	1.59E-06	-1.18
CFL1	cofilin 1	1.60E-06	9.26E-01
CTSF	cathepsin F	1.61E-06	-1.57
MPZL2	myelin protein zero like 2	1.61E-06	-8.93E-01
PLEKHA5	pleckstrin homology domain containing A5	1.62E-06	-1.31
APH1B	aph-1 homolog B, gamma-secretase subunit	1.64E-06	-1.01
TUBA4A	tubulin alpha 4a	1.65E-06	1.91
ADGRG6	adhesion G protein-coupled receptor G6	1.65E-06	2.22
SLIT2	slit guidance ligand 2	1.65E-06	2.94
SLC30A8	solute carrier family 30 member 8	1.67E-06	-2.38
MASTL	microtubule associated serine/threonine kinase like	1.67E-06	1.53
CNN1	calponin 1	1.67E-06	1.85
SNAP91	synaptosome associated protein 91	1.67E-06	8.20E-01
SSX2IP	SSX family member 2 interacting protein	1.68E-06	1.58
FANCD2	Fanconi anemia complementation group D2	1.68E-06	8.99E-01
GGH	gamma-glutamyl hydrolase	1.70E-06	1.94
CNRIP1	cannabinoid receptor interacting protein 1	1.70E-06	-1.15
LRRC6	leucine rich repeat containing 6	1.70E-06	8.43E-01

PARP3	poly(ADP-ribose) polymerase family member 3	1.74E-06	-1.07
WWOX	WW domain containing oxidoreductase	1.74E-06	8.90E-01
NUP35	nucleoporin 35	1.76E-06	1.28
CTSA	cathepsin A	1.76E-06	1.03
MBNL3	muscleblind like splicing regulator 3	1.76E-06	1.29
CBR3	carbonyl reductase 3	1.77E-06	-2.91
CAPZB	capping actin protein of muscle Z-line beta subunit	1.78E-06	-1.08
METTL8	methyltransferase like 8	1.79E-06	-1.23
PGM3	phosphoglucomutase 3	1.79E-06	1.1
ARMCX3	armadillo repeat containing, X-linked 3	1.80E-06	-1.86
FSTL5	follistatin like 5	1.81E-06	-1.59
C1orf115	chromosome 1 open reading frame 115	1.81E-06	1.29
ZPR1	ZPR1 zinc finger	1.83E-06	9.79E-01
GCNT2	glucosaminyl (N-acetyl) transferase 2, I-branching enzyme (I blood group)	1.84E-06	1.58
FAM198B	family with sequence similarity 198 member B	1.84E-06	-1.56
MIS18A	MIS18 kinetochore protein A	1.85E-06	1.54
CUBN	cubilin	1.88E-06	9.21E-01
KIAA1524	KIAA1524	1.89E-06	1.44
C3orf14	chromosome 3 open reading frame 14	1.89E-06	2.65
TFPI	tissue factor pathway inhibitor	1.90E-06	-2.06
CENPA	centromere protein A	1.90E-06	9.41E-01
BORA	bora, aurora kinase A activator	1.91E-06	1.22
SACS	sacsin molecular chaperone	1.94E-06	1.31
MMS22L	MMS22 like, DNA repair protein	1.94E-06	9.89E-01

SASS6	SAS-6 centriolar assembly protein	1.96E-06	1.64
SPAG5	sperm associated antigen 5	1.97E-06	1.56
EFNB3	ephrin B3	1.97E-06	-2
KIF15	kinesin family member 15	1.99E-06	2
ARRB1	arrestin beta 1	2.00E-06	-1.37
KLHL14	kelch like family member 14	2.03E-06	-4.16
UNG	uracil DNA glycosylase	2.03E-06	1.56
TRAPPC2	trafficking protein particle complex 2	2.03E-06	-8.65E-01
TMEM207	transmembrane protein 207	2.05E-06	7.35E-01
SLC27A3	solute carrier family 27 member 3	2.05E-06	-1.28
ZDHHC13	zinc finger DHHC-type containing 13	2.06E-06	1.37
SULF2	sulfatase 2	2.09E-06	-1.22
MTERF3	mitochondrial transcription termination factor 3	2.09E-06	1.27
HMGN2	high mobility group nucleosomal binding domain 2	2.12E-06	1.25
ICA1	islet cell autoantigen 1	2.13E-06	1.46
TYMSOS	TYMS opposite strand	2.14E-06	-1.66
FHL2	four and a half LIM domains 2	2.15E-06	8.13E-01
POMP	proteasome maturation protein	2.17E-06	9.35E-01
PTGS2	prostaglandin-endoperoxide synthase 2	2.17E-06	-1.17
PLA2G4C	phospholipase A2 group IVC	2.19E-06	-1.27
OSBPL7	oxysterol binding protein like 7	2.20E-06	-1.75
HMGB2	high mobility group box 2	2.21E-06	1.04
LGALS3	lectin, galactoside binding soluble 3	2.21E-06	1.34
CHAF1B	chromatin assembly factor 1 subunit B	2.23E-06	1.35
ARRDC4	arrestin domain containing 4	2.23E-06	1.27

UTP14C	UTP14, small subunit processome component homolog C (S. cerevisiae)	2.25E-06	-1.08
RFC4	replication factor C subunit 4	2.25E-06	2.52
SMYD3	SET and MYND domain containing 3	2.28E-06	-1.2
STON1	stonin 1	2.34E-06	-1.06
GUF1	GUF1 homolog, GTPase	2.34E-06	8.88E-01
KCNQ2	potassium voltage-gated channel subfamily Q member 2	2.36E-06	-2.65
TPX2	TPX2, microtubule nucleation factor	2.39E-06	1.93
SPC24	SPC24, NDC80 kinetochore complex component	2.40E-06	1.22
BRIX1	BRX1, biogenesis of ribosomes	2.40E-06	9.29E-01
BST2	bone marrow stromal cell antigen 2	2.40E-06	-3.41
CCDC178	coiled-coil domain containing 178	2.41E-06	-2
RAB23	RAB23, member RAS oncogene family	2.43E-06	1.03
TNFRSF21	TNF receptor superfamily member 21	2.43E-06	1.12
GEN1	GEN1, Holliday junction 5' flap endonuclease	2.43E-06	9.97E-01
MRE11A	MRE11 homolog A, double strand break repair nuclease	2.44E-06	1.21
OXA1L	OXA1L, mitochondrial inner membrane protein	2.45E-06	-1.18
CTIF	cap binding complex dependent translation initiation factor	2.45E-06	-8.85E-01
RFC3	replication factor C subunit 3	2.46E-06	1.18
ASS1	argininosuccinate synthase 1	2.47E-06	-1.15
CHRNA3	cholinergic receptor nicotinic alpha 3 subunit	2.48E-06	-1.21
GKAP1	G kinase anchoring protein 1	2.51E-06	1.08
STAT2	signal transducer and activator of transcription 2	2.51E-06	-9.95E-01

HES1	hes family bHLH transcription factor 1	2.52E-06	-1.16
SGO2	shugoshin 2	2.52E-06	1.92
MLF1	myeloid leukemia factor 1	2.53E-06	1.98
DNM3	dynamin 3	2.53E-06	-1.14
CYFIP2	cytoplasmic FMR1 interacting protein 2	2.54E-06	-2.93
TFAM	transcription factor A, mitochondrial	2.54E-06	8.50E-01
ACTR6	ARP6 actin-related protein 6 homolog	2.55E-06	1.02
PTPN2	protein tyrosine phosphatase, non-receptor type 2	2.56E-06	1.42
KREMEN2	kringle containing transmembrane protein 2	2.56E-06	-1.31
FSCN1	fascin actin-bundling protein 1	2.58E-06	-2.07
H2AFZ	H2A histone family member Z	2.60E-06	1
DMKN	dermokine	2.61E-06	1.47
SDC1	syndecan 1	2.63E-06	-1.83
AGBL5	ATP/GTP binding protein like 5	2.63E-06	-2.01
SLC12A6	solute carrier family 12 member 6	2.63E-06	1.16
PLCG1	phospholipase C gamma 1	2.63E-06	-1.36
SLC6A15	solute carrier family 6 member 15	2.63E-06	1.28
CENPE	centromere protein E	2.64E-06	1.63
KCNQ2	potassium voltage-gated channel subfamily Q member 2	2.64E-06	-1.36
COX7A2L	cytochrome c oxidase subunit 7A2 like	2.65E-06	-8.80E-01
ACHE	acetylcholinesterase (Cartwright blood group)	2.65E-06	-1.32
ZNF714	zinc finger protein 714	2.66E-06	1.08
TRAIP	TRAF interacting protein	2.66E-06	8.78E-01
TOB1	transducer of ERBB2, 1	2.68E-06	-1.3
MAFA-AS1	MAFA antisense RNA 1	2.68E-06	-1.13

EXO5	exonuclease 5	2.71E-06	8.60E-01
RBM11	RNA binding motif protein 11	2.71E-06	1.39
PTTG3P	pituitary tumor-transforming 3, pseudogene	2.73E-06	2.6
NTPCR	nucleoside-triphosphatase, cancer-related	2.75E-06	-1.73
PSMG1	proteasome assembly chaperone 1	2.76E-06	1.15
NME5	NME/NM23 family member 5	2.77E-06	9.15E-01
THAP11	THAP domain containing 11	2.78E-06	-8.68E-01
ACD	adrenocortical dysplasia homolog	2.78E-06	1.43
NUP210	nucleoporin 210	2.79E-06	1.1
CPA2	carboxypeptidase A2	2.79E-06	-1.33
LRR1	leucine rich repeat protein 1	2.80E-06	1.55
FCHSD2	FCH and double SH3 domains 2	2.85E-06	-1.01
PLRG1	pleiotropic regulator 1	2.85E-06	8.55E-01
ZNF692	zinc finger protein 692	2.85E-06	-1.33
FIGNL1	fidgetin like 1	2.87E-06	1.64
GLRB	glycine receptor beta	2.87E-06	1.4
MRPS30	mitochondrial ribosomal protein S30	2.88E-06	7.74E-01
SRGAP3	SLIT-ROBO Rho GTPase activating protein 3	2.89E-06	-1.2
TYSND1	trypsin domain containing 1	2.89E-06	-1.81
KARS	lysyl-tRNA synthetase	2.90E-06	7.66E-01
CXADR	coxsackie virus and adenovirus receptor	2.90E-06	1.25
PLA2G16	phospholipase A2 group XVI	2.91E-06	1.81
FES	FES proto-oncogene, tyrosine kinase	2.91E-06	-3.04
HCCS	holocytochrome c synthase	2.92E-06	1.3
TFPI2	tissue factor pathway inhibitor 2	2.94E-06	-1.09

IDO2	indoleamine 2,3-dioxygenase 2	2.96E-06	-1.93
IDUA	iduronidase, alpha-L-	2.96E-06	-1.28
DRAM1	DNA damage regulated autophagy modulator 1	2.98E-06	-1.86
CYB5D2	cytochrome b5 domain containing 2	2.99E-06	-9.07E-01
TMEM158	transmembrane protein 158 (gene/pseudogene)	3.00E-06	1.51
PIK3IP1	phosphoinositide-3-kinase interacting protein 1	3.01E-06	-1.24
BRINP3	BMP/retinoic acid inducible neural specific 3	3.02E-06	1.2
CCDC15	coiled-coil domain containing 15	3.02E-06	9.30E-01
EME1	essential meiotic structure-specific endonuclease 1	3.06E-06	7.99E-01
BRINP3	BMP/retinoic acid inducible neural specific 3	3.07E-06	1.42
CDT1	chromatin licensing and DNA replication factor 1	3.07E-06	1.68
POC5	POC5 centriolar protein	3.07E-06	8.57E-01
MARS2	methionyl-tRNA synthetase 2, mitochondrial	3.10E-06	9.04E-01
USB1	U6 snRNA biogenesis phosphodiesterase 1	3.11E-06	8.00E-01
FOLR1	folate receptor 1	3.12E-06	-1.11
KLHL14	kelch like family member 14	3.12E-06	-3.7
LARP1B	La ribonucleoprotein domain family member 1B	3.13E-06	1.18
RBM11	RNA binding motif protein 11	3.15E-06	1.16
SPHAR	S-phase response (cyclin related)	3.17E-06	-9.60E-01

RILPL1	Rab interacting lysosomal protein like 1	3.17E-06	-9.15E-01
MVP	major vault protein	3.17E-06	-8.67E-01
HGC6.3	uncharacterized LOC100128124	3.18E-06	-9.44E-01
ABHD17C	abhydrolase domain containing 17C	3.19E-06	7.45E-01
PAPSS2	3'-phosphoadenosine 5'-phosphosulfate synthase 2	3.20E-06	2.18
PRKCZ	protein kinase C zeta	3.20E-06	-1.16
CIT	citron rho-interacting serine/threonine kinase	3.21E-06	8.64E-01
MYPOP	Myb related transcription factor, partner of profilin	3.23E-06	-7.52E-01
RCAN1	regulator of calcineurin 1	3.24E-06	-2.14
LAMP1	lysosomal associated membrane protein 1	3.25E-06	-1.59
CNNM1	cyclin and CBS domain divalent metal cation transport mediator 1	3.27E-06	-1
TOMM20	translocase of outer mitochondrial membrane 20	3.27E-06	-1.1
BIVM	basic, immunoglobulin-like variable motif containing	3.28E-06	-1.69
SEL1L3	SEL1L family member 3	3.29E-06	1.4
ANO3	anoctamin 3	3.30E-06	1.05
SPEF2	sperm flagellar 2	3.30E-06	8.34E-01
ANXA2	annexin A2	3.33E-06	7.23E-01
MTFR1L	mitochondrial fission regulator 1 like	3.33E-06	-6.97E-01

SLC9A7	solute carrier family 9 member A7	3.35E-06	1.33
CENPL	centromere protein L	3.35E-06	1.3
TRIM6	tripartite motif containing 6	3.35E-06	-1.48
MXRA7	matrix remodeling associated 7	3.35E-06	-9.25E-01
TSEN2	tRNA splicing endonuclease subunit 2	3.37E-06	7.85E-01
JARID2	jumonji and AT-rich interaction domain containing 2	3.38E-06	-1.15
C3orf18	chromosome 3 open reading frame 18	3.39E-06	-8.51E-01
RBM12	RNA binding motif protein 12	3.39E-06	-9.20E-01
NSMCE4A	NSE4 homolog A, SMC5-SMC6 complex component	3.42E-06	1.23
EYA4	EYA transcriptional coactivator and phosphatase 4	3.46E-06	1.62
BTG3	BTG anti-proliferation factor 3	3.46E-06	1.44
C1orf54	chromosome 1 open reading frame 54	3.46E-06	-1.94
RNF138	ring finger protein 138	3.48E-06	1.24
ANKRA2	ankyrin repeat family A member 2	3.50E-06	-1.14
ARMCX3	armadillo repeat containing, X-linked 3	3.53E-06	-1.07
ATP6V1D	ATPase H+ transporting V1 subunit D	3.54E-06	9.99E-01
SH3KBP1	SH3 domain containing kinase binding protein 1	3.57E-06	1.9
RRM2	ribonucleotide reductase regulatory subunit M2	3.59E-06	2.66
CREM	cAMP responsive element modulator	3.62E-06	1.92
KATNA1	katanin catalytic subunit A1	3.62E-06	1.04

MANSC1	MANSC domain containing 1	3.63E-06	9.86E-01
PRR5	proline rich 5	3.64E-06	-9.69E-01
GPR84	G protein-coupled receptor 84	3.64E-06	-8.71E-01
TMPO	thymopoietin	3.67E-06	2.59
APPBP2	amyloid beta precursor protein binding protein 2	3.71E-06	-1.66
TRIAP1	TP53 regulated inhibitor of apoptosis 1	3.71E-06	-1.02
GRIN2C	glutamate ionotropic receptor NMDA type subunit 2C	3.71E-06	-1.41
NFATC2IP	nuclear factor of activated T-cells 2 interacting protein	3.73E-06	6.98E-01
TRIM2	tripartite motif containing 2	3.74E-06	1.72
THOC1	THO complex 1	3.75E-06	1.07
ANXA2P1	annexin A2 pseudogene 1	3.75E-06	1.03
TRNP1	TMF1-regulated nuclear protein 1	3.79E-06	-1.29
MMP16	matrix metallopeptidase 16	3.80E-06	7.99E-01
ZNF214	zinc finger protein 214	3.82E-06	7.50E-01
RHOQ	ras homolog family member Q	3.83E-06	-1.93
SRD5A3	steroid 5 alpha-reductase 3	3.84E-06	7.24E-01
IER5L	immediate early response 5 like	3.87E-06	-1.29
SLC9A9	solute carrier family 9 member A9	3.88E-06	6.72E-01
CENPJ	centromere protein J	3.92E-06	1.6
BRCA2	BRCA2, DNA repair associated	3.92E-06	1.17
MID1	midline 1	3.93E-06	1.8
SLC6A16	solute carrier family 6 member 16	3.93E-06	-1.01

ATP5L	ATP synthase, H+ transporting, mitochondrial Fo complex subunit G	3.95E-06	-1.45
PABPC4L	poly(A) binding protein cytoplasmic 4 like	3.95E-06	-1.28
PARD6A	par-6 family cell polarity regulator alpha	3.96E-06	1.48
SLC39A11	solute carrier family 39 member 11	3.97E-06	-1.33
TBP	TATA-box binding protein	3.99E-06	8.18E-01
OGDHL	oxoglutarate dehydrogenase-like	3.99E-06	-9.25E-01
WBP1	WW domain binding protein 1	4.03E-06	-1.82
AADAT	aminoadipate aminotransferase	4.03E-06	9.39E-01
ANKRD34B	ankyrin repeat domain 34B	4.04E-06	8.75E-01
NUP88	nucleoporin 88	4.04E-06	6.99E-01
SSUH2	ssu-2 homolog (C. elegans)	4.06E-06	9.48E-01
FBXO44	F-box protein 44	4.07E-06	-1.12
COQ8A	coenzyme Q8A	4.10E-06	-2.15
ORC3	origin recognition complex subunit 3	4.12E-06	1.47
ASF1B	anti-silencing function 1B histone chaperone	4.14E-06	1.72
FERMT1	fermitin family member 1	4.14E-06	1.21
KRT8	keratin 8	4.14E-06	8.37E-01
FAM111A	family with sequence similarity 111 member A	4.15E-06	1.91
NFKBIB	NFKB inhibitor beta	4.16E-06	9.52E-01
MORC1	MORC family CW-type zinc finger 1	4.20E-06	-7.84E-01
MEIS2	Meis homeobox 2	4.21E-06	-2.68

MXD4	MAX dimerization protein 4	4.22E-06	-2.33
UCA1	urothelial cancer associated 1 (non-protein coding)	4.23E-06	-2.38
DYNLT3	dynein light chain Tctex-type 3	4.26E-06	1.29
P4HA2	prolyl 4-hydroxylase subunit alpha 2	4.28E-06	-1.23
FAM13A	family with sequence similarity 13 member A	4.29E-06	-7.83E-01
OLFML3	olfactomedin like 3	4.31E-06	-1.21
NKIRAS1	NFKB inhibitor interacting Ras like 1	4.31E-06	1.05
MEMO1	mediator of cell motility 1	4.32E-06	-1.18
UPF3A	UPF3 regulator of nonsense transcripts homolog A (yeast)	4.33E-06	-1.17
BUB1B	BUB1 mitotic checkpoint serine/threonine kinase B	4.35E-06	1.37
HIC2	hypermethylated in cancer 2	4.35E-06	-1.53
FGFR1OP	FGFR1 oncogene partner	4.36E-06	8.46E-01
NUAK2	NUAK family kinase 2	4.37E-06	9.86E-01
CASP2	caspase 2	4.37E-06	9.06E-01
RILPL2	Rab interacting lysosomal protein like 2	4.39E-06	-1.56
TRMT6	tRNA methyltransferase 6	4.39E-06	1.54
BBS7	Bardet-Biedl syndrome 7	4.44E-06	-6.73E-01
SERPINB9	serpin family B member 9	4.45E-06	1.12
KIAA0101	KIAA0101	4.45E-06	2.61
RDH10	retinol dehydrogenase 10 (all-trans)	4.47E-06	2.95
PLD5	phospholipase D family member 5	4.47E-06	-9.85E-01

NEU1	neuraminidase 1 (lysosomal sialidase)	4.47E-06	-8.27E-01
PRPS2	phosphoribosyl pyrophosphate synthetase 2	4.50E-06	1.02
COLEC11	collectin subfamily member 11	4.54E-06	-1.76
NFIL3	nuclear factor, interleukin 3 regulated	4.55E-06	1.16
ZNF280A	zinc finger protein 280A	4.57E-06	-8.46E-01
PALMD	palmdelphin	4.58E-06	-1.2
EPYC	epiphycan	4.60E-06	-1.21
GDF11	growth differentiation factor 11	4.63E-06	-1.08
POLR3G	RNA polymerase III subunit G	4.63E-06	1.28
DCLK1	doublecortin like kinase 1	4.63E-06	1.15
MAP1A	microtubule associated protein 1A	4.66E-06	-1.44
MYOF	myoferlin	4.68E-06	2.74
SEMA3B	semaphorin 3B	4.71E-06	-8.35E-01
MYOF	myoferlin	4.71E-06	1.36
ICA1	islet cell autoantigen 1	4.71E-06	1.64
HOXC4	homeobox C4	4.72E-06	-1.29
NDUFAF7	NADH:ubiquinone oxidoreductase complex assembly factor 7	4.72E-06	-8.98E-01
SUOX	sulfite oxidase	4.72E-06	-8.78E-01
PUM3	pumilio RNA binding family member 3	4.75E-06	1.43
HMGB1	high mobility group box 1	4.77E-06	1.43
POLE3	DNA polymerase epsilon 3, accessory subunit	4.77E-06	1.45
CAMK2B	calcium/calmodulin dependent protein kinase II beta	4.78E-06	-9.32E-01

IL1RAP	interleukin 1 receptor accessory protein	4.79E-06	1.16
EFNB2	ephrin B2	4.84E-06	1.37
CD160	CD160 molecule	4.86E-06	8.69E-01
KCTD8	potassium channel tetramerization domain containing 8	4.87E-06	1.07
CASP4	caspase 4	4.89E-06	9.80E-01
KLHL24	kelch like family member 24	4.92E-06	-1.45
HS3ST3A1	heparan sulfate-glucosamine 3-sulfotransferase 3A1	4.94E-06	7.99E-01
VHL	von Hippel-Lindau tumor suppressor	4.95E-06	-6.81E-01
INIP	INTS3 and NABP interacting protein	4.96E-06	7.77E-01
TUBD1	tubulin delta 1	4.96E-06	-1.11
TMEM132A	transmembrane protein 132A	4.97E-06	-1.53
KBTBD8	kelch repeat and BTB domain containing 8	4.97E-06	8.34E-01
ZWILCH	zwilch kinetochore protein	4.97E-06	1.25
THAP10	THAP domain containing 10	4.98E-06	1.13
MFSD6	major facilitator superfamily domain containing 6	4.99E-06	8.06E-01
MME	membrane metallo-endopeptidase	4.99E-06	-3.66
TUBA3D	tubulin alpha 3d	5.01E-06	2.39
CCNH	cyclin H	5.03E-06	1.17
OPRL1	opioid related nociceptin receptor 1	5.03E-06	-1.06
COL6A1	collagen type VI alpha 1 chain	5.09E-06	-2.62
PI4KAP2	phosphatidylinositol 4-kinase alpha pseudogene 2	5.12E-06	-1.18
SLC25A42	solute carrier family 25 member 42	5.12E-06	-1.54

MAN2C1	mannosidase alpha class 2C member 1	5.13E-06	-7.38E-01
HSD11B1L	hydroxysteroid 11-beta dehydrogenase 1 like	5.15E-06	-1.02
CCDC47	coiled-coil domain containing 47	5.18E-06	-1.08
VPS37A	VPS37A, ESCRT-I subunit	5.19E-06	7.23E-01
PITPNC1	phosphatidylinositol transfer protein, cytoplasmic 1	5.19E-06	-1.47
SULF2	sulfatase 2	5.20E-06	-2.47
SFMBT1	Scm-like with four mbt domains 1	5.22E-06	7.76E-01
SESN2	sestrin 2	5.29E-06	-1.03
MCM6	minichromosome maintenance complex component 6	5.31E-06	1.42
THNSL1	threonine synthase like 1	5.32E-06	9.84E-01
NDC1	NDC1 transmembrane nucleoporin	5.33E-06	1.03
DIRC2	disrupted in renal carcinoma 2	5.35E-06	1.18
CENPK	centromere protein K	5.35E-06	1.73
RAB6B	RAB6B, member RAS oncogene family	5.35E-06	-1
TYSND1	trypsin domain containing 1	5.38E-06	-1.05
KNSTRN	kinetochore localized astrin/SPAG5 binding protein	5.39E-06	1.51
FRS2	fibroblast growth factor receptor substrate 2	5.40E-06	-1.04
NOP58	NOP58 ribonucleoprotein	5.47E-06	8.43E-01
ARID3A	AT-rich interaction domain 3A	5.48E-06	-9.75E-01
ARL4A	ADP ribosylation factor like GTPase 4A	5.49E-06	1.24
KIF11	kinesin family member 11	5.51E-06	2.01

ADARB1	adenosine deaminase, RNA specific B1	5.52E-06	-7.76E-01
SP140L	SP140 nuclear body protein like	5.52E-06	7.90E-01
FEN1	flap structure-specific endonuclease 1	5.52E-06	1.32
POLD3	DNA polymerase delta 3, accessory subunit	5.61E-06	1.09
CDC25A	cell division cycle 25A	5.62E-06	1.6
HLA-DMB	major histocompatibility complex, class II, DM beta	5.64E-06	-8.05E-01
TMEM170A	transmembrane protein 170A	5.67E-06	9.86E-01
PARP2	poly(ADP-ribose) polymerase 2	5.67E-06	1.72
CYCS	cytochrome c, somatic	5.68E-06	-1.08
RPL34	ribosomal protein L34	5.70E-06	-1.7
CHORDC1	cysteine and histidine rich domain containing 1	5.71E-06	1.36
HOXB4	homeobox B4	5.71E-06	1.12
COA5	cytochrome c oxidase assembly factor 5	5.72E-06	-9.25E-01
TRIM68	tripartite motif containing 68	5.72E-06	7.19E-01
CANX	calnexin	5.76E-06	-1.49
NOCT	nocturnin	5.78E-06	1.01
HNRNPAB	heterogeneous nuclear ribonucleoprotein A/B	5.83E-06	8.92E-01
CTSC	cathepsin C	5.90E-06	7.97E-01
PIDD1	p53-induced death domain protein 1	5.90E-06	-1.39
PSD3	pleckstrin and Sec7 domain containing 3	5.91E-06	8.60E-01
C9orf85	chromosome 9 open reading frame 85	5.93E-06	-1.72

FAM46A	family with sequence similarity 46 member A	5.99E-06	1.26
GATS	GATS, stromal antigen 3 opposite strand	5.99E-06	-7.97E-01
MCM7	minichromosome maintenance complex component 7	5.99E-06	2.13
UBAC2	UBA domain containing 2	6.00E-06	-1.55
PDSS1	prenyl (decaprenyl) diphosphate synthase, subunit 1	6.02E-06	1.26
PNISR	PNN interacting serine and arginine rich protein	6.03E-06	-1.07
WDR45	WD repeat domain 45	6.06E-06	-8.35E-01
RBFOX2	RNA binding protein, fox-1 homolog 2	6.14E-06	1.23
TSPYL2	TSPY like 2	6.17E-06	-1.02
COX20	COX20, cytochrome c oxidase assembly factor	6.17E-06	-1.69
FAM72A	family with sequence similarity 72 member A	6.17E-06	1.26
LSM3	LSM3 homolog, U6 small nuclear RNA and mRNA degradation associated	6.20E-06	1.4
FAM43B	family with sequence similarity 43 member B	6.22E-06	-8.76E-01
PLPPR3	phospholipid phosphatase related 3	6.25E-06	-9.98E-01
PMP22	peripheral myelin protein 22	6.27E-06	8.96E-01
LSM5	LSM5 homolog, U6 small nuclear RNA and mRNA degradation associated	6.28E-06	6.98E-01
SYP	synaptophysin	6.28E-06	-9.66E-01
SLFN11	schlafen family member 11	6.30E-06	1.01

SLC29A4	solute carrier family 29 member 4	6.30E-06	-1.44
RNF32	ring finger protein 32	6.30E-06	9.01E-01
TRIM9	tripartite motif containing 9	6.30E-06	7.64E-01
WDR78	WD repeat domain 78	6.31E-06	6.38E-01
BCL2L2	BCL2 like 2	6.32E-06	-1.04
TSPAN13	tetraspanin 13	6.33E-06	9.25E-01
ASTN2	astrotactin 2	6.34E-06	-1.24
RMI2	RecQ mediated genome instability 2	6.43E-06	1.68
PRKX	protein kinase, X-linked	6.47E-06	-1.09
GLA	galactosidase alpha	6.51E-06	1.62
P4HA2	prolyl 4-hydroxylase subunit alpha 2	6.51E-06	-1.26
PIK3CA	phosphatidylinositol-4,5-bisphosphate 3-kinase catalytic subunit alpha	6.53E-06	2.79
UNC13A	unc-13 homolog A	6.54E-06	-6.95E-01
ERAP2	endoplasmic reticulum aminopeptidase 2	6.56E-06	1.43
IFI44	interferon induced protein 44	6.59E-06	7.82E-01
FIGNL1	fidgetin like 1	6.60E-06	1.55
GMPR2	guanosine monophosphate reductase 2	6.64E-06	-7.35E-01
HLA-DRB1	major histocompatibility complex, class II, DR beta 1	6.64E-06	-1.03
LRRC36	leucine rich repeat containing 36	6.64E-06	7.63E-01
VWA5A	von Willebrand factor A domain containing 5A	6.68E-06	1.01

PI4KAP2	phosphatidylinositol 4-kinase alpha pseudogene 2	6.69E-06	-9.05E-01
PROSC	proline synthetase cotranscribed homolog (bacterial)	6.72E-06	9.57E-01
GTF3A	general transcription factor IIIA	6.73E-06	9.25E-01
HOXA10	homeobox A10	6.79E-06	6.71E-01
PTPN3	protein tyrosine phosphatase, non-receptor type 3	6.79E-06	1.1
USP37	ubiquitin specific peptidase 37	6.80E-06	1.16
ISCU	iron-sulfur cluster assembly enzyme	6.82E-06	-1.45
IQCK	IQ motif containing K	6.84E-06	1.02
KDM4D	lysine demethylase 4D	6.88E-06	9.42E-01
C5orf15	chromosome 5 open reading frame 15	6.90E-06	-8.91E-01
PRRG4	proline rich and Gla domain 4	6.91E-06	6.36E-01
SERINC3	serine incorporator 3	6.92E-06	-8.75E-01
ARID5B	AT-rich interaction domain 5B	6.94E-06	-1.93
NRP2	neuropilin 2	6.95E-06	6.19E-01
LOC105379362	uncharacterized LOC105379362	6.96E-06	-2.14
PANK1	pantothenate kinase 1	6.99E-06	-8.88E-01
DMC1	DNA meiotic recombinase 1	7.05E-06	9.44E-01
DHFR	dihydrofolate reductase	7.06E-06	1.13

ADHFE1	alcohol dehydrogenase, iron containing 1	7.07E-06	-8.56E-01
UBQLN1	ubiquilin 1	7.09E-06	1.36
DEPDC1B	DEP domain containing 1B	7.11E-06	1.22
PGS1	phosphatidylglycerophosphate synthase 1	7.12E-06	-7.25E-01
SYAP1	synapse associated protein 1	7.12E-06	7.54E-01
NDST4	N-deacetylase and N-sulfotransferase 4	7.17E-06	-1.51
SLC27A1	solute carrier family 27 member 1	7.31E-06	-9.11E-01
IGIP	IgA inducing protein	7.35E-06	-9.25E-01
PCED1A	PC-esterase domain containing 1A	7.39E-06	-9.73E-01
LRR1	leucine rich repeat protein 1	7.40E-06	1.39
HSD11B1L	hydroxysteroid 11-beta dehydrogenase 1 like	7.43E-06	-1.02
ATP1B2	ATPase Na+/K+ transporting subunit beta 2	7.45E-06	-8.90E-01
LPXN	leupaxin	7.45E-06	-9.50E-01
ANKRD24	ankyrin repeat domain 24	7.51E-06	-1.5
ITGB3BP	integrin subunit beta 3 binding protein	7.54E-06	1.73
FAM129A	family with sequence similarity 129 member A	7.55E-06	8.80E-01
RARRES2	retinoic acid receptor responder 2	7.56E-06	1.05
IQCC	IQ motif containing C	7.60E-06	9.78E-01

PASK	PAS domain containing serine/threonine kinase	7.61E-06	6.79E-01
HNRNPAB	heterogeneous nuclear ribonucleoprotein A/B	7.61E-06	1.05
DMD	dystrophin	7.69E-06	-1.3
EBI3	Epstein-Barr virus induced 3	7.69E-06	-1.46
ABHD10	abhydrolase domain containing 10	7.71E-06	6.66E-01
DDX41	DEAD-box helicase 41	7.72E-06	-8.82E-01
JADE1	jade family PHD finger 1	7.76E-06	1.25
MMP12	matrix metallopeptidase 12	7.78E-06	1.21
PSMC1	proteasome 26S subunit, ATPase 1	7.79E-06	7.69E-01
KXD1	KxDL motif containing 1	7.79E-06	8.54E-01
BCAT1	branched chain amino acid transaminase 1	7.80E-06	-2.6
COQ2	coenzyme Q2, polyprenyltransferase	7.81E-06	8.68E-01
KCNG1	potassium voltage-gated channel modifier subfamily G member 1	7.81E-06	-1.49
IDO1	indoleamine 2,3-dioxygenase 1	7.83E-06	-1.1
ATAD2	ATPase family, AAA domain containing 2	7.86E-06	2.6
GPR19	G protein-coupled receptor 19	7.87E-06	7.63E-01
AHNAK2	AHNAK nucleoprotein 2	7.90E-06	-1.11
A2M	alpha-2-macroglobulin	7.92E-06	-1.81
TADA2A	transcriptional adaptor 2A	7.93E-06	7.27E-01
BIVM	basic, immunoglobulin-like variable motif containing	7.98E-06	-1.63
C21orf33	chromosome 21 open reading frame 33	8.02E-06	-1.1

ZSCAN4	zinc finger and SCAN domain containing 4	8.03E-06	-8.58E-01
S100A10	S100 calcium binding protein A10	8.07E-06	-1.84
PRCP	prolylcarboxypeptidase	8.08E-06	-1.24
APOBEC3C	apolipoprotein B mRNA editing enzyme catalytic subunit 3C	8.09E-06	-1.07
DIDO1	death inducer-obliterator 1	8.14E-06	7.52E-01
KIF22	kinesin family member 22	8.14E-06	1.51
ESRRG	estrogen related receptor gamma	8.17E-06	-1.32
ZC3H12C	zinc finger CCCH-type containing 12C	8.18E-06	8.99E-01
EGLN1	egl-9 family hypoxia inducible factor 1	8.21E-06	-1.09
MSI2	musashi RNA binding protein 2	8.23E-06	9.54E-01
COL3A1	collagen type III alpha 1 chain	8.25E-06	-3.21
MBD3L5	methyl-CpG binding domain protein 3 like 5	8.29E-06	-9.31E-01
ZNF772	zinc finger protein 772	8.32E-06	7.30E-01
ACAD11	acyl-CoA dehydrogenase family member 11	8.44E-06	-1.31
PSRC1	proline and serine rich coiled-coil 1	8.50E-06	1.2
MUS81	MUS81 structure-specific endonuclease subunit	8.51E-06	1.23
ACTL6A	actin like 6A	8.54E-06	1.3
NOP56	NOP56 ribonucleoprotein	8.59E-06	1.23
ATP8B2	ATPase phospholipid transporting 8B2	8.62E-06	-1.47
BCAT2	branched chain amino acid transaminase 2	8.62E-06	-7.43E-01
C2orf69	chromosome 2 open reading frame 69	8.65E-06	1.4
DPYSL4	dihydropyrimidinase like 4	8.66E-06	-1.92

MFSD6	major facilitator superfamily domain containing 6	8.66E-06	8.67E-01
ATF3	activating transcription factor 3	8.66E-06	7.62E-01
MRI1	methylthioribose-1-phosphate isomerase 1	8.68E-06	-8.78E-01
CELF1	CUGBP, Elav-like family member 1	8.69E-06	8.61E-01
PNMA2	paraneoplastic Ma antigen 2	8.69E-06	-1.09
AMMECR1	Alport syndrome, mental retardation, midface hypoplasia and elliptocytosis chromosomal region gene 1	8.69E-06	6.46E-01
OXCT1	3-oxoacid CoA-transferase 1	8.70E-06	1.26
XIST	X inactive specific transcript (non-protein coding)	8.71E-06	-2.24
CNTFR	ciliary neurotrophic factor receptor	8.75E-06	-1.05
CEP152	centrosomal protein 152	8.77E-06	1.35
SAT1	spermidine/spermine N1-acetyltransferase 1	8.80E-06	-6.16E-01
KLHL2	kelch like family member 2	8.85E-06	1.08
SLC10A4	solute carrier family 10 member 4	8.86E-06	1.3
KRT18	keratin 18	8.94E-06	6.35E-01
RPS27L	ribosomal protein S27 like	8.96E-06	-1.03
ATM	ATM serine/threonine kinase	8.97E-06	-1.23
BARD1	BRCA1 associated RING domain 1	9.03E-06	1.91
RGL1	ral guanine nucleotide dissociation stimulator like 1	9.10E-06	-9.76E-01
JMJD8	jumonji domain containing 8	9.12E-06	-2.82
SYNCRIP	synaptotagmin binding cytoplasmic RNA interacting protein	9.14E-06	8.91E-01

LYAR	Ly1 antibody reactive	9.16E-06	1.34
CLGN	calmegin	9.17E-06	9.78E-01
RAB21	RAB21, member RAS oncogene family	9.18E-06	1.35
CENPI	centromere protein I	9.25E-06	1.19
PRKACB	protein kinase cAMP-activated catalytic subunit beta	9.26E-06	6.36E-01
SNX1	sorting nexin 1	9.30E-06	-7.32E-01
ANKRA2	ankyrin repeat family A member 2	9.33E-06	-1.55
PRPSAP1	phosphoribosyl pyrophosphate synthetase associated protein 1	9.34E-06	-7.46E-01
EIF2D	eukaryotic translation initiation factor 2D	9.34E-06	-8.52E-01
EDA2R	ectodysplasin A2 receptor	9.35E-06	-8.10E-01
PON2	paraoxonase 2	9.42E-06	1.18
ST13P4	suppression of tumorigenicity 13 (colon carcinoma) (Hsp70 interacting protein) pseudogene 4	9.45E-06	-9.65E-01
CLGN	calmegin	9.46E-06	9.71E-01
MTERF3	mitochondrial transcription termination factor 3	9.47E-06	1.39
CFD	complement factor D	9.48E-06	-2.34
POLE	DNA polymerase epsilon, catalytic subunit	9.48E-06	1.04
LIPA	lipase A, lysosomal acid type	9.51E-06	-1.66
SNX10	sorting nexin 10	9.53E-06	1.34
PRKAG2	protein kinase AMP-activated non-catalytic subunit gamma 2	9.53E-06	8.55E-01

FOXA1	forkhead box A1	9.54E-06	8.28E -01
ASRGL1	asparaginase like 1	9.59E-06	9.78E -01
MMP11	matrix metallopeptidase 11	9.61E-06	- 8.25E -01
SLC2A10	solute carrier family 2 member 10	9.63E-06	8.97E -01
HLA-DRB6	major histocompatibility complex, class II, DR beta 6 (pseudogene)	9.64E-06	- 8.17E -01
LSM6	LSM6 homolog, U6 small nuclear RNA and mRNA degradation associated	9.67E-06	1.27
BCHE	butyrylcholinesterase	9.68E-06	-1.09
C4orf46	chromosome 4 open reading frame 46	9.92E-06	1.25
SLC7A10	solute carrier family 7 member 10	1.00E-05	-1.47
EYA4	EYA transcriptional coactivator and phosphatase 4	1.00E-05	9.85E -01
TANC2	tetratricopeptide repeat, ankyrin repeat and coiled-coil containing 2	1.00E-05	-1.19
C18orf8	chromosome 18 open reading frame 8	1.00E-05	7.90E -01
CDC25C	cell division cycle 25C	1.01E-05	1.22
POC1A	POC1 centriolar protein A	1.01E-05	1.75
CENPE	centromere protein E	1.01E-05	1.71
RIF1	replication timing regulatory factor 1	1.01E-05	1.08
CCDC110	coiled-coil domain containing 110	1.01E-05	7.05E -01
ATIC	5-aminoimidazole-4-carboxamide ribonucleotide formyltransferase/IMP cyclohydrolase	1.02E-05	6.82E -01
MACROD1	MACRO domain containing 1	1.02E-05	-1.92
FAIM	Fas apoptotic inhibitory molecule	1.02E-05	1.11

PCID2	PCI domain containing 2	1.02E-05	-1.01
PLPP1	phospholipid phosphatase 1	1.02E-05	-7.29E-01
SEC61A2	Sec61 translocon alpha 2 subunit	1.03E-05	6.23E-01
OSTN	osteocrin	1.03E-05	-8.75E-01
SERPINH1	serpin family H member 1	1.03E-05	-1.31
CDKN1B	cyclin dependent kinase inhibitor 1B	1.03E-05	-7.30E-01
KLC3	kinesin light chain 3	1.04E-05	7.05E-01
COL1A2	collagen type I alpha 2 chain	1.04E-05	-2.76
NDN	necdin, MAGE family member	1.04E-05	-9.72E-01
ZSWIM5	zinc finger SWIM-type containing 5	1.05E-05	-7.30E-01
IL1RAPL1	interleukin 1 receptor accessory protein like 1	1.05E-05	7.60E-01
ANKRD50	ankyrin repeat domain 50	1.05E-05	-9.07E-01
KCNT2	potassium sodium-activated channel subfamily T member 2	1.05E-05	7.59E-01
CYBA	cytochrome b-245 alpha chain	1.06E-05	6.33E-01
FBXO22	F-box protein 22	1.06E-05	-1.14
TC2N	tandem C2 domains, nuclear	1.07E-05	6.55E-01
ADPRHL1	ADP-ribosylhydrolase like 1	1.07E-05	-7.57E-01

DPYD	dihydropyrimidine dehydrogenase	1.07E-05	8.66E-01
SCNN1A	sodium channel epithelial 1 alpha subunit	1.07E-05	-1.74
VEGFC	vascular endothelial growth factor C	1.07E-05	-9.62E-01
RBM20	RNA binding motif protein 20	1.07E-05	-1.91
CITED4	Cbp/p300 interacting transactivator with Glu/Asp rich carboxy-terminal domain 4	1.07E-05	7.30E-01
FBLN2	fibulin 2	1.08E-05	-1.03
BVES	blood vessel epicardial substance	1.08E-05	-2.2
SGMS2	sphingomyelin synthase 2	1.08E-05	7.49E-01
PRRX1	paired related homeobox 1	1.08E-05	8.14E-01
C1RL	complement C1r subcomponent like	1.08E-05	-1.02
RRM1	ribonucleotide reductase catalytic subunit M1	1.08E-05	1.63
NRGN	neurogranin	1.08E-05	1.41
PNPLA7	patatin like phospholipase domain containing 7	1.09E-05	-1.38
DCLRE1A	DNA cross-link repair 1A	1.09E-05	1.32
NRTN	neurturin	1.09E-05	-6.50E-01
PPP2R1B	protein phosphatase 2 scaffold subunit Abeta	1.10E-05	9.86E-01
ORC6	origin recognition complex subunit 6	1.10E-05	1.25
ANKLE1	ankyrin repeat and LEM domain containing 1	1.10E-05	-1.29
ALDH7A1	aldehyde dehydrogenase 7 family member A1	1.10E-05	-1.3
DCK	deoxycytidine kinase	1.10E-05	1.52
MAN2B1	mannosidase alpha class 2B member 1	1.10E-05	-9.54E-01
TP53INP1	tumor protein p53 inducible nuclear protein 1	1.11E-05	-2.33

FAM84B	family with sequence similarity 84 member B	1.11E-05	-1.41
FAM184A	family with sequence similarity 184 member A	1.11E-05	1.04
MIR214	microRNA 214	1.11E-05	-1.67
SLTM	SAFB like transcription modulator	1.11E-05	-1.53
UEVLD	UEV and lactate/malate dehyrogenase domains	1.12E-05	6.60E -01
RANBP1	RAN binding protein 1	1.12E-05	1.28
MCM3	minichromosome maintenance complex component 3	1.12E-05	2.01
ODC1	ornithine decarboxylase 1	1.13E-05	7.94E -01
NME7	NME/NM23 family member 7	1.14E-05	7.77E -01
ABRACL	ABRA C-terminal like	1.14E-05	9.54E -01
POLA2	DNA polymerase alpha 2, accessory subunit	1.14E-05	1.49
PTPN2	protein tyrosine phosphatase, non-receptor type 2	1.14E-05	9.96E -01
PTK7	protein tyrosine kinase 7 (inactive)	1.14E-05	-1.03
KIAA0355	KIAA0355	1.14E-05	-1.06
MAP7D1	MAP7 domain containing 1	1.15E-05	-1.19
PTTG1	pituitary tumor-transforming 1	1.15E-05	2.26
ARIH2	ariadne RBR E3 ubiquitin protein ligase 2	1.16E-05	-1.38
MBD2	methyl-CpG binding domain protein 2	1.16E-05	1.08
TMSB15B	thymosin beta 15B	1.16E-05	1.57
PYCARD	PYD and CARD domain containing	1.16E-05	9.22E -01
HMGB2	high mobility group box 2	1.17E-05	1.06
KNL1	kinetochore scaffold 1	1.17E-05	9.00E -01
MINA	MYC induced nuclear antigen	1.17E-05	7.05E -01

CYTH2	cytohesin 2	1.18E-05	-1.25
CMSS1	cms1 ribosomal small subunit homolog (yeast)	1.19E-05	1.67
RYBP	RING1 and YY1 binding protein	1.19E-05	-7.13E-01
MRS2	MRS2, magnesium transporter	1.20E-05	1
NEURL2	neuralized E3 ubiquitin protein ligase 2	1.21E-05	-1.83
NUP35	nucleoporin 35	1.21E-05	1.15
ACADVL	acyl-CoA dehydrogenase, very long chain	1.21E-05	-8.74E-01
ASAP3	ArfGAP with SH3 domain, ankyrin repeat and PH domain 3	1.21E-05	-1.24
HCCS	holocytochrome c synthase	1.21E-05	1.42
PIGA	phosphatidylinositol glycan anchor biosynthesis class A	1.21E-05	9.13E-01
SLC6A15	solute carrier family 6 member 15	1.22E-05	7.20E-01
B2M	beta-2-microglobulin	1.22E-05	6.63E-01
ZNF620	zinc finger protein 620	1.22E-05	5.97E-01
COL7A1	collagen type VII alpha 1 chain	1.22E-05	-8.09E-01
CSNK2A2	casein kinase 2 alpha 2	1.22E-05	-7.61E-01
LINGO1	leucine rich repeat and Ig domain containing 1	1.23E-05	-6.36E-01
DENND1A	DENN domain containing 1A	1.23E-05	8.75E-01
PRKAG2	protein kinase AMP-activated non-catalytic subunit gamma 2	1.23E-05	8.77E-01

PSMB8	proteasome subunit beta 8	1.24E-05	5.44E-01
SPOPL	speckle type BTB/POZ protein like	1.24E-05	8.19E-01
KMT5B	lysine methyltransferase 5B	1.25E-05	1.25
TRIM7	tripartite motif containing 7	1.25E-05	8.66E-01
TAF5	TATA-box binding protein associated factor 5	1.25E-05	1.38
EPHA5	EPH receptor A5	1.26E-05	7.78E-01
CEP57L1	centrosomal protein 57 like 1	1.26E-05	9.57E-01
ZNF385A	zinc finger protein 385A	1.26E-05	-8.09E-01
NOL3	nucleolar protein 3	1.27E-05	-8.24E-01
CBLN2	cerebellin 2 precursor	1.27E-05	-9.45E-01
ALPL	alkaline phosphatase, liver/bone/kidney	1.28E-05	-2.09
HNRNPAB	heterogeneous nuclear ribonucleoprotein A/B	1.28E-05	1.18
SMARCD3	SWI/SNF related, matrix associated, actin dependent regulator of chromatin, subfamily d, member 3	1.28E-05	-7.02E-01
MB21D2	Mab-21 domain containing 2	1.29E-05	8.84E-01
SMC2	structural maintenance of chromosomes 2	1.30E-05	1.64
GTPBP4	GTP binding protein 4	1.30E-05	7.10E-01
PARD6G	par-6 family cell polarity regulator gamma	1.31E-05	-9.85E-01

WIPI2	WD repeat domain, phosphoinositide interacting 2	1.31E-05	-7.24E-01
PSMB9	proteasome subunit beta 9	1.31E-05	1.06
OTULIN	OTU deubiquitinase with linear linkage specificity	1.32E-05	5.60E-01
ARPC5	actin related protein 2/3 complex subunit 5	1.34E-05	7.00E-01
LMO3	LIM domain only 3	1.34E-05	-9.77E-01
FAM53B	family with sequence similarity 53 member B	1.35E-05	-6.38E-01
ARHGAP44	Rho GTPase activating protein 44	1.35E-05	1.43
CYFIP2	cytoplasmic FMR1 interacting protein 2	1.35E-05	-1.43
MMP12	matrix metallopeptidase 12	1.35E-05	9.78E-01
WDHD1	WD repeat and HMG-box DNA binding protein 1	1.36E-05	1.33
VPS28	VPS28, ESCRT-I subunit	1.37E-05	-1.09
SNX7	sorting nexin 7	1.38E-05	1.06
FOS	Fos proto-oncogene, AP-1 transcription factor subunit	1.38E-05	-9.13E-01
SOWAHA	sosondowah ankyrin repeat domain family member A	1.39E-05	7.45E-01
RAB31	RAB31, member RAS oncogene family	1.40E-05	-7.44E-01
KCNQ2	potassium voltage-gated channel subfamily Q member 2	1.41E-05	-1.06
NDUFAF7	NADH:ubiquinone oxidoreductase complex assembly factor 7	1.42E-05	-1.32
TXLNG	taxilin gamma	1.42E-05	7.42E-01

XRCC3	X-ray repair cross complementing 3	1.43E-05	1.13
RPL39L	ribosomal protein L39 like	1.43E-05	1.41
DUSP23	dual specificity phosphatase 23	1.44E-05	1.3
LRRC8C	leucine rich repeat containing 8 family member C	1.44E-05	8.84E-01
GSAP	gamma-secretase activating protein	1.44E-05	7.33E-01
TMEM45A	transmembrane protein 45A	1.44E-05	-9.69E-01
CBR1	carbonyl reductase 1	1.45E-05	8.06E-01
CTSV	cathepsin V	1.45E-05	1.14
PI15	peptidase inhibitor 15	1.45E-05	-9.98E-01
TMEM101	transmembrane protein 101	1.45E-05	-1.28
BBOF1	basal body orientation factor 1	1.45E-05	1.09
COMMD3	COMM domain containing 3	1.45E-05	9.35E-01
CDK5RAP3	CDK5 regulatory subunit associated protein 3	1.45E-05	-8.20E-01
GTDC1	glycosyltransferase like domain containing 1	1.47E-05	6.84E-01
ABHD6	abhydrolase domain containing 6	1.47E-05	-5.69E-01
RNF213	ring finger protein 213	1.47E-05	7.50E-01
SKP2	S-phase kinase-associated protein 2, E3 ubiquitin protein ligase	1.47E-05	1.23
NUDT7	nudix hydrolase 7	1.48E-05	1.23

FOXL2	forkhead box L2	1.49E-05	-7.81E-01
AGRN	agrin	1.49E-05	-1.93
ZNF446	zinc finger protein 446	1.49E-05	-5.71E-01
FLNC	filamin C	1.50E-05	-1.75
PDSS1	prenyl (decaprenyl) diphosphate synthase, subunit 1	1.50E-05	1.07
CPSF4	cleavage and polyadenylation specific factor 4	1.51E-05	-9.43E-01
CHORDC1	cysteine and histidine rich domain containing 1	1.51E-05	1.55
NGFR	nerve growth factor receptor	1.52E-05	-1.38
CALR	calreticulin	1.52E-05	-1.16
RHOC	ras homolog family member C	1.53E-05	-6.65E-01
ZMYND11	zinc finger MYND-type containing 11	1.53E-05	6.57E-01
GNG11	G protein subunit gamma 11	1.53E-05	-1.64
GSG1	germ cell associated 1	1.53E-05	1.04
GSTM1	glutathione S-transferase mu 1	1.54E-05	-1.44
SDAD1	SDA1 domain containing 1	1.55E-05	9.11E-01
MKNK2	MAP kinase interacting serine/threonine kinase 2	1.55E-05	-1.43
SESN3	sestrin 3	1.56E-05	8.76E-01
ASB3	ankyrin repeat and SOCS box containing 3	1.57E-05	-7.84E-01
ARL4A	ADP ribosylation factor like GTPase 4A	1.57E-05	1.18

CDKN2A	cyclin dependent kinase inhibitor 2A	1.57E-05	1.43
KLHL28	kelch like family member 28	1.57E-05	-9.95E-01
OAS3	2'-5'-oligoadenylate synthetase 3	1.58E-05	8.41E-01
GRN	granulin	1.59E-05	-1.15
PCDH11X	protocadherin 11 X-linked	1.60E-05	-5.96E-01
CCDC74B	coiled-coil domain containing 74B	1.61E-05	8.84E-01
WLS	wntless Wnt ligand secretion mediator	1.61E-05	6.20E-01
PLAG1	PLAG1 zinc finger	1.62E-05	-6.72E-01
MMRN1	multimerin 1	1.62E-05	-1.11
CAMK1D	calcium/calmodulin dependent protein kinase ID	1.62E-05	-7.26E-01
HN1L	hematological and neurological expressed 1 like	1.62E-05	-6.68E-01
MAP2	microtubule associated protein 2	1.62E-05	6.14E-01
BACE2	beta-site APP-cleaving enzyme 2	1.64E-05	1.9
FAM127C	family with sequence similarity 127 member C	1.64E-05	-7.30E-01
LOC286254	uncharacterized LOC286254	1.65E-05	-2.05
CLUAP1	clusterin associated protein 1	1.66E-05	7.09E-01
LZTFL1	leucine zipper transcription factor like 1	1.66E-05	1.04
BAZ1A	bromodomain adjacent to zinc finger domain 1A	1.67E-05	2.17

TTI2	TELO2 interacting protein 2	1.67E-05	7.49E-01
JTB	jumping translocation breakpoint	1.67E-05	5.79E-01
PIP4K2A	phosphatidylinositol-5-phosphate 4-kinase type 2 alpha	1.68E-05	9.02E-01
LAMB2	laminin subunit beta 2	1.68E-05	-1.66
PSMA6	proteasome subunit alpha 6	1.69E-05	7.08E-01
TAGLN	transgelin	1.69E-05	2.39
RNF207	ring finger protein 207	1.70E-05	-8.23E-01
GOLGA8A	golgin A8 family member A	1.70E-05	-1.34
ALDH7A1	aldehyde dehydrogenase 7 family member A1	1.73E-05	-1.44
INF2	inverted formin, FH2 and WH2 domain containing	1.73E-05	7.90E-01
ZNF543	zinc finger protein 543	1.75E-05	5.79E-01
PLSCR1	phospholipid scramblase 1	1.76E-05	6.74E-01
PRAMEF4	PRAME family member 4	1.76E-05	-8.76E-01
EAF2	ELL associated factor 2	1.76E-05	1.32
SKP2	S-phase kinase-associated protein 2, E3 ubiquitin protein ligase	1.77E-05	1.38
MAP3K12	mitogen-activated protein kinase kinase kinase 12	1.77E-05	-1.04
RPA2	replication protein A2	1.77E-05	1.4
ZNF462	zinc finger protein 462	1.77E-05	9.37E-01
NBEA	neurobeachin	1.78E-05	9.88E-01

ARHGAP18	Rho GTPase activating protein 18	1.78E-05	8.03E-01
PPP2CA	protein phosphatase 2 catalytic subunit alpha	1.78E-05	7.88E-01
C11orf70	chromosome 11 open reading frame 70	1.79E-05	1.53
LRP1	LDL receptor related protein 1	1.79E-05	-9.61E-01
RNF170	ring finger protein 170	1.79E-05	-1.08
SKIL	SKI-like proto-oncogene	1.79E-05	1.1
PKMYT1	protein kinase, membrane associated tyrosine/threonine 1	1.80E-05	1.19
NECTIN3	nectin cell adhesion molecule 3	1.80E-05	-8.04E-01
ZNF581	zinc finger protein 581	1.80E-05	-1.68
PSMD1	proteasome 26S subunit, non-ATPase 1	1.81E-05	8.91E-01
CNTNAP2	contactin associated protein-like 2	1.81E-05	5.67E-01
MIPEP	mitochondrial intermediate peptidase	1.81E-05	7.30E-01
NT5C3A	5'-nucleotidase, cytosolic IIIA	1.82E-05	7.13E-01
ASNSD1	asparagine synthetase domain containing 1	1.82E-05	1.22
HELZ2	helicase with zinc finger 2	1.82E-05	1.35
SLBP	stem-loop binding protein	1.83E-05	5.95E-01
EIF1AD	eukaryotic translation initiation factor 1A domain containing	1.83E-05	8.79E-01
C11orf70	chromosome 11 open reading frame 70	1.84E-05	1.37
TP53I3	tumor protein p53 inducible protein 3	1.85E-05	-2.1
GRWD1	glutamate rich WD repeat containing 1	1.85E-05	8.47E-01

HNRNPD	heterogeneous nuclear ribonucleoprotein D	1.85E-05	6.03E-01
LINC01139	long intergenic non-protein coding RNA 1139	1.86E-05	-8.97E-01
ATRAID	all-trans retinoic acid induced differentiation factor	1.86E-05	-1.03
GSG2	germ cell associated 2, haspin	1.86E-05	7.03E-01
NOVA1	NOVA alternative splicing regulator 1	1.87E-05	1.02
FAR2	fatty acyl-CoA reductase 2	1.87E-05	5.57E-01
SNRPA1	small nuclear ribonucleoprotein polypeptide A'	1.88E-05	8.60E-01
IGDCC4	immunoglobulin superfamily DCC subclass member 4	1.88E-05	-1.81
KNSTRN	kinetochore localized astrin/SPAG5 binding protein	1.89E-05	1.15
RAB3GAP2	RAB3 GTPase activating non-catalytic protein subunit 2	1.89E-05	-7.55E-01
CLIP4	CAP-Gly domain containing linker protein family member 4	1.89E-05	8.46E-01
EMB	embigin	1.90E-05	1
RBBP7	RB binding protein 7, chromatin remodeling factor	1.90E-05	1.44
HPF1	histone PARylation factor 1	1.91E-05	1.07
SMPDL3A	sphingomyelin phosphodiesterase acid like 3A	1.91E-05	-6.82E-01
SFRP1	secreted frizzled related protein 1	1.91E-05	-2.47
COPS8	COP9 signalosome subunit 8	1.91E-05	8.07E-01
M6PR	mannose-6-phosphate receptor, cation dependent	1.91E-05	-7.66E-01

MARVELD3	MARVEL domain containing 3	1.92E-05	6.30E -01
C1orf174	chromosome 1 open reading frame 174	1.93E-05	7.80E -01
TET1	tet methylcytosine dioxygenase 1	1.94E-05	-1.06
PRSS16	protease, serine 16	1.94E-05	8.09E -01
ORC5	origin recognition complex subunit 5	1.94E-05	8.32E -01
PRCP	prolylcarboxypeptidase	1.94E-05	- 9.59E -01
FGF2	fibroblast growth factor 2	1.94E-05	1.17
MAK16	MAK16 homolog	1.96E-05	1
PRRT1	proline rich transmembrane protein 1	1.96E-05	- 7.53E -01
ACTL6A	actin like 6A	1.96E-05	1.26
FAM210B	family with sequence similarity 210 member B	1.96E-05	-1.82
GLRX	glutaredoxin	1.96E-05	1.17
TMBIM4	transmembrane BAX inhibitor motif containing 4	1.97E-05	-1.32
POLA1	DNA polymerase alpha 1, catalytic subunit	1.97E-05	1.1
CFL2	cofilin 2	1.98E-05	6.53E -01
HAUS8	HAUS augmin like complex subunit 8	1.98E-05	1.32
POLD3	DNA polymerase delta 3, accessory subunit	1.98E-05	1.02
PON2	paraoxonase 2	1.99E-05	1.26
CLCA2	chloride channel accessory 2	1.99E-05	- 7.84E -01
HNRNPD	heterogeneous nuclear ribonucleoprotein D	1.99E-05	6.56E -01

GLMN	glomulin, FKBP associated protein	1.99E-05	1.05
LHX3	LIM homeobox 3	1.99E-05	-1.58
VPS28	VPS28, ESCRT-I subunit	2.00E-05	-1.09
FAM172A	family with sequence similarity 172 member A	2.02E-05	-1.04
EIF3F	eukaryotic translation initiation factor 3 subunit F	2.03E-05	-7.78E-01
EVA1B	eva-1 homolog B	2.03E-05	-1.31
ZFP82	ZFP82 zinc finger protein	2.04E-05	8.87E-01
KATNAL1	katanin catalytic subunit A1 like 1	2.04E-05	1.1
PTTG1	pituitary tumor-transforming 1	2.06E-05	2.43
SPRY4	sprouty RTK signaling antagonist 4	2.06E-05	-1.85
PRRX2	paired related homeobox 2	2.06E-05	6.16E-01
ZNF322	zinc finger protein 322	2.06E-05	7.61E-01
HLA-A	major histocompatibility complex, class I, A	2.07E-05	-8.50E-01
PGM3	phosphoglucomutase 3	2.07E-05	1.18
AMY2A	amylase, alpha 2A (pancreatic)	2.07E-05	-6.55E-01
OTUD6B	OTU domain containing 6B	2.09E-05	7.60E-01
RAB32	RAB32, member RAS oncogene family	2.09E-05	-7.90E-01
DUSP11	dual specificity phosphatase 11	2.09E-05	7.11E-01
PHOSPHO2	phosphatase, orphan 2	2.10E-05	5.75E-01

ERICH5	glutamate rich 5	2.11E-05	6.34E-01
C14orf28	chromosome 14 open reading frame 28	2.12E-05	-6.04E-01
SUMO3	small ubiquitin-like modifier 3	2.12E-05	1.09
PRKAB2	protein kinase AMP-activated non-catalytic subunit beta 2	2.13E-05	-6.45E-01
ZNF480	zinc finger protein 480	2.14E-05	7.65E-01
PCK2	phosphoenolpyruvate carboxykinase 2, mitochondrial	2.14E-05	-6.80E-01
SAAL1	serum amyloid A like 1	2.14E-05	1.11
TMEM59	transmembrane protein 59	2.15E-05	-7.25E-01
ANXA11	annexin A11	2.15E-05	-6.15E-01
CA11	carbonic anhydrase 11	2.16E-05	-1.7
CASP2	caspase 2	2.16E-05	8.76E-01
MPLKIP	M-phase specific PLK1 interacting protein	2.16E-05	1.16
ATP6V1G2	ATPase H+ transporting V1 subunit G2	2.17E-05	-9.77E-01
RFX7	regulatory factor X7	2.18E-05	-7.81E-01
KLF9	Kruppel like factor 9	2.18E-05	-1.12
CSRP2	cysteine and glycine rich protein 2	2.18E-05	1.21
ZFP90	ZFP90 zinc finger protein	2.20E-05	-1.17

SUCO	SUN domain containing ossification factor	2.21E-05	-<br>9.25E<br>-01
SCAI	suppressor of cancer cell invasion	2.21E-05	6.41E<br>-01
CEACAM1	carcinoembryonic antigen related cell adhesion molecule 1	2.21E-05	-<br>7.91E<br>-01
AARD	alanine and arginine rich domain containing protein	2.22E-05	-1.56
WDR76	WD repeat domain 76	2.23E-05	1.09
IRF1	interferon regulatory factor 1	2.23E-05	1.24
ZNF362	zinc finger protein 362	2.23E-05	-1.06
PDK2	pyruvate dehydrogenase kinase 2	2.24E-05	-<br>8.06E<br>-01
ZMYND15	zinc finger MYND-type containing 15	2.24E-05	5.96E<br>-01
PIGW	phosphatidylinositol glycan anchor biosynthesis class W	2.25E-05	1.05
TLE2	transducin like enhancer of split 2	2.25E-05	-<br>7.93E<br>-01
S100A1	S100 calcium binding protein A1	2.27E-05	-1.29
ZNF26	zinc finger protein 26	2.27E-05	7.54E<br>-01
EIF3H	eukaryotic translation initiation factor 3 subunit H	2.28E-05	-<br>6.74E<br>-01
PRKCZ	protein kinase C zeta	2.30E-05	-1.21
SATB1	SATB homeobox 1	2.31E-05	-<br>9.21E<br>-01
INCENP	inner centromere protein	2.31E-05	9.97E<br>-01

TADA2A	transcriptional adaptor 2A	2.31E-05	7.97E-01
EIF3L	eukaryotic translation initiation factor 3 subunit L	2.32E-05	-7.16E-01
ST5	suppression of tumorigenicity 5	2.33E-05	-1.04
NARS2	asparaginyl-tRNA synthetase 2, mitochondrial (putative)	2.33E-05	7.68E-01
TUBA1B	tubulin alpha 1b	2.33E-05	1.34
BAZ1A	bromodomain adjacent to zinc finger domain 1A	2.33E-05	1.15
CNOT1	CCR4-NOT transcription complex subunit 1	2.34E-05	1.19
ITFG2	integrin alpha FG-GAP repeat containing 2	2.34E-05	6.35E-01
SVEP1	sushi, von Willebrand factor type A, EGF and pentraxin domain containing 1	2.35E-05	6.87E-01
ENO3	enolase 3	2.37E-05	-2.1
ATPAF1	ATP synthase mitochondrial F1 complex assembly factor 1	2.37E-05	-9.85E-01
PRRT2	proline rich transmembrane protein 2	2.37E-05	-1.21
SNHG8	small nucleolar RNA host gene 8	2.37E-05	-1.21
ATM	ATM serine/threonine kinase	2.39E-05	-9.72E-01
FOXD1	forkhead box D1	2.40E-05	-1.16
DUT	deoxyuridine triphosphatase	2.40E-05	1.03
UTP14A	UTP14A small subunit processome component	2.41E-05	1.15
GOLT1A	golgi transport 1A	2.42E-05	6.04E-01
PCGF6	polycomb group ring finger 6	2.43E-05	1.1
ARL4A	ADP ribosylation factor like GTPase 4A	2.43E-05	1.66

RNF144A	ring finger protein 144A	2.45E-05	9.46E-01
BAX	BCL2 associated X, apoptosis regulator	2.45E-05	-2.8
CRABP2	cellular retinoic acid binding protein 2	2.46E-05	2.29
DUSP14	dual specificity phosphatase 14	2.46E-05	-7.97E-01
MED30	mediator complex subunit 30	2.46E-05	1.86
CENPF	centromere protein F	2.47E-05	8.22E-01
SIRT2	sirtuin 2	2.47E-05	-6.82E-01
RND3	Rho family GTPase 3	2.48E-05	1.17
RSC1A1	regulatory solute carrier protein, family 1, member 1	2.49E-05	9.07E-01
MGAT3	mannosyl (beta-1,4-)-glycoprotein beta-1,4-N-acetylglucosaminyltransferase	2.49E-05	-1.14
EXOSC6	exosome component 6	2.49E-05	1.12
CD40	CD40 molecule	2.50E-05	-9.88E-01
POLD2	DAN polymerase delta 2, accessory subunit	2.50E-05	9.50E-01
CDC16	cell division cycle 16	2.51E-05	-7.67E-01
KIF1BP	KIF1 binding protein	2.52E-05	1.5
MFAP2	microfibrillar associated protein 2	2.52E-05	6.19E-01
CD3EAP	CD3e molecule associated protein	2.54E-05	5.17E-01
ABCA7	ATP binding cassette subfamily A member 7	2.55E-05	-1.12
ARL6IP6	ADP ribosylation factor like GTPase 6 interacting protein 6	2.56E-05	9.09E-01

GNG4	G protein subunit gamma 4	2.56E-05	-1
INTS10	integrator complex subunit 10	2.56E-05	7.82E-01
POGLUT1	protein O-glucosyltransferase 1	2.57E-05	-8.10E-01
CCDC160	coiled-coil domain containing 160	2.57E-05	9.52E-01
BOLA3	bolA family member 3	2.58E-05	9.71E-01
TCEB1	transcription elongation factor B subunit 1	2.59E-05	7.86E-01
PPP2CA	protein phosphatase 2 catalytic subunit alpha	2.61E-05	7.31E-01
B3GALNT1	beta-1,3-N-acetylgalactosaminyltransferase 1 (globoside blood group)	2.61E-05	6.28E-01
MYB	MYB proto-oncogene, transcription factor	2.61E-05	5.49E-01
MPP1	membrane palmitoylated protein 1	2.61E-05	7.18E-01
PSMA4	proteasome subunit alpha 4	2.62E-05	7.85E-01
SEPSECS	Sep (O-phosphoserine) tRNA:Sec (selenocysteine) tRNA synthase	2.63E-05	7.15E-01
TUBB4B	tubulin beta 4B class IVb	2.63E-05	1.35
HOGA1	4-hydroxy-2-oxoglutarate aldolase 1	2.65E-05	-8.02E-01
ACTN4	actinin alpha 4	2.65E-05	5.21E-01
RPS29	ribosomal protein S29	2.67E-05	-2.29
RFC2	replication factor C subunit 2	2.67E-05	1.24
RPL28	ribosomal protein L28	2.68E-05	-1.3
SPAG1	sperm associated antigen 1	2.68E-05	8.92E-01

RER1	retention in endoplasmic reticulum sorting receptor 1	2.68E-05	-7.06E-01
ZC3H12B	zinc finger CCCH-type containing 12B	2.68E-05	-9.08E-01
CMSS1	cms1 ribosomal small subunit homolog (yeast)	2.68E-05	1.39
SMARCD3	SWI/SNF related, matrix associated, actin dependent regulator of chromatin, subfamily d, member 3	2.70E-05	-6.86E-01
UCN	urocortin	2.70E-05	-1.27
RPL37	ribosomal protein L37	2.71E-05	-1.15
PSMC2	proteasome 26S subunit, ATPase 2	2.71E-05	8.72E-01
MEOX1	mesenchyme homeobox 1	2.72E-05	6.06E-01
LMCD1	LIM and cysteine rich domains 1	2.73E-05	6.76E-01
Sep-06	septin 6	2.74E-05	-1.05
ASB7	ankyrin repeat and SOCS box containing 7	2.74E-05	6.77E-01
MDC1	mediator of DNA damage checkpoint 1	2.75E-05	9.82E-01
USP48	ubiquitin specific peptidase 48	2.76E-05	5.00E-01
UBTD1	ubiquitin domain containing 1	2.78E-05	-1.09
GLI4	GLI family zinc finger 4	2.78E-05	-6.53E-01
EIF3C	eukaryotic translation initiation factor 3 subunit C	2.79E-05	8.69E-01
ALDH3A2	aldehyde dehydrogenase 3 family member A2	2.79E-05	7.28E-01
PLA2G12A	phospholipase A2 group XIIA	2.80E-05	7.19E-01

SH3YL1	SH3 and SYLF domain containing 1	2.80E-05	-5.88E-01
PCDH7	protocadherin 7	2.80E-05	-1.79
TUBB2B	tubulin beta 2B class IIb	2.81E-05	5.07E-01
ST5	suppression of tumorigenicity 5	2.82E-05	-7.34E-01
VWA5A	von Willebrand factor A domain containing 5A	2.82E-05	8.07E-01
RPL13AP6	ribosomal protein L13a pseudogene 6	2.83E-05	-1.21
C3orf58	chromosome 3 open reading frame 58	2.83E-05	2.48
CMAS	cytidine monophosphate N-acetylneuraminic acid synthetase	2.84E-05	8.88E-01
LOC645166	lymphocyte-specific protein 1 pseudogene	2.84E-05	1.49
FABP5	fatty acid binding protein 5	2.84E-05	1.57
NAA16	N(alpha)-acetyltransferase 16, NatA auxiliary subunit	2.84E-05	7.52E-01
DNAJC1	DnaJ heat shock protein family (Hsp40) member C1	2.85E-05	1.22
RABL2A	RAB, member of RAS oncogene family-like 2A	2.86E-05	-6.62E-01
RFNG	RFNG O-fucosylpeptide 3-beta-N-acetylglucosaminyltransferase	2.86E-05	-6.73E-01
VMP1	vacuole membrane protein 1	2.86E-05	-7.90E-01
TAGLN2	transgelin 2	2.87E-05	1.32
SOD1	superoxide dismutase 1, soluble	2.87E-05	6.34E-01
TPM2	tropomyosin 2 (beta)	2.88E-05	6.84E-01

RTN4IP1	reticulon 4 interacting protein 1	2.88E-05	6.54E -01
DERA	deoxyribose-phosphate aldolase	2.89E-05	6.65E -01
TOM1L2	target of myb1 like 2 membrane trafficking protein	2.89E-05	6.56E -01
EPOR	erythropoietin receptor	2.90E-05	-1.53
TUBB	tubulin beta class I	2.91E-05	9.09E -01
RASL11B	RAS like family 11 member B	2.91E-05	6.62E -01
UPF3A	UPF3 regulator of nonsense transcripts homolog A (yeast)	2.91E-05	-1.71
DSTN	destrin, actin depolymerizing factor	2.93E-05	9.34E -01
EIF2S2	eukaryotic translation initiation factor 2 subunit beta	2.94E-05	- 5.59E -01
NUF2	NUF2, NDC80 kinetochore complex component	2.95E-05	9.52E -01
CYB5R4	cytochrome b5 reductase 4	2.96E-05	1.66
NLGN1	neuroligin 1	2.96E-05	8.48E -01
VASH1	vasohibin 1	2.96E-05	- 7.07E -01
FARP1	FERM, ARH/RhoGEF and pleckstrin domain protein 1	2.97E-05	-1.28
NUP54	nucleoporin 54	2.97E-05	1.15
PRPF4	pre-mRNA processing factor 4	2.97E-05	7.97E -01
TBC1D32	TBC1 domain family member 32	2.99E-05	- 9.04E -01
GPR19	G protein-coupled receptor 19	3.00E-05	7.10E -01

GOT1	glutamic-oxaloacetic transaminase 1	3.00E-05	-7.64E-01
STARD10	StAR related lipid transfer domain containing 10	3.02E-05	-1.08
CA14	carbonic anhydrase 14	3.03E-05	-6.08E-01
DONSON	downstream neighbor of SON	3.03E-05	9.74E-01
GLRX2	glutaredoxin 2	3.03E-05	7.91E-01
SPTSSA	serine palmitoyltransferase small subunit A	3.04E-05	-8.18E-01
HNRNPDL	heterogeneous nuclear ribonucleoprotein D like	3.04E-05	-9.55E-01
PAGR1	PAXIP1 associated glutamate rich protein 1	3.06E-05	8.16E-01
CSDC2	cold shock domain containing C2	3.06E-05	-9.29E-01
HSPB1	heat shock protein family B (small) member 1	3.07E-05	1.26
KPNA4	karyopherin subunit alpha 4	3.07E-05	9.31E-01
ECHDC2	enoyl-CoA hydratase domain containing 2	3.09E-05	-1.11
FAM196A	family with sequence similarity 196 member A	3.09E-05	-7.04E-01
NUAK2	NUAK family kinase 2	3.09E-05	8.92E-01
TMSB10	thymosin beta 10	3.12E-05	1.46
CCNE2	cyclin E2	3.14E-05	6.32E-01
FAM114A1	family with sequence similarity 114 member A1	3.15E-05	5.84E-01

POLE4	DNA polymerase epsilon 4, accessory subunit	3.16E-05	1.11
TM2D1	TM2 domain containing 1	3.16E-05	-6.61E-01
DTNA	dystrobrevin alpha	3.17E-05	6.86E-01
SHROOM1	shroom family member 1	3.17E-05	5.91E-01
TNRC6B	trinucleotide repeat containing 6B	3.18E-05	-1.04
SP110	SP110 nuclear body protein	3.18E-05	8.56E-01
PRUNE2	prune homolog 2	3.19E-05	5.89E-01
GALK1	galactokinase 1	3.19E-05	-1.08
OVOL2	ovo like zinc finger 2	3.21E-05	5.12E-01
GUSBP2	glucuronidase, beta pseudogene 2	3.21E-05	-1.37
PARP3	poly(ADP-ribose) polymerase family member 3	3.23E-05	-1.03
TMEM62	transmembrane protein 62	3.23E-05	1
TDRD3	tudor domain containing 3	3.23E-05	-1.73
SNCG	synuclein gamma	3.25E-05	-1.85
SERPINB8	serpin family B member 8	3.25E-05	5.63E-01
KLHL3	kelch like family member 3	3.30E-05	-1.19
IRF2BP2	interferon regulatory factor 2 binding protein 2	3.30E-05	-1.11
TUBA1C	tubulin alpha 1c	3.31E-05	1.57
UAP1	UDP-N-acetylglucosamine pyrophosphorylase 1	3.31E-05	6.08E-01
CCNG1	cyclin G1	3.33E-05	-1.06
GATAD2B	GATA zinc finger domain containing 2B	3.33E-05	-6.87E-01

ZNF256	zinc finger protein 256	3.35E-05	5.96E-01
RBM22	RNA binding motif protein 22	3.35E-05	6.29E-01
FANCB	Fanconi anemia complementation group B	3.36E-05	1.06
SLC41A2	solute carrier family 41 member 2	3.37E-05	1.1
TOPBP1	topoisomerase (DNA) II binding protein 1	3.37E-05	1.11
CLHC1	clathrin heavy chain linker domain containing 1	3.38E-05	-5.43E-01
COL5A1	collagen type V alpha 1 chain	3.39E-05	-1.11
DNMT3B	DNA methyltransferase 3 beta	3.40E-05	1.02
TENM3	teneurin transmembrane protein 3	3.40E-05	-7.80E-01
NUP93	nucleoporin 93	3.40E-05	1.02
ACAD10	acyl-CoA dehydrogenase family member 10	3.40E-05	-8.98E-01
TIMP1	TIMP metallopeptidase inhibitor 1	3.41E-05	6.08E-01
RNF207	ring finger protein 207	3.41E-05	-1.04
OSBPL10	oxysterol binding protein like 10	3.43E-05	7.68E-01
LAD1	ladinin 1	3.44E-05	5.41E-01
UBASH3B	ubiquitin associated and SH3 domain containing B	3.44E-05	-9.32E-01
ATP6V1B1	ATPase H+ transporting V1 subunit B1	3.44E-05	-7.00E-01
GSTA4	glutathione S-transferase alpha 4	3.44E-05	-8.42E-01

B4GALNT1	beta-1,4-N-acetyl-galactosaminyltransferase 1	3.45E-05	7.26E-01
PPIC	peptidylprolyl isomerase C	3.45E-05	-8.59E-01
GSTM2	glutathione S-transferase mu 2 (muscle)	3.45E-05	-1.52
LEPROTL1	leptin receptor overlapping transcript-like 1	3.50E-05	5.87E-01
STX7	syntaxin 7	3.50E-05	1.07
EEF1E1	eukaryotic translation elongation factor 1 epsilon 1	3.51E-05	1.04
STARD4	StAR related lipid transfer domain containing 4	3.51E-05	1.84
EBPL	emopamil binding protein like	3.52E-05	-1.62
POLG2	DNA polymerase gamma 2, accessory subunit	3.54E-05	-6.87E-01
DNAJB11	DnaJ heat shock protein family (Hsp40) member B11	3.56E-05	-7.46E-01
BUD13	BUD13 homolog	3.56E-05	7.99E-01
BIRC5	baculoviral IAP repeat containing 5	3.57E-05	6.18E-01
FAM72B	family with sequence similarity 72 member B	3.57E-05	1.59
RAP1B	RAP1B, member of RAS oncogene family	3.58E-05	7.45E-01
CARD10	caspase recruitment domain family member 10	3.58E-05	-9.65E-01
NAV2	neuron navigator 2	3.59E-05	7.16E-01
DAAM1	dishevelled associated activator of morphogenesis 1	3.59E-05	8.33E-01

FKBP10	FK506 binding protein 10	3.60E-05	-6.47E-01
CST3	cystatin C	3.61E-05	-8.91E-01
MIB2	mindbomb E3 ubiquitin protein ligase 2	3.63E-05	-9.31E-01
MC1R	melanocortin 1 receptor	3.63E-05	-1.6
NELL2	neural EGFL like 2	3.63E-05	9.28E-01
ALG11	ALG11, alpha-1,2-mannosyltransferase	3.64E-05	-8.20E-01
TRAK1	trafficking kinesin protein 1	3.65E-05	4.70E-01
GPR37	G protein-coupled receptor 37	3.68E-05	6.62E-01
HNRNPA1P33	heterogeneous nuclear ribonucleoprotein A1 pseudogene 33	3.70E-05	-9.29E-01
TGIF1	TGFB induced factor homeobox 1	3.70E-05	5.85E-01
CD79B	CD79b molecule	3.70E-05	-1.41
GIMAP2	GTPase, IMAP family member 2	3.71E-05	6.71E-01
ATPAF1	ATP synthase mitochondrial F1 complex assembly factor 1	3.71E-05	7.87E-01
TMEM14C	transmembrane protein 14C	3.72E-05	-7.53E-01
TPM1	tropomyosin 1 (alpha)	3.72E-05	7.48E-01
RFC1	replication factor C subunit 1	3.73E-05	1.2
PTCD2	pentatricopeptide repeat domain 2	3.73E-05	5.46E-01

WDR41	WD repeat domain 41	3.73E-05	-8.28E-01
MKRN3	makorin ring finger protein 3	3.74E-05	-1.27
WDR20	WD repeat domain 20	3.74E-05	-6.15E-01
UBE2Q2	ubiquitin conjugating enzyme E2 Q2	3.75E-05	8.25E-01
KIF18A	kinesin family member 18A	3.75E-05	5.13E-01
NIPAL3	NIPA like domain containing 3	3.76E-05	-1.06
C1orf53	chromosome 1 open reading frame 53	3.76E-05	-1.16
KIAA0825	KIAA0825	3.77E-05	5.65E-01
TBC1D17	TBC1 domain family member 17	3.78E-05	-7.36E-01
DMPK	dystrophia myotonica protein kinase	3.78E-05	-1.15
C5AR1	complement component 5a receptor 1	3.78E-05	5.74E-01
HIST1H2BC	histone cluster 1, H2bc	3.78E-05	-8.21E-01
SPIN4	spindlin family member 4	3.78E-05	6.90E-01
VHL	von Hippel-Lindau tumor suppressor	3.79E-05	-8.35E-01
SLC12A4	solute carrier family 12 member 4	3.81E-05	-1.35
GEMIN2	gem nuclear organelle associated protein 2	3.81E-05	1.11
TMEM45A	transmembrane protein 45A	3.81E-05	-7.40E-01
ZNF701	zinc finger protein 701	3.82E-05	5.25E-01

GLUD2	glutamate dehydrogenase 2	3.82E-05	6.55E-01
ART5	ADP-ribosyltransferase 5	3.85E-05	-8.99E-01
IFNGR2	interferon gamma receptor 2 (interferon gamma transducer 1)	3.87E-05	-6.08E-01
DHCR24	24-dehydrocholesterol reductase	3.88E-05	1.1
ANGPTL2	angiopoietin like 2	3.89E-05	1.02
MCRIP1	MAPK regulated corepressor interacting protein 1	3.90E-05	-1.03
GLTSCR2	glioma tumor suppressor candidate region gene 2	3.91E-05	-9.57E-01
PIP4K2A	phosphatidylinositol-5-phosphate 4-kinase type 2 alpha	3.91E-05	8.27E-01
STAMBPL1	STAM binding protein like 1	3.93E-05	9.58E-01
EXO1	exonuclease 1	3.94E-05	1.01
NCOA3	nuclear receptor coactivator 3	3.94E-05	1.23
PIGC	phosphatidylinositol glycan anchor biosynthesis class C	3.95E-05	-5.94E-01
FGD6	FYVE, RhoGEF and PH domain containing 6	3.96E-05	7.49E-01
CDR2	cerebellar degeneration related protein 2	3.97E-05	8.38E-01
ARHGAP11A	Rho GTPase activating protein 11A	3.97E-05	7.95E-01
CIAO1	cytosolic iron-sulfur assembly component 1	3.97E-05	-8.53E-01
FAM63B	family with sequence similarity 63 member B	3.97E-05	-1.18
OSTF1	osteoclast stimulating factor 1	3.98E-05	1.03

NUDT1	nudix hydrolase 1	3.99E-05	2.01
CLUAP1	clusterin associated protein 1	4.01E-05	8.20E-01
C6orf48	chromosome 6 open reading frame 48	4.01E-05	-1.03
STXBP5	syntaxin binding protein 5	4.03E-05	-1.71
RPF2	ribosome production factor 2 homolog	4.04E-05	7.94E-01
SCG5	secretogranin V	4.04E-05	1.01
CCDC28A	coiled-coil domain containing 28A	4.04E-05	8.92E-01
OSBPL6	oxysterol binding protein like 6	4.05E-05	8.14E-01
ZNF695	zinc finger protein 695	4.05E-05	6.42E-01
LRRC40	leucine rich repeat containing 40	4.06E-05	9.41E-01
MOB1A	MOB kinase activator 1A	4.06E-05	1.52
PI4K2B	phosphatidylinositol 4-kinase type 2 beta	4.07E-05	1.29
NOP2	NOP2 nucleolar protein	4.08E-05	6.40E-01
TMSB4X	thymosin beta 4, X-linked	4.09E-05	2.5
SDHAP2	succinate dehydrogenase complex flavoprotein subunit A pseudogene 2	4.12E-05	-7.10E-01
ARL6	ADP ribosylation factor like GTPase 6	4.13E-05	6.79E-01
POLR3C	RNA polymerase III subunit C	4.14E-05	1.13
HSPA14	heat shock protein family A (Hsp70) member 14	4.14E-05	6.74E-01
SYBU	syntabulin	4.15E-05	6.22E-01
DACH1	dachshund family transcription factor 1	4.15E-05	-4.66E-01

ADA	adenosine deaminase	4.17E-05	-9.67E-01
COMMD8	COMM domain containing 8	4.17E-05	1.16
ID2	inhibitor of DNA binding 2, HLH protein	4.18E-05	9.54E-01
GCH1	GTP cyclohydrolase 1	4.19E-05	9.40E-01
UGGT2	UDP-glucose glycoprotein glucosyltransferase 2	4.19E-05	-6.58E-01
RHNO1	RAD9-HUS1-RAD1 interacting nuclear orphan 1	4.20E-05	9.85E-01
ZNF280D	zinc finger protein 280D	4.21E-05	-7.32E-01
GMPPB	GDP-mannose pyrophosphorylase B	4.21E-05	6.79E-01
ITM2C	integral membrane protein 2C	4.22E-05	-9.80E-01
SLC48A1	solute carrier family 48 member 1	4.22E-05	9.60E-01
DTNA	dystrobrevin alpha	4.24E-05	7.18E-01
AP3B2	adaptor related protein complex 3 beta 2 subunit	4.24E-05	6.30E-01
HAND2	heart and neural crest derivatives expressed 2	4.25E-05	5.81E-01
PDE5A	phosphodiesterase 5A	4.25E-05	5.80E-01
PWP1	PWP1 homolog, endonuclein	4.26E-05	7.20E-01
WDHD1	WD repeat and HMG-box DNA binding protein 1	4.27E-05	1.11
DMTN	dematin actin binding protein	4.28E-05	6.27E-01

CNOT9	CCR4-NOT transcription complex subunit 9	4.28E-05	1.27
BCL2L12	BCL2 like 12	4.29E-05	5.70E-01
RBBP9	RB binding protein 9, serine hydrolase	4.29E-05	5.57E-01
METTL1	methyltransferase like 1	4.30E-05	1.22
CAMK2G	calcium/calmodulin dependent protein kinase II gamma	4.31E-05	-5.57E-01
MIR365A	microRNA 365a	4.32E-05	9.87E-01
HRASLS	HRAS like suppressor	4.38E-05	1.09
HGF	hepatocyte growth factor	4.39E-05	-1.5
BMI1	BMI1 proto-oncogene, polycomb ring finger	4.39E-05	1.61
MBD3L5	methyl-CpG binding domain protein 3 like 5	4.39E-05	-1.93
LHX6	LIM homeobox 6	4.39E-05	7.69E-01
TPK1	thiamin pyrophosphokinase 1	4.40E-05	6.95E-01
ANO10	anoctamin 10	4.43E-05	-6.14E-01
GMPR2	guanosine monophosphate reductase 2	4.43E-05	-5.51E-01
ANAPC10	anaphase promoting complex subunit 10	4.43E-05	1.6
RBBP4	RB binding protein 4, chromatin remodeling factor	4.44E-05	-9.17E-01
SMAD5	SMAD family member 5	4.45E-05	-1.08
SH3GL3	SH3 domain containing GRB2 like 3, endophilin A3	4.45E-05	8.37E-01
SLC7A2	solute carrier family 7 member 2	4.45E-05	-1.46
SNORA12	small nucleolar RNA, H/ACA box 12	4.45E-05	-2.33

HADHB	hydroxyacyl-CoA dehydrogenase/3-ketoacyl-CoA thiolase/enoyl-CoA hydratase (trifunctional protein), beta subunit	4.50E-05	-6.11E-01
TP53INP1	tumor protein p53 inducible nuclear protein 1	4.50E-05	-2.15
POFUT2	protein O-fucosyltransferase 2	4.53E-05	-7.89E-01
FBXW7	F-box and WD repeat domain containing 7	4.56E-05	-1.24
PIGF	phosphatidylinositol glycan anchor biosynthesis class F	4.57E-05	-9.23E-01
CIART	circadian associated repressor of transcription	4.57E-05	-1.93
TM2D1	TM2 domain containing 1	4.61E-05	-1.03
C17orf62	chromosome 17 open reading frame 62	4.63E-05	-8.62E-01
CD27-AS1	CD27 antisense RNA 1	4.65E-05	1.31
DEK	DEK proto-oncogene	4.65E-05	1.38
ILVBL	ilvB acetolactate synthase like	4.65E-05	-7.22E-01
IGBP1P1	immunoglobulin (CD79A) binding protein 1 pseudogene 1	4.66E-05	-8.58E-01
IL1RAP	interleukin 1 receptor accessory protein	4.67E-05	9.51E-01
RPA3	replication protein A3	4.68E-05	9.94E-01
GUSBP2	glucuronidase, beta pseudogene 2	4.69E-05	-1.16
IFI44L	interferon induced protein 44 like	4.69E-05	5.53E-01
RPA1	replication protein A1	4.70E-05	8.62E-01
LZIC	leucine zipper and CTNNBIP1 domain containing	4.72E-05	5.80E-01

RELB	RELB proto-oncogene, NF-kB subunit	4.73E-05	7.36E-01
CKS1B	CDC28 protein kinase regulatory subunit 1B	4.73E-05	1.81
RAD23B	RAD23 homolog B, nucleotide excision repair protein	4.75E-05	1.16
SLC2A14	solute carrier family 2 member 14	4.76E-05	-1.03
VPS13A	vacuolar protein sorting 13 homolog A	4.77E-05	5.52E-01
KCNK6	potassium two pore domain channel subfamily K member 6	4.80E-05	5.20E-01
ATAD3A	ATPase family, AAA domain containing 3A	4.81E-05	9.58E-01
FBXO22	F-box protein 22	4.82E-05	-1.68
ZNF763	zinc finger protein 763	4.83E-05	-7.82E-01
ARV1	ARV1 homolog, fatty acid homeostasis modulator	4.84E-05	-8.55E-01
CPED1	cadherin like and PC-esterase domain containing 1	4.85E-05	4.76E-01
FTSJ1	FtsJ RNA methyltransferase homolog 1 (E. coli)	4.86E-05	1.03
MAP2	microtubule associated protein 2	4.86E-05	5.54E-01
PREP	prolyl endopeptidase	4.87E-05	-1.13
GUK1	guanylate kinase 1	4.87E-05	-6.01E-01
MEAF6	MYST/Esa1 associated factor 6	4.88E-05	6.14E-01
AP1S3	adaptor related protein complex 1 sigma 3 subunit	4.88E-05	8.15E-01
CTPS1	CTP synthase 1	4.89E-05	1.19

RGL3	ral guanine nucleotide dissociation stimulator like 3	4.89E-05	-1.1
DPYSL3	dihydropyrimidinase like 3	4.90E-05	5.41E-01
HELLS	helicase, lymphoid-specific	4.90E-05	1.12
GGTLC2	gamma-glutamyltransferase light chain 2	4.92E-05	-6.93E-01
H3F3B	H3 histone, family 3B	4.92E-05	-1.11
EYA4	EYA transcriptional coactivator and phosphatase 4	4.92E-05	1.43
ACSF2	acyl-CoA synthetase family member 2	4.93E-05	-1.27
ALDH1A1	aldehyde dehydrogenase 1 family member A1	4.94E-05	5.73E-01
ODF2	outer dense fiber of sperm tails 2	4.95E-05	8.76E-01
CAPZB	capping actin protein of muscle Z-line beta subunit	4.95E-05	-1.17
SMPDL3A	sphingomyelin phosphodiesterase acid like 3A	4.97E-05	-1.22
SET	SET nuclear proto-oncogene	4.98E-05	7.87E-01
THY1	Thy-1 cell surface antigen	4.98E-05	-7.11E-01
FKBP6	FK506 binding protein 6	4.98E-05	-1.22
SRPX	sushi repeat containing protein, X-linked	4.99E-05	6.10E-01
ATE1	arginyltransferase 1	5.01E-05	4.78E-01
ATE1	arginyltransferase 1	5.01E-05	4.98E-01
GPAM	glycerol-3-phosphate acyltransferase, mitochondrial	5.03E-05	1.11

MAPK10	mitogen-activated protein kinase 10	5.03E-05	-5.40E-01
CSAD	cysteine sulfinic acid decarboxylase	5.05E-05	-8.57E-01
GRN	granulin	5.05E-05	-1.12
HMX2	H6 family homeobox 2	5.05E-05	8.29E-01
CDK5RAP2	CDK5 regulatory subunit associated protein 2	5.06E-05	7.06E-01
ANKRD10	ankyrin repeat domain 10	5.08E-05	-1.21
BORCS7	BLOC-1 related complex subunit 7	5.10E-05	-9.11E-01
CNKSR3	CNKSR family member 3	5.11E-05	9.07E-01
BRCC3	BRCA1/BRCA2-containing complex subunit 3	5.12E-05	6.81E-01
RTN4	reticulon 4	5.12E-05	-1.8
TLX1	T-cell leukemia homeobox 1	5.13E-05	5.01E-01
TUBA1C	tubulin alpha 1c	5.14E-05	1.3
COMMD8	COMM domain containing 8	5.16E-05	1.02
CYP4F2	cytochrome P450 family 4 subfamily F member 2	5.17E-05	-5.99E-01
ZNF114	zinc finger protein 114	5.18E-05	-1
GPAM	glycerol-3-phosphate acyltransferase, mitochondrial	5.18E-05	1.04
NEFH	neurofilament heavy polypeptide	5.19E-05	1.46
COQ10A	coenzyme Q10A	5.22E-05	-1.26
HSPB3	heat shock protein family B (small) member 3	5.23E-05	-1.13

TTYH2	tweety family member 2	5.24E-05	-7.15E-01
MAP2K6	mitogen-activated protein kinase kinase 6	5.26E-05	-8.27E-01
VCAM1	vascular cell adhesion molecule 1	5.27E-05	-5.78E-01
HIST1H2BD	histone cluster 1, H2bd	5.31E-05	-6.96E-01
ATRAID	all-trans retinoic acid induced differentiation factor	5.31E-05	-7.98E-01
RANBP10	RAN binding protein 10	5.36E-05	1.87
PAQR3	progestin and adipoQ receptor family member 3	5.37E-05	1.08
ARG2	arginase 2	5.38E-05	4.64E-01
DIAPH3	diaphanous related formin 3	5.38E-05	1.44
RGS10	regulator of G-protein signaling 10	5.38E-05	-7.10E-01
FAM171B	family with sequence similarity 171 member B	5.39E-05	1.87
COL4A5	collagen type IV alpha 5 chain	5.40E-05	-1.56
ESD	esterase D	5.41E-05	-7.84E-01
METTL10	methyltransferase like 10	5.41E-05	5.29E-01
RSRC1	arginine and serine rich coiled-coil 1	5.41E-05	8.36E-01
PODNL1	podocan like 1	5.42E-05	-7.98E-01

PDE9A	phosphodiesterase 9A	5.43E-05	-8.61E-01
RAB26	RAB26, member RAS oncogene family	5.44E-05	-1.21
IMPAD1	inositol monophosphatase domain containing 1	5.44E-05	4.88E-01
HLA-DOA	major histocompatibility complex, class II, DO alpha	5.47E-05	-5.99E-01
PRKRA	protein activator of interferon induced protein kinase EIF2AK2	5.48E-05	5.84E-01
CEP95	centrosomal protein 95	5.49E-05	-6.96E-01
HSBP1	heat shock factor binding protein 1	5.54E-05	-1.08
TTPAL	alpha tocopherol transfer protein like	5.55E-05	4.81E-01
MIER1	MIER1 transcriptional regulator	5.57E-05	-5.44E-01
PLS1	plastin 1	5.57E-05	1.33
WTAP	Wilms tumor 1 associated protein	5.57E-05	7.35E-01
BUB3	BUB3, mitotic checkpoint protein	5.57E-05	6.30E-01
ADAMTS19	ADAM metallopeptidase with thrombospondin type 1 motif 19	5.58E-05	4.81E-01
UHRF1	ubiquitin like with PHD and ring finger domains 1	5.60E-05	1.76
ING4	inhibitor of growth family member 4	5.62E-05	-7.44E-01
PKMYT1	protein kinase, membrane associated tyrosine/threonine 1	5.63E-05	7.35E-01
TRMT61A	tRNA methyltransferase 61A	5.64E-05	6.49E-01

CENPW	centromere protein W	5.65E-05	1.79
C4orf3	chromosome 4 open reading frame 3	5.69E-05	-1.34
ALG6	ALG6, alpha-1,3-glucosyltransferase	5.69E-05	1.03
DYNC1I2	dynein cytoplasmic 1 intermediate chain 2	5.69E-05	-1.15
COL6A2	collagen type VI alpha 2 chain	5.69E-05	-1.21
THBS3	thrombospondin 3	5.71E-05	-9.12E-01
HEXDC	hexosaminidase D	5.73E-05	-1.03
EIF5A2	eukaryotic translation initiation factor 5A2	5.74E-05	9.90E-01
MED20	mediator complex subunit 20	5.75E-05	4.77E-01
MAP9	microtubule associated protein 9	5.76E-05	1.27
PRR11	proline rich 11	5.76E-05	5.26E-01
NUP98	nucleoporin 98	5.80E-05	9.76E-01
TADA2A	transcriptional adaptor 2A	5.82E-05	6.50E-01
CASP9	caspase 9	5.82E-05	6.51E-01
NAE1	NEDD8 activating enzyme E1 subunit 1	5.84E-05	5.87E-01
HABP4	hyaluronan binding protein 4	5.86E-05	6.06E-01
LRRCC1	leucine rich repeat and coiled-coil centrosomal protein 1	5.87E-05	1.02
IQCB1	IQ motif containing B1	5.88E-05	6.29E-01
DHRS4	dehydrogenase/reductase 4	5.89E-05	-7.04E-01
MPHOSPH6	M-phase phosphoprotein 6	5.90E-05	1.25

WDR13	WD repeat domain 13	5.90E-05	-7.98E-01
GGA2	golgi associated, gamma adaptin ear containing, ARF binding protein 2	5.91E-05	-7.96E-01
ERCC1	ERCC excision repair 1, endonuclease non-catalytic subunit	5.91E-05	-7.27E-01
GPC3	glypican 3	5.93E-05	4.57E-01
JOSD1	Josephin domain containing 1	5.95E-05	7.38E-01
ZNF184	zinc finger protein 184	5.96E-05	8.86E-01
PDCD4	programmed cell death 4 (neoplastic transformation inhibitor)	5.98E-05	-9.15E-01
HMGN2	high mobility group nucleosomal binding domain 2	6.00E-05	6.92E-01
DYX1C1	dyslexia susceptibility 1 candidate 1	6.01E-05	8.28E-01
NSMAF	neutral sphingomyelinase activation associated factor	6.02E-05	8.58E-01
LOC100129055	cyclin Y like 1 pseudogene	6.02E-05	-5.67E-01
CBX8	chromobox 8	6.02E-05	-1.02
CENPN	centromere protein N	6.03E-05	1.85
MAPRE3	microtubule associated protein RP/EB family member 3	6.04E-05	-1.01
PCSK6	proprotein convertase subtilisin/kexin type 6	6.05E-05	6.54E-01
LRCH2	leucine rich repeats and calponin homology domain containing 2	6.06E-05	5.94E-01

AZIN2	antizyme inhibitor 2	6.06E-05	-7.93E-01
TRAPPC10	trafficking protein particle complex 10	6.07E-05	-7.15E-01
MCOLN3	mucolipin 3	6.07E-05	-9.59E-01
YWHAQ	tyrosine 3-monooxygenase/tryptophan 5-monooxygenase activation protein theta	6.08E-05	7.12E-01
CNIH1	cornichon family AMPA receptor auxiliary protein 1	6.09E-05	-1.05
ABHD5	abhydrolase domain containing 5	6.09E-05	9.98E-01
PRPS2	phosphoribosyl pyrophosphate synthetase 2	6.11E-05	1.09
PALM	paralemmin	6.11E-05	-1.62
MAP3K14	mitogen-activated protein kinase kinase kinase 14	6.11E-05	1.07
FAM65C	family with sequence similarity 65 member C	6.12E-05	-1.24
CELF1	CUGBP, Elav-like family member 1	6.14E-05	7.67E-01
FAM13A	family with sequence similarity 13 member A	6.14E-05	-1.42
SF1	splicing factor 1	6.16E-05	-7.50E-01
PLPPR4	phospholipid phosphatase related 4	6.17E-05	-2.18
ENOX2	ecto-NOX disulfide-thiol exchanger 2	6.17E-05	4.31E-01
SLC38A9	solute carrier family 38 member 9	6.20E-05	4.44E-01
NR2C1	nuclear receptor subfamily 2 group C member 1	6.20E-05	7.45E-01
EMP3	epithelial membrane protein 3	6.20E-05	-1.76
MIR181A2HG	MIR181A2 host gene	6.21E-05	-1.06

APPL1	adaptor protein, phosphotyrosine interacting with PH domain and leucine zipper 1	6.22E-05	7.26E -01
PRIMPOL	primase and DNA directed polymerase	6.22E-05	8.35E -01
UGCG	UDP-glucose ceramide glucosyltransferase	6.23E-05	-1.86
PRAMEF9	PRAME family member 9	6.23E-05	- 6.28E -01
DNAJC18	DnaJ heat shock protein family (Hsp40) member C18	6.24E-05	- 4.73E -01
ST13	suppression of tumorigenicity 13 (colon carcinoma) (Hsp70 interacting protein)	6.24E-05	-1.09
CCDC89	coiled-coil domain containing 89	6.25E-05	- 6.00E -01
SMC2	structural maintenance of chromosomes 2	6.26E-05	1.04
CAMLG	calcium modulating ligand	6.27E-05	- 6.52E -01
LGALS1	galectin 1	6.27E-05	-1.16
EIF5A2	eukaryotic translation initiation factor 5A2	6.28E-05	1.21
PARVA	parvin alpha	6.30E-05	9.23E -01
ZNF618	zinc finger protein 618	6.30E-05	-1.01
FLRT2	fibronectin leucine rich transmembrane protein 2	6.31E-05	5.81E -01
TNFRSF12A	TNF receptor superfamily member 12A	6.33E-05	8.03E -01
RAC3	ras-related C3 botulinum toxin substrate 3 (rho family, small GTP binding protein Rac3)	6.33E-05	-1.41
WWTR1	WW domain containing transcription regulator 1	6.34E-05	1.25
ZNF845	zinc finger protein 845	6.35E-05	8.16E -01

WDR24	WD repeat domain 24	6.37E-05	-<br>7.25E<br>-01
GALK2	galactokinase 2	6.38E-05	-1
CALM1	calmodulin 1	6.38E-05	1.22
PIGK	phosphatidylinositol glycan anchor biosynthesis class K	6.40E-05	8.42E<br>-01
GRTP1	growth hormone regulated TBC protein 1	6.41E-05	-<br>5.02E<br>-01
PRMT5	protein arginine methyltransferase 5	6.43E-05	7.06E<br>-01
IKBIP	IKBKB interacting protein	6.44E-05	-1.29
CCNB1IP1	cyclin B1 interacting protein 1	6.44E-05	-<br>7.96E<br>-01
IQCB1	IQ motif containing B1	6.44E-05	8.06E<br>-01
CERS4	ceramide synthase 4	6.45E-05	-<br>4.77E<br>-01
PSMC3IP	PSMC3 interacting protein	6.45E-05	1
PTRHD1	peptidyl-tRNA hydrolase domain containing 1	6.45E-05	-1.59
CHN1	chimerin 1	6.49E-05	1.39
TIMELESS	timeless circadian clock	6.51E-05	1.38
HMGN5	high mobility group nucleosome binding domain 5	6.52E-05	1.23
GPN3	GPN-loop GTPase 3	6.54E-05	1.32
S100A13	S100 calcium binding protein A13	6.55E-05	-<br>5.31E<br>-01
RANBP1	RAN binding protein 1	6.56E-05	1.19
FBRSL1	fibrosin like 1	6.58E-05	-<br>7.56E<br>-01

URGCP	upregulator of cell proliferation	6.59E-05	-<br>8.91E<br>-01
P2RY1	purinergic receptor P2Y1	6.62E-05	-<br>5.81E<br>-01
TPK1	thiamin pyrophosphokinase 1	6.63E-05	1.06
ARHGAP11B	Rho GTPase activating protein 11B	6.63E-05	9.02E<br>-01
KIF24	kinesin family member 24	6.63E-05	5.27E<br>-01
DIMT1	DIM1 dimethyladenosine transferase 1 homolog	6.69E-05	7.89E<br>-01
NOTCH4	notch 4	6.70E-05	4.34E<br>-01
PTEN	phosphatase and tensin homolog	6.72E-05	8.50E<br>-01
LINC00173	long intergenic non-protein coding RNA 173	6.73E-05	-<br>8.55E<br>-01
DPP7	dipeptidyl peptidase 7	6.73E-05	6.57E<br>-01
SNHG7	small nucleolar RNA host gene 7	6.75E-05	-1.85
RNF144A	ring finger protein 144A	6.76E-05	8.13E<br>-01
ANK1	ankyrin 1	6.77E-05	-<br>7.54E<br>-01
LOC100288798	uncharacterized LOC100288798	6.77E-05	9.44E<br>-01
NOL7	nucleolar protein 7	6.78E-05	6.55E<br>-01
PGBD2	piggyBac transposable element derived 2	6.79E-05	-<br>8.89E<br>-01
JPH1	junctophilin 1	6.81E-05	6.09E<br>-01

CXorf56	chromosome X open reading frame 56	6.82E-05	7.20E-01
LMO2	LIM domain only 2	6.83E-05	7.60E-01
RIN1	Ras and Rab interactor 1	6.85E-05	-7.55E-01
ANXA7	annexin A7	6.86E-05	7.77E-01
ZW10	zw10 kinetochore protein	6.86E-05	7.73E-01
LOC100419583	ring finger protein 4 pseudogene	6.86E-05	5.76E-01
FIP1L1	factor interacting with PAPOLA and CPSF1	6.87E-05	7.81E-01
RNF128	ring finger protein 128, E3 ubiquitin protein ligase	6.87E-05	7.53E-01
ETV1	ETS variant 1	6.87E-05	-5.10E-01
PSAT1	phosphoserine aminotransferase 1	6.89E-05	-5.88E-01
BAIAP2	BAI1 associated protein 2	6.90E-05	-9.53E-01
TRIM13	tripartite motif containing 13	6.91E-05	-9.02E-01
TMEM14C	transmembrane protein 14C	6.91E-05	-5.03E-01
SF3B4	splicing factor 3b subunit 4	6.93E-05	7.42E-01
POLR1D	RNA polymerase I subunit D	6.97E-05	-9.02E-01

DMKN	dermokine	6.99E-05	1.72
ATG3	autophagy related 3	6.99E-05	9.38E-01
ALDH3B1	aldehyde dehydrogenase 3 family member B1	7.01E-05	-6.92E-01
TEX30	testis expressed 30	7.02E-05	1.35
PHF21B	PHD finger protein 21B	7.03E-05	-4.96E-01
TTC12	tetratricopeptide repeat domain 12	7.04E-05	5.98E-01
ZAK	sterile alpha motif and leucine zipper containing kinase AZK	7.04E-05	1
PLA2G3	phospholipase A2 group III	7.05E-05	5.02E-01
VPS26B	VPS26, retromer complex component B	7.06E-05	-5.20E-01
DLK2	delta like non-canonical Notch ligand 2	7.07E-05	7.74E-01
F2R	coagulation factor II thrombin receptor	7.08E-05	-1.46
KIAA0895	KIAA0895	7.09E-05	1.22
LRRC8B	leucine rich repeat containing 8 family member B	7.12E-05	5.18E-01
RAD51D	RAD51 paralog D	7.14E-05	5.62E-01
SAR1B	secretion associated Ras related GTPase 1B	7.14E-05	8.11E-01
TTYH3	tweety family member 3	7.19E-05	-1.56
GRIN3B	glutamate ionotropic receptor NMDA type subunit 3B	7.20E-05	-1.26
HLA-DPA1	major histocompatibility complex, class II, DP alpha 1	7.21E-05	-1.21
ESPNL	espin-like	7.23E-05	-1.29

PPIG	peptidylprolyl isomerase G	7.24E-05	8.88E-01
PLK1	polo like kinase 1	7.28E-05	1.32
DCAKD	dephospho-CoA kinase domain containing	7.29E-05	-1.06
PPA1	pyrophosphatase (inorganic) 1	7.29E-05	4.83E-01
CXCL16	C-X-C motif chemokine ligand 16	7.30E-05	1.68
PXYLP1	2-phosphoxylose phosphatase 1	7.33E-05	5.43E-01
RABGGTB	Rab geranylgeranyltransferase beta subunit	7.33E-05	-5.35E-01
RPAP3	RNA polymerase II associated protein 3	7.35E-05	1.15
CEP85L	centrosomal protein 85 like	7.37E-05	-6.28E-01
AEN	apoptosis enhancing nuclease	7.44E-05	-6.99E-01
ADK	adenosine kinase	7.44E-05	-9.68E-01
SLC10A4	solute carrier family 10 member 4	7.44E-05	6.01E-01
ZNF354A	zinc finger protein 354A	7.46E-05	8.19E-01
ZNF84	zinc finger protein 84	7.47E-05	-6.08E-01
RNF115	ring finger protein 115	7.47E-05	8.03E-01
NAE1	NEDD8 activating enzyme E1 subunit 1	7.50E-05	8.47E-01
SGSH	N-sulfoglucosamine sulfohydrolase	7.50E-05	-1.45

FBLN5	fibulin 5	7.52E-05	-8.30E-01
UTP4	UTP4, small subunit processome component	7.55E-05	6.11E-01
PCDH11X	protocadherin 11 X-linked	7.56E-05	-5.12E-01
SNORD96B	small nucleolar RNA, C/D box 96B	7.56E-05	9.26E-01
PLCXD1	phosphatidylinositol specific phospholipase C X domain containing 1	7.58E-05	-1.15
PPM1F	protein phosphatase, Mg2+/Mn2+ dependent 1F	7.59E-05	-1.28
UFD1L	ubiquitin fusion degradation 1 like (yeast)	7.59E-05	5.66E-01
ANKRD37	ankyrin repeat domain 37	7.63E-05	-2.02
FPR1	formyl peptide receptor 1	7.65E-05	4.21E-01
SERPINB8	serpin family B member 8	7.66E-05	4.88E-01
KRIT1	KRIT1, ankyrin repeat containing	7.67E-05	-7.76E-01
ZNF561	zinc finger protein 561	7.67E-05	-1.28
ZFP69B	ZFP69 zinc finger protein B	7.67E-05	7.02E-01
GPR108	G protein-coupled receptor 108	7.69E-05	-1.11
KCNV1	potassium voltage-gated channel modifier subfamily V member 1	7.73E-05	4.57E-01
RPL32	ribosomal protein L32	7.73E-05	-6.51E-01
OGFOD1	2-oxoglutarate and iron dependent oxygenase domain containing 1	7.73E-05	5.64E-01
RUNX3	runt related transcription factor 3	7.75E-05	-1.36

DIAPH3	diaphanous related formin 3	7.76E-05	7.97E-01
LAMA2	laminin subunit alpha 2	7.77E-05	6.14E-01
PPIH	peptidylprolyl isomerase H	7.79E-05	1.48
TANK	TRAF family member associated NFKB activator	7.79E-05	-7.24E-01
GPS1	G protein pathway suppressor 1	7.82E-05	-6.52E-01
PSMB2	proteasome subunit beta 2	7.82E-05	8.37E-01
MRPL1	mitochondrial ribosomal protein L1	7.88E-05	9.70E-01
ODF2	outer dense fiber of sperm tails 2	7.88E-05	5.76E-01
LOC81691	exonuclease NEF-sp	7.91E-05	8.41E-01
C16orf59	chromosome 16 open reading frame 59	7.92E-05	1.31
KDELR1	KDEL endoplasmic reticulum protein retention receptor 1	7.92E-05	-6.15E-01
BRI3BP	BRI3 binding protein	7.92E-05	1.18
PPARG	peroxisome proliferator activated receptor gamma	7.94E-05	4.97E-01
KIF1BP	KIF1 binding protein	7.95E-05	1.51
PTGER1	prostaglandin E receptor 1	7.96E-05	5.16E-01
AMZ2	archaelysin family metallopeptidase 2	7.96E-05	-5.07E-01
TAF9B	TATA-box binding protein associated factor 9b	7.97E-05	1.31

CHKA	choline kinase alpha	7.97E-05	-8.54E-01
C12orf76	chromosome 12 open reading frame 76	7.98E-05	-1.2
C16orf92	chromosome 16 open reading frame 92	7.99E-05	8.98E-01
GPALPP1	GPALPP motifs containing 1	8.02E-05	6.00E-01
ZNF200	zinc finger protein 200	8.02E-05	6.93E-01
ARMCX6	armadillo repeat containing, X-linked 6	8.02E-05	1.98
MEGF10	multiple EGF like domains 10	8.06E-05	-7.27E-01
BBS1	Bardet-Biedl syndrome 1	8.06E-05	-9.68E-01
YWHAH	tyrosine 3-monooxygenase/tryptophan 5-monooxygenase activation protein eta	8.11E-05	6.75E-01
ALG10B	ALG10B, alpha-1,2-glucosyltransferase	8.12E-05	7.81E-01
LEPR	leptin receptor	8.12E-05	6.33E-01
TEX19	testis expressed 19	8.14E-05	-1.04
JMJD1C	jumonji domain containing 1C	8.15E-05	-5.87E-01
DPP7	dipeptidyl peptidase 7	8.17E-05	6.65E-01
DNAAF3	dynein axonemal assembly factor 3	8.17E-05	-1.28
TRIM8	tripartite motif containing 8	8.18E-05	-1.2
AFF3	AF4/FMR2 family member 3	8.18E-05	5.07E-01
MDM2	MDM2 proto-oncogene	8.19E-05	-1.11
G6PC3	glucose-6-phosphatase catalytic subunit 3	8.19E-05	-1.15

RSPH9	radial spoke head 9 homolog	8.22E-05	-6.97E-01
OASL	2'-5'-oligoadenylate synthetase like	8.23E-05	4.00E-01
RIDA	reactive intermediate imine deaminase A homolog	8.25E-05	8.62E-01
MRPL3	mitochondrial ribosomal protein L3	8.30E-05	1.24
PGBD1	piggyBac transposable element derived 1	8.30E-05	6.19E-01
NPEPL1	aminopeptidase-like 1	8.32E-05	-9.25E-01
TMEM144	transmembrane protein 144	8.32E-05	-5.05E-01
SPATA20	spermatogenesis associated 20	8.33E-05	-1.48
CNOT1	CCR4-NOT transcription complex subunit 1	8.37E-05	9.02E-01
DLG2	discs large MAGUK scaffold protein 2	8.37E-05	-6.53E-01
MGAT4C	MGAT4 family member C	8.37E-05	-5.37E-01
VAMP2	vesicle associated membrane protein 2	8.37E-05	-1.16
LAPTM4B	lysosomal protein transmembrane 4 beta	8.41E-05	-7.85E-01
JAZF1	JAZF zinc finger 1	8.42E-05	6.20E-01
PLCG1	phospholipase C gamma 1	8.45E-05	-1.53
DIAPH3	diaphanous related formin 3	8.45E-05	5.46E-01
HSD17B12	hydroxysteroid 17-beta dehydrogenase 12	8.46E-05	-1.17

LRPAP1	LDL receptor related protein associated protein 1	8.47E-05	-1.23
MYO6	myosin VI	8.47E-05	6.83E-01
ISCU	iron-sulfur cluster assembly enzyme	8.49E-05	-1.63
TMEM88	transmembrane protein 88	8.49E-05	-1.63
VPS37A	VPS37A, ESCRT-I subunit	8.49E-05	7.56E-01
TRIM21	tripartite motif containing 21	8.52E-05	7.62E-01
C12orf76	chromosome 12 open reading frame 76	8.54E-05	-1.38
TNFRSF11B	TNF receptor superfamily member 11b	8.57E-05	-4.91E-01
FBLN2	fibulin 2	8.57E-05	-8.12E-01
TMEM167A	transmembrane protein 167A	8.57E-05	8.23E-01
RAB1A	RAB1A, member RAS oncogene family	8.60E-05	-6.97E-01
TRIM49C	tripartite motif containing 49C	8.65E-05	-7.65E-01
OBSCN	obscurin, cytoskeletal calmodulin and titin-interacting RhoGEF	8.65E-05	-6.24E-01
ASTN2	astrotactin 2	8.66E-05	-5.89E-01
SMU1	DNA replication regulator and spliceosomal factor	8.67E-05	6.51E-01
RPL13P5	ribosomal protein L13 pseudogene 5	8.67E-05	-8.00E-01

WDYHV1	WDYHV motif containing 1	8.69E-05	7.19E-01
MID2	midline 2	8.72E-05	-5.59E-01
HNRNPA1P10	heterogeneous nuclear ribonucleoprotein A1 pseudogene 10	8.72E-05	-8.69E-01
YPEL5	yippee like 5	8.73E-05	-1.38
SLC25A35	solute carrier family 25 member 35	8.73E-05	-6.05E-01
TATDN3	TatD DNase domain containing 3	8.75E-05	-4.60E-01
TSKU	tsukushi, small leucine rich proteoglycan	8.80E-05	-1.35
CARMIL2	capping protein regulator and myosin 1 linker 2	8.82E-05	7.74E-01
SKAP1	src kinase associated phosphoprotein 1	8.92E-05	5.42E-01
SNORD56	small nucleolar RNA, C/D box 56	8.94E-05	4.44E-01
CYP4F3	cytochrome P450 family 4 subfamily F member 3	8.95E-05	-4.77E-01
ZNF608	zinc finger protein 608	8.96E-05	-1.56
TMEM54	transmembrane protein 54	8.99E-05	8.95E-01
ITGB5	integrin subunit beta 5	9.02E-05	8.15E-01
HERC6	HECT and RLD domain containing E3 ubiquitin protein ligase family member 6	9.05E-05	9.72E-01
CMTM3	CKLF like MARVEL transmembrane domain containing 3	9.07E-05	-5.08E-01

PRKD1	protein kinase D1	9.07E-05	-7.43E-01
GPALPP1	GPALPP motifs containing 1	9.10E-05	9.24E-01
FOXL2NB	FOXL2 neighbor	9.11E-05	-1.11
LOC146880	Rho GTPase activating protein 27 pseudogene	9.12E-05	1.07
PARP9	poly(ADP-ribose) polymerase family member 9	9.14E-05	5.64E-01
ISYNA1	inositol-3-phosphate synthase 1	9.15E-05	-9.08E-01
CDK5RAP2	CDK5 regulatory subunit associated protein 2	9.15E-05	1.11
MED21	mediator complex subunit 21	9.16E-05	7.90E-01
MRPS35	mitochondrial ribosomal protein S35	9.16E-05	6.53E-01
ANXA2R	annexin A2 receptor	9.17E-05	-1.32
ATP6AP1L	ATPase H+ transporting accessory protein 1 like	9.17E-05	-8.94E-01
RNASEK	ribonuclease K	9.21E-05	-5.40E-01
TRNT1	tRNA nucleotidyl transferase 1	9.22E-05	7.18E-01
EZH2	enhancer of zeste 2 polycomb repressive complex 2 subunit	9.26E-05	1.42
CADM2	cell adhesion molecule 2	9.27E-05	7.50E-01
ZBTB44	zinc finger and BTB domain containing 44	9.27E-05	-1.22
GABPB1	GA binding protein transcription factor beta subunit 1	9.29E-05	8.65E-01
HBA2	hemoglobin subunit alpha 2	9.30E-05	4.80E-01

ITGA2	integrin subunit alpha 2	9.30E-05	9.03E-01
ATP9A	ATPase phospholipid transporting 9A (putative)	9.31E-05	1.1
USP47	ubiquitin specific peptidase 47	9.31E-05	-4.29E-01
SLC16A5	solute carrier family 16 member 5	9.33E-05	-1.3
RPA1	replication protein A1	9.33E-05	7.84E-01
EMBP1	embigin pseudogene 1	9.35E-05	1.01
HIRIP3	HIRA interacting protein 3	9.37E-05	1.03
ABCB1	ATP binding cassette subfamily B member 1	9.37E-05	7.22E-01
PSTK	phosphoseryl-tRNA kinase	9.40E-05	7.93E-01
MBOAT1	membrane bound O-acyltransferase domain containing 1	9.45E-05	5.70E-01
TMEM9	transmembrane protein 9	9.47E-05	-5.60E-01
RPA1	replication protein A1	9.49E-05	8.31E-01
KIAA1804	mixed lineage kinase 4	9.49E-05	5.97E-01
GABRD	gamma-aminobutyric acid type A receptor delta subunit	9.50E-05	-4.59E-01
SUOX	sulfite oxidase	9.53E-05	-7.52E-01
CNNM3	cyclin and CBS domain divalent metal cation transport mediator 3	9.53E-05	4.67E-01
ATP8B4	ATPase phospholipid transporting 8B4 (putative)	9.53E-05	-6.16E-01

ANXA2P1	annexin A2 pseudogene 1	9.56E-05	8.21E-01
CTGF	connective tissue growth factor	9.57E-05	7.95E-01
PTOV1	prostate tumor overexpressed 1	9.62E-05	-6.86E-01
UCHL5	ubiquitin C-terminal hydrolase L5	9.63E-05	9.24E-01
TMEM145	transmembrane protein 145	9.71E-05	-1.02
FKBP11	FK506 binding protein 11	9.71E-05	-8.36E-01
SULF2	sulfatase 2	9.72E-05	-1.67
ISPD	isoprenoid synthase domain containing	9.73E-05	6.03E-01
ZSWIM6	zinc finger SWIM-type containing 6	9.73E-05	-9.34E-01
CCNG2	cyclin G2	9.76E-05	-7.47E-01
ANKZF1	ankyrin repeat and zinc finger domain containing 1	9.76E-05	-6.49E-01
FAAP20	Fanconi anemia core complex associated protein 20	9.78E-05	-1.44
LINC01278	long intergenic non-protein coding RNA 1278	9.78E-05	5.20E-01
ING3	inhibitor of growth family member 3	9.79E-05	9.69E-01
SIX3	SIX homeobox 3	9.81E-05	8.53E-01
AP2A1	adaptor related protein complex 2 alpha 1 subunit	9.82E-05	-6.24E-01
CCNF	cyclin F	9.83E-05	1.72

PI15	peptidase inhibitor 15	9.87E-05	-8.35E-01
CEL	carboxyl ester lipase	9.88E-05	-8.68E-01
PDE1A	phosphodiesterase 1A	9.90E-05	-7.09E-01
PGRMC2	progesterone receptor membrane component 2	9.92E-05	-5.12E-01
CYGB	cytoglobin	9.92E-05	-5.34E-01
RBPJ	recombination signal binding protein for immunoglobulin kappa J region	9.95E-05	-7.02E-01
MIB2	mindbomb E3 ubiquitin protein ligase 2	1.00E-04	-9.12E-01
PSMD12	proteasome 26S subunit, non-ATPase 12	1.01E-04	1.05
IDH2	isocitrate dehydrogenase (NADP(+)) 2, mitochondrial	1.01E-04	-8.34E-01
MPP6	membrane palmitoylated protein 6	1.01E-04	6.33E-01
RBM28	RNA binding motif protein 28	1.01E-04	5.54E-01
ATG4C	autophagy related 4C cysteine peptidase	1.01E-04	7.58E-01
CFAP47	cilia and flagella associated protein 47	1.01E-04	4.47E-01
PPP3CC	protein phosphatase 3 catalytic subunit gamma	1.02E-04	6.56E-01
DCK	deoxycytidine kinase	1.02E-04	6.75E-01

MXRA8	matrix remodeling associated 8	1.02E-04	-1.75
ISOC2	isochorismatase domain containing 2	1.02E-04	-9.14E-01
RACGAP1	Rac GTPase activating protein 1	1.02E-04	6.62E-01
TPD52L2	tumor protein D52 like 2	1.02E-04	-5.55E-01
ADGRE5	adhesion G protein-coupled receptor E5	1.02E-04	-9.31E-01
STK33	serine/threonine kinase 33	1.02E-04	5.78E-01
MGAT4A	mannosyl (alpha-1,3-)-glycoprotein beta-1,4-N-acetylglucosaminyltransferase, isozyme A	1.02E-04	6.13E-01
METTL1	methyltransferase like 1	1.02E-04	1.37
CENPH	centromere protein H	1.03E-04	7.85E-01
CCDC112	coiled-coil domain containing 112	1.03E-04	9.05E-01
AGPS	alkylglycerone phosphate synthase	1.03E-04	8.96E-01
RPS6KA1	ribosomal protein S6 kinase A1	1.04E-04	-7.18E-01
NME7	NME/NM23 family member 7	1.04E-04	9.62E-01
DAB2IP	DAB2 interacting protein	1.04E-04	-5.55E-01
WASH1	WAS protein family homolog 1	1.04E-04	-8.72E-01
LOC100240735	uncharacterized LOC100240735	1.04E-04	-1.23
SCRIB	scribbled planar cell polarity protein	1.04E-04	-1.2

SLC20A2	solute carrier family 20 member 2	1.04E-04	-4.83E-01
LUC7L	LUC7 like	1.05E-04	-6.43E-01
GPR137B	G protein-coupled receptor 137B	1.05E-04	7.17E-01
CCND3	cyclin D3	1.05E-04	-1.08
C16orf86	chromosome 16 open reading frame 86	1.05E-04	-7.26E-01
ATRIP	ATR interacting protein	1.06E-04	7.71E-01
MAFF	MAF bZIP transcription factor F	1.06E-04	9.18E-01
RAC1	ras-related C3 botulinum toxin substrate 1 (rho family, small GTP binding protein Rac1)	1.06E-04	4.76E-01
IDI1	isopentenyl-diphosphate delta isomerase 1	1.06E-04	7.87E-01
ZDHHC21	zinc finger DHHC-type containing 21	1.06E-04	7.39E-01
ZNF200	zinc finger protein 200	1.06E-04	7.21E-01
CALY	calcyon neuron specific vesicular protein	1.06E-04	-1.36
CPS1	carbamoyl-phosphate synthase 1	1.07E-04	-6.32E-01
GLIDR	glioblastoma down-regulated RNA	1.07E-04	-8.64E-01
RSRP1	arginine and serine rich protein 1	1.07E-04	-9.49E-01
ZNF467	zinc finger protein 467	1.07E-04	-1.37
MYL9	myosin light chain 9	1.08E-04	6.21E-01

VPS37D	VPS37D, ESCRT-I subunit	1.08E-04	-1.16
PDGFRA	platelet derived growth factor receptor alpha	1.08E-04	-7.88E-01
TTLL7	tubulin tyrosine ligase like 7	1.08E-04	5.93E-01
TM9SF2	transmembrane 9 superfamily member 2	1.08E-04	-1.11
SIRT4	sirtuin 4	1.08E-04	-1.11
YWHAB	tyrosine 3-monooxygenase/tryptophan 5-monooxygenase activation protein beta	1.08E-04	6.36E-01
PSMA1	proteasome subunit alpha 1	1.09E-04	4.35E-01
MTHFD1	methylenetetrahydrofolate dehydrogenase, cyclohydrolase and formyltetrahydrofolate synthetase 1	1.09E-04	1.07
ENSA	endosulfine alpha	1.09E-04	8.22E-01
EXOC7	exocyst complex component 7	1.09E-04	-1.35
MYL5	myosin light chain 5	1.09E-04	-1.02
PRKX	protein kinase, X-linked	1.10E-04	-8.96E-01
INTS2	integrator complex subunit 2	1.10E-04	-7.89E-01
ARMCX2	armadillo repeat containing, X-linked 2	1.10E-04	4.54E-01
ARTN	artemin	1.10E-04	4.70E-01
SMA4	glucuronidase beta pseudogene	1.10E-04	-5.26E-01
STOX1	storkhead box 1	1.10E-04	6.83E-01
PFKFB4	6-phosphofructo-2-kinase/fructose-2,6-biphosphatase 4	1.10E-04	-1.69

EXOG	endo/exonuclease (5'-3'), endonuclease G-like	1.10E-04	7.64E-01
RBPMS	RNA binding protein with multiple splicing	1.10E-04	-6.60E-01
NBEA	neurobeachin	1.10E-04	8.88E-01
RHOBTB1	Rho related BTB domain containing 1	1.11E-04	-1.47
ALPK1	alpha kinase 1	1.11E-04	4.94E-01
CHCHD7	coiled-coil-helix-coiled-coil-helix domain containing 7	1.11E-04	-6.93E-01
LSM5	LSM5 homolog, U6 small nuclear RNA and mRNA degradation associated	1.11E-04	8.02E-01
SHMT1	serine hydroxymethyltransferase 1	1.11E-04	1.29
TRAPPC6B	trafficking protein particle complex 6B	1.11E-04	6.48E-01
LMF1	lipase maturation factor 1	1.12E-04	-6.86E-01
KIF1A	kinesin family member 1A	1.12E-04	7.78E-01
ABHD14B	abhydrolase domain containing 14B	1.12E-04	-6.32E-01
ALAS1	5'-aminolevulinate synthase 1	1.12E-04	4.37E-01
NKX3-1	NK3 homeobox 1	1.12E-04	4.89E-01
RBL1	RB transcriptional corepressor like 1	1.12E-04	6.54E-01
LIMS2	LIM zinc finger domain containing 2	1.13E-04	4.36E-01
L3MBTL1	l(3)mbt-like 1 (Drosophila)	1.13E-04	-5.45E-01

AZI2	5-azacytidine induced 2	1.13E-04	-6.21E-01
WSB1	WD repeat and SOCS box containing 1	1.13E-04	-9.56E-01
ACVR1	activin A receptor type 1	1.13E-04	-9.65E-01
UTP14A	UTP14A small subunit processome component	1.14E-04	9.04E-01
DNAH14	dynein axonemal heavy chain 14	1.14E-04	-7.12E-01
PXN	paxillin	1.14E-04	8.79E-01
ZC3H15	zinc finger CCCH-type containing 15	1.15E-04	1.24
MDK	midkine (neurite growth-promoting factor 2)	1.15E-04	6.85E-01
GMPS	guanine monophosphate synthase	1.15E-04	6.38E-01
NEIL2	nei like DNA glycosylase 2	1.15E-04	5.13E-01
PTMA	prothymosin, alpha	1.16E-04	5.03E-01
ARID2	AT-rich interaction domain 2	1.16E-04	-6.89E-01
PDCD2L	programmed cell death 2 like	1.16E-04	1.07
BAG4	BCL2 associated athanogene 4	1.16E-04	5.53E-01
PRRG1	proline rich and Gla domain 1	1.17E-04	-8.40E-01
RNH1	ribonuclease/angiogenin inhibitor 1	1.17E-04	5.30E-01

PDIA3P1	protein disulfide isomerase family A member 3 pseudogene 1	1.17E-04	-8.35E-01
PCBP4	poly(rC) binding protein 4	1.17E-04	-6.97E-01
SAMD8	sterile alpha motif domain containing 8	1.17E-04	6.18E-01
CSTF2T	cleavage stimulation factor subunit 2, tau variant	1.17E-04	5.03E-01
EVI5L	ecotropic viral integration site 5 like	1.18E-04	-1.29
C5orf34	chromosome 5 open reading frame 34	1.18E-04	9.33E-01
GSN	gelsolin	1.19E-04	-6.69E-01
RAB4A	RAB4A, member RAS oncogene family	1.19E-04	-6.47E-01
ZNF160	zinc finger protein 160	1.19E-04	-8.85E-01
ZNF484	zinc finger protein 484	1.19E-04	5.31E-01
CKS2	CDC28 protein kinase regulatory subunit 2	1.19E-04	8.19E-01
LAMC1	laminin subunit gamma 1	1.19E-04	-1.29
KLHL13	kelch like family member 13	1.19E-04	8.84E-01
TAP1	transporter 1, ATP binding cassette subfamily B member	1.20E-04	5.59E-01
NOLC1	nucleolar and coiled-body phosphoprotein 1	1.20E-04	1.16
ETS1	ETS proto-oncogene 1, transcription factor	1.20E-04	-7.75E-01
MLLT11	myeloid/lymphoid or mixed-lineage leukemia; translocated to, 11	1.20E-04	-1

MBL2	mannose binding lectin 2	1.20E-04	-7.08E-01
HHEX	hematopoietically expressed homeobox	1.20E-04	5.44E-01
CDS1	CDP-diacylglycerol synthase 1	1.21E-04	9.02E-01
AZIN1	antizyme inhibitor 1	1.21E-04	1.34
TMEM170B	transmembrane protein 170B	1.21E-04	4.77E-01
TPP1	tripeptidyl peptidase 1	1.21E-04	-5.98E-01
GLRX3	glutaredoxin 3	1.21E-04	6.57E-01
NYNRIN	NYN domain and retroviral integrase containing	1.21E-04	-1.12
PSMD11	proteasome 26S subunit, non-ATPase 11	1.21E-04	7.77E-01
CCNC	cyclin C	1.22E-04	7.07E-01
USP18	ubiquitin specific peptidase 18	1.22E-04	1.1
CDC16	cell division cycle 16	1.22E-04	-6.02E-01
ELOVL7	ELOVL fatty acid elongase 7	1.22E-04	4.77E-01
RNASEH2B	ribonuclease H2 subunit B	1.22E-04	6.91E-01
EXTL1	exostosin like glycosyltransferase 1	1.22E-04	-6.34E-01
KIAA0232	KIAA0232	1.23E-04	-5.91E-01
PDGFB	platelet derived growth factor subunit B	1.23E-04	4.43E-01

TIMM21	translocase of inner mitochondrial membrane 21	1.23E-04	-4.14E-01
ECH1	enoyl-CoA hydratase 1	1.23E-04	-6.15E-01
TM6SF1	transmembrane 6 superfamily member 1	1.23E-04	6.90E-01
SLITRK3	SLIT and NTRK like family member 3	1.23E-04	-5.40E-01
DDR1	discoidin domain receptor tyrosine kinase 1	1.23E-04	1.11
SMCO4	single-pass membrane protein with coiled-coil domains 4	1.24E-04	1.19
CYSRT1	cysteine rich tail 1	1.24E-04	-1.74
PPP2R2B	protein phosphatase 2 regulatory subunit Bbeta	1.24E-04	-8.27E-01
NUPR1	nuclear protein 1, transcriptional regulator	1.24E-04	-5.14E-01
JAM2	junctional adhesion molecule 2	1.24E-04	5.95E-01
CSNK1A1L	casein kinase 1 alpha 1 like	1.25E-04	7.82E-01
DPH6	diphthamine biosynthesis 6	1.25E-04	-5.87E-01
NRAS	neuroblastoma RAS viral oncogene homolog	1.26E-04	8.56E-01
METRNL	meteorin like, glial cell differentiation regulator	1.26E-04	-4.24E-01
BIRC3	baculoviral IAP repeat containing 3	1.26E-04	4.39E-01
TUBG1	tubulin gamma 1	1.26E-04	1.16

RECK	reversion inducing cysteine rich protein with kazal motifs	1.27E-04	-7.20E-01
SEPSECS	Sep (O-phosphoserine) tRNA:Sec (selenocysteine) tRNA synthase	1.27E-04	5.66E-01
FGF2	fibroblast growth factor 2	1.27E-04	1.21
LIAS	lipoic acid synthetase	1.27E-04	-6.01E-01
STK17B	serine/threonine kinase 17b	1.28E-04	-9.33E-01
DENND6B	DENN domain containing 6B	1.28E-04	-9.26E-01
GLO1	glyoxalase I	1.29E-04	6.10E-01
DHX37	DEAH-box helicase 37	1.29E-04	6.92E-01
TKT	transketolase	1.29E-04	-8.01E-01
OAS3	2'-5'-oligoadenylate synthetase 3	1.29E-04	4.18E-01
ID1	inhibitor of DNA binding 1, HLH protein	1.29E-04	8.82E-01
DBP	D-box binding PAR bZIP transcription factor	1.29E-04	-1.41
TIMM23	translocase of inner mitochondrial membrane 23	1.30E-04	6.12E-01
STAP2	signal transducing adaptor family member 2	1.30E-04	5.01E-01
PSMB8	proteasome subunit beta 8	1.30E-04	6.01E-01
CCDC18	coiled-coil domain containing 18	1.30E-04	4.92E-01
RHEBL1	Ras homolog enriched in brain like 1	1.31E-04	4.16E-01

IBA57	IBA57 homolog, iron-sulfur cluster assembly	1.31E-04	-5.92E-01
TMX2	thioredoxin related transmembrane protein 2	1.32E-04	-5.98E-01
RPL13	ribosomal protein L13	1.32E-04	-9.08E-01
C3orf52	chromosome 3 open reading frame 52	1.32E-04	6.32E-01
AMY1A	amylase, alpha 1A (salivary)	1.32E-04	5.68E-01
LSG1	large 60S subunit nuclear export GTPase 1	1.33E-04	8.09E-01
CKM	creatine kinase, M-type	1.33E-04	4.91E-01
SOD2	superoxide dismutase 2, mitochondrial	1.33E-04	6.98E-01
CYB5B	cytochrome b5 type B	1.34E-04	6.68E-01
GABPB1	GA binding protein transcription factor beta subunit 1	1.34E-04	8.49E-01
TRMT1L	tRNA methyltransferase 1 like	1.34E-04	6.87E-01
RPF1	ribosome production factor 1 homolog	1.35E-04	1.11
PRMT5	protein arginine methyltransferase 5	1.35E-04	8.40E-01
RAB22A	RAB22A, member RAS oncogene family	1.35E-04	4.67E-01
ADGRL1	adhesion G protein-coupled receptor L1	1.35E-04	-9.26E-01
PARP9	poly(ADP-ribose) polymerase family member 9	1.35E-04	9.90E-01
DONSON	downstream neighbor of SON	1.35E-04	1.41

LYPD6B	LY6/PLAUR domain containing 6B	1.35E-04	-8.39E-01
IGDCC3	immunoglobulin superfamily DCC subclass member 3	1.35E-04	-6.83E-01
TPM2	tropomyosin 2 (beta)	1.36E-04	8.92E-01
PLAGL1	PLAG1 like zinc finger 1	1.36E-04	-1.01
PGLS	6-phosphogluconolactonase	1.36E-04	-1.13
TMEM109	transmembrane protein 109	1.36E-04	5.89E-01
CARD9	caspase recruitment domain family member 9	1.36E-04	-7.00E-01
SLC26A6	solute carrier family 26 member 6	1.36E-04	-1.33
PIF1	PIF1 5'-to-3' DNA helicase	1.36E-04	5.34E-01
GBE1	glucan (1,4-alpha-), branching enzyme 1	1.36E-04	-1.32
TUBA1A	tubulin alpha 1a	1.37E-04	1.49
SELV	selenoprotein V	1.37E-04	7.30E-01
MYOM1	myomesin 1	1.37E-04	-4.37E-01
KLRC2	killer cell lectin like receptor C2	1.37E-04	6.29E-01
LAPTM4B	lysosomal protein transmembrane 4 beta	1.37E-04	-6.21E-01
NDRG2	NDRG family member 2	1.37E-04	-5.77E-01
ZZEF1	zinc finger ZZ-type and EF-hand domain containing 1	1.37E-04	-5.13E-01

NSG1	neuron specific gene family member 1	1.38E-04	-6.71E-01
BEX3	brain expressed X-linked 3	1.38E-04	-9.74E-01
RABGGTB	Rab geranylgeranyltransferase beta subunit	1.38E-04	-5.94E-01
ARL2BP	ADP ribosylation factor like GTPase 2 binding protein	1.39E-04	5.39E-01
NOTO	notochord homeobox	1.39E-04	5.93E-01
EZR	ezrin	1.39E-04	9.97E-01
GSS	glutathione synthetase	1.39E-04	-6.97E-01
ELK4	ELK4, ETS transcription factor	1.39E-04	5.18E-01
PIP4K2A	phosphatidylinositol-5-phosphate 4-kinase type 2 alpha	1.39E-04	4.97E-01
TSEN15	tRNA splicing endonuclease subunit 15	1.39E-04	6.72E-01
PITX2	paired like homeodomain 2	1.40E-04	4.84E-01
TLE6	transducin like enhancer of split 6	1.40E-04	-1.02
TM7SF3	transmembrane 7 superfamily member 3	1.40E-04	-1.64
GRB7	growth factor receptor bound protein 7	1.40E-04	5.87E-01
CTTN	cortactin	1.40E-04	-6.31E-01
PTGES3	prostaglandin E synthase 3	1.41E-04	8.30E-01
KATNBL1	katanin regulatory subunit B1 like 1	1.41E-04	6.70E-01

HSPH1	heat shock protein family H (Hsp110) member 1	1.41E-04	8.85E -01
LSM8	LSM8 homolog, U6 small nuclear RNA associated	1.41E-04	9.81E -01
TUFT1	tuftelin 1	1.41E-04	9.47E -01
TTC32	tetratricopeptide repeat domain 32	1.41E-04	- 6.92E -01
FAM89A	family with sequence similarity 89 member A	1.41E-04	- 8.25E -01
FOXO1	forkhead box O1	1.42E-04	5.09E -01
FAM104A	family with sequence similarity 104 member A	1.42E-04	- 7.95E -01
CD68	CD68 molecule	1.42E-04	-1.63
RSPH3	radial spoke 3 homolog	1.42E-04	4.76E -01
C9orf85	chromosome 9 open reading frame 85	1.42E-04	-1.12
PSMA3	proteasome subunit alpha 3	1.43E-04	8.13E -01
SAP30	Sin3A associated protein 30	1.43E-04	8.08E -01
MAGEH1	MAGE family member H1	1.44E-04	- 5.98E -01
ACADVL	acyl-CoA dehydrogenase, very long chain	1.44E-04	- 5.96E -01
CCNG2	cyclin G2	1.44E-04	-1.33
TUBB3	tubulin beta 3 class III	1.44E-04	6.79E -01
AIDA	axin interactor, dorsalization associated	1.44E-04	7.36E -01

UBQLN1	ubiquilin 1	1.44E-04	9.37E-01
CDH7	cadherin 7	1.45E-04	5.83E-01
PSMA5	proteasome subunit alpha 5	1.45E-04	6.63E-01
ZNF350	zinc finger protein 350	1.45E-04	4.88E-01
SPCS3	signal peptidase complex subunit 3	1.45E-04	7.85E-01
GNL2	G protein nucleolar 2	1.45E-04	8.86E-01
PALM	paralemmin	1.45E-04	-1.12
ZNF610	zinc finger protein 610	1.46E-04	5.80E-01
ALG1L	ALG1, chitobiosyldiphosphodolichol beta-mannosyltransferase like	1.46E-04	-7.86E-01
HDAC8	histone deacetylase 8	1.47E-04	-6.34E-01
RCC1	regulator of chromosome condensation 1	1.47E-04	1.1
C14orf159	chromosome 14 open reading frame 159	1.47E-04	-7.11E-01
MKNK2	MAP kinase interacting serine/threonine kinase 2	1.47E-04	-1.03
S100A10	S100 calcium binding protein A10	1.47E-04	-1.22
UACA	uveal autoantigen with coiled-coil domains and ankyrin repeats	1.48E-04	-7.74E-01
MPST	mercaptopyruvate sulfurtransferase	1.48E-04	-5.34E-01
ZNF91	zinc finger protein 91	1.48E-04	2.61

SUN2	Sad1 and UNC84 domain containing 2	1.49E-04	-7.09E-01
HEYL	hes related family bHLH transcription factor with YRPW motif-like	1.49E-04	7.84E-01
CDK5RAP3	CDK5 regulatory subunit associated protein 3	1.49E-04	-8.78E-01
EEF2KMT	eukaryotic elongation factor 2 lysine methyltransferase	1.49E-04	9.68E-01
CRADD	CASP2 and RIPK1 domain containing adaptor with death domain	1.50E-04	-9.36E-01
BOLA3	bolA family member 3	1.50E-04	9.40E-01
NEDD1	neural precursor cell expressed, developmentally down-regulated 1	1.50E-04	1.42
CLDN15	claudin 15	1.50E-04	-1.36
PDIA4	protein disulfide isomerase family A member 4	1.51E-04	-8.47E-01
C2orf15	chromosome 2 open reading frame 15	1.51E-04	5.39E-01
B4GAT1	beta-1,4-glucuronyltransferase 1	1.51E-04	-7.62E-01
PLD3	phospholipase D family member 3	1.52E-04	-1.47
FH	fumarate hydratase	1.52E-04	7.29E-01
CHD6	chromodomain helicase DNA binding protein 6	1.52E-04	-6.20E-01
ADI1	acireductone dioxygenase 1	1.52E-04	4.83E-01
MRFAP1L1	Morf4 family associated protein 1 like 1	1.52E-04	-4.97E-01

UEVLD	UEV and lactate/malate dehyrogenase domains	1.52E-04	6.70E-01
BYSL	bystin like	1.53E-04	6.20E-01
ELOVL1	ELOVL fatty acid elongase 1	1.53E-04	-4.82E-01
SLC35B1	solute carrier family 35 member B1	1.53E-04	-6.78E-01
MTPN	myotrophin	1.53E-04	6.24E-01
PLTP	phospholipid transfer protein	1.53E-04	-2.38
VPS51	VPS51, GARP complex subunit	1.53E-04	-1.08
NOS3	nitric oxide synthase 3	1.54E-04	-1.44
DRAXIN	dorsal inhibitory axon guidance protein	1.54E-04	-5.55E-01
PTPRG	protein tyrosine phosphatase, receptor type G	1.54E-04	1.05
SERPINE1	serpin family E member 1	1.54E-04	4.88E-01
CRELD1	cysteine rich with EGF like domains 1	1.55E-04	-1.2
MANEAL	mannosidase endo-alpha like	1.56E-04	7.09E-01
RPL32	ribosomal protein L32	1.56E-04	-1.14
SLC6A9	solute carrier family 6 member 9	1.56E-04	-8.42E-01
GPRIN3	GPRIN family member 3	1.57E-04	-7.70E-01
TRAPPC13	trafficking protein particle complex 13	1.57E-04	-4.70E-01
NEMP1	nuclear envelope integral membrane protein 1	1.57E-04	1.31

DCUN1D5	defective in cullin neddylation 1 domain containing 5	1.57E-04	6.65E-01
C5orf34	chromosome 5 open reading frame 34	1.57E-04	9.75E-01
GLE1	GLE1, RNA export mediator	1.57E-04	9.60E-01
LONRF3	LON peptidase N-terminal domain and ring finger 3	1.58E-04	4.86E-01
CTSH	cathepsin H	1.58E-04	1
IRF2BP2	interferon regulatory factor 2 binding protein 2	1.58E-04	-1.79
NFKBIA	NFKB inhibitor alpha	1.59E-04	9.75E-01
SNTB2	syntrophin beta 2	1.59E-04	-8.14E-01
PSMG1	proteasome assembly chaperone 1	1.59E-04	1.11
MEX3A	mex-3 RNA binding family member A	1.59E-04	-6.90E-01
POLD4	DNA polymerase delta 4, accessory subunit	1.59E-04	-1.06
KRT18	keratin 18	1.60E-04	8.33E-01
PHF19	PHD finger protein 19	1.60E-04	9.95E-01
VDAC3	voltage dependent anion channel 3	1.60E-04	4.31E-01
DDX11	DEAD/H-box helicase 11	1.60E-04	9.29E-01
SP110	SP110 nuclear body protein	1.60E-04	6.92E-01
LRTOMT	leucine rich transmembrane and O-methyltransferase domain containing	1.60E-04	3.77E-01
CEP162	centrosomal protein 162	1.60E-04	3.70E-01

DNAJC12	DnaJ heat shock protein family (Hsp40) member C12	1.61E-04	-7.68E-01
KHK	ketohexokinase	1.61E-04	5.07E-01
RAI1	retinoic acid induced 1	1.61E-04	-7.36E-01
NUP155	nucleoporin 155	1.61E-04	1.36
ZMYND11	zinc finger MYND-type containing 11	1.61E-04	6.30E-01
ZNF160	zinc finger protein 160	1.61E-04	-6.62E-01
MYL5	myosin light chain 5	1.61E-04	-1.01
DSTN	destrin, actin depolymerizing factor	1.61E-04	7.96E-01
SYTL4	synaptotagmin like 4	1.61E-04	6.56E-01
PAIP2	poly(A) binding protein interacting protein 2	1.62E-04	-4.69E-01
GALNT4	polypeptide N-acetylgalactosaminyltransferase 4	1.62E-04	-1.16
ZNF264	zinc finger protein 264	1.62E-04	-5.13E-01
CTSO	cathepsin O	1.62E-04	8.35E-01
PCGF6	polycomb group ring finger 6	1.62E-04	1.1
LOC146880	Rho GTPase activating protein 27 pseudogene	1.62E-04	6.16E-01
MAFG	MAF bZIP transcription factor G	1.62E-04	-5.34E-01

SPIRE2	spire type actin nucleation factor 2	1.63E-04	-5.88E-01
CHAC2	ChaC cation transport regulator homolog 2	1.63E-04	8.03E-01
ATG4C	autophagy related 4C cysteine peptidase	1.63E-04	8.00E-01
IKZF5	IKAROS family zinc finger 5	1.64E-04	4.19E-01
GPD2	glycerol-3-phosphate dehydrogenase 2	1.64E-04	1.13
NRG1	neuregulin 1	1.64E-04	5.24E-01
RMI1	RecQ mediated genome instability 1	1.64E-04	1.43
NUP37	nucleoporin 37	1.64E-04	8.26E-01
PI4KAP1	phosphatidylinositol 4-kinase alpha pseudogene 1	1.64E-04	-5.96E-01
L3MBTL1	l(3)mbt-like 1 (Drosophila)	1.65E-04	-6.35E-01
MEST	mesoderm specific transcript	1.65E-04	-1.2
DUSP11	dual specificity phosphatase 11	1.65E-04	6.99E-01
PAN2	PAN2 poly(A) specific ribonuclease subunit	1.65E-04	-4.84E-01
STRN	striatin	1.65E-04	7.71E-01
HIST3H2A	histone cluster 3, H2a	1.65E-04	-1.93
SNX17	sorting nexin 17	1.66E-04	-5.89E-01
SPP1	secreted phosphoprotein 1	1.66E-04	-5.72E-01

OR7E156P	olfactory receptor family 7 subfamily E member 156 pseudogene	1.66E-04	-9.15E-01
NLGN2	neuroligin 2	1.66E-04	-9.08E-01
FOXO3	forkhead box O3	1.66E-04	-9.17E-01
MALSU1	mitochondrial assembly of ribosomal large subunit 1	1.66E-04	6.35E-01
FAM43A	family with sequence similarity 43 member A	1.67E-04	-1.05
CREG1	cellular repressor of E1A stimulated genes 1	1.67E-04	-6.30E-01
BMPR2	bone morphogenetic protein receptor type 2	1.67E-04	-9.02E-01
CCDC50	coiled-coil domain containing 50	1.67E-04	4.63E-01
LPCAT1	lysophosphatidylcholine acyltransferase 1	1.68E-04	5.40E-01
RSBN1	round spermatid basic protein 1	1.68E-04	-4.42E-01
IFT52	intraflagellar transport 52	1.68E-04	8.92E-01
SKIL	SKI-like proto-oncogene	1.68E-04	5.63E-01
FBXW7	F-box and WD repeat domain containing 7	1.68E-04	-6.38E-01
MAGED2	MAGE family member D2	1.69E-04	-1.45
CYTH1	cytohesin 1	1.69E-04	-8.52E-01

ZNF518B	zinc finger protein 518B	1.69E-04	6.66E-01
PBX4	PBX homeobox 4	1.70E-04	4.54E-01
VASH2	vasohibin 2	1.70E-04	6.46E-01
CORO1B	coronin 1B	1.70E-04	-1.14
FAM173B	family with sequence similarity 173 member B	1.70E-04	4.08E-01
SEH1L	SEH1 like nucleoporin	1.70E-04	8.42E-01
C15orf57	chromosome 15 open reading frame 57	1.70E-04	-7.13E-01
OSGIN2	oxidative stress induced growth inhibitor family member 2	1.70E-04	7.31E-01
NINJ1	ninjurin 1	1.70E-04	-1.21
TKFC	triokinase and FMN cyclase	1.71E-04	-5.67E-01
GPR63	G protein-coupled receptor 63	1.71E-04	5.47E-01
RHBDF2	rhomboid 5 homolog 2	1.72E-04	-8.14E-01
GAD1	glutamate decarboxylase 1	1.72E-04	-4.85E-01
PRMT3	protein arginine methyltransferase 3	1.72E-04	4.32E-01
RBMS1	RNA binding motif single stranded interacting protein 1	1.73E-04	-5.87E-01
ANKLE1	ankyrin repeat and LEM domain containing 1	1.73E-04	-5.29E-01

GSPT1	G1 to S phase transition 1	1.73E-04	6.22E-01
MRPL19	mitochondrial ribosomal protein L19	1.73E-04	7.13E-01
TRIM3	tripartite motif containing 3	1.73E-04	-1.18
TBC1D9	TBC1 domain family member 9	1.73E-04	-7.34E-01
CSE1L	chromosome segregation 1 like	1.74E-04	1.23
STRA13	stimulated by retinoic acid 13	1.74E-04	9.44E-01
ARL6IP1	ADP ribosylation factor like GTPase 6 interacting protein 1	1.74E-04	1.31
TRIM36	tripartite motif containing 36	1.74E-04	4.79E-01
CARMIL3	capping protein regulator and myosin 1 linker 3	1.74E-04	-4.97E-01
MAP7	microtubule associated protein 7	1.75E-04	4.47E-01
FAM13B	family with sequence similarity 13 member B	1.75E-04	-6.27E-01
LINGO2	leucine rich repeat and Ig domain containing 2	1.75E-04	-7.45E-01
ERLIN1	ER lipid raft associated 1	1.76E-04	5.47E-01
TBC1D15	TBC1 domain family member 15	1.76E-04	6.41E-01
CDKN1A	cyclin dependent kinase inhibitor 1A	1.77E-04	-6.76E-01
RHOA	ras homolog family member A	1.77E-04	5.63E-01

METTL23	methyltransferase like 23	1.78E-04	-9.98E-01
C1QL4	complement C1q like 4	1.78E-04	-6.29E-01
ZNF615	zinc finger protein 615	1.78E-04	4.53E-01
C5orf30	chromosome 5 open reading frame 30	1.78E-04	5.45E-01
LARP6	La ribonucleoprotein domain family member 6	1.78E-04	-7.82E-01
CDCA7	cell division cycle associated 7	1.79E-04	5.00E-01
HLA-B	major histocompatibility complex, class I, B	1.79E-04	5.19E-01
HSD17B14	hydroxysteroid 17-beta dehydrogenase 14	1.79E-04	-1.39
SLC25A4	solute carrier family 25 member 4	1.79E-04	4.16E-01
GAMT	guanidinoacetate N-methyltransferase	1.79E-04	-1.59
WNK1	WNK lysine deficient protein kinase 1	1.80E-04	9.00E-01
MXRA7	matrix remodeling associated 7	1.80E-04	-5.92E-01
METTL18	methyltransferase like 18	1.80E-04	1.12
PPP2R2C	protein phosphatase 2 regulatory subunit Bgamma	1.80E-04	9.16E-01
C11orf49	chromosome 11 open reading frame 49	1.81E-04	-7.32E-01
CTF1	cardiotrophin 1	1.81E-04	-7.87E-01

CHAMP1	chromosome alignment maintaining phosphoprotein 1	1.81E-04	-8.89E-01
SNAP47	synaptosome associated protein 47	1.82E-04	-8.07E-01
RIOK1	RIO kinase 1	1.82E-04	6.51E-01
NEBL	nebulette	1.82E-04	6.12E-01
CPQ	carboxypeptidase Q	1.82E-04	5.99E-01
NAXD	NAD(P)HX dehydratase	1.83E-04	-7.70E-01
GPR162	G protein-coupled receptor 162	1.83E-04	-1.11
MRPL45	mitochondrial ribosomal protein L45	1.83E-04	-6.65E-01
NR2C2	nuclear receptor subfamily 2 group C member 2	1.83E-04	4.86E-01
LCLAT1	lysocardiolipin acyltransferase 1	1.83E-04	7.36E-01
POU1F1	POU class 1 homeobox 1	1.83E-04	5.52E-01
DNAJA1	DnaJ heat shock protein family (Hsp40) member A1	1.84E-04	3.82E-01
PPP2R2C	protein phosphatase 2 regulatory subunit Bgamma	1.84E-04	9.70E-01
SLC27A2	solute carrier family 27 member 2	1.84E-04	4.92E-01
DZIP1	DAZ interacting zinc finger protein 1	1.84E-04	-8.37E-01
SRSF5	serine and arginine rich splicing factor 5	1.84E-04	-6.36E-01

SNAP47	synaptosome associated protein 47	1.84E-04	-5.90E-01
SUN3	Sad1 and UNC84 domain containing 3	1.84E-04	-1.12
TATDN2	TatD DNase domain containing 2	1.84E-04	5.39E-01
TPT1	tumor protein, translationally-controlled 1	1.85E-04	-1.2
STRADA	STE20-related kinase adaptor alpha	1.85E-04	-1.15
YBX1	Y-box binding protein 1	1.85E-04	6.69E-01
DUSP22	dual specificity phosphatase 22	1.85E-04	-5.95E-01
SLC4A5	solute carrier family 4 member 5	1.85E-04	-9.51E-01
DYNC1LI1	dynein cytoplasmic 1 light intermediate chain 1	1.85E-04	1
POPDC3	popeye domain containing 3	1.85E-04	-8.90E-01
FAM220A	family with sequence similarity 220 member A	1.86E-04	9.44E-01
POLH	DNA polymerase eta	1.86E-04	-1.16
FNDC1	fibronectin type III domain containing 1	1.86E-04	-5.74E-01
WEE1	WEE1 G2 checkpoint kinase	1.87E-04	8.09E-01
CBX4	chromobox 4	1.87E-04	-1.03
SECISBP2L	SECIS binding protein 2 like	1.87E-04	-6.49E-01
GNPTG	N-acetylglucosamine-1-phosphate transferase gamma subunit	1.87E-04	-1.09

PALM3	paralemmin 3	1.88E-04	-<br>4.15E<br>-01
MLLT6	MLLT6, PHD finger domain containing	1.88E-04	7.21E<br>-01
AAMP	angio associated migratory cell protein	1.88E-04	5.78E<br>-01
NEK6	NIMA related kinase 6	1.89E-04	-<br>5.34E<br>-01
NAT1	N-acetyltransferase 1	1.89E-04	4.46E<br>-01
DTNBP1	dystrobrevin binding protein 1	1.89E-04	6.17E<br>-01
ENAH	enabled homolog (Drosophila)	1.89E-04	5.40E<br>-01
SLC45A3	solute carrier family 45 member 3	1.90E-04	6.12E<br>-01
SNX1	sorting nexin 1	1.90E-04	-<br>9.12E<br>-01
DFNB59	deafness, autosomal recessive 59	1.90E-04	-<br>5.95E<br>-01
CH25H	cholesterol 25-hydroxylase	1.91E-04	-<br>4.72E<br>-01
BRIX1	BRX1, biogenesis of ribosomes	1.91E-04	9.79E<br>-01
STAG2	stromal antigen 2	1.91E-04	4.53E<br>-01
C17orf82	chromosome 17 open reading frame 82	1.91E-04	-1.05
STAG1	stromal antigen 1	1.91E-04	7.94E<br>-01
NACAP1	nascent polypeptide associated complex alpha subunit pseudogene 1	1.92E-04	-<br>8.49E<br>-01

CCT3	chaperonin containing TCP1 subunit 3	1.92E-04	-5.23E-01
OST4	oligosaccharyltransferase complex subunit 4, non-catalytic	1.92E-04	-9.42E-01
SAT2	spermidine/spermine N1-acetyltransferase family member 2	1.92E-04	-7.35E-01
NUF2	NUF2, NDC80 kinetochore complex component	1.92E-04	5.09E-01
RBBP9	RB binding protein 9, serine hydrolase	1.93E-04	6.06E-01
ITGB1BP1	integrin subunit beta 1 binding protein 1	1.93E-04	5.90E-01
NUFIP1	NUFIP1, FMR1 interacting protein 1	1.93E-04	5.21E-01
ATL1	atlastin GTPase 1	1.94E-04	5.84E-01
HGF	hepatocyte growth factor	1.94E-04	-1.44
IRF3	interferon regulatory factor 3	1.94E-04	5.76E-01
NMD3	NMD3 ribosome export adaptor	1.94E-04	7.32E-01
GSTT1	glutathione S-transferase theta 1	1.95E-04	-1.13
MTHFD2	methylenetetrahydrofolate dehydrogenase (NADP+ dependent) 2, methenyltetrahydrofolate cyclohydrolase	1.95E-04	5.25E-01
DNAL1	dynein axonemal light chain 1	1.95E-04	1.04
EFHD2	EF-hand domain family member D2	1.95E-04	6.53E-01
SGK3	serum/glucocorticoid regulated kinase family member 3	1.96E-04	5.86E-01
GADD45A	growth arrest and DNA damage inducible alpha	1.96E-04	-1.22

PRDX1	peroxiredoxin 1	1.97E-04	4.63E-01
SCYL2	SCY1 like pseudokinase 2	1.97E-04	1.32
SOWAHC	sosondowah ankyrin repeat domain family member C	1.97E-04	-6.87E-01
ISG20	interferon stimulated exonuclease gene 20	1.98E-04	7.89E-01
DTX3	deltex E3 ubiquitin ligase 3	1.98E-04	-7.40E-01
MZF1	myeloid zinc finger 1	1.98E-04	-1.1
NEDD1	neural precursor cell expressed, developmentally down-regulated 1	1.98E-04	1.3
EIF2B3	eukaryotic translation initiation factor 2B subunit gamma	1.98E-04	9.82E-01
MAP1LC3B	microtubule associated protein 1 light chain 3 beta	1.99E-04	-7.39E-01
TMEM182	transmembrane protein 182	2.00E-04	4.08E-01
TANK	TRAF family member associated NFKB activator	2.00E-04	-6.76E-01
SNRNP70	small nuclear ribonucleoprotein U1 subunit 70	2.00E-04	5.01E-01
CWF19L1	CWF19-like 1, cell cycle control (S. pombe)	2.00E-04	5.35E-01
RANBP2	RAN binding protein 2	2.00E-04	1.19
GTF2E2	general transcription factor IIE subunit 2	2.01E-04	5.68E-01
ELL3	elongation factor for RNA polymerase II 3	2.01E-04	-5.24E-01
FAM89A	family with sequence similarity 89 member A	2.01E-04	-9.71E-01

CCDC84	coiled-coil domain containing 84	2.01E-04	7.40E-01
YPEL2	yippee like 2	2.01E-04	-1.07
CCNH	cyclin H	2.01E-04	7.67E-01
CDC16	cell division cycle 16	2.01E-04	-6.52E-01
VTA1	vesicle trafficking 1	2.01E-04	7.77E-01
UBE2C	ubiquitin conjugating enzyme E2 C	2.02E-04	6.60E-01
TCEAL4	transcription elongation factor A like 4	2.02E-04	-4.97E-01
RBM17	RNA binding motif protein 17	2.03E-04	-5.25E-01
PDZD4	PDZ domain containing 4	2.03E-04	-5.97E-01
FHL2	four and a half LIM domains 2	2.03E-04	8.84E-01
CDR2L	cerebellar degeneration related protein 2 like	2.03E-04	-9.91E-01
WDR27	WD repeat domain 27	2.04E-04	-6.19E-01
PTDSS2	phosphatidylserine synthase 2	2.04E-04	-6.13E-01
SACS	sacsin molecular chaperone	2.04E-04	1.33
SLC20A1	solute carrier family 20 member 1	2.04E-04	1.25
RRP7A	ribosomal RNA processing 7 homolog A	2.06E-04	9.41E-01
MATK	megakaryocyte-associated tyrosine kinase	2.06E-04	-1.99

E2F8	E2F transcription factor 8	2.06E-04	5.76E -01
TOMM20	translocase of outer mitochondrial membrane 20	2.06E-04	-1.32
GRAMD2	GRAM domain containing 2	2.06E-04	3.86E -01
RNF34	ring finger protein 34	2.06E-04	6.04E -01
ATRIP	ATR interacting protein	2.06E-04	7.86E -01
HIST1H3F	histone cluster 1, H3f	2.06E-04	- 7.00E -01
ZBBX	zinc finger B-box domain containing	2.07E-04	3.61E -01
TOP1P2	topoisomerase (DNA) I pseudogene 2	2.07E-04	6.11E -01
SRP9	signal recognition particle 9	2.07E-04	5.42E -01
DNAJB5	DnaJ heat shock protein family (Hsp40) member B5	2.07E-04	- 5.89E -01
GLB1	galactosidase beta 1	2.07E-04	- 4.48E -01
PARVB	parvin beta	2.08E-04	8.06E -01
APEX2	apurinic/apyrimidinic endodeoxyribonuclease 2	2.08E-04	7.50E -01
NCOR2	nuclear receptor corepressor 2	2.08E-04	-1.15
SLC25A46	solute carrier family 25 member 46	2.09E-04	8.54E -01
ELK4	ELK4, ETS transcription factor	2.09E-04	5.57E -01
SRSF10	serine and arginine rich splicing factor 10	2.09E-04	6.44E -01

DCXR	dicarbonyl and L-xylulose reductase	2.10E-04	-7.98E-01
DARS2	aspartyl-tRNA synthetase 2, mitochondrial	2.10E-04	5.49E-01
IKBIP	IKBKB interacting protein	2.10E-04	-1.25
SRSF8	serine and arginine rich splicing factor 8	2.10E-04	-5.49E-01
AEN	apoptosis enhancing nuclease	2.11E-04	-9.61E-01
CLDN12	claudin 12	2.12E-04	-7.04E-01
FUT4	fucosyltransferase 4	2.12E-04	4.69E-01
TSHZ2	teashirt zinc finger homeobox 2	2.13E-04	-1.01
MX1	MX dynamin like GTPase 1	2.13E-04	4.11E-01
RAB8A	RAB8A, member RAS oncogene family	2.13E-04	4.18E-01
LONP2	lon peptidase 2, peroxisomal	2.13E-04	-6.14E-01
ZDHHC23	zinc finger DHHC-type containing 23	2.14E-04	8.06E-01
ATP2B1	ATPase plasma membrane Ca2+ transporting 1	2.14E-04	6.11E-01
NET1	neuroepithelial cell transforming 1	2.15E-04	1.01
RNF165	ring finger protein 165	2.16E-04	-5.42E-01
GPN2	GPN-loop GTPase 2	2.16E-04	-8.73E-01

PRR13	proline rich 13	2.16E-04	7.05E -01
HCFC2	host cell factor C2	2.16E-04	6.80E -01
IK	IK cytokine, down-regulator of HLA II	2.17E-04	4.26E -01
C11orf63	chromosome 11 open reading frame 63	2.17E-04	4.48E -01
C4orf46	chromosome 4 open reading frame 46	2.18E-04	1.34
ANXA4	annexin A4	2.18E-04	-1.38
SEC22C	SEC22 homolog C, vesicle trafficking protein	2.19E-04	6.21E -01
NOL6	nucleolar protein 6	2.19E-04	9.72E -01
PLEKHA3	pleckstrin homology domain containing A3	2.19E-04	9.09E -01
ANKRD6	ankyrin repeat domain 6	2.19E-04	7.14E -01
RERG	RAS like estrogen regulated growth inhibitor	2.20E-04	- 5.04E -01
STAMBP	STAM binding protein	2.20E-04	5.51E -01
DNAH14	dynein axonemal heavy chain 14	2.20E-04	- 4.93E -01
PROC	protein C, inactivator of coagulation factors Va and VIIIa	2.21E-04	4.81E -01
TBC1D31	TBC1 domain family member 31	2.21E-04	7.70E -01
TPM4	tropomyosin 4	2.21E-04	7.91E -01
TUSC1	tumor suppressor candidate 1	2.21E-04	7.58E -01

TRIM66	tripartite motif containing 66	2.22E-04	-5.42E-01
MXD1	MAX dimerization protein 1	2.22E-04	-1.09
SOD2	superoxide dismutase 2, mitochondrial	2.22E-04	1.16
OXLD1	oxidoreductase like domain containing 1	2.23E-04	-1.25
NIF3L1	NGG1 interacting factor 3 like 1	2.23E-04	4.51E-01
FAM13A	family with sequence similarity 13 member A	2.23E-04	-1.43
HNRNPA2B1	heterogeneous nuclear ribonucleoprotein A2/B1	2.23E-04	5.45E-01
NDUFB5	NADH:ubiquinone oxidoreductase subunit B5	2.24E-04	-6.32E-01
SPA17	sperm autoantigenic protein 17	2.24E-04	1.17
HSD3B7	hydroxy-delta-5-steroid dehydrogenase, 3 beta- and steroid delta-isomerase 7	2.24E-04	5.99E-01
MED7	mediator complex subunit 7	2.24E-04	6.60E-01
OAF	out at first homolog	2.24E-04	-7.91E-01
CKS1B	CDC28 protein kinase regulatory subunit 1B	2.25E-04	1.79
USP42	ubiquitin specific peptidase 42	2.25E-04	-6.12E-01
TTPAL	alpha tocopherol transfer protein like	2.26E-04	5.77E-01
KLF6	Kruppel like factor 6	2.26E-04	9.66E-01
DLX5	distal-less homeobox 5	2.27E-04	-1.29
C3orf38	chromosome 3 open reading frame 38	2.27E-04	5.40E-01

UBE2Q1	ubiquitin conjugating enzyme E2 Q1	2.27E-04	-7.23E-01
STRBP	spermatid perinuclear RNA binding protein	2.27E-04	7.80E-01
SMAD3	SMAD family member 3	2.28E-04	-1.15
PRKAA2	protein kinase AMP-activated catalytic subunit alpha 2	2.28E-04	4.37E-01
FZD9	frizzled class receptor 9	2.29E-04	7.08E-01
KIAA2013	KIAA2013	2.29E-04	-7.90E-01
CCDC130	coiled-coil domain containing 130	2.29E-04	-8.20E-01
PIGF	phosphatidylinositol glycan anchor biosynthesis class F	2.29E-04	-7.11E-01
TSNAXIP1	translin associated factor X interacting protein 1	2.30E-04	6.20E-01
ARRDC4	arrestin domain containing 4	2.31E-04	5.86E-01
FAHD1	fumarylacetoacetate hydrolase domain containing 1	2.31E-04	-6.29E-01
SFXN4	sideroflexin 4	2.32E-04	-8.88E-01
CEP83	centrosomal protein 83	2.32E-04	9.39E-01
AFMID	arylformamidase	2.32E-04	-1.32
EFNA4	ephrin A4	2.32E-04	-1.02
LINC01089	long intergenic non-protein coding RNA 1089	2.33E-04	-4.68E-01

PARP10	poly(ADP-ribose) polymerase family member 10	2.33E-04	5.77E-01
RRP12	ribosomal RNA processing 12 homolog	2.35E-04	7.48E-01
ARL4A	ADP ribosylation factor like GTPase 4A	2.35E-04	6.25E-01
RINL	Ras and Rab interactor like	2.36E-04	-6.35E-01
NOA1	nitric oxide associated 1	2.36E-04	-3.74E-01
PLOD1	procollagen-lysine,2-oxoglutarate 5-dioxygenase 1	2.37E-04	-1.23
VPS29	VPS29, retromer complex component	2.37E-04	8.11E-01
RFC2	replication factor C subunit 2	2.37E-04	5.94E-01
DSC2	desmocollin 2	2.37E-04	3.48E-01
ARHGAP19	Rho GTPase activating protein 19	2.37E-04	1.16
DPH7	diphthamide biosynthesis 7	2.38E-04	5.26E-01
ETV6	ETS variant 6	2.38E-04	-6.18E-01
GTF3C2	general transcription factor IIIC subunit 2	2.38E-04	-8.26E-01
AZIN1	antizyme inhibitor 1	2.38E-04	9.30E-01
ZBTB26	zinc finger and BTB domain containing 26	2.39E-04	5.56E-01
CD79B	CD79b molecule	2.39E-04	-1.05
CENPT	centromere protein T	2.41E-04	5.84E-01

B4GALNT4	beta-1,4-N-acetyl-galactosaminyltransferase 4	2.41E-04	-7.54E-01
HP1BP3	heterochromatin protein 1 binding protein 3	2.41E-04	4.17E-01
MNX1	motor neuron and pancreas homeobox 1	2.42E-04	7.15E-01
ZNF423	zinc finger protein 423	2.42E-04	-7.55E-01
ZYX	zyxin	2.42E-04	8.33E-01
CLIP1	CAP-Gly domain containing linker protein 1	2.42E-04	8.59E-01
C10orf2	chromosome 10 open reading frame 2	2.43E-04	5.94E-01
NDFIP2	Nedd4 family interacting protein 2	2.43E-04	-7.30E-01
SNORD16	small nucleolar RNA, C/D box 16	2.43E-04	1.07
GRAP	GRB2-related adaptor protein	2.44E-04	3.97E-01
RAD51B	RAD51 paralog B	2.45E-04	6.20E-01
NAGLU	N-acetyl-alpha-glucosaminidase	2.45E-04	-5.96E-01
Sep-03	septin 3	2.46E-04	4.61E-01
ANKRD13C	ankyrin repeat domain 13C	2.46E-04	8.10E-01
ZHX3	zinc fingers and homeoboxes 3	2.46E-04	-6.44E-01
FBXO17	F-box protein 17	2.47E-04	-5.18E-01

CCDC66	coiled-coil domain containing 66	2.47E-04	4.95E<br>-01
CDK20	cyclin dependent kinase 20	2.47E-04	7.78E<br>-01
MFSD11	major facilitator superfamily domain containing 11	2.47E-04	-1.05
TNFRSF10B	TNF receptor superfamily member 10b	2.48E-04	-1.28
FAHD1	fumarylacetoacetate hydrolase domain containing 1	2.48E-04	-<br>8.86E<br>-01
SETMAR	SET domain and mariner transposase fusion gene	2.48E-04	5.33E<br>-01
POP4	POP4 homolog, ribonuclease P/MRP subunit	2.48E-04	6.62E<br>-01
RIOK3	RIO kinase 3	2.49E-04	-<br>4.24E<br>-01
ZFP92	ZFP92 zinc finger protein	2.49E-04	-<br>4.92E<br>-01
C4orf32	chromosome 4 open reading frame 32	2.49E-04	4.93E<br>-01
ACYP1	acylphosphatase 1	2.49E-04	1.21
GSE1	Gse1 coiled-coil protein	2.49E-04	-<br>6.86E<br>-01
CASP3	caspase 3	2.49E-04	6.50E<br>-01
GABPB1-AS1	GABPB1 antisense RNA 1	2.49E-04	-<br>6.43E<br>-01
ZNF165	zinc finger protein 165	2.49E-04	5.34E<br>-01
NBN	nibrin	2.51E-04	6.09E<br>-01
MRPL33	mitochondrial ribosomal protein L33	2.51E-04	-1.1

UAP1L1	UDP-N-acetylglucosamine pyrophosphorylase 1 like 1	2.51E-04	-1.03
ADAM19	ADAM metallopeptidase domain 19	2.52E-04	-6.19E-01
SPRED1	sprouty related EVH1 domain containing 1	2.52E-04	-6.23E-01
DBT	dihydrolipoamide branched chain transacylase E2	2.52E-04	-8.72E-01
CCDC8	coiled-coil domain containing 8	2.53E-04	-7.19E-01
FAM149A	family with sequence similarity 149 member A	2.54E-04	-6.13E-01
TGDS	TDP-glucose 4,6-dehydratase	2.55E-04	-1.03
RALGPS2	Ral GEF with PH domain and SH3 binding motif 2	2.55E-04	4.32E-01
PASK	PAS domain containing serine/threonine kinase	2.56E-04	5.93E-01
DYX1C1	dyslexia susceptibility 1 candidate 1	2.56E-04	7.33E-01
TNS3	tensin 3	2.56E-04	-6.14E-01
SPATA5L1	spermatogenesis associated 5 like 1	2.56E-04	8.21E-01
DDR2	discoidin domain receptor tyrosine kinase 2	2.57E-04	-1.75
SBK1	SH3 domain binding kinase 1	2.57E-04	-1.18
TRABD2A	TraB domain containing 2A	2.57E-04	3.76E-01
RPL4	ribosomal protein L4	2.58E-04	-4.74E-01

TMEM98	transmembrane protein 98	2.58E-04	3.45E-01
MCOLN2	mucolipin 2	2.61E-04	-4.85E-01
TEAD2	TEA domain transcription factor 2	2.61E-04	-6.78E-01
QARS	glutaminyl-tRNA synthetase	2.61E-04	-8.27E-01
CCDC74A	coiled-coil domain containing 74A	2.62E-04	5.64E-01
VPS33A	VPS33A, CORVET/HOPS core subunit	2.62E-04	5.83E-01
TPRKB	TP53RK binding protein	2.63E-04	1.46
LZTS2	leucine zipper tumor suppressor 2	2.63E-04	-5.46E-01
PRPS1	phosphoribosyl pyrophosphate synthetase 1	2.63E-04	6.31E-01
NAPG	NSF attachment protein gamma	2.64E-04	5.66E-01
NAGK	N-acetylglucosamine kinase	2.64E-04	9.69E-01
ATG10	autophagy related 10	2.64E-04	-6.80E-01
TRIP11	thyroid hormone receptor interactor 11	2.64E-04	8.00E-01
RBM47	RNA binding motif protein 47	2.65E-04	5.30E-01
G3BP1	G3BP stress granule assembly factor 1	2.65E-04	1.13
ITPR3	inositol 1,4,5-trisphosphate receptor type 3	2.65E-04	9.53E-01
ZSCAN29	zinc finger and SCAN domain containing 29	2.65E-04	3.52E-01

NDUFAB1	NADH:ubiquinone oxidoreductase subunit AB1	2.66E-04	3.47E-01
HNRNPM	heterogeneous nuclear ribonucleoprotein M	2.66E-04	7.94E-01
ST13P4	suppression of tumorigenicity 13 (colon carcinoma) (Hsp70 interacting protein) pseudogene 4	2.67E-04	-6.21E-01
KCNH2	potassium voltage-gated channel subfamily H member 2	2.67E-04	-4.71E-01
COPS3	COP9 signalosome subunit 3	2.67E-04	6.46E-01
DYNLL1	dynein light chain LC8-type 1	2.68E-04	4.66E-01
ZCCHC24	zinc finger CCHC-type containing 24	2.68E-04	-6.17E-01
DLG3	discs large MAGUK scaffold protein 3	2.68E-04	4.21E-01
LAMA2	laminin subunit alpha 2	2.69E-04	5.37E-01
YAE1D1	Yae1 domain containing 1	2.69E-04	7.81E-01
NAGPA	N-acetylglucosamine-1-phosphodiester alpha-N-acetylglucosaminidase	2.69E-04	6.06E-01
MKLN1	muskelin 1	2.71E-04	-7.96E-01
STAG2	stromal antigen 2	2.71E-04	1.45
NEIL3	nei like DNA glycosylase 3	2.71E-04	3.55E-01
SYTL2	synaptotagmin like 2	2.71E-04	1.4
P3H1	prolyl 3-hydroxylase 1	2.72E-04	-5.64E-01

TARBP1	TAR (HIV-1) RNA binding protein 1	2.72E-04	-8.93E-01
FGF11	fibroblast growth factor 11	2.72E-04	-6.96E-01
HADH	hydroxyacyl-CoA dehydrogenase	2.73E-04	5.08E-01
TRAM1L1	translocation associated membrane protein 1-like 1	2.74E-04	-8.80E-01
ID1	inhibitor of DNA binding 1, HLH protein	2.74E-04	1.2
FBXL15	F-box and leucine rich repeat protein 15	2.75E-04	-8.25E-01
LOC407835	mitogen-activated protein kinase kinase 2 pseudogene	2.75E-04	-8.75E-01
NR2C1	nuclear receptor subfamily 2 group C member 1	2.76E-04	4.80E-01
MRPL35	mitochondrial ribosomal protein L35	2.76E-04	9.53E-01
ZDHHC6	zinc finger DHHC-type containing 6	2.76E-04	6.65E-01
GAR1	GAR1 ribonucleoprotein	2.77E-04	9.96E-01
ZFP37	ZFP37 zinc finger protein	2.78E-04	7.22E-01
NCOA7	nuclear receptor coactivator 7	2.78E-04	1.08
ITM2C	integral membrane protein 2C	2.78E-04	-7.77E-01
CHST4	carbohydrate sulfotransferase 4	2.78E-04	6.21E-01
SLC25A12	solute carrier family 25 member 12	2.78E-04	-1.3
MYO1D	myosin ID	2.79E-04	4.51E-01

COLEC11	collectin subfamily member 11	2.79E-04	-1.16
RFNG	RFNG O-fucosylpeptide 3-beta-N-acetylglucosaminyltransferase	2.80E-04	-7.61E-01
ARL5B	ADP ribosylation factor like GTPase 5B	2.81E-04	6.57E-01
OIP5-AS1	OIP5 antisense RNA 1	2.81E-04	8.88E-01
STRADB	STE20-related kinase adaptor beta	2.82E-04	4.01E-01
ST6GALNAC3	ST6 N-acetylgalactosaminide alpha-2,6-sialyltransferase 3	2.82E-04	-4.95E-01
CALCB	calcitonin related polypeptide beta	2.83E-04	6.21E-01
PHACTR2	phosphatase and actin regulator 2	2.83E-04	-1.26
RECQL4	RecQ like helicase 4	2.83E-04	4.09E-01
UFSP2	UFM1 specific peptidase 2	2.83E-04	5.42E-01
PSMD14	proteasome 26S subunit, non-ATPase 14	2.84E-04	7.62E-01
CCT6P1	chaperonin containing TCP1 subunit 6 pseudogene 1	2.84E-04	9.92E-01
SALL2	spalt like transcription factor 2	2.84E-04	8.56E-01
SPAG1	sperm associated antigen 1	2.84E-04	3.82E-01
EPB41L4A-AS1	EPB41L4A antisense RNA 1	2.85E-04	-8.19E-01
RELL1	RELT like 1	2.86E-04	6.96E-01
NIM1K	NIM1 serine/threonine protein kinase	2.86E-04	-4.34E-01

NUP50	nucleoporin 50	2.87E-04	8.63E -01
CMC2	C-X9-C motif containing 2	2.88E-04	8.60E -01
CAPRIN1	cell cycle associated protein 1	2.88E-04	6.22E -01
DNTTIP2	deoxynucleotidyltransferase terminal interacting protein 2	2.88E-04	1.1
DNMT1	DNA (cytosine-5-)-methyltransferase 1	2.88E-04	9.53E -01
TMOD3	tropomodulin 3	2.88E-04	4.39E -01
CHCHD2	coiled-coil-helix-coiled-coil-helix domain containing 2	2.89E-04	3.41E -01
IKBIP	IKBKB interacting protein	2.89E-04	-1.14
RIMS3	regulating synaptic membrane exocytosis 3	2.89E-04	- 4.85E -01
CPSF3	cleavage and polyadenylation specific factor 3	2.90E-04	6.86E -01
ZNF451	zinc finger protein 451	2.90E-04	-1.07
GALNT16	polypeptide N-acetylgalactosaminyltransferase 16	2.90E-04	- 3.84E -01
TIMM23	translocase of inner mitochondrial membrane 23	2.90E-04	6.92E -01
EGR1	early growth response 1	2.90E-04	9.12E -01
MAP4K5	mitogen-activated protein kinase kinase kinase kinase 5	2.90E-04	- 6.80E -01
DDX56	DEAD-box helicase 56	2.91E-04	3.86E -01
C9orf24	chromosome 9 open reading frame 24	2.91E-04	3.91E -01
BUB3	BUB3, mitotic checkpoint protein	2.91E-04	1.15

ZMYM1	zinc finger MYM-type containing 1	2.91E-04	4.67E-01
KCTD9	potassium channel tetramerization domain containing 9	2.92E-04	6.31E-01
RABAC1	Rab acceptor 1	2.93E-04	-1.3
DDX59	DEAD-box helicase 59	2.93E-04	-3.90E-01
SPATA4	spermatogenesis associated 4	2.94E-04	5.12E-01
PPP3CA	protein phosphatase 3 catalytic subunit alpha	2.94E-04	7.63E-01
TERF1	telomeric repeat binding factor 1	2.95E-04	9.07E-01
THNSL1	threonine synthase like 1	2.95E-04	3.40E-01
MAPRE1	microtubule associated protein RP/EB family member 1	2.96E-04	5.23E-01
BCAS2	breast carcinoma amplified sequence 2	2.96E-04	1.11
GJA3	gap junction protein alpha 3	2.96E-04	-8.53E-01
PCDH11Y	protocadherin 11 Y-linked	2.96E-04	-5.45E-01
TPGS2	tubulin polyglutamylase complex subunit 2	2.96E-04	6.89E-01
ADORA2B	adenosine A2b receptor	2.97E-04	-7.30E-01
RAB23	RAB23, member RAS oncogene family	2.97E-04	8.83E-01
VPS36	vacuolar protein sorting 36 homolog	2.97E-04	-1.26
RHBDF2	rhomboid 5 homolog 2	2.97E-04	-9.24E-01

PRMT2	protein arginine methyltransferase 2	2.97E-04	-5.66E-01
BCAS2	breast carcinoma amplified sequence 2	2.98E-04	1.37
FAM161A	family with sequence similarity 161 member A	2.98E-04	9.01E-01
CLCNKA	chloride voltage-gated channel Ka	3.00E-04	-3.71E-01
NDUFAF4	NADH:ubiquinone oxidoreductase complex assembly factor 4	3.00E-04	1.15
PCDH11X	protocadherin 11 X-linked	3.00E-04	-4.95E-01
AKIRIN1	akirin 1	3.01E-04	-7.61E-01
ZNF672	zinc finger protein 672	3.01E-04	-1.24
SCLT1	sodium channel and clathrin linker 1	3.01E-04	8.47E-01
DOCK5	dedicator of cytokinesis 5	3.02E-04	3.60E-01
CHST14	carbohydrate sulfotransferase 14	3.02E-04	-7.74E-01
ATP7B	ATPase copper transporting beta	3.02E-04	-7.18E-01
ZNF490	zinc finger protein 490	3.03E-04	-5.22E-01
RALGDS	ral guanine nucleotide dissociation stimulator	3.03E-04	-1.52
ZMAT4	zinc finger matrin-type 4	3.04E-04	-4.12E-01

HIST1H3I///HIST1H3E	histone cluster 1, H3i///histone cluster 1, H3e	3.04E-04	-7.94E-01
LITAF	lipopolysaccharide induced TNF factor	3.05E-04	-8.02E-01
GCA	grancalcin	3.05E-04	4.29E-01
RELL2	RELT like 2	3.06E-04	-6.50E-01
HMGN4	high mobility group nucleosomal binding domain 4	3.06E-04	4.57E-01
CPEB1	cytoplasmic polyadenylation element binding protein 1	3.06E-04	6.69E-01
BIRC6	baculoviral IAP repeat containing 6	3.07E-04	-8.35E-01
TMEM117	transmembrane protein 117	3.07E-04	-8.59E-01
SNORD14C	small nucleolar RNA, C/D box 14C	3.08E-04	6.34E-01
UHMK1	U2AF homology motif (UHM) kinase 1	3.08E-04	1.02
GAR1	GAR1 ribonucleoprotein	3.08E-04	1.02
RBP1	retinol binding protein 1	3.08E-04	4.26E-01
ZFPM1	zinc finger protein, FOG family member 1	3.09E-04	-5.71E-01
KCNK4	potassium two pore domain channel subfamily K member 4	3.09E-04	3.72E-01
EZR	ezrin	3.09E-04	1
CORO6	coronin 6	3.10E-04	-3.96E-01

FBXL6	F-box and leucine rich repeat protein 6	3.10E-04	6.37E-01
MEF2D	myocyte enhancer factor 2D	3.10E-04	-7.80E-01
NCL	nucleolin	3.11E-04	8.62E-01
MYL10	myosin light chain 10	3.11E-04	-4.23E-01
SNORA24	small nucleolar RNA, H/ACA box 24	3.11E-04	-1.46
SERPINI1	serpin family I member 1	3.12E-04	7.82E-01
CCDC34	coiled-coil domain containing 34	3.13E-04	9.24E-01
EIF4E	eukaryotic translation initiation factor 4E	3.15E-04	9.45E-01
KIAA2022	KIAA2022	3.15E-04	3.83E-01
EIF3I	eukaryotic translation initiation factor 3 subunit I	3.15E-04	3.81E-01
CENPT	centromere protein T	3.15E-04	5.08E-01
DHRS11	dehydrogenase/reductase 11	3.15E-04	-5.34E-01
ITGA3	integrin subunit alpha 3	3.16E-04	-8.81E-01
B3GNT8	UDP-GlcNAc:betaGal beta-1,3-N-acetylglucosaminyltransferase 8	3.17E-04	-7.51E-01
FEZ1	fasciculation and elongation protein zeta 1	3.17E-04	-5.69E-01
C10orf82	chromosome 10 open reading frame 82	3.17E-04	3.84E-01

INPP4B	inositol polyphosphate-4-phosphatase type II B	3.18E-04	-9.77E-01
ZNF219	zinc finger protein 219	3.18E-04	-7.24E-01
VMO1	vitelline membrane outer layer 1 homolog	3.19E-04	1.02
LRRK2	leucine rich repeat kinase 2	3.19E-04	4.57E-01
CRTAP	cartilage associated protein	3.19E-04	-9.98E-01
TMEM179B	transmembrane protein 179B	3.20E-04	-7.25E-01
XRCC6	X-ray repair cross complementing 6	3.21E-04	6.48E-01
PIM1	Pim-1 proto-oncogene, serine/threonine kinase	3.21E-04	-9.14E-01
ASGR1	asialoglycoprotein receptor 1	3.22E-04	-1.29
QTRT2	queuine tRNA-ribosyltransferase accessory subunit 2	3.22E-04	8.52E-01
FAM86B1	family with sequence similarity 86 member B1	3.22E-04	5.34E-01
SETD3	SET domain containing 3	3.23E-04	-5.70E-01
SOX7	SRY-box 7	3.23E-04	4.89E-01
CEP78	centrosomal protein 78	3.24E-04	7.59E-01
MMADHC	methylmalonic aciduria and homocystinuria, cblD type	3.25E-04	5.98E-01
GPM6A	glycoprotein M6A	3.25E-04	-3.62E-01

PTPRE	protein tyrosine phosphatase, receptor type E	3.25E-04	4.78E -01
ERBB3	erb-b2 receptor tyrosine kinase 3	3.25E-04	4.46E -01
RAB2B	RAB2B, member RAS oncogene family	3.26E-04	- 3.97E -01
OTUD6B-AS1	OTUD6B antisense RNA 1 (head to head)	3.27E-04	7.07E -01
GGPS1	geranylgeranyl diphosphate synthase 1	3.27E-04	- 7.26E -01
H1FX	H1 histone family member X	3.28E-04	7.63E -01
PLEKHA1	pleckstrin homology domain containing A1	3.28E-04	- 7.52E -01
CAMKK2	calcium/calmodulin dependent protein kinase kinase 2	3.28E-04	- 8.69E -01
AHI1	Abelson helper integration site 1	3.29E-04	6.42E -01
ZCCHC7	zinc finger CCHC-type containing 7	3.30E-04	5.17E -01
MFSD13A	major facilitator superfamily domain containing 13A	3.31E-04	6.32E -01
USP32	ubiquitin specific peptidase 32	3.31E-04	- 5.01E -01
CD55	CD55 molecule (Cromer blood group)	3.32E-04	6.43E -01
IGF2R	insulin like growth factor 2 receptor	3.32E-04	- 9.83E -01
SNHG4	small nucleolar RNA host gene 4	3.32E-04	- 7.13E -01

TTC4	tetratricopeptide repeat domain 4	3.33E-04	4.37E-01
FAM155B	family with sequence similarity 155 member B	3.34E-04	-5.27E-01
CLPTM1L	CLPTM1 like	3.34E-04	3.97E-01
CENPM	centromere protein M	3.35E-04	1.25
MRPL33	mitochondrial ribosomal protein L33	3.35E-04	-1.1
RASSF1	Ras association domain family member 1	3.36E-04	4.62E-01
NEMP1	nuclear envelope integral membrane protein 1	3.37E-04	1.09
SMC4	structural maintenance of chromosomes 4	3.38E-04	1.75
VGLL4	vestigial like family member 4	3.38E-04	-7.56E-01
C9orf72	chromosome 9 open reading frame 72	3.38E-04	5.22E-01
GDE1	glycerophosphodiester phosphodiesterase 1	3.38E-04	-8.21E-01
PNKP	polynucleotide kinase 3'-phosphatase	3.38E-04	-5.98E-01
PSMB3	proteasome subunit beta 3	3.38E-04	8.79E-01
SDHAP1	succinate dehydrogenase complex flavoprotein subunit A pseudogene 1	3.39E-04	5.38E-01
ATP23	ATP23 metallopeptidase and ATP synthase assembly factor homolog	3.40E-04	6.48E-01
LMNB2	lamin B2	3.42E-04	7.44E-01
BRD7	bromodomain containing 7	3.42E-04	4.71E-01

UACA	uveal autoantigen with coiled-coil domains and ankyrin repeats	3.43E-04	-6.63E-01
RECQL	RecQ like helicase	3.44E-04	-5.80E-01
DEGS1	delta 4-desaturase, sphingolipid 1	3.44E-04	-5.73E-01
FERMT2	fermitin family member 2	3.44E-04	-5.46E-01
LOC730101	uncharacterized LOC730101	3.45E-04	8.03E-01
ZNF354B	zinc finger protein 354B	3.45E-04	7.28E-01
ACAT2	acetyl-CoA acetyltransferase 2	3.46E-04	5.47E-01
ZNF551	zinc finger protein 551	3.46E-04	6.73E-01
SEC22A	SEC22 homolog A, vesicle trafficking protein	3.47E-04	4.62E-01
TSEN15	tRNA splicing endonuclease subunit 15	3.48E-04	7.42E-01
CLDN14	claudin 14	3.48E-04	-7.34E-01
DENND1B	DENN domain containing 1B	3.48E-04	-4.71E-01
CACNA1H	calcium voltage-gated channel subunit alpha1 H	3.49E-04	-1.03
FGFR4	fibroblast growth factor receptor 4	3.50E-04	-8.85E-01
ADGRV1	adhesion G protein-coupled receptor V1	3.50E-04	3.50E-01

UTP15	UTP15, small subunit processome component	3.51E-04	6.49E-01
DPYSL2	dihydropyrimidinase like 2	3.51E-04	-7.35E-01
CHURC1	churchill domain containing 1	3.51E-04	-6.75E-01
ABCC4	ATP binding cassette subfamily C member 4	3.52E-04	-7.02E-01
LDHA	lactate dehydrogenase A	3.53E-04	5.28E-01
TMEM120A	transmembrane protein 120A	3.54E-04	-7.67E-01
PSMD6	proteasome 26S subunit, non-ATPase 6	3.56E-04	4.85E-01
CDKN1B	cyclin dependent kinase inhibitor 1B	3.56E-04	-7.74E-01
TPM1	tropomyosin 1 (alpha)	3.56E-04	7.31E-01
TMED10P1	transmembrane p24 trafficking protein 10 pseudogene 1	3.56E-04	-6.15E-01
HAUS7	HAUS augmin like complex subunit 7	3.57E-04	1.3
GPSM2	G-protein signaling modulator 2	3.59E-04	7.10E-01
QTRT1	queuine tRNA-ribosyltransferase catalytic subunit 1	3.59E-04	-1.01
ZNF613	zinc finger protein 613	3.59E-04	4.91E-01
HEY1	hes related family bHLH transcription factor with YRPW motif 1	3.60E-04	3.75E-01
RBM42	RNA binding motif protein 42	3.61E-04	6.85E-01

GPC2	glypican 2	3.62E-04	-6.22E-01
SNX7	sorting nexin 7	3.62E-04	7.45E-01
RNF217	ring finger protein 217	3.63E-04	6.04E-01
CGREF1	cell growth regulator with EF-hand domain 1	3.63E-04	3.36E-01
SNRPG	small nuclear ribonucleoprotein polypeptide G	3.63E-04	8.04E-01
HACD2	3-hydroxyacyl-CoA dehydratase 2	3.64E-04	6.97E-01
MBD3L5	methyl-CpG binding domain protein 3 like 5	3.65E-04	-4.29E-01
SKAP2	src kinase associated phosphoprotein 2	3.65E-04	5.19E-01
EPS8	epidermal growth factor receptor pathway substrate 8	3.65E-04	9.67E-01
NCAPD3	non-SMC condensin II complex subunit D3	3.66E-04	8.88E-01
AP3M2	adaptor related protein complex 3 mu 2 subunit	3.66E-04	7.27E-01
PGD	phosphogluconate dehydrogenase	3.66E-04	5.55E-01
BCCIP	BRCA2 and CDKN1A interacting protein	3.67E-04	1.07
DYX1C1	dyslexia susceptibility 1 candidate 1	3.68E-04	6.81E-01
STYK1	serine/threonine/tyrosine kinase 1	3.68E-04	4.76E-01
DYRK3	dual specificity tyrosine phosphorylation regulated kinase 3	3.68E-04	5.59E-01
FAM199X	family with sequence similarity 199, X-linked	3.68E-04	9.87E-01

OGFOD3	2-oxoglutarate and iron dependent oxygenase domain containing 3	3.68E-04	-3.98E-01
TRPM4	transient receptor potential cation channel subfamily M member 4	3.69E-04	-1.11
FAF1	Fas associated factor 1	3.69E-04	7.06E-01
ODF2L	outer dense fiber of sperm tails 2 like	3.69E-04	6.44E-01
BCCIP	BRCA2 and CDKN1A interacting protein	3.69E-04	4.20E-01
MAP3K8	mitogen-activated protein kinase kinase kinase 8	3.69E-04	-3.70E-01
NOP56	NOP56 ribonucleoprotein	3.70E-04	1.02
COLGALT1	collagen beta(1-O)galactosyltransferase 1	3.70E-04	5.29E-01
SIM1	single-minded family bHLH transcription factor 1	3.70E-04	8.31E-01
MGAT4C	MGAT4 family member C	3.70E-04	-4.54E-01
ASNS	asparagine synthetase (glutamine-hydrolyzing)	3.71E-04	5.64E-01
FOXK1	forkhead box K1	3.72E-04	-5.51E-01
EBNA1BP2	EBNA1 binding protein 2	3.72E-04	5.16E-01
SP110	SP110 nuclear body protein	3.73E-04	6.82E-01
GNPDA2	glucosamine-6-phosphate deaminase 2	3.73E-04	9.26E-01
LIAS	lipoic acid synthetase	3.73E-04	-6.48E-01

PDZD8	PDZ domain containing 8	3.73E-04	9.06E<br>-01
GNAI1	G protein subunit alpha i1	3.74E-04	5.96E<br>-01
MTURN	maturin, neural progenitor differentiation regulator homolog (Xenopus)	3.75E-04	-<br>6.55E<br>-01
GPT2	glutamic--pyruvic transaminase 2	3.76E-04	-<br>9.15E<br>-01
MEIS3	Meis homeobox 3	3.77E-04	-<br>4.05E<br>-01
SUPT3H	SPT3 homolog, SAGA and STAGA complex component	3.77E-04	-<br>4.85E<br>-01
LIN9	lin-9 DREAM MuvB core complex component	3.77E-04	1.22
PDCD4	programmed cell death 4 (neoplastic transformation inhibitor)	3.77E-04	-<br>9.57E<br>-01
COL6A2	collagen type VI alpha 2 chain	3.78E-04	-<br>4.77E<br>-01
DGCR14	DiGeorge syndrome critical region gene 14	3.78E-04	3.82E<br>-01
S1PR5	sphingosine-1-phosphate receptor 5	3.78E-04	-<br>4.35E<br>-01
NOP56	NOP56 ribonucleoprotein	3.79E-04	1.11
CDK1	cyclin dependent kinase 1	3.79E-04	7.18E<br>-01
SNRPB2	small nuclear ribonucleoprotein polypeptide B2	3.79E-04	4.50E<br>-01
STMN3	stathmin 3	3.79E-04	-1.03
GMNN	geminin, DNA replication inhibitor	3.79E-04	1.11

DUSP5	dual specificity phosphatase 5	3.79E-04	5.21E-01
POP1	POP1 homolog, ribonuclease P/MRP subunit	3.79E-04	6.13E-01
LIMS1	LIM zinc finger domain containing 1	3.80E-04	7.22E-01
MRPS9	mitochondrial ribosomal protein S9	3.80E-04	4.28E-01
IMPA1	inositol monophosphatase 1	3.80E-04	5.10E-01
RHOQ	ras homolog family member Q	3.80E-04	-5.40E-01
CCDC58	coiled-coil domain containing 58	3.81E-04	2.25
FLVCR2	feline leukemia virus subgroup C cellular receptor family member 2	3.81E-04	-4.74E-01
PRELID1	PRELI domain containing 1	3.81E-04	8.73E-01
AACS	acetoacetyl-CoA synthetase	3.82E-04	-5.47E-01
EPM2AIP1	EPM2A interacting protein 1	3.84E-04	-3.75E-01
GLS	glutaminase	3.85E-04	-8.04E-01
SLC39A1	solute carrier family 39 member 1	3.86E-04	-4.46E-01
BARX2	BARX homeobox 2	3.86E-04	-4.27E-01
TMEM187	transmembrane protein 187	3.86E-04	5.79E-01

CERS1	ceramide synthase 1	3.87E-04	-4.02E-01
C18orf54	chromosome 18 open reading frame 54	3.87E-04	5.45E-01
PSMG3-AS1	PSMG3 antisense RNA 1 (head to head)	3.87E-04	-3.29E-01
C9orf72	chromosome 9 open reading frame 72	3.88E-04	3.70E-01
MPHOSPH10	M-phase phosphoprotein 10	3.89E-04	6.91E-01
RASSF1	Ras association domain family member 1	3.89E-04	3.73E-01
KPNA5	karyopherin subunit alpha 5	3.89E-04	-4.57E-01
MRPL3	mitochondrial ribosomal protein L3	3.90E-04	7.21E-01
HLA-DMA	major histocompatibility complex, class II, DM alpha	3.90E-04	-1.1
NAA30	N(alpha)-acetyltransferase 30, NatC catalytic subunit	3.90E-04	6.49E-01
AMER1	APC membrane recruitment protein 1	3.90E-04	-9.16E-01
STUB1	STIP1 homology and U-box containing protein 1	3.90E-04	-8.02E-01
ZDHHC1	zinc finger DHHC-type containing 1	3.90E-04	-8.61E-01
TSPAN13	tetraspanin 13	3.91E-04	7.59E-01
XPO4	exportin 4	3.92E-04	5.71E-01

ABCB1	ATP binding cassette subfamily B member 1	3.93E-04	4.32E-01
ARL4A	ADP ribosylation factor like GTPase 4A	3.93E-04	1.09
ZNF207	zinc finger protein 207	3.93E-04	3.66E-01
GFM1	G elongation factor mitochondrial 1	3.94E-04	7.89E-01
LHPP	phospholysine phosphohistidine inorganic pyrophosphate phosphatase	3.95E-04	-7.02E-01
TLCD1	TLC domain containing 1	3.95E-04	-1.26
CEP72	centrosomal protein 72	3.96E-04	5.77E-01
PPIC	peptidylprolyl isomerase C	3.96E-04	-9.89E-01
DRG1	developmentally regulated GTP binding protein 1	3.97E-04	5.54E-01
PLXNB2	plexin B2	3.97E-04	-1.33
SRSF3	serine and arginine rich splicing factor 3	3.97E-04	8.90E-01
PGK1	phosphoglycerate kinase 1	3.98E-04	-3.42E-01
ZSWIM8	zinc finger SWIM-type containing 8	3.98E-04	-7.58E-01
ZMAT3	zinc finger matrin-type 3	3.98E-04	-8.32E-01
PLOD2	procollagen-lysine,2-oxoglutarate 5-dioxygenase 2	3.99E-04	-8.05E-01
LMTK2	lemur tyrosine kinase 2	4.00E-04	4.27E-01

LPCAT4	lysophosphatidylcholine acyltransferase 4	4.01E-04	-4.16E-01
EEF1A2	eukaryotic translation elongation factor 1 alpha 2	4.01E-04	1.3
TMEM69	transmembrane protein 69	4.01E-04	-9.46E-01
HGF	hepatocyte growth factor	4.02E-04	-7.80E-01
TOR1AIP2	torsin 1A interacting protein 2	4.04E-04	7.38E-01
DENR	density regulated re-initiation and release factor	4.06E-04	6.58E-01
PRKACB	protein kinase cAMP-activated catalytic subunit beta	4.07E-04	3.87E-01
CCDC90B	coiled-coil domain containing 90B	4.08E-04	-5.59E-01
ETF1	eukaryotic translation termination factor 1	4.08E-04	4.08E-01
CDC25C	cell division cycle 25C	4.08E-04	8.89E-01
BMP1	bone morphogenetic protein 1	4.09E-04	-5.23E-01
STAP2	signal transducing adaptor family member 2	4.09E-04	3.67E-01
C5	complement component 5	4.09E-04	4.76E-01
FAM220A	family with sequence similarity 220 member A	4.10E-04	6.47E-01
KIAA0895L	KIAA0895 like	4.10E-04	-8.57E-01

SNORA40	small nucleolar RNA, H/ACA box 40	4.10E-04	5.37E-01
ZNF33A	zinc finger protein 33A	4.10E-04	-6.45E-01
ANP32A	acidic nuclear phosphoprotein 32 family member A	4.11E-04	8.02E-01
TWISTNB	TWIST neighbor	4.11E-04	4.18E-01
NKIRAS2	NFKB inhibitor interacting Ras like 2	4.12E-04	-4.82E-01
COMMD10	COMM domain containing 10	4.12E-04	5.09E-01
C16orf72	chromosome 16 open reading frame 72	4.12E-04	-4.94E-01
EXTL3	exostosin like glycosyltransferase 3	4.13E-04	-7.76E-01
PDDC1	Parkinson disease 7 domain containing 1	4.13E-04	-7.71E-01
MNAT1	MNAT1, CDK activating kinase assembly factor	4.13E-04	7.56E-01
LUC7L3	LUC7 like 3 pre-mRNA splicing factor	4.14E-04	-1.82
RBM47	RNA binding motif protein 47	4.14E-04	3.75E-01
C22orf39	chromosome 22 open reading frame 39	4.14E-04	5.55E-01
ZBED8	zinc finger BED-type containing 8	4.14E-04	6.75E-01
EFNA1	ephrin A1	4.17E-04	-7.23E-01

NME4	NME/NM23 nucleoside diphosphate kinase 4	4.17E-04	-8.16E-01
CACFD1	calcium channel flower domain containing 1	4.17E-04	-3.32E-01
GNPDA2	glucosamine-6-phosphate deaminase 2	4.17E-04	9.09E-01
CNOT10	CCR4-NOT transcription complex subunit 10	4.18E-04	4.53E-01
CUL7	cullin 7	4.19E-04	-5.74E-01
MPZL2	myelin protein zero like 2	4.20E-04	-4.25E-01
DDX20	DEAD-box helicase 20	4.20E-04	4.50E-01
EIF3J	eukaryotic translation initiation factor 3 subunit J	4.21E-04	8.53E-01
PPT2	palmitoyl-protein thioesterase 2	4.22E-04	-6.05E-01
INTS7	integrator complex subunit 7	4.22E-04	7.68E-01
ARHGAP32	Rho GTPase activating protein 32	4.24E-04	-1.68
PTCHD1	patched domain containing 1	4.24E-04	4.76E-01
SGO2	shugoshin 2	4.24E-04	5.01E-01
NPHP1	nephrocystin 1	4.26E-04	7.22E-01
OSTC	oligosaccharyltransferase complex non-catalytic subunit	4.26E-04	-6.47E-01
AAED1	AhpC/TSA antioxidant enzyme domain containing 1	4.27E-04	8.73E-01

RBM7	RNA binding motif protein 7	4.27E-04	6.78E-01
MRPL18	mitochondrial ribosomal protein L18	4.27E-04	4.12E-01
ZMAT3	zinc finger matrin-type 3	4.27E-04	-8.62E-01
CDCA7L	cell division cycle associated 7 like	4.27E-04	9.23E-01
MTA3	metastasis associated 1 family member 3	4.28E-04	-3.69E-01
TMED4	transmembrane p24 trafficking protein 4	4.30E-04	-8.78E-01
GAMT	guanidinoacetate N-methyltransferase	4.30E-04	-1.38
TSPYL2	TSPY like 2	4.30E-04	-8.85E-01
DHRS7	dehydrogenase/reductase 7	4.31E-04	-4.52E-01
SRSF3	serine and arginine rich splicing factor 3	4.31E-04	7.75E-01
MSI2	musashi RNA binding protein 2	4.32E-04	-7.34E-01
HAUS1	HAUS augmin like complex subunit 1	4.32E-04	1.26
MCM7	minichromosome maintenance complex component 7	4.33E-04	5.36E-01
BEX3	brain expressed X-linked 3	4.34E-04	-5.64E-01
PTPN21	protein tyrosine phosphatase, non-receptor type 21	4.36E-04	4.47E-01
ME1	malic enzyme 1	4.37E-04	1.15

CTSB	cathepsin B	4.37E-04	-9.73E-01
GPN3	GPN-loop GTPase 3	4.38E-04	7.77E-01
MTFR1	mitochondrial fission regulator 1	4.38E-04	1.37
CLIC4	chloride intracellular channel 4	4.38E-04	3.75E-01
LOC440040	glutamate metabotropic receptor 5 pseudogene	4.40E-04	-3.86E-01
IFT122	intraflagellar transport 122	4.40E-04	3.93E-01
BCL6	B-cell CLL/lymphoma 6	4.42E-04	-4.66E-01
PGGHG	protein-glucosylgalactosylhydroxylysine glucosidase	4.43E-04	-1.27
VWA8	von Willebrand factor A domain containing 8	4.43E-04	-1.05
EFNA4	ephrin A4	4.43E-04	-1.16
SMARCE1	SWI/SNF related, matrix associated, actin dependent regulator of chromatin, subfamily e, member 1	4.43E-04	4.96E-01
RPL3	ribosomal protein L3	4.43E-04	-4.78E-01
PLS1	plastin 1	4.43E-04	1.39
CDK20	cyclin dependent kinase 20	4.43E-04	5.64E-01
MGP	matrix Gla protein	4.43E-04	-1.47
SNRNP25	small nuclear ribonucleoprotein U11/U12 subunit 25	4.44E-04	1.36
CCDC18	coiled-coil domain containing 18	4.44E-04	5.47E-01
C7orf25	chromosome 7 open reading frame 25	4.45E-04	6.18E-01

STEAP1	six transmembrane epithelial antigen of the prostate 1	4.45E-04	8.88E-01
MT2A	metallothionein 2A	4.45E-04	1.26
WHAMM	WAS protein homolog associated with actin, golgi membranes and microtubules	4.46E-04	5.25E-01
ENAH	enabled homolog (Drosophila)	4.46E-04	7.31E-01
PHB2	prohibitin 2	4.48E-04	-4.15E-01
MANSC1	MANSC domain containing 1	4.48E-04	7.85E-01
ATMIN	ATM interactor	4.49E-04	6.24E-01
CETN3	centrin 3	4.49E-04	8.98E-01
ZBTB22	zinc finger and BTB domain containing 22	4.49E-04	-6.85E-01
LSR	lipolysis stimulated lipoprotein receptor	4.50E-04	7.14E-01
TRAF5	TNF receptor associated factor 5	4.50E-04	9.07E-01
ATPAF2	ATP synthase mitochondrial F1 complex assembly factor 2	4.50E-04	8.02E-01
CAPN5	calpain 5	4.50E-04	-7.54E-01
GPNMB	glycoprotein nmb	4.52E-04	3.25E-01
DDTL	D-dopachrome tautomerase-like	4.52E-04	-4.77E-01
MINA	MYC induced nuclear antigen	4.53E-04	3.78E-01
TOR1AIP2	torsin 1A interacting protein 2	4.55E-04	8.60E-01

ANO3	anoctamin 3	4.55E-04	1.11
ZEB2	zinc finger E-box binding homeobox 2	4.55E-04	3.99E-01
PCNX1	pecanex homolog 1 (Drosophila)	4.55E-04	-7.24E-01
MIR503HG	MIR503 host gene	4.56E-04	-1.09
SLC25A38	solute carrier family 25 member 38	4.57E-04	-3.49E-01
THBS1	thrombospondin 1	4.59E-04	4.00E-01
STAP2	signal transducing adaptor family member 2	4.60E-04	4.52E-01
RXRA	retinoid X receptor alpha	4.61E-04	-1.25
EXOSC10	exosome component 10	4.61E-04	5.77E-01
PARD6A	par-6 family cell polarity regulator alpha	4.62E-04	8.26E-01
SF1	splicing factor 1	4.63E-04	-1.7
IFITM3	interferon induced transmembrane protein 3	4.63E-04	4.93E-01
CHUK	conserved helix-loop-helix ubiquitous kinase	4.63E-04	5.36E-01
C15orf52	chromosome 15 open reading frame 52	4.64E-04	-3.97E-01
ZNF140	zinc finger protein 140	4.64E-04	5.62E-01
H2AFY	H2A histone family member Y	4.65E-04	5.97E-01
CEP89	centrosomal protein 89	4.65E-04	4.01E-01
MXD3	MAX dimerization protein 3	4.66E-04	3.93E-01

KIAA0922	KIAA0922	4.66E-04	5.68E<br>-01
C21orf33	chromosome 21 open reading frame 33	4.67E-04	-1.4
FOXN3	forkhead box N3	4.68E-04	-1.23
PCGF6	polycomb group ring finger 6	4.68E-04	7.23E<br>-01
LRRC40	leucine rich repeat containing 40	4.68E-04	1.03
DRAXIN	dorsal inhibitory axon guidance protein	4.70E-04	-<br>4.58E<br>-01
POMZP3	POM121 and ZP3 fusion	4.70E-04	7.49E<br>-01
PTCD2	pentatricopeptide repeat domain 2	4.70E-04	-<br>5.04E<br>-01
ZNF17	zinc finger protein 17	4.72E-04	4.64E<br>-01
NR1H3	nuclear receptor subfamily 1 group H member 3	4.72E-04	-1.24
TDRP	testis development related protein	4.73E-04	-<br>5.99E<br>-01
NT5C	5', 3'-nucleotidase, cytosolic	4.73E-04	-<br>9.18E<br>-01
MTHFS	5,10-methenyltetrahydrofolate synthetase (5-formyltetrahydrofolate cyclo-ligase)	4.73E-04	8.50E<br>-01
SNORD110	small nucleolar RNA, C/D box 110	4.73E-04	5.32E<br>-01
GADD45A	growth arrest and DNA damage inducible alpha	4.74E-04	-1.04
SAR1B	secretion associated Ras related GTPase 1B	4.74E-04	8.59E<br>-01
DNAJC12	DnaJ heat shock protein family (Hsp40) member C12	4.74E-04	-<br>7.72E<br>-01

ZNF433	zinc finger protein 433	4.74E-04	-3.69E-01
ABCG1	ATP binding cassette subfamily G member 1	4.75E-04	-3.91E-01
PRDX5	peroxiredoxin 5	4.75E-04	-4.08E-01
RALGDS	ral guanine nucleotide dissociation stimulator	4.76E-04	-4.30E-01
CALM2	calmodulin 2	4.76E-04	5.80E-01
KREMEN2	kringle containing transmembrane protein 2	4.76E-04	-5.05E-01
CREBRF	CREB3 regulatory factor	4.77E-04	-9.09E-01
DUSP22	dual specificity phosphatase 22	4.78E-04	-4.53E-01
TTC31	tetratricopeptide repeat domain 31	4.80E-04	9.25E-01
TUBB6	tubulin beta 6 class V	4.80E-04	7.03E-01
IGBP1	immunoglobulin (CD79A) binding protein 1	4.82E-04	-7.91E-01
SNX2	sorting nexin 2	4.83E-04	-3.82E-01
TSPAN12	tetraspanin 12	4.83E-04	4.84E-01
ADM	adrenomedullin	4.83E-04	-9.53E-01

SIL1	SIL1 nucleotide exchange factor	4.85E-04	-1.12
FAM127C	family with sequence similarity 127 member C	4.85E-04	-1.54
BCS1L	BCS1 homolog, ubiquinol-cytochrome c reductase complex chaperone	4.86E-04	6.91E-01
PKN3	protein kinase N3	4.86E-04	8.97E-01
EFCAB7	EF-hand calcium binding domain 7	4.86E-04	7.57E-01
METTL23	methyltransferase like 23	4.86E-04	-1.02
SYF2	SYF2 pre-mRNA splicing factor	4.87E-04	-6.39E-01
IZUMO4	IZUMO family member 4	4.87E-04	-3.67E-01
KIAA1804	mixed lineage kinase 4	4.88E-04	3.87E-01
SCARB2	scavenger receptor class B member 2	4.88E-04	-6.45E-01
MMADHC	methylmalonic aciduria and homocystinuria, cblD type	4.88E-04	5.59E-01
EIF3M	eukaryotic translation initiation factor 3 subunit M	4.88E-04	4.51E-01
FAT1	FAT atypical cadherin 1	4.89E-04	-1.13
PTK2	protein tyrosine kinase 2	4.90E-04	-6.91E-01
SEC62	SEC62 homolog, preprotein translocation factor	4.91E-04	-5.65E-01
AGK	acylglycerol kinase	4.92E-04	5.09E-01
TRA2B	transformer 2 beta homolog (Drosophila)	4.92E-04	6.13E-01

XBP1	X-box binding protein 1	4.93E-04	-4.15E-01
ERGIC2	ERGIC and golgi 2	4.93E-04	5.05E-01
ANKLE2	ankyrin repeat and LEM domain containing 2	4.94E-04	4.83E-01
BPGM	bisphosphoglycerate mutase	4.94E-04	5.79E-01
CEP295	centrosomal protein 295	4.94E-04	1.06
KLRG1	killer cell lectin like receptor G1	4.95E-04	-8.11E-01
GAA	glucosidase alpha, acid	4.96E-04	-1.23
TBC1D22A	TBC1 domain family member 22A	4.96E-04	-7.54E-01
KMT5A	lysine methyltransferase 5A	4.96E-04	5.26E-01
FRG1	FSHD region gene 1	4.97E-04	4.96E-01
ETNK1	ethanolamine kinase 1	4.99E-04	4.78E-01
TST	thiosulfate sulfurtransferase	4.99E-04	-1.33
SQRDL	sulfide quinone reductase-like (yeast)	4.99E-04	7.35E-01
MST1	macrophage stimulating 1	5.00E-04	-7.51E-01
PSIP1	PC4 and SFRS1 interacting protein 1	5.00E-04	7.68E-01
EMILIN1	elastin microfibril interfacer 1	5.00E-04	-5.49E-01
LTA4H	leukotriene A4 hydrolase	5.00E-04	-3.84E-01

HIST2H2BE	histone cluster 2, H2be	5.00E-04	-3.22E-01
ARL6IP5	ADP ribosylation factor like GTPase 6 interacting protein 5	5.01E-04	-4.87E-01
PHOSPHO2	phosphatase, orphan 2	5.01E-04	5.97E-01
ANGPT1	angiopoietin 1	5.01E-04	-9.13E-01
SRSF2	serine and arginine rich splicing factor 2	5.02E-04	4.51E-01
VWA1	von Willebrand factor A domain containing 1	5.02E-04	-3.77E-01
HSD17B6	hydroxysteroid 17-beta dehydrogenase 6	5.03E-04	4.23E-01
LYPD6B	LY6/PLAUR domain containing 6B	5.04E-04	-4.15E-01
TMEM80	transmembrane protein 80	5.04E-04	-6.92E-01
CDC25B	cell division cycle 25B	5.05E-04	9.80E-01
NIT1	nitrilase 1	5.05E-04	-3.22E-01
TMEM110	transmembrane protein 110	5.05E-04	3.63E-01
PGM1	phosphoglucomutase 1	5.06E-04	-6.77E-01
HNRNPA1L2	heterogeneous nuclear ribonucleoprotein A1-like 2	5.06E-04	-8.13E-01

TMPRSS15	transmembrane protease, serine 15	5.06E-04	5.89E-01
HIF1A	hypoxia inducible factor 1 alpha subunit	5.08E-04	1.05
IER3	immediate early response 3	5.08E-04	-1.35
SULT1A1	sulfotransferase family 1A member 1	5.10E-04	-5.77E-01
MYOM2	myomesin 2	5.11E-04	4.86E-01
COL9A2	collagen type IX alpha 2 chain	5.12E-04	-6.44E-01
SLC25A20	solute carrier family 25 member 20	5.12E-04	-3.95E-01
TRIM9	tripartite motif containing 9	5.12E-04	6.28E-01
FAM149B1	family with sequence similarity 149 member B1	5.13E-04	-7.97E-01
GPRIN1	G protein regulated inducer of neurite outgrowth 1	5.13E-04	4.98E-01
ATF6	activating transcription factor 6	5.14E-04	4.74E-01
PIGV	phosphatidylinositol glycan anchor biosynthesis class V	5.15E-04	-7.10E-01
CDC123	cell division cycle 123	5.15E-04	5.43E-01
CD83	CD83 molecule	5.18E-04	-5.17E-01
PPP1R3D	protein phosphatase 1 regulatory subunit 3D	5.20E-04	-9.40E-01

CARD16	caspase recruitment domain family member 16	5.21E-04	-5.35E-01
DHDDS	dehydrodolichyl diphosphate synthase subunit	5.22E-04	4.17E-01
DMRTA1	DMRT like family A1	5.22E-04	4.45E-01
ADARB1	adenosine deaminase, RNA specific B1	5.24E-04	-4.56E-01
CTSC	cathepsin C	5.24E-04	5.91E-01
DNA2	DNA replication helicase/nuclease 2	5.24E-04	4.16E-01
SLC35B2	solute carrier family 35 member B2	5.25E-04	-5.08E-01
RARRES3	retinoic acid receptor responder 3	5.26E-04	3.94E-01
CENPI	centromere protein I	5.26E-04	4.65E-01
APLP1	amyloid beta precursor like protein 1	5.27E-04	-1.26
MFSD14A	major facilitator superfamily domain containing 14A	5.27E-04	-7.51E-01
SNRK	SNF related kinase	5.27E-04	4.68E-01
UBA52	ubiquitin A-52 residue ribosomal protein fusion product 1	5.29E-04	-1.08
AKR1C2	aldo-keto reductase family 1, member C2	5.29E-04	-4.97E-01
HPS3	HPS3, biogenesis of lysosomal organelles complex 2 subunit 1	5.29E-04	1.43
CDC6	cell division cycle 6	5.30E-04	4.25E-01

PYGB	phosphorylase, glycogen; brain	5.31E-04	9.87E -01
FAM175B	family with sequence similarity 175 member B	5.31E-04	3.57E -01
ARSG	arylsulfatase G	5.31E-04	- 4.49E -01
ANKLE1	ankyrin repeat and LEM domain containing 1	5.32E-04	- 5.47E -01
NQO1	NAD(P)H quinone dehydrogenase 1	5.32E-04	- 6.03E -01
BCKDHA	branched chain keto acid dehydrogenase E1, alpha polypeptide	5.32E-04	-1.26
LINC00997	long intergenic non-protein coding RNA 997	5.32E-04	- 3.39E -01
ASCC3	activating signal cointegrator 1 complex subunit 3	5.32E-04	-1.15
MED4	mediator complex subunit 4	5.33E-04	- 6.87E -01
CRISPLD2	cysteine rich secretory protein LCCL domain containing 2	5.34E-04	- 4.85E -01
EFL1	elongation factor like GTPase 1	5.34E-04	6.68E -01
VKORC1L1	vitamin K epoxide reductase complex subunit 1 like 1	5.34E-04	8.22E -01
LRRN2	leucine rich repeat neuronal 2	5.35E-04	3.77E -01
SLC26A6	solute carrier family 26 member 6	5.35E-04	- 5.27E -01
KIF5C	kinesin family member 5C	5.35E-04	4.02E -01

TIPARP	TCDD inducible poly(ADP-ribose) polymerase	5.36E-04	8.63E -01
HYAL2	hyaluronoglucosaminidase 2	5.37E-04	-5.23E -01
SAMD4A	sterile alpha motif domain containing 4A	5.37E-04	-5.50E -01
SPC25	SPC25, NDC80 kinetochore complex component	5.37E-04	4.86E -01
COPS8	COP9 signalosome subunit 8	5.38E-04	6.92E -01
TRIM5	tripartite motif containing 5	5.39E-04	-1.04
ANXA11	annexin A11	5.39E-04	-8.02E -01
MTMR11	myotubularin related protein 11	5.39E-04	5.70E -01
PPIL6	peptidylprolyl isomerase like 6	5.40E-04	6.49E -01
SEC11A	SEC11 homolog A, signal peptidase complex subunit	5.41E-04	-5.90E -01
ACAD8	acyl-CoA dehydrogenase family member 8	5.41E-04	-6.18E -01
DPH2	DPH2 homolog	5.41E-04	5.97E -01
SLC25A37	solute carrier family 25 member 37	5.41E-04	-5.27E -01
PRAMEF5	PRAME family member 5	5.42E-04	-3.79E -01
NIFK	nucleolar protein interacting with the FHA domain of MKI67	5.42E-04	5.78E -01

GIMAP2	GTPase, IMAP family member 2	5.43E-04	3.75E-01
SETD4	SET domain containing 4	5.43E-04	7.11E-01
SLITRK4	SLIT and NTRK like family member 4	5.43E-04	-6.55E-01
CPEB3	cytoplasmic polyadenylation element binding protein 3	5.43E-04	-5.25E-01
CYP26A1	cytochrome P450 family 26 subfamily A member 1	5.43E-04	-4.79E-01
SRSF5	serine and arginine rich splicing factor 5	5.44E-04	-5.19E-01
ID3	inhibitor of DNA binding 3, HLH protein	5.46E-04	6.96E-01
RNF141	ring finger protein 141	5.47E-04	4.37E-01
MPV17	MPV17, mitochondrial inner membrane protein	5.47E-04	-9.97E-01
SENP5	SUMO1/sentrin specific peptidase 5	5.47E-04	4.93E-01
CFAP20	cilia and flagella associated protein 20	5.48E-04	5.83E-01
SNRPD3	small nuclear ribonucleoprotein D3 polypeptide	5.50E-04	-6.94E-01
PIN4	peptidylprolyl cis/trans isomerase, NIMA-interacting 4	5.50E-04	-4.43E-01
NCOA1	nuclear receptor coactivator 1	5.51E-04	-5.78E-01

ADNP	activity dependent neuroprotector homeobox	5.51E-04	-3.06E-01
ZBTB5	zinc finger and BTB domain containing 5	5.53E-04	-1.26
MXRA7	matrix remodeling associated 7	5.53E-04	-1.02
PAXBP1	PAX3 and PAX7 binding protein 1	5.54E-04	4.09E-01
CARD16	caspase recruitment domain family member 16	5.55E-04	-4.91E-01
RNASEK	ribonuclease K	5.56E-04	-5.26E-01
CDC42SE2	CDC42 small effector 2	5.57E-04	7.38E-01
INPP5B	inositol polyphosphate-5-phosphatase B	5.57E-04	-3.23E-01
RANGRF	RAN guanine nucleotide release factor	5.58E-04	6.47E-01
PPME1	protein phosphatase methylesterase 1	5.58E-04	6.52E-01
MDK	midkine (neurite growth-promoting factor 2)	5.59E-04	4.31E-01
CABYR	calcium binding tyrosine phosphorylation regulated	5.61E-04	-6.38E-01
AMY1B	amylase, alpha 1B (salivary)	5.61E-04	5.22E-01
UQCC1	ubiquinol-cytochrome c reductase complex assembly factor 1	5.61E-04	-6.59E-01
ABCC5	ATP binding cassette subfamily C member 5	5.61E-04	-9.02E-01

CCP110	centriolar coiled-coil protein 110	5.62E-04	-3.84E-01
CCDC121	coiled-coil domain containing 121	5.63E-04	-5.05E-01
HDHD2	haloacid dehalogenase like hydrolase domain containing 2	5.64E-04	-4.41E-01
PSMC3IP	PSMC3 interacting protein	5.65E-04	6.61E-01
ATG16L1	autophagy related 16 like 1	5.65E-04	6.97E-01
APITD1	apoptosis-inducing, TAF9-like domain 1	5.65E-04	5.14E-01
CPVL	carboxypeptidase, vitellogenic like	5.67E-04	3.42E-01
CDK5RAP1	CDK5 regulatory subunit associated protein 1	5.67E-04	5.29E-01
TUBE1	tubulin epsilon 1	5.67E-04	5.00E-01
SP100	SP100 nuclear antigen	5.68E-04	4.23E-01
OSBPL9	oxysterol binding protein like 9	5.68E-04	6.18E-01
C1GALT1C1	C1GALT1 specific chaperone 1	5.68E-04	-6.66E-01
ADAMTS3	ADAM metallopeptidase with thrombospondin type 1 motif 3	5.69E-04	8.84E-01
ELAVL1	ELAV like RNA binding protein 1	5.69E-04	-5.88E-01
THRA	thyroid hormone receptor, alpha	5.70E-04	-4.88E-01

MAP3K1	mitogen-activated protein kinase kinase kinase 1	5.70E-04	4.41E-01
TMEM25	transmembrane protein 25	5.70E-04	4.57E-01
PCDHGB6	protocadherin gamma subfamily B, 6	5.72E-04	-7.48E-01
RUNDC1	RUN domain containing 1	5.72E-04	5.53E-01
DLGAP1	DLG associated protein 1	5.72E-04	4.05E-01
WNK1	WNK lysine deficient protein kinase 1	5.73E-04	4.12E-01
RPL39L	ribosomal protein L39 like	5.74E-04	1.44
SSSCA1	Sjogren syndrome/scleroderma autoantigen 1	5.75E-04	8.65E-01
FDXR	ferredoxin reductase	5.75E-04	-9.18E-01
MFSD14A	major facilitator superfamily domain containing 14A	5.75E-04	-6.06E-01
NACC2	NACC family member 2	5.76E-04	4.05E-01
CTRL	chymotrypsin like	5.76E-04	4.98E-01
ABCF2	ATP binding cassette subfamily F member 2	5.76E-04	6.98E-01
TBPL1	TATA-box binding protein like 1	5.76E-04	5.55E-01
CHST12	carbohydrate (chondroitin 4) sulfotransferase 12	5.77E-04	-8.23E-01
POTEF	POTE ankyrin domain family member F	5.78E-04	5.19E-01
HYLS1	HYLS1, centriolar and ciliogenesis associated	5.80E-04	4.62E-01

SEC14L1	SEC14 like lipid binding 1	5.81E-04	-7.20E-01
MCOLN3	mucolipin 3	5.81E-04	-9.27E-01
RPL10A	ribosomal protein L10a	5.82E-04	-7.28E-01
ZNF407	zinc finger protein 407	5.83E-04	-4.12E-01
CTPS2	CTP synthase 2	5.83E-04	6.96E-01
TRO	trophinin	5.83E-04	-5.99E-01
SLC24A1	solute carrier family 24 member 1	5.85E-04	-3.72E-01
EIF2S1	eukaryotic translation initiation factor 2 subunit alpha	5.86E-04	6.04E-01
OLMALINC	oligodendrocyte maturation-associated long intergenic non-coding RNA	5.86E-04	-9.10E-01
BBS4	Bardet-Biedl syndrome 4	5.87E-04	-7.27E-01
MUSK	muscle associated receptor tyrosine kinase	5.87E-04	4.45E-01
LOC646214	p21 protein (Cdc42/Rac)-activated kinase 2 pseudogene	5.88E-04	8.28E-01
ADRM1	adhesion regulating molecule 1	5.88E-04	5.36E-01
ASAH2B	N-acylsphingosine amidohydrolase 2B	5.89E-04	6.81E-01
LAMA5	laminin subunit alpha 5	5.89E-04	-1.11

CTSB	cathepsin B	5.89E-04	-8.40E-01
XKR6	XK related 6	5.90E-04	-9.04E-01
CDKN2A	cyclin dependent kinase inhibitor 2A	5.92E-04	1.26
FAM91A1	family with sequence similarity 91 member A1	5.92E-04	8.68E-01
CSE1L	chromosome segregation 1 like	5.93E-04	1.3
HNRNPM	heterogeneous nuclear ribonucleoprotein M	5.93E-04	6.85E-01
ELOVL6	ELOVL fatty acid elongase 6	5.93E-04	-4.09E-01
MARS	methionyl-tRNA synthetase	5.94E-04	8.82E-01
WTAP	Wilms tumor 1 associated protein	5.95E-04	9.80E-01
C1D	C1D nuclear receptor corepressor	5.96E-04	1.13
TDO2	tryptophan 2,3-dioxygenase	5.96E-04	5.66E-01
POU2F1	POU class 2 homeobox 1	5.99E-04	5.47E-01
TMEM184C	transmembrane protein 184C	6.00E-04	-5.97E-01
NBPF10	neuroblastoma breakpoint family member 10	6.01E-04	4.92E-01
EXOC6	exocyst complex component 6	6.02E-04	9.39E-01
TGFB2	transforming growth factor beta 2	6.02E-04	-1.07
SERF2	small EDRK-rich factor 2	6.03E-04	-5.69E-01

PRDX1	peroxiredoxin 1	6.04E-04	4.60E -01
CFH	complement factor H	6.04E-04	- 5.48E -01
NEMP1	nuclear envelope integral membrane protein 1	6.05E-04	1.11
SBDS	SBDS ribosome assembly guanine nucleotide exchange factor	6.08E-04	6.70E -01
MT1G	metallothionein 1G	6.11E-04	3.23E -01
CNOT7	CCR4-NOT transcription complex subunit 7	6.11E-04	7.98E -01
ANAPC1	anaphase promoting complex subunit 1	6.12E-04	- 5.38E -01
SNHG7	small nucleolar RNA host gene 7	6.13E-04	-1.25
ATP5G3	ATP synthase, H+ transporting, mitochondrial Fo complex subunit C3 (subunit 9)	6.13E-04	- 4.81E -01
BAALC	brain and acute leukemia, cytoplasmic	6.15E-04	3.63E -01
SMTN	smoothelin	6.16E-04	- 6.92E -01
XRCC4	X-ray repair cross complementing 4	6.16E-04	8.83E -01
PRR14L	proline rich 14 like	6.16E-04	- 4.91E -01
SPP1	secreted phosphoprotein 1	6.17E-04	- 3.68E -01
HINT3	histidine triad nucleotide binding protein 3	6.17E-04	8.73E -01
USP3	ubiquitin specific peptidase 3	6.20E-04	5.45E -01

CCDC127	coiled-coil domain containing 127	6.21E-04	-5.95E-01
SYT14	synaptotagmin 14	6.23E-04	3.07E-01
EFCAB11	EF-hand calcium binding domain 11	6.23E-04	1.11
DYRK3	dual specificity tyrosine phosphorylation regulated kinase 3	6.25E-04	5.08E-01
RIPK2	receptor interacting serine/threonine kinase 2	6.26E-04	5.64E-01
BMP2K	BMP2 inducible kinase	6.26E-04	4.79E-01
BAIAP2L2	BAI1 associated protein 2 like 2	6.26E-04	-6.50E-01
GLRX3	glutaredoxin 3	6.26E-04	7.59E-01
TBX2	T-box 2	6.30E-04	-1.13
TWF1	twinfilin actin binding protein 1	6.30E-04	1.3
SYVN1	synoviolin 1	6.31E-04	-7.84E-01
SLC16A10	solute carrier family 16 member 10	6.31E-04	-4.31E-01
KPNA2	karyopherin subunit alpha 2	6.32E-04	7.92E-01
ANXA5	annexin A5	6.34E-04	-4.61E-01
SEMA3E	semaphorin 3E	6.34E-04	5.53E-01
RPS15A	ribosomal protein S15a	6.35E-04	-3.96E-01

TCIRG1	T-cell immune regulator 1, ATPase H+ transporting V0 subunit a3	6.35E-04	-8.00E-01
HTR7	5-hydroxytryptamine receptor 7	6.37E-04	5.44E-01
CCDC50	coiled-coil domain containing 50	6.37E-04	7.90E-01
C19orf12	chromosome 19 open reading frame 12	6.39E-04	-5.07E-01
SYCE2	synaptonemal complex central element protein 2	6.39E-04	3.63E-01
SGSM2	small G protein signaling modulator 2	6.40E-04	-7.88E-01
HNRNPCL3	heterogeneous nuclear ribonucleoprotein C-like 3	6.41E-04	5.72E-01
ATP6AP2	ATPase H+ transporting accessory protein 2	6.42E-04	-4.26E-01
CASP7	caspase 7	6.42E-04	4.34E-01
YEATS4	YEATS domain containing 4	6.42E-04	9.62E-01
POFUT2	protein O-fucosyltransferase 2	6.43E-04	-7.51E-01
PRR7	proline rich 7 (synaptic)	6.44E-04	-6.85E-01
KCNQ1OT1	KCNQ1 opposite strand/antisense transcript 1 (non-protein coding)	6.44E-04	-7.34E-01
TRIP4	thyroid hormone receptor interactor 4	6.44E-04	4.56E-01
RABGGTA	Rab geranylgeranyltransferase alpha subunit	6.45E-04	-5.34E-01

SETDB1	SET domain bifurcated 1	6.45E-04	-7.96E-01
ATP5SL	ATP5S like	6.46E-04	-5.73E-01
HYAL1	hyaluronoglucosaminidase 1	6.46E-04	-4.93E-01
SOGA1	suppressor of glucose, autophagy associated 1	6.46E-04	-8.83E-01
ZNF233	zinc finger protein 233	6.48E-04	5.75E-01
HPRT1	hypoxanthine phosphoribosyltransferase 1	6.48E-04	3.74E-01
LIME1	Lck interacting transmembrane adaptor 1	6.48E-04	-5.97E-01
NOTCH1	notch 1	6.49E-04	-6.48E-01
ZNF491	zinc finger protein 491	6.49E-04	-5.01E-01
ANKAR	ankyrin and armadillo repeat containing	6.50E-04	2.98E-01
DNAJC4	DnaJ heat shock protein family (Hsp40) member C4	6.50E-04	-6.88E-01
TATDN1	TatD DNase domain containing 1	6.50E-04	5.60E-01
C6orf89	chromosome 6 open reading frame 89	6.51E-04	3.95E-01
STRADA	STE20-related kinase adaptor alpha	6.51E-04	-3.54E-01

BCAR3	breast cancer anti-estrogen resistance 3	6.51E-04	3.90E-01
WDFY2	WD repeat and FYVE domain containing 2	6.52E-04	-6.14E-01
SNRNP40	small nuclear ribonucleoprotein U5 subunit 40	6.52E-04	4.91E-01
IMMP1L	inner mitochondrial membrane peptidase subunit 1	6.53E-04	6.42E-01
TTLL1	tubulin tyrosine ligase like 1	6.54E-04	-3.40E-01
ENDOV	endonuclease V	6.56E-04	-3.81E-01
MICB	MHC class I polypeptide-related sequence B	6.56E-04	8.45E-01
ZNF670	zinc finger protein 670	6.56E-04	3.93E-01
STUB1	STIP1 homology and U-box containing protein 1	6.57E-04	-1.07
ATP2B1	ATPase plasma membrane Ca2+ transporting 1	6.57E-04	5.08E-01
FAM222B	family with sequence similarity 222 member B	6.58E-04	-5.37E-01
HNF4G	hepatocyte nuclear factor 4 gamma	6.58E-04	5.50E-01
G3BP1	G3BP stress granule assembly factor 1	6.58E-04	8.16E-01
NDUFAF6	NADH:ubiquinone oxidoreductase complex assembly factor 6	6.59E-04	5.71E-01
KLHL15	kelch like family member 15	6.59E-04	3.28E-01
TACO1	translational activator of cytochrome c oxidase I	6.60E-04	-8.68E-01

ZCWPW2	zinc finger CW-type and PWWP domain containing 2	6.62E-04	3.53E-01
FBLN1	fibulin 1	6.63E-04	-5.19E-01
ZFP1	ZFP1 zinc finger protein	6.63E-04	4.35E-01
FAH	fumarylacetoacetate hydrolase	6.64E-04	-6.31E-01
SLC4A7	solute carrier family 4 member 7	6.65E-04	8.53E-01
METTL21A	methyltransferase like 21A	6.67E-04	6.02E-01
FAM49B	family with sequence similarity 49 member B	6.67E-04	5.35E-01
KHDC1L	KH domain containing 1 like	6.68E-04	-9.15E-01
FOXO3	forkhead box O3	6.68E-04	-7.73E-01
LACTB2	lactamase beta 2	6.69E-04	1.06
RAB3B	RAB3B, member RAS oncogene family	6.69E-04	3.83E-01
RASSF9	Ras association domain family member 9	6.70E-04	-4.77E-01
ADRA2B	adrenoceptor alpha 2B	6.70E-04	-3.20E-01
CDKN2AIP	CDKN2A interacting protein	6.71E-04	6.71E-01
SMS	spermine synthase	6.71E-04	3.60E-01
NF2	neurofibromin 2	6.72E-04	3.76E-01

SNX33	sorting nexin 33	6.73E-04	-3.46E-01
EIF1AX	eukaryotic translation initiation factor 1A, X-linked	6.74E-04	8.18E-01
POLB	DNA polymerase beta	6.76E-04	8.18E-01
EMC8	ER membrane protein complex subunit 8	6.76E-04	4.31E-01
SNORD104	small nucleolar RNA, C/D box 104	6.77E-04	-1.25
NUS1	NUS1 dehydrodolichyl diphosphate synthase subunit	6.78E-04	-6.47E-01
FAM184B	family with sequence similarity 184 member B	6.78E-04	-4.94E-01
PHF21A	PHD finger protein 21A	6.79E-04	-8.59E-01
TM7SF2	transmembrane 7 superfamily member 2	6.80E-04	-9.51E-01
CYR61	cysteine rich angiogenic inducer 61	6.81E-04	7.18E-01
CNRIP1	cannabinoid receptor interacting protein 1	6.81E-04	-5.61E-01
OSBP	oxysterol binding protein	6.81E-04	-8.57E-01
STAT6	signal transducer and activator of transcription 6	6.83E-04	-7.65E-01
BMP2K	BMP2 inducible kinase	6.85E-04	3.49E-01
CCT2	chaperonin containing TCP1 subunit 2	6.85E-04	6.73E-01

PKP4	plakophilin 4	6.87E-04	8.08E -01
EID2	EP300 interacting inhibitor of differentiation 2	6.87E-04	- 6.61E -01
CDC7	cell division cycle 7	6.87E-04	9.02E -01
CYP51A1	cytochrome P450 family 51 subfamily A member 1	6.87E-04	8.84E -01
ZNF570	zinc finger protein 570	6.87E-04	4.32E -01
IER3IP1	immediate early response 3 interacting protein 1	6.88E-04	5.52E -01
DLEU1	deleted in lymphocytic leukemia 1	6.91E-04	- 7.50E -01
DLX2	distal-less homeobox 2	6.92E-04	- 7.24E -01
FAM160B1	family with sequence similarity 160 member B1	6.93E-04	5.00E -01
P4HB	prolyl 4-hydroxylase subunit beta	6.93E-04	-1.11
ERI2	ERI1 exoribonuclease family member 2	6.94E-04	6.34E -01
CBX2	chromobox 2	6.94E-04	- 9.05E -01
SNX8	sorting nexin 8	6.95E-04	6.64E -01
RPS17	ribosomal protein S17	6.95E-04	- 7.40E -01
POLR2D	RNA polymerase II subunit D	6.96E-04	5.92E -01
N4BP1	NEDD4 binding protein 1	6.96E-04	- 6.39E -01

NFXL1	nuclear transcription factor, X-box binding like 1	6.98E-04	1.05
SOX12	SRY-box 12	7.00E-04	-3.01E-01
BLOC1S2	biogenesis of lysosomal organelles complex 1 subunit 2	7.01E-04	-8.67E-01
FAM196A	family with sequence similarity 196 member A	7.01E-04	-3.47E-01
PDIA5	protein disulfide isomerase family A member 5	7.02E-04	-3.28E-01
CTBS	chitobiase	7.03E-04	-6.22E-01
SDHA	succinate dehydrogenase complex flavoprotein subunit A	7.04E-04	-5.63E-01
EXOSC10	exosome component 10	7.05E-04	5.82E-01
APMAP	adipocyte plasma membrane associated protein	7.05E-04	4.82E-01
NCBP1	nuclear cap binding protein subunit 1	7.08E-04	5.40E-01
ASIC1	acid sensing ion channel subunit 1	7.10E-04	-6.67E-01
CHMP2B	charged multivesicular body protein 2B	7.10E-04	5.45E-01
MICU2	mitochondrial calcium uptake 2	7.11E-04	6.25E-01
PPWD1	peptidylprolyl isomerase domain and WD repeat containing 1	7.12E-04	-7.68E-01
UBE2A	ubiquitin conjugating enzyme E2 A	7.12E-04	6.08E-01

AMDHD2	amidohydrolase domain containing 2	7.15E-04	6.09E-01
TGFB3	transforming growth factor beta 3	7.15E-04	-8.20E-01
NARF	nuclear prelamin A recognition factor	7.16E-04	-1.09
TMEM201	transmembrane protein 201	7.18E-04	3.38E-01
LRSAM1	leucine rich repeat and sterile alpha motif containing 1	7.19E-04	-9.40E-01
ACTR5	ARP5 actin-related protein 5 homolog	7.19E-04	6.45E-01
MCUR1	mitochondrial calcium uniporter regulator 1	7.19E-04	8.88E-01
ONECUT2	one cut homeobox 2	7.19E-04	-5.51E-01
KCNK12	potassium two pore domain channel subfamily K member 12	7.20E-04	-8.77E-01
ART5	ADP-ribosyltransferase 5	7.21E-04	-7.37E-01
PLA2G4A	phospholipase A2 group IVA	7.21E-04	5.31E-01
MRPL11	mitochondrial ribosomal protein L11	7.23E-04	4.56E-01
TMEM106A	transmembrane protein 106A	7.23E-04	-8.84E-01
TRAF5	TNF receptor associated factor 5	7.23E-04	8.92E-01
APOD	apolipoprotein D	7.23E-04	-3.40E-01

ARHGAP4	Rho GTPase activating protein 4	7.24E-04	-7.27E-01
CPEB2	cytoplasmic polyadenylation element binding protein 2	7.25E-04	3.28E-01
SURF1	surfeit 1	7.26E-04	-6.90E-01
GADD45GIP1	GADD45G interacting protein 1	7.26E-04	7.91E-01
BLMH	bleomycin hydrolase	7.29E-04	-5.79E-01
ZNF248	zinc finger protein 248	7.29E-04	-5.33E-01
ARFGAP1	ADP ribosylation factor GTPase activating protein 1	7.30E-04	-8.45E-01
SMCR5	Smith-Magenis syndrome chromosome region, candidate 5 (non-protein coding)	7.30E-04	-6.92E-01
PUM1	pumilio RNA binding family member 1	7.35E-04	-4.18E-01
UBL3	ubiquitin like 3	7.35E-04	-4.56E-01
CWF19L2	CWF19-like 2, cell cycle control (S. pombe)	7.38E-04	7.24E-01
CUL4A	cullin 4A	7.38E-04	-6.72E-01
GDAP1	ganglioside induced differentiation associated protein 1	7.39E-04	9.13E-01
RTCA	RNA 3'-terminal phosphate cyclase	7.40E-04	5.64E-01

ADAMTS20	ADAM metallopeptidase with thrombospondin type 1 motif 20	7.42E-04	3.68E-01
TWF1	twinfilin actin binding protein 1	7.43E-04	1.34
TXNDC12	thioredoxin domain containing 12	7.43E-04	-4.71E-01
NEK11	NIMA related kinase 11	7.43E-04	4.91E-01
KCNIP3	potassium voltage-gated channel interacting protein 3	7.44E-04	-5.29E-01
FAM162A	family with sequence similarity 162 member A	7.46E-04	-4.82E-01
CCND1	cyclin D1	7.47E-04	-1.11
ABAT	4-aminobutyrate aminotransferase	7.48E-04	-8.35E-01
OLMALINC	oligodendrocyte maturation-associated long intergenic non-coding RNA	7.49E-04	-9.22E-01
PRPF3	pre-mRNA processing factor 3	7.50E-04	6.29E-01
TGIF2	TGFB induced factor homeobox 2	7.50E-04	-5.14E-01
RNASEL	ribonuclease L	7.51E-04	-3.79E-01
ASPSCR1	ASPSCR1, UBX domain containing tether for SLC2A4	7.51E-04	-8.51E-01
FEZ1	fasciculation and elongation protein zeta 1	7.51E-04	-4.78E-01
RAP1B	RAP1B, member of RAS oncogene family	7.51E-04	9.24E-01

SENP5	SUMO1/sentrin specific peptidase 5	7.52E-04	-4.58E-01
DEPTOR	DEP domain containing MTOR-interacting protein	7.54E-04	2.95E-01
SS18L1	SS18L1, nBAF chromatin remodeling complex subunit	7.55E-04	-5.62E-01
RPS6KB1	ribosomal protein S6 kinase B1	7.57E-04	-6.77E-01
LONRF1	LON peptidase N-terminal domain and ring finger 1	7.58E-04	1.14
TFDP2	transcription factor Dp-2	7.58E-04	-4.09E-01
CDC20	cell division cycle 20	7.58E-04	1.65
METAP1	methionyl aminopeptidase 1	7.59E-04	7.35E-01
DRAP1	DR1 associated protein 1	7.60E-04	6.19E-01
WDR53	WD repeat domain 53	7.60E-04	5.43E-01
TBC1D7	TBC1 domain family member 7	7.61E-04	5.97E-01
SNRPB	small nuclear ribonucleoprotein polypeptides B and B1	7.61E-04	7.69E-01
B2M	beta-2-microglobulin	7.62E-04	7.05E-01
FOXC2	forkhead box C2	7.66E-04	-4.28E-01
KLHDC2	kelch domain containing 2	7.66E-04	-4.30E-01

PAAF1	proteasomal ATPase associated factor 1	7.67E-04	-5.27E-01
ZNF30	zinc finger protein 30	7.68E-04	4.68E-01
DCLK1	doublecortin like kinase 1	7.70E-04	3.09E-01
STAU2	staufen double-stranded RNA binding protein 2	7.70E-04	7.99E-01
OGFR	opioid growth factor receptor	7.70E-04	4.65E-01
FUCA1	fucosidase, alpha-L- 1, tissue	7.71E-04	-6.64E-01
IPO8	importin 8	7.71E-04	7.24E-01
ISCA1	iron-sulfur cluster assembly 1	7.74E-04	4.88E-01
RBMX2	RNA binding motif protein, X-linked 2	7.74E-04	5.18E-01
AKAP6	A-kinase anchoring protein 6	7.74E-04	3.38E-01
PLEKHF2	pleckstrin homology and FYVE domain containing 2	7.76E-04	7.85E-01
PLAGL1	PLAG1 like zinc finger 1	7.77E-04	-9.60E-01
DUSP18	dual specificity phosphatase 18	7.77E-04	4.00E-01
TCP1	t-complex 1	7.78E-04	9.54E-01
PRKCQ-AS1	PRKCQ antisense RNA 1	7.78E-04	3.56E-01
MEIS2	Meis homeobox 2	7.79E-04	-7.75E-01

MED13	mediator complex subunit 13	7.81E-04	4.28E-01
FAM107B	family with sequence similarity 107 member B	7.81E-04	-4.88E-01
ZNF586	zinc finger protein 586	7.83E-04	6.18E-01
PRSS57	protease, serine 57	7.83E-04	-4.44E-01
EFCAB7	EF-hand calcium binding domain 7	7.84E-04	5.75E-01
TRMT11	tRNA methyltransferase 11 homolog	7.84E-04	6.24E-01
VDAC2	voltage dependent anion channel 2	7.84E-04	7.45E-01
MSH2	mutS homolog 2	7.85E-04	1.18
ME2	malic enzyme 2	7.86E-04	5.64E-01
MRM2	mitochondrial rRNA methyltransferase 2	7.86E-04	8.77E-01
GOLGA2	golgin A2	7.87E-04	-5.58E-01
GINS4	GINS complex subunit 4	7.90E-04	1.05
UQCR10	ubiquinol-cytochrome c reductase, complex III subunit X	7.91E-04	-7.11E-01
CD151	CD151 molecule (Raph blood group)	7.93E-04	6.06E-01
PPTC7	PTC7 protein phosphatase homolog	7.94E-04	-9.40E-01
RASL11A	RAS like family 11 member A	7.94E-04	3.47E-01

RABL2B	RAB, member of RAS oncogene family-like 2B	7.95E-04	-7.41E-01
YY1AP1	YY1 associated protein 1	7.96E-04	-4.48E-01
MMADHC	methylmalonic aciduria and homocystinuria, cblD type	7.97E-04	5.72E-01
RPL7A	ribosomal protein L7a	7.97E-04	-4.09E-01
ZNF789	zinc finger protein 789	7.97E-04	4.04E-01
KIFAP3	kinesin associated protein 3	7.98E-04	3.29E-01
PITRM1	pitrilysin metallopeptidase 1	7.98E-04	6.76E-01
POMGNT2	protein O-linked mannose N-acetylglucosaminyltransferase 2 (beta 1,4-)	7.98E-04	6.38E-01
SDF4	stromal cell derived factor 4	7.99E-04	-4.73E-01
ZDHHC8P1	zinc finger DHHC-type containing 8 pseudogene 1	8.01E-04	-5.81E-01
PROSER1	proline and serine rich 1	8.03E-04	9.59E-01
KLHL13	kelch like family member 13	8.03E-04	4.67E-01
RPS27A	ribosomal protein S27a	8.03E-04	-4.33E-01
CES2	carboxylesterase 2	8.03E-04	-7.31E-01
GRIN1	glutamate ionotropic receptor NMDA type subunit 1	8.05E-04	5.96E-01

SLC38A10	solute carrier family 38 member 10	8.06E-04	-7.20E-01
RNF217	ring finger protein 217	8.06E-04	3.17E-01
ICAM4	intercellular adhesion molecule 4 (Landsteiner-Wiener blood group)	8.07E-04	-4.59E-01
TMEM69	transmembrane protein 69	8.07E-04	-1.11
RUFY2	RUN and FYVE domain containing 2	8.10E-04	4.34E-01
NTF4	neurotrophin 4	8.11E-04	-2.87E-01
CAMKMT	calmodulin-lysine N-methyltransferase	8.12E-04	-8.59E-01
KCNJ18	potassium voltage-gated channel subfamily J member 18	8.12E-04	-9.30E-01
ZP3	zona pellucida glycoprotein 3 (sperm receptor)	8.12E-04	5.95E-01
MAGOHB	mago homolog B, exon junction complex core component	8.15E-04	3.64E-01
KHNYN	KH and NYN domain containing	8.16E-04	-6.36E-01
MUT	methylmalonyl-CoA mutase	8.16E-04	3.70E-01
NDUFAF5	NADH:ubiquinone oxidoreductase complex assembly factor 5	8.17E-04	6.69E-01
HOXB8	homeobox B8	8.17E-04	1.08
PEX13	peroxisomal biogenesis factor 13	8.18E-04	6.77E-01
FAM208A	family with sequence similarity 208 member A	8.19E-04	-4.11E-01

FAM127B	family with sequence similarity 127 member B	8.19E-04	-1.02
MANF	mesencephalic astrocyte derived neurotrophic factor	8.20E-04	-7.85E-01
PPM1A	protein phosphatase, Mg2+/Mn2+ dependent 1A	8.20E-04	6.04E-01
ARHGEF39	Rho guanine nucleotide exchange factor 39	8.21E-04	9.08E-01
PPP1R18	protein phosphatase 1 regulatory subunit 18	8.23E-04	-8.23E-01
TACC2	transforming acidic coiled-coil containing protein 2	8.23E-04	6.72E-01
SNHG1	small nucleolar RNA host gene 1	8.24E-04	7.61E-01
KANK1	KN motif and ankyrin repeat domains 1	8.25E-04	1.26
SPTBN4	spectrin beta, non-erythrocytic 4	8.26E-04	-4.44E-01
AXIN2	axin 2	8.27E-04	-7.17E-01
UBE2D4	ubiquitin conjugating enzyme E2 D4 (putative)	8.28E-04	8.40E-01
ADAMTSL4	ADAMTS like 4	8.29E-04	-4.17E-01
MIEF1	mitochondrial elongation factor 1	8.31E-04	-7.64E-01
UTP11	UTP11, small subunit processome component homolog (S. cerevisiae)	8.31E-04	7.82E-01
ROM1	retinal outer segment membrane protein 1	8.32E-04	-5.61E-01

DCAF10	DDB1 and CUL4 associated factor 10	8.32E-04	-4.80E-01
SMC4	structural maintenance of chromosomes 4	8.34E-04	6.59E-01
MCMBP	minichromosome maintenance complex binding protein	8.35E-04	5.41E-01
PGAP1	post-GPI attachment to proteins 1	8.36E-04	-5.43E-01
STAT1	signal transducer and activator of transcription 1	8.37E-04	5.50E-01
MGME1	mitochondrial genome maintenance exonuclease 1	8.37E-04	-3.07E-01
RFPL4B	ret finger protein like 4B	8.37E-04	-4.82E-01
WASF3	WAS protein family member 3	8.38E-04	-8.44E-01
OCIAD1	OCIA domain containing 1	8.38E-04	-6.81E-01
NGLY1	N-glycanase 1	8.41E-04	5.71E-01
AES	amino-terminal enhancer of split	8.41E-04	-5.71E-01
RASIP1	Ras interacting protein 1	8.42E-04	-5.31E-01
FOXS1	forkhead box S1	8.43E-04	-4.71E-01
AGO3	argonaute 3, RISC catalytic component	8.43E-04	4.11E-01

WDHD1	WD repeat and HMG-box DNA binding protein 1	8.45E-04	3.71E-01
STK25	serine/threonine kinase 25	8.46E-04	-5.06E-01
C9orf72	chromosome 9 open reading frame 72	8.46E-04	9.87E-01
C11orf31	chromosome 11 open reading frame 31	8.47E-04	-4.33E-01
MGP	matrix Gla protein	8.47E-04	-1.07
FYCO1	FYVE and coiled-coil domain containing 1	8.48E-04	8.42E-01
ZFP64	ZFP64 zinc finger protein	8.49E-04	-3.76E-01
RNFT1	ring finger protein, transmembrane 1	8.49E-04	-9.44E-01
PPP2R3B	protein phosphatase 2 regulatory subunit B"beta	8.50E-04	-3.90E-01
WLS	wntless Wnt ligand secretion mediator	8.50E-04	4.63E-01
GIN1	gypsy retrotransposon integrase 1	8.51E-04	3.78E-01
PFAS	phosphoribosylformylglycinamidine synthase	8.51E-04	8.21E-01
BEND7	BEN domain containing 7	8.52E-04	3.00E-01
NFKB2	nuclear factor kappa B subunit 2	8.54E-04	5.07E-01
WARS	tryptophanyl-tRNA synthetase	8.54E-04	5.78E-01
MARVELD3	MARVEL domain containing 3	8.54E-04	4.05E-01

SNTB2	syntrophin beta 2	8.55E-04	-7.28E-01
FAM213B	family with sequence similarity 213 member B	8.57E-04	4.13E-01
DKC1	dyskerin pseudouridine synthase 1	8.59E-04	8.01E-01
SETDB2	SET domain bifurcated 2	8.61E-04	-4.39E-01
RRP9	ribosomal RNA processing 9, small subunit (SSU) processome component, homolog (yeast)	8.61E-04	6.11E-01
SNCB	synuclein beta	8.61E-04	-6.72E-01
SGCB	sarcoglycan beta	8.61E-04	5.43E-01
NDUFA5	NADH:ubiquinone oxidoreductase subunit A5	8.63E-04	8.30E-01
CNTNAP1	contactin associated protein 1	8.65E-04	-9.28E-01
GDF11	growth differentiation factor 11	8.65E-04	-4.47E-01
NRSN2	neurensin 2	8.65E-04	-6.99E-01
DNAJC30	DnaJ heat shock protein family (Hsp40) member C30	8.66E-04	-3.10E-01
PBX3	PBX homeobox 3	8.67E-04	6.78E-01
L3MBTL3	l(3)mbt-like 3 (Drosophila)	8.67E-04	-4.28E-01
PHC1	polyhomeotic homolog 1	8.68E-04	-1.25

CLCC1	chloride channel CLIC like 1	8.69E-04	3.33E-01
IFT140	intraflagellar transport 140	8.69E-04	-5.02E-01
SRP54	signal recognition particle 54	8.70E-04	3.64E-01
TNFRSF10B	TNF receptor superfamily member 10b	8.70E-04	-9.51E-01
CRTAP	cartilage associated protein	8.71E-04	-1.48
PLK2	polo like kinase 2	8.72E-04	-4.72E-01
COQ8B	coenzyme Q8B	8.72E-04	-7.27E-01
PSEN2	presenilin 2	8.73E-04	-6.62E-01
CLTA	clathrin light chain A	8.73E-04	-6.20E-01
POLH	DNA polymerase eta	8.74E-04	-5.44E-01
WBP11	WW domain binding protein 11	8.75E-04	1.09
RDH14	retinol dehydrogenase 14 (all-trans/9-cis/11-cis)	8.76E-04	-7.12E-01
RINT1	RAD50 interactor 1	8.76E-04	9.07E-01
MTAP	methylthioadenosine phosphorylase	8.78E-04	1.09
CALY	calcyon neuron specific vesicular protein	8.78E-04	-6.67E-01

TRIM5	tripartite motif containing 5	8.78E-04	-3.49E-01
DTWD2	DTW domain containing 2	8.79E-04	4.31E-01
OMA1	OMA1 zinc metallopeptidase	8.79E-04	5.22E-01
SPINT1	serine peptidase inhibitor, Kunitz type 1	8.80E-04	2.79E-01
GRSF1	G-rich RNA sequence binding factor 1	8.81E-04	5.04E-01
HNRNPU	heterogeneous nuclear ribonucleoprotein U	8.81E-04	-3.68E-01
WBP11	WW domain binding protein 11	8.82E-04	1.18
PLD6	phospholipase D family member 6	8.85E-04	-4.19E-01
CENPO	centromere protein O	8.85E-04	4.32E-01
RPL35A	ribosomal protein L35a	8.87E-04	-7.04E-01
TBL1XR1	transducin (beta)-like 1 X-linked receptor 1	8.87E-04	5.30E-01
ATP2C1	ATPase secretory pathway Ca2+ transporting 1	8.89E-04	6.95E-01
C1GALT1	core 1 synthase, glycoprotein-N-acetylgalactosamine 3-beta-galactosyltransferase 1	8.89E-04	7.33E-01
MORN4	MORN repeat containing 4	8.90E-04	5.05E-01
FBXW2	F-box and WD repeat domain containing 2	8.92E-04	5.22E-01
RAD17	RAD17 checkpoint clamp loader component	8.92E-04	-3.81E-01

STAG1	stromal antigen 1	8.92E-04	6.51E-01
HSPA1L	heat shock protein family A (Hsp70) member 1 like	8.94E-04	3.75E-01
TRIM26	tripartite motif containing 26	8.94E-04	-3.82E-01
PRSS12	protease, serine 12	8.94E-04	-4.16E-01
UHRF1BP1L	UHRF1 binding protein 1 like	8.95E-04	8.14E-01
CEP152	centrosomal protein 152	8.95E-04	7.47E-01
SCAND2P	SCAN domain containing 2 pseudogene	8.96E-04	-5.36E-01
DARS	aspartyl-tRNA synthetase	8.96E-04	4.40E-01
PRPF18	pre-mRNA processing factor 18	8.96E-04	6.00E-01
NME2	NME/NM23 nucleoside diphosphate kinase 2	8.97E-04	6.04E-01
C19orf66	chromosome 19 open reading frame 66	8.98E-04	-5.33E-01
MIOS	meiosis regulator for oocyte development	8.99E-04	7.07E-01
SERINC3	serine incorporator 3	8.99E-04	-5.04E-01
PRPF38A	pre-mRNA processing factor 38A	8.99E-04	6.39E-01
KCTD10	potassium channel tetramerization domain containing 10	8.99E-04	-4.59E-01

SFSWAP	splicing factor SWAP homolog	8.99E-04	-<br>4.26E<br>-01
POLR3G	RNA polymerase III subunit G	9.00E-04	7.89E<br>-01
MRAP2	melanocortin 2 receptor accessory protein 2	9.00E-04	3.19E<br>-01
BZW1	basic leucine zipper and W2 domains 1	9.03E-04	1.25
SEMA4F	ssemaphorin 4F	9.04E-04	5.80E<br>-01
KIF5B	kinesin family member 5B	9.04E-04	1.06
SLC25A24	solute carrier family 25 member 24	9.04E-04	8.91E<br>-01
CBS	cystathionine-beta-synthase	9.05E-04	-<br>8.07E<br>-01
DSG2	desmoglein 2	9.06E-04	5.96E<br>-01
NICN1	nicolin 1	9.07E-04	-<br>8.86E<br>-01
DCBLD2	discoidin, CUB and LCCL domain containing 2	9.07E-04	-<br>3.92E<br>-01
FAM13B	family with sequence similarity 13 member B	9.08E-04	-<br>5.96E<br>-01
GRB2	growth factor receptor bound protein 2	9.08E-04	-<br>7.99E<br>-01
PLAG1	PLAG1 zinc finger	9.10E-04	-<br>7.28E<br>-01
FAM86C1	family with sequence similarity 86 member C1	9.10E-04	6.13E<br>-01

DGAT1	diacylglycerol O-acyltransferase 1	9.10E-04	-5.32E-01
AHNAK2	AHNAK nucleoprotein 2	9.11E-04	-7.20E-01
CEP135	centrosomal protein 135	9.12E-04	8.78E-01
MSTO1	misato 1, mitochondrial distribution and morphology regulator	9.12E-04	7.17E-01
PRKCSH	protein kinase C substrate 80K-H	9.15E-04	-5.42E-01
SNRNP27	small nuclear ribonucleoprotein U4/U6.U5 subunit 27	9.19E-04	4.70E-01
PLEKHB1	pleckstrin homology domain containing B1	9.20E-04	-3.55E-01
ATG12	autophagy related 12	9.23E-04	-5.08E-01
DOCK11	dedicator of cytokinesis 11	9.23E-04	4.79E-01
LOC286437	uncharacterized LOC286437	9.25E-04	-4.00E-01
ZDBF2	zinc finger DBF-type containing 2	9.25E-04	3.98E-01
NDUFAF7	NADH:ubiquinone oxidoreductase complex assembly factor 7	9.25E-04	-9.72E-01
CATIP	ciliogenesis associated TTC17 interacting protein	9.25E-04	-8.01E-01
ISPD	isoprenoid synthase domain containing	9.25E-04	3.15E-01
FAM174B	family with sequence similarity 174 member B	9.25E-04	5.02E-01

GABARAPL1	GABA type A receptor associated protein like 1	9.26E-04	-3.58E-01
LDOC1	leucine zipper down-regulated in cancer 1	9.28E-04	5.70E-01
LSS	lanosterol synthase (2,3-oxidosqualene-lanosterol cyclase)	9.29E-04	-4.34E-01
GPATCH4	G-patch domain containing 4	9.32E-04	7.35E-01
TGFBR3	transforming growth factor beta receptor 3	9.32E-04	-1.07
KIAA0319L	KIAA0319 like	9.33E-04	-5.00E-01
PLEKHA7	pleckstrin homology domain containing A7	9.34E-04	3.65E-01
LINC00294	long intergenic non-protein coding RNA 294	9.35E-04	-7.22E-01
DCAF4	DDB1 and CUL4 associated factor 4	9.38E-04	4.03E-01
MT1A	metallothionein 1A	9.39E-04	1.29
GRAMD4	GRAM domain containing 4	9.39E-04	-8.63E-01
ARL17B	ADP ribosylation factor like GTPase 17B	9.40E-04	-5.47E-01
ATP2A2	ATPase sarcoplasmic/endoplasmic reticulum Ca2+ transporting 2	9.40E-04	7.56E-01
NPAS1	neuronal PAS domain protein 1	9.40E-04	-9.43E-01
PDCD5	programmed cell death 5	9.42E-04	1.05
ZNF800	zinc finger protein 800	9.45E-04	3.62E-01

TUSC2	tumor suppressor candidate 2	9.46E-04	-5.32E-01
RBPMS2	RNA binding protein with multiple splicing 2	9.48E-04	-6.58E-01
GNL3	G protein nucleolar 3	9.48E-04	3.20E-01
ZNF226	zinc finger protein 226	9.49E-04	-6.09E-01
HSP90AA1	heat shock protein 90 alpha family class A member 1	9.50E-04	6.94E-01
ERCC3	ERCC excision repair 3, TFIIH core complex helicase subunit	9.50E-04	7.71E-01
PEX14	peroxisomal biogenesis factor 14	9.51E-04	-4.43E-01
RGS17	regulator of G-protein signaling 17	9.51E-04	-3.18E-01
PHF20L1	PHD finger protein 20-like 1	9.51E-04	4.27E-01
SNORD4B	small nucleolar RNA, C/D box 4B	9.55E-04	-5.31E-01
TOR1B	torsin family 1 member B	9.55E-04	4.57E-01
TOR1A	torsin family 1 member A	9.57E-04	-5.19E-01
ADCK2	aarF domain containing kinase 2	9.59E-04	8.35E-01
RAB24	RAB24, member RAS oncogene family	9.60E-04	-5.61E-01
CMAS	cytidine monophosphate N-acetylneuraminic acid synthetase	9.60E-04	6.52E-01

MPHOSPH9	M-phase phosphoprotein 9	9.60E-04	6.44E -01
TIGD7	tigger transposable element derived 7	9.61E-04	3.29E -01
TNIP1	TNFAIP3 interacting protein 1	9.62E-04	- 5.47E -01
PAGR1	PAXIP1 associated glutamate rich protein 1	9.63E-04	4.54E -01
SLAIN1	SLAIN motif family member 1	9.64E-04	2.95E -01
TRAF3IP2	TRAF3 interacting protein 2	9.66E-04	- 6.75E -01
HOXB13	homeobox B13	9.66E-04	- 6.59E -01
ZNF33B	zinc finger protein 33B	9.66E-04	- 6.51E -01
COL4A6	collagen type IV alpha 6 chain	9.68E-04	- 9.91E -01
LCAT	lecithin-cholesterol acyltransferase	9.70E-04	- 7.77E -01
GCNT1	glucosaminyl (N-acetyl) transferase 1, core 2	9.71E-04	3.60E -01
SSFA2	sperm specific antigen 2	9.72E-04	5.88E -01
SH3BGR	SH3 domain binding glutamate rich protein	9.74E-04	-1.24
DDX60	DEXD/H-box helicase 60	9.75E-04	6.27E -01
LIN54	lin-54 DREAM MuvB core complex component	9.76E-04	3.90E -01
TBCCD1	TBCC domain containing 1	9.77E-04	4.44E -01

PRMT2	protein arginine methyltransferase 2	9.78E-04	-6.36E-01
CCDC15	coiled-coil domain containing 15	9.78E-04	4.11E-01
BNIP3L	BCL2 interacting protein 3 like	9.81E-04	-7.50E-01
NOL8	nucleolar protein 8	9.81E-04	7.55E-01
PYGO1	pygopus family PHD finger 1	9.82E-04	-5.71E-01
EXT1	exostosin glycosyltransferase 1	9.83E-04	-5.70E-01
EIF3L	eukaryotic translation initiation factor 3 subunit L	9.83E-04	-4.47E-01
CDK18	cyclin dependent kinase 18	9.83E-04	3.21E-01
MORF4L1	mortality factor 4 like 1	9.84E-04	5.37E-01
COG2	component of oligomeric golgi complex 2	9.85E-04	-5.48E-01
TRIM33	tripartite motif containing 33	9.86E-04	3.97E-01
SSRP1	structure specific recognition protein 1	9.86E-04	6.12E-01
RAI1	retinoic acid induced 1	9.88E-04	-7.25E-01
PARVA	parvin alpha	9.88E-04	3.90E-01
BRINP1	BMP/retinoic acid inducible neural specific 1	9.88E-04	3.02E-01

ITGB1BP1	integrin subunit beta 1 binding protein 1	9.89E-04	6.29E -01
LCE1B	late cornified envelope 1B	9.91E-04	- 5.38E -01
NSD1	nuclear receptor binding SET domain protein 1	9.91E-04	3.08E -01
GSTK1	glutathione S-transferase kappa 1	9.91E-04	- 6.62E -01
SNW1	SNW domain containing 1	9.93E-04	4.29E -01
DLEU2L	deleted in lymphocytic leukemia 2-like	9.93E-04	7.11E -01
TRNT1	tRNA nucleotidyl transferase 1	9.96E-04	4.89E -01
TPGS1	tubulin polyglutamylase complex subunit 1	9.96E-04	- 3.09E -01
ZSCAN5A	zinc finger and SCAN domain containing 5A	9.98E-04	3.34E -01
H2AFV	H2A histone family member V	9.99E-04	3.75E -01
SLC43A2	solute carrier family 43 member 2	1.00E-03	-1.09
ZNF442	zinc finger protein 442	1.00E-03	- 4.40E -01
EEF1G	eukaryotic translation elongation factor 1 gamma	1.00E-03	-1.01
NOC3L	NOC3 like DNA replication regulator	1.00E-03	6.49E -01
SNAPC1	small nuclear RNA activating complex polypeptide 1	1.00E-03	- 4.39E -01
RGAG4	retrotransposon gag domain containing 4	1.01E-03	- 7.41E -01

RAB24	RAB24, member RAS oncogene family	1.01E-03	-7.22E-01
ORC2	origin recognition complex subunit 2	1.01E-03	6.39E-01
NT5C3A	5'-nucleotidase, cytosolic IIIA	1.01E-03	5.04E-01
HSP90AA1	heat shock protein 90 alpha family class A member 1	1.01E-03	1.29
CLTB	clathrin light chain B	1.01E-03	7.21E-01
CCT6A	chaperonin containing TCP1 subunit 6A	1.01E-03	7.68E-01
FAM200B	family with sequence similarity 200 member B	1.01E-03	-4.44E-01
TP53BP2	tumor protein p53 binding protein 2	1.01E-03	-8.03E-01
FOXD2-AS1	FOXD2 antisense RNA 1 (head to head)	1.01E-03	3.75E-01
ZNF443	zinc finger protein 443	1.01E-03	6.65E-01
ZNF330	zinc finger protein 330	1.01E-03	4.24E-01
SNCA	synuclein alpha	1.02E-03	-6.58E-01
CCNG2	cyclin G2	1.02E-03	-5.59E-01
PABPC5	poly(A) binding protein cytoplasmic 5	1.02E-03	-3.11E-01
PPM1M	protein phosphatase, Mg2+/Mn2+ dependent 1M	1.02E-03	-8.05E-01

TNFRSF10D	TNF receptor superfamily member 10d	1.02E-03	4.03E-01
PIAS2	protein inhibitor of activated STAT 2	1.02E-03	4.65E-01
METTL18	methyltransferase like 18	1.02E-03	6.61E-01
CHD2	chromodomain helicase DNA binding protein 2	1.02E-03	4.81E-01
KBTBD7	kelch repeat and BTB domain containing 7	1.02E-03	-5.07E-01
ATG16L1	autophagy related 16 like 1	1.02E-03	5.18E-01
FBN2	fibrillin 2	1.02E-03	8.82E-01
PRSS23	protease, serine 23	1.02E-03	-3.30E-01
C2orf68	chromosome 2 open reading frame 68	1.03E-03	-5.05E-01
BCL2L12	BCL2 like 12	1.03E-03	1.04
ZNF101	zinc finger protein 101	1.03E-03	3.68E-01
PPRC1	peroxisome proliferator-activated receptor gamma, coactivator-related 1	1.03E-03	5.60E-01
ADGRB1	adhesion G protein-coupled receptor B1	1.03E-03	-3.10E-01
BANF1	barrier to autointegration factor 1	1.03E-03	8.00E-01
HEXA	hexosaminidase subunit alpha	1.03E-03	-4.46E-01
CD99L2	CD99 molecule like 2	1.03E-03	-3.40E-01

TMEM175	transmembrane protein 175	1.03E-03	-7.69E-01
DPP7	dipeptidyl peptidase 7	1.03E-03	9.86E-01
TSC22D1	TSC22 domain family member 1	1.03E-03	-7.78E-01
CDC42EP4	CDC42 effector protein 4	1.03E-03	-6.84E-01
NHLRC3	NHL repeat containing 3	1.03E-03	3.66E-01
SARM1	sterile alpha and TIR motif containing 1	1.04E-03	-4.03E-01
SYT17	synaptotagmin 17	1.04E-03	-4.00E-01
HAX1	HCLS1 associated protein X-1	1.04E-03	6.80E-01
LOX	lysyl oxidase	1.04E-03	6.38E-01
SKP1	S-phase kinase-associated protein 1	1.04E-03	6.23E-01
GTF2H2	general transcription factor IIH subunit 2	1.04E-03	8.86E-01
MAT2A	methionine adenosyltransferase 2A	1.04E-03	6.91E-01
TSC1	tuberous sclerosis 1	1.04E-03	-7.02E-01
CLDN16	claudin 16	1.04E-03	3.74E-01
CRLF3	cytokine receptor like factor 3	1.05E-03	5.69E-01

PIK3CD	phosphatidylinositol-4,5-bisphosphate 3-kinase catalytic subunit delta	1.05E-03	-4.99E-01
ABCB6	ATP binding cassette subfamily B member 6 (Langereis blood group)	1.05E-03	-7.95E-01
INPP5J	inositol polyphosphate-5-phosphatase J	1.05E-03	3.40E-01
MOB4	MOB family member 4, phocein	1.05E-03	3.96E-01
XXYLT1	xyloside xylosyltransferase 1	1.05E-03	-9.08E-01
H3F3C	H3 histone, family 3C	1.05E-03	-8.16E-01
PGM2L1	phosphoglucomutase 2 like 1	1.05E-03	5.01E-01
EIF4G2	eukaryotic translation initiation factor 4 gamma 2	1.05E-03	1.49
MLST8	MTOR associated protein, LST8 homolog	1.06E-03	-3.77E-01
PPP1R15A	protein phosphatase 1 regulatory subunit 15A	1.06E-03	6.31E-01
VPS50	VPS50, EARP/GARPII complex subunit	1.06E-03	6.89E-01
LRRC42	leucine rich repeat containing 42	1.06E-03	3.31E-01
ADAMTS7	ADAM metallopeptidase with thrombospondin type 1 motif 7	1.06E-03	-8.30E-01
TTC17	tetratricopeptide repeat domain 17	1.06E-03	-7.39E-01
CMIP	c-Maf inducing protein	1.06E-03	-5.03E-01

RWDD1	RWD domain containing 1	1.06E-03	4.04E-01
MEGF8	multiple EGF like domains 8	1.06E-03	-6.72E-01
WDR81	WD repeat domain 81	1.06E-03	-8.13E-01
DIABLO	diablo IAP-binding mitochondrial protein	1.06E-03	-3.02E-01
ABHD15	abhydrolase domain containing 15	1.06E-03	-9.91E-01
SUMO1P1	SUMO1 pseudogene 1	1.07E-03	5.85E-01
MERTK	MER proto-oncogene, tyrosine kinase	1.07E-03	-9.46E-01
PBDC1	polysaccharide biosynthesis domain containing 1	1.07E-03	4.94E-01
CSNK1G2	casein kinase 1 gamma 2	1.07E-03	-6.78E-01
ZNF682	zinc finger protein 682	1.07E-03	6.37E-01
SIVA1	SIVA1 apoptosis inducing factor	1.07E-03	1.03
PSEN2	presenilin 2	1.07E-03	-3.86E-01
TMEM79	transmembrane protein 79	1.07E-03	8.24E-01
LOC729970	hCG2028352-like	1.07E-03	-3.02E-01
NCAM2	neural cell adhesion molecule 2	1.07E-03	-9.03E-01

NUP62	nucleoporin 62	1.07E-03	4.18E -01
SEPP1	selenoprotein P, plasma, 1	1.08E-03	-8.13E -01
POLR1D	RNA polymerase I subunit D	1.08E-03	-3.27E -01
ZNF579	zinc finger protein 579	1.08E-03	-4.88E -01
MT1X	metallothionein 1X	1.08E-03	1.13
MYL6	myosin light chain 6	1.09E-03	4.37E -01
MBLAC2	metallo-beta-lactamase domain containing 2	1.09E-03	-5.36E -01
HAGHL	hydroxyacylglutathione hydrolase-like	1.09E-03	-7.14E -01
PAQR4	progestin and adipoQ receptor family member 4	1.09E-03	3.43E -01
ASB8	ankyrin repeat and SOCS box containing 8	1.09E-03	-4.56E -01
CPT2	carnitine palmitoyltransferase 2	1.09E-03	-5.30E -01
ELMOD2	ELMO domain containing 2	1.09E-03	4.64E -01
SMG5	SMG5, nonsense mediated mRNA decay factor	1.09E-03	6.97E -01
PCDHB2	protocadherin beta 2	1.09E-03	-5.10E -01
CRELD2	cysteine rich with EGF like domains 2	1.10E-03	-1.18

ASNS	asparagine synthetase (glutamine-hydrolyzing)	1.10E-03	5.77E-01
AADAT	aminoadipate aminotransferase	1.10E-03	5.99E-01
ATP6V0A2	ATPase H+ transporting V0 subunit a2	1.10E-03	3.99E-01
SCAMP1	secretory carrier membrane protein 1	1.10E-03	-3.18E-01
ERGIC1	endoplasmic reticulum-golgi intermediate compartment 1	1.10E-03	-5.44E-01
TYSND1	trypsin domain containing 1	1.10E-03	-9.08E-01
ZNF675	zinc finger protein 675	1.10E-03	6.56E-01
MRPL45	mitochondrial ribosomal protein L45	1.10E-03	-8.87E-01
SEH1L	SEH1 like nucleoporin	1.11E-03	1.27
VGF	VGF nerve growth factor inducible	1.11E-03	-6.87E-01
TRIP6	thyroid hormone receptor interactor 6	1.11E-03	-5.23E-01
CDC23	cell division cycle 23	1.12E-03	7.14E-01
RAD51D	RAD51 paralog D	1.12E-03	3.23E-01
GPRASP2	G protein-coupled receptor associated sorting protein 2	1.12E-03	1.28
RFX5	regulatory factor X5	1.12E-03	-1
HIVEP2	human immunodeficiency virus type I enhancer binding protein 2	1.12E-03	-3.72E-01

SMIM14	small integral membrane protein 14	1.12E-03	-5.66E-01
ZC3HAV1	zinc finger CCCH-type containing, antiviral 1	1.12E-03	9.40E-01
MAP3K11	mitogen-activated protein kinase kinase kinase 11	1.12E-03	5.87E-01
SNX31	sorting nexin 31	1.13E-03	-4.77E-01
ZNF737	zinc finger protein 737	1.13E-03	4.28E-01
RAD51B	RAD51 paralog B	1.13E-03	3.47E-01
CUEDC2	CUE domain containing 2	1.13E-03	-3.26E-01
N4BP2L2	NEDD4 binding protein 2 like 2	1.13E-03	-3.27E-01
FOLR1	folate receptor 1	1.13E-03	-6.78E-01
DCAF17	DDB1 and CUL4 associated factor 17	1.13E-03	5.00E-01
GAS2L1	growth arrest specific 2 like 1	1.13E-03	-5.94E-01
BNIP2	BCL2 interacting protein 2	1.13E-03	6.43E-01
GOLT1B	golgi transport 1B	1.13E-03	8.69E-01
CDCA4	cell division cycle associated 4	1.13E-03	7.07E-01
VPS50	VPS50, EARP/GARPII complex subunit	1.14E-03	3.25E-01

JMJD7-PLA2G4B	JMJD7-PLA2G4B readthrough	1.14E-03	-3.97E-01
ATP6V1A	ATPase H+ transporting V1 subunit A	1.14E-03	-7.15E-01
THAP5	THAP domain containing 5	1.14E-03	7.71E-01
NFIA	nuclear factor I A	1.14E-03	3.21E-01
DMRT2	doublesex and mab-3 related transcription factor 2	1.14E-03	3.37E-01
PPIL4	peptidylprolyl isomerase like 4	1.14E-03	3.34E-01
ZNF614	zinc finger protein 614	1.14E-03	1.11
FAM86HP	family with sequence similarity 86, member A pseudogene	1.14E-03	4.55E-01
CFAP206	cilia and flagella associated protein 206	1.15E-03	2.90E-01
XRCC6	X-ray repair cross complementing 6	1.15E-03	4.54E-01
SLC25A23	solute carrier family 25 member 23	1.15E-03	-5.07E-01
C10orf10	chromosome 10 open reading frame 10	1.15E-03	-2.86E-01
TMEM129	transmembrane protein 129	1.15E-03	-4.00E-01
TRAPPC13	trafficking protein particle complex 13	1.15E-03	-5.04E-01
F2RL1	F2R like trypsin receptor 1	1.16E-03	3.29E-01
GCLC	glutamate-cysteine ligase catalytic subunit	1.16E-03	4.27E-01

GGACT	gamma-glutamylamine cyclotransferase	1.16E-03	-5.78E-01
RARS2	arginyl-tRNA synthetase 2, mitochondrial	1.16E-03	-4.29E-01
KLF13	Kruppel like factor 13	1.16E-03	6.58E-01
UST	uronyl 2-sulfotransferase	1.16E-03	-1.05
C1RL-AS1	C1RL antisense RNA 1	1.16E-03	-4.54E-01
DCAF13P3	DDB1 and CUL4 associated factor 13 pseudogene 3	1.16E-03	-5.35E-01
GPAT3	glycerol-3-phosphate acyltransferase 3	1.16E-03	5.33E-01
PIGA	phosphatidylinositol glycan anchor biosynthesis class A	1.16E-03	3.93E-01
SUPT4H1	SPT4 homolog, DSIF elongation factor subunit	1.16E-03	-6.45E-01
ZSWIM2	zinc finger SWIM-type containing 2	1.17E-03	4.71E-01
SLC35E1	solute carrier family 35 member E1	1.17E-03	-8.61E-01
DUS4L	dihydrouridine synthase 4 like	1.17E-03	5.12E-01
ACTR1B	ARP1 actin-related protein 1 homolog B, centractin beta	1.17E-03	-4.17E-01
CASP8AP2	caspase 8 associated protein 2	1.17E-03	4.72E-01
EZH2	enhancer of zeste 2 polycomb repressive complex 2 subunit	1.17E-03	9.49E-01

HOXC6	homeobox C6	1.17E-03	-9.31E-01
NR2C2AP	nuclear receptor 2C2 associated protein	1.17E-03	8.18E-01
AK1	adenylate kinase 1	1.17E-03	-1.01
CCDC90B	coiled-coil domain containing 90B	1.17E-03	-6.06E-01
GALNT12	polypeptide N-acetylgalactosaminyltransferase 12	1.18E-03	-6.30E-01
BBC3	BCL2 binding component 3	1.18E-03	-4.76E-01
PKP2	plakophilin 2	1.18E-03	6.64E-01
RNF41	ring finger protein 41	1.18E-03	-3.70E-01
RBBP6	RB binding protein 6, ubiquitin ligase	1.19E-03	-3.08E-01
TBC1D16	TBC1 domain family member 16	1.19E-03	-3.90E-01
SDCBP	syndecan binding protein	1.19E-03	9.93E-01
HOOK2	hook microtubule tethering protein 2	1.19E-03	3.39E-01
LEPR	leptin receptor	1.19E-03	4.07E-01
FBXW5	F-box and WD repeat domain containing 5	1.19E-03	-6.34E-01
PXYLP1	2-phosphoxylose phosphatase 1	1.19E-03	4.88E-01

IRAK2	interleukin 1 receptor associated kinase 2	1.19E-03	-5.60E-01
MLF2	myeloid leukemia factor 2	1.19E-03	-8.18E-01
PSMB4	proteasome subunit beta 4	1.19E-03	4.77E-01
ZNF680	zinc finger protein 680	1.20E-03	6.09E-01
PDK3	pyruvate dehydrogenase kinase 3	1.20E-03	-6.56E-01
CCDC189	coiled-coil domain containing 189	1.20E-03	8.43E-01
PFKFB3	6-phosphofructo-2-kinase/fructose-2,6-biphosphatase 3	1.20E-03	-4.80E-01
TSC22D1	TSC22 domain family member 1	1.21E-03	-1.47
EXOSC3	exosome component 3	1.21E-03	5.24E-01
ZNF347	zinc finger protein 347	1.21E-03	3.17E-01
FOLH1	folate hydrolase (prostate-specific membrane antigen) 1	1.21E-03	2.99E-01
SERTAD2	SERTA domain containing 2	1.21E-03	-7.57E-01
ZNF597	zinc finger protein 597	1.21E-03	3.83E-01
RCN1	reticulocalbin 1	1.21E-03	-3.30E-01
ACADSB	acyl-CoA dehydrogenase, short/branched chain	1.22E-03	3.85E-01
HIST1H4C	histone cluster 1, H4c	1.22E-03	2.63

SNCB	synuclein beta	1.22E-03	-6.52E-01
AP3B1	adaptor related protein complex 3 beta 1 subunit	1.22E-03	-1.02
SPAG1	sperm associated antigen 1	1.22E-03	4.27E-01
ARHGEF7	Rho guanine nucleotide exchange factor 7	1.22E-03	-7.48E-01
PPP1CB	protein phosphatase 1 catalytic subunit beta	1.22E-03	-1.15
SPATA18	spermatogenesis associated 18	1.22E-03	-8.51E-01
PSMA3	proteasome subunit alpha 3	1.22E-03	5.87E-01
ZNF213	zinc finger protein 213	1.23E-03	3.96E-01
YIPF1	Yip1 domain family member 1	1.23E-03	-4.44E-01
RAD51	RAD51 recombinase	1.23E-03	-5.45E-01
MGRN1	mahogunin ring finger 1	1.23E-03	-8.18E-01
LCLAT1	lysocardiolipin acyltransferase 1	1.23E-03	9.64E-01
CTNNBIP1	catenin beta interacting protein 1	1.23E-03	-7.85E-01
MBD3L4	methyl-CpG binding domain protein 3 like 4	1.24E-03	-4.27E-01
HMBOX1	homeobox containing 1	1.24E-03	-6.11E-01

COL1A1	collagen type I alpha 1 chain	1.24E-03	-4.10E-01
C21orf58	chromosome 21 open reading frame 58	1.24E-03	-7.82E-01
SHQ1	SHQ1, H/ACA ribonucleoprotein assembly factor	1.24E-03	7.48E-01
MID2	midline 2	1.24E-03	4.30E-01
ANKRD16	ankyrin repeat domain 16	1.25E-03	2.82E-01
APIP	APAF1 interacting protein	1.25E-03	8.46E-01
NOC4L	nucleolar complex associated 4 homolog	1.25E-03	5.20E-01
ENTPD6	ectonucleoside triphosphate diphosphohydrolase 6 (putative)	1.25E-03	-3.25E-01
ABHD3	abhydrolase domain containing 3	1.25E-03	-6.12E-01
SOX18	SRY-box 18	1.26E-03	-1.14
CTNNBL1	catenin beta like 1	1.26E-03	5.06E-01
CHD8	chromodomain helicase DNA binding protein 8	1.26E-03	-7.51E-01
RMND1	required for meiotic nuclear division 1 homolog	1.26E-03	-5.48E-01
BARX1	BARX homeobox 1	1.26E-03	4.82E-01
S100A13	S100 calcium binding protein A13	1.26E-03	-8.42E-01

TMEM259	transmembrane protein 259	1.26E-03	-5.05E-01
PLIN3	perilipin 3	1.27E-03	-6.84E-01
GYG1	glycogenin 1	1.27E-03	5.95E-01
STAMBP	STAM binding protein	1.27E-03	6.38E-01
ZNF586	zinc finger protein 586	1.27E-03	4.16E-01
NDUFAF5	NADH:ubiquinone oxidoreductase complex assembly factor 5	1.27E-03	6.83E-01
DIAPH1	diaphanous related formin 1	1.27E-03	8.30E-01
MAPK6	mitogen-activated protein kinase 6	1.27E-03	7.55E-01
HIST1H3E	histone cluster 1, H3e	1.27E-03	-4.06E-01
C21orf33	chromosome 21 open reading frame 33	1.27E-03	-1.1
TCP1	t-complex 1	1.27E-03	6.07E-01
RRAGD	Ras related GTP binding D	1.27E-03	-3.89E-01
SYCP2	synaptonemal complex protein 2	1.27E-03	-1.25
COMMD6	COMM domain containing 6	1.27E-03	-9.07E-01
SH3RF2	SH3 domain containing ring finger 2	1.27E-03	6.48E-01
PSMC3	proteasome 26S subunit, ATPase 3	1.28E-03	1.34
ALDH1A1	aldehyde dehydrogenase 1 family member A1	1.28E-03	2.74E-01

ABCA11P	ATP binding cassette subfamily A member 11, pseudogene	1.28E-03	3.31E -01
CCT6P1	chaperonin containing TCP1 subunit 6 pseudogene 1	1.28E-03	7.33E -01
TDO2	tryptophan 2,3-dioxygenase	1.28E-03	3.71E -01
EVL	Enah/Vasp-like	1.28E-03	- 8.67E -01
BBS2	Bardet-Biedl syndrome 2	1.28E-03	- 7.82E -01
APIP	APAF1 interacting protein	1.28E-03	7.38E -01
SP100	SP100 nuclear antigen	1.28E-03	3.10E -01
TCEA2	transcription elongation factor A2	1.28E-03	- 9.35E -01
UPK1A	uroplakin 1A	1.28E-03	-1.41
CREBRF	CREB3 regulatory factor	1.29E-03	- 6.68E -01
SPANXA1	sperm protein associated with the nucleus, X-linked, family member A1	1.29E-03	3.57E -01
ALMS1	ALMS1, centrosome and basal body associated protein	1.29E-03	4.01E -01
PLPP1	phospholipid phosphatase 1	1.29E-03	- 6.21E -01
ASPH	aspartate beta-hydroxylase	1.29E-03	6.73E -01
LOC100130745	uncharacterized LOC100130745	1.30E-03	- 3.61E -01

PPM1L	protein phosphatase, Mg2+/Mn2+ dependent 1L	1.30E-03	-3.65E-01
MYC	v-myc avian myelocytomatosis viral oncogene homolog	1.30E-03	-5.42E-01
NUDT1	nudix hydrolase 1	1.30E-03	4.84E-01
L3MBTL2	L3MBTL2 polycomb repressive complex 1 subunit	1.30E-03	3.75E-01
TMEM106B	transmembrane protein 106B	1.30E-03	-1.12
TOP3A	topoisomerase (DNA) III alpha	1.30E-03	5.94E-01
TRIM25	tripartite motif containing 25	1.31E-03	-7.25E-01
RAB18	RAB18, member RAS oncogene family	1.31E-03	7.70E-01
AKTIP	AKT interacting protein	1.31E-03	8.86E-01
TIFA	TRAF interacting protein with forkhead associated domain	1.31E-03	3.38E-01
NBN	nibrin	1.31E-03	1.14
AMMECR1	Alport syndrome, mental retardation, midface hypoplasia and elliptocytosis chromosomal region gene 1	1.31E-03	4.93E-01
HECA	hdc homolog, cell cycle regulator	1.31E-03	9.03E-01
TOE1	target of EGR1, member 1 (nuclear)	1.31E-03	4.25E-01
FAIM	Fas apoptotic inhibitory molecule	1.32E-03	9.43E-01
HAUS5	HAUS augmin like complex subunit 5	1.32E-03	3.29E-01
ANAPC4	anaphase promoting complex subunit 4	1.32E-03	4.17E-01

GPHN	gephyrin	1.32E-03	-6.36E-01
OBFC1	oligonucleotide/oligosaccharide binding fold containing 1	1.32E-03	-8.27E-01
CSNK2B	casein kinase 2 beta	1.32E-03	-5.59E-01
C1orf109	chromosome 1 open reading frame 109	1.32E-03	7.32E-01
C1QTNF9B	C1q and tumor necrosis factor related protein 9B	1.32E-03	4.03E-01
SPOPL	speckle type BTB/POZ protein like	1.32E-03	6.04E-01
NSL1	NSL1, MIS12 kinetochore complex component	1.32E-03	4.70E-01
PAICS	phosphoribosylaminoimidazole carboxylase; phosphoribosylaminoimidazolesuccinocarboxamide synthase	1.33E-03	6.41E-01
GLIPR2	GLI pathogenesis related 2	1.33E-03	-6.77E-01
HNRNPDL	heterogeneous nuclear ribonucleoprotein D like	1.33E-03	-4.65E-01
RPRD2	regulation of nuclear pre-mRNA domain containing 2	1.33E-03	-3.55E-01
KITLG	KIT ligand	1.33E-03	2.92E-01
TRAPPC6A	trafficking protein particle complex 6A	1.33E-03	-1.32
ZBTB2	zinc finger and BTB domain containing 2	1.34E-03	6.86E-01
RNF219	ring finger protein 219	1.34E-03	5.27E-01

PYGL	phosphorylase, glycogen, liver	1.34E-03	7.49E-01
CAMK2D	calcium/calmodulin dependent protein kinase II delta	1.34E-03	-5.34E-01
NID2	nidogen 2	1.34E-03	-1.05
ARHGEF39	Rho guanine nucleotide exchange factor 39	1.34E-03	4.12E-01
UNC93B1	unc-93 homolog B1 (C. elegans)	1.34E-03	-4.26E-01
ZNF669	zinc finger protein 669	1.34E-03	-6.80E-01
MOSPD3	motile sperm domain containing 3	1.34E-03	-7.89E-01
HOXC6	homeobox C6	1.34E-03	-3.35E-01
GAA	glucosidase alpha, acid	1.35E-03	-8.75E-01
STX1A	syntaxin 1A	1.35E-03	-6.30E-01
FTL	ferritin light chain	1.35E-03	-3.19E-01
SSB	Sjogren syndrome antigen B	1.35E-03	6.44E-01
TMEM68	transmembrane protein 68	1.35E-03	-3.60E-01
LTBP4	latent transforming growth factor beta binding protein 4	1.35E-03	-7.77E-01

RBPMS	RNA binding protein with multiple splicing	1.35E-03	-4.23E-01
ADGRE5	adhesion G protein-coupled receptor E5	1.35E-03	-9.59E-01
LEPR	leptin receptor	1.35E-03	3.24E-01
LRRC59	leucine rich repeat containing 59	1.36E-03	6.58E-01
SLITRK5	SLIT and NTRK like family member 5	1.36E-03	-4.69E-01
TMEM161A	transmembrane protein 161A	1.36E-03	-3.53E-01
EXOSC2	exosome component 2	1.36E-03	7.82E-01
MAPK9	mitogen-activated protein kinase 9	1.36E-03	5.10E-01
ASUN	asunder, spermatogenesis regulator	1.36E-03	8.47E-01
SNORD96A	small nucleolar RNA, C/D box 96A	1.36E-03	1.05
ASAH2B	N-acylsphingosine amidohydrolase 2B	1.36E-03	3.52E-01
ASAH1	N-acylsphingosine amidohydrolase 1	1.37E-03	-5.76E-01
TDP2	tyrosyl-DNA phosphodiesterase 2	1.37E-03	8.00E-01
CTNS	cystinosin, lysosomal cystine transporter	1.37E-03	2.92E-01
IFI6	interferon alpha inducible protein 6	1.37E-03	1.22
LUM	lumican	1.37E-03	-4.03E-01

GLTSCR1L	GLTSCR1 like	1.37E-03	-7.81E-01
FASTKD3	FAST kinase domains 3	1.38E-03	4.34E-01
HOXA13	homeobox A13	1.38E-03	4.03E-01
ATL1	atlastin GTPase 1	1.38E-03	3.46E-01
GLTP	glycolipid transfer protein	1.38E-03	-4.14E-01
TAF13	TATA-box binding protein associated factor 13	1.38E-03	5.10E-01
PRKD2	protein kinase D2	1.38E-03	-5.56E-01
LMBRD1	LMBR1 domain containing 1	1.38E-03	-6.53E-01
GJC2	gap junction protein gamma 2	1.38E-03	-9.87E-01
C9orf142	chromosome 9 open reading frame 142	1.38E-03	9.97E-01
JAG2	jagged 2	1.39E-03	-7.65E-01
SLC6A15	solute carrier family 6 member 15	1.39E-03	5.02E-01
H2AFX	H2A histone family member X	1.39E-03	1.06
C17orf58	chromosome 17 open reading frame 58	1.39E-03	-3.37E-01
ERGIC3	ERGIC and golgi 3	1.39E-03	-8.55E-01

S100A13	S100 calcium binding protein A13	1.39E-03	-7.14E-01
BICD1	BICD cargo adaptor 1	1.40E-03	-4.14E-01
CHCHD3	coiled-coil-helix-coiled-coil-helix domain containing 3	1.40E-03	5.37E-01
SLC25A40	solute carrier family 25 member 40	1.40E-03	4.75E-01
WDR12	WD repeat domain 12	1.40E-03	5.07E-01
RTEL1	regulator of telomere elongation helicase 1	1.41E-03	-4.26E-01
ACADVL	acyl-CoA dehydrogenase, very long chain	1.41E-03	-4.13E-01
LPIN1	lipin 1	1.41E-03	-3.45E-01
COL18A1	collagen type XVIII alpha 1 chain	1.41E-03	-5.25E-01
AHSA2	AHA1, activator of heat shock 90kDa protein ATPase homolog 2 (yeast)	1.41E-03	-1.01
GSTT2	glutathione S-transferase theta 2 (gene/pseudogene)	1.41E-03	-7.82E-01
DCAF11	DDB1 and CUL4 associated factor 11	1.41E-03	-5.83E-01
ACOX2	acyl-CoA oxidase 2	1.41E-03	-3.44E-01
OCIAD2	OCIA domain containing 2	1.41E-03	3.85E-01

RNASE4	ribonuclease A family member 4	1.42E-03	5.52E-01
CPSF3L	cleavage and polyadenylation specific factor 3-like	1.42E-03	-4.10E-01
GABPB1	GA binding protein transcription factor beta subunit 1	1.42E-03	3.83E-01
GRHPR	glyoxylate and hydroxypyruvate reductase	1.42E-03	-3.91E-01
IPO11	importin 11	1.42E-03	5.46E-01
AS3MT	arsenite methyltransferase	1.42E-03	7.15E-01
EI24	EI24, autophagy associated transmembrane protein	1.43E-03	-8.03E-01
GNG8	G protein subunit gamma 8	1.43E-03	-5.48E-01
TRPC1	transient receptor potential cation channel subfamily C member 1	1.43E-03	-8.18E-01
CSF2RA	colony stimulating factor 2 receptor alpha subunit	1.43E-03	-7.58E-01
ITFG1	integrin alpha FG-GAP repeat containing 1	1.43E-03	-8.25E-01
PRAF2	PRA1 domain family member 2	1.43E-03	-5.92E-01
RUNX2	runt related transcription factor 2	1.43E-03	-8.67E-01
C17orf58	chromosome 17 open reading frame 58	1.43E-03	-3.97E-01

LASP1	LIM and SH3 protein 1	1.43E-03	9.85E-01
HIST1H2AG	histone cluster 1, H2ag	1.43E-03	-4.09E-01
HIBADH	3-hydroxyisobutyrate dehydrogenase	1.44E-03	-5.07E-01
YIPF3	Yip1 domain family member 3	1.44E-03	-5.92E-01
PSMD10	proteasome 26S subunit, non-ATPase 10	1.44E-03	5.19E-01
RNFT2	ring finger protein, transmembrane 2	1.44E-03	-4.45E-01
SLC31A2	solute carrier family 31 member 2	1.44E-03	5.70E-01
MYCBP2	MYC binding protein 2, E3 ubiquitin protein ligase	1.44E-03	-1.02
NHS	NHS actin remodeling regulator	1.44E-03	-4.69E-01
PSIP1	PC4 and SFRS1 interacting protein 1	1.44E-03	9.24E-01
SOBP	sine oculis binding protein homolog	1.45E-03	-1.1
SEPW1	selenoprotein W, 1	1.45E-03	-4.98E-01
ABHD2	abhydrolase domain containing 2	1.45E-03	2.88E-01
CDC5L	cell division cycle 5 like	1.45E-03	1.02
CAPN2	calpain 2	1.45E-03	-5.29E-01

ZNF600	zinc finger protein 600	1.45E-03	-5.25E-01
REEP6	receptor accessory protein 6	1.45E-03	-1.02
FAM173B	family with sequence similarity 173 member B	1.46E-03	5.64E-01
SECISBP2	SECIS binding protein 2	1.46E-03	-4.53E-01
CCNJ	cyclin J	1.46E-03	5.99E-01
PIK3CB	phosphatidylinositol-4,5-bisphosphate 3-kinase catalytic subunit beta	1.46E-03	1.03
SDHAF3	succinate dehydrogenase complex assembly factor 3	1.46E-03	4.84E-01
DCUN1D4	defective in cullin neddylation 1 domain containing 4	1.46E-03	5.19E-01
LIF	leukemia inhibitory factor	1.46E-03	2.79E-01
ZNF235	zinc finger protein 235	1.47E-03	5.02E-01
GCC1	GRIP and coiled-coil domain containing 1	1.47E-03	4.99E-01
MRI1	methylthioribose-1-phosphate isomerase 1	1.48E-03	-7.28E-01
ZYX	zyxin	1.48E-03	7.19E-01
SLFN11	schlafen family member 11	1.48E-03	4.40E-01
BUD31	BUD31 homolog	1.48E-03	4.96E-01
ADAMTS20	ADAM metallopeptidase with thrombospondin type 1 motif 20	1.48E-03	3.12E-01
UGDH	UDP-glucose 6-dehydrogenase	1.48E-03	2.92E-01

ITGA6	integrin subunit alpha 6	1.48E-03	-5.32E-01
CWC22	CWC22 homolog, spliceosome-associated protein	1.48E-03	7.78E-01
ENPP1	ectonucleotide pyrophosphatase/phosphodiesterase 1	1.48E-03	2.62E-01
VBP1	VHL binding protein 1	1.48E-03	6.47E-01
SERPINE2	serpin family E member 2	1.49E-03	4.57E-01
CARD8	caspase recruitment domain family member 8	1.49E-03	-3.95E-01
PDE1A	phosphodiesterase 1A	1.49E-03	-3.71E-01
SUSD2	sushi domain containing 2	1.49E-03	-4.32E-01
VIM	vimentin	1.50E-03	6.24E-01
MID2	midline 2	1.50E-03	2.94E-01
PPP1R7	protein phosphatase 1 regulatory subunit 7	1.50E-03	4.22E-01
FASTKD3	FAST kinase domains 3	1.50E-03	5.94E-01
CPT1A	carnitine palmitoyltransferase 1A	1.50E-03	-3.45E-01
RPL13AP3	ribosomal protein L13a pseudogene 3	1.50E-03	-4.64E-01
ATP5G2	ATP synthase, H+ transporting, mitochondrial Fo complex subunit C2 (subunit 9)	1.51E-03	-8.40E-01

HOOK3	hook microtubule tethering protein 3	1.51E-03	3.59E-01
PRICKLE1	prickle planar cell polarity protein 1	1.51E-03	7.57E-01
KIAA1524	KIAA1524	1.51E-03	6.07E-01
SAP18	Sin3A associated protein 18	1.52E-03	-4.13E-01
HOXB3	homeobox B3	1.52E-03	5.03E-01
STEAP2	STEAP2 metalloreductase	1.52E-03	-5.71E-01
N6AMT1	N-6 adenine-specific DNA methyltransferase 1 (putative)	1.53E-03	-2.79E-01
AKR1C3	aldo-keto reductase family 1, member C3	1.53E-03	-7.01E-01
GXYLT1	glucoside xylosyltransferase 1	1.54E-03	-7.13E-01
NACC2	NACC family member 2	1.54E-03	6.58E-01
CTSZ	cathepsin Z	1.54E-03	3.04E-01
TLE4	transducin like enhancer of split 4	1.54E-03	-1.21
CACNB3	calcium voltage-gated channel auxiliary subunit beta 3	1.54E-03	-5.88E-01
CISD1	CDGSH iron sulfur domain 1	1.54E-03	4.02E-01
UGP2	UDP-glucose pyrophosphorylase 2	1.54E-03	-8.87E-01

NOC2LP2	NOC2 like nucleolar associated transcriptional repressor pseudogene 2	1.54E-03	3.73E-01
CLEC4A	C-type lectin domain family 4 member A	1.54E-03	5.09E-01
FBLN1	fibulin 1	1.54E-03	-5.64E-01
JRKL	JRK-like	1.54E-03	3.87E-01
TERF1	telomeric repeat binding factor 1	1.55E-03	7.85E-01
AKTIP	AKT interacting protein	1.55E-03	7.99E-01
NUTM1	NUT midline carcinoma family member 1	1.55E-03	2.76E-01
SBDSP1	Shwachman-Bodian-Diamond syndrome pseudogene 1	1.55E-03	6.28E-01
CNOT8	CCR4-NOT transcription complex subunit 8	1.55E-03	-5.57E-01
TPM2	tropomyosin 2 (beta)	1.55E-03	5.83E-01
CD59	CD59 molecule	1.56E-03	-4.04E-01
AKR1C4	aldo-keto reductase family 1, member C4	1.56E-03	-3.83E-01
TCTEX1D2	Tctex1 domain containing 2	1.56E-03	9.02E-01
PROSER1	proline and serine rich 1	1.56E-03	1.01
WRN	Werner syndrome RecQ like helicase	1.56E-03	3.24E-01
APH1B	aph-1 homolog B, gamma-secretase subunit	1.56E-03	-8.60E-01

FAM3A	family with sequence similarity 3 member A	1.56E-03	-5.86E-01
CCDC148	coiled-coil domain containing 148	1.56E-03	4.85E-01
C17orf100	chromosome 17 open reading frame 100	1.56E-03	-5.91E-01
CDK5R1	cyclin dependent kinase 5 regulatory subunit 1	1.56E-03	2.90E-01
SORT1	sortilin 1	1.57E-03	2.71E-01
C19orf12	chromosome 19 open reading frame 12	1.57E-03	-6.00E-01
SYN1	synapsin I	1.57E-03	5.22E-01
GK	glycerol kinase	1.58E-03	3.82E-01
AVPR2	arginine vasopressin receptor 2	1.58E-03	-3.16E-01
TSC22D1	TSC22 domain family member 1	1.58E-03	-7.42E-01
BTNL9	butyrophilin like 9	1.58E-03	3.94E-01
PORCN	porcupine homolog (Drosophila)	1.58E-03	-4.22E-01
TSPAN31	tetraspanin 31	1.58E-03	-7.75E-01
S1PR1	sphingosine-1-phosphate receptor 1	1.58E-03	-7.45E-01
DDX3X	DEAD-box helicase 3, X-linked	1.58E-03	1.24

HIPK2	homeodomain interacting protein kinase 2	1.58E-03	3.72E-01
AKIRIN2	akirin 2	1.59E-03	4.86E-01
KANK3	KN motif and ankyrin repeat domains 3	1.59E-03	-3.19E-01
ATG3	autophagy related 3	1.59E-03	6.05E-01
OCIAD2	OCIA domain containing 2	1.59E-03	3.70E-01
MLEC	malectin	1.60E-03	-8.81E-01
COL4A3BP	collagen type IV alpha 3 binding protein	1.60E-03	-5.63E-01
SLC39A7	solute carrier family 39 member 7	1.60E-03	-2.66E-01
PPP4R3B	protein phosphatase 4 regulatory subunit 3B	1.60E-03	-7.33E-01
SNRPB	small nuclear ribonucleoprotein polypeptides B and B1	1.60E-03	4.32E-01
FLRT3	fibronectin leucine rich transmembrane protein 3	1.60E-03	7.50E-01
GATSL3	GATS protein like 3	1.60E-03	-5.03E-01
SPOCD1	SPOC domain containing 1	1.60E-03	-6.17E-01
TRIT1	tRNA isopentenyltransferase 1	1.60E-03	4.13E-01
CNRIP1	cannabinoid receptor interacting protein 1	1.60E-03	-4.70E-01

C17orf53	chromosome 17 open reading frame 53	1.61E-03	9.38E-01
CTGF	connective tissue growth factor	1.61E-03	7.02E-01
CERS6	ceramide synthase 6	1.61E-03	-1.14
AK4	adenylate kinase 4	1.61E-03	-4.51E-01
POLR2K	RNA polymerase II subunit K	1.61E-03	7.75E-01
DHRS4	dehydrogenase/reductase 4	1.61E-03	-1.06
ZNF391	zinc finger protein 391	1.61E-03	3.45E-01
VWA1	von Willebrand factor A domain containing 1	1.61E-03	-3.53E-01
PTPN4	protein tyrosine phosphatase, non-receptor type 4	1.61E-03	-5.01E-01
RHBDD2	rhomboid domain containing 2	1.62E-03	-3.77E-01
RALB	RALB Ras like proto-oncogene B	1.62E-03	-4.93E-01
ZNF571	zinc finger protein 571	1.62E-03	3.94E-01
POLR3G	RNA polymerase III subunit G	1.62E-03	6.80E-01
BRWD3	bromodomain and WD repeat domain containing 3	1.62E-03	-5.31E-01
SP1	Sp1 transcription factor	1.62E-03	-8.65E-01

EMC7	ER membrane protein complex subunit 7	1.62E-03	-5.06E-01
NSUN6	NOP2/Sun RNA methyltransferase family member 6	1.62E-03	4.14E-01
GBP2	guanylate binding protein 2	1.62E-03	3.94E-01
DCAF13P3	DDB1 and CUL4 associated factor 13 pseudogene 3	1.62E-03	1.05
GNG5	G protein subunit gamma 5	1.62E-03	6.86E-01
SSR1	signal sequence receptor subunit 1	1.62E-03	-4.74E-01
CRAT	carnitine O-acetyltransferase	1.63E-03	-4.62E-01
CLCC1	chloride channel CLIC like 1	1.63E-03	3.30E-01
ALDH1B1	aldehyde dehydrogenase 1 family member B1	1.63E-03	5.46E-01
RMDN1	regulator of microtubule dynamics 1	1.63E-03	-4.56E-01
MAPKAP1	mitogen-activated protein kinase associated protein 1	1.63E-03	5.67E-01
RPL29	ribosomal protein L29	1.63E-03	-8.99E-01
MRI1	methylthioribose-1-phosphate isomerase 1	1.63E-03	-2.86E-01
CYHR1	cysteine and histidine rich 1	1.63E-03	-4.14E-01
NUP43	nucleoporin 43	1.63E-03	6.07E-01

RNF115	ring finger protein 115	1.64E-03	8.32E-01
GSDMB	gasdermin B	1.64E-03	-4.43E-01
PDLIM5	PDZ and LIM domain 5	1.64E-03	3.54E-01
NOL4	nucleolar protein 4	1.65E-03	4.67E-01
ACAP1	ArfGAP with coiled-coil, ankyrin repeat and PH domains 1	1.66E-03	2.95E-01
PPIG	peptidylprolyl isomerase G	1.66E-03	9.36E-01
ZNF766	zinc finger protein 766	1.66E-03	5.11E-01
ATXN2L	ataxin 2 like	1.66E-03	-3.21E-01
SLC39A3	solute carrier family 39 member 3	1.66E-03	4.88E-01
P2RX6	purinergic receptor P2X 6	1.67E-03	-1.09
TMEM94	transmembrane protein 94	1.67E-03	-1.04
AP2M1	adaptor related protein complex 2 mu 1 subunit	1.67E-03	3.37E-01
ZNF300P1	zinc finger protein 300 pseudogene 1	1.67E-03	-3.35E-01
PKNOX1	PBX/knotted 1 homeobox 1	1.68E-03	2.89E-01
GMPPB	GDP-mannose pyrophosphorylase B	1.68E-03	5.08E-01
C1orf43	chromosome 1 open reading frame 43	1.68E-03	-8.45E-01
GALNT18	polypeptide N-acetylgalactosaminyltransferase 18	1.68E-03	-3.45E-01

ARL16	ADP ribosylation factor like GTPase 16	1.68E-03	-7.94E-01
DENND1A	DENN domain containing 1A	1.68E-03	8.25E-01
LMBR1	limb development membrane protein 1	1.68E-03	7.01E-01
COBL	cordon-bleu WH2 repeat protein	1.68E-03	3.51E-01
ZMYND11	zinc finger MYND-type containing 11	1.69E-03	7.01E-01
CCDC150	coiled-coil domain containing 150	1.69E-03	5.66E-01
CENPV	centromere protein V	1.69E-03	1.57
ATP6AP1	ATPase H+ transporting accessory protein 1	1.69E-03	-7.90E-01
SDHAP3	succinate dehydrogenase complex flavoprotein subunit A pseudogene 3	1.69E-03	3.35E-01
FRAS1	Fraser extracellular matrix complex subunit 1	1.70E-03	2.83E-01
NOL3	nucleolar protein 3	1.70E-03	-1
YIF1B	Yip1 interacting factor homolog B, membrane trafficking protein	1.70E-03	-5.40E-01
EPS15	epidermal growth factor receptor pathway substrate 15	1.70E-03	4.13E-01
STRC	stereocilin	1.70E-03	-4.43E-01
EPT1	ethanolaminephosphotransferase 1	1.70E-03	-6.52E-01
MICAL1	microtubule associated monooxygenase, calponin and LIM domain containing 1	1.71E-03	-5.16E-01

PRDX3	peroxiredoxin 3	1.71E-03	4.92E-01
APOBEC3F	apolipoprotein B mRNA editing enzyme catalytic subunit 3F	1.71E-03	-3.36E-01
WDR33	WD repeat domain 33	1.71E-03	5.86E-01
NDUFAF4	NADH:ubiquinone oxidoreductase complex assembly factor 4	1.71E-03	9.31E-01
EPN2	epsin 2	1.71E-03	-4.28E-01
BLVRB	biliverdin reductase B	1.72E-03	-1.07
PAICS	phosphoribosylaminoimidazole carboxylase; phosphoribosylaminoimidazolesuccinocarboxamide synthase	1.72E-03	6.58E-01
SPRTN	SprT-like N-terminal domain	1.72E-03	-3.94E-01
ZFP1	ZFP1 zinc finger protein	1.72E-03	4.91E-01
DHRS4L2	dehydrogenase/reductase 4 like 2	1.72E-03	-1.13
CRLF3	cytokine receptor like factor 3	1.73E-03	4.80E-01
ATP5F1	ATP synthase, H+ transporting, mitochondrial Fo complex subunit B1	1.73E-03	-6.40E-01
HLCS	holocarboxylase synthetase	1.73E-03	-3.61E-01
NDE1	nudE neurodevelopment protein 1	1.73E-03	-4.00E-01
HSPA8	heat shock protein family A (Hsp70) member 8	1.73E-03	6.70E-01

MYLIP	myosin regulatory light chain interacting protein	1.73E-03	-4.21E-01
CEP41	centrosomal protein 41	1.73E-03	5.08E-01
TSC22D3	TSC22 domain family member 3	1.74E-03	-3.99E-01
SERBP1	SERPINE1 mRNA binding protein 1	1.74E-03	-3.60E-01
IL21R	interleukin 21 receptor	1.74E-03	-2.49E-01
ECI2	enoyl-CoA delta isomerase 2	1.74E-03	2.80E-01
PIGT	phosphatidylinositol glycan anchor biosynthesis class T	1.74E-03	4.27E-01
ACSS2	acyl-CoA synthetase short-chain family member 2	1.75E-03	-5.24E-01
UPF3B	UPF3 regulator of nonsense transcripts homolog B (yeast)	1.75E-03	5.93E-01
SLC25A20	solute carrier family 25 member 20	1.75E-03	-5.34E-01
SNHG5	small nucleolar RNA host gene 5	1.75E-03	-8.81E-01
MMP23B	matrix metallopeptidase 23B	1.75E-03	-3.96E-01
FUZ	fuzzy planar cell polarity protein	1.75E-03	-2.78E-01
PGF	placental growth factor	1.76E-03	-8.26E-01

RAB3A	RAB3A, member RAS oncogene family	1.76E-03	-5.24E-01
SLC35A5	solute carrier family 35 member A5	1.76E-03	-2.90E-01
RNPC3	RNA binding region (RNP1, RRM) containing 3	1.76E-03	-6.54E-01
TICAM2	toll like receptor adaptor molecule 2	1.76E-03	5.08E-01
CCZ1B	CCZ1 homolog B, vacuolar protein trafficking and biogenesis associated	1.76E-03	5.80E-01
KLRG2	killer cell lectin like receptor G2	1.76E-03	-2.63E-01
C2orf76	chromosome 2 open reading frame 76	1.77E-03	8.15E-01
TMEM106B	transmembrane protein 106B	1.77E-03	-1.09
EPRS	glutamyl-prolyl-tRNA synthetase	1.77E-03	6.98E-01
CCDC93	coiled-coil domain containing 93	1.77E-03	5.21E-01
TWF2	twinfilin actin binding protein 2	1.77E-03	-6.18E-01
P2RY11	purinergic receptor P2Y11	1.78E-03	-8.41E-01
CHD9	chromodomain helicase DNA binding protein 9	1.78E-03	-1.09
TTC32	tetratricopeptide repeat domain 32	1.78E-03	-6.62E-01
CDK1	cyclin dependent kinase 1	1.78E-03	5.79E-01

TNFRSF1A	TNF receptor superfamily member 1A	1.78E-03	-6.68E-01
EFL1	elongation factor like GTPase 1	1.79E-03	6.38E-01
STEAP2	STEAP2 metalloreductase	1.79E-03	-5.88E-01
ABCE1	ATP binding cassette subfamily E member 1	1.79E-03	1.44
SF3B2	splicing factor 3b subunit 2	1.79E-03	1.07
SUB1	SUB1 homolog, transcriptional regulator	1.79E-03	4.96E-01
CTNNAL1	catenin alpha like 1	1.79E-03	1.01
SPX	spexin hormone	1.79E-03	3.08E-01
SSR2	signal sequence receptor subunit 2	1.80E-03	-8.59E-01
SAE1	SUMO1 activating enzyme subunit 1	1.80E-03	4.52E-01
LINC00704	long intergenic non-protein coding RNA 704	1.80E-03	3.07E-01
EIF4G2	eukaryotic translation initiation factor 4 gamma 2	1.80E-03	1.23
PTRF	polymerase I and transcript release factor	1.80E-03	9.53E-01
LAMTOR4	late endosomal/lysosomal adaptor, MAPK and MTOR activator 4	1.80E-03	-9.38E-01
RGS12	regulator of G-protein signaling 12	1.80E-03	4.41E-01
LRTOMT	leucine rich transmembrane and O-methyltransferase domain containing	1.80E-03	4.02E-01
CDK5R1	cyclin dependent kinase 5 regulatory subunit 1	1.80E-03	5.91E-01

METTL5	methyltransferase like 5	1.80E-03	6.56E -01
DUSP12	dual specificity phosphatase 12	1.81E-03	5.66E -01
FEZ1	fasciculation and elongation protein zeta 1	1.81E-03	- 3.16E -01
BRSK1	BR serine/threonine kinase 1	1.81E-03	- 6.10E -01
WBSCR27	Williams Beuren syndrome chromosome region 27	1.81E-03	-1.06
CALD1	caldesmon 1	1.81E-03	5.43E -01
AVEN	apoptosis and caspase activation inhibitor	1.81E-03	5.42E -01
LGMN	legumain	1.82E-03	- 3.91E -01
NAA15	N(alpha)-acetyltransferase 15, NatA auxiliary subunit	1.82E-03	8.06E -01
SRSF12	serine and arginine rich splicing factor 12	1.82E-03	5.60E -01
TMEM63B	transmembrane protein 63B	1.82E-03	- 9.84E -01
LPAR2	lysophosphatidic acid receptor 2	1.82E-03	- 4.92E -01
NAP1L2	nucleosome assembly protein 1 like 2	1.82E-03	3.38E -01
CPVL	carboxypeptidase, vitellogenic like	1.82E-03	4.44E -01
METTL2A	methyltransferase like 2A	1.82E-03	- 5.56E -01

ALDH1A3	aldehyde dehydrogenase 1 family member A3	1.82E-03	-5.99E-01
RPIA	ribose 5-phosphate isomerase A	1.82E-03	7.28E-01
KCNG3	potassium voltage-gated channel modifier subfamily G member 3	1.82E-03	3.14E-01
ZSCAN12P1	zinc finger and SCAN domain containing 12 pseudogene 1	1.82E-03	-7.63E-01
NFASC	neurofascin	1.82E-03	4.29E-01
PURB	purine rich element binding protein B	1.83E-03	-3.76E-01
ZNF667	zinc finger protein 667	1.83E-03	-5.99E-01
SLC25A4	solute carrier family 25 member 4	1.83E-03	5.57E-01
SLC22A15	solute carrier family 22 member 15	1.83E-03	-8.43E-01
PBX2	PBX homeobox 2	1.83E-03	-9.33E-01
IGFLR1	IGF like family receptor 1	1.83E-03	5.43E-01
MTFMT	mitochondrial methionyl-tRNA formyltransferase	1.83E-03	4.04E-01
KLRC3	killer cell lectin like receptor C3	1.84E-03	3.07E-01
SNORD1A	small nucleolar RNA, C/D box 1A	1.84E-03	-3.84E-01
TMEM245	transmembrane protein 245	1.84E-03	3.71E-01

GDPD1	glycerophosphodiester phosphodiesterase domain containing 1	1.84E-03	-5.50E-01
ALG11	ALG11, alpha-1,2-mannosyltransferase	1.84E-03	-4.89E-01
ERBB3	erb-b2 receptor tyrosine kinase 3	1.84E-03	5.91E-01
CPEB1	cytoplasmic polyadenylation element binding protein 1	1.85E-03	3.79E-01
HERC5	HECT and RLD domain containing E3 ubiquitin protein ligase 5	1.85E-03	6.20E-01
PRAME	preferentially expressed antigen in melanoma	1.85E-03	-5.29E-01
CTDSP1	CTD small phosphatase 1	1.85E-03	-3.92E-01
RTN4R	reticulon 4 receptor	1.86E-03	4.13E-01
RPEL1	ribulose-5-phosphate-3-epimerase like 1	1.86E-03	6.80E-01
HSCB	HscB mitochondrial iron-sulfur cluster cochaperone	1.86E-03	-1.02
BCCIP	BRCA2 and CDKN1A interacting protein	1.86E-03	4.99E-01
TAF13	TATA-box binding protein associated factor 13	1.86E-03	4.88E-01
BMS1	BMS1, ribosome biogenesis factor	1.86E-03	7.53E-01
CCDC43	coiled-coil domain containing 43	1.87E-03	-4.79E-01
CABIN1	calcineurin binding protein 1	1.87E-03	-5.63E-01
NCAPD2	non-SMC condensin I complex subunit D2	1.87E-03	1.04

MARVELD3	MARVEL domain containing 3	1.87E-03	3.56E-01
SRFBP1	serum response factor binding protein 1	1.87E-03	3.46E-01
TMEM14A	transmembrane protein 14A	1.87E-03	6.26E-01
RBBP8	RB binding protein 8, endonuclease	1.88E-03	3.25E-01
ZDHHC6	zinc finger DHHC-type containing 6	1.88E-03	7.22E-01
SNRPD1	small nuclear ribonucleoprotein D1 polypeptide	1.88E-03	7.06E-01
ERCC5	ERCC excision repair 5, endonuclease	1.88E-03	-9.54E-01
PHIP	pleckstrin homology domain interacting protein	1.88E-03	6.96E-01
DACH2	dachshund family transcription factor 2	1.89E-03	3.75E-01
TEX264	testis expressed 264	1.89E-03	-6.34E-01
SYCP2	synaptonemal complex protein 2	1.89E-03	-1.45
NCKIPSD	NCK interacting protein with SH3 domain	1.89E-03	7.14E-01
SUZ12	SUZ12 polycomb repressive complex 2 subunit	1.89E-03	4.65E-01
DNMT3B	DNA methyltransferase 3 beta	1.89E-03	7.42E-01
ITGB2	integrin subunit beta 2	1.89E-03	-5.85E-01
KLC1	kinesin light chain 1	1.89E-03	5.67E-01
HOXC11	homeobox C11	1.89E-03	-3.89E-01

MAPK9	mitogen-activated protein kinase 9	1.89E-03	8.34E-01
NDRG2	NDRG family member 2	1.90E-03	-3.03E-01
MRPS15	mitochondrial ribosomal protein S15	1.90E-03	6.50E-01
ALDH18A1	aldehyde dehydrogenase 18 family member A1	1.90E-03	4.83E-01
GSG1	germ cell associated 1	1.90E-03	3.16E-01
LIMK2	LIM domain kinase 2	1.90E-03	-5.90E-01
IFRD2	interferon related developmental regulator 2	1.90E-03	5.62E-01
CYB5A	cytochrome b5 type A	1.90E-03	-7.77E-01
TMEM126B	transmembrane protein 126B	1.90E-03	-5.57E-01
IFITM2	interferon induced transmembrane protein 2	1.90E-03	6.74E-01
GCH1	GTP cyclohydrolase 1	1.90E-03	3.12E-01
ANKDD1A	ankyrin repeat and death domain containing 1A	1.90E-03	5.12E-01
CCNI	cyclin I	1.90E-03	-4.73E-01
DAAM1	dishevelled associated activator of morphogenesis 1	1.91E-03	8.29E-01
MAP7D3	MAP7 domain containing 3	1.91E-03	5.84E-01

ITPKC	inositol-trisphosphate 3-kinase C	1.91E-03	-3.65E-01
UTP6	UTP6, small subunit processome component	1.91E-03	3.16E-01
ISG15	ISG15 ubiquitin-like modifier	1.92E-03	1.08
NDUFA4	NDUFA4, mitochondrial complex associated	1.92E-03	3.73E-01
WDYHV1	WDYHV motif containing 1	1.92E-03	7.26E-01
NDST2	N-deacetylase and N-sulfotransferase 2	1.93E-03	-3.99E-01
DMD	dystrophin	1.94E-03	-3.93E-01
SH3RF1	SH3 domain containing ring finger 1	1.94E-03	7.72E-01
MRPL50	mitochondrial ribosomal protein L50	1.94E-03	5.33E-01
GAD1	glutamate decarboxylase 1	1.94E-03	-2.89E-01
XYLB	xylulokinase	1.94E-03	5.43E-01
MRPL10	mitochondrial ribosomal protein L10	1.94E-03	-3.57E-01
EPHA3	EPH receptor A3	1.94E-03	-8.41E-01
SCN1A	sodium voltage-gated channel alpha subunit 1	1.94E-03	3.54E-01
ELMOD3	ELMO domain containing 3	1.94E-03	3.69E-01
RARB	retinoic acid receptor beta	1.95E-03	2.61E-01

GOT2	glutamic-oxaloacetic transaminase 2	1.95E-03	5.78E-01
CD79B	CD79b molecule	1.95E-03	-2.87E-01
DIS3L	DIS3 like exosome 3'-5' exoribonuclease	1.95E-03	6.09E-01
TRAPPC4	trafficking protein particle complex 4	1.95E-03	8.35E-01
CDK12	cyclin dependent kinase 12	1.95E-03	6.68E-01
PTTG2	pituitary tumor-transforming 2	1.95E-03	3.75E-01
DR1	down-regulator of transcription 1	1.95E-03	1.12
AASDH	aminoadipate-semialdehyde dehydrogenase	1.95E-03	-4.78E-01
RBCK1	RANBP2-type and C3HC4-type zinc finger containing 1	1.95E-03	-6.34E-01
GFPT1	glutamine--fructose-6-phosphate transaminase 1	1.96E-03	5.73E-01
XPC	XPC complex subunit, DNA damage recognition and repair factor	1.96E-03	-1.41
CTDSPL2	CTD small phosphatase like 2	1.96E-03	9.54E-01
ZNF226	zinc finger protein 226	1.96E-03	-6.37E-01
CRCP	CGRP receptor component	1.96E-03	-5.55E-01
CDKN2B	cyclin dependent kinase inhibitor 2B	1.96E-03	4.03E-01
FAM98A	family with sequence similarity 98 member A	1.96E-03	3.72E-01

AES	amino-terminal enhancer of split	1.96E-03	-4.90E-01
POGZ	pogo transposable element with ZNF domain	1.96E-03	-3.95E-01
CCDC13	coiled-coil domain containing 13	1.96E-03	-4.35E-01
ZNF431	zinc finger protein 431	1.97E-03	5.17E-01
MFAP1	microfibrillar associated protein 1	1.97E-03	4.47E-01
GLG1	golgi glycoprotein 1	1.97E-03	-8.10E-01
MAN1A1	mannosidase alpha class 1A member 1	1.97E-03	-1.03
ENOSF1	enolase superfamily member 1	1.98E-03	-4.48E-01
GPR4	G protein-coupled receptor 4	1.98E-03	-5.00E-01
ACSL4	acyl-CoA synthetase long-chain family member 4	1.98E-03	7.38E-01
RNF11	ring finger protein 11	1.99E-03	5.29E-01
PHAX	phosphorylated adaptor for RNA export	1.99E-03	-7.03E-01
TDRD1	tudor domain containing 1	1.99E-03	-6.98E-01
CYTH1	cytohesin 1	1.99E-03	-6.07E-01

ANKRD16	ankyrin repeat domain 16	1.99E-03	-6.81E-01
KIAA0408	KIAA0408	1.99E-03	-5.86E-01
CDK10	cyclin dependent kinase 10	1.99E-03	-2.63E-01
CFAP69	cilia and flagella associated protein 69	2.00E-03	3.89E-01
ZNF197	zinc finger protein 197	2.00E-03	4.33E-01
SIL1	SIL1 nucleotide exchange factor	2.00E-03	-9.11E-01
UFD1L	ubiquitin fusion degradation 1 like (yeast)	2.00E-03	4.77E-01
SLK	STE20 like kinase	2.00E-03	9.33E-01
KLHL23	kelch like family member 23	2.00E-03	4.22E-01
FZD7	frizzled class receptor 7	2.00E-03	-3.93E-01
HIGD2A	HIG1 hypoxia inducible domain family member 2A	2.00E-03	-8.18E-01
HSF2	heat shock transcription factor 2	2.00E-03	-9.00E-01
PCDHB12	protocadherin beta 12	2.00E-03	-2.58E-01
PAOX	polyamine oxidase (exo-N4-amino)	2.01E-03	-5.40E-01

FBXL19-AS1	FBXL19 antisense RNA 1 (head to head)	2.01E-03	-6.71E-01
GIN1	gypsy retrotransposon integrase 1	2.01E-03	-2.51E-01
CYBRD1	cytochrome b reductase 1	2.01E-03	-9.49E-01
MYL12A	myosin light chain 12A	2.01E-03	4.08E-01
PTS	6-pyruvoyltetrahydropterin synthase	2.01E-03	4.41E-01
RTN1	reticulon 1	2.02E-03	2.65E-01
CCNE1	cyclin E1	2.02E-03	4.67E-01
CGB5	chorionic gonadotropin beta subunit 5	2.02E-03	-2.68E-01
LRRCC1	leucine rich repeat and coiled-coil centrosomal protein 1	2.02E-03	2.93E-01
JUP	junction plakoglobin	2.02E-03	-8.89E-01
SNORA5C	small nucleolar RNA, H/ACA box 5C	2.02E-03	-3.22E-01
FAM76A	family with sequence similarity 76 member A	2.03E-03	4.27E-01
ASTN2	astrotactin 2	2.03E-03	-1.05
MRGBP	MRG/MORF4L binding protein	2.03E-03	7.33E-01
MIR330	microRNA 330	2.04E-03	-7.99E-01

VPS37B	VPS37B, ESCRT-I subunit	2.04E-03	7.49E-01
NMNAT1	nicotinamide nucleotide adenylyltransferase 1	2.04E-03	-4.34E-01
USP9X	ubiquitin specific peptidase 9, X-linked	2.04E-03	-5.45E-01
HDGFRP3	hepatoma-derived growth factor, related protein 3	2.05E-03	6.84E-01
POLR1E	RNA polymerase I subunit E	2.06E-03	4.31E-01
TNFAIP8L1	TNF alpha induced protein 8 like 1	2.06E-03	-9.69E-01
RAB30	RAB30, member RAS oncogene family	2.06E-03	4.01E-01
NCBP2	nuclear cap binding protein subunit 2	2.06E-03	-4.46E-01
RAP1A	RAP1A, member of RAS oncogene family	2.06E-03	3.90E-01
LAP3	leucine aminopeptidase 3	2.06E-03	6.37E-01
DCAF13	DDB1 and CUL4 associated factor 13	2.07E-03	8.33E-01
UBE2K	ubiquitin conjugating enzyme E2 K	2.07E-03	8.19E-01
LSM3	LSM3 homolog, U6 small nuclear RNA and mRNA degradation associated	2.07E-03	1.06
PGGT1B	protein geranylgeranyltransferase type I subunit beta	2.08E-03	5.66E-01
APEH	acylaminoacyl-peptide hydrolase	2.08E-03	-5.50E-01

C14orf2	chromosome 14 open reading frame 2	2.08E-03	-6.99E-01
PI4K2A	phosphatidylinositol 4-kinase type 2 alpha	2.08E-03	-5.65E-01
ATXN7L2	ataxin 7 like 2	2.09E-03	4.69E-01
AKR1A1	aldo-keto reductase family 1 member A1	2.09E-03	3.84E-01
C17orf80	chromosome 17 open reading frame 80	2.09E-03	-7.19E-01
DHX58	DEXH-box helicase 58	2.10E-03	2.64E-01
CCM2	CCM2 scaffolding protein	2.10E-03	7.41E-01
ZNF786	zinc finger protein 786	2.10E-03	-8.58E-01
SSR4	signal sequence receptor subunit 4	2.10E-03	-5.24E-01
BCCIP	BRCA2 and CDKN1A interacting protein	2.11E-03	3.73E-01
OSBPL5	oxysterol binding protein like 5	2.11E-03	-4.68E-01
PTBP1	polypyrimidine tract binding protein 1	2.11E-03	6.66E-01
U2AF2	U2 small nuclear RNA auxiliary factor 2	2.11E-03	4.08E-01
COG5	component of oligomeric golgi complex 5	2.11E-03	4.01E-01
EPHA8	EPH receptor A8	2.11E-03	-2.52E-01

ARPC1A	actin related protein 2/3 complex subunit 1A	2.12E-03	3.64E-01
TMEM267	transmembrane protein 267	2.13E-03	-8.47E-01
CAP1	adenylate cyclase associated protein 1	2.13E-03	3.43E-01
FBXO17	F-box protein 17	2.13E-03	-4.08E-01
HABP4	hyaluronan binding protein 4	2.14E-03	2.61E-01
TAP2	transporter 2, ATP binding cassette subfamily B member	2.14E-03	5.91E-01
SIRT2	sirtuin 2	2.14E-03	-5.00E-01
AGPAT3	1-acylglycerol-3-phosphate O-acyltransferase 3	2.15E-03	2.52E-01
ECI1	enoyl-CoA delta isomerase 1	2.15E-03	-9.06E-01
NAP1L5	nucleosome assembly protein 1 like 5	2.15E-03	-4.45E-01
TAGLN2	transgelin 2	2.15E-03	4.41E-01
BCLAF1	BCL2 associated transcription factor 1	2.16E-03	7.06E-01
MBNL1	muscleblind like splicing regulator 1	2.16E-03	5.32E-01
SERPINI1	serpin family I member 1	2.16E-03	6.00E-01
NR2F6	nuclear receptor subfamily 2 group F member 6	2.16E-03	-7.67E-01

APPBP2	amyloid beta precursor protein binding protein 2	2.16E-03	-5.12E-01
CBX5	chromobox 5	2.16E-03	8.35E-01
MAGED1	MAGE family member D1	2.16E-03	2.66E-01
CEP104	centrosomal protein 104	2.16E-03	5.87E-01
LINC01278	long intergenic non-protein coding RNA 1278	2.17E-03	3.92E-01
ABLIM1	actin binding LIM protein 1	2.17E-03	7.54E-01
TMEM178A	transmembrane protein 178A	2.17E-03	-2.98E-01
LIMS1	LIM zinc finger domain containing 1	2.17E-03	8.45E-01
UNC119	unc-119 lipid binding chaperone	2.17E-03	-9.50E-01
PREPL	prolyl endopeptidase-like	2.17E-03	-1.13
MAP7	microtubule associated protein 7	2.17E-03	3.75E-01
BLCAP	bladder cancer associated protein	2.17E-03	-6.86E-01
UBE2K	ubiquitin conjugating enzyme E2 K	2.17E-03	5.57E-01
SYNJ2BP	synaptojanin 2 binding protein	2.18E-03	-6.02E-01
SGCA	sarcoglycan alpha	2.18E-03	-4.85E-01

TRIM5	tripartite motif containing 5	2.18E-03	-5.25E-01
PDE1A	phosphodiesterase 1A	2.18E-03	-4.01E-01
COPZ1	coatomer protein complex subunit zeta 1	2.18E-03	-3.11E-01
INPP5F	inositol polyphosphate-5-phosphatase F	2.19E-03	2.59E-01
IDH3G	isocitrate dehydrogenase 3 (NAD(+)) gamma	2.19E-03	-7.85E-01
AZU1	azurocidin 1	2.19E-03	-3.67E-01
APLP1	amyloid beta precursor like protein 1	2.19E-03	-7.80E-01
ABCE1	ATP binding cassette subfamily E member 1	2.19E-03	1.29
C11orf49	chromosome 11 open reading frame 49	2.20E-03	-4.68E-01
PDGFRB	platelet derived growth factor receptor beta	2.20E-03	-5.83E-01
IFIT2	interferon induced protein with tetratricopeptide repeats 2	2.20E-03	5.68E-01
MAPK8IP1	mitogen-activated protein kinase 8 interacting protein 1	2.20E-03	4.33E-01
CRSP8P	mediator complex subunit 27 pseudogene	2.20E-03	7.84E-01
HINT1	histidine triad nucleotide binding protein 1	2.20E-03	5.71E-01
TEN1	TEN1 CST complex subunit	2.21E-03	-6.12E-01

PDF	peptide deformylase (mitochondrial)	2.21E-03	4.83E-01
TRIB2	tribbles pseudokinase 2	2.21E-03	2.77E-01
MPZL1	myelin protein zero like 1	2.21E-03	8.22E-01
ZFHX4	zinc finger homeobox 4	2.21E-03	4.24E-01
C8orf88	chromosome 8 open reading frame 88	2.21E-03	2.84E-01
CTDSPL2	CTD small phosphatase like 2	2.21E-03	8.39E-01
UBE2O	ubiquitin conjugating enzyme E2 O	2.22E-03	-7.41E-01
ING2	inhibitor of growth family member 2	2.22E-03	5.11E-01
H2AFJ	H2A histone family member J	2.23E-03	-9.83E-01
NMD3	NMD3 ribosome export adaptor	2.23E-03	8.50E-01
CRYBG3	crystallin beta-gamma domain containing 3	2.24E-03	2.74E-01
GCNT1	glucosaminyl (N-acetyl) transferase 1, core 2	2.24E-03	3.32E-01
MRPL42	mitochondrial ribosomal protein L42	2.24E-03	6.84E-01
NFIX	nuclear factor I X	2.24E-03	-7.31E-01
COX18	COX18, cytochrome c oxidase assembly factor	2.24E-03	3.04E-01
CNFN	cornifelin	2.25E-03	-8.62E-01

ABCF2	ATP binding cassette subfamily F member 2	2.25E-03	2.77E-01
TSPYL2	TSPY like 2	2.25E-03	-5.85E-01
EIF2A	eukaryotic translation initiation factor 2A	2.25E-03	-3.86E-01
SQLE	squalene epoxidase	2.25E-03	5.58E-01
FAM217B	family with sequence similarity 217 member B	2.25E-03	-8.99E-01
GCN1	GCN1, eIF2 alpha kinase activator homolog	2.25E-03	6.48E-01
LINC00461	long intergenic non-protein coding RNA 461	2.25E-03	-4.05E-01
LOXL4	lysyl oxidase like 4	2.25E-03	-3.63E-01
NUBP1	nucleotide binding protein 1	2.25E-03	5.40E-01
CDC37L1	cell division cycle 37 like 1	2.26E-03	6.56E-01
RBM47	RNA binding motif protein 47	2.26E-03	2.80E-01
ZBTB25	zinc finger and BTB domain containing 25	2.26E-03	2.78E-01
BEX1	brain expressed X-linked 1	2.27E-03	-1.21
KIF3A	kinesin family member 3A	2.27E-03	4.39E-01
LINC00936	long intergenic non-protein coding RNA 936	2.27E-03	-8.03E-01
HNRNPH1	heterogeneous nuclear ribonucleoprotein H1 (H)	2.27E-03	1.08

RFESD	Rieske Fe-S domain containing	2.28E-03	3.31E-01
MED11	mediator complex subunit 11	2.28E-03	-5.89E-01
GNG7	G protein subunit gamma 7	2.28E-03	-5.42E-01
MYT1	myelin transcription factor 1	2.28E-03	-2.73E-01
USP38	ubiquitin specific peptidase 38	2.28E-03	5.43E-01
GDPD5	glycerophosphodiester phosphodiesterase domain containing 5	2.28E-03	-2.36E-01
BEX3	brain expressed X-linked 3	2.28E-03	-9.33E-01
ILVBL	ilvB acetolactate synthase like	2.28E-03	-7.57E-01
ZKSCAN3	zinc finger with KRAB and SCAN domains 3	2.28E-03	3.82E-01
ALDH9A1	aldehyde dehydrogenase 9 family member A1	2.29E-03	5.05E-01
SLC37A1	solute carrier family 37 member 1	2.29E-03	3.99E-01
ADAMTSL4	ADAMTS like 4	2.29E-03	-6.32E-01
EIF4A3	eukaryotic translation initiation factor 4A3	2.30E-03	2.53E-01
SEC22C	SEC22 homolog C, vesicle trafficking protein	2.31E-03	3.21E-01
SUGP2	SURP and G-patch domain containing 2	2.31E-03	-6.75E-01

CLK3	CDC like kinase 3	2.31E-03	-8.35E-01
SP4	Sp4 transcription factor	2.31E-03	4.97E-01
KHDRBS3	KH RNA binding domain containing, signal transduction associated 3	2.32E-03	4.15E-01
TRIP12	thyroid hormone receptor interactor 12	2.32E-03	1.35
TDP2	tyrosyl-DNA phosphodiesterase 2	2.32E-03	9.23E-01
ORMDL1	ORMDL sphingolipid biosynthesis regulator 1	2.32E-03	-4.78E-01
ABHD14A	abhydrolase domain containing 14A	2.32E-03	-1.26
CORO2A	coronin 2A	2.32E-03	-3.92E-01
CYP20A1	cytochrome P450 family 20 subfamily A member 1	2.33E-03	5.72E-01
SPRED2	sprouty related EVH1 domain containing 2	2.33E-03	-6.25E-01
DDX31	DEAD-box helicase 31	2.33E-03	3.26E-01
CLASRP	CLK4 associating serine/arginine rich protein	2.34E-03	-3.15E-01
SHC1	SHC adaptor protein 1	2.34E-03	-5.93E-01
EVI5	ecotropic viral integration site 5	2.34E-03	-7.00E-01
ANP32E	acidic nuclear phosphoprotein 32 family member E	2.34E-03	3.33E-01

METTL9	methyltransferase like 9	2.34E-03	-6.70E-01
PDE12	phosphodiesterase 12	2.34E-03	6.23E-01
FBXO38	F-box protein 38	2.35E-03	-5.71E-01
NCL	nucleolin	2.35E-03	5.66E-01
FAM188A	family with sequence similarity 188 member A	2.35E-03	4.14E-01
MINCR	MYC-induced long noncoding RNA	2.35E-03	-2.86E-01
SCML1	sex comb on midleg-like 1 (Drosophila)	2.35E-03	-8.62E-01
DHX16	DEAH-box helicase 16	2.35E-03	6.14E-01
USP12	ubiquitin specific peptidase 12	2.35E-03	5.73E-01
CCNYL1	cyclin Y like 1	2.36E-03	4.33E-01
PA2G4	proliferation-associated 2G4	2.36E-03	3.22E-01
NDUFA4L2	NDUFA4, mitochondrial complex associated like 2	2.36E-03	-4.00E-01
MCU	mitochondrial calcium uniporter	2.36E-03	6.49E-01
P2RY2	purinergic receptor P2Y2	2.36E-03	4.48E-01
SPHK2	sphingosine kinase 2	2.36E-03	-9.22E-01

SIMC1	SUMO interacting motifs containing 1	2.36E-03	5.54E-01
SULT1A2	sulfotransferase family 1A member 2	2.36E-03	-3.32E-01
ARIH1	ariadne RBR E3 ubiquitin protein ligase 1	2.37E-03	4.13E-01
CD47	CD47 molecule	2.37E-03	3.64E-01
PPP4R4	protein phosphatase 4 regulatory subunit 4	2.37E-03	3.71E-01
WLS	wntless Wnt ligand secretion mediator	2.37E-03	3.54E-01
RANBP3	RAN binding protein 3	2.38E-03	4.69E-01
HOXA11-AS	HOXA11 antisense RNA	2.38E-03	2.92E-01
MAGEL2	MAGE family member L2	2.38E-03	-4.61E-01
PRKACB	protein kinase cAMP-activated catalytic subunit beta	2.38E-03	4.04E-01
IGSF3	immunoglobulin superfamily member 3	2.38E-03	-3.16E-01
UQCC1	ubiquinol-cytochrome c reductase complex assembly factor 1	2.38E-03	-5.34E-01
RBM18	RNA binding motif protein 18	2.38E-03	4.75E-01
RTCA	RNA 3'-terminal phosphate cyclase	2.39E-03	6.05E-01
NOP10	NOP10 ribonucleoprotein	2.39E-03	2.98E-01
FAM213A	family with sequence similarity 213 member A	2.39E-03	3.93E-01

SEMA3B	semaphorin 3B	2.40E-03	-2.99E-01
CSHL1	chorionic somatomammotropin hormone like 1	2.40E-03	-5.24E-01
TMEM126B	transmembrane protein 126B	2.40E-03	-3.46E-01
SLC12A9	solute carrier family 12 member 9	2.40E-03	-8.27E-01
TPGS2	tubulin polyglutamylase complex subunit 2	2.40E-03	5.65E-01
ZNF782	zinc finger protein 782	2.40E-03	2.97E-01
SRR	serine racemase	2.41E-03	4.09E-01
COL13A1	collagen type XIII alpha 1 chain	2.41E-03	-2.95E-01
DCUN1D5	defective in cullin neddylation 1 domain containing 5	2.41E-03	6.61E-01
PNMA3	paraneoplastic Ma antigen 3	2.41E-03	8.40E-01
IFFO1	intermediate filament family orphan 1	2.41E-03	-6.86E-01
DDX24	DEAD-box helicase 24	2.41E-03	4.79E-01
PPP1R15B	protein phosphatase 1 regulatory subunit 15B	2.42E-03	9.07E-01
DDOST	dolichyl-diphosphooligosaccharide--protein glycosyltransferase non-catalytic subunit	2.42E-03	-5.56E-01
MAP3K3	mitogen-activated protein kinase kinase 3	2.42E-03	-3.05E-01

TTF1	transcription termination factor 1	2.43E-03	6.27E-01
YY1	YY1 transcription factor	2.43E-03	4.32E-01
ETNK1	ethanolamine kinase 1	2.43E-03	3.53E-01
RCOR2	REST corepressor 2	2.43E-03	-1.21
KHDRBS3	KH RNA binding domain containing, signal transduction associated 3	2.43E-03	2.92E-01
Mar-08	membrane associated ring-CH-type finger 8	2.44E-03	2.84E-01
WSB1	WD repeat and SOCS box containing 1	2.44E-03	-7.93E-01
SIX2	SIX homeobox 2	2.45E-03	-3.44E-01
SHMT2	serine hydroxymethyltransferase 2	2.45E-03	-2.84E-01
SSBP4	single stranded DNA binding protein 4	2.46E-03	-4.89E-01
CEP78	centrosomal protein 78	2.46E-03	7.04E-01
ADGRL2	adhesion G protein-coupled receptor L2	2.46E-03	1.08
SLF2	SMC5-SMC6 complex localization factor 2	2.46E-03	3.59E-01
C17orf58	chromosome 17 open reading frame 58	2.46E-03	-3.95E-01
HNRNPH3	heterogeneous nuclear ribonucleoprotein H3	2.47E-03	8.91E-01
GGA1	golgi associated, gamma adaptin ear containing, ARF binding protein 1	2.47E-03	-2.93E-01

MRPS21	mitochondrial ribosomal protein S21	2.47E-03	-7.29E-01
LRRC20	leucine rich repeat containing 20	2.47E-03	6.34E-01
GNAI2	G protein subunit alpha i2	2.48E-03	-5.01E-01
ZAK	sterile alpha motif and leucine zipper containing kinase AZK	2.48E-03	4.58E-01
ODF2	outer dense fiber of sperm tails 2	2.48E-03	2.53E-01
NR2C1	nuclear receptor subfamily 2 group C member 1	2.48E-03	3.94E-01
VBP1	VHL binding protein 1	2.48E-03	6.73E-01
PARP4	poly(ADP-ribose) polymerase family member 4	2.48E-03	5.96E-01
SLC25A3	solute carrier family 25 member 3	2.49E-03	-3.18E-01
HACE1	HECT domain and ankyrin repeat containing E3 ubiquitin protein ligase 1	2.49E-03	-3.31E-01
ELFN1	extracellular leucine rich repeat and fibronectin type III domain containing 1	2.50E-03	-2.84E-01
BZW1	basic leucine zipper and W2 domains 1	2.50E-03	9.43E-01
FCGR3A	Fc fragment of IgG receptor IIIa	2.50E-03	-4.33E-01
BRE	brain and reproductive organ-expressed (TNFRSF1A modulator)	2.50E-03	-3.32E-01
NCSTN	nicastrin	2.50E-03	-6.67E-01

CA5B	carbonic anhydrase 5B	2.51E-03	-4.12E-01
PTER	phosphotriesterase related	2.51E-03	3.06E-01
GEMIN5	gem nuclear organelle associated protein 5	2.51E-03	7.97E-01
ARL6IP1	ADP ribosylation factor like GTPase 6 interacting protein 1	2.52E-03	7.41E-01
TMEM38B	transmembrane protein 38B	2.52E-03	5.77E-01
RAP1GDS1	Rap1 GTPase-GDP dissociation stimulator 1	2.53E-03	8.46E-01
MAU2	MAU2 sister chromatid cohesion factor	2.54E-03	-7.41E-01
RPS8	ribosomal protein S8	2.54E-03	-4.15E-01
GALNT7	polypeptide N-acetylgalactosaminyltransferase 7	2.54E-03	7.12E-01
SIPA1L2	signal induced proliferation associated 1 like 2	2.54E-03	4.77E-01
ALG10B	ALG10B, alpha-1,2-glucosyltransferase	2.54E-03	3.28E-01
ECHDC1	ethylmalonyl-CoA decarboxylase 1	2.54E-03	4.97E-01
MBTD1	mbt domain containing 1	2.54E-03	-6.79E-01
DNTTIP1	deoxynucleotidyltransferase terminal interacting protein 1	2.54E-03	4.76E-01
RPS3	ribosomal protein S3	2.54E-03	-5.08E-01
RPAP3	RNA polymerase II associated protein 3	2.54E-03	4.53E-01

PRMT9	protein arginine methyltransferase 9	2.55E-03	-2.77E-01
CLK3	CDC like kinase 3	2.55E-03	-9.29E-01
CHIC1	cysteine rich hydrophobic domain 1	2.55E-03	3.00E-01
TXNRD1	thioredoxin reductase 1	2.56E-03	8.33E-01
SLC38A2	solute carrier family 38 member 2	2.56E-03	8.20E-01
FABP5	fatty acid binding protein 5	2.56E-03	5.85E-01
PRR5	proline rich 5	2.56E-03	-6.29E-01
TPBG	trophoblast glycoprotein	2.56E-03	3.87E-01
VANGL2	VANGL planar cell polarity protein 2	2.56E-03	-1.27
RAP2A	RAP2A, member of RAS oncogene family	2.56E-03	-9.22E-01
HLA-H	major histocompatibility complex, class I, H (pseudogene)	2.57E-03	3.46E-01
PSMB10	proteasome subunit beta 10	2.57E-03	-9.14E-01
PHLDB3	pleckstrin homology like domain family B member 3	2.58E-03	-3.22E-01
CMTM4	CKLF like MARVEL transmembrane domain containing 4	2.58E-03	-6.40E-01
KDM2B	lysine demethylase 2B	2.59E-03	-3.54E-01

SHMT1	serine hydroxymethyltransferase 1	2.59E-03	4.07E-01
TM2D3	TM2 domain containing 3	2.59E-03	-4.22E-01
PIGC	phosphatidylinositol glycan anchor biosynthesis class C	2.59E-03	-5.01E-01
DNAJB2	DnaJ heat shock protein family (Hsp40) member B2	2.60E-03	-3.17E-01
SAMM50	SAMM50 sorting and assembly machinery component	2.60E-03	-7.86E-01
EML2	echinoderm microtubule associated protein like 2	2.60E-03	-3.89E-01
LOC148413	uncharacterized LOC148413	2.60E-03	-9.15E-01
CA9	carbonic anhydrase 9	2.60E-03	-3.12E-01
ZKSCAN4	zinc finger with KRAB and SCAN domains 4	2.60E-03	-4.35E-01
CARMIL3	capping protein regulator and myosin 1 linker 3	2.61E-03	-2.80E-01
CEBPB	CCAAT/enhancer binding protein beta	2.61E-03	-5.87E-01
RFC1	replication factor C subunit 1	2.61E-03	5.20E-01
SGK223	homolog of rat pragma of Rnd2	2.61E-03	-3.42E-01

SCFD1	sec1 family domain containing 1	2.61E-03	3.14E-01
SFXN4	sideroflexin 4	2.62E-03	-9.93E-01
TRMT11	tRNA methyltransferase 11 homolog	2.62E-03	3.78E-01
CCDC25	coiled-coil domain containing 25	2.62E-03	-5.49E-01
C12orf65	chromosome 12 open reading frame 65	2.63E-03	8.43E-01
NIN	ninein	2.64E-03	4.35E-01
SMIM14	small integral membrane protein 14	2.64E-03	-6.61E-01
GPR3	G protein-coupled receptor 3	2.64E-03	3.65E-01
PROS1	protein S (alpha)	2.64E-03	-6.98E-01
TUBGCP5	tubulin gamma complex associated protein 5	2.64E-03	3.98E-01
SIK1	salt inducible kinase 1	2.64E-03	5.67E-01
CCT8	chaperonin containing TCP1 subunit 8	2.65E-03	5.48E-01
WASH3P	WAS protein family homolog 3 pseudogene	2.65E-03	-4.66E-01
DDX6	DEAD-box helicase 6	2.66E-03	3.15E-01
DNASE1L1	deoxyribonuclease 1 like 1	2.66E-03	-4.19E-01

PPIL1	peptidylprolyl isomerase like 1	2.66E-03	6.08E-01
ATG9A	autophagy related 9A	2.67E-03	-6.26E-01
SERINC3	serine incorporator 3	2.67E-03	-5.07E-01
E2F7	E2F transcription factor 7	2.67E-03	4.75E-01
CSF2RA	colony stimulating factor 2 receptor alpha subunit	2.67E-03	-3.03E-01
GNPNAT1	glucosamine-phosphate N-acetyltransferase 1	2.67E-03	-4.98E-01
GGCT	gamma-glutamylcyclotransferase	2.67E-03	6.77E-01
RAP1GDS1	Rap1 GTPase-GDP dissociation stimulator 1	2.67E-03	-6.29E-01
CHD7	chromodomain helicase DNA binding protein 7	2.67E-03	-5.52E-01
F3	coagulation factor III, tissue factor	2.68E-03	2.83E-01
TARBP2	TARBP2, RISC loading complex RNA binding subunit	2.68E-03	-7.00E-01
CTNNAL1	catenin alpha like 1	2.68E-03	7.48E-01
CEP83	centrosomal protein 83	2.68E-03	1.04
COMMD2	COMM domain containing 2	2.68E-03	6.75E-01
MAP3K3	mitogen-activated protein kinase kinase kinase 3	2.68E-03	-4.14E-01

CCR10	C-C motif chemokine receptor 10	2.69E-03	-6.88E-01
TMEM189	transmembrane protein 189	2.69E-03	-2.76E-01
ACSS2	acyl-CoA synthetase short-chain family member 2	2.69E-03	-4.82E-01
PDZD2	PDZ domain containing 2	2.69E-03	-4.75E-01
C1S	complement component 1, s subcomponent	2.69E-03	-3.43E-01
PEX16	peroxisomal biogenesis factor 16	2.70E-03	-7.84E-01
MED7	mediator complex subunit 7	2.70E-03	5.29E-01
TICRR	TOPBP1 interacting checkpoint and replication regulator	2.70E-03	5.69E-01
CERCAM	cerebral endothelial cell adhesion molecule	2.71E-03	-5.43E-01
HNRNPA1	heterogeneous nuclear ribonucleoprotein A1	2.71E-03	-8.06E-01
CDK6	cyclin dependent kinase 6	2.71E-03	-4.92E-01
LRRC23	leucine rich repeat containing 23	2.71E-03	-3.61E-01
DAZAP2	DAZ associated protein 2	2.72E-03	3.18E-01
DDX28	DEAD-box helicase 28	2.73E-03	3.65E-01

SLC38A1	solute carrier family 38 member 1	2.73E-03	1.01
ARSA	arylsulfatase A	2.73E-03	-4.63E-01
FAAP100	Fanconi anemia core complex associated protein 100	2.74E-03	-7.53E-01
TRIM28	tripartite motif containing 28	2.74E-03	4.17E-01
SLC2A12	solute carrier family 2 member 12	2.75E-03	-4.80E-01
LZTR1	leucine zipper like transcription regulator 1	2.75E-03	-7.68E-01
MGST1	microsomal glutathione S-transferase 1	2.75E-03	4.36E-01
C7orf49	chromosome 7 open reading frame 49	2.75E-03	5.14E-01
SPECC1L	sperm antigen with calponin homology and coiled-coil domains 1 like	2.75E-03	-4.12E-01
CCDC184	coiled-coil domain containing 184	2.75E-03	-2.97E-01
FAM149A	family with sequence similarity 149 member A	2.76E-03	-2.97E-01
ZNF823	zinc finger protein 823	2.76E-03	4.65E-01
VSIG10	V-set and immunoglobulin domain containing 10	2.76E-03	2.87E-01
GART	phosphoribosylglycinamide formyltransferase, phosphoribosylglycinamide synthetase, phosphoribosylaminoimidazole synthetase	2.76E-03	7.11E-01
RTBDN	retbindin	2.76E-03	-2.69E-01

OAZ1	ornithine decarboxylase antizyme 1	2.76E-03	3.71E-01
RMND5B	required for meiotic nuclear division 5 homolog B	2.76E-03	-3.33E-01
PFKM	phosphofructokinase, muscle	2.77E-03	-7.23E-01
ERCC8	ERCC excision repair 8, CSA ubiquitin ligase complex subunit	2.77E-03	3.41E-01
UBE2W	ubiquitin conjugating enzyme E2 W (putative)	2.77E-03	7.34E-01
DEAF1	DEAF1, transcription factor	2.77E-03	-3.28E-01
SPANXA1	sperm protein associated with the nucleus, X-linked, family member A1	2.78E-03	3.05E-01
SPG7	SPG7, paraplegin matrix AAA peptidase subunit	2.78E-03	-7.78E-01
COL5A2	collagen type V alpha 2 chain	2.78E-03	7.34E-01
SMC3	structural maintenance of chromosomes 3	2.79E-03	8.67E-01
PTBP3	polypyrimidine tract binding protein 3	2.79E-03	-6.28E-01
MRPS34	mitochondrial ribosomal protein S34	2.79E-03	-7.08E-01
WDR74	WD repeat domain 74	2.80E-03	-5.54E-01
RBM18	RNA binding motif protein 18	2.80E-03	4.38E-01
PIGH	phosphatidylinositol glycan anchor biosynthesis class H	2.80E-03	-3.88E-01

MOXD1	monooxygenase DBH like 1	2.80E-03	3.21E-01
ACTG1P4	actin gamma 1 pseudogene 4	2.80E-03	8.65E-01
NTF4	neurotrophin 4	2.81E-03	-4.29E-01
ZNF443	zinc finger protein 443	2.81E-03	2.56E-01
LOC100134868	uncharacterized LOC100134868	2.82E-03	-7.63E-01
SCAF11	SR-related CTD associated factor 11	2.82E-03	5.64E-01
DUSP6	dual specificity phosphatase 6	2.82E-03	1.05
DHX40	DEAH-box helicase 40	2.82E-03	-7.69E-01
SMYD4	SET and MYND domain containing 4	2.83E-03	4.02E-01
PTPN13	protein tyrosine phosphatase, non-receptor type 13	2.83E-03	-6.91E-01
DNAJC2	DnaJ heat shock protein family (Hsp40) member C2	2.84E-03	1.02
EIF3K	eukaryotic translation initiation factor 3 subunit K	2.84E-03	-5.15E-01
ADRM1	adhesion regulating molecule 1	2.84E-03	3.76E-01
TNPO1	transportin 1	2.86E-03	-1.01
BORCS7	BLOC-1 related complex subunit 7	2.86E-03	-9.35E-01
CBLC	Cbl proto-oncogene C	2.86E-03	2.48E-01

PMEL	premelanosome protein	2.86E-03	-2.84E-01
PTK7	protein tyrosine kinase 7 (inactive)	2.86E-03	-8.07E-01
SLC38A10	solute carrier family 38 member 10	2.86E-03	-6.04E-01
TYROBP	TYRO protein tyrosine kinase binding protein	2.87E-03	-9.24E-01
BOK	BOK, BCL2 family apoptosis regulator	2.87E-03	4.24E-01
ZNF735	zinc finger protein 735	2.87E-03	1.63
CCDC173	coiled-coil domain containing 173	2.87E-03	2.78E-01
UPF3B	UPF3 regulator of nonsense transcripts homolog B (yeast)	2.88E-03	4.05E-01
PDP2	pyruvate dehyrogenase phosphatase catalytic subunit 2	2.88E-03	-4.91E-01
CDK10	cyclin dependent kinase 10	2.88E-03	-5.50E-01
VHL	von Hippel-Lindau tumor suppressor	2.88E-03	-6.92E-01
GARS	glycyl-tRNA synthetase	2.89E-03	4.10E-01
HSD17B8	hydroxysteroid 17-beta dehydrogenase 8	2.89E-03	-9.85E-01
KDM4B	lysine demethylase 4B	2.90E-03	-5.92E-01
ITGB5	integrin subunit beta 5	2.90E-03	4.88E-01

GOLM1	golgi membrane protein 1	2.90E-03	2.65E-01
PF4V1	platelet factor 4 variant 1	2.90E-03	2.40E-01
AHCYL2	adenosylhomocysteinase like 2	2.90E-03	-4.25E-01
PRKRIP1	PRKR interacting protein 1 (IL11 inducible)	2.90E-03	-6.32E-01
MPST	mercaptopyruvate sulfurtransferase	2.90E-03	-9.48E-01
PIK3R1	phosphoinositide-3-kinase regulatory subunit 1	2.90E-03	-8.82E-01
TNFAIP3	TNF alpha induced protein 3	2.91E-03	2.66E-01
PIK3CA	phosphatidylinositol-4,5-bisphosphate 3-kinase catalytic subunit alpha	2.91E-03	6.74E-01
SVIP	small VCP interacting protein	2.91E-03	3.35E-01
NUDT15	nudix hydrolase 15	2.91E-03	3.27E-01
BTNL10	butyrophilin like 10	2.92E-03	-6.05E-01
CRYZ	crystallin zeta	2.92E-03	-4.05E-01
NGDN	neuroguidin	2.92E-03	6.00E-01
AAMDC	adipogenesis associated Mth938 domain containing	2.92E-03	-1.07
LOC730101	uncharacterized LOC730101	2.92E-03	2.53E-01

SHD	Src homology 2 domain containing transforming protein D	2.92E-03	-2.30E-01
ANAPC1	anaphase promoting complex subunit 1	2.93E-03	4.86E-01
XRCC2	X-ray repair cross complementing 2	2.93E-03	4.27E-01
ABL1	ABL proto-oncogene 1, non-receptor tyrosine kinase	2.93E-03	3.92E-01
P4HA2	prolyl 4-hydroxylase subunit alpha 2	2.93E-03	-6.13E-01
UBE2Q2P2	ubiquitin conjugating enzyme E2 Q2 pseudogene 2	2.94E-03	-2.96E-01
TBC1D8B	TBC1 domain family member 8B	2.94E-03	-4.72E-01
WDR12	WD repeat domain 12	2.94E-03	4.95E-01
THOC7	THO complex 7	2.94E-03	4.78E-01
INO80E	INO80 complex subunit E	2.94E-03	6.16E-01
METTL23	methyltransferase like 23	2.94E-03	-8.62E-01
C9orf106	chromosome 9 open reading frame 106	2.94E-03	-2.81E-01
SNORD25	small nucleolar RNA, C/D box 25	2.94E-03	2.94E-01
GBA2	glucosylceramidase beta 2	2.95E-03	-3.82E-01
GEN1	GEN1, Holliday junction 5' flap endonuclease	2.97E-03	4.80E-01

SPN	sialophorin	2.97E-03	-6.72E-01
TM9SF1	transmembrane 9 superfamily member 1	2.98E-03	-4.57E-01
ALG10	ALG10, alpha-1,2-glucosyltransferase	2.98E-03	4.01E-01
STX5	syntaxin 5	2.98E-03	-4.13E-01
TM9SF1	transmembrane 9 superfamily member 1	2.98E-03	-5.67E-01
PPP1R13L	protein phosphatase 1 regulatory subunit 13 like	2.99E-03	4.40E-01
PLEC	plectin	2.99E-03	2.83E-01
COLEC12	collectin subfamily member 12	2.99E-03	-2.68E-01
LTV1	LTV1 ribosome biogenesis factor	2.99E-03	5.79E-01
ASB3	ankyrin repeat and SOCS box containing 3	2.99E-03	-5.70E-01
ZNRF2P1	zinc and ring finger 2 pseudogene 1	2.99E-03	2.97E-01
IFI27L1	interferon alpha inducible protein 27 like 1	3.00E-03	2.78E-01
CCDC113	coiled-coil domain containing 113	3.00E-03	3.86E-01
ALG10B	ALG10B, alpha-1,2-glucosyltransferase	3.00E-03	7.36E-01
TMEM187	transmembrane protein 187	3.00E-03	5.92E-01

ZNF207	zinc finger protein 207	3.00E-03	4.55E-01
RIOK2	RIO kinase 2	3.00E-03	6.15E-01
TMEM232	transmembrane protein 232	3.00E-03	2.27E-01
AFF4	AF4/FMR2 family member 4	3.01E-03	-8.94E-01
KYAT1	kynurenine aminotransferase 1	3.01E-03	-5.04E-01
BCL2L12	BCL2 like 12	3.01E-03	9.55E-01
TOP1MT	topoisomerase (DNA) I, mitochondrial	3.01E-03	-6.51E-01
SNAPC1	small nuclear RNA activating complex polypeptide 1	3.01E-03	-7.41E-01
ANKRD52	ankyrin repeat domain 52	3.02E-03	3.02E-01
LSP1	lymphocyte-specific protein 1	3.02E-03	6.18E-01
SGPL1	sphingosine-1-phosphate lyase 1	3.02E-03	3.29E-01
MRC2	mannose receptor C type 2	3.02E-03	-3.41E-01
TUB	tubby bipartite transcription factor	3.03E-03	6.04E-01
TEX11	testis expressed 11	3.03E-03	-5.61E-01
RRS1	ribosome biogenesis regulator homolog	3.03E-03	5.48E-01

HNRNPF	heterogeneous nuclear ribonucleoprotein F	3.03E-03	4.09E-01
USP49	ubiquitin specific peptidase 49	3.03E-03	-6.28E-01
NCOR2	nuclear receptor corepressor 2	3.04E-03	-1.05
FRRS1	ferric chelate reductase 1	3.04E-03	-3.08E-01
ALDH4A1	aldehyde dehydrogenase 4 family member A1	3.04E-03	-6.51E-01
PSMD2	proteasome 26S subunit, non-ATPase 2	3.05E-03	6.31E-01
PRKD3	protein kinase D3	3.05E-03	-6.57E-01
CT45A5	cancer/testis antigen family 45, member A5	3.05E-03	3.49E-01
FHIT	fragile histidine triad	3.05E-03	-4.50E-01
FAM110A	family with sequence similarity 110 member A	3.05E-03	2.92E-01
SNX7	sorting nexin 7	3.05E-03	2.42E-01
FCGBP	Fc fragment of IgG binding protein	3.06E-03	-3.17E-01
ARID3B	AT-rich interaction domain 3B	3.06E-03	-7.84E-01
NEO1	neogenin 1	3.07E-03	3.15E-01
LETMD1	LETM1 domain containing 1	3.07E-03	-5.67E-01

MARS2	methionyl-tRNA synthetase 2, mitochondrial	3.07E-03	7.56E-01
CENPO	centromere protein O	3.09E-03	3.69E-01
PI4KB	phosphatidylinositol 4-kinase beta	3.09E-03	-6.91E-01
BTBD7	BTB domain containing 7	3.09E-03	3.23E-01
LIPG	lipase G, endothelial type	3.09E-03	-4.50E-01
RHBDF1	rhomboid 5 homolog 1	3.09E-03	-1.03
FAT1	FAT atypical cadherin 1	3.09E-03	-1.14
ADGRB1	adhesion G protein-coupled receptor B1	3.09E-03	-2.33E-01
BMP8B	bone morphogenetic protein 8b	3.10E-03	-2.67E-01
FOXK1	forkhead box K1	3.10E-03	2.22E-01
CTPS2	CTP synthase 2	3.11E-03	6.61E-01
TINF2	TERF1 interacting nuclear factor 2	3.11E-03	-6.55E-01
TUBGCP4	tubulin gamma complex associated protein 4	3.11E-03	7.77E-01
ARHGEF17	Rho guanine nucleotide exchange factor 17	3.11E-03	-5.39E-01
ADAMTSL5	ADAMTS like 5	3.12E-03	-4.44E-01

SPRY4	sprouty RTK signaling antagonist 4	3.12E-03	-5.17E-01
PAK1IP1	PAK1 interacting protein 1	3.12E-03	5.71E-01
NMT2	N-myristoyltransferase 2	3.13E-03	3.82E-01
CCDC88B	coiled-coil domain containing 88B	3.14E-03	-5.00E-01
GLOD4	glyoxalase domain containing 4	3.14E-03	3.11E-01
CFL2	cofilin 2	3.14E-03	5.41E-01
ACYP1	acylphosphatase 1	3.14E-03	9.94E-01
ASPHD2	aspartate beta-hydroxylase domain containing 2	3.15E-03	3.18E-01
SLC46A1	solute carrier family 46 member 1	3.15E-03	-4.43E-01
CEP41	centrosomal protein 41	3.15E-03	3.78E-01
C6orf99	chromosome 6 open reading frame 99	3.15E-03	2.34E-01
TWSG1	twisted gastrulation BMP signaling modulator 1	3.15E-03	4.45E-01
LOC644936	actin, beta pseudogene	3.15E-03	1.21
CCT7	chaperonin containing TCP1 subunit 7	3.15E-03	4.11E-01
RABL2A	RAB, member of RAS oncogene family-like 2A	3.15E-03	-4.14E-01
TSPYL1	TSPY like 1	3.17E-03	-3.14E-01

LGALS9B	galectin 9B	3.17E-03	4.00E-01
RPS6KA4	ribosomal protein S6 kinase A4	3.17E-03	5.74E-01
ZDHHC9	zinc finger DHHC-type containing 9	3.18E-03	-1.11
ZNF252P	zinc finger protein 252, pseudogene	3.18E-03	-4.15E-01
TBKBP1	TBK1 binding protein 1	3.18E-03	-5.18E-01
STX16	syntaxin 16	3.19E-03	-9.15E-01
RPL17	ribosomal protein L17	3.19E-03	-7.15E-01
HSPA8	heat shock protein family A (Hsp70) member 8	3.19E-03	6.16E-01
SHMT1	serine hydroxymethyltransferase 1	3.19E-03	4.44E-01
PDE4B	phosphodiesterase 4B	3.20E-03	3.18E-01
SNORA61	small nucleolar RNA, H/ACA box 61	3.20E-03	-6.46E-01
ZNF146	zinc finger protein 146	3.20E-03	5.20E-01
NDST3	N-deacetylase and N-sulfotransferase 3	3.21E-03	-3.39E-01
FAM129A	family with sequence similarity 129 member A	3.21E-03	7.17E-01
KDELR3	KDEL endoplasmic reticulum protein retention receptor 3	3.22E-03	-4.84E-01

PUS7	pseudouridylate synthase 7 (putative)	3.22E-03	4.00E-01
SCRIB	scribbled planar cell polarity protein	3.22E-03	-2.87E-01
ORC5	origin recognition complex subunit 5	3.22E-03	6.42E-01
MLXIPL	MLX interacting protein like	3.23E-03	-5.56E-01
ADAM17	ADAM metallopeptidase domain 17	3.23E-03	-4.73E-01
NABP2	nucleic acid binding protein 2	3.23E-03	6.38E-01
PGGT1B	protein geranylgeranyltransferase type I subunit beta	3.24E-03	6.28E-01
AGPAT2	1-acylglycerol-3-phosphate O-acyltransferase 2	3.24E-03	-5.88E-01
LETM1	leucine zipper and EF-hand containing transmembrane protein 1	3.24E-03	5.39E-01
BTN2A1	butyrophilin subfamily 2 member A1	3.24E-03	5.29E-01
NCAPH	non-SMC condensin I complex subunit H	3.25E-03	2.56E-01
RAB11FIP1	RAB11 family interacting protein 1	3.25E-03	-4.34E-01
TFIP11	tuftelin interacting protein 11	3.25E-03	4.55E-01
MAPRE2	microtubule associated protein RP/EB family member 2	3.25E-03	5.11E-01
PPIA	peptidylprolyl isomerase A	3.25E-03	-7.76E-01

CELF5	CUGBP, Elav-like family member 5	3.25E-03	-4.56E-01
MEST	mesoderm specific transcript	3.25E-03	-2.58E-01
NPEPPS	aminopeptidase puromycin sensitive	3.25E-03	-7.23E-01
FKBP2	FK506 binding protein 2	3.26E-03	-9.18E-01
C9orf116	chromosome 9 open reading frame 116	3.26E-03	4.69E-01
PJA2	praja ring finger ubiquitin ligase 2	3.26E-03	-5.30E-01
SERF2	small EDRK-rich factor 2	3.26E-03	-6.60E-01
ILF2	interleukin enhancer binding factor 2	3.26E-03	5.54E-01
PNPLA8	patatin like phospholipase domain containing 8	3.27E-03	4.45E-01
SLC22A17	solute carrier family 22 member 17	3.27E-03	-6.11E-01
MRPL51	mitochondrial ribosomal protein L51	3.27E-03	4.27E-01
HIST2H2AA3	histone cluster 2, H2aa3	3.27E-03	-8.59E-01
POLR2H	RNA polymerase II subunit H	3.28E-03	-3.06E-01
AMDHD1	amidohydrolase domain containing 1	3.28E-03	2.76E-01

TUBG2	tubulin gamma 2	3.29E-03	-5.67E-01
UBE2W	ubiquitin conjugating enzyme E2 W (putative)	3.29E-03	7.38E-01
SOX13	SRY-box 13	3.29E-03	3.91E-01
CLUAP1	clusterin associated protein 1	3.29E-03	4.72E-01
PI4K2B	phosphatidylinositol 4-kinase type 2 beta	3.30E-03	4.26E-01
LAMTOR3	late endosomal/lysosomal adaptor, MAPK and MTOR activator 3	3.30E-03	-2.63E-01
ZNF319	zinc finger protein 319	3.30E-03	2.45E-01
UTP23	UTP23, small subunit processome component	3.30E-03	5.60E-01
RNASEH2C	ribonuclease H2 subunit C	3.30E-03	3.11E-01
DNM1	dynamin 1	3.31E-03	-2.69E-01
RAD9A	RAD9 checkpoint clamp component A	3.31E-03	2.98E-01
NPIPB5	nuclear pore complex interacting protein family member B5	3.32E-03	-4.99E-01
SNX7	sorting nexin 7	3.32E-03	8.60E-01
VRK2	vaccinia related kinase 2	3.33E-03	7.49E-01
NEK8	NIMA related kinase 8	3.33E-03	-4.44E-01
RIF1	replication timing regulatory factor 1	3.33E-03	5.53E-01

MIR216A	microRNA 216a	3.33E-03	-2.89E-01
KIF1B	kinesin family member 1B	3.33E-03	-1.06
ZNF679	zinc finger protein 679	3.34E-03	1.27
LRRC29	leucine rich repeat containing 29	3.34E-03	-4.57E-01
MCM3AP	minichromosome maintenance complex component 3 associated protein	3.34E-03	-7.27E-01
SELT	selenoprotein T	3.34E-03	4.14E-01
RN7SK	RNA, 7SK small nuclear	3.34E-03	-7.60E-01
GK5	glycerol kinase 5 (putative)	3.34E-03	6.18E-01
RNF125	ring finger protein 125	3.35E-03	3.02E-01
SPECC1	sperm antigen with calponin homology and coiled-coil domains 1	3.35E-03	2.78E-01
KLHL29	kelch like family member 29	3.35E-03	-5.80E-01
CD2AP	CD2 associated protein	3.36E-03	9.28E-01
SRGN	serglycin	3.36E-03	2.46E-01
VCAM1	vascular cell adhesion molecule 1	3.36E-03	-2.35E-01
INSL3	insulin like 3	3.37E-03	-4.32E-01

ADGRA2	adhesion G protein-coupled receptor A2	3.37E-03	-7.96E-01
TMEM14B	transmembrane protein 14B	3.37E-03	2.32E-01
PDK3	pyruvate dehydrogenase kinase 3	3.37E-03	4.50E-01
FAM86B3P	family with sequence similarity 86, member A pseudogene	3.40E-03	3.44E-01
MLXIPL	MLX interacting protein like	3.40E-03	-3.98E-01
AP2A1	adaptor related protein complex 2 alpha 1 subunit	3.40E-03	-6.36E-01
DNAJB6	DnaJ heat shock protein family (Hsp40) member B6	3.40E-03	-8.07E-01
TMEM231	transmembrane protein 231	3.40E-03	3.58E-01
FAXC	failed axon connections homolog	3.41E-03	2.81E-01
ACO2	aconitase 2	3.41E-03	-6.28E-01
MPC2	mitochondrial pyruvate carrier 2	3.42E-03	4.68E-01
NIN	ninein	3.42E-03	3.67E-01
UQCR10	ubiquinol-cytochrome c reductase, complex III subunit X	3.43E-03	-8.23E-01
UBE2NL	ubiquitin conjugating enzyme E2 N like (gene/pseudogene)	3.43E-03	4.09E-01
POLA1	DNA polymerase alpha 1, catalytic subunit	3.43E-03	2.26E-01

DIAPH3	diaphanous related formin 3	3.43E-03	2.99E-01
WWP2	WW domain containing E3 ubiquitin protein ligase 2	3.44E-03	4.06E-01
GLB1L2	galactosidase beta 1 like 2	3.44E-03	3.99E-01
SLC16A5	solute carrier family 16 member 5	3.44E-03	-2.31E-01
EHD4	EH domain containing 4	3.44E-03	4.68E-01
CLN8	ceroid-lipofuscinosis, neuronal 8	3.44E-03	-4.46E-01
DENND2C	DENN domain containing 2C	3.44E-03	3.11E-01
MOB1A	MOB kinase activator 1A	3.45E-03	8.18E-01
GLDC	glycine decarboxylase	3.45E-03	-7.87E-01
PNISR	PNN interacting serine and arginine rich protein	3.45E-03	-8.36E-01
TMEM59L	transmembrane protein 59 like	3.45E-03	-5.24E-01
SNORA50C	small nucleolar RNA, H/ACA box 50C	3.45E-03	-6.08E-01
CHST13	carbohydrate sulfotransferase 13	3.45E-03	5.42E-01
NOL8	nucleolar protein 8	3.45E-03	3.36E-01
TRAF7	TNF receptor associated factor 7	3.46E-03	-3.95E-01

ICAM5	intercellular adhesion molecule 5	3.46E-03	-4.85E-01
HLA-DRB4	major histocompatibility complex, class II, DR beta 4	3.46E-03	-6.56E-01
SPHK1	sphingosine kinase 1	3.46E-03	3.67E-01
BRI3BP	BRI3 binding protein	3.46E-03	7.65E-01
MTPN	myotrophin	3.46E-03	6.93E-01
KDM6B	lysine demethylase 6B	3.47E-03	-3.40E-01
MAPKAPK5	mitogen-activated protein kinase-activated protein kinase 5	3.47E-03	5.43E-01
EPB41L2	erythrocyte membrane protein band 4.1 like 2	3.47E-03	6.96E-01
SDHC	succinate dehydrogenase complex subunit C	3.47E-03	-5.52E-01
PDZK1	PDZ domain containing 1	3.48E-03	2.76E-01
DDX17	DEAD-box helicase 17	3.48E-03	-7.87E-01
ACP1	acid phosphatase 1, soluble	3.48E-03	-3.92E-01
BICC1	BicC family RNA binding protein 1	3.48E-03	2.89E-01
DEF8	differentially expressed in FDCP 8 homolog	3.49E-03	7.95E-01
SLC25A40	solute carrier family 25 member 40	3.49E-03	3.90E-01

DOK6	docking protein 6	3.49E-03	2.63E-01
CCDC14	coiled-coil domain containing 14	3.50E-03	9.57E-01
WDR48	WD repeat domain 48	3.50E-03	-5.49E-01
MAN1B1	mannosidase alpha class 1B member 1	3.50E-03	-5.20E-01
DLG4	discs large MAGUK scaffold protein 4	3.51E-03	-6.58E-01
NME3	NME/NM23 nucleoside diphosphate kinase 3	3.51E-03	-7.24E-01
PHF19	PHD finger protein 19	3.51E-03	8.71E-01
IFITM1	interferon induced transmembrane protein 1	3.52E-03	9.98E-01
MRPL38	mitochondrial ribosomal protein L38	3.52E-03	-2.90E-01
GPX8	glutathione peroxidase 8 (putative)	3.54E-03	-7.55E-01
DHFR2	dihydrofolate reductase 2	3.54E-03	-3.64E-01
HCFC1R1	host cell factor C1 regulator 1	3.54E-03	-5.96E-01
KRBA2	KRAB-A domain containing 2	3.54E-03	-2.57E-01
SIDT2	SID1 transmembrane family member 2	3.54E-03	-8.10E-01

AP1M1	adaptor related protein complex 1 mu 1 subunit	3.55E-03	-7.76E-01
PSMA4	proteasome subunit alpha 4	3.55E-03	5.78E-01
ANKRD11	ankyrin repeat domain 11	3.55E-03	-7.14E-01
SCAMP1	secretory carrier membrane protein 1	3.56E-03	-5.02E-01
CIDEC	cell death inducing DFFA like effector c	3.56E-03	2.54E-01
ODF2L	outer dense fiber of sperm tails 2 like	3.56E-03	4.46E-01
TRIML2	tripartite motif family like 2	3.56E-03	-4.05E-01
WHSC1	Wolf-Hirschhorn syndrome candidate 1	3.56E-03	4.27E-01
FBLN5	fibulin 5	3.57E-03	-5.22E-01
STBD1	starch binding domain 1	3.58E-03	3.63E-01
SYCP3	synaptonemal complex protein 3	3.58E-03	-2.88E-01
ZBED9	zinc finger BED-type containing 9	3.59E-03	6.70E-01
THAP1	THAP domain containing 1	3.59E-03	3.93E-01
IDS	iduronate 2-sulfatase	3.60E-03	-3.40E-01
PHC3	polyhomeotic homolog 3	3.60E-03	5.22E-01

SH3PXD2A	SH3 and PX domains 2A	3.60E-03	-5.64E-01
WDR62	WD repeat domain 62	3.61E-03	3.16E-01
UBB	ubiquitin B	3.61E-03	4.12E-01
NDUFC1	NADH:ubiquinone oxidoreductase subunit C1	3.62E-03	4.97E-01
DERL1	derlin 1	3.62E-03	5.64E-01
ZNF425	zinc finger protein 425	3.62E-03	-4.00E-01
SLTM	SAFB like transcription modulator	3.62E-03	-7.59E-01
VEGFA	vascular endothelial growth factor A	3.62E-03	-6.38E-01
MYH9	myosin, heavy chain 9, non-muscle	3.62E-03	9.29E-01
STX7	syntaxin 7	3.62E-03	4.77E-01
ZMYND19	zinc finger MYND-type containing 19	3.62E-03	9.08E-01
RAB7A	RAB7A, member RAS oncogene family	3.63E-03	-2.55E-01
SRRM2	serine/arginine repetitive matrix 2	3.63E-03	-5.31E-01
RARB	retinoic acid receptor beta	3.64E-03	2.39E-01
TRIM13	tripartite motif containing 13	3.65E-03	-4.50E-01

PLPP3	phospholipid phosphatase 3	3.65E-03	2.64E-01
SLC22A5	solute carrier family 22 member 5	3.65E-03	4.71E-01
RGMA	repulsive guidance molecule family member a	3.65E-03	-2.45E-01
IDNK	IDNK, gluconokinase	3.65E-03	5.25E-01
GUSBP11	glucuronidase, beta pseudogene 11	3.66E-03	-3.09E-01
HOXC6	homeobox C6	3.66E-03	-6.59E-01
ITGB5	integrin subunit beta 5	3.67E-03	4.71E-01
OTUD6B	OTU domain containing 6B	3.67E-03	8.17E-01
TIMMDC1	translocase of inner mitochondrial membrane domain containing 1	3.67E-03	-4.95E-01
KIF13B	kinesin family member 13B	3.67E-03	3.83E-01
CDH12	cadherin 12	3.67E-03	-3.33E-01
ZNF223	zinc finger protein 223	3.68E-03	-6.77E-01
HDAC2	histone deacetylase 2	3.68E-03	5.73E-01
PPM1N	protein phosphatase, Mg2+/Mn2+ dependent 1N (putative)	3.68E-03	-5.75E-01
LEMD3	LEM domain containing 3	3.68E-03	-6.95E-01

NFATC1	nuclear factor of activated T-cells 1	3.69E-03	-3.10E-01
CTSL	cathepsin L	3.69E-03	-5.13E-01
PGGT1B	protein geranylgeranyltransferase type I subunit beta	3.69E-03	-3.75E-01
OAT	ornithine aminotransferase	3.69E-03	5.53E-01
MYO9B	myosin IXB	3.69E-03	-7.12E-01
ZNF416	zinc finger protein 416	3.70E-03	4.48E-01
WRNIP1	Werner helicase interacting protein 1	3.70E-03	7.37E-01
SOHLH2	spermatogenesis and oogenesis specific basic helix-loop-helix 2	3.70E-03	3.05E-01
SUPV3L1	Suv3 like RNA helicase	3.70E-03	2.96E-01
TDRD1	tudor domain containing 1	3.70E-03	-7.09E-01
FBXO3	F-box protein 3	3.70E-03	5.50E-01
HIST1H2AC	histone cluster 1, H2ac	3.71E-03	-9.74E-01
ADGRB3	adhesion G protein-coupled receptor B3	3.71E-03	3.46E-01
TMEM179B	transmembrane protein 179B	3.72E-03	-7.77E-01
RARA	retinoic acid receptor alpha	3.72E-03	-8.49E-01

SMDT1	single-pass membrane protein with aspartate rich tail 1	3.72E-03	-3.89E-01
ELL	elongation factor for RNA polymerase II	3.72E-03	3.41E-01
FILIP1L	filamin A interacting protein 1 like	3.73E-03	-3.54E-01
GPATCH4	G-patch domain containing 4	3.73E-03	6.39E-01
TTC33	tetratricopeptide repeat domain 33	3.73E-03	7.50E-01
ZBED1	zinc finger BED-type containing 1	3.74E-03	-4.95E-01
FEZ2	fasciculation and elongation protein zeta 2	3.74E-03	-5.74E-01
PFN1	profilin 1	3.74E-03	4.09E-01
PSPH	phosphoserine phosphatase	3.75E-03	-6.90E-01
ZFPM2	zinc finger protein, FOG family member 2	3.75E-03	-7.85E-01
RNF11	ring finger protein 11	3.76E-03	3.36E-01
ZDHHC4	zinc finger DHHC-type containing 4	3.76E-03	-4.35E-01
NFYC	nuclear transcription factor Y subunit gamma	3.76E-03	3.02E-01
UQCC1	ubiquinol-cytochrome c reductase complex assembly factor 1	3.77E-03	-5.02E-01

PPIB	peptidylprolyl isomerase B	3.77E-03	-8.07E-01
MAT2B	methionine adenosyltransferase 2B	3.77E-03	9.58E-01
MEX3D	mex-3 RNA binding family member D	3.78E-03	4.87E-01
UBE2V2	ubiquitin conjugating enzyme E2 V2	3.79E-03	3.86E-01
PPFIA1	PTPRF interacting protein alpha 1	3.79E-03	4.55E-01
NF2	neurofibromin 2	3.80E-03	3.22E-01
CMTM4	CKLF like MARVEL transmembrane domain containing 4	3.80E-03	-9.23E-01
CNOT7	CCR4-NOT transcription complex subunit 7	3.80E-03	9.40E-01
AGPAT2	1-acylglycerol-3-phosphate O-acyltransferase 2	3.81E-03	-3.21E-01
OAT	ornithine aminotransferase	3.81E-03	3.42E-01
ZNF34	zinc finger protein 34	3.82E-03	2.48E-01
GPD2	glycerol-3-phosphate dehydrogenase 2	3.82E-03	8.22E-01
LIX1L	limb and CNS expressed 1 like	3.82E-03	9.42E-01
GRM4	glutamate metabotropic receptor 4	3.82E-03	-2.25E-01
ITGA1	integrin subunit alpha 1	3.83E-03	2.32E-01
NMRK1	nicotinamide riboside kinase 1	3.83E-03	4.34E-01

CPT1C	carnitine palmitoyltransferase 1C	3.84E-03	-8.54E-01
MIR99AHG	mir-99a-let-7c cluster host gene	3.84E-03	-5.81E-01
ARHGAP17	Rho GTPase activating protein 17	3.85E-03	6.77E-01
SRRM2-AS1	SRRM2 antisense RNA 1	3.85E-03	-2.62E-01
CAPN1	calpain 1	3.86E-03	-1.02
NDUFB1	NADH:ubiquinone oxidoreductase subunit B1	3.87E-03	-6.53E-01
METTL2B	methyltransferase like 2B	3.87E-03	3.74E-01
PCYOX1	prenylcysteine oxidase 1	3.88E-03	-7.72E-01
EML4	echinoderm microtubule associated protein like 4	3.88E-03	8.99E-01
RFXAP	regulatory factor X associated protein	3.89E-03	3.62E-01
MAEL	maelstrom spermatogenic transposon silencer	3.89E-03	-2.93E-01
REV1	REV1, DNA directed polymerase	3.89E-03	-4.12E-01
CBWD1	COBW domain containing 1	3.89E-03	9.41E-01
ANKRD46	ankyrin repeat domain 46	3.89E-03	-3.28E-01
HIKESHI	Hikeshi, heat shock protein nuclear import factor	3.90E-03	5.23E-01

TUBA3FP	tubulin alpha 3f pseudogene	3.90E-03	2.85E-01
MECR	mitochondrial trans-2-enoyl-CoA reductase	3.90E-03	2.87E-01
SDHA	succinate dehydrogenase complex flavoprotein subunit A	3.90E-03	-4.70E-01
PSMD3	proteasome 26S subunit, non-ATPase 3	3.91E-03	3.46E-01
SLC6A8	solute carrier family 6 member 8	3.91E-03	-3.31E-01
DCN	decorin	3.91E-03	-6.20E-01
TNC	tenascin C	3.92E-03	-3.64E-01
UTF1	undifferentiated embryonic cell transcription factor 1	3.92E-03	-2.79E-01
MOSPD1	motile sperm domain containing 1	3.92E-03	3.36E-01
SEC22C	SEC22 homolog C, vesicle trafficking protein	3.94E-03	4.84E-01
RSAD1	radical S-adenosyl methionine domain containing 1	3.94E-03	-9.00E-01
YBEY	ybeY metallopeptidase (putative)	3.94E-03	-9.23E-01
S100A13	S100 calcium binding protein A13	3.95E-03	-6.70E-01
ZC3H14	zinc finger CCCH-type containing 14	3.95E-03	6.55E-01
ZDHHC14	zinc finger DHHC-type containing 14	3.95E-03	3.59E-01

LCLAT1	lysocardiolipin acyltransferase 1	3.95E-03	8.31E-01
ABHD4	abhydrolase domain containing 4	3.95E-03	-5.64E-01
OGDH	oxoglutarate dehydrogenase	3.96E-03	3.49E-01
SLC35A1	solute carrier family 35 member A1	3.96E-03	-3.56E-01
RAD18	RAD18, E3 ubiquitin protein ligase	3.96E-03	3.35E-01
TSEN15	tRNA splicing endonuclease subunit 15	3.96E-03	6.48E-01
PLPPR5	phospholipid phosphatase related 5	3.96E-03	-6.75E-01
CPEB3	cytoplasmic polyadenylation element binding protein 3	3.96E-03	-4.54E-01
FEM1B	fem-1 homolog B	3.97E-03	2.94E-01
FAM72B	family with sequence similarity 72 member B	3.97E-03	7.04E-01
SKP2	S-phase kinase-associated protein 2, E3 ubiquitin protein ligase	3.97E-03	5.37E-01
TMEM259	transmembrane protein 259	3.97E-03	-6.53E-01
ACBD4	acyl-CoA binding domain containing 4	3.97E-03	-2.54E-01
HS2ST1	heparan sulfate 2-O-sulfotransferase 1	3.98E-03	7.34E-01
RHBDD2	rhomboid domain containing 2	3.98E-03	-3.76E-01

IFT20	intraflagellar transport 20	3.98E-03	-6.01E-01
EED	embryonic ectoderm development	3.98E-03	5.73E-01
C20orf24	chromosome 20 open reading frame 24	3.98E-03	3.14E-01
C11orf95	chromosome 11 open reading frame 95	3.98E-03	-6.41E-01
PPP2R5A	protein phosphatase 2 regulatory subunit B'alpha	3.99E-03	4.71E-01
ID2	inhibitor of DNA binding 2, HLH protein	3.99E-03	9.05E-01
SLBP	stem-loop binding protein	4.00E-03	3.61E-01
LOC100506691	uncharacterized LOC100506691	4.00E-03	-3.00E-01
SPATA2L	spermatogenesis associated 2 like	4.01E-03	5.37E-01
ZNF519	zinc finger protein 519	4.02E-03	2.72E-01
ZBTB6	zinc finger and BTB domain containing 6	4.02E-03	-2.98E-01
PPA2	pyrophosphatase (inorganic) 2	4.02E-03	2.99E-01
ZNF430	zinc finger protein 430	4.03E-03	3.80E-01
EXOSC3	exosome component 3	4.03E-03	6.24E-01
CPPED1	calcineurin like phosphoesterase domain containing 1	4.03E-03	-3.06E-01
ANP32B	acidic nuclear phosphoprotein 32 family member B	4.03E-03	3.39E-01

GALNS	galactosamine (N-acetyl)-6-sulfatase	4.03E-03	-5.02E-01
HBP1	HMG-box transcription factor 1	4.03E-03	-2.93E-01
ZNF573	zinc finger protein 573	4.04E-03	-3.80E-01
NRG4	neuregulin 4	4.04E-03	-6.33E-01
CCT6A	chaperonin containing TCP1 subunit 6A	4.05E-03	6.32E-01
ESD	esterase D	4.05E-03	-4.36E-01
RANGAP1	Ran GTPase activating protein 1	4.05E-03	1.22
NMB	neuromedin B	4.06E-03	7.81E-01
PHKG2	phosphorylase kinase catalytic subunit gamma 2	4.06E-03	-6.48E-01
SPAG4	sperm associated antigen 4	4.06E-03	-8.63E-01
MSANTD3	Myb/SANT DNA binding domain containing 3	4.06E-03	4.60E-01
C19orf48	chromosome 19 open reading frame 48	4.06E-03	6.87E-01
TPD52L2	tumor protein D52 like 2	4.06E-03	-6.46E-01
SCARB1	scavenger receptor class B member 1	4.07E-03	-5.74E-01
ARL5B	ADP ribosylation factor like GTPase 5B	4.08E-03	9.56E-01

DEFB104A	defensin beta 104A	4.08E-03	2.29E-01
CPSF4	cleavage and polyadenylation specific factor 4	4.09E-03	-3.99E-01
CBWD1	COBW domain containing 1	4.09E-03	3.23E-01
PYROXD1	pyridine nucleotide-disulphide oxidoreductase domain 1	4.10E-03	7.09E-01
CTDSP2	CTD small phosphatase 2	4.10E-03	-9.07E-01
EIF4E	eukaryotic translation initiation factor 4E	4.11E-03	4.46E-01
PRKAR2B	protein kinase cAMP-dependent type II regulatory subunit beta	4.12E-03	2.84E-01
ACACB	acetyl-CoA carboxylase beta	4.12E-03	-4.98E-01
ZNF771	zinc finger protein 771	4.12E-03	-2.91E-01
PCSK1N	proprotein convertase subtilisin/kexin type 1 inhibitor	4.13E-03	-8.53E-01
PRDX5	peroxiredoxin 5	4.13E-03	-8.30E-01
ARL14EP	ADP ribosylation factor like GTPase 14 effector protein	4.13E-03	-6.34E-01
GSTO1	glutathione S-transferase omega 1	4.14E-03	6.19E-01
PPP2R3C	protein phosphatase 2 regulatory subunit B"gamma	4.14E-03	4.01E-01
CAMK2D	calcium/calmodulin dependent protein kinase II delta	4.14E-03	-2.48E-01

CD40	CD40 molecule	4.14E-03	-2.71E-01
DDHD1	DDHD domain containing 1	4.14E-03	6.69E-01
TRAPPC3	trafficking protein particle complex 3	4.14E-03	-5.66E-01
TXNL1	thioredoxin like 1	4.15E-03	5.87E-01
IGFBP6	insulin like growth factor binding protein 6	4.15E-03	7.84E-01
HAGH	hydroxyacylglutathione hydrolase	4.15E-03	-8.11E-01
ITSN1	intersectin 1	4.15E-03	-4.11E-01
IVNS1ABP	influenza virus NS1A binding protein	4.16E-03	5.32E-01
C4orf47	chromosome 4 open reading frame 47	4.17E-03	4.73E-01
CPT1B	carnitine palmitoyltransferase 1B	4.18E-03	-4.62E-01
EIF5A	eukaryotic translation initiation factor 5A	4.18E-03	4.07E-01
CRLS1	cardiolipin synthase 1	4.18E-03	3.26E-01
TADA3	transcriptional adaptor 3	4.19E-03	-2.89E-01
MKRN2	makorin ring finger protein 2	4.19E-03	5.39E-01
GAS2L3	growth arrest specific 2 like 3	4.19E-03	4.95E-01

XPR1	xenotropic and polytropic retrovirus receptor 1	4.19E-03	-2.88E-01
JMY	junction mediating and regulatory protein, p53 cofactor	4.19E-03	6.76E-01
FAM167A	family with sequence similarity 167 member A	4.20E-03	-3.60E-01
SLC25A32	solute carrier family 25 member 32	4.20E-03	7.04E-01
TPT1-AS1	TPT1 antisense RNA 1	4.21E-03	-5.13E-01
KBTBD2	kelch repeat and BTB domain containing 2	4.21E-03	4.85E-01
ARHGAP17	Rho GTPase activating protein 17	4.21E-03	7.11E-01
VPS11	VPS11, CORVET/HOPS core subunit	4.22E-03	-2.99E-01
CCDC88C	coiled-coil domain containing 88C	4.22E-03	2.97E-01
PEX13	peroxisomal biogenesis factor 13	4.22E-03	8.44E-01
APLP2	amyloid beta precursor like protein 2	4.23E-03	7.18E-01
ADAP1	ArfGAP with dual PH domains 1	4.23E-03	-6.97E-01
SP140L	SP140 nuclear body protein like	4.23E-03	3.32E-01
KDM1B	lysine demethylase 1B	4.23E-03	5.77E-01
H1F0	H1 histone family member 0	4.23E-03	-3.71E-01
ALDOC	aldolase, fructose-bisphosphate C	4.24E-03	-1.22

NRBP2	nuclear receptor binding protein 2	4.24E-03	-5.69E-01
GTF2F1	general transcription factor IIF subunit 1	4.24E-03	-3.21E-01
HTRA2	HtrA serine peptidase 2	4.25E-03	3.14E-01
USP1	ubiquitin specific peptidase 1	4.25E-03	7.36E-01
STX2	syntaxin 2	4.25E-03	3.82E-01
ZNF217	zinc finger protein 217	4.25E-03	-4.65E-01
EIF4B	eukaryotic translation initiation factor 4B	4.26E-03	-1
EIF4G2	eukaryotic translation initiation factor 4 gamma 2	4.26E-03	1.37
SASH1	SAM and SH3 domain containing 1	4.26E-03	-3.37E-01
ERC1	ELKS/RAB6-interacting/CAST family member 1	4.26E-03	4.14E-01
NUDT16L1	nudix hydrolase 16 like 1	4.26E-03	-5.47E-01
PDIA6	protein disulfide isomerase family A member 6	4.27E-03	-5.90E-01
BUB3	BUB3, mitotic checkpoint protein	4.28E-03	1.03
DNAJB6	DnaJ heat shock protein family (Hsp40) member B6	4.28E-03	5.56E-01
CUEDC1	CUE domain containing 1	4.28E-03	-1.16
HIGD1A	HIG1 hypoxia inducible domain family member 1A	4.28E-03	-4.68E-01

PSMD5-AS1	PSMD5 antisense RNA 1 (head to head)	4.29E-03	-5.58E-01
CCL5	C-C motif chemokine ligand 5	4.29E-03	3.97E-01
FAM174A	family with sequence similarity 174 member A	4.29E-03	4.15E-01
MAPK1	mitogen-activated protein kinase 1	4.29E-03	4.51E-01
OSBPL2	oxysterol binding protein like 2	4.29E-03	-3.96E-01
PTPRD	protein tyrosine phosphatase, receptor type D	4.30E-03	-8.69E-01
DNAJA2	DnaJ heat shock protein family (Hsp40) member A2	4.31E-03	4.37E-01
PLGLB1	plasminogen-like B1	4.31E-03	-3.55E-01
RIDA	reactive intermediate imine deaminase A homolog	4.32E-03	5.26E-01
FABP5	fatty acid binding protein 5	4.32E-03	1.04
RAB24	RAB24, member RAS oncogene family	4.32E-03	-4.93E-01
B3GALNT2	beta-1,3-N-acetylgalactosaminyltransferase 2	4.32E-03	2.68E-01
ACP6	acid phosphatase 6, lysophosphatidic	4.32E-03	2.86E-01
MYL7	myosin light chain 7	4.33E-03	3.47E-01
NLE1	notchless homolog 1	4.34E-03	4.69E-01
ESAM	endothelial cell adhesion molecule	4.34E-03	2.90E-01

NHSL2	NHS like 2	4.34E-03	-2.43E-01
GSTT2B	glutathione S-transferase theta 2B (gene/pseudogene)	4.34E-03	-4.14E-01
CLDND2	claudin domain containing 2	4.34E-03	-8.33E-01
RAP1A	RAP1A, member of RAS oncogene family	4.35E-03	4.07E-01
LDB3	LIM domain binding 3	4.35E-03	2.48E-01
S100A16	S100 calcium binding protein A16	4.35E-03	-3.86E-01
COPRS	coordinator of PRMT5 and differentiation stimulator	4.36E-03	1.02
RAP1GDS1	Rap1 GTPase-GDP dissociation stimulator 1	4.36E-03	3.40E-01
NAAA	N-acylethanolamine acid amidase	4.36E-03	2.75E-01
CYP39A1	cytochrome P450 family 39 subfamily A member 1	4.36E-03	-2.99E-01
MFNG	MFNG O-fucosylpeptide 3-beta-N-acetylglucosaminyltransferase	4.36E-03	3.12E-01
MED31	mediator complex subunit 31	4.36E-03	5.62E-01
CLN3	CLN3, battenin	4.37E-03	-3.30E-01
PLA2G2D	phospholipase A2 group IID	4.38E-03	-7.09E-01
RSRP1	arginine and serine rich protein 1	4.38E-03	-7.54E-01

CAPZA1	capping actin protein of muscle Z-line alpha subunit 1	4.39E-03	4.82E-01
PDLIM1	PDZ and LIM domain 1	4.39E-03	-2.47E-01
TP53RK	TP53 regulating kinase	4.40E-03	-3.62E-01
AMY2B	amylase, alpha 2B (pancreatic)	4.40E-03	-4.37E-01
TIMM22	translocase of inner mitochondrial membrane 22 homolog (yeast)	4.40E-03	-5.53E-01
COMMD4	COMM domain containing 4	4.40E-03	7.87E-01
AKIRIN1	akirin 1	4.40E-03	-2.48E-01
IGSF3	immunoglobulin superfamily member 3	4.40E-03	-5.40E-01
KCNJ4	potassium voltage-gated channel subfamily J member 4	4.41E-03	-5.35E-01
TMEM259	transmembrane protein 259	4.42E-03	-4.80E-01
REV1	REV1, DNA directed polymerase	4.42E-03	-4.28E-01
EBAG9	estrogen receptor binding site associated, antigen, 9	4.43E-03	-2.80E-01
ELP3	elongator acetyltransferase complex subunit 3	4.43E-03	-3.61E-01

YME1L1	YME1 like 1 ATPase	4.43E-03	7.74E-01
DUSP3	dual specificity phosphatase 3	4.43E-03	-6.08E-01
LOC105378828	uncharacterized LOC105378828	4.44E-03	3.01E-01
MYNN	myoneurin	4.44E-03	4.46E-01
CPT1A	carnitine palmitoyltransferase 1A	4.44E-03	-2.73E-01
C17orf89	chromosome 17 open reading frame 89	4.44E-03	-9.35E-01
PHF14	PHD finger protein 14	4.45E-03	4.16E-01
ZNF555	zinc finger protein 555	4.45E-03	3.64E-01
KCNH3	potassium voltage-gated channel subfamily H member 3	4.45E-03	-2.39E-01
NISCH	nischarin	4.46E-03	-9.21E-01
ASXL2	additional sex combs like 2, transcriptional regulator	4.46E-03	-6.45E-01
NFYA	nuclear transcription factor Y subunit alpha	4.46E-03	4.52E-01
N4BP2L1	NEDD4 binding protein 2 like 1	4.46E-03	2.54E-01
OR52I1	olfactory receptor family 52 subfamily I member 1	4.46E-03	2.33E-01
ITPKB	inositol-trisphosphate 3-kinase B	4.46E-03	-4.92E-01

PGAM4	phosphoglycerate mutase family member 4	4.47E-03	5.34E-01
ME2	malic enzyme 2	4.47E-03	6.77E-01
RFPL1S	RFPL1 antisense RNA 1	4.47E-03	-3.53E-01
WDR46	WD repeat domain 46	4.47E-03	2.75E-01
ABCC10	ATP binding cassette subfamily C member 10	4.47E-03	-2.72E-01
ATF4	activating transcription factor 4	4.48E-03	2.79E-01
SIVA1	SIVA1 apoptosis inducing factor	4.48E-03	8.18E-01
ASB13	ankyrin repeat and SOCS box containing 13	4.48E-03	4.00E-01
RPL29	ribosomal protein L29	4.49E-03	-9.48E-01
HIBCH	3-hydroxyisobutyryl-CoA hydrolase	4.49E-03	-6.02E-01
MAGIX	MAGI family member, X-linked	4.49E-03	-3.65E-01
TUSC3	tumor suppressor candidate 3	4.49E-03	-6.81E-01
TMEM167A	transmembrane protein 167A	4.49E-03	6.75E-01
N4BP2L1	NEDD4 binding protein 2 like 1	4.50E-03	3.09E-01
KRT16	keratin 16	4.50E-03	-2.45E-01

LUZP1	leucine zipper protein 1	4.51E-03	-4.96E-01
GALM	galactose mutarotase	4.51E-03	-3.12E-01
SNORD3D	small nucleolar RNA, C/D box 3D	4.51E-03	-8.45E-01
FGFR4	fibroblast growth factor receptor 4	4.51E-03	-4.91E-01
TMEM97	transmembrane protein 97	4.51E-03	5.72E-01
PPP6R2	protein phosphatase 6 regulatory subunit 2	4.51E-03	-9.30E-01
SRPK1	SRSF protein kinase 1	4.51E-03	7.77E-01
OSBPL11	oxysterol binding protein like 11	4.51E-03	4.03E-01
OSBPL1A	oxysterol binding protein like 1A	4.52E-03	-5.65E-01
SNAR-A1	small ILF3/NF90-associated RNA A1	4.52E-03	2.61
TTC27	tetratricopeptide repeat domain 27	4.52E-03	3.51E-01
MSL2	male-specific lethal 2 homolog (Drosophila)	4.53E-03	4.36E-01
NAF1	nuclear assembly factor 1 ribonucleoprotein	4.53E-03	4.06E-01
C8orf44	chromosome 8 open reading frame 44	4.53E-03	-2.53E-01
PCNX2	pecanex homolog 2 (Drosophila)	4.53E-03	-2.76E-01

ALDH6A1	aldehyde dehydrogenase 6 family member A1	4.54E-03	-3.84E-01
SNORD27	small nucleolar RNA, C/D box 27	4.54E-03	4.53E-01
CHPT1	choline phosphotransferase 1	4.54E-03	-3.85E-01
PPP1R15B	protein phosphatase 1 regulatory subunit 15B	4.55E-03	8.20E-01
PAK1IP1	PAK1 interacting protein 1	4.55E-03	5.69E-01
PTP4A3	protein tyrosine phosphatase type IVA, member 3	4.56E-03	-5.51E-01
SKIV2L	Ski2 like RNA helicase	4.56E-03	-5.48E-01
FAM219B	family with sequence similarity 219 member B	4.57E-03	-3.82E-01
ATG5	autophagy related 5	4.57E-03	-3.67E-01
HACD3	3-hydroxyacyl-CoA dehydratase 3	4.58E-03	-2.93E-01
HYKK	hydroxylysine kinase	4.58E-03	-5.49E-01
FLJ35934	FLJ35934	4.58E-03	-7.37E-01
HMCES	5-hydroxymethylcytosine (hmC) binding, ES cell-specific	4.59E-03	6.01E-01
AGGF1	angiogenic factor with G-patch and FHA domains 1	4.59E-03	-3.65E-01

RPL7	ribosomal protein L7	4.59E-03	-4.24E-01
YIPF1	Yip1 domain family member 1	4.60E-03	-3.87E-01
TPM3P9	tropomyosin 3 pseudogene 9	4.60E-03	9.57E-01
PWP2	PWP2 periodic tryptophan protein homolog (yeast)	4.60E-03	4.07E-01
SLAMF7	SLAM family member 7	4.61E-03	-3.00E-01
GEMIN2	gem nuclear organelle associated protein 2	4.62E-03	7.97E-01
IPO13	importin 13	4.62E-03	-4.66E-01
LACE1	lactation elevated 1	4.63E-03	2.72E-01
MGAT1	mannosyl (alpha-1,3-)-glycoprotein beta-1,2-N-acetylglucosaminyltransferase	4.63E-03	-4.92E-01
NAT14	N-acetyltransferase 14 (putative)	4.63E-03	-4.99E-01
TNPO1	transportin 1	4.64E-03	6.27E-01
CALCOCO1	calcium binding and coiled-coil domain 1	4.64E-03	-5.23E-01
IFT52	intraflagellar transport 52	4.64E-03	4.01E-01
DIAPH1	diaphanous related formin 1	4.64E-03	1.01
RBCK1	RANBP2-type and C3HC4-type zinc finger containing 1	4.65E-03	-4.84E-01

CPEB4	cytoplasmic polyadenylation element binding protein 4	4.65E-03	5.04E-01
DCLRE1B	DNA cross-link repair 1B	4.66E-03	3.15E-01
NET1	neuroepithelial cell transforming 1	4.66E-03	4.48E-01
TP53TG1	TP53 target 1 (non-protein coding)	4.66E-03	-7.95E-01
NPM1	nucleophosmin	4.66E-03	2.51E-01
NUP205	nucleoporin 205	4.67E-03	9.24E-01
C14orf166	chromosome 14 open reading frame 166	4.67E-03	3.34E-01
KLF10	Kruppel like factor 10	4.68E-03	3.66E-01
NAPA	NSF attachment protein alpha	4.68E-03	-3.37E-01
FBXL6	F-box and leucine rich repeat protein 6	4.69E-03	4.40E-01
MPZL3	myelin protein zero like 3	4.69E-03	2.23E-01
ALG3	ALG3, alpha-1,3- mannosyltransferase	4.69E-03	-5.48E-01
SORD	sorbitol dehydrogenase	4.70E-03	6.94E-01
SYNGR1	synaptogyrin 1	4.70E-03	-3.90E-01
CALCOCO2	calcium binding and coiled-coil domain 2	4.70E-03	-2.17E-01

PDCD6	programmed cell death 6	4.70E-03	-2.38E-01
DSEL	dermatan sulfate epimerase-like	4.71E-03	2.29E-01
RPL37A	ribosomal protein L37a	4.71E-03	-5.33E-01
DUS1L	dihydrouridine synthase 1 like	4.72E-03	-4.30E-01
ALDH5A1	aldehyde dehydrogenase 5 family member A1	4.73E-03	4.28E-01
WDR91	WD repeat domain 91	4.74E-03	3.48E-01
RNF103	ring finger protein 103	4.76E-03	3.60E-01
NEXN	nexilin F-actin binding protein	4.77E-03	-3.39E-01
NUCB1	nucleobindin 1	4.77E-03	-5.73E-01
RPL31	ribosomal protein L31	4.77E-03	-2.84E-01
RNF5P1	ring finger protein 5 pseudogene 1	4.77E-03	-8.35E-01
SCRN2	secernin 2	4.78E-03	-2.94E-01
MOSPD3	motile sperm domain containing 3	4.78E-03	-6.20E-01
MRPS18C	mitochondrial ribosomal protein S18C	4.78E-03	6.62E-01

TPRKB	TP53RK binding protein	4.78E-03	7.44E-01
LSM14A	LSM14A, mRNA processing body assembly factor	4.79E-03	-3.60E-01
OLFM3	olfactomedin 3	4.81E-03	-9.23E-01
CHD6	chromodomain helicase DNA binding protein 6	4.81E-03	-4.25E-01
TCF20	transcription factor 20	4.81E-03	-6.15E-01
SHF	Src homology 2 domain containing F	4.81E-03	-2.80E-01
C14orf93	chromosome 14 open reading frame 93	4.82E-03	-5.80E-01
DOCK6	dedicator of cytokinesis 6	4.83E-03	-5.91E-01
TET1	tet methylcytosine dioxygenase 1	4.83E-03	-8.60E-01
NDUFAF2	NADH:ubiquinone oxidoreductase complex assembly factor 2	4.83E-03	5.66E-01
MEIS3P1	Meis homeobox 3 pseudogene 1	4.83E-03	5.35E-01
FAM53A	family with sequence similarity 53 member A	4.84E-03	-5.56E-01
NME1	NME/NM23 nucleoside diphosphate kinase 1	4.85E-03	-7.09E-01
ZNF616	zinc finger protein 616	4.85E-03	3.76E-01

FBXO9	F-box protein 9	4.86E-03	-4.06E-01
APC2	APC2, WNT signaling pathway regulator	4.86E-03	-3.71E-01
ACER3	alkaline ceramidase 3	4.86E-03	-2.71E-01
MANEA	mannosidase endo-alpha	4.86E-03	2.58E-01
USP13	ubiquitin specific peptidase 13 (isopeptidase T-3)	4.87E-03	-3.61E-01
MTRF1	mitochondrial translational release factor 1	4.88E-03	2.21E-01
FAM174A	family with sequence similarity 174 member A	4.88E-03	5.80E-01
ARL5A	ADP ribosylation factor like GTPase 5A	4.88E-03	2.20E-01
CFAP36	cilia and flagella associated protein 36	4.88E-03	5.38E-01
FUS	FUS RNA binding protein	4.89E-03	6.85E-01
P2RX6P	purinergic receptor P2X 6 pseudogene	4.90E-03	2.30E-01
UBA2	ubiquitin like modifier activating enzyme 2	4.91E-03	5.27E-01
BIRC3	baculoviral IAP repeat containing 3	4.91E-03	-6.91E-01
ETF1	eukaryotic translation termination factor 1	4.91E-03	4.59E-01
TRIOBP	TRIO and F-actin binding protein	4.92E-03	-8.39E-01

MKI67	marker of proliferation Ki-67	4.93E-03	3.09E -01
CDCA4	cell division cycle associated 4	4.93E-03	5.21E -01
P3H4	prolyl 3-hydroxylase family member 4 (non-enzymatic)	4.93E-03	- 6.19E -01
TMPO	thymopoietin	4.94E-03	4.90E -01
MAP3K5	mitogen-activated protein kinase kinase kinase 5	4.96E-03	3.57E -01
TRIM65	tripartite motif containing 65	4.96E-03	- 3.17E -01
EIF2B5	eukaryotic translation initiation factor 2B subunit epsilon	4.97E-03	- 7.02E -01
RFX7	regulatory factor X7	4.97E-03	- 4.06E -01
GGT8P	gamma-glutamyltransferase 8 pseudogene	4.97E-03	- 2.77E -01
PDE9A	phosphodiesterase 9A	4.97E-03	- 2.48E -01
SCO1	SCO1 cytochrome c oxidase assembly protein	4.98E-03	4.15E -01
ZNF738	zinc finger protein 738	4.98E-03	- 4.21E -01
SMAD9	SMAD family member 9	4.98E-03	9.52E -01
COL27A1	collagen type XXVII alpha 1 chain	5.00E-03	- 3.63E -01

P4HA1	prolyl 4-hydroxylase subunit alpha 1	5.01E-03	-8.12E-01
PLCB1	phospholipase C beta 1	5.01E-03	-3.10E-01
SLC13A3	solute carrier family 13 member 3	5.01E-03	2.29E-01
UHRF1BP1	UHRF1 binding protein 1	5.01E-03	5.01E-01
SLC29A4	solute carrier family 29 member 4	5.02E-03	-3.53E-01
VPS4A	vacuolar protein sorting 4 homolog A	5.02E-03	4.19E-01
RPL30	ribosomal protein L30	5.03E-03	-4.32E-01
CEACAM1	carcinoembryonic antigen related cell adhesion molecule 1	5.03E-03	-2.47E-01
CCDC82	coiled-coil domain containing 82	5.04E-03	4.30E-01
NAA25	N(alpha)-acetyltransferase 25, NatB auxiliary subunit	5.04E-03	2.75E-01
CENPM	centromere protein M	5.05E-03	5.33E-01
STAT1	signal transducer and activator of transcription 1	5.05E-03	4.11E-01
SLC35C1	solute carrier family 35 member C1	5.05E-03	-3.76E-01
ARID1A	AT-rich interaction domain 1A	5.06E-03	-4.83E-01
MPP2	membrane palmitoylated protein 2	5.06E-03	-4.44E-01

PDIA3P1	protein disulfide isomerase family A member 3 pseudogene 1	5.07E-03	-3.51E-01
MTUS1	microtubule associated tumor suppressor 1	5.09E-03	-3.56E-01
BLOC1S4	biogenesis of lysosomal organelles complex 1 subunit 4	5.09E-03	-8.10E-01
UBE3C	ubiquitin protein ligase E3C	5.09E-03	6.92E-01
IRAK4	interleukin 1 receptor associated kinase 4	5.10E-03	2.98E-01
REEP3	receptor accessory protein 3	5.10E-03	4.29E-01
SLC9A6	solute carrier family 9 member A6	5.10E-03	8.36E-01
TOP1MT	topoisomerase (DNA) I, mitochondrial	5.10E-03	-5.87E-01
EIF1	eukaryotic translation initiation factor 1	5.11E-03	2.29E-01
C1GALT1C1	C1GALT1 specific chaperone 1	5.11E-03	-5.53E-01
LRFN5	leucine rich repeat and fibronectin type III domain containing 5	5.11E-03	-3.25E-01
RPL26	ribosomal protein L26	5.11E-03	-5.00E-01
DDT	D-dopachrome tautomerase	5.11E-03	-5.70E-01
IFT46	intraflagellar transport 46	5.11E-03	3.99E-01
FKBP1B	FK506 binding protein 1B	5.12E-03	3.52E-01

LATS1	large tumor suppressor kinase 1	5.12E-03	2.59E-01
BCL2	BCL2, apoptosis regulator	5.12E-03	2.55E-01
UGT3A2	UDP glycosyltransferase family 3 member A2	5.12E-03	4.99E-01
ZNF214	zinc finger protein 214	5.12E-03	2.44E-01
FEM1C	fem-1 homolog C	5.12E-03	5.81E-01
FAM127B	family with sequence similarity 127 member B	5.12E-03	-6.07E-01
GFRA1	GDNF family receptor alpha 1	5.13E-03	-2.43E-01
TCF19	transcription factor 19	5.13E-03	3.66E-01
RHOC	ras homolog family member C	5.13E-03	-8.01E-01
IRAIN	IGF1R antisense imprinted non-protein coding RNA	5.13E-03	-2.86E-01
Sep-03	septin 3	5.14E-03	3.93E-01
TBC1D8B	TBC1 domain family member 8B	5.14E-03	4.11E-01
PRMT2	protein arginine methyltransferase 2	5.14E-03	-7.16E-01
TRMT10C	tRNA methyltransferase 10C, mitochondrial RNase P subunit	5.14E-03	8.69E-01
UFM1	ubiquitin fold modifier 1	5.14E-03	3.47E-01

ZFAND3	zinc finger AN1-type containing 3	5.15E-03	-3.58E-01
KIF2A	kinesin family member 2A	5.17E-03	6.34E-01
ALKBH7	alkB homolog 7	5.17E-03	-9.99E-01
FOXA3	forkhead box A3	5.18E-03	5.13E-01
MAST3	microtubule associated serine/threonine kinase 3	5.18E-03	-9.60E-01
ZNF211	zinc finger protein 211	5.18E-03	3.61E-01
PTP4A3	protein tyrosine phosphatase type IVA, member 3	5.19E-03	-3.66E-01
C8orf58	chromosome 8 open reading frame 58	5.19E-03	-3.28E-01
ELK4	ELK4, ETS transcription factor	5.19E-03	2.68E-01
MAGEA4	MAGE family member A4	5.20E-03	2.76E-01
C12orf43	chromosome 12 open reading frame 43	5.21E-03	4.08E-01
UBXN6	UBX domain protein 6	5.21E-03	-6.81E-01
TOP1P1	topoisomerase (DNA) I pseudogene 1	5.21E-03	6.46E-01
CD68	CD68 molecule	5.22E-03	-6.31E-01
DBR1	debranching RNA lariats 1	5.22E-03	3.38E-01

TMEM5	transmembrane protein 5	5.23E-03	4.93E-01
HNRNPK	heterogeneous nuclear ribonucleoprotein K	5.24E-03	-4.87E-01
RSG1	REM2 and RAB like small GTPase 1	5.25E-03	2.78E-01
SNRNP25	small nuclear ribonucleoprotein U11/U12 subunit 25	5.25E-03	4.63E-01
EIF3J-AS1	EIF3J antisense RNA 1 (head to head)	5.25E-03	-4.59E-01
PIR	pirin	5.27E-03	6.63E-01
JMJD6	arginine demethylase and lysine hydroxylase	5.27E-03	-4.32E-01
JAG2	jagged 2	5.28E-03	-4.61E-01
CWC27	CWC27 spliceosome associated protein homolog	5.28E-03	3.84E-01
YBEY	ybeY metallopeptidase (putative)	5.28E-03	-9.01E-01
CLP1	cleavage and polyadenylation factor I subunit 1	5.28E-03	-2.43E-01
RUSC2	RUN and SH3 domain containing 2	5.28E-03	6.09E-01
TMEM135	transmembrane protein 135	5.28E-03	-6.81E-01
CTH	cystathionine gamma-lyase	5.28E-03	-2.59E-01
DNM1L	dynamin 1 like	5.29E-03	7.93E-01

DCDC2	doublecortin domain containing 2	5.29E-03	3.68E-01
RPUSD3	RNA pseudouridylate synthase domain containing 3	5.29E-03	4.63E-01
SPRYD7	SPRY domain containing 7	5.30E-03	-5.48E-01
SLC9A6	solute carrier family 9 member A6	5.30E-03	7.02E-01
AKAP12	A-kinase anchoring protein 12	5.30E-03	3.24E-01
KCTD6	potassium channel tetramerization domain containing 6	5.30E-03	2.98E-01
PLCE1	phospholipase C epsilon 1	5.31E-03	2.47E-01
N4BP2	NEDD4 binding protein 2	5.32E-03	-7.43E-01
FBXL8	F-box and leucine rich repeat protein 8	5.33E-03	-2.23E-01
YARS2	tyrosyl-tRNA synthetase 2	5.33E-03	3.14E-01
SLC25A43	solute carrier family 25 member 43	5.33E-03	-4.14E-01
IFI27	interferon alpha inducible protein 27	5.34E-03	2.72E-01
NLRP11	NLR family pyrin domain containing 11	5.35E-03	2.30E-01
SEZ6L2	seizure related 6 homolog like 2	5.35E-03	-7.94E-01
CSTF3	cleavage stimulation factor subunit 3	5.35E-03	-6.97E-01

MATR3	matrin 3	5.35E-03	-4.27E-01
RRAS	related RAS viral (r-ras) oncogene homolog	5.37E-03	-5.79E-01
ARMC1	armadillo repeat containing 1	5.37E-03	6.36E-01
DPY30	dpy-30, histone methyltransferase complex regulatory subunit	5.38E-03	3.48E-01
MIR99AHG	mir-99a-let-7c cluster host gene	5.38E-03	-9.09E-01
RNF187	ring finger protein 187	5.39E-03	-4.77E-01
PCBP4	poly(rC) binding protein 4	5.39E-03	-4.08E-01
LYL1	LYL1, basic helix-loop-helix family member	5.39E-03	3.25E-01
COBLL1	cordon-bleu WH2 repeat protein like 1	5.39E-03	2.88E-01
IKZF4	IKAROS family zinc finger 4	5.40E-03	2.28E-01
PNPO	pyridoxamine 5'-phosphate oxidase	5.40E-03	-4.20E-01
RAB4B	RAB4B, member RAS oncogene family	5.40E-03	-3.55E-01
PSD4	pleckstrin and Sec7 domain containing 4	5.40E-03	2.02E-01
FXR1	FMR1 autosomal homolog 1	5.41E-03	-6.61E-01
SYCE2	synaptonemal complex central element protein 2	5.41E-03	2.42E-01

COQ3	coenzyme Q3, methyltransferase	5.41E-03	5.98E-01
POC1B	POC1 centriolar protein B	5.41E-03	5.85E-01
SLC35C2	solute carrier family 35 member C2	5.41E-03	-3.33E-01
GIGYF2	GRB10 interacting GYF protein 2	5.42E-03	-6.93E-01
SAMD9	sterile alpha motif domain containing 9	5.42E-03	3.43E-01
MAPKAPK5	mitogen-activated protein kinase-activated protein kinase 5	5.42E-03	2.54E-01
TSPAN6	tetraspanin 6	5.43E-03	-4.11E-01
TTC30B	tetratricopeptide repeat domain 30B	5.43E-03	2.76E-01
NCOA4	nuclear receptor coactivator 4	5.43E-03	-4.86E-01
HDAC10	histone deacetylase 10	5.43E-03	-2.76E-01
MXI1	MAX interactor 1, dimerization protein	5.44E-03	-7.03E-01
CECR5-AS1	CECR5 antisense RNA 1	5.44E-03	-6.92E-01
RPE	ribulose-5-phosphate-3-epimerase	5.44E-03	5.36E-01
SDHC	succinate dehydrogenase complex subunit C	5.44E-03	-7.10E-01

HEMK1	HemK methyltransferase family member 1	5.45E-03	-2.68E-01
BPGM	bisphosphoglycerate mutase	5.45E-03	4.49E-01
MTHFR	methylenetetrahydrofolate reductase (NAD(P)H)	5.46E-03	-5.26E-01
FLVCR1-AS1	FLVCR1 antisense RNA 1 (head to head)	5.46E-03	-1.01
CRYZL1	crystallin zeta like 1	5.46E-03	-7.55E-01
CRYZL1	crystallin zeta like 1	5.47E-03	-4.17E-01
USP15	ubiquitin specific peptidase 15	5.47E-03	-6.95E-01
ZBTB34	zinc finger and BTB domain containing 34	5.47E-03	-5.29E-01
PCBP2	poly(rC) binding protein 2	5.47E-03	-4.44E-01
ZFP69B	ZFP69 zinc finger protein B	5.47E-03	3.98E-01
TRIM60	tripartite motif containing 60	5.48E-03	2.33E-01
LARS2	leucyl-tRNA synthetase 2, mitochondrial	5.48E-03	4.31E-01
SPEG	SPEG complex locus	5.49E-03	-2.31E-01
CHD3	chromodomain helicase DNA binding protein 3	5.50E-03	-3.62E-01
BBS5	Bardet-Biedl syndrome 5	5.50E-03	2.48E-01

GATAD2A	GATA zinc finger domain containing 2A	5.51E-03	5.36E-01
STK24	serine/threonine kinase 24	5.51E-03	-4.49E-01
BRD7	bromodomain containing 7	5.52E-03	4.93E-01
KDM7A	lysine demethylase 7A	5.52E-03	-2.28E-01
HIST1H2BD	histone cluster 1, H2bd	5.52E-03	-7.69E-01
POT1	protection of telomeres 1	5.52E-03	5.02E-01
TPST2	tyrosylprotein sulfotransferase 2	5.53E-03	-5.16E-01
CAMK2B	calcium/calmodulin dependent protein kinase II beta	5.54E-03	-3.04E-01
HIST1H4E	histone cluster 1, H4e	5.54E-03	2.13E-01
RNF5	ring finger protein 5	5.54E-03	-7.72E-01
RNF216P1	ring finger protein 216 pseudogene 1	5.54E-03	5.10E-01
HES2	hes family bHLH transcription factor 2	5.54E-03	-3.79E-01
ZNF569	zinc finger protein 569	5.54E-03	4.18E-01
ZBTB11-AS1	ZBTB11 antisense RNA 1	5.55E-03	2.96E-01
MBP	myelin basic protein	5.55E-03	2.53E-01

DCTPP1	dCTP pyrophosphatase 1	5.55E-03	5.23E-01
RRN3	RRN3 homolog, RNA polymerase I transcription factor	5.55E-03	4.34E-01
INO80D	INO80 complex subunit D	5.56E-03	-3.46E-01
CHD1	chromodomain helicase DNA binding protein 1	5.56E-03	2.66E-01
ACOT1	acyl-CoA thioesterase 1	5.56E-03	-1.06
NAA20	N(alpha)-acetyltransferase 20, NatB catalytic subunit	5.56E-03	4.45E-01
KIAA0895L	KIAA0895 like	5.56E-03	-3.16E-01
FOSL1	FOS like 1, AP-1 transcription factor subunit	5.56E-03	3.41E-01
TRAPPC1	trafficking protein particle complex 1	5.56E-03	-3.90E-01
CT45A5	cancer/testis antigen family 45, member A5	5.57E-03	3.39E-01
U2AF1	U2 small nuclear RNA auxiliary factor 1	5.57E-03	6.60E-01
SHC1	SHC adaptor protein 1	5.57E-03	-7.19E-01
SPG7	SPG7, paraplegin matrix AAA peptidase subunit	5.57E-03	-2.07E-01
VPS33B	VPS33B, late endosome and lysosome associated	5.57E-03	-3.16E-01
PPP2R5C	protein phosphatase 2 regulatory subunit B'gamma	5.57E-03	4.16E-01
SQLE	squalene epoxidase	5.58E-03	7.45E-01

PRCC	papillary renal cell carcinoma (translocation-associated)	5.59E-03	-2.88E-01
GDI2	GDP dissociation inhibitor 2	5.59E-03	6.00E-01
GID8	GID complex subunit 8 homolog	5.59E-03	5.25E-01
FBXO3	F-box protein 3	5.59E-03	4.08E-01
RAB34	RAB34, member RAS oncogene family	5.60E-03	3.34E-01
NARS	asparaginyl-tRNA synthetase	5.60E-03	4.04E-01
SCAND1	SCAN domain containing 1	5.60E-03	-8.82E-01
PPP2R5E	protein phosphatase 2 regulatory subunit B'epsilon	5.62E-03	6.24E-01
LEP	leptin	5.62E-03	-8.27E-01
VPS13A	vacuolar protein sorting 13 homolog A	5.62E-03	2.77E-01
ATP8A1	ATPase phospholipid transporting 8A1	5.62E-03	2.72E-01
KDELC1	KDEL motif containing 1	5.65E-03	-5.06E-01
AK2	adenylate kinase 2	5.67E-03	4.09E-01
THAP12	THAP domain containing 12	5.69E-03	5.89E-01
WRB	tryptophan rich basic protein	5.69E-03	-2.50E-01
FAM86C2P	family with sequence similarity 86, member A pseudogene	5.70E-03	4.14E-01

ANAPC13	anaphase promoting complex subunit 13	5.70E-03	3.24E-01
FLJ26850	FLJ26850 protein	5.71E-03	-2.15E-01
DLG2	discs large MAGUK scaffold protein 2	5.72E-03	-2.33E-01
ODF2L	outer dense fiber of sperm tails 2 like	5.72E-03	2.82E-01
LRP4	LDL receptor related protein 4	5.73E-03	-7.01E-01
P2RX4	purinergic receptor P2X 4	5.73E-03	-5.04E-01
LCE1F	late cornified envelope 1F	5.74E-03	-2.34E-01
CCDC34	coiled-coil domain containing 34	5.74E-03	8.40E-01
ZNF644	zinc finger protein 644	5.74E-03	-3.74E-01
TBC1D4	TBC1 domain family member 4	5.75E-03	-8.29E-01
TMEM209	transmembrane protein 209	5.76E-03	8.82E-01
MRPS16	mitochondrial ribosomal protein S16	5.76E-03	-3.17E-01
GPAA1	glycosylphosphatidylinositol anchor attachment 1	5.77E-03	-5.06E-01
SASH1	SAM and SH3 domain containing 1	5.77E-03	-3.09E-01

DNASE2	deoxyribonuclease 2, lysosomal	5.77E-03	-6.35E-01
CEBPA	CCAAT/enhancer binding protein alpha	5.77E-03	-5.39E-01
DDX47	DEAD-box helicase 47	5.77E-03	2.49E-01
IFT172	intraflagellar transport 172	5.78E-03	-2.86E-01
DAD1	defender against cell death 1	5.78E-03	-6.28E-01
THYN1	thymocyte nuclear protein 1	5.78E-03	5.21E-01
USP51	ubiquitin specific peptidase 51	5.78E-03	-3.93E-01
UNC5B	unc-5 netrin receptor B	5.78E-03	-4.48E-01
MRPL42P5	mitochondrial ribosomal protein L42 pseudogene 5	5.79E-03	-3.53E-01
MYO5A	myosin VA	5.79E-03	5.02E-01
GULP1	GULP, engulfment adaptor PTB domain containing 1	5.81E-03	-4.05E-01
PARN	poly(A)-specific ribonuclease	5.81E-03	5.13E-01
BIRC2	baculoviral IAP repeat containing 2	5.82E-03	7.30E-01
PANX2	pannexin 2	5.82E-03	2.97E-01

GH1	growth hormone 1	5.82E-03	-2.51E-01
FXN	frataxin	5.83E-03	2.32E-01
PPP2R3A	protein phosphatase 2 regulatory subunit B"alpha	5.83E-03	-5.46E-01
HSPD1	heat shock protein family D (Hsp60) member 1	5.83E-03	3.28E-01
URGCP	upregulator of cell proliferation	5.84E-03	3.66E-01
ZNF473	zinc finger protein 473	5.85E-03	5.44E-01
GABPB1	GA binding protein transcription factor beta subunit 1	5.85E-03	4.66E-01
PPP4R2	protein phosphatase 4 regulatory subunit 2	5.85E-03	7.75E-01
FLJ26850	FLJ26850 protein	5.86E-03	-8.15E-01
SP4	Sp4 transcription factor	5.87E-03	6.03E-01
ZRANB2	zinc finger RANBP2-type containing 2	5.87E-03	2.12E-01
FEZ2	fasciculation and elongation protein zeta 2	5.87E-03	-6.73E-01
PLGRKT	plasminogen receptor with a C-terminal lysine	5.89E-03	5.23E-01
CSNK1E	casein kinase 1 epsilon	5.89E-03	-4.57E-01
MSRA	methionine sulfoxide reductase A	5.89E-03	-5.97E-01

USP11	ubiquitin specific peptidase 11	5.90E-03	6.95E-01
ARAP1	ArfGAP with RhoGAP domain, ankyrin repeat and PH domain 1	5.90E-03	-4.03E-01
MED10	mediator complex subunit 10	5.90E-03	5.37E-01
ZNF460	zinc finger protein 460	5.91E-03	2.58E-01
RNF38	ring finger protein 38	5.91E-03	5.41E-01
SDF4	stromal cell derived factor 4	5.92E-03	-7.57E-01
DYRK1B	dual specificity tyrosine phosphorylation regulated kinase 1B	5.93E-03	-2.87E-01
HAUS2	HAUS augmin like complex subunit 2	5.93E-03	-7.07E-01
CNPY3	canopy FGF signaling regulator 3	5.93E-03	-9.71E-01
RARS	arginyl-tRNA synthetase	5.94E-03	4.22E-01
ALAD	aminolevulinate dehydratase	5.94E-03	-4.98E-01
SARAF	store-operated calcium entry associated regulatory factor	5.94E-03	-2.78E-01
GOSR2	golgi SNAP receptor complex member 2	5.94E-03	3.20E-01
CCDC59	coiled-coil domain containing 59	5.95E-03	4.57E-01
PFKL	phosphofructokinase, liver type	5.95E-03	-5.11E-01

ZNF266	zinc finger protein 266	5.96E-03	4.57E-01
ZNF623	zinc finger protein 623	5.96E-03	-2.09E-01
ZNF273	zinc finger protein 273	5.96E-03	1.96E-01
RNF31	ring finger protein 31	5.96E-03	-2.13E-01
SUSD6	sushi domain containing 6	5.96E-03	-8.07E-01
PLAC1	placenta specific 1	5.96E-03	2.31E-01
LOC642852	uncharacterized LOC642852	5.97E-03	-6.27E-01
RBBP6	RB binding protein 6, ubiquitin ligase	5.97E-03	-3.07E-01
SARDH	sarcosine dehydrogenase	5.97E-03	-2.91E-01
NDUFB11	NADH:ubiquinone oxidoreductase subunit B11	5.98E-03	-4.90E-01
ADGRE1	adhesion G protein-coupled receptor E1	5.99E-03	-3.62E-01
R3HDM1	R3H domain containing 1	5.99E-03	7.96E-01
BCLAF1	BCL2 associated transcription factor 1	5.99E-03	9.52E-01
RASSF5	Ras association domain family member 5	6.00E-03	2.50E-01
WLS	wntless Wnt ligand secretion mediator	6.00E-03	3.20E-01

PHKA2	phosphorylase kinase regulatory subunit alpha 2	6.00E-03	-4.93E-01
WAC	WW domain containing adaptor with coiled-coil	6.01E-03	3.54E-01
ESYT2	extended synaptotagmin 2	6.01E-03	-7.62E-01
SPATA17	spermatogenesis associated 17	6.02E-03	3.11E-01
AK4	adenylate kinase 4	6.02E-03	-3.39E-01
LYSMD3	LysM domain containing 3	6.02E-03	6.55E-01
SVIL	supervillin	6.03E-03	-7.64E-01
FAM122B	family with sequence similarity 122B	6.03E-03	8.36E-01
LOC401127	WD repeat domain 5 pseudogene	6.03E-03	4.96E-01
ARHGAP45	Rho GTPase activating protein 45	6.04E-03	4.41E-01
HSPA13	heat shock protein family A (Hsp70) member 13	6.04E-03	4.99E-01
CCND2	cyclin D2	6.04E-03	-9.17E-01
PEX3	peroxisomal biogenesis factor 3	6.05E-03	5.50E-01
GPS1	G protein pathway suppressor 1	6.06E-03	-6.19E-01
PTPN13	protein tyrosine phosphatase, non-receptor type 13	6.07E-03	-8.36E-01

ASB3	ankyrin repeat and SOCS box containing 3	6.07E-03	-7.23E-01
RFWD3	ring finger and WD repeat domain 3	6.07E-03	8.46E-01
KDSR	3-ketodihydrosphingosine reductase	6.07E-03	-2.53E-01
ART1	ADP-ribosyltransferase 1	6.08E-03	-2.88E-01
SRGN	serglycin	6.09E-03	2.34E-01
FARSB	phenylalanyl-tRNA synthetase beta subunit	6.09E-03	2.19E-01
TPST2	tyrosylprotein sulfotransferase 2	6.10E-03	-2.75E-01
RPL26L1	ribosomal protein L26 like 1	6.10E-03	4.39E-01
RAB11FIP3	RAB11 family interacting protein 3	6.10E-03	5.63E-01
TBC1D5	TBC1 domain family member 5	6.10E-03	-3.60E-01
ARGLU1	arginine and glutamate rich 1	6.11E-03	-6.10E-01
RBM5	RNA binding motif protein 5	6.11E-03	-5.36E-01
TMEM159	transmembrane protein 159	6.11E-03	-4.22E-01
RPL36	ribosomal protein L36	6.12E-03	-3.78E-01

PTMS	parathymosin	6.12E-03	-5.10E-01
YIPF6	Yip1 domain family member 6	6.13E-03	-4.81E-01
HOXB2	homeobox B2	6.14E-03	7.19E-01
TNS4	tensin 4	6.14E-03	-2.16E-01
CCDC120	coiled-coil domain containing 120	6.14E-03	2.53E-01
SYAP1	synapse associated protein 1	6.15E-03	-4.02E-01
GABARAPL2	GABA type A receptor associated protein like 2	6.15E-03	-4.33E-01
SSBP2	single stranded DNA binding protein 2	6.16E-03	-2.84E-01
ADK	adenosine kinase	6.16E-03	3.49E-01
JADE1	jade family PHD finger 1	6.16E-03	2.37E-01
CS	citrate synthase	6.17E-03	3.32E-01
THOC2	THO complex 2	6.17E-03	-4.75E-01
ACTR3	ARP3 actin related protein 3 homolog	6.18E-03	6.44E-01
MOAP1	modulator of apoptosis 1	6.18E-03	-6.41E-01
SLC48A1	solute carrier family 48 member 1	6.19E-03	3.41E-01

S100A3	S100 calcium binding protein A3	6.19E-03	-6.93E-01
SUGCT	succinyl-CoA:glutarate-CoA transferase	6.19E-03	-3.53E-01
PAF1	PAF1 homolog, Paf1/RNA polymerase II complex component	6.20E-03	4.20E-01
HLCS	holocarboxylase synthetase	6.21E-03	-2.37E-01
ATP6V1F	ATPase H+ transporting V1 subunit F	6.22E-03	-4.05E-01
TP53INP2	tumor protein p53 inducible nuclear protein 2	6.22E-03	-5.51E-01
PPP1R11	protein phosphatase 1 regulatory inhibitor subunit 11	6.22E-03	5.77E-01
MIER2	MIER family member 2	6.23E-03	-3.75E-01
SEMA3F	semaphorin 3F	6.23E-03	-2.14E-01
MAPK11	mitogen-activated protein kinase 11	6.23E-03	-3.05E-01
CTSH	cathepsin H	6.23E-03	2.59E-01
HTATSF1	HIV-1 Tat specific factor 1	6.23E-03	2.60E-01
ACTRT3	actin related protein T3	6.23E-03	2.88E-01
CNPY2	canopy FGF signaling regulator 2	6.24E-03	-6.17E-01

FAM204A	family with sequence similarity 204 member A	6.24E-03	2.65E-01
PDPK1	3-phosphoinositide dependent protein kinase 1	6.24E-03	-5.33E-01
GRB2	growth factor receptor bound protein 2	6.24E-03	-3.32E-01
TAX1BP1	Tax1 binding protein 1	6.25E-03	3.33E-01
RAB40C	RAB40C, member RAS oncogene family	6.26E-03	-6.93E-01
LRRC32	leucine rich repeat containing 32	6.26E-03	-2.47E-01
SENP7	SUMO1/sentrin specific peptidase 7	6.27E-03	-4.51E-01
SLIRP	SRA stem-loop interacting RNA binding protein	6.27E-03	3.82E-01
RBSN	rabenosyn, RAB effector	6.27E-03	3.37E-01
TTLL7	tubulin tyrosine ligase like 7	6.27E-03	4.18E-01
ZMYND12	zinc finger MYND-type containing 12	6.28E-03	2.77E-01
PTBP2	polypyrimidine tract binding protein 2	6.28E-03	4.80E-01
CDK7	cyclin dependent kinase 7	6.29E-03	5.34E-01
CKLF	chemokine like factor	6.31E-03	7.93E-01
FBXL16	F-box and leucine rich repeat protein 16	6.31E-03	2.20E-01
TRAF3IP1	TRAF3 interacting protein 1	6.32E-03	3.14E-01

HYI	hydroxypyruvate isomerase (putative)	6.32E-03	2.34E-01
CLSPN	claspin	6.32E-03	2.50E-01
ZNF18	zinc finger protein 18	6.32E-03	6.28E-01
OSBPL1A	oxysterol binding protein like 1A	6.33E-03	-4.76E-01
ADD2	adducin 2	6.34E-03	-3.03E-01
PPP2R2A	protein phosphatase 2 regulatory subunit Balpha	6.34E-03	2.57E-01
ITCH	itchy E3 ubiquitin protein ligase	6.35E-03	2.03E-01
APH1A	aph-1 homolog A, gamma-secretase subunit	6.35E-03	-3.50E-01
SNORA57	small nucleolar RNA, H/ACA box 57	6.35E-03	-8.48E-01
CD83	CD83 molecule	6.35E-03	-2.28E-01
EFR3B	EFR3 homolog B	6.35E-03	-2.58E-01
MIR30B	microRNA 30b	6.35E-03	4.33E-01
RBMX	RNA binding motif protein, X-linked	6.36E-03	2.58E-01
ADK	adenosine kinase	6.36E-03	-8.56E-01
LAMP2	lysosomal associated membrane protein 2	6.36E-03	-3.96E-01

DROSHA	drosha ribonuclease III	6.36E-03	1.03
CD151	CD151 molecule (Raph blood group)	6.36E-03	3.08E-01
CYB561	cytochrome b561	6.37E-03	3.12E-01
PLXNB3	plexin B3	6.37E-03	-4.48E-01
SLC4A3	solute carrier family 4 member 3	6.38E-03	2.51E-01
TYW3	tRNA-yW synthesizing protein 3 homolog	6.38E-03	-5.23E-01
SLC9A2	solute carrier family 9 member A2	6.38E-03	2.78E-01
CPM	carboxypeptidase M	6.38E-03	-3.84E-01
CPA3	carboxypeptidase A3	6.39E-03	3.69E-01
TCEA1	transcription elongation factor A1	6.40E-03	7.80E-01
SMARCC2	SWI/SNF related, matrix associated, actin dependent regulator of chromatin subfamily c member 2	6.40E-03	2.08E-01
SLC35A3	solute carrier family 35 member A3	6.41E-03	4.09E-01
ZDHHC8	zinc finger DHHC-type containing 8	6.41E-03	-7.95E-01
OTOF	otoferlin	6.42E-03	-2.36E-01
CNPPD1	cyclin Pas1/PHO80 domain containing 1	6.42E-03	3.37E-01
FSIP1	fibrous sheath interacting protein 1	6.42E-03	3.39E-01

CKS2	CDC28 protein kinase regulatory subunit 2	6.43E-03	7.01E-01
GRK6	G protein-coupled receptor kinase 6	6.43E-03	3.43E-01
SLC35B3	solute carrier family 35 member B3	6.43E-03	2.44E-01
SFT2D2	SFT2 domain containing 2	6.43E-03	3.23E-01
NUP58	nucleoporin 58	6.44E-03	3.92E-01
GLRX2	glutaredoxin 2	6.46E-03	6.59E-01
TUBB	tubulin beta class I	6.46E-03	9.54E-01
SIM1	single-minded family bHLH transcription factor 1	6.46E-03	5.05E-01
TACC2	transforming acidic coiled-coil containing protein 2	6.46E-03	4.08E-01
PAXIP1-AS1	PAXIP1 antisense RNA 1 (head to head)	6.47E-03	-6.35E-01
CAMK2B	calcium/calmodulin dependent protein kinase II beta	6.48E-03	-2.94E-01
PNISR	PNN interacting serine and arginine rich protein	6.48E-03	-7.49E-01
HPRT1	hypoxanthine phosphoribosyltransferase 1	6.49E-03	4.35E-01
DYRK1B	dual specificity tyrosine phosphorylation regulated kinase 1B	6.50E-03	-4.18E-01
STEAP1	six transmembrane epithelial antigen of the prostate 1	6.50E-03	4.87E-01
PPP1R13B	protein phosphatase 1 regulatory subunit 13B	6.52E-03	-3.98E-01

DVL3	dishevelled segment polarity protein 3	6.52E-03	-4.99E-01
NCF1	neutrophil cytosolic factor 1	6.53E-03	2.08E-01
MOV10	Mov10 RISC complex RNA helicase	6.54E-03	-5.62E-01
MEAF6	MYST/Esa1 associated factor 6	6.55E-03	2.97E-01
TCEAL1	transcription elongation factor A like 1	6.55E-03	-4.57E-01
SCML1	sex comb on midleg-like 1 (Drosophila)	6.56E-03	-5.99E-01
IFNAR2	interferon alpha and beta receptor subunit 2	6.56E-03	2.85E-01
TRAPPC9	trafficking protein particle complex 9	6.56E-03	-5.70E-01
CHMP1A	charged multivesicular body protein 1A	6.57E-03	3.53E-01
CASC1	cancer susceptibility candidate 1	6.57E-03	2.22E-01
YWHAZ	tyrosine 3-monooxygenase/tryptophan 5-monooxygenase activation protein zeta	6.57E-03	-7.73E-01
BTBD2	BTB domain containing 2	6.58E-03	-6.50E-01
PTBP3	polypyrimidine tract binding protein 3	6.58E-03	-7.02E-01
PITPNM1	phosphatidylinositol transfer protein membrane associated 1	6.58E-03	-4.32E-01

SNUPN	snurportin 1	6.58E-03	4.36E-01
TAMM41	TAM41 mitochondrial translocator assembly and maintenance homolog	6.59E-03	3.88E-01
SUV39H2	suppressor of variegation 3-9 homolog 2	6.59E-03	2.72E-01
TNNT3	troponin T3, fast skeletal type	6.60E-03	-2.81E-01
DAPP1	dual adaptor of phosphotyrosine and 3-phosphoinositides 1	6.61E-03	-4.03E-01
HELZ	helicase with zinc finger	6.62E-03	-9.81E-01
RAB43	RAB43, member RAS oncogene family	6.63E-03	-2.99E-01
HOMER1	homer scaffolding protein 1	6.63E-03	8.08E-01
UGP2	UDP-glucose pyrophosphorylase 2	6.63E-03	-3.62E-01
SMARCA5	SWI/SNF related, matrix associated, actin dependent regulator of chromatin, subfamily a, member 5	6.64E-03	1.15
FOXD4	forkhead box D4	6.64E-03	-2.79E-01
VEGFB	vascular endothelial growth factor B	6.65E-03	-8.74E-01
ATG14	autophagy related 14	6.66E-03	3.41E-01
POLR3B	RNA polymerase III subunit B	6.67E-03	4.85E-01

CHMP3	charged multivesicular body protein 3	6.67E-03	-7.94E-01
INTS1	integrator complex subunit 1	6.67E-03	-7.68E-01
PIGH	phosphatidylinositol glycan anchor biosynthesis class H	6.67E-03	-3.50E-01
Sep-06	septin 6	6.68E-03	-7.40E-01
GPX3	glutathione peroxidase 3	6.69E-03	-3.22E-01
GNS	glucosamine (N-acetyl)-6-sulfatase	6.69E-03	-5.65E-01
AHCY	adenosylhomocysteinase	6.69E-03	-7.37E-01
RPS6KA3	ribosomal protein S6 kinase A3	6.69E-03	2.43E-01
C11orf63	chromosome 11 open reading frame 63	6.69E-03	2.94E-01
CEP85L	centrosomal protein 85 like	6.70E-03	-4.43E-01
ASUN	asunder, spermatogenesis regulator	6.71E-03	9.19E-01
MBOAT2	membrane bound O-acyltransferase domain containing 2	6.72E-03	-6.03E-01
COPS5	COP9 signalosome subunit 5	6.72E-03	2.76E-01
SMTN	smoothelin	6.74E-03	-2.23E-01

ZNF75D	zinc finger protein 75D	6.74E-03	-2.72E-01
DROSHA	drosha ribonuclease III	6.74E-03	7.14E-01
ITCH-IT1	ITCH intronic transcript 1	6.74E-03	-4.27E-01
SRP68	signal recognition particle 68	6.74E-03	-4.86E-01
SSB	Sjogren syndrome antigen B	6.74E-03	1.45
TSPAN9	tetraspanin 9	6.75E-03	-5.20E-01
CLN8	ceroid-lipofuscinosis, neuronal 8	6.75E-03	-2.98E-01
MANEA	mannosidase endo-alpha	6.76E-03	4.72E-01
MCAM	melanoma cell adhesion molecule	6.76E-03	2.69E-01
ZNF789	zinc finger protein 789	6.76E-03	-3.54E-01
SRI	sorcin	6.76E-03	3.14E-01
PGAP3	post-GPI attachment to proteins 3	6.77E-03	-4.77E-01
FBXO21	F-box protein 21	6.77E-03	-4.38E-01
WNT10B	Wnt family member 10B	6.77E-03	-3.45E-01

CNPY2	canopy FGF signaling regulator 2	6.78E-03	-5.17E-01
PCSK6	proprotein convertase subtilisin/kexin type 6	6.78E-03	2.05E-01
MOK	MOK protein kinase	6.80E-03	4.83E-01
SNORA11	small nucleolar RNA, H/ACA box 11	6.80E-03	-4.86E-01
ZSWIM7	zinc finger SWIM-type containing 7	6.81E-03	-5.89E-01
TRIM49	tripartite motif containing 49	6.81E-03	-2.58E-01
RNY5	RNA, Ro-associated Y5	6.82E-03	-6.17E-01
BRD9	bromodomain containing 9	6.83E-03	4.81E-01
APAF1	apoptotic peptidase activating factor 1	6.86E-03	-6.13E-01
PLOD3	procollagen-lysine,2-oxoglutarate 5-dioxygenase 3	6.86E-03	-4.70E-01
INPP5D	inositol polyphosphate-5-phosphatase D	6.87E-03	-6.58E-01
GORAB	golgin, RAB6 interacting	6.87E-03	4.95E-01
ACOT2	acyl-CoA thioesterase 2	6.87E-03	-1.21
TTLL4	tubulin tyrosine ligase like 4	6.88E-03	5.03E-01
LRRC1	leucine rich repeat containing 1	6.88E-03	5.16E-01

RAD51B	RAD51 paralog B	6.89E-03	3.15E-01
FDFT1	farnesyl-diphosphate farnesyltransferase 1	6.90E-03	-2.34E-01
RING1	ring finger protein 1	6.91E-03	-2.67E-01
PUM2	pumilio RNA binding family member 2	6.91E-03	-9.08E-01
S100A13	S100 calcium binding protein A13	6.91E-03	-9.78E-01
CORO7	coronin 7	6.92E-03	-3.28E-01
CRY1	cryptochrome circadian clock 1	6.92E-03	5.01E-01
TBC1D32	TBC1 domain family member 32	6.92E-03	-8.96E-01
DMWD	dystrophia myotonica, WD repeat containing	6.93E-03	-3.50E-01
TMEM91	transmembrane protein 91	6.93E-03	-8.49E-01
SNRNP35	small nuclear ribonucleoprotein U11/U12 subunit 35	6.94E-03	-5.96E-01
SRP19	signal recognition particle 19	6.94E-03	2.45E-01
AIF1	allograft inflammatory factor 1	6.94E-03	2.28E-01
PIN4P1	peptidylprolyl cis/trans isomerase, NIMA-interacting 4 pseudogene 1	6.94E-03	-4.32E-01

RSF1	remodeling and spacing factor 1	6.94E-03	5.60E-01
PDCD4-AS1	PDCD4 antisense RNA 1	6.95E-03	-2.64E-01
SNORD6	small nucleolar RNA, C/D box 6	6.95E-03	-2.94E-01
TMEM181	transmembrane protein 181	6.98E-03	-4.50E-01
CUTA	cutA divalent cation tolerance homolog	6.98E-03	-3.63E-01
LAMTOR1	late endosomal/lysosomal adaptor, MAPK and MTOR activator 1	7.00E-03	-6.30E-01
IDE	insulin degrading enzyme	7.02E-03	5.54E-01
ELK1	ELK1, ETS transcription factor	7.03E-03	-3.42E-01
PLP1	proteolipid protein 1	7.03E-03	-2.86E-01
BOP1	block of proliferation 1	7.04E-03	3.79E-01
MAP4K4	mitogen-activated protein kinase kinase kinase kinase 4	7.04E-03	-5.46E-01
TUBE1	tubulin epsilon 1	7.04E-03	5.05E-01
SIX5	SIX homeobox 5	7.04E-03	-6.81E-01
SCCPDH	saccharopine dehydrogenase (putative)	7.05E-03	-1.90E-01

RABEPK	Rab9 effector protein with kelch motifs	7.06E-03	4.26E-01
MPG	N-methylpurine DNA glycosylase	7.06E-03	-2.86E-01
C15orf57	chromosome 15 open reading frame 57	7.07E-03	-5.85E-01
WDR33	WD repeat domain 33	7.07E-03	4.71E-01
ZNF670	zinc finger protein 670	7.08E-03	2.52E-01
PDE4A	phosphodiesterase 4A	7.08E-03	3.66E-01
MIR1253	microRNA 1253	7.09E-03	-2.97E-01
SOCS6	suppressor of cytokine signaling 6	7.09E-03	2.67E-01
SSBP1	single stranded DNA binding protein 1	7.09E-03	4.62E-01
AP2M1	adaptor related protein complex 2 mu 1 subunit	7.09E-03	-3.45E-01
SNHG5	small nucleolar RNA host gene 5	7.10E-03	-3.40E-01
PIP5K1B	phosphatidylinositol-4-phosphate 5-kinase type 1 beta	7.11E-03	-2.79E-01
TTC39B	tetratricopeptide repeat domain 39B	7.11E-03	3.53E-01
C4orf32	chromosome 4 open reading frame 32	7.11E-03	2.64E-01
KLHL8	kelch like family member 8	7.11E-03	-3.88E-01

PEA15	phosphoprotein enriched in astrocytes 15	7.12E-03	5.86E-01
EDIL3	EGF like repeats and discoidin domains 3	7.12E-03	-1.01
NFKB2	nuclear factor kappa B subunit 2	7.13E-03	4.26E-01
POGLUT1	protein O-glucosyltransferase 1	7.13E-03	-5.71E-01
SNORA62	small nucleolar RNA, H/ACA box 62	7.14E-03	3.18E-01
TMEM203	transmembrane protein 203	7.14E-03	4.33E-01
ZNF410	zinc finger protein 410	7.15E-03	6.83E-01
SNORD3A	small nucleolar RNA, C/D box 3A	7.16E-03	-9.62E-01
APLP2	amyloid beta precursor like protein 2	7.16E-03	5.93E-01
FBXO43	F-box protein 43	7.17E-03	2.45E-01
HIF1A	hypoxia inducible factor 1 alpha subunit	7.17E-03	1.25
ERLEC1	endoplasmic reticulum lectin 1	7.17E-03	-5.66E-01
DLEU1	deleted in lymphocytic leukemia 1	7.17E-03	-5.93E-01
KAT5	lysine acetyltransferase 5	7.18E-03	-2.95E-01
TMEM156	transmembrane protein 156	7.19E-03	-5.45E-01
SNRPE	small nuclear ribonucleoprotein polypeptide E	7.21E-03	9.04E-01

TAOK2	TAO kinase 2	7.21E-03	3.28E-01
ADAMTSL4	ADAMTS like 4	7.21E-03	-5.07E-01
MYO10	myosin X	7.22E-03	2.23E-01
TLN2	talin 2	7.23E-03	2.04E-01
COX19	COX19, cytochrome c oxidase assembly factor	7.24E-03	-4.50E-01
ZFYVE19	zinc finger FYVE-type containing 19	7.24E-03	2.53E-01
EMC3	ER membrane protein complex subunit 3	7.24E-03	-3.29E-01
Mar-10	membrane associated ring-CH-type finger 10	7.25E-03	2.21E-01
ELF3	E74 like ETS transcription factor 3	7.26E-03	2.80E-01
PDP2	pyruvate dehyrogenase phosphatase catalytic subunit 2	7.26E-03	-2.73E-01
TROVE2	TROVE domain family member 2	7.26E-03	8.39E-01
CEP44	centrosomal protein 44	7.27E-03	4.53E-01
AQP11	aquaporin 11	7.27E-03	2.42E-01
NCOA5	nuclear receptor coactivator 5	7.29E-03	5.22E-01
C17orf100	chromosome 17 open reading frame 100	7.30E-03	-2.69E-01
COA1	cytochrome c oxidase assembly factor 1 homolog	7.30E-03	3.15E-01

INTS12	integrator complex subunit 12	7.30E-03	-3.75E-01
ZSCAN22	zinc finger and SCAN domain containing 22	7.31E-03	2.53E-01
RTBDN	retbindin	7.32E-03	-2.75E-01
DAP	death associated protein	7.32E-03	5.05E-01
RRAGA	Ras related GTP binding A	7.32E-03	-3.49E-01
ZNF234	zinc finger protein 234	7.32E-03	-5.62E-01
POLR3F	RNA polymerase III subunit F	7.32E-03	5.08E-01
CBLN3	cerebellin 3 precursor	7.33E-03	-2.81E-01
RABIF	RAB interacting factor	7.33E-03	3.29E-01
SIAE	sialic acid acetylesterase	7.35E-03	2.93E-01
ARID4B	AT-rich interaction domain 4B	7.35E-03	-1.1
MTRR	5-methyltetrahydrofolate-homocysteine methyltransferase reductase	7.36E-03	3.20E-01
CCDC184	coiled-coil domain containing 184	7.36E-03	-3.00E-01
SCARNA12	small Cajal body-specific RNA 12	7.36E-03	-2.96E-01
CNOT6L	CCR4-NOT transcription complex subunit 6 like	7.38E-03	3.70E-01

SLC2A10	solute carrier family 2 member 10	7.39E-03	3.52E-01
STOX1	storkhead box 1	7.39E-03	3.27E-01
LCN12	lipocalin 12	7.39E-03	-2.26E-01
HAT1	histone acetyltransferase 1	7.40E-03	6.81E-01
MBTPS1	membrane bound transcription factor peptidase, site 1	7.41E-03	-4.76E-01
RAB2A	RAB2A, member RAS oncogene family	7.42E-03	4.41E-01
TCEA3	transcription elongation factor A3	7.42E-03	-2.53E-01
ARPC4	actin related protein 2/3 complex subunit 4	7.42E-03	-8.23E-01
HSP90B1	heat shock protein 90 beta family member 1	7.42E-03	-4.18E-01
TIMM10	translocase of inner mitochondrial membrane 10 homolog (yeast)	7.42E-03	1.08
HDAC1	histone deacetylase 1	7.42E-03	-4.39E-01
COPS4	COP9 signalosome subunit 4	7.43E-03	4.96E-01
SRY	sex determining region Y	7.44E-03	2.03E-01
BMPR1A	bone morphogenetic protein receptor type 1A	7.45E-03	1.05
ZNF516	zinc finger protein 516	7.45E-03	-2.62E-01

TMEM60	transmembrane protein 60	7.46E-03	-4.14E-01
UBE2E1	ubiquitin conjugating enzyme E2 E1	7.46E-03	2.82E-01
SNORD89	small nucleolar RNA, C/D box 89	7.46E-03	2.00E-01
ARHGEF16	Rho guanine nucleotide exchange factor 16	7.46E-03	5.22E-01
C1orf106	chromosome 1 open reading frame 106	7.47E-03	6.47E-01
PLEKHF1	pleckstrin homology and FYVE domain containing 1	7.47E-03	-4.34E-01
ZNF582	zinc finger protein 582	7.48E-03	2.51E-01
SNORD18C	small nucleolar RNA, C/D box 18C	7.50E-03	3.06E-01
OCEL1	occludin/ELL domain containing 1	7.50E-03	-6.50E-01
TMEM241	transmembrane protein 241	7.51E-03	-3.21E-01
SFXN1	sideroflexin 1	7.53E-03	7.60E-01
HNRNPH3	heterogeneous nuclear ribonucleoprotein H3	7.53E-03	7.17E-01
CHMP2A	charged multivesicular body protein 2A	7.54E-03	-9.91E-01
CLCNKA	chloride voltage-gated channel Ka	7.54E-03	-3.53E-01
SMARCA5	SWI/SNF related, matrix associated, actin dependent regulator of chromatin, subfamily a, member 5	7.54E-03	1.06

FABP5P3	fatty acid binding protein 5 pseudogene 3	7.54E-03	2.24E-01
KLC1	kinesin light chain 1	7.54E-03	3.15E-01
SAMD11	sterile alpha motif domain containing 11	7.56E-03	-4.56E-01
C14orf79	chromosome 14 open reading frame 79	7.57E-03	2.66E-01
KMT2E	lysine methyltransferase 2E	7.57E-03	-7.01E-01
MIR599	microRNA 599	7.58E-03	-3.81E-01
MATK	megakaryocyte-associated tyrosine kinase	7.58E-03	-3.99E-01
CAMSAP3	calmodulin regulated spectrin associated protein family member 3	7.58E-03	2.86E-01
CTSL	cathepsin L	7.59E-03	-3.96E-01
IDH3B	isocitrate dehydrogenase 3 (NAD(+)) beta	7.60E-03	-3.97E-01
IL25	interleukin 25	7.60E-03	-6.56E-01
CKAP5	cytoskeleton associated protein 5	7.61E-03	7.85E-01
SEMA4C	semaphorin 4C	7.61E-03	-4.07E-01
RPTOR	regulatory associated protein of MTOR complex 1	7.62E-03	-3.00E-01

SLC35D2	solute carrier family 35 member D2	7.62E-03	-5.13E-01
OR7E156P	olfactory receptor family 7 subfamily E member 156 pseudogene	7.62E-03	-5.99E-01
RBM15	RNA binding motif protein 15	7.62E-03	2.86E-01
XYLT2	xylosyltransferase 2	7.63E-03	-5.14E-01
DDAH1	dimethylarginine dimethylaminohydrolase 1	7.63E-03	1.05
EIF2AK4	eukaryotic translation initiation factor 2 alpha kinase 4	7.63E-03	-5.02E-01
PPP2R5B	protein phosphatase 2 regulatory subunit B'beta	7.63E-03	-2.98E-01
AGPAT2	1-acylglycerol-3-phosphate O-acyltransferase 2	7.64E-03	-3.82E-01
LARP1B	La ribonucleoprotein domain family member 1B	7.64E-03	2.96E-01
MOB4	MOB family member 4, phocein	7.65E-03	5.55E-01
ARMT1	acidic residue methyltransferase 1	7.65E-03	2.56E-01
DCLRE1C	DNA cross-link repair 1C	7.67E-03	-6.17E-01
DPP7	dipeptidyl peptidase 7	7.67E-03	3.10E-01
C10orf76	chromosome 10 open reading frame 76	7.68E-03	-2.19E-01
GSDMB	gasdermin B	7.68E-03	-2.50E-01

C17orf96	chromosome 17 open reading frame 96	7.69E-03	-3.67E-01
PTH	parathyroid hormone	7.69E-03	2.03E-01
ARID4B	AT-rich interaction domain 4B	7.70E-03	-5.65E-01
ABLIM1	actin binding LIM protein 1	7.70E-03	5.53E-01
ANXA2	annexin A2	7.71E-03	3.30E-01
CMTM4	CKLF like MARVEL transmembrane domain containing 4	7.71E-03	-2.50E-01
DNAJC10	DnaJ heat shock protein family (Hsp40) member C10	7.72E-03	9.47E-01
CCNY	cyclin Y	7.72E-03	-7.96E-01
WDR5B	WD repeat domain 5B	7.72E-03	-3.01E-01
GTF3C5	general transcription factor IIIC subunit 5	7.73E-03	3.86E-01
CHRAC1	chromatin accessibility complex 1	7.74E-03	3.21E-01
TTC21A	tetratricopeptide repeat domain 21A	7.74E-03	-2.50E-01
STRAP	serine/threonine kinase receptor associated protein	7.75E-03	4.96E-01
MSMO1	methylsterol monooxygenase 1	7.75E-03	2.99E-01
SDHAP1	succinate dehydrogenase complex flavoprotein subunit A pseudogene 1	7.76E-03	3.56E-01

P4HA1	prolyl 4-hydroxylase subunit alpha 1	7.76E-03	-4.44E-01
TUBB2A	tubulin beta 2A class IIa	7.77E-03	3.91E-01
FAM26E	family with sequence similarity 26 member E	7.77E-03	1.95E-01
TMEM33	transmembrane protein 33	7.78E-03	4.26E-01
GATB	glutamyl-tRNA amidotransferase subunit B	7.78E-03	-4.71E-01
GPM6A	glycoprotein M6A	7.80E-03	-2.74E-01
TBC1D8B	TBC1 domain family member 8B	7.81E-03	-4.64E-01
RNF26	ring finger protein 26	7.82E-03	5.69E-01
SFXN3	sideroflexin 3	7.82E-03	2.45E-01
AMT	aminomethyltransferase	7.82E-03	-4.14E-01
ERVMER34-1	endogenous retrovirus group MER34 member 1	7.82E-03	2.91E-01
WNT4	Wnt family member 4	7.82E-03	-2.50E-01
OCRL	OCRL, inositol polyphosphate-5-phosphatase	7.84E-03	9.25E-01
FAM207A	family with sequence similarity 207 member A	7.84E-03	3.20E-01
TP53AIP1	tumor protein p53 regulated apoptosis inducing protein 1	7.84E-03	-2.16E-01

POLR2J	RNA polymerase II subunit J	7.85E-03	4.14E-01
RPL11	ribosomal protein L11	7.86E-03	-2.60E-01
GFI1	growth factor independent 1 transcriptional repressor	7.86E-03	2.31E-01
PAPOLA	poly(A) polymerase alpha	7.87E-03	6.45E-01
LOC93622	Morf4 family associated protein 1 like 1 pseudogene	7.87E-03	3.92E-01
PEX7	peroxisomal biogenesis factor 7	7.87E-03	4.44E-01
UBE3D	ubiquitin protein ligase E3D	7.87E-03	-2.38E-01
PLEC	plectin	7.88E-03	2.33E-01
PDK1	pyruvate dehydrogenase kinase 1	7.89E-03	-4.62E-01
ELAC2	elaC ribonuclease Z 2	7.91E-03	3.72E-01
OR4L1	olfactory receptor family 4 subfamily L member 1	7.92E-03	-1.95E-01
SLC2A5	solute carrier family 2 member 5	7.93E-03	2.50E-01
CCNYL1	cyclin Y like 1	7.93E-03	3.09E-01
NME1-NME2	NME1-NME2 readthrough	7.93E-03	-4.30E-01
HMGA2	high mobility group AT-hook 2	7.93E-03	3.76E-01

ZNF770	zinc finger protein 770	7.93E-03	-2.74E-01
WRN	Werner syndrome RecQ like helicase	7.95E-03	4.08E-01
HLA-F	major histocompatibility complex, class I, F	7.95E-03	3.38E-01
WSB2	WD repeat and SOCS box containing 2	7.96E-03	4.34E-01
ERMARD	ER membrane associated RNA degradation	7.97E-03	3.04E-01
STX3	syntaxin 3	7.97E-03	2.65E-01
ZNF28	zinc finger protein 28	7.99E-03	3.05E-01
PPDPF	pancreatic progenitor cell differentiation and proliferation factor	7.99E-03	-5.91E-01
POLR3K	RNA polymerase III subunit K	8.00E-03	8.05E-01
G3BP2	G3BP stress granule assembly factor 2	8.00E-03	9.07E-01
C3orf38	chromosome 3 open reading frame 38	8.02E-03	3.71E-01
PRR5	proline rich 5	8.03E-03	-2.73E-01
OTUD5	OTU deubiquitinase 5	8.05E-03	-2.34E-01
RAB15	RAB15, member RAS oncogene family	8.05E-03	3.64E-01
KCTD11	potassium channel tetramerization domain containing 11	8.05E-03	-4.29E-01
TOP1	topoisomerase (DNA) I	8.06E-03	3.47E-01

ASTE1	asteroid homolog 1 (Drosophila)	8.06E-03	2.54E-01
IP6K2	inositol hexakisphosphate kinase 2	8.06E-03	-8.60E-01
FBLN1	fibulin 1	8.06E-03	-3.38E-01
TRIM11	tripartite motif containing 11	8.07E-03	-4.68E-01
SNORA73B	small nucleolar RNA, H/ACA box 73B	8.08E-03	-2.97E-01
CCDC50	coiled-coil domain containing 50	8.08E-03	-3.26E-01
LRWD1	leucine rich repeats and WD repeat domain containing 1	8.10E-03	6.44E-01
CITED1	Cbp/p300 interacting transactivator with Glu/Asp rich carboxy-terminal domain 1	8.11E-03	-2.42E-01
IDS	iduronate 2-sulfatase	8.12E-03	-2.55E-01
HDAC4	histone deacetylase 4	8.13E-03	-2.82E-01
KANSL1L	KAT8 regulatory NSL complex subunit 1 like	8.14E-03	-2.15E-01
RNF213	ring finger protein 213	8.14E-03	-5.20E-01
UBE2E3	ubiquitin conjugating enzyme E2 E3	8.15E-03	4.48E-01
ZNF736	zinc finger protein 736	8.15E-03	4.72E-01

DQX1	DEAQ-box RNA dependent ATPase 1	8.15E-03	-3.56E-01
TMED5	transmembrane p24 trafficking protein 5	8.16E-03	4.12E-01
DHRS4-AS1	DHRS4 antisense RNA 1	8.16E-03	-6.08E-01
SNORD69	small nucleolar RNA, C/D box 69	8.16E-03	3.65E-01
SSBP3	single stranded DNA binding protein 3	8.16E-03	-5.70E-01
MATN2	matrilin 2	8.17E-03	2.20E-01
BRD3	bromodomain containing 3	8.19E-03	-4.97E-01
SGK3	serum/glucocorticoid regulated kinase family member 3	8.19E-03	3.94E-01
AGFG1	ArfGAP with FG repeats 1	8.20E-03	-3.80E-01
MYBBP1A	MYB binding protein 1a	8.21E-03	4.11E-01
DBT	dihydrolipoamide branched chain transacylase E2	8.21E-03	-4.87E-01
C12orf4	chromosome 12 open reading frame 4	8.22E-03	5.22E-01
LARP7	La ribonucleoprotein domain family member 7	8.22E-03	4.71E-01
ZNF765	zinc finger protein 765	8.23E-03	2.19E-01
NUP188	nucleoporin 188	8.24E-03	6.57E-01

DNAJC27	DnaJ heat shock protein family (Hsp40) member C27	8.25E-03	-6.25E-01
LINC01315	long intergenic non-protein coding RNA 1315	8.26E-03	-5.14E-01
LCE3C	late cornified envelope 3C	8.27E-03	-1.90E-01
C11orf65	chromosome 11 open reading frame 65	8.27E-03	2.44E-01
CTTN	cortactin	8.28E-03	-3.76E-01
TUBGCP2	tubulin gamma complex associated protein 2	8.28E-03	3.54E-01
RPS9	ribosomal protein S9	8.29E-03	-5.87E-01
GATSL2	GATS protein like 2	8.31E-03	-2.09E-01
LOC100190986	uncharacterized LOC100190986	8.31E-03	-4.27E-01
GSTM1	glutathione S-transferase mu 1	8.31E-03	-7.70E-01
OXLD1	oxidoreductase like domain containing 1	8.33E-03	-7.10E-01
GTPBP3	GTP binding protein 3 (mitochondrial)	8.34E-03	-2.31E-01
PDRG1	p53 and DNA damage regulated 1	8.36E-03	5.72E-01
KRR1	KRR1, small subunit processome component homolog	8.37E-03	4.73E-01

HACD2	3-hydroxyacyl-CoA dehydratase 2	8.37E-03	6.55E-01
HIST1H3H	histone cluster 1, H3h	8.37E-03	-5.76E-01
SOX7	SRY-box 7	8.38E-03	2.35E-01
FBXO15	F-box protein 15	8.38E-03	5.45E-01
SNORD30	small nucleolar RNA, C/D box 30	8.38E-03	6.86E-01
C7orf26	chromosome 7 open reading frame 26	8.38E-03	2.90E-01
ZNF737	zinc finger protein 737	8.39E-03	3.07E-01
ZNF23	zinc finger protein 23	8.39E-03	3.33E-01
QKI	QKI, KH domain containing RNA binding	8.40E-03	-1.99E-01
AEBP1	AE binding protein 1	8.40E-03	3.42E-01
SDC4	syndecan 4	8.41E-03	2.60E-01
CCPG1	cell cycle progression 1	8.41E-03	-4.81E-01
ATP8B3	ATPase phospholipid transporting 8B3	8.41E-03	2.37E-01
DHDDS	dehydrodolichyl diphosphate synthase subunit	8.42E-03	4.37E-01
CLIP1	CAP-Gly domain containing linker protein 1	8.43E-03	3.31E-01
EIF5	eukaryotic translation initiation factor 5	8.43E-03	-1.94E-01

PCED1B	PC-esterase domain containing 1B	8.43E-03	2.74E-01
LRRC37B	leucine rich repeat containing 37B	8.44E-03	-2.64E-01
LOC646626	uncharacterized LOC646626	8.44E-03	-2.69E-01
EIF2AK1	eukaryotic translation initiation factor 2 alpha kinase 1	8.44E-03	4.32E-01
GFOD1	glucose-fructose oxidoreductase domain containing 1	8.46E-03	-2.86E-01
MZT1	mitotic spindle organizing protein 1	8.46E-03	2.80E-01
AK2	adenylate kinase 2	8.47E-03	6.01E-01
JRK	Jrk helix-turn-helix protein	8.47E-03	2.58E-01
ABL2	ABL proto-oncogene 2, non-receptor tyrosine kinase	8.47E-03	-2.01E-01
QSOX2	quiescin sulfhydryl oxidase 2	8.49E-03	-4.91E-01
CBX7	chromobox 7	8.50E-03	5.32E-01
HMSD	histocompatibility minor serpin domain containing	8.50E-03	2.42E-01
PSMB6	proteasome subunit beta 6	8.50E-03	5.14E-01
EGFEM1P	EGF like and EMI domain containing 1, pseudogene	8.52E-03	-3.47E-01
THAP6	THAP domain containing 6	8.52E-03	2.76E-01

ZC3H14	zinc finger CCCH-type containing 14	8.52E-03	6.53E-01
S1PR1	sphingosine-1-phosphate receptor 1	8.54E-03	-3.59E-01
RNF150	ring finger protein 150	8.55E-03	-7.56E-01
CXorf38	chromosome X open reading frame 38	8.55E-03	4.75E-01
ZNF768	zinc finger protein 768	8.55E-03	-2.73E-01
EEF2KMT	eukaryotic elongation factor 2 lysine methyltransferase	8.56E-03	5.24E-01
ERICH1	glutamate rich 1	8.57E-03	2.91E-01
PCLO	piccolo presynaptic cytomatrix protein	8.58E-03	-3.10E-01
TNFSF14	tumor necrosis factor superfamily member 14	8.61E-03	-6.82E-01
MTMR9	myotubularin related protein 9	8.61E-03	-4.42E-01
ZNF681	zinc finger protein 681	8.61E-03	1.06
DENND1A	DENN domain containing 1A	8.61E-03	-3.22E-01
CSNK1E	casein kinase 1 epsilon	8.62E-03	-3.46E-01
SNORD58B	small nucleolar RNA, C/D box 58B	8.64E-03	-3.83E-01

NAT9	N-acetyltransferase 9 (putative)	8.65E-03	-7.83E-01
HAUS6	HAUS augmin like complex subunit 6	8.66E-03	3.87E-01
SFT2D1	SFT2 domain containing 1	8.66E-03	-2.70E-01
ULBP2	UL16 binding protein 2	8.66E-03	3.45E-01
RAB3IL1	RAB3A interacting protein like 1	8.66E-03	-4.65E-01
POGK	pogo transposable element with KRAB domain	8.67E-03	4.57E-01
NT5DC1	5'-nucleotidase domain containing 1	8.67E-03	4.94E-01
PNMA6A	paraneoplastic Ma antigen family member 6A	8.68E-03	2.98E-01
CDK2	cyclin dependent kinase 2	8.69E-03	5.27E-01
FTH1	ferritin heavy chain 1	8.70E-03	-5.21E-01
SLC25A5	solute carrier family 25 member 5	8.70E-03	2.15E-01
RAB4B	RAB4B, member RAS oncogene family	8.70E-03	-4.46E-01
DNAJC19	DnaJ heat shock protein family (Hsp40) member C19	8.71E-03	5.38E-01
LOC105375847	uncharacterized LOC105375847	8.71E-03	3.27E-01
FAM86DP	family with sequence similarity 86, member D, pseudogene	8.71E-03	3.77E-01
AMIGO1	adhesion molecule with Ig like domain 1	8.73E-03	2.36E-01

GJA1	gap junction protein alpha 1	8.74E-03	4.25E-01
SNAI3	snail family transcriptional repressor 3	8.74E-03	-2.40E-01
TAF5L	TATA-box binding protein associated factor 5 like	8.75E-03	5.70E-01
OPA3	optic atrophy 3 (autosomal recessive, with chorea and spastic paraplegia)	8.76E-03	-1.12
TM9SF1	transmembrane 9 superfamily member 1	8.76E-03	-3.25E-01
TBL1X	transducin (beta)-like 1X-linked	8.77E-03	-6.75E-01
PORCN	porcupine homolog (Drosophila)	8.79E-03	-2.09E-01
SLC25A15	solute carrier family 25 member 15	8.79E-03	-4.73E-01
CHI3L1	chitinase 3 like 1	8.80E-03	-3.39E-01
HMG20B	high mobility group 20B	8.80E-03	-5.78E-01
SRGAP3	SLIT-ROBO Rho GTPase activating protein 3	8.81E-03	-2.60E-01
CYP2S1	cytochrome P450 family 2 subfamily S member 1	8.82E-03	2.52E-01
GPR137C	G protein-coupled receptor 137C	8.83E-03	3.66E-01
GPR89B	G protein-coupled receptor 89B	8.84E-03	7.48E-01
POLD1	DAN polymerase delta 1, catalytic subunit	8.84E-03	5.69E-01

TRIM48	tripartite motif containing 48	8.84E-03	-3.86E-01
SLC1A5	solute carrier family 1 member 5	8.85E-03	4.17E-01
COA6	cytochrome c oxidase assembly factor 6	8.86E-03	-5.39E-01
ABHD1	abhydrolase domain containing 1	8.86E-03	2.22E-01
FAM127A	family with sequence similarity 127 member A	8.86E-03	-4.86E-01
MRPL57	mitochondrial ribosomal protein L57	8.87E-03	-5.18E-01
YOD1	YOD1 deubiquitinase	8.89E-03	2.58E-01
DNAJC10	DnaJ heat shock protein family (Hsp40) member C10	8.90E-03	6.15E-01
RNASET2	ribonuclease T2	8.91E-03	-8.11E-01
ITPRIP	inositol 1,4,5-trisphosphate receptor interacting protein	8.92E-03	3.99E-01
DNPH1	2'-deoxynucleoside 5'-phosphate N-hydrolase 1	8.92E-03	-6.60E-01
NVL	nuclear VCP-like	8.92E-03	-2.95E-01
ZNF250	zinc finger protein 250	8.94E-03	2.89E-01
CNBD2	cyclic nucleotide binding domain containing 2	8.94E-03	-2.02E-01
MCL1	BCL2 family apoptosis regulator	8.95E-03	6.60E-01

ZDHHC17	zinc finger DHHC-type containing 17	8.95E-03	-3.23E-01
NUDT21	nudix hydrolase 21	8.95E-03	7.10E-01
SLC12A5	solute carrier family 12 member 5	8.96E-03	-1.93E-01
IREB2	iron responsive element binding protein 2	8.96E-03	5.83E-01
SPRY1	sprouty RTK signaling antagonist 1	8.97E-03	4.03E-01
OLFM2	olfactomedin 2	8.97E-03	-2.70E-01
FAM49B	family with sequence similarity 49 member B	8.97E-03	7.36E-01
KLHDC4	kelch domain containing 4	8.99E-03	4.07E-01
PPP1R26	protein phosphatase 1 regulatory subunit 26	8.99E-03	3.15E-01
OSBPL9	oxysterol binding protein like 9	9.00E-03	8.88E-01
MOXD1	monooxygenase DBH like 1	9.02E-03	3.40E-01
POLM	DNA polymerase mu	9.02E-03	-3.11E-01
THAP1	THAP domain containing 1	9.04E-03	4.38E-01
TCF25	transcription factor 25	9.04E-03	-4.74E-01
PHF11	PHD finger protein 11	9.04E-03	-4.87E-01

FAM168A	family with sequence similarity 168 member A	9.05E-03	-5.06E-01
OAZ3	ornithine decarboxylase antizyme 3	9.06E-03	2.20E-01
CHMP5	charged multivesicular body protein 5	9.07E-03	3.28E-01
FMC1	formation of mitochondrial complex V assembly factor 1 homolog	9.08E-03	-6.61E-01
TM6SF2	transmembrane 6 superfamily member 2	9.09E-03	-2.76E-01
ST13P4	suppression of tumorigenicity 13 (colon carcinoma) (Hsp70 interacting protein) pseudogene 4	9.09E-03	-2.78E-01
SLC25A18	solute carrier family 25 member 18	9.11E-03	-2.04E-01
RUNDC3B	RUN domain containing 3B	9.11E-03	3.24E-01
TXN	thioredoxin	9.12E-03	3.72E-01
DUSP6	dual specificity phosphatase 6	9.12E-03	4.34E-01
FAM92B	family with sequence similarity 92 member B	9.12E-03	-3.13E-01
C1orf43	chromosome 1 open reading frame 43	9.13E-03	-5.34E-01
SNRPC	small nuclear ribonucleoprotein polypeptide C	9.13E-03	4.00E-01
PTCD1	pentatricopeptide repeat domain 1	9.13E-03	5.39E-01
CYP2U1	cytochrome P450 family 2 subfamily U member 1	9.15E-03	2.26E-01

RFX5	regulatory factor X5	9.15E-03	-2.24E-01
HAPLN4	hyaluronan and proteoglycan link protein 4	9.15E-03	-3.57E-01
ZNF234	zinc finger protein 234	9.17E-03	-5.44E-01
AP2A1	adaptor related protein complex 2 alpha 1 subunit	9.17E-03	-2.72E-01
PPFIBP1	PPFIA binding protein 1	9.18E-03	-4.22E-01
SIK2	salt inducible kinase 2	9.19E-03	3.24E-01
PTGR2	prostaglandin reductase 2	9.19E-03	-2.94E-01
ALOX12B	arachidonate 12-lipoxygenase, 12R type	9.20E-03	-2.03E-01
PIAS2	protein inhibitor of activated STAT 2	9.20E-03	5.62E-01
C11orf63	chromosome 11 open reading frame 63	9.21E-03	2.03E-01
RBM38	RNA binding motif protein 38	9.22E-03	-5.87E-01
CCDC93	coiled-coil domain containing 93	9.23E-03	4.04E-01
MANEAL	mannosidase endo-alpha like	9.23E-03	2.58E-01
ZC2HC1C	zinc finger C2HC-type containing 1C	9.24E-03	5.50E-01

KLHL30	kelch like family member 30	9.25E-03	-2.49E-01
LMBRD2	LMBR1 domain containing 2	9.26E-03	2.14E-01
SIGLEC6	sialic acid binding Ig like lectin 6	9.27E-03	-1.93E-01
PKDCC	protein kinase domain containing, cytoplasmic	9.27E-03	-3.89E-01
CYB5RL	cytochrome b5 reductase like	9.27E-03	2.64E-01
TIMM23	translocase of inner mitochondrial membrane 23	9.27E-03	4.22E-01
POLR3E	RNA polymerase III subunit E	9.27E-03	2.02E-01
TANGO2	transport and golgi organization 2 homolog	9.28E-03	3.80E-01
DPRXP4	divergent-paired related homeobox pseudogene 4	9.28E-03	-3.34E-01
CS	citrate synthase	9.28E-03	4.69E-01
MRPL49	mitochondrial ribosomal protein L49	9.29E-03	-2.00E-01
MYO19	myosin XIX	9.29E-03	3.31E-01
UNC50	unc-50 inner nuclear membrane RNA binding protein	9.29E-03	-3.41E-01
CADM4	cell adhesion molecule 4	9.30E-03	2.54E-01
PYCR2	pyrroline-5-carboxylate reductase family member 2	9.31E-03	-2.63E-01

RPL37	ribosomal protein L37	9.31E-03	-3.45E-01
SMS	spermine synthase	9.32E-03	3.06E-01
CENPC	centromere protein C	9.32E-03	6.23E-01
CAMK2B	calcium/calmodulin dependent protein kinase II beta	9.32E-03	-3.31E-01
EEF2	eukaryotic translation elongation factor 2	9.33E-03	-4.49E-01
ALKBH1	alkB homolog 1, histone H2A dioxygenase	9.34E-03	3.20E-01
TRRAP	transformation/transcription domain associated protein	9.34E-03	-5.63E-01
KYNU	kynureninase	9.35E-03	2.45E-01
ZNF584	zinc finger protein 584	9.36E-03	3.30E-01
ZNF222	zinc finger protein 222	9.36E-03	3.82E-01
DTWD1	DTW domain containing 1	9.37E-03	5.21E-01
DKKL1	dickkopf like acrosomal protein 1	9.38E-03	-4.82E-01
FTL	ferritin light chain	9.39E-03	-4.15E-01
IFI27L1	interferon alpha inducible protein 27 like 1	9.41E-03	5.58E-01
WDR4	WD repeat domain 4	9.41E-03	2.94E-01

GPANK1	G-patch domain and ankyrin repeats 1	9.43E-03	-2.49E-01
RPP30	ribonuclease P/MRP subunit p30	9.44E-03	2.52E-01
RPL8	ribosomal protein L8	9.47E-03	-3.37E-01
DCUN1D2	defective in cullin neddylation 1 domain containing 2	9.49E-03	-2.15E-01
MORC4	MORC family CW-type zinc finger 4	9.49E-03	7.23E-01
UVRAG	UV radiation resistance associated	9.49E-03	-4.46E-01
R3HDM4	R3H domain containing 4	9.50E-03	-4.37E-01
PKIG	protein kinase (cAMP-dependent, catalytic) inhibitor gamma	9.50E-03	2.75E-01
ARMC12	armadillo repeat containing 12	9.50E-03	-3.55E-01
TECR	trans-2,3-enoyl-CoA reductase	9.51E-03	-4.77E-01
NUBPL	nucleotide binding protein like	9.51E-03	-5.60E-01
CCDC102A	coiled-coil domain containing 102A	9.52E-03	-5.66E-01
Sep-15	15 kDa selenoprotein	9.52E-03	4.47E-01
METTL13	methyltransferase like 13	9.53E-03	-4.51E-01

HEATR6	HEAT repeat containing 6	9.53E-03	-5.19E-01
MAFG	MAF bZIP transcription factor G	9.53E-03	-3.99E-01
DAP3	death associated protein 3	9.55E-03	1.91E-01
ACHE	acetylcholinesterase (Cartwright blood group)	9.56E-03	-4.71E-01
TMEM253	transmembrane protein 253	9.56E-03	-3.96E-01
ANKRD13D	ankyrin repeat domain 13D	9.57E-03	-2.06E-01
PGBD3	piggyBac transposable element derived 3	9.59E-03	3.08E-01
FAM46B	family with sequence similarity 46 member B	9.60E-03	2.54E-01
RASA1	RAS p21 protein activator 1	9.62E-03	-3.66E-01
ST6GAL1	ST6 beta-galactoside alpha-2,6-sialyltransferase 1	9.63E-03	-5.78E-01
PRR35	proline rich 35	9.63E-03	-2.39E-01
CATSPERG	cation channel sperm associated auxiliary subunit gamma	9.64E-03	-2.48E-01
DCUN1D1	defective in cullin neddylation 1 domain containing 1	9.66E-03	6.86E-01
PIGS	phosphatidylinositol glycan anchor biosynthesis class S	9.66E-03	-3.18E-01

SYPL2	synaptophysin like 2	9.66E-03	-2.91E-01
SACM1L	SAC1 suppressor of actin mutations 1-like (yeast)	9.67E-03	5.69E-01
BIN3	bridging integrator 3	9.68E-03	2.72E-01
UBE3C	ubiquitin protein ligase E3C	9.68E-03	6.17E-01
BBS7	Bardet-Biedl syndrome 7	9.68E-03	4.78E-01
FAM198B	family with sequence similarity 198 member B	9.68E-03	-4.65E-01
TBC1D8B	TBC1 domain family member 8B	9.68E-03	2.64E-01
ARMCX6	armadillo repeat containing, X-linked 6	9.69E-03	2.47E-01
MED7	mediator complex subunit 7	9.69E-03	5.46E-01
OGFOD3	2-oxoglutarate and iron dependent oxygenase domain containing 3	9.69E-03	-2.86E-01
MRPS5	mitochondrial ribosomal protein S5	9.69E-03	2.51E-01
FBXW4	F-box and WD repeat domain containing 4	9.70E-03	-3.71E-01
SMPDL3B	sphingomyelin phosphodiesterase acid like 3B	9.70E-03	-4.48E-01
TCEA1	transcription elongation factor A1	9.73E-03	7.18E-01
RNVU1-18	RNA, variant U1 small nuclear 18	9.74E-03	-1.12
LRRFIP2	LRR binding FLII interacting protein 2	9.74E-03	2.90E-01

SEPN1	selenoprotein N, 1	9.75E-03	-6.47E-01
TRIM23	tripartite motif containing 23	9.76E-03	-5.53E-01
AASDHPPT	aminoadipate-semialdehyde dehydrogenase-phosphopantetheinyl transferase	9.77E-03	4.22E-01
MRM3	mitochondrial rRNA methyltransferase 3	9.78E-03	6.07E-01
FAM92A1	family with sequence similarity 92 member A1	9.78E-03	5.46E-01
MRPL2	mitochondrial ribosomal protein L2	9.79E-03	4.83E-01
ARHGAP11B	Rho GTPase activating protein 11B	9.79E-03	1.98E-01
PPP3R1	protein phosphatase 3 regulatory subunit B, alpha	9.79E-03	3.33E-01
RNF149	ring finger protein 149	9.81E-03	2.61E-01
TMEM214	transmembrane protein 214	9.81E-03	-4.37E-01
MON2	MON2 homolog, regulator of endosome-to-Golgi trafficking	9.81E-03	-9.78E-01
SRP72	signal recognition particle 72	9.82E-03	3.10E-01
POT1	protection of telomeres 1	9.82E-03	3.30E-01
ANKZF1	ankyrin repeat and zinc finger domain containing 1	9.83E-03	-2.05E-01
LAMP2	lysosomal associated membrane protein 2	9.83E-03	-3.76E-01

SAP30BP	SAP30 binding protein	9.84E-03	-3.12E-01
RABL3	RAB, member of RAS oncogene family like 3	9.84E-03	4.98E-01
NR2C1	nuclear receptor subfamily 2 group C member 1	9.85E-03	2.40E-01
WASH3P	WAS protein family homolog 3 pseudogene	9.87E-03	-2.93E-01
TRIM4	tripartite motif containing 4	9.87E-03	-2.78E-01
RPP25L	ribonuclease P/MRP subunit p25 like	9.88E-03	-4.67E-01
ACSM3	acyl-CoA synthetase medium-chain family member 3	9.88E-03	-1.94E-01
EDC4	enhancer of mRNA decapping 4	9.88E-03	5.83E-01
PRDM8	PR/SET domain 8	9.88E-03	2.51E-01
COG5	component of oligomeric golgi complex 5	9.90E-03	4.12E-01
RPS4X	ribosomal protein S4, X-linked	9.90E-03	-4.92E-01
VEGFB	vascular endothelial growth factor B	9.90E-03	-3.50E-01
NUBP2	nucleotide binding protein 2	9.91E-03	-4.77E-01
IGDCC4	immunoglobulin superfamily DCC subclass member 4	9.93E-03	-5.35E-01

PTPRF	protein tyrosine phosphatase, receptor type F	9.96E-03	-5.82E-01
UROD	uroporphyrinogen decarboxylase	9.96E-03	3.71E-01
GPBP1	GC-rich promoter binding protein 1	9.97E-03	9.75E-01
SPNS1	sphingolipid transporter 1 (putative)	9.97E-03	-4.95E-01
C1QBP	complement C1q binding protein	9.98E-03	3.20E-01
PARP14	poly(ADP-ribose) polymerase family member 14	9.99E-03	5.04E-01
UNC119	unc-119 lipid binding chaperone	1.00E-02	-2.24E-01
MED23	mediator complex subunit 23	1.00E-02	-5.79E-01
ABCB9	ATP binding cassette subfamily B member 9	1.00E-02	-4.18E-01
COX5A	cytochrome c oxidase subunit 5A	1.00E-02	2.26E-01
ETV3	ETS variant 3	1.01E-02	-1.82E-01
CHKB	choline kinase beta	1.01E-02	-1.93E-01
ZFHX3	zinc finger homeobox 3	1.01E-02	-5.59E-01
TRMT10B	tRNA methyltransferase 10B	1.01E-02	-2.31E-01

VEZT	vezatin, adherens junctions transmembrane protein	1.01E-02	5.04E-01
DDAH2	dimethylarginine dimethylaminohydrolase 2	1.01E-02	-6.96E-01
EXOC6	exocyst complex component 6	1.01E-02	2.66E-01
LAMP2	lysosomal associated membrane protein 2	1.01E-02	-3.05E-01
CSNK1D	casein kinase 1 delta	1.01E-02	-4.13E-01
KLHDC3	kelch domain containing 3	1.01E-02	-4.39E-01
PINX1	PIN2/TERF1 interacting, telomerase inhibitor 1	1.01E-02	4.88E-01
MIR488	microRNA 488	1.01E-02	-5.22E-01
A2ML1	alpha-2-macroglobulin like 1	1.01E-02	-2.00E-01
GALNT9	polypeptide N-acetylgalactosaminyltransferase 9	1.01E-02	-2.52E-01
CD58	CD58 molecule	1.02E-02	4.19E-01
MSH2	mutS homolog 2	1.02E-02	9.82E-01
BHLHE40	basic helix-loop-helix family member e40	1.02E-02	3.94E-01
SH3GL1	SH3 domain containing GRB2 like 1, endophilin A2	1.02E-02	-6.05E-01

GAS7	growth arrest specific 7	1.02E-02	-3.62E-01
PPP1R16A	protein phosphatase 1 regulatory subunit 16A	1.02E-02	4.33E-01
GTPBP8	GTP binding protein 8 (putative)	1.02E-02	5.08E-01
CPD	carboxypeptidase D	1.02E-02	3.55E-01
SLC11A2	solute carrier family 11 member 2	1.02E-02	-6.48E-01
SLK	STE20 like kinase	1.02E-02	8.07E-01
MPI	mannose phosphate isomerase	1.02E-02	-3.85E-01
LOC100130452	uncharacterized LOC100130452	1.02E-02	-3.22E-01
ANTXR1	anthrax toxin receptor 1	1.02E-02	-2.38E-01
COQ7	coenzyme Q7, hydroxylase	1.02E-02	-2.20E-01
DUSP15	dual specificity phosphatase 15	1.02E-02	-8.98E-01
SCOC	short coiled-coil protein	1.02E-02	7.10E-01
LMO4	LIM domain only 4	1.02E-02	2.89E-01
SHROOM3	shroom family member 3	1.02E-02	-3.29E-01

MEIS3	Meis homeobox 3	1.02E-02	-2.79E-01
SART1	squamous cell carcinoma antigen recognized by T-cells 1	1.02E-02	2.37E-01
C9orf24	chromosome 9 open reading frame 24	1.02E-02	3.31E-01
PLEKHA4	pleckstrin homology domain containing A4	1.03E-02	-3.06E-01
C1QTNF9B-AS1	C1QTNF9B antisense RNA 1	1.03E-02	2.08E-01
AK2	adenylate kinase 2	1.03E-02	2.87E-01
FLJ42627	uncharacterized LOC645644	1.03E-02	-3.48E-01
NPDC1	neural proliferation, differentiation and control 1	1.03E-02	-2.92E-01
SNX16	sorting nexin 16	1.03E-02	5.94E-01
KLHL5	kelch like family member 5	1.03E-02	-4.67E-01
KLC4	kinesin light chain 4	1.03E-02	3.33E-01
TMEM138	transmembrane protein 138	1.04E-02	5.48E-01
PPP4R4	protein phosphatase 4 regulatory subunit 4	1.04E-02	2.18E-01
TRIM13	tripartite motif containing 13	1.04E-02	-6.05E-01
USP46	ubiquitin specific peptidase 46	1.04E-02	4.53E-01

BNIP3L	BCL2 interacting protein 3 like	1.04E-02	-6.98E-01
TAF3	TATA-box binding protein associated factor 3	1.04E-02	-2.30E-01
DHX29	DEAH-box helicase 29	1.04E-02	7.98E-01
MTMR6	myotubularin related protein 6	1.04E-02	4.82E-01
SLC50A1	solute carrier family 50 member 1	1.04E-02	-5.08E-01
PNRC2	proline rich nuclear receptor coactivator 2	1.04E-02	-4.19E-01
ADGRG1	adhesion G protein-coupled receptor G1	1.04E-02	-3.00E-01
CDK19	cyclin dependent kinase 19	1.04E-02	-5.32E-01
AK5	adenylate kinase 5	1.04E-02	2.01E-01
PHYH	phytanoyl-CoA 2-hydroxylase	1.04E-02	-2.42E-01
PGAP2	post-GPI attachment to proteins 2	1.04E-02	-2.02E-01
ANKRD28	ankyrin repeat domain 28	1.04E-02	-4.02E-01
NSUN5	NOP2/Sun RNA methyltransferase family member 5	1.05E-02	2.82E-01
C16orf58	chromosome 16 open reading frame 58	1.05E-02	-6.56E-01

AGTPBP1	ATP/GTP binding protein 1	1.05E-02	1
SUMO1P3	SUMO1 pseudogene 3	1.05E-02	2.88E-01
NFATC3	nuclear factor of activated T-cells 3	1.05E-02	2.46E-01
KCNG3	potassium voltage-gated channel modifier subfamily G member 3	1.05E-02	2.95E-01
RELN	reelin	1.05E-02	-2.30E-01
WDR34	WD repeat domain 34	1.05E-02	6.08E-01
RPS23	ribosomal protein S23	1.05E-02	3.16E-01
SYF2	SYF2 pre-mRNA splicing factor	1.05E-02	-4.61E-01
LYPLA1	lysophospholipase I	1.05E-02	6.99E-01
STEAP3	STEAP3 metalloreductase	1.05E-02	3.40E-01
PDE8A	phosphodiesterase 8A	1.05E-02	2.21E-01
MSL3	male-specific lethal 3 homolog (Drosophila)	1.05E-02	7.17E-01
PLEKHG4B	pleckstrin homology and RhoGEF domain containing G4B	1.05E-02	-2.59E-01
CBFB	core-binding factor beta subunit	1.06E-02	7.84E-01
TMEM97	transmembrane protein 97	1.06E-02	-7.15E-01
MEGF9	multiple EGF like domains 9	1.06E-02	3.75E-01

LRP10	LDL receptor related protein 10	1.06E-02	-5.78E-01
IQCE	IQ motif containing E	1.06E-02	2.15E-01
SOCS2	suppressor of cytokine signaling 2	1.06E-02	2.49E-01
HACL1	2-hydroxyacyl-CoA lyase 1	1.06E-02	4.35E-01
RNF149	ring finger protein 149	1.06E-02	2.64E-01
SSTR2	somatostatin receptor 2	1.06E-02	-5.09E-01
CDC42SE2	CDC42 small effector 2	1.06E-02	6.19E-01
PGK1	phosphoglycerate kinase 1	1.06E-02	-5.22E-01
SHOC2	SHOC2, leucine rich repeat scaffold protein	1.06E-02	9.18E-01
SERHL2	serine hydrolase-like 2	1.06E-02	-3.47E-01
C12orf66	chromosome 12 open reading frame 66	1.06E-02	2.28E-01
TMBIM1	transmembrane BAX inhibitor motif containing 1	1.06E-02	-4.63E-01
ARHGEF12	Rho guanine nucleotide exchange factor 12	1.06E-02	-2.60E-01
RAP2C	RAP2C, member of RAS oncogene family	1.06E-02	5.15E-01
ADAM8	ADAM metallopeptidase domain 8	1.06E-02	-4.15E-01

B4GAT1	beta-1,4-glucuronyltransferase 1	1.06E-02	-9.35E-01
PPP1R9A	protein phosphatase 1 regulatory subunit 9A	1.06E-02	8.60E-01
RBM4B	RNA binding motif protein 4B	1.07E-02	-6.26E-01
SLC35E1	solute carrier family 35 member E1	1.07E-02	-4.25E-01
MRPL42	mitochondrial ribosomal protein L42	1.07E-02	3.43E-01
HKR1	HKR1, GLI-Kruppel zinc finger family member	1.07E-02	2.61E-01
DLK1	delta like non-canonical Notch ligand 1	1.07E-02	-2.69E-01
ACOT7	acyl-CoA thioesterase 7	1.07E-02	4.74E-01
CCNG1	cyclin G1	1.07E-02	-8.82E-01
RNU4-2	RNA, U4 small nuclear 2	1.07E-02	-1.11
IP6K2	inositol hexakisphosphate kinase 2	1.07E-02	-6.94E-01
PSMC6	proteasome 26S subunit, ATPase 6	1.07E-02	5.83E-01
ATG10	autophagy related 10	1.08E-02	-4.18E-01
DNAJC24	DnaJ heat shock protein family (Hsp40) member C24	1.08E-02	6.40E-01
MYH9	myosin, heavy chain 9, non-muscle	1.08E-02	1

CNNM2	cyclin and CBS domain divalent metal cation transport mediator 2	1.08E-02	-2.22E-01
PRDM11	PR/SET domain 11	1.08E-02	3.04E-01
ACOT9	acyl-CoA thioesterase 9	1.08E-02	4.76E-01
SLC30A5	solute carrier family 30 member 5	1.08E-02	6.10E-01
RNF114	ring finger protein 114	1.08E-02	-2.54E-01
CCBE1	collagen and calcium binding EGF domains 1	1.08E-02	-4.98E-01
DNAH1	dynein axonemal heavy chain 1	1.08E-02	-2.65E-01
LDHD	lactate dehydrogenase D	1.08E-02	-3.69E-01
FOXN3-AS1	FOXN3 antisense RNA 1	1.08E-02	2.06E-01
FAM129B	family with sequence similarity 129 member B	1.08E-02	-2.87E-01
SCRN1	secernin 1	1.08E-02	-6.77E-01
ARHGAP10	Rho GTPase activating protein 10	1.08E-02	3.47E-01
DCTD	dCMP deaminase	1.08E-02	-4.59E-01
WDR26	WD repeat domain 26	1.08E-02	-8.40E-01

EFCAB1	EF-hand calcium binding domain 1	1.08E-02	-2.57E-01
RAD51C	RAD51 paralog C	1.08E-02	-5.69E-01
SPG20	spastic paraplegia 20 (Troyer syndrome)	1.08E-02	2.30E-01
SFTPD	surfactant protein D	1.09E-02	3.43E-01
ACAT2	acetyl-CoA acetyltransferase 2	1.09E-02	4.08E-01
FAM103A1	family with sequence similarity 103 member A1	1.09E-02	3.91E-01
DNPEP	aspartyl aminopeptidase	1.09E-02	4.00E-01
DUSP2	dual specificity phosphatase 2	1.09E-02	-1.96E-01
SNRPN	small nuclear ribonucleoprotein polypeptide N	1.09E-02	-5.43E-01
GOLPH3L	golgi phosphoprotein 3 like	1.09E-02	5.85E-01
RNF4	ring finger protein 4	1.09E-02	5.21E-01
SELT	selenoprotein T	1.10E-02	4.91E-01
LILRA3	leukocyte immunoglobulin like receptor A3	1.10E-02	1.96E-01
CCDC86	coiled-coil domain containing 86	1.10E-02	4.90E-01
WDR86	WD repeat domain 86	1.10E-02	-2.55E-01
TGIF1	TGFB induced factor homeobox 1	1.10E-02	4.47E-01

PINX1	PIN2/TERF1 interacting, telomerase inhibitor 1	1.10E-02	3.16E-01
ARPIN	actin-related protein 2/3 complex inhibitor	1.10E-02	4.24E-01
HSCB	HscB mitochondrial iron-sulfur cluster cochaperone	1.10E-02	-8.63E-01
MBP	myelin basic protein	1.10E-02	2.51E-01
BDH2	3-hydroxybutyrate dehydrogenase, type 2	1.10E-02	-2.84E-01
ASCC2	activating signal cointegrator 1 complex subunit 2	1.10E-02	-7.67E-01
HNRNPH3	heterogeneous nuclear ribonucleoprotein H3	1.10E-02	5.11E-01
EXPH5	exophilin 5	1.10E-02	2.75E-01
TRAF3IP1	TRAF3 interacting protein 1	1.10E-02	2.95E-01
ARF1	ADP ribosylation factor 1	1.10E-02	-4.91E-01
FBXW2	F-box and WD repeat domain containing 2	1.11E-02	-3.07E-01
ZNF567	zinc finger protein 567	1.11E-02	4.81E-01
ALAS1	5'-aminolevulinate synthase 1	1.11E-02	2.63E-01
HSPA13	heat shock protein family A (Hsp70) member 13	1.11E-02	4.74E-01
PCSK7	proprotein convertase subtilisin/kexin type 7	1.11E-02	-4.29E-01

GSR	glutathione-disulfide reductase	1.11E-02	4.67E-01
MPHOSPH10	M-phase phosphoprotein 10	1.11E-02	4.21E-01
FZD3	frizzled class receptor 3	1.11E-02	3.89E-01
HIST1H4J	histone cluster 1, H4j	1.11E-02	2.22E-01
MPC1	mitochondrial pyruvate carrier 1	1.11E-02	3.95E-01
BOLA2	bolA family member 2	1.11E-02	3.96E-01
ZCCHC10	zinc finger CCHC-type containing 10	1.11E-02	4.88E-01
MAPKBP1	mitogen-activated protein kinase binding protein 1	1.11E-02	-2.43E-01
RAB3GAP1	RAB3 GTPase activating protein catalytic subunit 1	1.11E-02	-3.69E-01
SNRNP27	small nuclear ribonucleoprotein U4/U6.U5 subunit 27	1.11E-02	4.06E-01
TGFBI	transforming growth factor beta induced	1.12E-02	2.04E-01
ATMIN	ATM interactor	1.12E-02	4.99E-01
AIFM1	apoptosis inducing factor, mitochondria associated 1	1.12E-02	-3.79E-01
SHFM1	split hand/foot malformation (ectrodactyly) type 1	1.12E-02	4.84E-01
ZNF431	zinc finger protein 431	1.12E-02	2.57E-01
FOXD4L3	forkhead box D4-like 3	1.12E-02	-2.23E-01

NAT8L	N-acetyltransferase 8 like	1.12E-02	1.97E-01
ELAC1	elaC ribonuclease Z 1	1.12E-02	2.41E-01
ANAPC16	anaphase promoting complex subunit 16	1.12E-02	-4.81E-01
DIDO1	death inducer-obliterator 1	1.12E-02	-4.51E-01
ANKRD44	ankyrin repeat domain 44	1.12E-02	-5.66E-01
MFN1	mitofusin 1	1.12E-02	2.50E-01
PANK1	pantothenate kinase 1	1.12E-02	-2.69E-01
FAM57A	family with sequence similarity 57 member A	1.13E-02	4.53E-01
LY96	lymphocyte antigen 96	1.13E-02	1.85E-01
PSMB9	proteasome subunit beta 9	1.13E-02	2.39E-01
TRIM15	tripartite motif containing 15	1.13E-02	2.00E-01
OR10G8	olfactory receptor family 10 subfamily G member 8	1.13E-02	-2.05E-01
NME1-NME2	NME1-NME2 readthrough	1.13E-02	-8.40E-01
RASSF5	Ras association domain family member 5	1.13E-02	-2.24E-01
DNMT3B	DNA methyltransferase 3 beta	1.13E-02	3.22E-01

CCDC57	coiled-coil domain containing 57	1.13E-02	-3.19E-01
REEP1	receptor accessory protein 1	1.13E-02	2.15E-01
SPARC	secreted protein acidic and cysteine rich	1.13E-02	-3.40E-01
KISS1R	KISS1 receptor	1.14E-02	5.17E-01
ALDOA	aldolase, fructose-bisphosphate A	1.14E-02	3.18E-01
ARHGEF40	Rho guanine nucleotide exchange factor 40	1.14E-02	-2.10E-01
PPM1B	protein phosphatase, Mg2+/Mn2+ dependent 1B	1.14E-02	-3.62E-01
THOC5	THO complex 5	1.14E-02	2.81E-01
NLK	nemo like kinase	1.14E-02	2.45E-01
SMNDC1	survival motor neuron domain containing 1	1.14E-02	3.88E-01
GNB4	G protein subunit beta 4	1.14E-02	-6.07E-01
C1orf101	chromosome 1 open reading frame 101	1.14E-02	-2.51E-01
BCAS4	breast carcinoma amplified sequence 4	1.14E-02	-7.54E-01
SPRTN	SprT-like N-terminal domain	1.14E-02	-4.26E-01
GPATCH2	G-patch domain containing 2	1.14E-02	3.47E-01

NXT1	nuclear transport factor 2 like export factor 1	1.14E-02	6.12E-01
CCND2	cyclin D2	1.14E-02	-6.69E-01
STX2	syntaxin 2	1.14E-02	2.74E-01
COASY	Coenzyme A synthase	1.14E-02	-3.33E-01
SULT1A3	sulfotransferase family 1A member 3	1.14E-02	-2.93E-01
DDHD1	DDHD domain containing 1	1.14E-02	4.76E-01
KDELR3	KDEL endoplasmic reticulum protein retention receptor 3	1.14E-02	-4.15E-01
MIR1914	microRNA 1914	1.15E-02	-2.57E-01
PSME1	proteasome activator subunit 1	1.15E-02	3.63E-01
MAGOH	mago homolog, exon junction complex core component	1.15E-02	7.75E-01
CLTA	clathrin light chain A	1.15E-02	-3.84E-01
CD58	CD58 molecule	1.15E-02	4.31E-01
TM4SF4	transmembrane 4 L six family member 4	1.15E-02	2.06E-01
ATXN1L	ataxin 1 like	1.15E-02	3.26E-01
EXOSC10	exosome component 10	1.15E-02	5.10E-01

KRTAP3-1	keratin associated protein 3-1	1.15E-02	2.22E<br>-01
EYA3	EYA transcriptional coactivator and phosphatase 3	1.15E-02	2.77E<br>-01
IL15RA	interleukin 15 receptor subunit alpha	1.15E-02	2.09E<br>-01
BAG2	BCL2 associated athanogene 2	1.15E-02	3.16E<br>-01
OSCP1	organic solute carrier partner 1	1.15E-02	4.23E<br>-01
MUTYH	mutY DNA glycosylase	1.15E-02	5.15E<br>-01
GLT8D1	glycosyltransferase 8 domain containing 1	1.15E-02	-<br>2.59E<br>-01
C11orf24	chromosome 11 open reading frame 24	1.15E-02	-<br>4.75E<br>-01
SFN	stratifin	1.15E-02	-<br>2.48E<br>-01
EBF4	early B-cell factor 4	1.15E-02	-<br>1.70E<br>-01
FIBP	FGF1 intracellular binding protein	1.16E-02	6.22E<br>-01
GTSE1	G2 and S-phase expressed 1	1.16E-02	3.53E<br>-01
PTPN2	protein tyrosine phosphatase, non-receptor type 2	1.16E-02	3.12E<br>-01
ZNF205	zinc finger protein 205	1.16E-02	-<br>1.93E<br>-01
SLC17A7	solute carrier family 17 member 7	1.16E-02	2.67E<br>-01
ANTXR2	anthrax toxin receptor 2	1.16E-02	2.06E<br>-01

ERGIC3	ERGIC and golgi 3	1.16E-02	-4.44E-01
LMOD3	leiomodin 3	1.16E-02	-6.66E-01
SMPDL3B	sphingomyelin phosphodiesterase acid like 3B	1.16E-02	-2.74E-01
MRPL17	mitochondrial ribosomal protein L17	1.16E-02	8.02E-01
PPM1K	protein phosphatase, Mg2+/Mn2+ dependent 1K	1.16E-02	-5.21E-01
MMGT1	membrane magnesium transporter 1	1.16E-02	5.80E-01
PNPLA8	patatin like phospholipase domain containing 8	1.17E-02	6.43E-01
KIAA1468	KIAA1468	1.17E-02	7.55E-01
TRPS1	transcriptional repressor GATA binding 1	1.17E-02	-4.29E-01
DCLK2	doublecortin like kinase 2	1.17E-02	2.69E-01
HNRNPR	heterogeneous nuclear ribonucleoprotein R	1.17E-02	4.12E-01
GM2A	GM2 ganglioside activator	1.17E-02	-2.74E-01
ERCC1	ERCC excision repair 1, endonuclease non-catalytic subunit	1.17E-02	-5.95E-01
Mar-06	membrane associated ring-CH-type finger 6	1.17E-02	-8.96E-01
ZNF283	zinc finger protein 283	1.17E-02	3.93E-01

RPL12	ribosomal protein L12	1.18E-02	-5.29E-01
ITGA5	integrin subunit alpha 5	1.18E-02	-5.51E-01
CUTA	cutA divalent cation tolerance homolog	1.18E-02	-5.54E-01
C7orf43	chromosome 7 open reading frame 43	1.18E-02	-2.41E-01
ALDH6A1	aldehyde dehydrogenase 6 family member A1	1.18E-02	-3.24E-01
SH2D3C	SH2 domain containing 3C	1.18E-02	-2.68E-01
PDX1	pancreatic and duodenal homeobox 1	1.18E-02	1.95E-01
MERTK	MER proto-oncogene, tyrosine kinase	1.18E-02	-5.94E-01
CCDC26	CCDC26 long non-coding RNA	1.18E-02	1.78E-01
CEP85	centrosomal protein 85	1.18E-02	5.49E-01
TTC13	tetratricopeptide repeat domain 13	1.18E-02	4.09E-01
DYNLL1	dynein light chain LC8-type 1	1.19E-02	3.91E-01
HMGXB3	HMG-box containing 3	1.19E-02	-3.01E-01
MTF1	metal regulatory transcription factor 1	1.19E-02	3.02E-01

PRR14	proline rich 14	1.19E-02	-5.20E-01
TADA2B	transcriptional adaptor 2B	1.19E-02	-5.30E-01
ENC1	ectodermal-neural cortex 1	1.19E-02	2.18E-01
PCDHAC1	protocadherin alpha subfamily C, 1	1.19E-02	2.17E-01
ZNF337	zinc finger protein 337	1.19E-02	-4.57E-01
CATSPER2	cation channel sperm associated 2	1.19E-02	-2.49E-01
SEMA4B	semaphorin 4B	1.19E-02	-6.42E-01
TERF2	telomeric repeat binding factor 2	1.19E-02	-3.71E-01
RNU1-3	RNA, U1 small nuclear 3	1.19E-02	-1.06
PPFIBP2	PPFIA binding protein 2	1.19E-02	2.21E-01
TMEM131	transmembrane protein 131	1.19E-02	-5.93E-01
KIF23	kinesin family member 23	1.19E-02	2.48E-01
NME1	NME/NM23 nucleoside diphosphate kinase 1	1.19E-02	-7.81E-01
RNF123	ring finger protein 123	1.19E-02	-4.45E-01

TMCO1	transmembrane and coiled-coil domains 1	1.19E-02	-2.83E-01
TMEM243	transmembrane protein 243	1.19E-02	-2.59E-01
TSC22D4	TSC22 domain family member 4	1.19E-02	-3.63E-01
CHPT1	choline phosphotransferase 1	1.19E-02	-2.17E-01
MAPK15	mitogen-activated protein kinase 15	1.20E-02	-2.67E-01
PPP2R3B	protein phosphatase 2 regulatory subunit B"beta	1.20E-02	-4.06E-01
OXTR	oxytocin receptor	1.20E-02	3.83E-01
ARID2	AT-rich interaction domain 2	1.20E-02	-3.85E-01
KLF5	Kruppel like factor 5	1.20E-02	3.20E-01
NOP2	NOP2 nucleolar protein	1.20E-02	5.24E-01
CDV3	CDV3 homolog	1.20E-02	6.04E-01
IWS1	IWS1, SUPT6H interacting protein	1.20E-02	6.32E-01
EPS15L1	epidermal growth factor receptor pathway substrate 15 like 1	1.20E-02	4.36E-01
GOLGA6L6	golgin A6 family-like 6	1.20E-02	-5.52E-01
PTPN9	protein tyrosine phosphatase, non-receptor type 9	1.20E-02	3.35E-01

TRMT1L	tRNA methyltransferase 1 like	1.20E-02	4.48E-01
CSNK1G1	casein kinase 1 gamma 1	1.20E-02	-6.11E-01
SEPHS2	selenophosphate synthetase 2	1.20E-02	-5.61E-01
ARFGAP1	ADP ribosylation factor GTPase activating protein 1	1.20E-02	-2.24E-01
DDRGK1	DDRGK domain containing 1	1.20E-02	5.36E-01
CDC40	cell division cycle 40	1.20E-02	-4.46E-01
ZNF526	zinc finger protein 526	1.21E-02	-5.24E-01
EMP1	epithelial membrane protein 1	1.21E-02	4.52E-01
ZDHHC20	zinc finger DHHC-type containing 20	1.21E-02	3.66E-01
G2E3	G2/M-phase specific E3 ubiquitin protein ligase	1.21E-02	3.29E-01
SMIM7	small integral membrane protein 7	1.21E-02	4.98E-01
MEGF6	multiple EGF like domains 6	1.21E-02	-7.20E-01
HSPA4	heat shock protein family A (Hsp70) member 4	1.21E-02	4.96E-01
GGA1	golgi associated, gamma adaptin ear containing, ARF binding protein 1	1.21E-02	-2.25E-01
EEF1E1	eukaryotic translation elongation factor 1 epsilon 1	1.21E-02	2.13E-01

PANX1	pannexin 1	1.21E-02	5.20E-01
NAAA	N-acylethanolamine acid amidase	1.21E-02	3.68E-01
EMID1	EMI domain containing 1	1.21E-02	-4.31E-01
HNRNPM	heterogeneous nuclear ribonucleoprotein M	1.21E-02	5.95E-01
MACF1	microtubule-actin crosslinking factor 1	1.21E-02	-6.51E-01
GGT7	gamma-glutamyltransferase 7	1.21E-02	-2.52E-01
ENDOD1	endonuclease domain containing 1	1.22E-02	4.53E-01
CACNA1C	calcium voltage-gated channel subunit alpha1 C	1.22E-02	-2.26E-01
CSTF3	cleavage stimulation factor subunit 3	1.22E-02	-5.90E-01
FAM122A	family with sequence similarity 122A	1.22E-02	-1.94E-01
LIMS1	LIM zinc finger domain containing 1	1.22E-02	7.32E-01
RABIF	RAB interacting factor	1.22E-02	2.10E-01
PEX5	peroxisomal biogenesis factor 5	1.22E-02	-3.47E-01
MAN1A2	mannosidase alpha class 1A member 2	1.22E-02	3.81E-01
TBCA	tubulin folding cofactor A	1.22E-02	2.65E-01

SNX11	sorting nexin 11	1.22E-02	4.65E-01
PCYT1B	phosphate cytidylyltransferase 1, choline, beta	1.22E-02	2.45E-01
SDHAP1	succinate dehydrogenase complex flavoprotein subunit A pseudogene 1	1.22E-02	-4.89E-01
SLC39A4	solute carrier family 39 member 4	1.22E-02	6.65E-01
MICALL1	MICAL like 1	1.23E-02	-5.81E-01
TMEM51	transmembrane protein 51	1.23E-02	3.57E-01
TMEM222	transmembrane protein 222	1.23E-02	-2.32E-01
RRNAD1	ribosomal RNA adenine dimethylase domain containing 1	1.23E-02	-2.95E-01
MIR604	microRNA 604	1.23E-02	-1.98E-01
TFRC	transferrin receptor	1.23E-02	4.55E-01
GIPC1	GIPC PDZ domain containing family member 1	1.23E-02	-4.77E-01
LGMN	legumain	1.23E-02	-4.15E-01
UBA6	ubiquitin like modifier activating enzyme 6	1.23E-02	4.28E-01
RCCD1	RCC1 domain containing 1	1.24E-02	-2.46E-01

ZCCHC14	zinc finger CCHC-type containing 14	1.24E-02	-6.40E-01
ANKRD49	ankyrin repeat domain 49	1.24E-02	4.80E-01
PLGLB1	plasminogen-like B1	1.24E-02	-2.09E-01
TRPV2	transient receptor potential cation channel subfamily V member 2	1.24E-02	-2.12E-01
TEX9	testis expressed 9	1.24E-02	2.81E-01
CREB3L2	cAMP responsive element binding protein 3 like 2	1.24E-02	-6.42E-01
LRRK1	leucine rich repeat kinase 1	1.24E-02	1.98E-01
SPCS3	signal peptidase complex subunit 3	1.24E-02	5.92E-01
FAM171A1	family with sequence similarity 171 member A1	1.24E-02	-3.87E-01
DNAJC12	DnaJ heat shock protein family (Hsp40) member C12	1.24E-02	-4.41E-01
IVD	isovaleryl-CoA dehydrogenase	1.25E-02	-5.51E-01
SEC24B	SEC24 homolog B, COPII coat complex component	1.25E-02	-1.72E-01
CAMK2N1	calcium/calmodulin dependent protein kinase II inhibitor 1	1.25E-02	2.83E-01
G3BP2	G3BP stress granule assembly factor 2	1.25E-02	6.13E-01
HMGB3	high mobility group box 3	1.25E-02	3.01E-01

NECTIN3	nectin cell adhesion molecule 3	1.25E-02	-3.44E-01
SAXO2	stabilizer of axonemal microtubules 2	1.25E-02	2.03E-01
NDUFB8	NADH:ubiquinone oxidoreductase subunit B8	1.25E-02	-2.57E-01
KIAA1143	KIAA1143	1.25E-02	6.17E-01
POR	cytochrome p450 oxidoreductase	1.25E-02	-4.06E-01
FAM206A	family with sequence similarity 206 member A	1.25E-02	2.54E-01
SLITRK6	SLIT and NTRK like family member 6	1.25E-02	2.71E-01
PDSS2	prenyl (decaprenyl) diphosphate synthase, subunit 2	1.26E-02	-2.74E-01
ZNF112	zinc finger protein 112	1.26E-02	3.43E-01
PTER	phosphotriesterase related	1.26E-02	2.77E-01
RBMY3AP	RNA binding motif protein, Y-linked, family 3, member A pseudogene	1.26E-02	3.15E-01
GABRE	gamma-aminobutyric acid type A receptor epsilon subunit	1.26E-02	2.57E-01
ANGPTL3	angiopoietin like 3	1.26E-02	2.17E-01
FBXO30	F-box protein 30	1.26E-02	4.07E-01
KANK2	KN motif and ankyrin repeat domains 2	1.26E-02	-2.87E-01

RABGAP1L	RAB GTPase activating protein 1 like	1.26E-02	-5.19E-01
MYO7A	myosin VIIA	1.26E-02	-2.79E-01
WDR37	WD repeat domain 37	1.26E-02	-3.85E-01
AASS	aminoadipate-semialdehyde synthase	1.26E-02	-2.52E-01
KDM4C	lysine demethylase 4C	1.27E-02	-3.69E-01
IFNLR1	interferon lambda receptor 1	1.27E-02	3.75E-01
NFYC-AS1	NFYC antisense RNA 1	1.27E-02	-3.54E-01
HIC1	hypermethylated in cancer 1	1.27E-02	1.99E-01
NDRG1	N-myc downstream regulated 1	1.27E-02	2.90E-01
MED22	mediator complex subunit 22	1.27E-02	4.02E-01
MRPS22	mitochondrial ribosomal protein S22	1.27E-02	3.58E-01
TAF7L	TATA-box binding protein associated factor 7 like	1.28E-02	1.78E-01
NAPRT	nicotinate phosphoribosyltransferase	1.28E-02	3.12E-01
DHX15	DEAH-box helicase 15	1.28E-02	4.39E-01
DHRS3	dehydrogenase/reductase 3	1.28E-02	3.34E-01

SMPD4	sphingomyelin phosphodiesterase 4	1.28E-02	1.80E-01
CASP8	caspase 8	1.28E-02	2.70E-01
ATF6B	activating transcription factor 6 beta	1.28E-02	-3.07E-01
ARF1	ADP ribosylation factor 1	1.28E-02	-3.16E-01
ZNF92	zinc finger protein 92	1.28E-02	4.31E-01
EID3	EP300 interacting inhibitor of differentiation 3	1.29E-02	2.46E-01
ARHGEF39	Rho guanine nucleotide exchange factor 39	1.29E-02	2.45E-01
DBX1	developing brain homeobox 1	1.29E-02	2.36E-01
DBN1	drebrin 1	1.29E-02	2.91E-01
OSCAR	osteoclast associated, immunoglobulin-like receptor	1.29E-02	-1.96E-01
TMEM256	transmembrane protein 256	1.29E-02	-6.84E-01
MAGEC2	MAGE family member C2	1.29E-02	-2.44E-01
ZADH2	zinc binding alcohol dehydrogenase domain containing 2	1.29E-02	-3.86E-01
GGCT	gamma-glutamylcyclotransferase	1.29E-02	3.98E-01
LRRFIP2	LRR binding FLII interacting protein 2	1.29E-02	4.87E-01

TMEM105	transmembrane protein 105	1.29E-02	-1.94E-01
ACVR2A	activin A receptor type 2A	1.29E-02	-2.47E-01
CCDC184	coiled-coil domain containing 184	1.30E-02	-2.94E-01
SPRY1	sprouty RTK signaling antagonist 1	1.30E-02	1.81E-01
MLEC	malectin	1.30E-02	-8.08E-01
PCDH9	protocadherin 9	1.30E-02	4.16E-01
CHST15	carbohydrate (N-acetylgalactosamine 4-sulfate 6-O) sulfotransferase 15	1.31E-02	-3.09E-01
MUM1L1	MUM1 like 1	1.31E-02	-6.65E-01
SYPL1	synaptophysin like 1	1.32E-02	2.14E-01
ASCC3	activating signal cointegrator 1 complex subunit 3	1.32E-02	-1.19
RERE	arginine-glutamic acid dipeptide repeats	1.32E-02	-5.05E-01
ZC3H10	zinc finger CCCH-type containing 10	1.32E-02	2.19E-01
UQCRBP1	ubiquinol-cytochrome c reductase binding protein pseudogene 1	1.32E-02	-3.12E-01
BBS7	Bardet-Biedl syndrome 7	1.32E-02	6.60E-01

TACC1	transforming acidic coiled-coil containing protein 1	1.32E-02	-3.53E-01
GCAT	glycine C-acetyltransferase	1.32E-02	3.84E-01
SLC5A6	solute carrier family 5 member 6	1.32E-02	-6.14E-01
JADE2	jade family PHD finger 2	1.32E-02	-2.51E-01
MAPK1	mitogen-activated protein kinase 1	1.32E-02	4.81E-01
AP1G2	adaptor related protein complex 1 gamma 2 subunit	1.32E-02	-6.18E-01
C1orf74	chromosome 1 open reading frame 74	1.32E-02	5.95E-01
CBLL1	Cbl proto-oncogene like 1	1.32E-02	3.77E-01
HECTD2	HECT domain E3 ubiquitin protein ligase 2	1.32E-02	4.19E-01
FANCD2	Fanconi anemia complementation group D2	1.32E-02	2.40E-01
STRIP2	striatin interacting protein 2	1.32E-02	-6.37E-01
CDK9	cyclin dependent kinase 9	1.32E-02	-3.41E-01
UBE2R2	ubiquitin conjugating enzyme E2 R2	1.32E-02	-4.38E-01
SNORA26	small nucleolar RNA, H/ACA box 26	1.33E-02	-3.62E-01

KIAA1211L	KIAA1211 like	1.33E-02	-2.60E-01
PRPSAP2	phosphoribosyl pyrophosphate synthetase associated protein 2	1.33E-02	-2.85E-01
BAX	BCL2 associated X, apoptosis regulator	1.33E-02	-3.58E-01
MAPK10	mitogen-activated protein kinase 10	1.33E-02	1.85E-01
TCHP	trichoplein keratin filament binding	1.33E-02	2.92E-01
KAT2A	lysine acetyltransferase 2A	1.33E-02	-4.81E-01
B3GALT6	beta-1,3-galactosyltransferase 6	1.33E-02	-5.17E-01
C20orf24	chromosome 20 open reading frame 24	1.33E-02	3.84E-01
GALNT2	polypeptide N-acetylgalactosaminyltransferase 2	1.33E-02	-6.31E-01
PCK2	phosphoenolpyruvate carboxykinase 2, mitochondrial	1.33E-02	-3.81E-01
KLHL18	kelch like family member 18	1.33E-02	5.85E-01
DHCR7	7-dehydrocholesterol reductase	1.33E-02	4.45E-01
FSTL1	follistatin like 1	1.33E-02	-7.24E-01
ERCC8	ERCC excision repair 8, CSA ubiquitin ligase complex subunit	1.34E-02	3.31E-01

LINC00160	long intergenic non-protein coding RNA 160	1.34E-02	-2.61E-01
PHF19	PHD finger protein 19	1.34E-02	4.81E-01
LYSMD2	LysM domain containing 2	1.34E-02	2.45E-01
SRA1	steroid receptor RNA activator 1	1.34E-02	3.41E-01
EIF4A1	eukaryotic translation initiation factor 4A1	1.34E-02	-6.71E-01
NRP2	neuropilin 2	1.34E-02	2.15E-01
ROCK1P1	Rho associated coiled-coil containing protein kinase 1 pseudogene 1	1.34E-02	8.07E-01
ISCA1	iron-sulfur cluster assembly 1	1.34E-02	5.63E-01
PPP1R1B	protein phosphatase 1 regulatory inhibitor subunit 1B	1.34E-02	3.00E-01
AATK	apoptosis associated tyrosine kinase	1.34E-02	-3.16E-01
ASCC3	activating signal cointegrator 1 complex subunit 3	1.35E-02	-4.01E-01
VPS13B	vacuolar protein sorting 13 homolog B	1.35E-02	2.36E-01
NSUN3	NOP2/Sun RNA methyltransferase family member 3	1.35E-02	3.29E-01
HIF1A	hypoxia inducible factor 1 alpha subunit	1.35E-02	1.03
TBCB	tubulin folding cofactor B	1.35E-02	6.75E-01
ACOT8	acyl-CoA thioesterase 8	1.35E-02	-3.44E-01

CLK2P1	CDC like kinase 2, pseudogene 1	1.35E-02	-3.05E-01
MRPL12	mitochondrial ribosomal protein L12	1.35E-02	-4.92E-01
FAM47E	family with sequence similarity 47 member E	1.35E-02	2.30E-01
NIPA1	non imprinted in Prader-Willi/Angelman syndrome 1	1.35E-02	3.98E-01
C19orf54	chromosome 19 open reading frame 54	1.35E-02	-3.29E-01
SIAH1	siah E3 ubiquitin protein ligase 1	1.35E-02	-2.04E-01
SYT15	synaptotagmin 15	1.35E-02	-5.27E-01
OGDH	oxoglutarate dehydrogenase	1.36E-02	1.91E-01
PCAT4	prostate cancer associated transcript 4 (non-protein coding)	1.36E-02	1.97E-01
CRYL1	crystallin lambda 1	1.36E-02	2.75E-01
FBXO28	F-box protein 28	1.36E-02	2.48E-01
ZNF93	zinc finger protein 93	1.36E-02	2.15E-01
JUP	junction plakoglobin	1.36E-02	-3.28E-01
NPIPB3	nuclear pore complex interacting protein family member B3	1.36E-02	-7.53E-01
HEXIM2	hexamethylene bisacetamide inducible 2	1.36E-02	-4.93E-01

APEX1	apurinic/apyrimidinic endodeoxyribonuclease 1	1.36E-02	-3.42E-01
EPB41L1	erythrocyte membrane protein band 4.1 like 1	1.36E-02	-1.99E-01
TSPAN7	tetraspanin 7	1.37E-02	2.70E-01
JAKMIP3	Janus kinase and microtubule interacting protein 3	1.37E-02	2.71E-01
TNFSF4	tumor necrosis factor superfamily member 4	1.37E-02	-2.71E-01
C2orf42	chromosome 2 open reading frame 42	1.37E-02	2.35E-01
TSR3	TSR3, acp transferase ribosome maturation factor	1.37E-02	3.36E-01
IFIH1	interferon induced with helicase C domain 1	1.37E-02	3.57E-01
ZHX2	zinc fingers and homeoboxes 2	1.37E-02	2.11E-01
CAB39L	calcium binding protein 39 like	1.37E-02	-3.04E-01
SIVA1	SIVA1 apoptosis inducing factor	1.37E-02	9.23E-01
PDE8B	phosphodiesterase 8B	1.37E-02	2.24E-01
FAM98C	family with sequence similarity 98 member C	1.38E-02	-4.55E-01
UBE2Z	ubiquitin conjugating enzyme E2 Z	1.38E-02	1.83E-01
KLF4	Kruppel like factor 4	1.38E-02	2.62E-01

MAP3K14-AS1	MAP3K14 antisense RNA 1	1.38E-02	-2.18E-01
DEPDC4	DEP domain containing 4	1.38E-02	2.41E-01
EMC1	ER membrane protein complex subunit 1	1.38E-02	3.05E-01
ERCC8	ERCC excision repair 8, CSA ubiquitin ligase complex subunit	1.38E-02	-1.67E-01
ADPRHL1	ADP-ribosylhydrolase like 1	1.38E-02	-2.37E-01
TEX15	testis expressed 15	1.38E-02	9.89E-01
MZT1	mitotic spindle organizing protein 1	1.39E-02	4.35E-01
TTC19	tetratricopeptide repeat domain 19	1.39E-02	5.93E-01
KLHL20	kelch like family member 20	1.39E-02	-3.44E-01
STK26	serine/threonine protein kinase 26	1.39E-02	3.50E-01
EGFR	epidermal growth factor receptor	1.39E-02	2.03E-01
IFT122	intraflagellar transport 122	1.39E-02	3.10E-01
UBE2I	ubiquitin conjugating enzyme E2 I	1.39E-02	4.35E-01
ALG9	ALG9, alpha-1,2-mannosyltransferase	1.39E-02	-3.27E-01
TERT	telomerase reverse transcriptase	1.39E-02	2.25E-01
SLC25A25	solute carrier family 25 member 25	1.39E-02	4.06E-01

LGALS3BP	galectin 3 binding protein	1.39E-02	2.16E-01
SVBP	small vasohibin binding protein	1.40E-02	-5.36E-01
DLD	dihydrolipoamide dehydrogenase	1.40E-02	4.76E-01
BCAP29	B-cell receptor-associated protein 29	1.40E-02	2.64E-01
CARS2	cysteinyl-tRNA synthetase 2, mitochondrial (putative)	1.40E-02	-2.82E-01
DHRS13	dehydrogenase/reductase 13	1.40E-02	-5.72E-01
DTNB	dystrobrevin beta	1.40E-02	-2.92E-01
STK11	serine/threonine kinase 11	1.40E-02	-2.83E-01
NAAA	N-acylethanolamine acid amidase	1.40E-02	3.87E-01
LRRC20	leucine rich repeat containing 20	1.40E-02	2.17E-01
TEX10	testis expressed 10	1.40E-02	3.15E-01
PPP1R3B	protein phosphatase 1 regulatory subunit 3B	1.40E-02	2.27E-01
CD82	CD82 molecule	1.41E-02	-2.73E-01
NUCKS1	nuclear casein kinase and cyclin dependent kinase substrate 1	1.41E-02	3.88E-01
KLHL42	kelch like family member 42	1.41E-02	-4.28E-01

SP3	Sp3 transcription factor	1.41E-02	5.45E-01
CYP20A1	cytochrome P450 family 20 subfamily A member 1	1.41E-02	2.32E-01
Mar-01	membrane associated ring-CH-type finger 1	1.41E-02	-1.76E-01
SNRPA	small nuclear ribonucleoprotein polypeptide A	1.41E-02	-5.48E-01
SMC3	structural maintenance of chromosomes 3	1.41E-02	1.15
ACAP2	ArfGAP with coiled-coil, ankyrin repeat and PH domains 2	1.41E-02	-2.67E-01
HAGH	hydroxyacylglutathione hydrolase	1.42E-02	-7.58E-01
LINC00202-2	long intergenic non-protein coding RNA 202-2	1.42E-02	-5.38E-01
FYTTD1	forty-two-three domain containing 1	1.42E-02	-6.49E-01
CD9	CD9 molecule	1.42E-02	2.07E-01
SF3B3	splicing factor 3b subunit 3	1.42E-02	-5.33E-01
NPC2	NPC intracellular cholesterol transporter 2	1.42E-02	-3.44E-01
CNPY3	canopy FGF signaling regulator 3	1.42E-02	-3.74E-01
HDGFRP2	hepatoma-derived growth factor-related protein 2	1.42E-02	-4.24E-01

MAP2K7	mitogen-activated protein kinase kinase 7	1.42E-02	-2.43E-01
ACOT9	acyl-CoA thioesterase 9	1.42E-02	2.58E-01
FBXO18	F-box protein, helicase, 18	1.43E-02	-4.87E-01
FAM229B	family with sequence similarity 229 member B	1.43E-02	-4.14E-01
TAF5L	TATA-box binding protein associated factor 5 like	1.43E-02	2.00E-01
PSMB1	proteasome subunit beta 1	1.43E-02	3.70E-01
SPANXD	SPANX family member D	1.43E-02	2.36E-01
ZWINT	ZW10 interacting kinetochore protein	1.43E-02	2.73E-01
NFKBIZ	NFKB inhibitor zeta	1.43E-02	-2.58E-01
HNRNPL	heterogeneous nuclear ribonucleoprotein L	1.43E-02	7.05E-01
GALT	galactose-1-phosphate uridylyltransferase	1.43E-02	-5.00E-01
RPL32	ribosomal protein L32	1.44E-02	-4.07E-01
KRT17	keratin 17	1.44E-02	1.77E-01
CDNF	cerebral dopamine neurotrophic factor	1.44E-02	-1.89E-01
RWDD2A	RWD domain containing 2A	1.44E-02	-3.88E-01

TP53I11	tumor protein p53 inducible protein 11	1.44E-02	-4.06E-01
SCAP	SREBF chaperone	1.44E-02	-7.92E-01
PRLR	prolactin receptor	1.44E-02	2.04E-01
NMT2	N-myristoyltransferase 2	1.44E-02	4.39E-01
ATP5L	ATP synthase, H+ transporting, mitochondrial Fo complex subunit G	1.44E-02	-6.19E-01
H6PD	hexose-6-phosphate dehydrogenase/glucose 1-dehydrogenase	1.44E-02	-3.16E-01
PTK2B	protein tyrosine kinase 2 beta	1.44E-02	-2.27E-01
NCK1	NCK adaptor protein 1	1.44E-02	-3.56E-01
FAM131B	family with sequence similarity 131 member B	1.44E-02	-3.12E-01
TNNC1	troponin C1, slow skeletal and cardiac type	1.45E-02	-6.53E-01
RAD51	RAD51 recombinase	1.45E-02	5.22E-01
NFKBIB	NFKB inhibitor beta	1.45E-02	3.31E-01
C19orf45	chromosome 19 open reading frame 45	1.45E-02	-4.19E-01
ZNF358	zinc finger protein 358	1.45E-02	-7.09E-01

ALDH1A2	aldehyde dehydrogenase 1 family member A2	1.45E-02	2.28E-01
IRF4	interferon regulatory factor 4	1.45E-02	3.21E-01
VEZF1	vascular endothelial zinc finger 1	1.45E-02	-4.39E-01
CMTM3	CKLF like MARVEL transmembrane domain containing 3	1.45E-02	-2.59E-01
RNU11	RNA, U11 small nuclear	1.45E-02	-3.16E-01
UBE2L6	ubiquitin conjugating enzyme E2 L6	1.45E-02	-2.89E-01
GCSH	glycine cleavage system protein H	1.45E-02	4.61E-01
BAG5	BCL2 associated athanogene 5	1.45E-02	3.88E-01
ARRDC1	arrestin domain containing 1	1.45E-02	2.74E-01
MBD3L3	methyl-CpG binding domain protein 3 like 3	1.46E-02	-3.08E-01
AGAP3	ArfGAP with GTPase domain, ankyrin repeat and PH domain 3	1.46E-02	2.82E-01
PRKD2	protein kinase D2	1.46E-02	-2.98E-01
DENND4C	DENN domain containing 4C	1.46E-02	-3.81E-01
SLU7	SLU7 homolog, splicing factor	1.46E-02	4.90E-01
BTBD6	BTB domain containing 6	1.46E-02	2.06E-01

SDHAP1	succinate dehydrogenase complex flavoprotein subunit A pseudogene 1	1.46E-02	-4.48E-01
FBXO45	F-box protein 45	1.46E-02	-3.28E-01
SDC2	syndecan 2	1.46E-02	3.86E-01
TMEM44	transmembrane protein 44	1.47E-02	-7.31E-01
GPR107	G protein-coupled receptor 107	1.47E-02	-1.81E-01
MIR574	microRNA 574	1.47E-02	3.66E-01
PGPEP1L	pyroglutamyl-peptidase I-like	1.47E-02	-4.83E-01
LRCH4	leucine rich repeats and calponin homology domain containing 4	1.47E-02	-3.47E-01
CDKN2C	cyclin dependent kinase inhibitor 2C	1.47E-02	-6.94E-01
SEPN1	selenoprotein N, 1	1.47E-02	-7.43E-01
CCDC151	coiled-coil domain containing 151	1.47E-02	-3.81E-01
FAM181B	family with sequence similarity 181 member B	1.47E-02	-2.52E-01
CIAO1	cytosolic iron-sulfur assembly component 1	1.47E-02	-4.33E-01

PATZ1	POZ/BTB and AT hook containing zinc finger 1	1.47E-02	1.72E-01
NOP14-AS1	NOP14 antisense RNA 1	1.47E-02	-3.32E-01
MIS12	MIS12, kinetochore complex component	1.47E-02	2.73E-01
WBSCR22	Williams-Beuren syndrome chromosome region 22	1.48E-02	3.99E-01
ERI2	ERI1 exoribonuclease family member 2	1.48E-02	3.60E-01
TMEM70	transmembrane protein 70	1.48E-02	3.03E-01
ZNF232	zinc finger protein 232	1.48E-02	3.09E-01
LAS1L	LAS1 like, ribosome biogenesis factor	1.48E-02	4.57E-01
PINK1	PTEN induced putative kinase 1	1.48E-02	-4.52E-01
TNFSF15	tumor necrosis factor superfamily member 15	1.48E-02	-5.69E-01
NSUN2	NOP2/Sun RNA methyltransferase family member 2	1.48E-02	4.84E-01
PHYH	phytanoyl-CoA 2-hydroxylase	1.48E-02	-4.08E-01
MIR657	microRNA 657	1.48E-02	1.64E-01
PSMD10	proteasome 26S subunit, non-ATPase 10	1.48E-02	4.07E-01
TMEM164	transmembrane protein 164	1.48E-02	-4.79E-01
DHRSX	dehydrogenase/reductase X-linked	1.49E-02	2.88E-01

EIF2B2	eukaryotic translation initiation factor 2B subunit beta	1.49E-02	-1.91E-01
STAC2	SH3 and cysteine rich domain 2	1.49E-02	-2.29E-01
ZNF268	zinc finger protein 268	1.49E-02	4.51E-01
MRPS31	mitochondrial ribosomal protein S31	1.49E-02	4.57E-01
CSTF3	cleavage stimulation factor subunit 3	1.49E-02	-6.11E-01
FAM120C	family with sequence similarity 120C	1.49E-02	3.14E-01
FAM181B	family with sequence similarity 181 member B	1.49E-02	-2.04E-01
TAF4B	TATA-box binding protein associated factor 4b	1.49E-02	7.53E-01
SMC5	structural maintenance of chromosomes 5	1.49E-02	3.83E-01
DCTN2	dynactin subunit 2	1.49E-02	-1.84E-01
MANBAL	mannosidase beta like	1.49E-02	-2.65E-01
ZBTB43	zinc finger and BTB domain containing 43	1.49E-02	-5.13E-01
JMJD4	jumonji domain containing 4	1.49E-02	-3.68E-01
SEMA6B	semaphorin 6B	1.49E-02	-3.87E-01

MYCBP	MYC binding protein	1.49E-02	1.91E -01
LOC101927038	uncharacterized LOC101927038	1.49E-02	2.15E -01
CDIP1	cell death inducing p53 target 1	1.49E-02	- 3.63E -01
FAM161A	family with sequence similarity 161 member A	1.50E-02	6.15E -01
KPTN	kaptin, actin binding protein	1.50E-02	- 4.88E -01
CHEK2	checkpoint kinase 2	1.50E-02	2.83E -01
TXNRD1	thioredoxin reductase 1	1.50E-02	8.50E -01
RGS4	regulator of G-protein signaling 4	1.50E-02	2.28E -01
GNAI1	G protein subunit alpha i1	1.50E-02	4.99E -01
HMBOX1	homeobox containing 1	1.50E-02	- 3.86E -01
RAD23A	RAD23 homolog A, nucleotide excision repair protein	1.50E-02	3.19E -01
LRIG3	leucine rich repeats and immunoglobulin like domains 3	1.50E-02	3.17E -01
KCNMB2	potassium calcium-activated channel subfamily M regulatory beta subunit 2	1.51E-02	- 2.30E -01
TUBB4A	tubulin beta 4A class IVa	1.51E-02	- 2.44E -01
VAV2	vav guanine nucleotide exchange factor 2	1.51E-02	- 2.06E -01

POMT1	protein O-mannosyltransferase 1	1.51E-02	3.22E-01
RPL23A	ribosomal protein L23a	1.51E-02	-3.16E-01
BRSK2	BR serine/threonine kinase 2	1.51E-02	-4.35E-01
ADK	adenosine kinase	1.51E-02	-5.08E-01
RAX2	retina and anterior neural fold homeobox 2	1.51E-02	-6.54E-01
WDR19	WD repeat domain 19	1.52E-02	-5.17E-01
TCTEX1D2	Tctex1 domain containing 2	1.52E-02	7.62E-01
SHC2	SHC adaptor protein 2	1.52E-02	-3.90E-01
TMEM55A	transmembrane protein 55A	1.52E-02	-5.91E-01
PTP4A3	protein tyrosine phosphatase type IVA, member 3	1.52E-02	-3.34E-01
HIST2H2AA4	histone cluster 2, H2aa4	1.52E-02	-7.09E-01
DNAJC5	DnaJ heat shock protein family (Hsp40) member C5	1.52E-02	2.68E-01
GRPEL1	GrpE like 1, mitochondrial	1.52E-02	2.47E-01
USP39	ubiquitin specific peptidase 39	1.52E-02	2.07E-01

SMG6	SMG6, nonsense mediated mRNA decay factor	1.52E-02	-2.27E-01
CCDC96	coiled-coil domain containing 96	1.52E-02	-1.99E-01
GALK2	galactokinase 2	1.52E-02	-4.39E-01
BBS2	Bardet-Biedl syndrome 2	1.52E-02	-6.27E-01
KLK1	kallikrein 1	1.52E-02	2.07E-01
NXN	nucleoredoxin	1.52E-02	-3.23E-01
SHISA5	shisa family member 5	1.52E-02	1.73E-01
CHCHD10	coiled-coil-helix-coiled-coil-helix domain containing 10	1.52E-02	-4.61E-01
C3orf62	chromosome 3 open reading frame 62	1.53E-02	-2.58E-01
SLC35F1	solute carrier family 35 member F1	1.53E-02	1.70E-01
ZIC2	Zic family member 2	1.53E-02	2.91E-01
PHF1	PHD finger protein 1	1.53E-02	-1.80E-01
MYNN	myoneurin	1.53E-02	3.27E-01
MAFG-AS1	MAFG antisense RNA 1 (head to head)	1.53E-02	-2.91E-01

ROCK1	Rho associated coiled-coil containing protein kinase 1	1.53E-02	8.30E-01
RPL10A	ribosomal protein L10a	1.53E-02	-2.59E-01
TEX11	testis expressed 11	1.53E-02	-2.24E-01
VEZT	vezatin, adherens junctions transmembrane protein	1.53E-02	5.87E-01
FHOD3	formin homology 2 domain containing 3	1.54E-02	2.19E-01
SRXN1	sulfiredoxin 1	1.54E-02	4.89E-01
RNPEP	arginyl aminopeptidase	1.54E-02	-4.79E-01
SNORA21	small nucleolar RNA, H/ACA box 21	1.54E-02	-3.87E-01
CTSL	cathepsin L	1.54E-02	-9.67E-01
PAQR5	progestin and adipoQ receptor family member 5	1.54E-02	-1.83E-01
NCBP2	nuclear cap binding protein subunit 2	1.54E-02	-4.50E-01
KDM4A	lysine demethylase 4A	1.54E-02	-3.01E-01
SPOP	speckle type BTB/POZ protein	1.54E-02	-3.71E-01
CSF2RA	colony stimulating factor 2 receptor alpha subunit	1.54E-02	-4.45E-01

WDR5	WD repeat domain 5	1.54E-02	-2.11E-01
RPL13P5	ribosomal protein L13 pseudogene 5	1.54E-02	-2.25E-01
ATL1	atlastin GTPase 1	1.55E-02	2.40E-01
SNCA	synuclein alpha	1.55E-02	-2.33E-01
SUPT16H	SPT16 homolog, facilitates chromatin remodeling subunit	1.55E-02	8.97E-01
PSTPIP2	proline-serine-threonine phosphatase interacting protein 2	1.55E-02	-3.33E-01
FAM120A	family with sequence similarity 120A	1.55E-02	3.27E-01
RBMY2FP	RNA binding motif protein, Y-linked, family 2, member F pseudogene	1.55E-02	-1.96E-01
KLF14	Kruppel like factor 14	1.56E-02	-2.30E-01
MRPL11	mitochondrial ribosomal protein L11	1.56E-02	5.96E-01
IL1R1	interleukin 1 receptor type 1	1.56E-02	3.81E-01
C6orf62	chromosome 6 open reading frame 62	1.56E-02	4.47E-01
TLR2	toll like receptor 2	1.56E-02	1.72E-01
AXIN1	axin 1	1.56E-02	3.61E-01
CRLF1	cytokine receptor like factor 1	1.56E-02	-2.90E-01

CCDC151	coiled-coil domain containing 151	1.56E-02	-5.42E-01
BORCS6	BLOC-1 related complex subunit 6	1.56E-02	-2.09E-01
TOP1	topoisomerase (DNA) I	1.56E-02	4.36E-01
OARD1	O-acyl-ADP-ribose deacylase 1	1.56E-02	-4.82E-01
ZSCAN31	zinc finger and SCAN domain containing 31	1.56E-02	3.35E-01
ARHGAP21	Rho GTPase activating protein 21	1.56E-02	2.89E-01
ZDHHC18	zinc finger DHHC-type containing 18	1.57E-02	3.92E-01
C1R	complement C1r subcomponent	1.57E-02	1.91E-01
KIF1B	kinesin family member 1B	1.57E-02	3.52E-01
LIPJ	lipase family member J	1.57E-02	2.21E-01
FOXD4L5	forkhead box D4-like 5	1.57E-02	-1.62E-01
HMGCL	3-hydroxymethyl-3-methylglutaryl-CoA lyase	1.57E-02	-4.07E-01
ARPC2	actin related protein 2/3 complex subunit 2	1.57E-02	2.66E-01
LIPE	lipase E, hormone sensitive type	1.57E-02	-2.50E-01
SRPK2	SRSF protein kinase 2	1.57E-02	-5.79E-01

CARNMT1	carnosine N-methyltransferase 1	1.57E-02	5.63E-01
NDRG4	NDRG family member 4	1.57E-02	-2.00E-01
ACOT2	acyl-CoA thioesterase 2	1.57E-02	-3.85E-01
SPCS2	signal peptidase complex subunit 2	1.57E-02	-3.42E-01
TLN1	talin 1	1.58E-02	-4.79E-01
LINC01119	long intergenic non-protein coding RNA 1119	1.58E-02	-2.85E-01
TIMM10B	translocase of inner mitochondrial membrane 10 homolog B (yeast)	1.58E-02	-2.00E-01
ZNF629	zinc finger protein 629	1.58E-02	-1.97E-01
FIGN	fidgetin, microtubule severing factor	1.58E-02	2.14E-01
SYNGR3	synaptogyrin 3	1.58E-02	-2.54E-01
PHF20L1	PHD finger protein 20-like 1	1.58E-02	2.87E-01
ERP44	endoplasmic reticulum protein 44	1.58E-02	-1.98E-01
RFX3	regulatory factor X3	1.58E-02	1.91E-01
PIK3R4	phosphoinositide-3-kinase regulatory subunit 4	1.58E-02	5.69E-01

MYBPHL	myosin binding protein H like	1.58E-02	-2.58E-01
ICA1	islet cell autoantigen 1	1.59E-02	2.16E-01
HIST2H2AB	histone cluster 2, H2ab	1.59E-02	-4.62E-01
NOVA1	NOVA alternative splicing regulator 1	1.59E-02	2.30E-01
RPLP0	ribosomal protein lateral stalk subunit P0	1.59E-02	-7.39E-01
MAGEF1	MAGE family member F1	1.59E-02	-5.67E-01
LOC100130476	uncharacterized LOC100130476	1.59E-02	-3.68E-01
CNOT4	CCR4-NOT transcription complex subunit 4	1.59E-02	4.11E-01
CENPM	centromere protein M	1.59E-02	5.91E-01
PXK	PX domain containing serine/threonine kinase like	1.59E-02	2.01E-01
FAM227B	family with sequence similarity 227 member B	1.60E-02	1.85E-01
MRPL47	mitochondrial ribosomal protein L47	1.60E-02	5.53E-01
RAB5B	RAB5B, member RAS oncogene family	1.60E-02	-5.11E-01
GNAL	G protein subunit alpha L	1.60E-02	2.56E-01
LLGL1	LLGL1, scribble cell polarity complex component	1.60E-02	-3.39E-01

DERL2	derlin 2	1.60E-02	-2.96E-01
XAB2	XPA binding protein 2	1.60E-02	4.65E-01
TPTE2P3	transmembrane phosphoinositide 3-phosphatase and tensin homolog 2 pseudogene 3	1.60E-02	-2.32E-01
ESF1	ESF1 nucleolar pre-rRNA processing protein homolog	1.60E-02	5.60E-01
ONECUT2	one cut homeobox 2	1.60E-02	-2.10E-01
GRHL3	grainyhead like transcription factor 3	1.61E-02	-2.41E-01
WARS	tryptophanyl-tRNA synthetase	1.61E-02	3.16E-01
ZSCAN2	zinc finger and SCAN domain containing 2	1.61E-02	-3.58E-01
C21orf2	chromosome 21 open reading frame 2	1.61E-02	-4.65E-01
ADAP2	ArfGAP with dual PH domains 2	1.61E-02	-3.74E-01
DPH3	diphthamide biosynthesis 3	1.61E-02	4.73E-01
MRPL9	mitochondrial ribosomal protein L9	1.61E-02	4.12E-01
MCCC1	methylcrotonoyl-CoA carboxylase 1	1.61E-02	-3.02E-01
PSMG3	proteasome assembly chaperone 3	1.61E-02	4.04E-01

SNX4	sorting nexin 4	1.61E-02	-3.73E-01
DPY19L4	dpy-19 like 4 (C. elegans)	1.62E-02	-3.86E-01
CTSC	cathepsin C	1.62E-02	1.96E-01
ATP6V1H	ATPase H+ transporting V1 subunit H	1.62E-02	-5.00E-01
RPS13	ribosomal protein S13	1.62E-02	3.06E-01
CCDC91	coiled-coil domain containing 91	1.62E-02	4.33E-01
MYO3B	myosin IIIB	1.62E-02	-4.53E-01
MIR761	microRNA 761	1.62E-02	2.73E-01
GALT	galactose-1-phosphate uridylyltransferase	1.62E-02	-4.92E-01
HADHA	hydroxyacyl-CoA dehydrogenase/3-ketoacyl-CoA thiolase/enoyl-CoA hydratase (trifunctional protein), alpha subunit	1.62E-02	-5.72E-01
WDR47	WD repeat domain 47	1.62E-02	1.92E-01
PRKDC	protein kinase, DNA-activated, catalytic polypeptide	1.62E-02	4.38E-01
PTPRH	protein tyrosine phosphatase, receptor type H	1.63E-02	-1.90E-01
ZC3HAV1	zinc finger CCCH-type containing, antiviral 1	1.63E-02	5.56E-01
PCDHGC3	protocadherin gamma subfamily C, 3	1.63E-02	1.68E-01

GNB5	G protein subunit beta 5	1.63E-02	2.15E-01
STXBP1	syntaxin binding protein 1	1.63E-02	2.90E-01
SLC9A7	solute carrier family 9 member A7	1.63E-02	2.78E-01
PDE4DIP	phosphodiesterase 4D interacting protein	1.63E-02	1.59E-01
ARHGEF6	Rac/Cdc42 guanine nucleotide exchange factor 6	1.63E-02	2.74E-01
FOXRED2	FAD dependent oxidoreductase domain containing 2	1.63E-02	4.04E-01
CAMKK2	calcium/calmodulin dependent protein kinase kinase 2	1.64E-02	-1.99E-01
RND1	Rho family GTPase 1	1.64E-02	1.75E-01
KPNB1	karyopherin subunit beta 1	1.64E-02	3.16E-01
GSKIP	GSK3B interacting protein	1.64E-02	3.71E-01
CCL3L3	C-C motif chemokine ligand 3 like 3	1.64E-02	2.26E-01
PSPC1	paraspeckle component 1	1.64E-02	4.08E-01
D2HGDH	D-2-hydroxyglutarate dehydrogenase	1.64E-02	-6.01E-01
ORMDL1	ORMDL sphingolipid biosynthesis regulator 1	1.64E-02	-2.09E-01
PCDH7	protocadherin 7	1.64E-02	-7.74E-01
CUL2	cullin 2	1.64E-02	3.73E-01

CACNG6	calcium voltage-gated channel auxiliary subunit gamma 6	1.64E-02	1.95E-01
DNAJB5	DnaJ heat shock protein family (Hsp40) member B5	1.64E-02	1.88E-01
HIST2H2AA3	histone cluster 2, H2aa3	1.64E-02	-9.06E-01
KIAA0391	KIAA0391	1.65E-02	2.72E-01
TET1	tet methylcytosine dioxygenase 1	1.65E-02	-4.60E-01
WBP11P1	WW domain binding protein 11 pseudogene 1	1.65E-02	2.89E-01
LINC00092	long intergenic non-protein coding RNA 92	1.65E-02	-3.67E-01
PKDCC	protein kinase domain containing, cytoplasmic	1.65E-02	-2.68E-01
DHX15	DEAH-box helicase 15	1.65E-02	6.56E-01
TNNT1	troponin T1, slow skeletal type	1.65E-02	-6.03E-01
CHMP6	charged multivesicular body protein 6	1.65E-02	-3.30E-01
KCTD5	potassium channel tetramerization domain containing 5	1.65E-02	-3.56E-01
PAPD4	poly(A) RNA polymerase D4, non-canonical	1.65E-02	6.83E-01
SBF1	SET binding factor 1	1.65E-02	-3.97E-01
GALNT13	polypeptide N-acetylgalactosaminyltransferase 13	1.65E-02	2.71E-01

USP21	ubiquitin specific peptidase 21	1.65E-02	-2.69E-01
BDNF	brain derived neurotrophic factor	1.65E-02	1.84E-01
MPC1L	mitochondrial pyruvate carrier 1-like	1.66E-02	-2.24E-01
RPP25L	ribonuclease P/MRP subunit p25 like	1.66E-02	-6.32E-01
C16orf74	chromosome 16 open reading frame 74	1.66E-02	-3.02E-01
FBXO38	F-box protein 38	1.66E-02	-4.94E-01
ACBD7	acyl-CoA binding domain containing 7	1.66E-02	-1.81E-01
CCDC103	coiled-coil domain containing 103	1.66E-02	2.56E-01
NBEAP1	neurobeachin pseudogene 1	1.66E-02	-2.27E-01
LMNA	lamin A/C	1.66E-02	-2.15E-01
CAMK1D	calcium/calmodulin dependent protein kinase ID	1.66E-02	-3.12E-01
SIPA1L1	signal induced proliferation associated 1 like 1	1.66E-02	-2.56E-01
RPS6	ribosomal protein S6	1.66E-02	-2.19E-01

ARL13B	ADP ribosylation factor like GTPase 13B	1.66E-02	8.26E-01
SLC5A8	solute carrier family 5 member 8	1.66E-02	-6.19E-01
TERC	telomerase RNA component	1.66E-02	2.15E-01
CSPP1	centrosome and spindle pole associated protein 1	1.66E-02	3.30E-01
PNO1	partner of NOB1 homolog	1.67E-02	3.37E-01
SNRPF	small nuclear ribonucleoprotein polypeptide F	1.67E-02	4.95E-01
XPO4	exportin 4	1.67E-02	4.01E-01
ACTR3B	ARP3 actin related protein 3 homolog B	1.67E-02	-2.58E-01
FZD4	frizzled class receptor 4	1.67E-02	-2.28E-01
BTBD3	BTB domain containing 3	1.67E-02	5.83E-01
BICDL2	BICD family like cargo adaptor 2	1.67E-02	2.04E-01
Sep-09	septin 9	1.67E-02	-4.06E-01
ZNF10	zinc finger protein 10	1.67E-02	3.76E-01
STOX2	storkhead box 2	1.67E-02	-3.44E-01
EHMT1	euchromatic histone lysine methyltransferase 1	1.67E-02	2.09E-01

EIF3A	eukaryotic translation initiation factor 3 subunit A	1.67E-02	-2.53E-01
NDUFS7	NADH:ubiquinone oxidoreductase core subunit S7	1.68E-02	-6.24E-01
RABL2B	RAB, member of RAS oncogene family-like 2B	1.68E-02	-3.42E-01
HEXB	hexosaminidase subunit beta	1.68E-02	1.66E-01
UIMC1	ubiquitin interaction motif containing 1	1.68E-02	-4.67E-01
LOC284648	uncharacterized LOC284648	1.68E-02	2.21E-01
MED12	mediator complex subunit 12	1.68E-02	-1.68E-01
TAF15	TATA-box binding protein associated factor 15	1.68E-02	4.13E-01
FNBP1L	formin binding protein 1 like	1.69E-02	3.68E-01
SIVA1	SIVA1 apoptosis inducing factor	1.69E-02	7.43E-01
INPP5F	inositol polyphosphate-5-phosphatase F	1.69E-02	-3.81E-01
TXNDC16	thioredoxin domain containing 16	1.69E-02	3.01E-01
PURG	purine rich element binding protein G	1.69E-02	-2.50E-01
LBP	lipopolysaccharide binding protein	1.69E-02	-1.57E-01

TIMM21	translocase of inner mitochondrial membrane 21	1.69E-02	-2.02E-01
CWC22	CWC22 homolog, spliceosome-associated protein	1.69E-02	9.49E-01
ZCCHC8	zinc finger CCHC-type containing 8	1.69E-02	4.51E-01
SNX29P2	sorting nexin 29 pseudogene 2	1.69E-02	-5.28E-01
SCMH1	sex comb on midleg homolog 1 (Drosophila)	1.69E-02	-2.41E-01
HSPA9	heat shock protein family A (Hsp70) member 9	1.70E-02	5.12E-01
MAD2L2	MAD2 mitotic arrest deficient-like 2 (yeast)	1.70E-02	4.27E-01
ERCC1	ERCC excision repair 1, endonuclease non-catalytic subunit	1.70E-02	-4.04E-01
SWSAP1	SWIM-type zinc finger 7 associated protein 1	1.70E-02	-3.46E-01
MAP2K3	mitogen-activated protein kinase kinase 3	1.70E-02	4.52E-01
WDR59	WD repeat domain 59	1.70E-02	-5.48E-01
ERBB2	erb-b2 receptor tyrosine kinase 2	1.70E-02	-5.51E-01
ATRN	attractin	1.70E-02	-3.46E-01
DCTN5	dynactin subunit 5	1.70E-02	-3.87E-01

ACBD3	acyl-CoA binding domain containing 3	1.70E-02	-6.75E-01
FAM71F1	family with sequence similarity 71 member F1	1.70E-02	1.63E-01
ITPRIPL1	inositol 1,4,5-trisphosphate receptor interacting protein-like 1	1.70E-02	2.06E-01
NPIPA1	nuclear pore complex interacting protein family member A1	1.70E-02	-2.64E-01
FYN	FYN proto-oncogene, Src family tyrosine kinase	1.71E-02	-3.21E-01
PREB	prolactin regulatory element binding	1.71E-02	-2.17E-01
CELSR3	cadherin EGF LAG seven-pass G-type receptor 3	1.71E-02	-5.11E-01
CRIP2	cysteine rich protein 2	1.71E-02	2.11E-01
Sep-10	septin 10	1.71E-02	4.16E-01
NUDT14	nudix hydrolase 14	1.71E-02	-5.78E-01
MTUS1	microtubule associated tumor suppressor 1	1.71E-02	1.89E-01
KMT2A	lysine methyltransferase 2A	1.72E-02	-5.18E-01
PARP1	poly(ADP-ribose) polymerase 1	1.72E-02	5.38E-01
LINC01184	long intergenic non-protein coding RNA 1184	1.72E-02	-2.97E-01
SEC23A	Sec23 homolog A, coat complex II component	1.72E-02	9.36E-01

SPINT1	serine peptidase inhibitor, Kunitz type 1	1.72E-02	1.65E-01
DHFR2	dihydrofolate reductase 2	1.72E-02	3.12E-01
RBFOX2	RNA binding protein, fox-1 homolog 2	1.72E-02	4.09E-01
TCERG1	transcription elongation regulator 1	1.73E-02	7.64E-01
SLC35F6	solute carrier family 35 member F6	1.73E-02	-4.33E-01
UGP2	UDP-glucose pyrophosphorylase 2	1.73E-02	1.90E-01
NUDT2	nudix hydrolase 2	1.73E-02	-4.96E-01
TBX1	T-box 1	1.73E-02	-2.81E-01
POTEC	POTE ankyrin domain family member C	1.73E-02	-1.68E-01
RFTN1	raftlin, lipid raft linker 1	1.73E-02	-3.05E-01
C21orf59	chromosome 21 open reading frame 59	1.73E-02	4.37E-01
HBQ1	hemoglobin subunit theta 1	1.73E-02	-6.30E-01
NCKAP5L	NCK associated protein 5 like	1.74E-02	-5.21E-01
IP6K2	inositol hexakisphosphate kinase 2	1.74E-02	-7.66E-01
ICA1L	islet cell autoantigen 1 like	1.74E-02	3.31E-01

HRAS	HRas proto-oncogene, GTPase	1.74E-02	-2.83E-01
HBZ	hemoglobin subunit zeta	1.74E-02	2.10E-01
MLLT10	myeloid/lymphoid or mixed-lineage leukemia; translocated to, 10	1.74E-02	-2.73E-01
UNC13A	unc-13 homolog A	1.74E-02	-3.44E-01
ZNF564	zinc finger protein 564	1.75E-02	-2.27E-01
BORCS5	BLOC-1 related complex subunit 5	1.75E-02	1.65E-01
CHTF18	chromosome transmission fidelity factor 18	1.75E-02	3.77E-01
TXN	thioredoxin	1.75E-02	4.06E-01
RPGRIP1L	RPGRIP1 like	1.75E-02	1.70E-01
REXO1	RNA exonuclease 1 homolog	1.75E-02	5.15E-01
SDF2	stromal cell derived factor 2	1.76E-02	-3.65E-01
GLE1	GLE1, RNA export mediator	1.76E-02	3.11E-01
COG4	component of oligomeric golgi complex 4	1.76E-02	-3.47E-01
C17orf51	chromosome 17 open reading frame 51	1.76E-02	2.55E-01
PDCD6IP	programmed cell death 6 interacting protein	1.76E-02	-8.37E-01

CLDND1	claudin domain containing 1	1.76E-02	3.17E-01
NPB	neuropeptide B	1.76E-02	-1.75E-01
PCTP	phosphatidylcholine transfer protein	1.76E-02	-4.33E-01
ZMYM4	zinc finger MYM-type containing 4	1.76E-02	-4.36E-01
SSH3	slingshot protein phosphatase 3	1.76E-02	3.45E-01
WNK1	WNK lysine deficient protein kinase 1	1.76E-02	2.29E-01
LOC494141	solute carrier family 25 member 51 pseudogene	1.76E-02	2.03E-01
OS9	OS9, endoplasmic reticulum lectin	1.76E-02	-3.00E-01
RPS6KA3	ribosomal protein S6 kinase A3	1.76E-02	5.83E-01
KIAA2026	KIAA2026	1.77E-02	-4.71E-01
STK19	serine/threonine kinase 19	1.77E-02	-4.50E-01
LRRC26	leucine rich repeat containing 26	1.77E-02	2.28E-01
PLEKHH3	pleckstrin homology, MyTH4 and FERM domain containing H3	1.77E-02	-4.32E-01
SNCB	synuclein beta	1.77E-02	-2.00E-01

BTG4	BTG anti-proliferation factor 4	1.77E-02	-3.71E-01
RBMX2	RNA binding motif protein, X-linked 2	1.77E-02	-2.22E-01
PTPRO	protein tyrosine phosphatase, receptor type O	1.77E-02	-2.63E-01
TSPAN4	tetraspanin 4	1.77E-02	-5.46E-01
GLMN	glomulin, FKBP associated protein	1.77E-02	2.18E-01
JAGN1	jagunal homolog 1	1.77E-02	-3.50E-01
RPL34	ribosomal protein L34	1.78E-02	-6.13E-01
ZNF789	zinc finger protein 789	1.78E-02	-1.96E-01
SPRR1A	small proline rich protein 1A	1.78E-02	-2.75E-01
MFAP5	microfibrillar associated protein 5	1.78E-02	-2.49E-01
ACO1	aconitase 1	1.78E-02	4.77E-01
CCDC134	coiled-coil domain containing 134	1.79E-02	1.87E-01
DEF6	DEF6, guanine nucleotide exchange factor	1.79E-02	3.57E-01
ACSM6	acyl-CoA synthetase medium-chain family member 6	1.79E-02	1.69E-01

LTBP2	latent transforming growth factor beta binding protein 2	1.79E-02	-2.75E-01
PLAC9	placenta specific 9	1.79E-02	-2.40E-01
SFR1	SWI5 dependent homologous recombination repair protein 1	1.79E-02	1.84E-01
SAMD14	sterile alpha motif domain containing 14	1.79E-02	2.23E-01
LOC100288893	uncharacterized LOC100288893	1.79E-02	-1.97E-01
RASA2	RAS p21 protein activator 2	1.79E-02	3.13E-01
DPM1	dolichyl-phosphate mannosyltransferase subunit 1, catalytic	1.80E-02	-2.74E-01
LUC7L3	LUC7 like 3 pre-mRNA splicing factor	1.80E-02	-4.55E-01
ZNF876P	zinc finger protein 876, pseudogene	1.80E-02	1.66E-01
ZNF706	zinc finger protein 706	1.80E-02	-2.23E-01
CDKN1C	cyclin dependent kinase inhibitor 1C	1.80E-02	-2.20E-01
SLC39A3	solute carrier family 39 member 3	1.80E-02	-7.17E-01
ANGPT2	angiopoietin 2	1.80E-02	-1.84E-01
TMEM230	transmembrane protein 230	1.80E-02	2.05E-01

ANKMY1	ankyrin repeat and MYND domain containing 1	1.80E-02	-2.89E-01
SUMO1P3	SUMO1 pseudogene 3	1.80E-02	5.31E-01
RPL23AP7	ribosomal protein L23a pseudogene 7	1.80E-02	-8.17E-01
ADAM22	ADAM metallopeptidase domain 22	1.81E-02	3.72E-01
CNBP	CCHC-type zinc finger nucleic acid binding protein	1.81E-02	-3.22E-01
SLC25A33	solute carrier family 25 member 33	1.81E-02	5.39E-01
STAT3	signal transducer and activator of transcription 3	1.81E-02	-3.80E-01
TCEANC2	transcription elongation factor A N-terminal and central domain containing 2	1.81E-02	-2.14E-01
CCS	copper chaperone for superoxide dismutase	1.81E-02	-1.06
MYL6B	myosin light chain 6B	1.81E-02	-6.53E-01
GALNT6	polypeptide N-acetylgalactosaminyltransferase 6	1.81E-02	2.17E-01
CLOCK	clock circadian regulator	1.81E-02	3.46E-01
MMP19	matrix metallopeptidase 19	1.81E-02	1.89E-01
NPAT	nuclear protein, coactivator of histone transcription	1.81E-02	5.58E-01
KLHL11	kelch like family member 11	1.81E-02	1.61E-01

HSPBAP1	HSPB1 associated protein 1	1.81E-02	-2.38E-01
SNORD38A	small nucleolar RNA, C/D box 38A	1.81E-02	-2.69E-01
LIN7C	lin-7 homolog C, crumbs cell polarity complex component	1.81E-02	4.76E-01
USP34	ubiquitin specific peptidase 34	1.82E-02	-3.87E-01
C2orf49	chromosome 2 open reading frame 49	1.82E-02	6.32E-01
BCAN	brevican	1.82E-02	2.36E-01
RHBDD1	rhomboid domain containing 1	1.82E-02	1.80E-01
SUMO2	small ubiquitin-like modifier 2	1.82E-02	3.00E-01
TCEA1	transcription elongation factor A1	1.82E-02	8.05E-01
HSD17B1	hydroxysteroid 17-beta dehydrogenase 1	1.82E-02	-7.49E-01
ARHGEF25	Rho guanine nucleotide exchange factor 25	1.82E-02	-2.53E-01
GNE	glucosamine (UDP-N-acetyl)-2-epimerase/N-acetylmannosamine kinase	1.82E-02	-3.71E-01
SRI	sorcin	1.82E-02	3.83E-01
CHIC1	cysteine rich hydrophobic domain 1	1.83E-02	1.94E-01
SRD5A1	steroid 5 alpha-reductase 1	1.83E-02	3.26E-01

CHMP2A	charged multivesicular body protein 2A	1.83E-02	-6.64E-01
PFDN5	prefoldin subunit 5	1.83E-02	-2.40E-01
USP10	ubiquitin specific peptidase 10	1.83E-02	6.29E-01
FBXO31	F-box protein 31	1.83E-02	4.67E-01
NAA16	N(alpha)-acetyltransferase 16, NatA auxiliary subunit	1.83E-02	2.78E-01
IVNS1ABP	influenza virus NS1A binding protein	1.83E-02	5.97E-01
TMEM74B	transmembrane protein 74B	1.83E-02	-4.41E-01
PCYOX1L	prenylcysteine oxidase 1 like	1.84E-02	7.28E-01
CT45A1	cancer/testis antigen family 45, member A1	1.84E-02	2.92E-01
PLPP2	phospholipid phosphatase 2	1.84E-02	-2.43E-01
RHOG	ras homolog family member G	1.84E-02	-3.85E-01
C1orf50	chromosome 1 open reading frame 50	1.85E-02	-7.03E-01
OSCP1	organic solute carrier partner 1	1.85E-02	2.42E-01
HMGCS1	3-hydroxy-3-methylglutaryl-CoA synthase 1	1.85E-02	-3.18E-01
RIT1	Ras like without CAAX 1	1.85E-02	-4.99E-01

TSPAN4	tetraspanin 4	1.85E-02	-6.14E-01
MRPL18	mitochondrial ribosomal protein L18	1.85E-02	3.88E-01
CACNB2	calcium voltage-gated channel auxiliary subunit beta 2	1.85E-02	-2.30E-01
HDAC5	histone deacetylase 5	1.85E-02	-1.86E-01
APBB3	amyloid beta precursor protein binding family B member 3	1.85E-02	-5.85E-01
MT1H	metallothionein 1H	1.85E-02	1.95E-01
UBE2I	ubiquitin conjugating enzyme E2 I	1.85E-02	2.13E-01
DNASE2B	deoxyribonuclease 2 beta	1.85E-02	-1.63E-01
NQO2	NAD(P)H quinone dehydrogenase 2	1.85E-02	5.80E-01
ALDH3A2	aldehyde dehydrogenase 3 family member A2	1.86E-02	4.59E-01
SPANXD	SPANX family member D	1.86E-02	2.05E-01
MR1	major histocompatibility complex, class I-related	1.86E-02	-3.78E-01
TNFRSF19	TNF receptor superfamily member 19	1.86E-02	2.41E-01
NRXN1	neurexin 1	1.87E-02	-3.04E-01
CIRBP	cold inducible RNA binding protein	1.87E-02	-3.72E-01

MRPL46	mitochondrial ribosomal protein L46	1.87E-02	5.26E-01
ORAI3	ORAI calcium release-activated calcium modulator 3	1.87E-02	-3.76E-01
ARAF	A-Raf proto-oncogene, serine/threonine kinase	1.87E-02	6.58E-01
STAM2	signal transducing adaptor molecule 2	1.87E-02	5.82E-01
PLS3	plastin 3	1.87E-02	4.88E-01
DLG5-AS1	DLG5 antisense RNA 1	1.87E-02	1.84E-01
G3BP2	G3BP stress granule assembly factor 2	1.88E-02	6.63E-01
ZC4H2	zinc finger C4H2-type containing	1.88E-02	2.32E-01
UBE2E1	ubiquitin conjugating enzyme E2 E1	1.88E-02	4.59E-01
FOXE1	forkhead box E1	1.88E-02	2.16E-01
NUDT18	nudix hydrolase 18	1.88E-02	-4.53E-01
FRS3	fibroblast growth factor receptor substrate 3	1.88E-02	-5.38E-01
SCAND2P	SCAN domain containing 2 pseudogene	1.88E-02	-1.53E-01
BSG	basigin (Ok blood group)	1.88E-02	-3.53E-01
EML5	echinoderm microtubule associated protein like 5	1.88E-02	2.03E-01

TAF1L	TATA-box binding protein associated factor 1 like	1.88E-02	-2.40E-01
SLC22A2	solute carrier family 22 member 2	1.88E-02	-1.71E-01
CLCN2	chloride voltage-gated channel 2	1.89E-02	-1.74E-01
MXRA5	matrix remodeling associated 5	1.89E-02	-1.82E-01
LINC00961	long intergenic non-protein coding RNA 961	1.89E-02	2.28E-01
ALKBH1	alkB homolog 1, histone H2A dioxygenase	1.89E-02	3.21E-01
WIPF1	WAS/WASL interacting protein family member 1	1.90E-02	2.05E-01
GABRA5	gamma-aminobutyric acid type A receptor alpha5 subunit	1.90E-02	-1.72E-01
RUFY2	RUN and FYVE domain containing 2	1.90E-02	3.99E-01
KIAA0141	KIAA0141	1.90E-02	-3.86E-01
FAM63A	family with sequence similarity 63 member A	1.90E-02	-6.95E-01
ISCA2	iron-sulfur cluster assembly 2	1.90E-02	4.19E-01
USP50	ubiquitin specific peptidase 50	1.90E-02	1.52E-01
YRDC	yrdC N6-threonylcarbamoyltransferase domain containing	1.90E-02	1.84E-01
TMEM214	transmembrane protein 214	1.90E-02	-3.50E-01

METTL22	methyltransferase like 22	1.90E-02	-3.11E-01
VRK3	vaccinia related kinase 3	1.91E-02	-5.72E-01
SMIM19	small integral membrane protein 19	1.91E-02	-3.60E-01
SNRNP70	small nuclear ribonucleoprotein U1 subunit 70	1.91E-02	3.90E-01
SNORD36C	small nucleolar RNA, C/D box 36C	1.91E-02	-3.21E-01
GAN	gigaxonin	1.91E-02	1.54E-01
LOC727924	uncharacterized LOC727924	1.91E-02	-1.66E-01
HNRNPH1	heterogeneous nuclear ribonucleoprotein H1 (H)	1.92E-02	3.97E-01
ACSL1	acyl-CoA synthetase long-chain family member 1	1.92E-02	4.73E-01
PRDM4	PR/SET domain 4	1.92E-02	-3.22E-01
TRMT2B	tRNA methyltransferase 2 homolog B	1.92E-02	2.06E-01
ZNF24	zinc finger protein 24	1.92E-02	3.87E-01
FNDC5	fibronectin type III domain containing 5	1.92E-02	-8.85E-01
KCNE2	potassium voltage-gated channel subfamily E regulatory subunit 2	1.93E-02	2.02E-01
SNORD3C	small nucleolar RNA, C/D box 3C	1.93E-02	-7.10E-01

ANKRD13D	ankyrin repeat domain 13D	1.93E-02	-5.66E-01
FAM20C	family with sequence similarity 20 member C	1.93E-02	-4.60E-01
SPTLC1	serine palmitoyltransferase long chain base subunit 1	1.93E-02	-4.08E-01
COX6B2	cytochrome c oxidase subunit 6B2	1.93E-02	-2.02E-01
GHDC	GH3 domain containing	1.93E-02	-3.41E-01
ERMP1	endoplasmic reticulum metallopeptidase 1	1.94E-02	-1.85E-01
RNF146	ring finger protein 146	1.94E-02	5.37E-01
IMP3	IMP3, U3 small nucleolar ribonucleoprotein	1.94E-02	-2.14E-01
DBI	diazepam binding inhibitor, acyl-CoA binding protein	1.95E-02	3.38E-01
DLX4	distal-less homeobox 4	1.95E-02	-2.56E-01
YBEY	ybeY metallopeptidase (putative)	1.95E-02	-7.87E-01
CSNK1A1	casein kinase 1 alpha 1	1.95E-02	2.11E-01
TBXA2R	thromboxane A2 receptor	1.95E-02	-2.05E-01
MZT1	mitotic spindle organizing protein 1	1.95E-02	5.09E-01

FIRRE	firre intergenic repeating RNA element	1.95E-02	1.97E-01
CRK	CRK proto-oncogene, adaptor protein	1.95E-02	-4.24E-01
NUPL2	nucleoporin like 2	1.95E-02	2.87E-01
MELTF	melanotransferrin	1.95E-02	-1.72E-01
MTMR3	myotubularin related protein 3	1.96E-02	5.89E-01
HCG4	HLA complex group 4 (non-protein coding)	1.96E-02	-1.83E-01
HBG1	hemoglobin subunit gamma 1	1.96E-02	-1.69E-01
PLAUR	plasminogen activator, urokinase receptor	1.96E-02	1.99E-01
MTX2	metaxin 2	1.96E-02	2.71E-01
CHST3	carbohydrate sulfotransferase 3	1.96E-02	-2.23E-01
NCAN	neurocan	1.96E-02	-2.64E-01
B3GAT3	beta-1,3-glucuronyltransferase 3	1.96E-02	-2.99E-01
AMZ2	archaelysin family metallopeptidase 2	1.96E-02	-4.19E-01
ZNF81	zinc finger protein 81	1.97E-02	-4.04E-01

LYRM7	LYR motif containing 7	1.97E-02	-2.81E-01
C11orf91	chromosome 11 open reading frame 91	1.97E-02	1.70E-01
TSC22D3	TSC22 domain family member 3	1.97E-02	-1.67E-01
DENND4C	DENN domain containing 4C	1.97E-02	-4.15E-01
FAM89B	family with sequence similarity 89 member B	1.97E-02	-4.41E-01
IQCG	IQ motif containing G	1.97E-02	2.51E-01
CYP2R1	cytochrome P450 family 2 subfamily R member 1	1.97E-02	2.86E-01
TMEM191A	transmembrane protein 191A (pseudogene)	1.97E-02	-5.81E-01
SMYD2	SET and MYND domain containing 2	1.97E-02	-2.15E-01
SNRNP48	small nuclear ribonucleoprotein U11/U12 subunit 48	1.97E-02	2.70E-01
PIH1D3	PIH1 domain containing 3	1.98E-02	1.87E-01
PLGLA	plasminogen-like A (pseudogene)	1.98E-02	3.03E-01
NTN1	netrin 1	1.98E-02	-1.57E-01
MAP2K3	mitogen-activated protein kinase kinase 3	1.98E-02	-2.32E-01
LOXL3	lysyl oxidase like 3	1.98E-02	1.57E-01

ZNF490	zinc finger protein 490	1.98E-02	-1.98E-01
NSUN5	NOP2/Sun RNA methyltransferase family member 5	1.98E-02	-2.85E-01
PIGM	phosphatidylinositol glycan anchor biosynthesis class M	1.98E-02	-1.79E-01
BRE	brain and reproductive organ-expressed (TNFRSF1A modulator)	1.98E-02	-3.87E-01
ZNF562	zinc finger protein 562	1.99E-02	-3.93E-01
SLC31A1	solute carrier family 31 member 1	1.99E-02	-3.68E-01
TXN2	thioredoxin 2	1.99E-02	-4.59E-01
SSBP4	single stranded DNA binding protein 4	1.99E-02	-4.82E-01
TPMT	thiopurine S-methyltransferase	1.99E-02	4.58E-01
PRAMEF6	PRAME family member 6	1.99E-02	-1.72E-01
FLT4	fms related tyrosine kinase 4	1.99E-02	1.96E-01
TRPM6	transient receptor potential cation channel subfamily M member 6	1.99E-02	3.26E-01
GAD1	glutamate decarboxylase 1	1.99E-02	-3.71E-01

SEC61A1	Sec61 translocon alpha 1 subunit	2.00E-02	-6.12E-01
CD19	CD19 molecule	2.00E-02	-1.66E-01
ZNF667	zinc finger protein 667	2.00E-02	-6.09E-01
NSFL1C	NSFL1 cofactor	2.00E-02	3.19E-01
FAM131C	family with sequence similarity 131 member C	2.00E-02	-1.93E-01
CACHD1	cache domain containing 1	2.00E-02	-6.89E-01
PDGFB	platelet derived growth factor subunit B	2.00E-02	2.15E-01
PITHD1	PITH domain containing 1	2.01E-02	4.62E-01
GCHFR	GTP cyclohydrolase I feedback regulator	2.01E-02	-7.79E-01
DZIP3	DAZ interacting zinc finger protein 3	2.01E-02	-1.74E-01
PLCH2	phospholipase C eta 2	2.01E-02	-2.00E-01
PRMT6	protein arginine methyltransferase 6	2.01E-02	3.47E-01
EIF2AK1	eukaryotic translation initiation factor 2 alpha kinase 1	2.01E-02	4.49E-01
PHGDH	phosphoglycerate dehydrogenase	2.01E-02	-3.38E-01

RBM48	RNA binding motif protein 48	2.01E-02	2.77E-01
TEX26	testis expressed 26	2.01E-02	-1.68E-01
DEPDC5	DEP domain containing 5	2.02E-02	-2.36E-01
SUMF2	sulfatase modifying factor 2	2.02E-02	-3.70E-01
AP1AR	adaptor related protein complex 1 associated regulatory protein	2.03E-02	5.82E-01
GTF2A1L	general transcription factor IIA subunit 1 like	2.03E-02	-2.37E-01
FBXO33	F-box protein 33	2.03E-02	-1.97E-01
SMARCB1	SWI/SNF related, matrix associated, actin dependent regulator of chromatin, subfamily b, member 1	2.03E-02	2.34E-01
ALDH3A1	aldehyde dehydrogenase 3 family member A1	2.03E-02	-2.05E-01
EIF5	eukaryotic translation initiation factor 5	2.03E-02	-1.73E-01
C11orf68	chromosome 11 open reading frame 68	2.03E-02	-1.78E-01
NAGA	alpha-N-acetylgalactosaminidase	2.03E-02	3.19E-01
MAN1A2	mannosidase alpha class 1A member 2	2.04E-02	3.31E-01
HEATR3	HEAT repeat containing 3	2.04E-02	-2.85E-01

RPS6KA6	ribosomal protein S6 kinase A6	2.04E-02	3.18E-01
PRKCSH	protein kinase C substrate 80K-H	2.04E-02	-2.71E-01
RNY3	RNA, Ro-associated Y3	2.04E-02	-2.76E-01
CCDC22	coiled-coil domain containing 22	2.05E-02	1.65E-01
ANGPTL4	angiopoietin like 4	2.05E-02	-4.27E-01
PPP4R4	protein phosphatase 4 regulatory subunit 4	2.05E-02	2.25E-01
TRAPPC5	trafficking protein particle complex 5	2.05E-02	-6.35E-01
DEPDC7	DEP domain containing 7	2.05E-02	4.44E-01
RNF114	ring finger protein 114	2.05E-02	-2.56E-01
TMEM44	transmembrane protein 44	2.05E-02	-5.31E-01
SATL1	spermidine/spermine N1-acetyl transferase-like 1	2.05E-02	-2.28E-01
UBR4	ubiquitin protein ligase E3 component n-recognin 4	2.05E-02	5.38E-01
GRINA	glutamate ionotropic receptor NMDA type subunit associated protein 1	2.06E-02	-4.19E-01
GLB1	galactosidase beta 1	2.06E-02	-2.63E-01

MTMR2	myotubularin related protein 2	2.06E-02	2.62E-01
LYPLA2	lysophospholipase II	2.06E-02	-6.13E-01
ANKRD6	ankyrin repeat domain 6	2.06E-02	2.58E-01
SARS2	seryl-tRNA synthetase 2, mitochondrial	2.06E-02	-3.23E-01
APCDD1	APC down-regulated 1	2.06E-02	-1.71E-01
FRAT1	frequently rearranged in advanced T-cell lymphomas 1	2.06E-02	-1.61E-01
MGC57346	ADP-ribosylation factor pseudogene	2.06E-02	4.39E-01
REEP4	receptor accessory protein 4	2.06E-02	3.58E-01
RAB27A	RAB27A, member RAS oncogene family	2.07E-02	1.57E-01
CCL20	C-C motif chemokine ligand 20	2.07E-02	1.67E-01
TDRD6	tudor domain containing 6	2.07E-02	-2.18E-01
ZNF141	zinc finger protein 141	2.07E-02	2.09E-01
IPP	intracisternal A particle-promoted polypeptide	2.07E-02	-3.43E-01
SOS2	SOS Ras/Rho guanine nucleotide exchange factor 2	2.07E-02	1.64E-01
INTS8	integrator complex subunit 8	2.07E-02	4.80E-01

PFDN4	prefoldin subunit 4	2.07E-02	4.82E-01
TMEM27	transmembrane protein 27	2.08E-02	1.73E-01
CEP192	centrosomal protein 192	2.08E-02	3.25E-01
ARPC4	actin related protein 2/3 complex subunit 4	2.08E-02	-6.99E-01
CBX2	chromobox 2	2.08E-02	-2.64E-01
KAT6A	lysine acetyltransferase 6A	2.08E-02	-1
SKIV2L2	Ski2 like RNA helicase 2	2.08E-02	7.15E-01
NEK1	NIMA related kinase 1	2.08E-02	3.18E-01
RAD17	RAD17 checkpoint clamp loader component	2.08E-02	-2.31E-01
CRCP	CGRP receptor component	2.08E-02	1.78E-01
COMMD1	copper metabolism domain containing 1	2.08E-02	4.80E-01
CLDN11	claudin 11	2.08E-02	-2.55E-01
NOP2	NOP2 nucleolar protein	2.08E-02	2.94E-01
PLEKHF2	pleckstrin homology and FYVE domain containing 2	2.08E-02	2.98E-01
PPP4R1	protein phosphatase 4 regulatory subunit 1	2.08E-02	6.14E-01
ATAT1	alpha tubulin acetyltransferase 1	2.09E-02	-2.45E-01
NCKAP1	NCK associated protein 1	2.09E-02	1.11

MFN2	mitofusin 2	2.09E-02	4.82E-01
TFG	TRK-fused gene	2.09E-02	-4.96E-01
ERI3	ERI1 exoribonuclease family member 3	2.09E-02	5.11E-01
PDIK1L	PDLIM1 interacting kinase 1 like	2.09E-02	4.25E-01
GID4	GID complex subunit 4 homolog	2.10E-02	2.98E-01
TBX1	T-box 1	2.10E-02	-2.88E-01
PRELID3B	PRELI domain containing 3B	2.10E-02	3.86E-01
INTS6-AS1	INTS6 antisense RNA 1	2.10E-02	-1.77E-01
ATG4A	autophagy related 4A cysteine peptidase	2.10E-02	-1.79E-01
ACAD8	acyl-CoA dehydrogenase family member 8	2.10E-02	-3.50E-01
S100PBP	S100P binding protein	2.10E-02	-2.18E-01
ZBTB21	zinc finger and BTB domain containing 21	2.10E-02	4.33E-01
MYLK	myosin light chain kinase	2.10E-02	2.41E-01
RASAL3	RAS protein activator like 3	2.10E-02	-2.19E-01
DENND2C	DENN domain containing 2C	2.11E-02	3.06E-01

PPP1R2P3	protein phosphatase 1 regulatory inhibitor subunit 2 pseudogene 3	2.11E-02	-1.87E-01
TXNDC5	thioredoxin domain containing 5	2.11E-02	5.57E-01
RFXANK	regulatory factor X associated ankyrin containing protein	2.11E-02	-7.32E-01
NCDN	neurochondrin	2.11E-02	-2.84E-01
HTR4	5-hydroxytryptamine receptor 4	2.11E-02	-1.59E-01
C6orf52	chromosome 6 open reading frame 52	2.11E-02	3.75E-01
TOMM5	translocase of outer mitochondrial membrane 5	2.11E-02	4.99E-01
HAT1	histone acetyltransferase 1	2.12E-02	5.64E-01
MED23	mediator complex subunit 23	2.12E-02	-4.49E-01
USP6	ubiquitin specific peptidase 6	2.12E-02	-8.24E-01
ENTPD3	ectonucleoside triphosphate diphosphohydrolase 3	2.12E-02	-3.26E-01
GAGE12H	G antigen 12H	2.12E-02	-2.09E-01
NTAN1	N-terminal asparagine amidase	2.13E-02	2.67E-01
GNB1L	G protein subunit beta 1 like	2.13E-02	-3.15E-01

BEND6	BEN domain containing 6	2.13E-02	1.62E-01
INTU	inturned planar cell polarity protein	2.13E-02	3.49E-01
CARD17	caspase recruitment domain family member 17	2.13E-02	-1.60E-01
HNRNPUL1	heterogeneous nuclear ribonucleoprotein U like 1	2.13E-02	-4.40E-01
DICER1	dicer 1, ribonuclease III	2.13E-02	1.72E-01
DENR	density regulated re-initiation and release factor	2.13E-02	-5.40E-01
XRN2	5'-3' exoribonuclease 2	2.13E-02	5.36E-01
GLT1D1	glycosyltransferase 1 domain containing 1	2.13E-02	1.57E-01
HIST1H2BE	histone cluster 1, H2be	2.13E-02	-4.69E-01
THAP1	THAP domain containing 1	2.13E-02	2.87E-01
PDIA2	protein disulfide isomerase family A member 2	2.13E-02	-2.09E-01
TUSC3	tumor suppressor candidate 3	2.13E-02	-2.46E-01
AGTPBP1	ATP/GTP binding protein 1	2.13E-02	6.26E-01
TRIM56	tripartite motif containing 56	2.13E-02	2.00E-01
HILPDA	hypoxia inducible lipid droplet associated	2.13E-02	-7.07E-01

TBC1D19	TBC1 domain family member 19	2.13E-02	2.25E-01
WDR45	WD repeat domain 45	2.13E-02	-5.79E-01
MEIG1	meiosis/spermiogenesis associated 1	2.14E-02	1.94E-01
AGO3	argonaute 3, RISC catalytic component	2.14E-02	2.08E-01
PEMT	phosphatidylethanolamine N-methyltransferase	2.14E-02	-5.35E-01
GATC	glutamyl-tRNA amidotransferase subunit C	2.15E-02	-5.01E-01
ALDH3A2	aldehyde dehydrogenase 3 family member A2	2.15E-02	3.22E-01
PTCH1	patched 1	2.15E-02	-2.26E-01
PLD3	phospholipase D family member 3	2.15E-02	-1.77E-01
CNST	consortin, connexin sorting protein	2.15E-02	-8.59E-01
SNUPN	snurportin 1	2.15E-02	3.94E-01
UPF2	UPF2 regulator of nonsense transcripts homolog (yeast)	2.15E-02	-8.49E-01
FAM189B	family with sequence similarity 189 member B	2.15E-02	-3.50E-01
RPL14	ribosomal protein L14	2.15E-02	-4.18E-01

DUT	deoxyuridine triphosphatase	2.15E-02	-6.93E-01
PRDX3	peroxiredoxin 3	2.15E-02	3.89E-01
OPN5	opsin 5	2.15E-02	-1.87E-01
SMAD5	SMAD family member 5	2.15E-02	-7.78E-01
EIF2S3	eukaryotic translation initiation factor 2 subunit gamma	2.15E-02	-2.44E-01
BICDL2	BICD family like cargo adaptor 2	2.16E-02	1.60E-01
MROH6	maestro heat like repeat family member 6	2.16E-02	2.46E-01
TFPT	TCF3 fusion partner	2.16E-02	-6.98E-01
MOB4	MOB family member 4, phocein	2.16E-02	6.21E-01
ALDH1B1	aldehyde dehydrogenase 1 family member B1	2.16E-02	3.59E-01
FAM35A	family with sequence similarity 35 member A	2.16E-02	6.37E-01
CPE	carboxypeptidase E	2.16E-02	-5.53E-01
CFH	complement factor H	2.16E-02	-3.55E-01
USP42	ubiquitin specific peptidase 42	2.16E-02	-1.56E-01

BPTF	bromodomain PHD finger transcription factor	2.16E-02	-4.99E-01
EPHB2	EPH receptor B2	2.16E-02	-2.47E-01
SEC24A	SEC24 homolog A, COPII coat complex component	2.17E-02	-3.32E-01
HEG1	heart development protein with EGF like domains 1	2.17E-02	2.60E-01
NAA10	N(alpha)-acetyltransferase 10, NatA catalytic subunit	2.17E-02	5.80E-01
SLC7A6	solute carrier family 7 member 6	2.17E-02	-3.45E-01
SMAD4	SMAD family member 4	2.17E-02	3.26E-01
LOC100131023	uncharacterized LOC100131023	2.17E-02	-1.99E-01
HOOK3	hook microtubule tethering protein 3	2.17E-02	4.05E-01
DUS3L	dihydrouridine synthase 3 like	2.17E-02	-3.04E-01
C18orf54	chromosome 18 open reading frame 54	2.17E-02	3.65E-01
OLA1	Obg-like ATPase 1	2.18E-02	5.28E-01
CUL1	cullin 1	2.18E-02	4.98E-01
COG8	component of oligomeric golgi complex 8	2.18E-02	-2.96E-01
Sep-05	septin 5	2.18E-02	-7.11E-01

RWDD2A	RWD domain containing 2A	2.18E-02	3.67E-01
MORC2-AS1	MORC2 antisense RNA 1	2.18E-02	-4.02E-01
POM121C	POM121 transmembrane nucleoporin C	2.19E-02	-5.12E-01
TAF1A	TATA-box binding protein associated factor, RNA polymerase I subunit A	2.19E-02	3.18E-01
TMEM217	transmembrane protein 217	2.19E-02	-1.60E-01
SCN2A	sodium voltage-gated channel alpha subunit 2	2.19E-02	-2.07E-01
GGTLC1	gamma-glutamyltransferase light chain 1	2.19E-02	3.78E-01
ZNF143	zinc finger protein 143	2.19E-02	4.29E-01
PDE1A	phosphodiesterase 1A	2.19E-02	-2.10E-01
MIR607	microRNA 607	2.19E-02	-3.67E-01
SEMA4D	semaphorin 4D	2.20E-02	4.57E-01
CLIP2	CAP-Gly domain containing linker protein 2	2.20E-02	-3.13E-01
MED7	mediator complex subunit 7	2.20E-02	3.24E-01
GRK6	G protein-coupled receptor kinase 6	2.20E-02	2.38E-01
INIP	INTS3 and NABP interacting protein	2.20E-02	-5.06E-01

CCDC80	coiled-coil domain containing 80	2.21E-02	-3.93E-01
ZBTB40	zinc finger and BTB domain containing 40	2.21E-02	-4.89E-01
TXNDC17	thioredoxin domain containing 17	2.21E-02	2.74E-01
PLXNB1	plexin B1	2.21E-02	2.51E-01
UTP20	UTP20, small subunit processome component	2.21E-02	2.67E-01
PTPRA	protein tyrosine phosphatase, receptor type A	2.21E-02	-2.12E-01
TMEM121	transmembrane protein 121	2.22E-02	2.42E-01
DPH1	diphthamide biosynthesis 1	2.22E-02	-2.01E-01
DGKB	diacylglycerol kinase beta	2.22E-02	-2.17E-01
SUMO1	small ubiquitin-like modifier 1	2.22E-02	6.86E-01
MYL6	myosin light chain 6	2.22E-02	2.60E-01
C2orf68	chromosome 2 open reading frame 68	2.22E-02	-4.39E-01
ATXN7L3	ataxin 7 like 3	2.22E-02	-4.79E-01
CPXM1	carboxypeptidase X, M14 family member 1	2.22E-02	1.95E-01
METTL26	methyltransferase like 26	2.22E-02	2.77E-01

TOP1MT	topoisomerase (DNA) I, mitochondrial	2.23E-02	-5.09E-01
NIN	ninein	2.23E-02	4.04E-01
LARS	leucyl-tRNA synthetase	2.23E-02	5.11E-01
SLC35E2B	solute carrier family 35 member E2B	2.23E-02	-5.27E-01
PLPP4	phospholipid phosphatase 4	2.23E-02	1.98E-01
GTF2IRD2B	GTF2I repeat domain containing 2B	2.24E-02	-3.82E-01
FTCD	formimidoyltransferase cyclodeaminase	2.24E-02	-1.99E-01
MRPS6	mitochondrial ribosomal protein S6	2.24E-02	-1.79E-01
PDHA1	pyruvate dehydrogenase (lipoamide) alpha 1	2.24E-02	2.58E-01
NDST1	N-deacetylase and N-sulfotransferase 1	2.24E-02	-2.79E-01
BAG3	BCL2 associated athanogene 3	2.24E-02	4.06E-01
TAPBPL	TAP binding protein like	2.24E-02	1.87E-01
AGPAT5	1-acylglycerol-3-phosphate O-acyltransferase 5	2.24E-02	4.76E-01
PDHX	pyruvate dehydrogenase complex component X	2.24E-02	4.35E-01
EPHA10	EPH receptor A10	2.25E-02	1.92E-01

DNASE1L1	deoxyribonuclease 1 like 1	2.25E-02	-1.52E-01
ZNF254	zinc finger protein 254	2.25E-02	-3.40E-01
RPPH1	ribonuclease P RNA component H1	2.26E-02	-5.94E-01
FAM172A	family with sequence similarity 172 member A	2.26E-02	-6.26E-01
RBM14	RNA binding motif protein 14	2.26E-02	3.85E-01
FOXP1-IT1	FOXP1 intronic transcript 1	2.27E-02	2.08E-01
DLST	dihydrolipoamide S-succinyltransferase	2.27E-02	2.79E-01
SNURF	SNRPN upstream reading frame	2.27E-02	-6.68E-01
WDR75	WD repeat domain 75	2.27E-02	3.52E-01
H2AFY2	H2A histone family member Y2	2.28E-02	-2.16E-01
ESPN	espin	2.28E-02	1.63E-01
LINC00115	long intergenic non-protein coding RNA 115	2.28E-02	-2.51E-01
PSMA6	proteasome subunit alpha 6	2.28E-02	4.21E-01
GORAB	golgin, RAB6 interacting	2.28E-02	4.45E-01
CYP20A1	cytochrome P450 family 20 subfamily A member 1	2.28E-02	4.01E-01

ROBO2	roundabout guidance receptor 2	2.28E-02	-3.59E-01
WNT8B	Wnt family member 8B	2.28E-02	-1.51E-01
FKBP1A	FK506 binding protein 1A	2.28E-02	-4.47E-01
SDCBP	syndecan binding protein	2.29E-02	7.51E-01
CETN4P	centrin 4, pseudogene	2.29E-02	1.83E-01
CARD14	caspase recruitment domain family member 14	2.29E-02	-4.66E-01
MCMDC2	minichromosome maintenance domain containing 2	2.29E-02	-3.81E-01
WDR3	WD repeat domain 3	2.29E-02	2.29E-01
CEP290	centrosomal protein 290	2.29E-02	6.07E-01
CNDP2	CNDP dipeptidase 2 (metallopeptidase M20 family)	2.29E-02	2.67E-01
G6PC	glucose-6-phosphatase catalytic subunit	2.29E-02	-2.07E-01
PINLYP	phospholipase A2 inhibitor and LY6/PLAUR domain containing	2.29E-02	-1.92E-01
GSTT2B	glutathione S-transferase theta 2B (gene/pseudogene)	2.29E-02	-5.79E-01
APOBEC3H	apolipoprotein B mRNA editing enzyme catalytic subunit 3H	2.29E-02	-1.49E-01

DAXX	death domain associated protein	2.30E-02	2.62E-01
C1QTNF3	C1q and tumor necrosis factor related protein 3	2.30E-02	-2.27E-01
FOXP4	forkhead box P4	2.30E-02	-1.47E-01
SFT2D2	SFT2 domain containing 2	2.30E-02	4.29E-01
TAF4	TATA-box binding protein associated factor 4	2.30E-02	2.97E-01
SNORD49A	small nucleolar RNA, C/D box 49A	2.30E-02	-2.77E-01
CHRNA4	cholinergic receptor nicotinic alpha 4 subunit	2.30E-02	-1.71E-01
SCFD1	sec1 family domain containing 1	2.31E-02	2.31E-01
MIR425	microRNA 425	2.31E-02	-1.85E-01
ZBED3	zinc finger BED-type containing 3	2.31E-02	-2.05E-01
SLC7A11	solute carrier family 7 member 11	2.31E-02	1.73E-01
FLT3LG	fms related tyrosine kinase 3 ligand	2.31E-02	1.78E-01
GTF3C2	general transcription factor IIIC subunit 2	2.31E-02	-5.85E-01
RLIM	ring finger protein, LIM domain interacting	2.31E-02	-2.74E-01

PTPRR	protein tyrosine phosphatase, receptor type R	2.31E-02	-1.70E-01
LURAP1	leucine rich adaptor protein 1	2.31E-02	2.22E-01
HLA-DRB6	major histocompatibility complex, class II, DR beta 6 (pseudogene)	2.31E-02	-2.84E-01
ACLY	ATP citrate lyase	2.32E-02	5.41E-01
ROCK1	Rho associated coiled-coil containing protein kinase 1	2.32E-02	6.81E-01
HNRNPA2B1	heterogeneous nuclear ribonucleoprotein A2/B1	2.32E-02	4.00E-01
HBS1L	HBS1 like translational GTPase	2.32E-02	-1.58E-01
RAC1	ras-related C3 botulinum toxin substrate 1 (rho family, small GTP binding protein Rac1)	2.32E-02	1.87E-01
GGCX	gamma-glutamyl carboxylase	2.32E-02	2.76E-01
MAN2A1	mannosidase alpha class 2A member 1	2.32E-02	7.45E-01
LOC283788	FSHD region gene 1 pseudogene	2.32E-02	3.39E-01
SPG11	spastic paraplegia 11 (autosomal recessive)	2.32E-02	-6.55E-01
HARS2	histidyl-tRNA synthetase 2, mitochondrial	2.33E-02	5.68E-01
SGK1	serum/glucocorticoid regulated kinase 1	2.33E-02	-1.78E-01
ZNF92	zinc finger protein 92	2.33E-02	1.47E-01
TTC1	tetratricopeptide repeat domain 1	2.33E-02	4.65E-01

EPB41	erythrocyte membrane protein band 4.1	2.33E-02	-2.17E-01
SERGEF	secretion regulating guanine nucleotide exchange factor	2.34E-02	-4.45E-01
CAPS2	calcyphosine 2	2.34E-02	-4.16E-01
UHMK1	U2AF homology motif (UHM) kinase 1	2.34E-02	9.23E-01
TTI1	TELO2 interacting protein 1	2.34E-02	4.42E-01
LRRC26	leucine rich repeat containing 26	2.34E-02	2.67E-01
CLCN7	chloride voltage-gated channel 7	2.34E-02	-7.71E-01
SGPP1	sphingosine-1-phosphate phosphatase 1	2.34E-02	1.72E-01
OAS2	2'-5'-oligoadenylate synthetase 2	2.35E-02	1.60E-01
SCN2B	sodium voltage-gated channel beta subunit 2	2.35E-02	1.51E-01
ATP1A1	ATPase Na+/K+ transporting subunit alpha 1	2.35E-02	-5.94E-01
PATL1	PAT1 homolog 1, processing body mRNA decay factor	2.35E-02	3.53E-01
RNVU1-7	RNA, variant U1 small nuclear 7	2.35E-02	-9.44E-01
GPX8	glutathione peroxidase 8 (putative)	2.35E-02	-2.79E-01
KLF4	Kruppel like factor 4	2.35E-02	1.60E-01

GEMIN4	gem nuclear organelle associated protein 4	2.35E-02	5.02E-01
RSL1D1	ribosomal L1 domain containing 1	2.36E-02	2.23E-01
CD7	CD7 molecule	2.36E-02	-2.87E-01
BBS9	Bardet-Biedl syndrome 9	2.36E-02	-2.30E-01
NPAS2	neuronal PAS domain protein 2	2.36E-02	-1.96E-01
LBX2	ladybird homeobox 2	2.36E-02	2.16E-01
ANKRD23	ankyrin repeat domain 23	2.36E-02	2.09E-01
SLC25A52	solute carrier family 25 member 52	2.36E-02	2.15E-01
CARHSP1	calcium regulated heat stable protein 1	2.37E-02	6.94E-01
FOXO4	forkhead box O4	2.37E-02	-2.89E-01
ZNF3	zinc finger protein 3	2.37E-02	-2.12E-01
TOM1L2	target of myb1 like 2 membrane trafficking protein	2.37E-02	2.83E-01
SNRPF	small nuclear ribonucleoprotein polypeptide F	2.37E-02	5.09E-01
MANEA	mannosidase endo-alpha	2.37E-02	4.41E-01
PPM1A	protein phosphatase, Mg2+/Mn2+ dependent 1A	2.37E-02	-4.15E-01

MOB3A	MOB kinase activator 3A	2.38E-02	-3.15E-01
CYB5D1	cytochrome b5 domain containing 1	2.38E-02	-1.92E-01
PLEC	plectin	2.38E-02	1.78E-01
FLAD1	flavin adenine dinucleotide synthetase 1	2.38E-02	5.86E-01
H3F3AP4	H3 histone, family 3A, pseudogene 4	2.38E-02	2.40E-01
FOXR1	forkhead box R1	2.38E-02	2.47E-01
BAMBI	BMP and activin membrane bound inhibitor	2.38E-02	2.48E-01
UROS	uroporphyrinogen III synthase	2.38E-02	-5.72E-01
BAD	BCL2 associated agonist of cell death	2.38E-02	-4.56E-01
CCL5	C-C motif chemokine ligand 5	2.38E-02	3.32E-01
ULK1	unc-51 like autophagy activating kinase 1	2.39E-02	-4.47E-01
NUDT2	nudix hydrolase 2	2.39E-02	-5.94E-01
MED1	mediator complex subunit 1	2.39E-02	-4.91E-01
MAZ	MYC associated zinc finger protein	2.39E-02	1.90E-01
MB	myoglobin	2.39E-02	1.67E-01

ZBTB9	zinc finger and BTB domain containing 9	2.39E-02	4.63E-01
RPP21	ribonuclease P/MRP subunit p21	2.40E-02	6.65E-01
ZSWIM8	zinc finger SWIM-type containing 8	2.40E-02	-6.63E-01
MTNR1A	melatonin receptor 1A	2.40E-02	-1.67E-01
KCNK10	potassium two pore domain channel subfamily K member 10	2.40E-02	1.62E-01
CA12	carbonic anhydrase 12	2.40E-02	-4.66E-01
PPFIA1	PTPRF interacting protein alpha 1	2.40E-02	4.54E-01
MIR98	microRNA 98	2.40E-02	-4.94E-01
UBXN4	UBX domain protein 4	2.40E-02	2.32E-01
CHKB-AS1	CHKB antisense RNA 1 (head to head)	2.41E-02	2.74E-01
PPP2R5D	protein phosphatase 2 regulatory subunit B'delta	2.41E-02	6.07E-01
SLC18B1	solute carrier family 18 member B1	2.41E-02	2.98E-01
AMHR2	anti-Mullerian hormone receptor type 2	2.41E-02	2.07E-01
MRPL13	mitochondrial ribosomal protein L13	2.42E-02	3.84E-01
TIPRL	TOR signaling pathway regulator	2.42E-02	4.34E-01
ZNF473	zinc finger protein 473	2.42E-02	2.29E-01

HIST1H2AM	histone cluster 1, H2am	2.42E-02	-2.85E-01
LUC7L2	LUC7 like 2, pre-mRNA splicing factor	2.42E-02	-1.98E-01
HAS1	hyaluronan synthase 1	2.42E-02	-1.77E-01
SNAI3-AS1	SNAI3 antisense RNA 1	2.42E-02	-2.26E-01
SETD1B	SET domain containing 1B	2.43E-02	1.77E-01
GAR1	GAR1 ribonucleoprotein	2.44E-02	7.23E-01
NECAB3	N-terminal EF-hand calcium binding protein 3	2.44E-02	-3.75E-01
TADA1	transcriptional adaptor 1	2.44E-02	2.05E-01
TPM4	tropomyosin 4	2.44E-02	3.01E-01
MROH1	maestro heat like repeat family member 1	2.44E-02	1.70E-01
NDOR1	NADPH dependent diflavin oxidoreductase 1	2.44E-02	1.91E-01
ATP5D	ATP synthase, H+ transporting, mitochondrial F1 complex, delta subunit	2.44E-02	-6.03E-01
NOXA1	NADPH oxidase activator 1	2.44E-02	2.05E-01
ARRB2	arrestin beta 2	2.44E-02	3.90E-01
TRIM49D1	tripartite motif containing 49D1	2.44E-02	-2.35E-01

CHMP2A	charged multivesicular body protein 2A	2.44E-02	-7.94E-01
AKR7A3	aldo-keto reductase family 7 member A3	2.44E-02	1.71E-01
LARP1B	La ribonucleoprotein domain family member 1B	2.45E-02	2.15E-01
RBBP5	RB binding protein 5, histone lysine methyltransferase complex subunit	2.45E-02	-3.39E-01
ERICH1	glutamate rich 1	2.45E-02	2.77E-01
HEBP2	heme binding protein 2	2.45E-02	3.39E-01
MMP20	matrix metallopeptidase 20	2.45E-02	-1.90E-01
SYNC	syncoilin, intermediate filament protein	2.45E-02	-4.30E-01
LAMB2P1	laminin subunit beta 2 pseudogene 1	2.46E-02	-1.50E-01
ARMC10	armadillo repeat containing 10	2.46E-02	2.60E-01
CBWD5	COBW domain containing 5	2.46E-02	4.05E-01
ZNF257	zinc finger protein 257	2.46E-02	1.73E-01
SH3TC1	SH3 domain and tetratricopeptide repeats 1	2.46E-02	-1.73E-01
ZFC3H1	zinc finger C3H1-type containing	2.46E-02	-5.93E-01
SIRT5	sirtuin 5	2.46E-02	-1.94E-01

ZNF287	zinc finger protein 287	2.46E-02	1.97E-01
LIMS1	LIM zinc finger domain containing 1	2.46E-02	6.82E-01
SLC1A4	solute carrier family 1 member 4	2.47E-02	2.11E-01
SMG1	SMG1, nonsense mediated mRNA decay associated PI3K related kinase	2.47E-02	4.30E-01
LINC00908	long intergenic non-protein coding RNA 908	2.47E-02	1.64E-01
CHGA	chromogranin A	2.47E-02	2.28E-01
ASAP2	ArfGAP with SH3 domain, ankyrin repeat and PH domain 2	2.47E-02	3.16E-01
OXCT2	3-oxoacid CoA-transferase 2	2.48E-02	2.26E-01
HPS1	HPS1, biogenesis of lysosomal organelles complex 3 subunit 1	2.48E-02	4.01E-01
SEZ6L2	seizure related 6 homolog like 2	2.48E-02	-6.69E-01
COLQ	collagen like tail subunit of asymmetric acetylcholinesterase	2.48E-02	-2.50E-01
SCO2	SCO2 cytochrome c oxidase assembly protein	2.48E-02	-4.56E-01
PYGO1	pygopus family PHD finger 1	2.48E-02	1.80E-01
ANK3	ankyrin 3, node of Ranvier (ankyrin G)	2.48E-02	-5.43E-01
CT45A5	cancer/testis antigen family 45, member A5	2.48E-02	2.46E-01
PLEKHA1	pleckstrin homology domain containing A1	2.49E-02	-2.78E-01

AFTPH	aftiphilin	2.49E-02	2.12E<br>-01
LSM4	LSM4 homolog, U6 small nuclear RNA and mRNA degradation associated	2.49E-02	5.78E<br>-01
RPRML	reprimo like	2.49E-02	-<br>1.56E<br>-01
RMDN3	regulator of microtubule dynamics 3	2.49E-02	2.71E<br>-01
SPATS2	spermatogenesis associated serine rich 2	2.49E-02	-<br>4.65E<br>-01
MBNL1	muscleblind like splicing regulator 1	2.49E-02	9.40E<br>-01
TEX261	testis expressed 261	2.49E-02	3.29E<br>-01
SRM	spermidine synthase	2.50E-02	4.34E<br>-01
IL17RD	interleukin 17 receptor D	2.50E-02	-<br>4.97E<br>-01
GTF2IRD1	GTF2I repeat domain containing 1	2.50E-02	3.11E<br>-01
ROGDI	rogdi homolog	2.50E-02	-<br>2.28E<br>-01
FOXK1	forkhead box K1	2.50E-02	2.63E<br>-01
CASP7	caspase 7	2.50E-02	3.37E<br>-01
LOC105371506	uncharacterized LOC105371506	2.50E-02	-<br>1.80E<br>-01
TMEM190	transmembrane protein 190	2.50E-02	-<br>3.35E<br>-01

CYP39A1	cytochrome P450 family 39 subfamily A member 1	2.50E-02	-1.70E-01
RPS4X	ribosomal protein S4, X-linked	2.50E-02	-2.93E-01
HSPD1	heat shock protein family D (Hsp60) member 1	2.51E-02	5.98E-01
HIF1AN	hypoxia inducible factor 1 alpha subunit inhibitor	2.51E-02	2.93E-01
CRNKL1	crooked neck pre-mRNA splicing factor 1	2.51E-02	3.12E-01
SULT1C3	sulfotransferase family 1C member 3	2.51E-02	1.84E-01
DBF4	DBF4 zinc finger	2.51E-02	2.01E-01
MT3	metallothionein 3	2.51E-02	1.67E-01
NXNL1	nucleoredoxin-like 1	2.51E-02	-2.66E-01
ABCA10	ATP binding cassette subfamily A member 10	2.51E-02	1.94E-01
GPD1L	glycerol-3-phosphate dehydrogenase 1-like	2.51E-02	-2.45E-01
WAC	WW domain containing adaptor with coiled-coil	2.52E-02	4.22E-01
GOLGA8B	golgin A8 family member B	2.52E-02	-9.19E-01
FCHO1	FCH domain only 1	2.53E-02	-3.34E-01
NET1	neuroepithelial cell transforming 1	2.53E-02	-2.01E-01

GET4	golgi to ER traffic protein 4	2.53E-02	-2.18E-01
BRK1	BRICK1, SCAR/WAVE actin nucleating complex subunit	2.54E-02	-4.58E-01
ZPLD1	zona pellucida like domain containing 1	2.54E-02	-2.80E-01
TBX21	T-box 21	2.54E-02	-3.52E-01
NUDT5	nudix hydrolase 5	2.54E-02	2.77E-01
ZNF197	zinc finger protein 197	2.54E-02	3.25E-01
GK	glycerol kinase	2.54E-02	2.87E-01
MYL9	myosin light chain 9	2.54E-02	5.45E-01
TRIML1	tripartite motif family like 1	2.55E-02	-1.59E-01
ATP6V1B2	ATPase H+ transporting V1 subunit B2	2.55E-02	4.08E-01
CAAP1	caspase activity and apoptosis inhibitor 1	2.55E-02	1.72E-01
CAPN12	calpain 12	2.55E-02	-1.66E-01
TRIM71	tripartite motif containing 71	2.55E-02	-3.99E-01
MORF4L1	mortality factor 4 like 1	2.55E-02	3.03E-01
HIPK1	homeodomain interacting protein kinase 1	2.55E-02	1.81E-01

FAM189B	family with sequence similarity 189 member B	2.55E-02	-3.80E-01
ARHGAP15	Rho GTPase activating protein 15	2.55E-02	3.60E-01
WARS2	tryptophanyl tRNA synthetase 2, mitochondrial	2.56E-02	3.18E-01
KRT39	keratin 39	2.56E-02	1.75E-01
ZNF189	zinc finger protein 189	2.56E-02	5.30E-01
PNKD	paroxysmal nonkinesigenic dyskinesia	2.57E-02	-3.00E-01
DDC	dopa decarboxylase	2.57E-02	1.66E-01
NDUFA13	NADH:ubiquinone oxidoreductase subunit A13	2.57E-02	-5.63E-01
ST6GALNAC2	ST6 N-acetylgalactosaminide alpha-2,6-sialyltransferase 2	2.57E-02	1.61E-01
SNORD36A	small nucleolar RNA, C/D box 36A	2.57E-02	3.69E-01
CEP63	centrosomal protein 63	2.57E-02	4.90E-01
TEX14	testis expressed 14, intercellular bridge forming factor	2.57E-02	2.32E-01
MMGT1	membrane magnesium transporter 1	2.57E-02	3.91E-01
PALLD	palladin, cytoskeletal associated protein	2.58E-02	5.37E-01
PTPMT1	protein tyrosine phosphatase, mitochondrial 1	2.58E-02	4.29E-01
EEF1B2	eukaryotic translation elongation factor 1 beta 2	2.58E-02	-4.44E-01

PRTG	protogenin	2.58E-02	-2.02E-01
PDCD2	programmed cell death 2	2.58E-02	3.60E-01
NAP1L1	nucleosome assembly protein 1 like 1	2.58E-02	-3.39E-01
MIGA1	mitoguardin 1	2.58E-02	-5.34E-01
DHX29	DEAH-box helicase 29	2.58E-02	8.24E-01
SEC23B	Sec23 homolog B, coat complex II component	2.58E-02	3.58E-01
IPO7	importin 7	2.58E-02	1.15
ZMYM5	zinc finger MYM-type containing 5	2.58E-02	1.72E-01
KIR2DL1	killer cell immunoglobulin like receptor, two Ig domains and long cytoplasmic tail 1	2.58E-02	1.72E-01
GNB4	G protein subunit beta 4	2.59E-02	-3.80E-01
TROVE2	TROVE domain family member 2	2.59E-02	-5.45E-01
NME1	NME/NM23 nucleoside diphosphate kinase 1	2.59E-02	-1.83E-01
CIDECP	cell death-inducing DFFA-like effector c pseudogene	2.59E-02	-5.87E-01
RTN3	reticulon 3	2.59E-02	4.54E-01
FNBP1	formin binding protein 1	2.59E-02	-4.22E-01

PODXL2	podocalyxin like 2	2.59E-02	-3.61E-01
SULT1A3	sulfotransferase family 1A member 3	2.59E-02	-1.98E-01
SMAP1	small ArfGAP 1	2.59E-02	-2.50E-01
ERP29	endoplasmic reticulum protein 29	2.59E-02	-8.97E-01
CNTLN	centlein	2.59E-02	2.46E-01
MAGEA9B	MAGE family member A9B	2.59E-02	1.90E-01
RECQL5	RecQ like helicase 5	2.59E-02	1.58E-01
CNPY4	canopy FGF signaling regulator 4	2.60E-02	-2.64E-01
ANKRD28	ankyrin repeat domain 28	2.60E-02	-2.02E-01
MED13	mediator complex subunit 13	2.60E-02	-2.24E-01
LINC00515	long intergenic non-protein coding RNA 515	2.60E-02	-1.81E-01
PTGS2	prostaglandin-endoperoxide synthase 2	2.60E-02	-3.65E-01
KLK10	kallikrein related peptidase 10	2.60E-02	2.38E-01
HEATR5B	HEAT repeat containing 5B	2.60E-02	-7.18E-01

GALNT3	polypeptide N-acetylgalactosaminyltransferase 3	2.60E-02	-3.88E-01
METTL21A	methyltransferase like 21A	2.60E-02	-2.38E-01
CBX3	chromobox 3	2.61E-02	5.53E-01
ENHO	energy homeostasis associated	2.61E-02	-2.95E-01
PUS1	pseudouridylate synthase 1	2.61E-02	1.58E-01
ATP5D	ATP synthase, H+ transporting, mitochondrial F1 complex, delta subunit	2.61E-02	-2.91E-01
ATP6V0A1	ATPase H+ transporting V0 subunit a1	2.61E-02	-5.10E-01
TARS	threonyl-tRNA synthetase	2.61E-02	5.61E-01
PMS2CL	PMS2 C-terminal like pseudogene	2.61E-02	3.80E-01
HHATL	hedgehog acyltransferase-like	2.61E-02	-3.45E-01
ST7	suppression of tumorigenicity 7	2.61E-02	1.74E-01
ATP8B2	ATPase phospholipid transporting 8B2	2.61E-02	-1.84E-01
PTPA	protein phosphatase 2 phosphatase activator	2.61E-02	-2.20E-01
HSPA13	heat shock protein family A (Hsp70) member 13	2.62E-02	6.07E-01

GGA3	golgi associated, gamma adaptin ear containing, ARF binding protein 3	2.62E-02	-3.59E-01
ZNF790	zinc finger protein 790	2.62E-02	2.06E-01
IFT43	intraflagellar transport 43	2.63E-02	-4.34E-01
LRRC37A4P	leucine rich repeat containing 37 member A4, pseudogene	2.63E-02	-2.43E-01
TAZ	tafazzin	2.63E-02	-1.61E-01
GAPDHS	glyceraldehyde-3-phosphate dehydrogenase, spermatogenic	2.63E-02	-1.98E-01
PCNA	proliferating cell nuclear antigen	2.63E-02	2.89E-01
MFAP3L	microfibrillar associated protein 3 like	2.63E-02	1.57E-01
MED23	mediator complex subunit 23	2.64E-02	-1.81E-01
PHLDB1	pleckstrin homology like domain family B member 1	2.64E-02	-5.40E-01
ENY2	ENY2, transcription and export complex 2 subunit	2.64E-02	2.19E-01
MEA1	male-enhanced antigen 1	2.64E-02	5.84E-01
RWDD4	RWD domain containing 4	2.64E-02	1.46E-01
NTMT1	N-terminal Xaa-Pro-Lys N-methyltransferase 1	2.65E-02	5.52E-01
LRCH4	leucine rich repeats and calponin homology domain containing 4	2.65E-02	-2.92E-01

ID4	inhibitor of DNA binding 4, HLH protein	2.65E-02	5.70E-01
CSNK1A1	casein kinase 1 alpha 1	2.65E-02	2.35E-01
RAB11FIP2	RAB11 family interacting protein 2	2.66E-02	-3.41E-01
SPTLC2	serine palmitoyltransferase long chain base subunit 2	2.66E-02	-1.69E-01
MAPKAPK3	mitogen-activated protein kinase-activated protein kinase 3	2.66E-02	-4.69E-01
SERPINA10	serpin family A member 10	2.66E-02	1.88E-01
CHRNA5	cholinergic receptor nicotinic alpha 5 subunit	2.66E-02	-6.26E-01
PKM	pyruvate kinase, muscle	2.66E-02	-5.34E-01
ZNF813	zinc finger protein 813	2.66E-02	-2.51E-01
ZNF577	zinc finger protein 577	2.66E-02	-5.02E-01
KANK4	KN motif and ankyrin repeat domains 4	2.66E-02	-1.69E-01
CHMP4A	charged multivesicular body protein 4A	2.66E-02	-4.26E-01
AHNAK	AHNAK nucleoprotein	2.67E-02	-3.10E-01
BZW1	basic leucine zipper and W2 domains 1	2.67E-02	3.02E-01

WDR5	WD repeat domain 5	2.67E-02	-1.99E-01
STARD3NL	STARD3 N-terminal like	2.67E-02	3.96E-01
RPLP0	ribosomal protein lateral stalk subunit P0	2.67E-02	-3.52E-01
TMEM116	transmembrane protein 116	2.67E-02	3.73E-01
TSN	translin	2.67E-02	4.21E-01
S100A13	S100 calcium binding protein A13	2.67E-02	-1.63E-01
SASS6	SAS-6 centriolar assembly protein	2.68E-02	1.69E-01
DDX39A	DEAD-box helicase 39A	2.68E-02	2.11E-01
STK19	serine/threonine kinase 19	2.68E-02	-5.01E-01
TMSB15B	thymosin beta 15B	2.68E-02	2.03E-01
RHOF	ras homolog family member F, filopodia associated	2.68E-02	-2.31E-01
PYDC1	pyrin domain containing 1	2.68E-02	1.70E-01
CDKN2C	cyclin dependent kinase inhibitor 2C	2.68E-02	2.07E-01
POLD2	DAN polymerase delta 2, accessory subunit	2.68E-02	3.55E-01
RDH5	retinol dehydrogenase 5	2.69E-02	1.61E-01

PCBP2	poly(rC) binding protein 2	2.69E-02	-2.24E-01
RNF126	ring finger protein 126	2.69E-02	-2.40E-01
INO80	INO80 complex subunit	2.69E-02	4.02E-01
MPG	N-methylpurine DNA glycosylase	2.69E-02	-1.88E-01
ZDHHC13	zinc finger DHHC-type containing 13	2.70E-02	4.41E-01
SDF2L1	stromal cell derived factor 2 like 1	2.70E-02	-6.97E-01
NPEPPS	aminopeptidase puromycin sensitive	2.70E-02	-3.38E-01
GCNT2	glucosaminyl (N-acetyl) transferase 2, I-branching enzyme (I blood group)	2.70E-02	2.53E-01
LRRC8B	leucine rich repeat containing 8 family member B	2.71E-02	2.11E-01
OPA3	optic atrophy 3 (autosomal recessive, with chorea and spastic paraplegia)	2.71E-02	-1.56E-01
SHPRH	SNF2 histone linker PHD RING helicase	2.71E-02	-3.17E-01
CCS	copper chaperone for superoxide dismutase	2.71E-02	-6.21E-01
ANXA9	annexin A9	2.71E-02	-1.63E-01
PAQR6	progestin and adipoQ receptor family member 6	2.71E-02	-2.08E-01

FARS2	phenylalanyl-tRNA synthetase 2, mitochondrial	2.71E-02	2.99E-01
DNAJC17	DnaJ heat shock protein family (Hsp40) member C17	2.71E-02	3.63E-01
CD84	CD84 molecule	2.72E-02	-1.90E-01
C16orf62	chromosome 16 open reading frame 62	2.72E-02	-2.85E-01
PGP	phosphoglycolate phosphatase	2.72E-02	2.48E-01
TRIQK	triple QxxK/R motif containing	2.72E-02	-4.32E-01
HES3	hes family bHLH transcription factor 3	2.72E-02	-1.85E-01
KIRREL2	kin of IRRE like 2 (Drosophila)	2.72E-02	-1.78E-01
CAPRIN1	cell cycle associated protein 1	2.73E-02	4.76E-01
RERE	arginine-glutamic acid dipeptide repeats	2.73E-02	-5.69E-01
SOCS2	suppressor of cytokine signaling 2	2.73E-02	1.97E-01
CYB561	cytochrome b561	2.73E-02	3.85E-01
ACAT1	acetyl-CoA acetyltransferase 1	2.73E-02	-1.96E-01
PLAC8	placenta specific 8	2.73E-02	3.13E-01
ATP6V1H	ATPase H+ transporting V1 subunit H	2.73E-02	-1.51E-01

PRRT3	proline rich transmembrane protein 3	2.73E-02	5.01E-01
ABAT	4-aminobutyrate aminotransferase	2.73E-02	-2.94E-01
ZNF585B	zinc finger protein 585B	2.73E-02	-1.69E-01
TNFRSF19	TNF receptor superfamily member 19	2.73E-02	1.94E-01
COX14	COX14, cytochrome c oxidase assembly factor	2.73E-02	-7.03E-01
PHC3	polyhomeotic homolog 3	2.74E-02	2.26E-01
ATP5S	ATP synthase, H+ transporting, mitochondrial Fo complex subunit s (factor B)	2.74E-02	-4.95E-01
NINL	ninein like	2.74E-02	-3.03E-01
NDUFA2	NADH:ubiquinone oxidoreductase subunit A2	2.74E-02	-3.11E-01
MIR299	microRNA 299	2.74E-02	1.58E-01
TBC1D3B	TBC1 domain family member 3B	2.74E-02	-2.32E-01
CWC15	CWC15 spliceosome-associated protein	2.74E-02	3.34E-01
SERF1B	small EDRK-rich factor 1B	2.74E-02	-1.67E-01
ZFYVE1	zinc finger FYVE-type containing 1	2.74E-02	-5.65E-01

FSD1	fibronectin type III and SPRY domain containing 1	2.74E-02	3.72E-01
WNK3	WNK lysine deficient protein kinase 3	2.74E-02	-2.97E-01
DPH3	diphthamide biosynthesis 3	2.74E-02	3.99E-01
TSPAN33	tetraspanin 33	2.74E-02	4.26E-01
RAE1	ribonucleic acid export 1	2.74E-02	2.16E-01
DENND5B	DENN domain containing 5B	2.75E-02	-4.06E-01
C15orf41	chromosome 15 open reading frame 41	2.75E-02	3.86E-01
SYN2	synapsin II	2.75E-02	1.95E-01
SKP1	S-phase kinase-associated protein 1	2.75E-02	-2.29E-01
GABRB3	gamma-aminobutyric acid type A receptor beta3 subunit	2.75E-02	-3.45E-01
ATP5I	ATP synthase, H+ transporting, mitochondrial Fo complex subunit E	2.75E-02	3.13E-01
TSC22D3	TSC22 domain family member 3	2.75E-02	-3.01E-01
TSC1	tuberous sclerosis 1	2.75E-02	-4.18E-01
METTL21A	methyltransferase like 21A	2.75E-02	-1.86E-01
XAF1	XIAP associated factor 1	2.75E-02	-4.22E-01

TFDP2	transcription factor Dp-2	2.75E-02	-3.08E-01
GSTM4	glutathione S-transferase mu 4	2.76E-02	-2.47E-01
SLC44A1	solute carrier family 44 member 1	2.76E-02	-2.24E-01
NAIF1	nuclear apoptosis inducing factor 1	2.76E-02	-5.63E-01
C2orf47	chromosome 2 open reading frame 47	2.76E-02	2.35E-01
UBTF	upstream binding transcription factor, RNA polymerase I	2.76E-02	2.76E-01
TMPPE	transmembrane protein with metallophosphoesterase domain	2.77E-02	1.79E-01
CIAPIN1	cytokine induced apoptosis inhibitor 1	2.77E-02	4.14E-01
WAS	Wiskott-Aldrich syndrome	2.77E-02	-2.05E-01
SCAND1	SCAN domain containing 1	2.77E-02	-6.92E-01
IFNK	interferon kappa	2.77E-02	1.61E-01
KMT5C	lysine methyltransferase 5C	2.77E-02	-2.07E-01
VAMP3	vesicle associated membrane protein 3	2.77E-02	4.22E-01
PIH1D2	PIH1 domain containing 2	2.78E-02	2.32E-01
NBPF1	neuroblastoma breakpoint family member 1	2.78E-02	-5.70E-01

KPNA2	karyopherin subunit alpha 2	2.78E-02	4.71E-01
WDR45B	WD repeat domain 45B	2.78E-02	-3.97E-01
ATP6V1C1	ATPase H+ transporting V1 subunit C1	2.78E-02	-5.89E-01
IL15RA	interleukin 15 receptor subunit alpha	2.78E-02	2.04E-01
RRM2B	ribonucleotide reductase regulatory TP53 inducible subunit M2B	2.78E-02	-7.23E-01
TAX1BP3	Tax1 binding protein 3	2.78E-02	-4.96E-01
CASK	calcium/calmodulin dependent serine protein kinase	2.78E-02	-6.06E-01
KNOP1	lysine rich nucleolar protein 1	2.79E-02	4.23E-01
SMG8	SMG8, nonsense mediated mRNA decay factor	2.79E-02	-4.60E-01
HLA-DRB4	major histocompatibility complex, class II, DR beta 4	2.79E-02	-2.14E-01
FUT4	fucosyltransferase 4	2.79E-02	2.24E-01
SRCIN1	SRC kinase signaling inhibitor 1	2.79E-02	-2.47E-01
KRBA1	KRAB-A domain containing 1	2.79E-02	-2.05E-01
TERF2IP	TERF2 interacting protein	2.79E-02	-3.08E-01

AKR1B1	aldo-keto reductase family 1 member B	2.80E-02	1.91E-01
UBE4B	ubiquitination factor E4B	2.80E-02	-4.66E-01
C19orf68	chromosome 19 open reading frame 68	2.80E-02	-2.83E-01
MTERF2	mitochondrial transcription termination factor 2	2.80E-02	2.00E-01
GARNL3	GTPase activating Rap/RanGAP domain like 3	2.80E-02	-2.22E-01
CC2D1B	coiled-coil and C2 domain containing 1B	2.80E-02	-1.62E-01
LOC100130298	hCG1816373-like	2.81E-02	1.71E-01
PGA3	pepsinogen 3, group I (pepsinogen A)	2.81E-02	-1.55E-01
SNORA5A	small nucleolar RNA, H/ACA box 5A	2.81E-02	1.65E-01
ZNF808	zinc finger protein 808	2.81E-02	1.71E-01
UBC	ubiquitin C	2.81E-02	2.08E-01
SLC50A1	solute carrier family 50 member 1	2.81E-02	-5.20E-01
TECPR1	tectonin beta-propeller repeat containing 1	2.82E-02	-4.49E-01
EBLN2	endogenous Bornavirus-like nucleoprotein 2	2.82E-02	-1.90E-01

PLXDC2	plexin domain containing 2	2.82E-02	-2.41E-01
IFNB1	interferon beta 1	2.82E-02	1.76E-01
GAPDH	glyceraldehyde-3-phosphate dehydrogenase	2.82E-02	1.60E-01
SCAND1	SCAN domain containing 1	2.82E-02	-7.00E-01
HECTD4	HECT domain E3 ubiquitin protein ligase 4	2.82E-02	-5.64E-01
NOC2L	NOC2 like nucleolar associated transcriptional repressor	2.83E-02	3.57E-01
CATSPER2P1	cation channel sperm associated 2 pseudogene 1	2.83E-02	-4.14E-01
FKBP14	FK506 binding protein 14	2.83E-02	-3.09E-01
VCPIP1	valosin containing protein interacting protein 1	2.83E-02	-2.72E-01
XKR6	XK related 6	2.83E-02	-2.48E-01
TLR3	toll like receptor 3	2.83E-02	2.73E-01
IGSF11	immunoglobulin superfamily member 11	2.83E-02	-1.88E-01
KRTAP3-2	keratin associated protein 3-2	2.83E-02	1.53E-01
MTHFD1L	methylenetetrahydrofolate dehydrogenase (NADP+ dependent) 1-like	2.83E-02	6.76E-01

AQP12B	aquaporin 12B	2.83E-02	-3.07E-01
HSPA12A	heat shock protein family A (Hsp70) member 12A	2.83E-02	1.68E-01
EEF2K	eukaryotic elongation factor 2 kinase	2.83E-02	-3.45E-01
SLX4	SLX4 structure-specific endonuclease subunit	2.85E-02	3.75E-01
OSBPL8	oxysterol binding protein like 8	2.85E-02	5.36E-01
SRSF1	serine and arginine rich splicing factor 1	2.85E-02	3.40E-01
ABL2	ABL proto-oncogene 2, non-receptor tyrosine kinase	2.85E-02	2.46E-01
KMT5B	lysine methyltransferase 5B	2.85E-02	3.22E-01
RGP1	RGP1 homolog, RAB6A GEF complex partner 1	2.86E-02	1.94E-01
XBP1	X-box binding protein 1	2.86E-02	-3.30E-01
UBQLN3	ubiquilin 3	2.86E-02	-1.44E-01
OR4N2	olfactory receptor family 4 subfamily N member 2	2.86E-02	-1.52E-01
PLA2G16	phospholipase A2 group XVI	2.87E-02	1.46E-01
FUT6	fucosyltransferase 6	2.87E-02	-4.69E-01
PLOD2	procollagen-lysine,2-oxoglutarate 5-dioxygenase 2	2.87E-02	-3.56E-01

PDCD7	programmed cell death 7	2.87E-02	3.05E-01
LGSN	lengsin, lens protein with glutamine synthetase domain	2.87E-02	1.67E-01
OR6F1	olfactory receptor family 6 subfamily F member 1	2.87E-02	-1.44E-01
RAB24	RAB24, member RAS oncogene family	2.87E-02	-3.14E-01
PCDHB2	protocadherin beta 2	2.87E-02	-2.56E-01
GSTZ1	glutathione S-transferase zeta 1	2.87E-02	-5.40E-01
GART	phosphoribosylglycinamide formyltransferase, phosphoribosylglycinamide synthetase, phosphoribosylaminoimidazole synthetase	2.87E-02	5.56E-01
ARMCX5	armadillo repeat containing, X-linked 5	2.87E-02	7.32E-01
PAGE2B	PAGE family member 2B	2.88E-02	1.74E-01
PHYKPL	5-phosphohydroxy-L-lysine phospho-lyase	2.88E-02	-2.29E-01
MRPL39	mitochondrial ribosomal protein L39	2.88E-02	-2.50E-01
PRPF4B	pre-mRNA processing factor 4B	2.88E-02	-3.77E-01
RHBDL2	rhomboid like 2	2.88E-02	-4.41E-01
DHX34	DEAH-box helicase 34	2.89E-02	3.87E-01

RPL29P2	ribosomal protein L29 pseudogene 2	2.89E-02	-4.61E-01
MALL	mal, T-cell differentiation protein like	2.89E-02	1.40E-01
SNORD113-3	small nucleolar RNA, C/D box 113-3	2.89E-02	-1.47E-01
RGS3	regulator of G-protein signaling 3	2.89E-02	-2.91E-01
KIF25	kinesin family member 25	2.89E-02	-1.63E-01
ING1	inhibitor of growth family member 1	2.89E-02	-3.47E-01
PLAC8	placenta specific 8	2.89E-02	4.53E-01
PHF5A	PHD finger protein 5A	2.89E-02	6.71E-01
CRCP	CGRP receptor component	2.90E-02	-2.60E-01
APOC1	apolipoprotein C1	2.90E-02	-5.00E-01
TMEM50A	transmembrane protein 50A	2.90E-02	1.83E-01
BRI3BP	BRI3 binding protein	2.90E-02	2.60E-01
KIAA0907	KIAA0907	2.91E-02	-2.83E-01
C10orf12	chromosome 10 open reading frame 12	2.91E-02	2.09E-01

SHC1	SHC adaptor protein 1	2.91E-02	-2.35E-01
MRPL15	mitochondrial ribosomal protein L15	2.91E-02	2.09E-01
SLC35F2	solute carrier family 35 member F2	2.92E-02	2.69E-01
ZNF25	zinc finger protein 25	2.92E-02	-2.36E-01
CLOCK	clock circadian regulator	2.92E-02	-9.12E-01
NNMT	nicotinamide N-methyltransferase	2.92E-02	-1.43E-01
GRK2	G protein-coupled receptor kinase 2	2.92E-02	5.04E-01
SUCNR1	succinate receptor 1	2.92E-02	-2.00E-01
STX17	syntaxin 17	2.92E-02	1.85E-01
HNRNPC	heterogeneous nuclear ribonucleoprotein C (C1/C2)	2.92E-02	1.47E-01
SNORD91B	small nucleolar RNA, C/D box 91B	2.92E-02	1.96E-01
ZNF77	zinc finger protein 77	2.93E-02	2.18E-01
FANCC	Fanconi anemia complementation group C	2.93E-02	2.60E-01
CRY1	cryptochrome circadian clock 1	2.93E-02	5.69E-01
PLAC8L1	PLAC8 like 1	2.93E-02	-1.44E-01

DNAJA3	DnaJ heat shock protein family (Hsp40) member A3	2.93E-02	-2.80E-01
AGA	aspartylglucosaminidase	2.93E-02	-1.90E-01
CHST6	carbohydrate sulfotransferase 6	2.94E-02	-1.84E-01
PICK1	protein interacting with PRKCA 1	2.94E-02	-5.25E-01
TMEM154	transmembrane protein 154	2.94E-02	1.54E-01
ADCY3	adenylate cyclase 3	2.94E-02	-4.40E-01
PMS1	PMS1 homolog 1, mismatch repair system component	2.94E-02	5.20E-01
PIGP	phosphatidylinositol glycan anchor biosynthesis class P	2.94E-02	-6.09E-01
PSTPIP1	proline-serine-threonine phosphatase interacting protein 1	2.94E-02	-2.30E-01
LINC01300	long intergenic non-protein coding RNA 1300	2.94E-02	-3.82E-01
PCDHB19P	protocadherin beta 19 pseudogene	2.95E-02	-4.46E-01
UBL7	ubiquitin like 7	2.95E-02	-3.10E-01
TGFBR3L	transforming growth factor beta receptor 3 like	2.95E-02	1.65E-01
CCR2	C-C motif chemokine receptor 2	2.95E-02	1.61E-01

SEC23A	Sec23 homolog A, coat complex II component	2.95E-02	5.55E-01
BOLA2	bolA family member 2	2.95E-02	-6.06E-01
C8orf88	chromosome 8 open reading frame 88	2.96E-02	1.70E-01
PREPL	prolyl endopeptidase-like	2.96E-02	-1.01
RBM41	RNA binding motif protein 41	2.96E-02	2.02E-01
MAP4K4	mitogen-activated protein kinase kinase kinase kinase 4	2.96E-02	-5.28E-01
SSX1	SSX family member 1	2.96E-02	-1.66E-01
CLNS1A	chloride nucleotide-sensitive channel 1A	2.96E-02	-1.94E-01
WDR6	WD repeat domain 6	2.96E-02	-3.44E-01
RALYL	RALY RNA binding protein-like	2.96E-02	-2.07E-01
ETV4	ETS variant 4	2.96E-02	3.85E-01
NUDT21	nudix hydrolase 21	2.96E-02	5.46E-01
LEMD3	LEM domain containing 3	2.96E-02	-5.62E-01
TRAP1	TNF receptor associated protein 1	2.97E-02	2.09E-01
RNU1-1	RNA, U1 small nuclear 1	2.97E-02	-7.01E-01

ADAT2	adenosine deaminase, tRNA specific 2	2.97E-02	-1.93E-01
LINC01003	long intergenic non-protein coding RNA 1003	2.97E-02	-2.19E-01
FKBP15	FK506 binding protein 15	2.98E-02	3.13E-01
G6PC2	glucose-6-phosphatase catalytic subunit 2	2.98E-02	1.49E-01
ANKRD36B	ankyrin repeat domain 36B	2.98E-02	-3.89E-01
COPG2	coatomer protein complex subunit gamma 2	2.98E-02	2.56E-01
BET1	Bet1 golgi vesicular membrane trafficking protein	2.98E-02	3.87E-01
BTF3	basic transcription factor 3	2.99E-02	-5.70E-01
VPS72	vacuolar protein sorting 72 homolog	2.99E-02	3.35E-01
GALE	UDP-galactose-4-epimerase	2.99E-02	4.13E-01
ILK	integrin linked kinase	2.99E-02	-1.47E-01
DALRD3	DALR anticodon binding domain containing 3	2.99E-02	-1.90E-01
SNN	stannin	2.99E-02	-2.58E-01
USP53	ubiquitin specific peptidase 53	3.00E-02	5.55E-01
RMND5A	required for meiotic nuclear division 5 homolog A	3.00E-02	2.32E-01

SLC23A2	solute carrier family 23 member 2	3.00E-02	-3.50E-01
SLC25A1	solute carrier family 25 member 1	3.00E-02	-4.89E-01
INAFM1	InaF motif containing 1	3.00E-02	5.50E-01
PDGFA	platelet derived growth factor subunit A	3.00E-02	-1.82E-01
ARHGDIG	Rho GDP dissociation inhibitor gamma	3.00E-02	-2.91E-01
SLCO3A1	solute carrier organic anion transporter family member 3A1	3.00E-02	-1.48E-01
ANKAR	ankyrin and armadillo repeat containing	3.00E-02	1.61E-01
NUDCD2	NudC domain containing 2	3.00E-02	1.59E-01
SLC6A10P	solute carrier family 6 member 10, pseudogene	3.01E-02	-4.86E-01
ACADS	acyl-CoA dehydrogenase, C-2 to C-3 short chain	3.01E-02	-2.50E-01
ZNF74	zinc finger protein 74	3.01E-02	2.26E-01
CISD1	CDGSH iron sulfur domain 1	3.01E-02	2.83E-01
SYNE1	spectrin repeat containing nuclear envelope protein 1	3.01E-02	-2.27E-01
AMPD3	adenosine monophosphate deaminase 3	3.01E-02	-1.45E-01

ARHGAP42	Rho GTPase activating protein 42	3.01E-02	1.63E-01
LDHC	lactate dehydrogenase C	3.01E-02	-4.14E-01
MBNL1	muscleblind like splicing regulator 1	3.01E-02	1.70E-01
MRPL34	mitochondrial ribosomal protein L34	3.01E-02	-5.65E-01
MAGED1	MAGE family member D1	3.01E-02	-3.66E-01
SUMF1	sulfatase modifying factor 1	3.01E-02	-3.48E-01
OSCP1	organic solute carrier partner 1	3.01E-02	3.43E-01
SLC25A15	solute carrier family 25 member 15	3.02E-02	-1.58E-01
SYNM	synemin	3.02E-02	4.25E-01
SHROOM4	shroom family member 4	3.02E-02	-5.17E-01
SLC39A10	solute carrier family 39 member 10	3.02E-02	-7.37E-01
NTN5	netrin 5	3.02E-02	-2.38E-01
MROH1	maestro heat like repeat family member 1	3.03E-02	1.79E-01
TGFBR3L	transforming growth factor beta receptor 3 like	3.03E-02	1.75E-01
RNF14	ring finger protein 14	3.03E-02	4.52E-01

LINC00173	long intergenic non-protein coding RNA 173	3.04E-02	-1.52E-01
KCNT2	potassium sodium-activated channel subfamily T member 2	3.04E-02	1.77E-01
LRPPRC	leucine rich pentatricopeptide repeat containing	3.04E-02	-7.18E-01
GLUD1	glutamate dehydrogenase 1	3.04E-02	4.88E-01
SNORD11B	small nucleolar RNA, C/D box 11B	3.04E-02	1.53E-01
MPZL2	myelin protein zero like 2	3.04E-02	-2.60E-01
BPHL	biphenyl hydrolase like	3.05E-02	-2.02E-01
PDHB	pyruvate dehydrogenase (lipoamide) beta	3.05E-02	1.68E-01
OR52K1	olfactory receptor family 52 subfamily K member 1	3.05E-02	-1.34E-01
CDCP1	CUB domain containing protein 1	3.05E-02	-2.24E-01
LINC00161	long intergenic non-protein coding RNA 161	3.05E-02	-1.39E-01
TROVE2	TROVE domain family member 2	3.05E-02	-6.13E-01
CC2D1A	coiled-coil and C2 domain containing 1A	3.05E-02	2.93E-01
POLR2J4	RNA polymerase II subunit J4, pseudogene	3.05E-02	-4.32E-01

NUDT6	nudix hydrolase 6	3.05E-02	2.55E-01
LTBP4	latent transforming growth factor beta binding protein 4	3.06E-02	-1.94E-01
AHR	aryl hydrocarbon receptor	3.06E-02	1.50E-01
CSRP1	cysteine and glycine rich protein 1	3.06E-02	2.28E-01
CSRNP1	cysteine and serine rich nuclear protein 1	3.06E-02	3.64E-01
GFPT2	glutamine-fructose-6-phosphate transaminase 2	3.06E-02	2.15E-01
OR13C9	olfactory receptor family 13 subfamily C member 9	3.07E-02	-1.95E-01
CLEC12A	C-type lectin domain family 12 member A	3.07E-02	-2.03E-01
SMCHD1	structural maintenance of chromosomes flexible hinge domain containing 1	3.07E-02	3.22E-01
RND2	Rho family GTPase 2	3.07E-02	-2.63E-01
KLHL11	kelch like family member 11	3.07E-02	1.41E-01
PHYH	phytanoyl-CoA 2-hydroxylase	3.07E-02	-7.80E-01
C19orf57	chromosome 19 open reading frame 57	3.07E-02	2.00E-01
PXDN	peroxidasin	3.07E-02	-2.65E-01
RHOBTB3	Rho related BTB domain containing 3	3.08E-02	-2.72E-01

MGST2	microsomal glutathione S-transferase 2	3.08E-02	-2.50E-01
GRASP	general receptor for phosphoinositides 1 associated scaffold protein	3.08E-02	-1.99E-01
DLX3	distal-less homeobox 3	3.08E-02	-2.22E-01
HPS5	HPS5, biogenesis of lysosomal organelles complex 2 subunit 2	3.08E-02	6.13E-01
FAM189A1	family with sequence similarity 189 member A1	3.08E-02	-1.63E-01
FAM133B	family with sequence similarity 133 member B	3.09E-02	3.60E-01
JADE1	jade family PHD finger 1	3.09E-02	-3.27E-01
DLX6-AS1	DLX6 antisense RNA 1	3.09E-02	-2.00E-01
R3HDM2	R3H domain containing 2	3.09E-02	-1.87E-01
CTHRC1	collagen triple helix repeat containing 1	3.09E-02	-1.54E-01
ZDHHC11	zinc finger DHHC-type containing 11	3.09E-02	-1.55E-01
BLOC1S5	biogenesis of lysosomal organelles complex 1 subunit 5	3.10E-02	2.09E-01
PFDN5	prefoldin subunit 5	3.10E-02	-3.01E-01

TMEM169	transmembrane protein 169	3.10E-02	-1.97E-01
TAS2R20	taste 2 receptor member 20	3.10E-02	-1.61E-01
HYMAI	hydatidiform mole associated and imprinted (non-protein coding)	3.10E-02	-1.55E-01
SAMD1	sterile alpha motif domain containing 1	3.10E-02	3.30E-01
BRAF	B-Raf proto-oncogene, serine/threonine kinase	3.11E-02	4.05E-01
CUTC	cutC copper transporter	3.11E-02	3.76E-01
CCDC125	coiled-coil domain containing 125	3.11E-02	-4.59E-01
HEPHL1	hephaestin like 1	3.11E-02	1.57E-01
PPP1R14B	protein phosphatase 1 regulatory inhibitor subunit 14B	3.11E-02	-5.29E-01
TMED10	transmembrane p24 trafficking protein 10	3.11E-02	-9.20E-01
FABP6	fatty acid binding protein 6	3.12E-02	1.64E-01
DEFB110	defensin beta 110	3.12E-02	-1.53E-01
THNSL2	threonine synthase like 2	3.12E-02	2.77E-01
THEM5	thioesterase superfamily member 5	3.13E-02	2.78E-01
PKN1	protein kinase N1	3.13E-02	3.11E-01

TFDP1	transcription factor Dp-1	3.13E-02	3.86E-01
SNORD35B	small nucleolar RNA, C/D box 35B	3.13E-02	-5.46E-01
ELF2	E74 like ETS transcription factor 2	3.13E-02	-2.93E-01
MDM1	Mdm1 nuclear protein	3.14E-02	1.60E-01
LOC100130992	uncharacterized LOC100130992	3.14E-02	-1.40E-01
PRAME	preferentially expressed antigen in melanoma	3.14E-02	-1.66E-01
SFPQ	splicing factor proline and glutamine rich	3.14E-02	4.23E-01
NPTN	neuroplastin	3.14E-02	-1.61E-01
RBX1	ring-box 1	3.14E-02	2.84E-01
KAZN	kazrin, periplakin interacting protein	3.15E-02	-1.57E-01
CLIC1	chloride intracellular channel 1	3.15E-02	2.08E-01
VAMP4	vesicle associated membrane protein 4	3.15E-02	3.36E-01
SENP8	SUMO/sentrin peptidase family member, NEDD8 specific	3.15E-02	2.49E-01
DUSP22	dual specificity phosphatase 22	3.15E-02	-6.00E-01
TNFRSF14	TNF receptor superfamily member 14	3.15E-02	3.87E-01

BLZF1	basic leucine zipper nuclear factor 1	3.15E-02	-6.40E-01
SELO	selenoprotein O	3.15E-02	-1.98E-01
SNX30	sorting nexin family member 30	3.16E-02	-3.44E-01
EXO5	exonuclease 5	3.16E-02	2.34E-01
ZBTB46	zinc finger and BTB domain containing 46	3.16E-02	-2.40E-01
WDR17	WD repeat domain 17	3.16E-02	-1.98E-01
TALDO1	transaldolase 1	3.16E-02	2.02E-01
PIGP	phosphatidylinositol glycan anchor biosynthesis class P	3.16E-02	-3.04E-01
HIST2H2AC	histone cluster 2, H2ac	3.17E-02	-6.40E-01
SCFD2	sec1 family domain containing 2	3.17E-02	1.67E-01
PQLC1	PQ loop repeat containing 1	3.17E-02	3.32E-01
FRMD1	FERM domain containing 1	3.17E-02	-1.58E-01
GNAI3	G protein subunit alpha i3	3.17E-02	2.35E-01
NEDD4	neural precursor cell expressed, developmentally down-regulated 4, E3 ubiquitin protein ligase	3.17E-02	2.21E-01

LSR	lipolysis stimulated lipoprotein receptor	3.17E-02	3.05E-01
CNOT2	CCR4-NOT transcription complex subunit 2	3.18E-02	-3.08E-01
BMF	Bcl2 modifying factor	3.18E-02	1.69E-01
PRKDC	protein kinase, DNA-activated, catalytic polypeptide	3.18E-02	9.33E-01
ZNF107	zinc finger protein 107	3.18E-02	2.36E-01
ANKRD34A	ankyrin repeat domain 34A	3.18E-02	-3.39E-01
RHBDL2	rhomboid like 2	3.18E-02	-3.92E-01
ZNF227	zinc finger protein 227	3.18E-02	5.96E-01
KLHL28	kelch like family member 28	3.19E-02	-5.24E-01
TTC13	tetratricopeptide repeat domain 13	3.19E-02	1.51E-01
MRPS28	mitochondrial ribosomal protein S28	3.19E-02	4.23E-01
CD36	CD36 molecule	3.19E-02	2.23E-01
MAP6D1	MAP6 domain containing 1	3.19E-02	3.99E-01
TSNAX	translin associated factor X	3.19E-02	-6.94E-01
TUBG1	tubulin gamma 1	3.19E-02	4.29E-01
PAK2	p21 (RAC1) activated kinase 2	3.20E-02	4.07E-01

RGS10	regulator of G-protein signaling 10	3.20E-02	-5.71E-01
FBN1	fibrillin 1	3.20E-02	1.71E-01
FAM96A	family with sequence similarity 96 member A	3.20E-02	1.47E-01
DDX39B	DEAD-box helicase 39B	3.20E-02	1.91E-01
TRAF2	TNF receptor associated factor 2	3.20E-02	1.52E-01
PNPLA2	patatin like phospholipase domain containing 2	3.20E-02	3.42E-01
CCL2	C-C motif chemokine ligand 2	3.20E-02	2.09E-01
GNGT1	G protein subunit gamma transducin 1	3.20E-02	1.62E-01
CCNL2	cyclin L2	3.21E-02	-3.51E-01
RGPD2	RANBP2-like and GRIP domain containing 2	3.21E-02	3.28E-01
SP7	Sp7 transcription factor	3.21E-02	1.47E-01
PTCHD4	patched domain containing 4	3.21E-02	-1.52E-01
PAK1	p21 (RAC1) activated kinase 1	3.21E-02	3.53E-01
EIF5B	eukaryotic translation initiation factor 5B	3.22E-02	2.16E-01
FAM102B	family with sequence similarity 102 member B	3.22E-02	6.13E-01
HOXC10	homeobox C10	3.22E-02	-4.42E-01

HCN2	hyperpolarization activated cyclic nucleotide gated potassium channel 2	3.22E-02	-1.55E-01
ZDHHC16	zinc finger DHHC-type containing 16	3.22E-02	-1.95E-01
BCYRN1	brain cytoplasmic RNA 1	3.22E-02	6.80E-01
OVCA2	ovarian tumor suppressor candidate 2	3.22E-02	-1.89E-01
PCM1	pericentriolar material 1	3.22E-02	7.31E-01
SNORD14B	small nucleolar RNA, C/D box 14B	3.23E-02	2.43E-01
WNT3A	Wnt family member 3A	3.23E-02	-1.63E-01
NACC1	nucleus accumbens associated 1	3.23E-02	2.25E-01
GSTA3	glutathione S-transferase alpha 3	3.23E-02	1.54E-01
B3GNTL1	UDP-GlcNAc:betaGal beta-1,3-N-acetylglucosaminyltransferase-like 1	3.23E-02	-1.98E-01
EXTL2	exostosin like glycosyltransferase 2	3.23E-02	-5.95E-01
ZNF587	zinc finger protein 587	3.24E-02	1.46E-01
MYRIP	myosin VIIA and Rab interacting protein	3.24E-02	1.97E-01
TMEM65	transmembrane protein 65	3.24E-02	4.50E-01
MAST1	microtubule associated serine/threonine kinase 1	3.24E-02	-5.20E-01

LOC440895	two pore channel 3 pseudogene	3.24E-02	-2.03E-01
ZNF202	zinc finger protein 202	3.24E-02	2.02E-01
C1orf122	chromosome 1 open reading frame 122	3.25E-02	-5.87E-01
TSNAX-DISC1///DISC1///TSNAX	TSNAX-DISC1 readthrough (NMD candidate)///disrupted in schizophrenia 1///translin associated factor X	3.25E-02	2.19E-01
CLPX	caseinolytic mitochondrial matrix peptidase chaperone subunit	3.25E-02	5.30E-01
ZNF195	zinc finger protein 195	3.25E-02	-5.79E-01
NEBL	nebulette	3.25E-02	1.62E-01
RAB5C	RAB5C, member RAS oncogene family	3.25E-02	-4.49E-01
ZNF423	zinc finger protein 423	3.25E-02	-1.71E-01
MTPAP	mitochondrial poly(A) polymerase	3.26E-02	3.39E-01
PIN1P1	peptidylprolyl cis/trans isomerase, NIMA-interacting 1 pseudogene 1	3.26E-02	2.01E-01
TBX1	T-box 1	3.26E-02	-1.84E-01
YTHDC1	YTH domain containing 1	3.26E-02	3.04E-01
ATR	ATR serine/threonine kinase	3.27E-02	1.54E-01
SIK1	salt inducible kinase 1	3.27E-02	3.93E-01

FAM160A2	family with sequence similarity 160 member A2	3.27E-02	3.07E-01
CDV3	CDV3 homolog	3.27E-02	4.08E-01
KLHL9	kelch like family member 9	3.27E-02	-1.91E-01
PPT2	palmitoyl-protein thioesterase 2	3.27E-02	-2.02E-01
PLD1	phospholipase D1	3.27E-02	1.86E-01
RAB39B	RAB39B, member RAS oncogene family	3.28E-02	-2.74E-01
XIAP	X-linked inhibitor of apoptosis	3.28E-02	-2.28E-01
API5	apoptosis inhibitor 5	3.28E-02	7.92E-01
SNORA36A	small nucleolar RNA, H/ACA box 36A	3.28E-02	1.84E-01
ZC3H14	zinc finger CCCH-type containing 14	3.28E-02	1.65E-01
CXCL2	C-X-C motif chemokine ligand 2	3.28E-02	1.75E-01
AK4	adenylate kinase 4	3.28E-02	-2.78E-01
COMTD1	catechol-O-methyltransferase domain containing 1	3.29E-02	-5.83E-01
LINC00094	long intergenic non-protein coding RNA 94	3.29E-02	3.78E-01
CD177	CD177 molecule	3.29E-02	-1.55E-01

MRPS24	mitochondrial ribosomal protein S24	3.29E-02	5.40E-01
IFT88	intraflagellar transport 88	3.29E-02	4.24E-01
FGD5P1	FYVE, RhoGEF and PH domain containing 5 pseudogene 1	3.29E-02	-3.66E-01
FAM8A1	family with sequence similarity 8 member A1	3.29E-02	-4.43E-01
FAM214A	family with sequence similarity 214 member A	3.30E-02	-4.42E-01
CDC26	cell division cycle 26	3.30E-02	4.94E-01
MMP25	matrix metallopeptidase 25	3.30E-02	1.36E-01
PRODH	proline dehydrogenase 1	3.30E-02	-1.69E-01
OR4K14	olfactory receptor family 4 subfamily K member 14	3.30E-02	-1.67E-01
RPS6KA5	ribosomal protein S6 kinase A5	3.30E-02	3.82E-01
CLCN5	chloride voltage-gated channel 5	3.31E-02	1.70E-01
NUDT11	nudix hydrolase 11	3.31E-02	-2.04E-01
POLR3A	RNA polymerase III subunit A	3.31E-02	-3.34E-01
KLHL7	kelch like family member 7	3.31E-02	4.43E-01
PACSIN3	protein kinase C and casein kinase substrate in neurons 3	3.31E-02	-2.69E-01

PHYHD1	phytanoyl-CoA dioxygenase domain containing 1	3.31E-02	-2.74E-01
SDHAP1	succinate dehydrogenase complex flavoprotein subunit A pseudogene 1	3.31E-02	-1.79E-01
PTPN14	protein tyrosine phosphatase, non-receptor type 14	3.31E-02	-2.54E-01
GMPPA	GDP-mannose pyrophosphorylase A	3.32E-02	-1.70E-01
STOML2	stomatin like 2	3.32E-02	2.38E-01
NUPR1	nuclear protein 1, transcriptional regulator	3.32E-02	-2.50E-01
RANBP6	RAN binding protein 6	3.32E-02	3.60E-01
MIR625	microRNA 625	3.33E-02	-1.44E-01
SAFB2	scaffold attachment factor B2	3.33E-02	2.52E-01
GJA10	gap junction protein alpha 10	3.33E-02	-2.20E-01
CDC14B	cell division cycle 14B	3.34E-02	-3.52E-01
NFX1	nuclear transcription factor, X-box binding 1	3.34E-02	-2.28E-01
RNF4	ring finger protein 4	3.34E-02	3.10E-01
PPT2	palmitoyl-protein thioesterase 2	3.35E-02	-4.78E-01

SLC26A2	solute carrier family 26 member 2	3.35E-02	-<br>3.62E<br>-01

Supplementary Table 2. Up-regulated and down-regulated differentially expressed genes from two groups of GSE126519 dataset.

**Up-regulating genes**			**Down-regulating genes**		
**Gene ID**	**p-value**	**log2FC**	**Gene ID**	**p-value**	**log2FC**
SDPR	1.43E-07	6.74	*CDKN1A*	2.85E-07	-6.08
NABP1	1.65E-07	4.41	*DKK1*	5.49E-06	-5.76
NCAPG	1.65E-07	4.03	*NTS*	4.28E-07	-5.74
NEK2	1.78E-07	4.44	*S100A4*	1.65E-07	-5.53
EPDR1	2.04E-07	3.49	*GDF15*	1.43E-07	-5.01
NUP62CL	2.30E-07	3.63	*CYP1B1*	3.70E-06	-4.9
IGFBP3	2.85E-07	6.44	*TYRP1*	1.14E-06	-4.73
CLDN1	2.85E-07	5.33	*MAP1LC3A*	3.82E-07	-4.45
PBK	2.85E-07	3.77	*C8orf4*	5.53E-07	-4.36
ATP1B1	3.82E-07	5.5	*HAPLN1*	2.04E-07	-4.32
TM4SF1	3.82E-07	4.88	*NAP1L3*	2.23E-06	-4.1
GBP1	3.82E-07	4.16	*PCDH20*	1.13E-06	-4.09
FAM9C	3.82E-07	3.8	*VCAN*	2.58E-05	-4.08
CDC45	3.82E-07	3.77	*UCHL1*	9.09E-07	-4.05
MCM10	3.82E-07	3.71	*TWIST1*	8.73E-07	-4.01
DLGAP5	3.82E-07	3.69	*ITM2A*	1.14E-06	-4
TMEM30B	3.82E-07	3.64	*RGS16*	3.82E-07	-3.98
CDCA5	3.82E-07	3.57	*CDH10*	4.15E-06	-3.86
OIP5	3.82E-07	3.46	*RERGL*	3.90E-06	-3.82
RBM24	3.82E-07	3.32	*KLHL14*	0.000146	-3.7

SLC16A14	3.82E-07	3.24	*S100A11*	6.35E-06	-3.69
ACTG2	3.82E-07	3.1	*PCOLCE*	1.83E-06	-3.67
RAB3IP	3.82E-07	3.05	*MME*	0.000203	-3.66
DTNA	3.82E-07	2.96	*PDE4C*	8.34E-07	-3.65
GALC	3.82E-07	2.74	*HRC*	2.00E-05	-3.6
ISL1	4.28E-07	5.72	*ITM2B*	5.00E-06	-3.52
HMMR	4.28E-07	3.18	*PLBD1*	3.82E-07	-3.41
POSTN	4.38E-07	5.47	*BST2*	0.000123	-3.41
MELK	4.38E-07	3.16	*CD248*	6.69E-06	-3.38
CEP55	4.45E-07	4.1	*CD70*	5.35E-05	-3.35
EPCAM	5.12E-07	4.08	*LHX8*	3.82E-07	-3.32
CD36	5.12E-07	3.27	*ANTXR1*	1.78E-05	-3.25
LPAR1	5.39E-07	2.63	*EOMES*	1.33E-06	-3.23
MID1	5.53E-07	4.72	*LIN28A*	9.80E-07	-3.21
NRG1	5.71E-07	4.1	*BMP7*	1.20E-05	-3.21
NUSAP1	5.71E-07	4.02	*COL3A1*	0.000293	-3.21
TMEFF2	5.71E-07	3.44	*TCEAL8*	3.42E-05	-3.16
CCNO	5.71E-07	3.02	*AXL*	1.18E-05	-3.13
P3H2	5.83E-07	4.76	*PPP1R14A*	1.27E-05	-3.07
DDIAS	5.86E-07	2.44	*GCG*	3.84E-06	-3.05
TMEM98	6.30E-07	2.65	*FES*	0.000139	-3.04
B3GALNT1	6.48E-07	2.21	*TMEM47*	4.47E-06	-2.99
PRTFDC1	6.51E-07	2.54	*QPCT*	5.12E-07	-2.97
FLRT3	6.95E-07	3.79	*CBR3*	9.74E-05	-2.91
EXO1	7.00E-07	3.17	*WDR63*	3.25E-06	-2.9
MMP3	7.58E-07	4.21	*PNCK*	3.56E-06	-2.85
CITED2	7.79E-07	2.91	*JMJD8*	0.000316	-2.82
ARHGAP28	8.73E-07	2.96	*BAX*	0.000649	-2.8
CCNA2	9.02E-07	3.66	*COL1A2*	0.000346	-2.76
RAD51AP1	9.02E-07	3.41	*MIR199A2*	8.05E-05	-2.74
MDK	9.08E-07	4.37	*SESN1*	8.24E-05	-2.74
SYNE4	9.08E-07	2.63	*ALX1*	1.28E-06	-2.71
ANKRD1	9.92E-07	4.48	*NOTCH3*	2.69E-06	-2.71
SGCE	9.92E-07	2.87	*SH3BP4*	3.98E-05	-2.69
MARCKS	9.97E-07	2.6	*MEIS2*	0.000181	-2.68
RGS20	1.06E-06	3.3	*KERA*	2.94E-05	-2.67
FANCD2	1.13E-06	2.88	*PLPPR5*	3.30E-06	-2.67
SKA3	1.14E-06	2.27	*QPRT*	1.21E-05	-2.63
PKIA	1.28E-06	2.18	*COL6A1*	0.000206	-2.62
AUNIP	1.28E-06	2.18	*PHLDA3*	4.33E-06	-2.6
KIF14	1.28E-06	2.18	*BCAT1*	0.000282	-2.6
CGNL1	1.30E-06	2.28	*LCN15*	5.37E-05	-2.59
SCHIP1	1.39E-06	2.29	*C14orf169*	2.75E-05	-2.57
KIF4A	1.39E-06	2.14	*INPP1*	6.86E-05	-2.54
KCNJ16	1.40E-06	3.01	*NKX2-5*	2.47E-05	-2.53
POLE2	1.42E-06	2.38	*TLX3*	5.42E-06	-2.52

C9ORF3	1.42E-06	2.33	*VIP*	7.94E-06	-2.5
PRIM1	1.45E-06	2.73	*ABCA1*	1.71E-05	-2.5
HJURP	1.48E-06	2.32	*SFRP1*	0.000539	-2.47
LRRTM4	1.49E-06	2.9	*SNAI2*	5.71E-07	-2.46
SGO1	1.58E-06	2.33	*EFHD1*	5.12E-07	-2.46
MMP1	1.59E-06	4.26	*ALDH2*	6.72E-07	-2.46
CHODL	1.59E-06	2.52	*PERP*	8.26E-05	-2.43
SLF1	1.66E-06	3.14	*SSPO*	6.35E-06	-2.42
TNFRSF19	1.68E-06	4.17	*PLCL2*	3.92E-05	-2.42
UBE2E2	1.84E-06	3.27	*ZNF20*	4.43E-06	-2.41
CCDC138	1.84E-06	3.08	*FGF18*	3.25E-05	-2.41
TPD52L1	1.86E-06	3.44	*CLIP3*	2.21E-05	-2.39
MT1F	1.88E-06	2.31	*TCEAL3*	6.88E-05	-2.38
RNLS	1.93E-06	3.02	*PLTP*	0.0025	-2.38
KIF20B	1.98E-06	2.07	*LAMC3*	4.43E-06	-2.38
KCNJ2	2.17E-06	3.49	*SLC30A8*	9.35E-05	-2.38
SPOCK2	2.23E-06	4.46	*UCA1*	0.000182	-2.38
SCN9A	2.29E-06	2.55	*CD99*	2.87E-05	-2.37
CDC25C	2.30E-06	1.8	*CLEC11A*	4.67E-05	-2.36
BIRC3	2.40E-06	2.11	*CKB*	1.93E-06	-2.34
MGST1	2.57E-06	3.35	*CFD*	0.000325	-2.34
MYO1B	2.72E-06	3.83	*MXD4*	0.000181	-2.33
NDC80	2.72E-06	3.1	*SNORA12*	0.001	-2.33
FAM72D	2.72E-06	2.46	*MBNL2*	1.38E-05	-2.29
DSN1	2.72E-06	2	*SOX8*	1.66E-05	-2.29
MCM8	2.76E-06	1.66	*RPS29*	0.000693	-2.29
ASB5	2.85E-06	2.9	*LRRC4C*	5.27E-05	-2.28
KIF11	2.89E-06	2.42	*TFAP2A*	1.52E-05	-2.27
CPQ	3.14E-06	1.62	*TIGAR*	3.71E-06	-2.26
CCNB1	3.25E-06	2.09	*XIST*	0.000305	-2.24
AURKA	3.33E-06	1.83	*FGFR3*	3.69E-05	-2.23
ATP11B	3.56E-06	2.66	*H19*	4.10E-05	-2.23
ZNF204P	3.66E-06	2.44	*APOE*	1.71E-05	-2.21
NMU	3.82E-06	3.44	*BVES*	0.000355	-2.2
FEN1	3.82E-06	2.73	*MYOC*	4.51E-06	-2.19
ANXA3	4.10E-06	2.9	*ANK3*	1.21E-05	-2.19
MESP1	4.10E-06	1.95	*PLPPR4*	0.00128	-2.18
CKAP2L	4.10E-06	1.47	*SSBP2*	4.33E-06	-2.18
ERI1	4.28E-06	3.2	*RPL13A*	1.57E-05	-2.17
UBE2C	4.33E-06	4.01	*PPM1D*	2.52E-05	-2.17
DBNDD2	4.33E-06	3.96	*BTG2*	7.18E-05	-2.16
CRISPLD1	4.33E-06	2.21	*TP53INP1*	0.00101	-2.15
MEOX1	4.33E-06	1.86	*COQ8A*	0.000177	-2.15
PDE4B	4.33E-06	1.82	*COL23A1*	2.94E-05	-2.14
MOCOS	4.33E-06	1.81	*LOC105379362*	0.000258	-2.14
ESCO2	4.35E-06	2.33	*GRHL3*	1.78E-05	-2.13

ERCC6L	4.35E-06	2.02	*TP53I3*	0.000529	-2.1
DYNLT3	4.43E-06	3.48	*ALPL*	4.00E-04	-2.09
TTK	4.43E-06	3.44	*SERPING1*	1.23E-05	-2.08
AP1S2	4.43E-06	2.77	*DKK3*	1.72E-05	-2.07
LRRN3	4.43E-06	2.72	*SAMD5*	3.84E-06	-2.07
DEPDC1B	4.43E-06	2.7	*HS1BP3*	1.29E-05	-2.07
GATM	4.43E-06	2.41	*FSCN1*	0.000129	-2.07
LGALS8	4.43E-06	2.13	*TFPI*	0.000103	-2.06
MCM5	4.43E-06	2.09	*RCAN1*	5.99E-05	-2.05
CENPA	4.54E-06	2.1	*GAS6*	5.09E-05	-2.05
FXYD5	5.04E-06	3.95	*HSPA5*	7.61E-06	-2.05
NMI	5.04E-06	2.35	*JPH3*	3.17E-05	-2.05
ISOC1	5.04E-06	2.32	*LOC286254*	0.000486	-2.05
CCNB2	5.05E-06	2.97	*FOXG1*	3.29E-05	-2.03
CDCA8	5.05E-06	2.48	*CLYBL*	4.47E-05	-2.02
DEPDC1	5.07E-06	3.16	*NES*	4.10E-05	-2.02
FBXO5	5.29E-06	2.21	*ANKRD37*	0.00149	-2.02
FAM64A	5.37E-06	2.23	*AGBL5*	0.000131	-2.01
RACGAP1	5.42E-06	2.08	*CA2*	2.94E-05	-2
CXCL1	5.45E-06	3.92	*EFNB3*	0.000106	-2
GBP1P1	5.92E-06	2	*CCDC178*	0.000124	-2
SPATS2L	5.92E-06	1.87	*GYG2*	3.01E-05	-1.99
ANXA1	6.09E-06	3.47	*GPER1*	3.25E-06	-1.99
PLK4	6.26E-06	2.54	*DACT3*	4.61E-05	-1.99
MRAP2	6.32E-06	1.87	*CHFR*	4.94E-05	-1.99
INA	6.64E-06	2.52	*TRIM51*	8.44E-05	-1.99
KIF2C	6.64E-06	2.37	*MATK*	0.00307	-1.99
CDK2	6.73E-06	1.8	*SLC2A3*	4.51E-05	-1.96
MBP	6.75E-06	2.3	*FAM133A*	3.56E-06	-1.95
AURKB	6.79E-06	3.17	*PROCR*	5.53E-05	-1.95
ZWINT	6.93E-06	2.77	*C1orf54*	0.000157	-1.94
FAM111A	7.18E-06	2.54	*RHOQ*	0.000169	-1.93
WLS	7.18E-06	1.73	*ARID5B*	0.000258	-1.93
SKIDA1	7.23E-06	2.18	*AGRN*	0.000449	-1.93
BUB1	7.27E-06	2.77	*CIART*	0.00102	-1.93
ZNF816	7.44E-06	1.35	*HIST3H2A*	0.00263	-1.93
SERPINB1	7.51E-06	2.73	*DDX54*	8.04E-06	-1.92
TAGLN	7.68E-06	2.58	*DPYSL4*	0.000304	-1.92
RIPPLY3	7.76E-06	2.03	*MACROD1*	0.000342	-1.92
NUP107	7.76E-06	1.78	*NKD2*	1.52E-05	-1.91
MOCS1	7.76E-06	1.47	*STRA6*	4.22E-05	-1.91
IL1RAP	7.94E-06	2.8	*CYB5A*	6.02E-05	-1.91
PSMC3IP	7.97E-06	1.43	*RBM20*	0.000355	-1.91
ADAMTS1	8.04E-06	2.89	*RTN2*	9.87E-06	-1.9
AP1M2	8.07E-06	1.96	*PLPP7*	1.90E-06	-1.89
TOP2A	8.08E-06	3.85	*OCA2*	1.19E-05	-1.88

HPSE	8.11E-06	1.45	*SPACA6*	1.78E-05	-1.88
SHCBP1	8.13E-06	2.32	*SIRPA*	3.64E-05	-1.88
CDCA3	8.32E-06	2.98	*TNFSF9*	1.74E-05	-1.86
TSPAN12	8.32E-06	1.4	*DRAM1*	0.000142	-1.86
MCM4	8.39E-06	2.32	*UGCG*	0.00129	-1.86
PSD3	8.43E-06	2.51	*CMBL*	1.29E-05	-1.85
SKA1	8.47E-06	1.85	*DCN*	1.47E-05	-1.85
NFIB	8.91E-06	2.21	*RGS2*	1.56E-05	-1.85
TACC3	9.16E-06	2.42	*OXR1*	3.59E-05	-1.85
GRB14	9.16E-06	1.89	*SPRY4*	0.000568	-1.85
CEP128	9.32E-06	1.65	*SNCG*	8.00E-04	-1.85
LHX2	9.36E-06	1.27	*PHPT1*	1.21E-05	-1.83
ENO2	9.58E-06	2.01	*SDC1*	0.000131	-1.83
IL12A	1.01E-05	1.32	*NEURL2*	0.000385	-1.83
FANCI	1.04E-05	2.83	*WBP1*	0.000176	-1.82
MAD2L1	1.06E-05	2.89	*FAM210B*	0.000549	-1.82
SLC38A4	1.06E-05	1.32	*LUC7L3*	0.00522	-1.82
B3GNT5	1.08E-05	1.62	*ENO3*	1.63E-05	-1.81
SPAG6	1.12E-05	2	*A2M*	0.000285	-1.81
BIRC5	1.13E-05	3.12	*IGDCC4*	0.000534	-1.81
SPC25	1.13E-05	2.42	*MID1IP1*	1.57E-05	-1.8
PTPRK	1.20E-05	2.29	*RTN4*	0.00111	-1.8
TDRD7	1.20E-05	2.01	*PCCA*	1.84E-05	-1.79
BIN1	1.20E-05	1.74	*PCDH7*	0.000717	-1.79
LRRCC1	1.21E-05	2.56	*CXXC5*	1.97E-05	-1.77
KIF20A	1.21E-05	2.25	*CDC42EP5*	4.85E-06	-1.77
IFRD1	1.21E-05	1.76	*FEZ1*	5.02E-05	-1.77
MAP7D2	1.21E-05	1.73	*SLC8A2*	6.06E-05	-1.77
NCAPG2	1.23E-05	1.85	*ERO1A*	5.29E-05	-1.76
PRKCH	1.29E-05	2.17	*EMP3*	0.00128	-1.76
GCLM	1.29E-05	1.37	*EEF1A1*	4.87E-05	-1.75
TK1	1.31E-05	2.31	*ACY1*	5.82E-05	-1.75
KIFC1	1.31E-05	2.04	*OSBPL7*	0.000115	-1.75
MLKL	1.31E-05	1.96	*FLNC*	0.000451	-1.75
EPHX4	1.31E-05	1.74	*DDR2*	0.00364	-1.75
GALNT14	1.33E-05	1.12	*PCDHB16*	2.05E-05	-1.74
FOXM1	1.37E-05	1.81	*LPCAT3*	3.08E-05	-1.74
NXT2	1.37E-05	1.78	*SCNN1A*	0.000354	-1.74
PAX6	1.38E-05	2.23	*CYSRT1*	0.00213	-1.74
ZAK	1.38E-05	1.64	*NTPCR*	0.000134	-1.73
BLM	1.38E-05	1.34	*TDRD3*	0.000795	-1.73
TOM1L2	1.41E-05	2.19	*DLX1*	3.70E-06	-1.72
DTL	1.49E-05	3.41	*STXBP5*	0.000933	-1.71
VGLL3	1.49E-05	2.32	*RPL34*	0.000224	-1.7
NEIL3	1.49E-05	1.64	*CA11*	0.000589	-1.7
HOXB5	1.50E-05	1.26	*SF1*	0.00569	-1.7

SESTD1	1.51E-05	1.42	*FZD8*	3.81E-05	-1.69
ZFP36L1	1.51E-05	1.18	*COX20*	0.000238	-1.69
NR2F2	1.52E-05	2.92	*PFKFB4*	0.00195	-1.69
PDGFC	1.53E-05	2.33	*RUNDC3A*	4.08E-06	-1.68
NEBL	1.55E-05	1.83	*FZD2*	5.88E-05	-1.68
TYRO3	1.55E-05	1.09	*CERS5*	7.38E-05	-1.68
RNF175	1.57E-05	1.43	*ZNF581*	0.000519	-1.68
CASC10	1.57E-05	1.36	*ARHGAP32*	0.00533	-1.68
PAWR	1.60E-05	1.96	*MIR214*	0.000362	-1.67
FAM83D	1.61E-05	1.72	*TYMSOS*	0.000113	-1.66
NPR2	1.61E-05	1.45	*APPBP2*	0.000165	-1.66
RFC4	1.63E-05	2.6	*LIPA*	0.000325	-1.66
E2F2	1.66E-05	2.85	*LAMB2*	0.000494	-1.66
FZD6	1.71E-05	2.23	*STC2*	7.15E-06	-1.65
POLQ	1.74E-05	1.84	*CXXC4*	1.29E-05	-1.65
ADAMTS5	1.76E-05	1.59	*SLC25A6*	3.32E-05	-1.64
DDX10	1.78E-05	2.26	*GNG11*	0.000458	-1.64
ERRFI1	1.78E-05	1.84	*TM7SF3*	0.00233	-1.64
FRY	1.78E-05	1.11	*BIVM*	0.000286	-1.63
RAD54L	1.82E-05	1.74	*GLT8D2*	3.70E-06	-1.63
OGFRL1	1.85E-05	2.78	*VWCE*	4.75E-05	-1.63
ASPM	1.86E-05	2.22	*RRM2B*	7.02E-05	-1.63
MTFR2	1.93E-05	2.01	*TMEM88*	0.00161	-1.63
PRC1	2.00E-05	3.17	*CD68*	0.00237	-1.63
SATB2	2.00E-05	2.17	*GPC5*	1.16E-05	-1.62
PARPBP	2.00E-05	1.73	*EBPL*	0.000848	-1.62
ZDHHC13	2.01E-05	1.51	*MC1R*	0.000868	-1.6
KIF18A	2.03E-05	1.45	*FSTL5*	9.88E-05	-1.59
C9ORF40	2.05E-05	1.27	*LAMP1*	0.00015	-1.59
VRK1	2.08E-05	2.23	*PTRHD1*	0.00132	-1.59
SMARCA2	2.08E-05	1.3	*LHX3*	0.000554	-1.58
SUV39H1	2.11E-05	1.83	*ZMYND8*	1.38E-05	-1.57
TRAPPC3L	2.15E-05	2.92	*PTPRU*	6.64E-06	-1.57
MASTL	2.16E-05	1.82	*PPP1R3C*	4.74E-05	-1.57
HS6ST2	2.21E-05	2.45	*CES2*	7.74E-05	-1.57
SH2D4A	2.21E-05	2.41	*CTSF*	9.13E-05	-1.57
PCGF5	2.21E-05	1.44	*SEMA6A*	2.86E-05	-1.56
TMSB15A	2.31E-05	2.41	*SEMA6B*	2.87E-05	-1.56
BRCA1	2.31E-05	1.94	*FAM198B*	1.00E-04	-1.56
SLC39A8	2.32E-05	2.38	*RILPL2*	0.000186	-1.56
PMAIP1	2.36E-05	1.87	*AARD*	0.000602	-1.56
CDCA2	2.40E-05	1.64	*COL4A5*	0.00116	-1.56
GINS2	2.47E-05	2.75	*TTYH3*	0.00143	-1.56
IQCK	2.47E-05	1.52	*ZNF608*	0.00168	-1.56
TROAP	2.48E-05	2.1	*DHRS2*	7.14E-05	-1.55
MLIP	2.49E-05	1.41	*UBAC2*	0.000233	-1.55

STIL	2.62E-05	2.06	*MXRA8*	1.21E-05	-1.54
UCP2	2.67E-05	2.11	*RBPMS*	6.35E-05	-1.54
ANLN	2.71E-05	2.95	*SLC25A42*	0.000207	-1.54
FILIP1	2.71E-05	1.7	*FAM127C*	0.00589	-1.54
TRPC4	2.71E-05	1.39	*FBXO2*	1.37E-05	-1.53
KCNMB4	2.76E-05	1.47	*HIC2*	0.000185	-1.53
EXOSC8	2.78E-05	1.94	*TMEM132A*	0.000203	-1.53
TRIP13	2.89E-05	1.38	*EPOR*	0.000734	-1.53
C1ORF112	2.90E-05	1.69	*GSTM2*	0.000833	-1.52
DNAJC9	2.98E-05	1.5	*NADSYN1*	1.42E-05	-1.52
SLC27A6	2.98E-05	1.38	*RALGDS*	0.00412	-1.52
MND1	3.00E-05	3	*GPC6*	1.78E-05	-1.51
JUN	3.00E-05	1.72	*TCF4*	5.27E-05	-1.51
ISPD	3.00E-05	1.02	*NDST4*	0.000264	-1.51
TMEM237	3.01E-05	1.26	*CEACAM1*	3.53E-05	-1.5
HEY1	3.06E-05	1.92	*NDRG4*	5.55E-05	-1.5
DUT	3.06E-05	1.3	*CLN5*	6.44E-05	-1.5
MAT2B	3.13E-05	1.23	*DDIT4*	6.70E-05	-1.5
BBOF1	3.19E-05	1.43	*ANKRD24*	0.000275	-1.5
RPP40	3.31E-05	1.61	*SCD5*	4.10E-05	-1.49
MCUB	3.31E-05	1.41	*KCNG1*	0.000282	-1.49
THNSL2	3.35E-05	2.61	*TRIM6*	0.000153	-1.48
KIF23	3.35E-05	2.23	*SPATA20*	0.00159	-1.48
KAZALD1	3.35E-05	1.81	*PITPNC1*	0.000209	-1.47
PIR	3.35E-05	1.65	*ATP8B2*	0.000304	-1.47
RMI1	3.35E-05	1.47	*SLC7A10*	0.000339	-1.47
AGR2	3.35E-05	1.37	*RHOBTB1*	0.00196	-1.47
HMGB1	3.38E-05	2.51	*PLD3*	0.00248	-1.47
KIAA1147	3.38E-05	2.41	*TSC22D1*	0.0117	-1.47
ZNF426	3.43E-05	1.08	*EBI3*	0.000279	-1.46
MCM2	3.47E-05	2.3	*SLC7A2*	0.001	-1.46
DNAJC15	3.47E-05	1.58	*F2R*	0.00141	-1.46
ELL2	3.53E-05	1.87	*SNORA24*	0.00419	-1.46
TM4SF18	3.53E-05	1.67	*MAGED2*	0.00267	-1.45
LRR1	3.59E-05	1.7	*ISCU*	0.000255	-1.45
BUB3	3.59E-05	1.42	*SLTM*	4.54E-05	-1.45
TUBG1	3.59E-05	1.39	*CANX*	2.65E-05	-1.45
STMN1	3.69E-05	1.73	*ATP5L*	0.000173	-1.45
FMN2	3.69E-05	1.29	*KLHL24*	0.000202	-1.45
PAQR7	3.69E-05	1.23	*SGSH*	0.00147	-1.45
HAUS8	3.70E-05	1.8	*HGF*	0.00294	-1.44
SPRY2	3.70E-05	1.4	*HOXC13*	8.13E-06	-1.44
PRMT6	3.71E-05	1.47	*MAP1A*	0.000194	-1.44
HELLS	3.76E-05	1.74	*SLC29A4*	0.000241	-1.44
PDLIM3	3.76E-05	1.54	*GSTM1*	0.000462	-1.44
ZNF583	3.80E-05	1.22	*FAAP20*	0.00179	-1.44

UBE2T	3.82E-05	2.01	*NOS3*	0.0025	-1.44
CHEK1	3.82E-05	1.12	*CYFIP2*	0.000418	-1.43
RFC5	3.86E-05	2.16	*FAM13A*	0.00128	-1.42
CENPV	3.92E-05	1.95	*PLCD1*	2.08E-05	-1.42
SIX1	3.92E-05	1.93	*CKMT1B*	2.87E-05	-1.42
PIK3AP1	3.93E-05	1.2	*C1orf52*	2.75E-05	-1.41
SCG2	3.93E-05	1.19	*PTGES*	4.78E-05	-1.41
ECT2	3.98E-05	1.5	*DNAH7*	4.86E-05	-1.41
NUF2	4.03E-05	2.62	*CFLAR*	5.14E-05	-1.41
MUC15	4.04E-05	2.46	*SLC22A18*	8.86E-05	-1.41
CDKN3	4.10E-05	2.72	*GRIN2C*	0.000165	-1.41
USP1	4.22E-05	2.12	*FAM84B*	0.000362	-1.41
NETO2	4.23E-05	1.79	*RAC3*	0.0013	-1.41
RNASEH2A	4.24E-05	2.26	*DBP*	0.00221	-1.41
GINS3	4.29E-05	1.67	*UPK1A*	0.0123	-1.41
PLCG2	4.29E-05	1.26	*XPC*	0.0168	-1.41
MAR-02	4.29E-05	1.11	*SLC2A1*	7.19E-05	-1.4
SKAP1	4.31E-05	1.72	*PIDD1*	0.00023	-1.39
SEH1L	4.32E-05	1.3	*HSD17B14*	0.00279	-1.39
ARL6	4.41E-05	1.84	*GAMT*	0.00538	-1.38
MCTP1	4.47E-05	1.15	*SOSTDC1*	2.68E-05	-1.38
ELOVL4	4.51E-05	1.63	*PNPLA7*	0.000358	-1.38
DNA2	4.53E-05	1.44	*ARIH2*	0.000372	-1.38
TNNI3	4.54E-05	1.19	*NGFR*	0.000456	-1.38
CENPU	4.60E-05	1.55	*YPEL5*	0.00164	-1.38
LRRC1	4.62E-05	1.1	*ANXA4*	0.00321	-1.38
NUP54	4.69E-05	1.38	*ARRB1*	0.000107	-1.37
PUS7L	4.69E-05	1.19	*ZNF467*	0.00192	-1.37
PTPRE	4.71E-05	1.68	*PLCG1*	0.000131	-1.36
DDX52	4.74E-05	1.16	*RUNX3*	0.00151	-1.36
CDH7	4.79E-05	1.16	*CALY*	0.00191	-1.36
KNTC1	4.83E-05	1.39	*CLDN15*	0.00246	-1.36
CCR1	4.95E-05	1.25	*TMC6*	3.80E-05	-1.35
ATL3	4.99E-05	1.19	*SARS*	5.82E-05	-1.35
ACTA2	5.04E-05	1.92	*SLC12A4*	0.000895	-1.35
SIX4	5.04E-05	1.58	*TSKU*	0.00166	-1.35
TESK1	5.04E-05	1.08	*EXOC7*	0.00194	-1.35
DSCC1	5.09E-05	2.36	*IER3*	0.00609	-1.35
HAUS6	5.09E-05	1.43	*CKMT1A*	1.79E-05	-1.34
CLIC3	5.12E-05	1.14	*GOLGA8A*	0.000498	-1.34
SPAG1	5.14E-05	2.37	*C4orf3*	0.00121	-1.34
SPDL1	5.27E-05	1.93	*IFIT1*	6.97E-05	-1.33
TMSB15B	5.37E-05	1.78	*CPA2*	0.000135	-1.33
TOX	5.50E-05	1.25	*ZNF692*	0.000138	-1.33
GUCY1A3	5.65E-05	1.26	*SLC39A11*	0.000174	-1.33
MYBL1	5.65E-05	1.01	*SLC26A6*	0.00229	-1.33

MMP16	5.78E-05	2.05	*CCNG2*	0.00239	-1.33
RAD54B	5.82E-05	1.39	*PLXNB2*	0.00506	-1.33
MTBP	5.85E-05	1.64	*TST*	0.00601	-1.33
CCDC77	5.88E-05	1.95	*ACHE*	0.000131	-1.32
TMEM106C	6.38E-05	1.08	*ESRRG*	0.000291	-1.32
ESPL1	6.52E-05	1.4	*TMBIM4*	0.000549	-1.32
MMP10	6.54E-05	1.43	*ANXA2R*	0.00171	-1.32
NFIA	6.61E-05	2.35	*GBE1*	0.0023	-1.32
CLDN16	6.65E-05	2	*AFMID*	0.00336	-1.32
KLF2	6.65E-05	1.7	*TRAPPC6A*	0.0126	-1.32
CHMP4C	6.66E-05	1.41	*FAXDC2*	3.43E-05	-1.31
XK	6.67E-05	1.1	*GHR*	5.65E-05	-1.31
CSTF2	6.76E-05	1.64	*PLEKHA5*	9.19E-05	-1.31
SNORD57	6.81E-05	1.01	*KREMEN2*	0.000128	-1.31
EXOSC9	6.86E-05	1.65	*ACAD11*	0.000299	-1.31
TTC26	6.96E-05	1.43	*SERPINH1*	0.000346	-1.31
ZNF684	6.96E-05	1.15	*EVA1B*	0.000563	-1.31
SHTN1	7.14E-05	2.1	*ALDH7A1*	0.000361	-1.3
ZWILCH	7.14E-05	1.82	*DKFZP564C152*	1.21E-05	-1.3
SH3GLB1	7.18E-05	1.81	*TOB1*	0.000132	-1.3
FOXR1	7.18E-05	1.42	*DMD*	0.000279	-1.3
TMEM56	7.19E-05	1.59	*RPL28*	0.000693	-1.3
RFC3	7.38E-05	2.34	*SLC16A5*	0.00173	-1.3
PEG10	7.44E-05	2.66	*SLC25A12*	0.00387	-1.3
RBBP8	7.52E-05	2.53	*RABAC1*	0.00401	-1.3
TIPIN	7.62E-05	2.03	*RPL15*	3.38E-05	-1.29
HOXB6	7.68E-05	1.7	*DDB2*	6.17E-05	-1.29
ARHGAP44	7.70E-05	1.59	*TRNP1*	0.000168	-1.29
ASB7	7.70E-05	1.19	*IER5L*	0.000171	-1.29
CENPQ	7.80E-05	1.95	*HOXC4*	0.000196	-1.29
NT5C2	7.80E-05	1.82	*ANKLE1*	0.000361	-1.29
CXORF57	7.80E-05	1.54	*S100A1*	0.000611	-1.29
ADGRV1	7.80E-05	1.36	*ESPNL*	0.00143	-1.29
BTG3	8.00E-05	1.83	*EVI5L*	0.00206	-1.29
MAR-01	8.05E-05	1.3	*LAMC1*	0.00208	-1.29
GPI	8.07E-05	1.12	*DLX5*	0.0033	-1.29
LYAR	8.11E-05	1.52	*ASGR1*	0.00431	-1.29
FANCG	8.16E-05	1.87	*NUDT10*	2.72E-05	-1.28
CHAF1A	8.21E-05	1.35	*SLC27A3*	0.000109	-1.28
TRMT5	8.25E-05	1.22	*IDUA*	0.000141	-1.28
PHLDA1	8.34E-05	1.7	*PABPC4L*	0.000173	-1.28
IL17RB	8.36E-05	1.23	*TMEM101*	0.000441	-1.28
APITD1	8.56E-05	1.31	*FARP1*	0.000746	-1.28
CHDH	8.79E-05	1.46	*PPM1F*	0.00149	-1.28
KBTBD11	8.79E-05	1.25	*ZNF561*	0.0015	-1.28
CT45A3	8.90E-05	1.4	*DNAAF3*	0.00157	-1.28

ZNF215	9.06E-05	1.29	*ACSF2*	0.00109	-1.27
SLIT2	9.30E-05	2.94	*PLA2G4C*	0.000114	-1.27
ADGRG6	9.30E-05	2.22	*UCN*	0.000697	-1.27
TUBA4A	9.30E-05	1.91	*MKRN3*	0.000884	-1.27
CNN1	9.35E-05	1.85	*PGGHG*	0.00551	-1.27
SSX2IP	9.36E-05	1.58	*VANGL2*	0.0208	-1.27
GGH	9.46E-05	1.94	*FCGRT*	1.21E-05	-1.26
NUP35	9.73E-05	1.28	*MLH1*	4.44E-05	-1.26
MBNL3	9.74E-05	1.29	*COQ10A*	0.00113	-1.26
CTSA	9.74E-05	1.03	*GRIN3B*	0.00143	-1.26
C1ORF115	9.91E-05	1.29	*PHACTR2*	0.00392	-1.26
GCNT2	1.00E-04	1.58	*VPS36*	0.00406	-1.26
MIS18A	0.000101	1.54	*TLCD1*	0.00504	-1.26
C3ORF14	0.000103	2.65	*APLP1*	0.00626	-1.26
KIAA1524	0.000103	1.44	*BCKDHA*	0.0063	-1.26
BORA	0.000103	1.22	*ZBTB5*	0.00647	-1.26
SASS6	0.000105	1.64	*ABHD14A*	0.0192	-1.26
KIF15	0.000106	2	*SNHG7*	0.007	-1.25
SPAG5	0.000106	1.56	*SYCP2*	0.0122	-1.25
UNG	0.000108	1.56	*SYTL1*	2.90E-05	-1.25
HMGN2	0.000112	1.25	*CYTH2*	0.000377	-1.25
LGALS3	0.000115	1.34	*OXLD1*	0.00326	-1.25
CHAF1B	0.000116	1.35	*RXRA*	0.00567	-1.25
ARRDC4	0.000116	1.27	*SNORD104*	0.00755	-1.25
TPX2	0.000123	1.93	*PHC1*	0.00912	-1.25
SPC24	0.000123	1.22	*RIMKLB*	2.58E-05	-1.24
MRE11A	0.000124	1.21	*PIK3IP1*	0.000143	-1.24
TNFRSF21	0.000124	1.12	*ASAP3*	0.000385	-1.24
RAB23	0.000124	1.03	*FBXW7*	0.00102	-1.24
SGO2	0.000127	1.92	*FAM65C*	0.00127	-1.24
GKAP1	0.000127	1.08	*ZNF672*	0.00409	-1.24
MLF1	0.000128	1.98	*NR1H3*	0.00577	-1.24
PTPN2	0.000128	1.42	*SH3BGR*	0.00993	-1.24
ACTR6	0.000128	1.02	*P4HA2*	0.000183	-1.23
SLC6A15	0.000131	1.28	*BCAS3*	3.21E-05	-1.23
SLC12A6	0.000131	1.16	*PLAU*	3.69E-05	-1.23
ZNF714	0.000131	1.08	*PLCD4*	4.47E-05	-1.23
PTTG3P	0.000133	2.6	*METTL8*	9.82E-05	-1.23
RBM11	0.000133	1.39	*LRPAP1*	0.00161	-1.23
ACD	0.000135	1.43	*LOC100240735*	0.00188	-1.23
PSMG1	0.000135	1.15	*PLOD1*	0.00342	-1.23
NUP210	0.000135	1.1	*FOXN3*	0.00573	-1.23
FIGNL1	0.000138	1.64	*SULF2*	0.00011	-1.22
PLA2G16	0.000139	1.81	*S100A10*	0.00243	-1.22
GLRB	0.000139	1.4	*SCPEP1*	7.61E-05	-1.22
CXADR	0.000139	1.25	*SMPDL3A*	0.00109	-1.22

TMEM158	0.000142	1.51	*FKBP6*	0.00109	-1.22
CDT1	0.000145	1.68	*ZBTB44*	0.00172	-1.22
BRINP3	0.000145	1.42	*ALDOC*	0.0307	-1.22
LARP1B	0.000146	1.18	*BEX1*	0.0189	-1.21
PAPSS2	0.000149	2.18	*FAM110D*	7.81E-05	-1.21
SEL1L3	0.000151	1.4	*CDO1*	2.58E-05	-1.21
SLC9A7	0.000153	1.33	*ANKRD10*	0.00111	-1.21
CENPL	0.000153	1.3	*CHRNA3*	0.000126	-1.21
NSMCE4A	0.000155	1.23	*WIF1*	7.14E-05	-1.21
EYA4	0.000157	1.62	*OLFML3*	0.000184	-1.21
RNF138	0.000157	1.24	*EPYC*	0.000193	-1.21
RRM2	0.000161	2.66	*PRRT2*	0.000632	-1.21
SH3KBP1	0.000161	1.9	*SNHG8*	0.000632	-1.21
CREM	0.000162	1.92	*RPL13AP6*	0.00072	-1.21
KATNA1	0.000163	1.04	*RAB26*	0.00117	-1.21
TMPO	0.000164	2.59	*COL6A2*	0.00121	-1.21
TRIM2	0.000166	1.72	*HLA-DPA1*	0.00143	-1.21
THOC1	0.000166	1.07	*NINJ1*	0.00268	-1.21
ANXA2P1	0.000166	1.03	*TLE4*	0.0141	-1.21
CENPJ	0.000172	1.6	*RCOR2*	0.0199	-1.21
BRCA2	0.000172	1.17	*ACOT2*	0.0443	-1.21
PARD6A	0.000173	1.48	*C12orf76*	0.00154	-1.2
ORC3	0.000178	1.47	*FRAT2*	2.87E-05	-1.2
ASF1B	0.000179	1.72	*RPP25*	3.73E-05	-1.2
FERMT1	0.000179	1.21	*ZDHHC11*	6.65E-05	-1.2
NKIRAS1	0.000184	1.05	*SMYD3*	0.000118	-1.2
BUB1B	0.000185	1.37	*SRGAP3*	0.000139	-1.2
TRMT6	0.000186	1.54	*PALMD*	0.000192	-1.2
RDH10	0.000188	2.95	*TRIM8*	0.00157	-1.2
KIAA0101	0.000188	2.61	*SCRIB*	0.00188	-1.2
SERPINB9	0.000188	1.12	*CRELD1*	0.00252	-1.2
NFIL3	0.000191	1.16	*MEST*	0.00263	-1.2
POLR3G	0.000193	1.28	*TPT1*	0.00284	-1.2
DCLK1	0.000193	1.15	*TRIM4*	7.19E-05	-1.19
MYOF	0.000195	2.74	*TANC2*	0.000339	-1.19
ICA1	0.000196	1.64	*MAP7D1*	0.00037	-1.19
POLE3	0.000197	1.45	*KLHL3*	0.00081	-1.19
PUM3	0.000197	1.43	*ZNF79*	1.70E-05	-1.18
EFNB2	0.000199	1.37	*PLD6*	9.07E-05	-1.18
KCTD8	2.00E-04	1.07	*OXA1L*	0.000125	-1.18
THAP10	0.000203	1.13	*MEMO1*	0.000184	-1.18
TUBA3D	0.000204	2.39	*FAM63B*	0.000923	-1.18
CCNH	0.000204	1.17	*TRIM3*	0.00272	-1.18
MCM6	0.000213	1.42	*SBK1*	0.00364	-1.18
NDC1	0.000213	1.03	*CRELD2*	0.0108	-1.18
CENPK	0.000214	1.73	*HSD17B12*	0.00161	-1.17

DIRC2	0.000214	1.18	*UPF3A*	0.000184	-1.17
KNSTRN	0.000215	1.51	*RETSAT*	1.21E-05	-1.17
CDC25A	0.000222	1.6	*PTGS2*	0.000114	-1.17
POLD3	0.000222	1.09	*ZFP90*	0.000597	-1.17
PARP2	0.000223	1.72	*COLEC11*	0.00388	-1.16
HOXB4	0.000224	1.12	*PRKCZ*	0.000149	-1.16
NOCT	0.000226	1.01	*APOBEC3F*	2.91E-05	-1.16
MCM7	0.000233	2.13	*RPL22*	7.02E-05	-1.16
FAM46A	0.000233	1.26	*HES1*	0.000127	-1.16
PDSS1	0.000234	1.26	*CALR*	0.000456	-1.16
RBFOX2	0.000237	1.23	*FOXD1*	0.000638	-1.16
FAM72A	0.000238	1.26	*C1orf53*	0.000888	-1.16
LSM3	0.000239	1.4	*LGALS1*	0.00129	-1.16
SLFN11	0.000241	1.01	*VAMP2*	0.00159	-1.16
RMI2	0.000244	1.68	*VPS37D*	0.00193	-1.16
GLA	0.000247	1.62	*GALNT4*	0.00259	-1.16
PIK3CA	0.000248	2.79	*POLH*	0.00285	-1.16
ERAP2	0.000248	1.43	*CUEDC1*	0.0309	-1.16
VWA5A	0.000251	1.01	*ASS1*	0.000125	-1.15
USP37	0.000254	1.16	*CNPY4*	2.29E-05	-1.15
PTPN3	0.000254	1.1	*CNRIP1*	9.46E-05	-1.15
DHFR	0.000261	1.13	*JARID2*	0.000154	-1.15
UBQLN1	0.000262	1.36	*RPL37*	0.000698	-1.15
ITGB3BP	0.000275	1.73	*DMPK*	0.000889	-1.15
RARRES2	0.000276	1.05	*DYNC1I2*	0.00121	-1.15
JADE1	0.000281	1.25	*PLCXD1*	0.00149	-1.15
MMP12	0.000282	1.21	*G6PC3*	0.00157	-1.15
ATAD2	0.000283	2.6	*STRADA*	0.00284	-1.15
KIF22	0.000291	1.51	*SMAD3*	0.00331	-1.15
MUS81	0.000301	1.23	*ASCC3*	0.0063	-1.15
PSRC1	0.000301	1.2	*PPP1CB*	0.0118	-1.15
ACTL6A	0.000302	1.3	*FBXO22*	0.000353	-1.14
NOP56	0.000303	1.23	*ANKRA2*	0.000158	-1.14
C2ORF69	0.000304	1.4	*IKBIP*	0.00398	-1.14
OXCT1	0.000304	1.26	*CORO1B*	0.00268	-1.14
CEP152	0.000306	1.35	*EFNA1*	7.55E-05	-1.14
SLC10A4	0.000309	1.3	*DNM3*	0.000128	-1.14
KLHL2	0.000309	1.08	*MGAT3*	0.000657	-1.14
BARD1	0.000314	1.91	*RPL32*	0.00253	-1.14
RAB21	0.000317	1.35	*SOX18*	0.0121	-1.14
CENPI	0.00032	1.19	*CERS6*	0.0146	-1.14
MTERF3	0.000325	1.39	*DGKA*	5.81E-05	-1.13
POLE	0.000325	1.04	*FAT1*	0.00592	-1.13
SNX10	0.000326	1.34	*TRAPPC12*	3.76E-05	-1.13
LSM6	0.000328	1.27	*PRKAB1*	3.76E-05	-1.13
POC1A	0.00034	1.75	*TMCO3*	5.14E-05	-1.13

CENPE	0.000341	1.71	*MAFA-AS1*	0.000132	-1.13
RIF1	0.000341	1.08	*PREP*	0.00108	-1.13
FAIM	0.000342	1.11	*HSPB3*	0.00113	-1.13
RRM1	0.000355	1.63	*PGLS*	0.00229	-1.13
NRGN	0.000356	1.41	*GSTT1*	0.00295	-1.13
DCLRE1A	0.000358	1.32	*TBX2*	0.00717	-1.13
DCK	0.000361	1.52	*DHRS4L2*	0.0154	-1.13
ORC6	0.000361	1.25	*PREPL*	0.0183	-1.13
MCM3	0.000362	2.01	*C9orf85*	0.00237	-1.12
RANBP1	0.000362	1.28	*PALM*	0.0024	-1.12
FAM184A	0.000362	1.04	*GRN*	0.0011	-1.12
POLA2	0.000368	1.49	*COL26A1*	1.64E-05	-1.12
MBD2	0.000373	1.08	*SPIN3*	3.13E-05	-1.12
HMGB2	0.000374	1.06	*FBXO44*	0.000177	-1.12
CMSS1	0.000381	1.67	*KLF9*	0.000593	-1.12
HCCS	0.000386	1.42	*ABCA7*	0.000668	-1.12
TAF5	0.000393	1.38	*NYNRIN*	0.0021	-1.12
KMT5B	0.000393	1.25	*SUN3*	0.00284	-1.12
HNRNPAB	0.000401	1.18	*IRF2BP2*	0.00081	-1.11
SMC2	0.000406	1.64	*HOTAIR*	5.82E-05	-1.11
PSMB9	0.000409	1.06	*TMEM261*	2.80E-05	-1.11
WDHD1	0.000419	1.33	*ZFP64*	3.98E-05	-1.11
SNX7	0.000425	1.06	*FOLR1*	0.000146	-1.11
XRCC3	0.000438	1.13	*TUBD1*	0.000203	-1.11
DUSP23	0.00044	1.3	*AHNAK2*	0.000284	-1.11
CTSV	0.000441	1.14	*MMRN1*	0.000479	-1.11
NUDT7	0.000449	1.23	*ECHDC2*	0.000768	-1.11
CHORDC1	0.000454	1.55	*COL5A1*	0.000826	-1.11
GSG1	0.000459	1.04	*H3F3B*	0.00108	-1.11
CDKN2A	0.000467	1.43	*GPR108*	0.0015	-1.11
BACE2	0.000483	1.9	*MDM2*	0.00157	-1.11
LZTFL1	0.00049	1.04	*FOXL2NB*	0.0017	-1.11
BAZ1A	0.000491	2.17	*TM9SF2*	0.00194	-1.11
EAF2	0.000512	1.32	*SIRT4*	0.00194	-1.11
RPA2	0.000513	1.4	*GPR162*	0.00283	-1.11
SKP2	0.000513	1.38	*TRPM4*	0.0048	-1.11
C11ORF70	0.000517	1.53	*LAMA5*	0.00678	-1.11
SKIL	0.000517	1.1	*P4HB*	0.00769	-1.11
PKMYT1	0.000518	1.19	*CCND1*	0.00816	-1.11
HELZ2	0.000521	1.35	*ZDHHC9*	0.0245	-1.11
ASNSD1	0.000521	1.22	*C21orf33*	0.0122	-1.1
NOVA1	0.000532	1.02	*TOMM20*	0.000151	-1.1
RBBP7	0.000539	1.44	*IDO1*	0.000282	-1.1
HPF1	0.000539	1.07	*RGL3*	0.00108	-1.1
GLRX	0.000549	1.17	*MZF1*	0.00299	-1.1
POLA1	0.000551	1.1	*MRPL33*	0.00445	-1.1

PON2	0.000553	1.26	*HLA-DMA*	0.00499	-1.1
GLMN	0.000554	1.05	*SOBP*	0.0134	-1.1
KATNAL1	0.000564	1.1	*ARID4B*	0.0466	-1.1
PTTG1	0.000568	2.43	*BCHE*	0.000329	-1.09
PGM3	0.00057	1.18	*TMEM106B*	0.0157	-1.09
SUMO3	0.000581	1.09	*TFPI2*	0.00014	-1.09
SAAL1	0.000585	1.11	*EGLN1*	0.000292	-1.09
MPLKIP	0.00059	1.16	*PNMA2*	0.000304	-1.09
CSRP2	0.000593	1.21	*VPS28*	0.000556	-1.09
IRF1	0.000602	1.24	*UBTD1*	0.000713	-1.09
WDR76	0.000602	1.09	*ST13*	0.00129	-1.09
PIGW	0.000606	1.05	*GNPTG*	0.00287	-1.09
TUBA1B	0.000623	1.34	*MXD1*	0.00325	-1.09
CNOT1	0.000625	1.19	*MIR503HG*	0.00562	-1.09
UTP14A	0.000641	1.15	*NARF*	0.00788	-1.09
ARL4A	0.000644	1.66	*SLC43A2*	0.0101	-1.09
CRABP2	0.00065	2.29	*P2RX6*	0.015	-1.09
MED30	0.000651	1.86	*CHD9*	0.0158	-1.09
RND3	0.000656	1.17	*CAPZB*	9.80E-05	-1.08
EXOSC6	0.000657	1.12	*UTP14C*	0.000117	-1.08
TUBB4B	0.000684	1.35	*GDF11*	0.000193	-1.08
RFC2	0.000693	1.24	*CCDC47*	0.000209	-1.08
C3ORF58	0.000722	2.48	*CYCS*	0.000223	-1.08
FABP5	0.000723	1.57	*RNF170*	0.000517	-1.08
LOC645166	0.000723	1.49	*STARD10*	0.000756	-1.08
DNAJC1	0.000724	1.22	*GALK1*	0.000787	-1.08
TAGLN2	0.000727	1.32	*SMAD5*	0.001	-1.08
CYB5R4	0.000744	1.66	*HSBP1*	0.00119	-1.08
HSPB1	0.000764	1.26	*CCND3*	0.00189	-1.08
TMSB10	0.000774	1.46	*VPS51*	0.0025	-1.08
POLE4	0.000782	1.11	*UBA52*	0.00628	-1.08
TUBA1C	0.000811	1.57	*IDO2*	6.63E-05	-1.07
FANCB	0.00082	1.06	*ARMCX3*	0.000159	-1.07
TOPBP1	0.000823	1.11	*MGP*	0.00897	-1.07
SLC41A2	0.000823	1.1	*GUSBP2*	8.79E-05	-1.07
DNMT3B	0.000826	1.02	*BTG1*	7.55E-05	-1.07
NUP93	0.000826	1.02	*DGKB*	9.04E-05	-1.07
STARD4	0.000845	1.84	*APOBEC3C*	0.000289	-1.07
STX7	0.000845	1.07	*YPEL2*	0.00302	-1.07
EEF1E1	0.000845	1.04	*ZNF451*	0.00398	-1.07
FAM72B	0.000858	1.59	*TGFB2*	0.0069	-1.07
RFC1	0.000884	1.2	*TGFBR3*	0.0096	-1.07
GEMIN2	0.000895	1.11	*BLVRB*	0.0153	-1.07
DHCR24	0.000909	1.1	*AAMDC*	0.023	-1.07
ANGPTL2	0.00091	1.02	*KCNQ2*	0.000432	-1.06
NCOA3	0.000918	1.23	*STON1*	0.000121	-1.06

NUDT1	0.000924	2.01	*OPRL1*	0.000204	-1.06
OSTF1	0.000924	1.03	*KIAA0355*	0.000369	-1.06
SCG5	0.000934	1.01	*ZNF362*	0.000604	-1.06
MOB1A	0.000937	1.52	*CCNG1*	0.000813	-1.06
PI4K2B	0.000939	1.29	*NIPAL3*	0.000887	-1.06
TMSB4X	0.000942	2.5	*MIR181A2HG*	0.00128	-1.06
POLR3C	0.000951	1.13	*DCAKD*	0.00144	-1.06
COMMD8	0.000957	1.16	*POLD4*	0.00257	-1.06
CNOT9	0.000974	1.27	*DHRS4*	0.0146	-1.06
HRASLS	0.000991	1.09	*WBSCR27*	0.016	-1.06
BMI1	0.000992	1.61	*KIF1B*	0.0255	-1.06
ANAPC10	0.000999	1.6	*ACOT1*	0.0377	-1.06
DEK	0.00104	1.38	*CD79B*	0.00345	-1.05
CD27-AS1	0.00104	1.31	*ASTN2*	0.0173	-1.05
CKS1B	0.00105	1.81	*NCOR2*	0.0237	-1.05
RAD23B	0.00106	1.16	*PCOLCE2*	3.29E-05	-1.05
CTPS1	0.00108	1.19	*CNTFR*	0.000306	-1.05
FTSJ1	0.00108	1.03	*Sep-06*	0.000704	-1.05
GPAM	0.0011	1.11	*CNIH1*	0.00127	-1.05
NEFH	0.00113	1.46	*FAM43A*	0.00264	-1.05
RANBP10	0.00116	1.87	*C17orf82*	0.00291	-1.05
FAM171B	0.00116	1.87	*MFSD11*	0.00354	-1.05
DIAPH3	0.00116	1.44	*VWA8*	0.00551	-1.05
PAQR3	0.00116	1.08	*UST*	0.0113	-1.05
UHRF1	0.00119	1.76	*NID2*	0.0127	-1.05
CENPW	0.0012	1.79	*GADD45A*	0.00579	-1.04
ALG6	0.00121	1.03	*RAB40B*	6.35E-05	-1.04
MAP9	0.00122	1.27	*FRS2*	0.000215	-1.04
MPHOSPH6	0.00124	1.25	*BCL2L2*	0.000241	-1.04
CENPN	0.00126	1.85	*MAP3K12*	0.000513	-1.04
PRPS2	0.00127	1.09	*FAM172A*	0.000559	-1.04
MAP3K14	0.00127	1.07	*ST5*	0.000623	-1.04
EIF5A2	0.00129	1.21	*TNRC6B*	0.000787	-1.04
WWTR1	0.0013	1.25	*TEX19*	0.00156	-1.04
CALM1	0.00131	1.22	*TRIM5*	0.00635	-1.04
CHN1	0.00132	1.39	*TMEM94*	0.015	-1.04
TIMELESS	0.00133	1.38	*CACNA1H*	0.00459	-1.03
GPN3	0.00133	1.32	*MKNK2*	0.00243	-1.03
HMGN5	0.00133	1.23	*PARP3*	0.000795	-1.03
TPK1	0.00134	1.06	*SESN2*	0.000212	-1.03
DMKN	0.0014	1.72	*HLA-DRB1*	0.00025	-1.03
TEX30	0.0014	1.35	*RPS27L*	0.000312	-1.03
KIAA0895	0.00141	1.22	*ATRAID*	0.00053	-1.03
CXCL16	0.00144	1.68	*MCRIP1*	0.000912	-1.03
PLK1	0.00144	1.32	*C6orf48*	0.00093	-1.03
RPAP3	0.00145	1.15	*TM2D1*	0.00103	-1.03

PPIH	0.00152	1.48	*SLC2A14*	0.00106	-1.03
KIF1BP	0.00154	1.51	*HEXDC*	0.00122	-1.03
C16ORF59	0.00154	1.31	*CBX4*	0.00287	-1.03
TAF9B	0.00154	1.31	*UAP1L1*	0.00358	-1.03
BRI3BP	0.00154	1.18	*TGDS*	0.00362	-1.03
ARMCX6	0.00155	1.98	*STMN3*	0.00489	-1.03
MRPL3	0.00159	1.24	*MAN1A1*	0.0169	-1.03
CDK5RAP2	0.00171	1.11	*RHBDF1*	0.024	-1.03
LOC146880	0.00171	1.07	*EFNA4*	0.00336	-1.02
EZH2	0.00172	1.42	*MRPS25*	4.86E-05	-1.02
ATP9A	0.00173	1.1	*TRIAP1*	0.000165	-1.02
HIRIP3	0.00173	1.03	*HSD11B1L*	0.000272	-1.02
EMBP1	0.00173	1.01	*C1RL*	0.000355	-1.02
CCNF	0.0018	1.72	*CBX8*	0.00126	-1.02
PSMD12	0.00184	1.05	*TMEM145*	0.00178	-1.02
METTL1	0.00186	1.37	*TLE6*	0.00233	-1.02
MTHFD1	0.00194	1.07	*FAM127B*	0.00874	-1.02
SHMT1	0.00197	1.29	*AP3B1*	0.0118	-1.02
ZC3H15	0.00202	1.24	*MYCBP2*	0.0134	-1.02
PDCD2L	0.00204	1.07	*REEP6*	0.0135	-1.02
NOLC1	0.00208	1.16	*HSCB*	0.0163	-1.02
AZIN1	0.00209	1.34	*CAPN1*	0.0286	-1.02
USP18	0.00211	1.1	*MYL5*	0.00259	-1.01
DDR1	0.00212	1.11	*FCHSD2*	0.000138	-1.01
SMCO4	0.00213	1.19	*SLC6A16*	0.000173	-1.01
FGF2	0.00218	1.21	*PCID2*	0.000342	-1.01
DONSON	0.00228	1.41	*MAPRE3*	0.00126	-1.01
RPF1	0.00228	1.11	*ZNF618*	0.0013	-1.01
TUBA1A	0.0023	1.49	*TSHZ2*	0.00315	-1.01
ZNF91	0.00243	2.61	*QTRT1*	0.00471	-1.01
RCC1	0.00243	1.1	*EEF1G*	0.0101	-1.01
NEDD1	0.00246	1.42	*AK1*	0.0114	-1.01
PTPRG	0.00251	1.05	*AHSA2*	0.0132	-1.01
NEMP1	0.00255	1.31	*TNPO1*	0.0226	-1.01
NUP155	0.00258	1.36	*FLVCR1-AS1*	0.0372	-1.01
PCGF6	0.0026	1.1	*EDIL3*	0.0455	-1.01
GPD2	0.00261	1.13			
ARL6IP1	0.00272	1.31			
METTL18	0.00279	1.12			
DNAL1	0.00296	1.04			
SCYL2	0.00297	1.32			
RANBP2	0.00301	1.19			
SACS	0.00305	1.33			
SLC20A1	0.00305	1.25			
NET1	0.00317	1.01			
C4ORF46	0.0032	1.34			

SOD2	0.00325	1.16	
SPA17	0.00327	1.17	
ARHGAP19	0.00343	1.16	
SNORD16	0.00349	1.07	
ACYP1	0.00355	1.21	
TPRKB	0.00371	1.46	
G3BP1	0.00373	1.13	
STAG2	0.00379	1.45	
SYTL2	0.0038	1.4	
ID1	0.00382	1.2	
NCOA7	0.00386	1.08	
DNTTIP2	0.00397	1.1	
BCAS2	0.00406	1.37	
NDUFAF4	0.00409	1.15	
GAR1	0.00417	1.02	
UHMK1	0.00417	1.02	
VMO1	0.00428	1.02	
CENPM	0.00444	1.25	
SMC4	0.00448	1.75	
HAUS7	0.00468	1.3	
BCCIP	0.00479	1.07	
LIN9	0.00488	1.22	
GMNN	0.00489	1.11	
CCDC58	0.00491	2.25	
EEF1A2	0.00509	1.3	
HAUS1	0.0054	1.26	
ME1	0.00545	1.15	
MTFR1	0.00546	1.37	
PLS1	0.00551	1.39	
SNRNP25	0.00551	1.36	
MT2A	0.00552	1.26	
ANO3	0.00561	1.11	
LRRC40	0.00574	1.03	
CEP295	0.00597	1.06	
HPS3	0.00628	1.43	
RPL39L	0.00665	1.44	
CSE1L	0.00682	1.3	
C1D	0.00684	1.13	
EFCAB11	0.00711	1.11	
LACTB2	0.00748	1.06	
NFXL1	0.00773	1.05	
TWF1	0.00812	1.34	
CDC20	0.00825	1.65	
LONRF1	0.00825	1.14	
MSH2	0.00847	1.18	
GINS4	0.00851	1.05	

HOXB8	0.00872	1.08	
KANK1	0.00879	1.26	
MTAP	0.00919	1.09	
WBP11	0.00921	1.18	
BZW1	0.00937	1.25	
KIF5B	0.00937	1.06	
MT1A	0.00966	1.29	
PDCD5	0.00968	1.05	
HSP90AA1	0.0102	1.29	
BCL2L12	0.0103	1.04	
EIF4G2	0.0105	1.49	
SIVA1	0.0106	1.03	
MT1X	0.0107	1.13	
GPRASP2	0.011	1.28	
ZNF614	0.0112	1.11	
HIST1H4C	0.0118	2.63	
PSMC3	0.0122	1.34	
NBN	0.0125	1.14	
SNORD96A	0.0128	1.05	
IFI6	0.0129	1.22	
H2AFX	0.013	1.06	
CDC5L	0.0134	1.02	
PIK3CB	0.0135	1.03	
PROSER1	0.0142	1.01	
DDX3X	0.0144	1.24	
DCAF13P3	0.0146	1.05	
ABCE1	0.0158	1.44	
SF3B2	0.0158	1.07	
CTNNAL1	0.0158	1.01	
NCAPD2	0.0163	1.04	
ISG15	0.0166	1.08	
DR1	0.0168	1.12	
HNRNPH1	0.0189	1.08	
TRIP12	0.0192	1.35	
ADGRL2	0.0201	1.08	
CEP83	0.0215	1.04	
SLC38A1	0.0218	1.01	
DUSP6	0.0224	1.05	
DNAJC2	0.0225	1.02	
ZNF735	0.0227	1.63	
LOC644936	0.0244	1.21	
ZNF679	0.0255	1.27	
RANGAP1	0.0296	1.22	
COPRS	0.0313	1.02	
SNAR-A1	0.0321	2.61	
DIAPH1	0.0328	1.01	

DROSHA	0.0418	1.03	
SMARCA5	0.0431	1.15	
SSB	0.0436	1.45	
HIF1A	0.0458	1.25	
TIMM10	0.047	1.08	
BMPR1A	0.0471	1.05	
DDAH1	0.048	1.05	

Supplementary Table 3. GO analysis of differentially expressed genes associated with ovarian cancer.

Regulation	Category	Term	Count	%	p-value	Fold enrichment	FDI
Up	GOTERM_BP_FAT	GO:0022402~cell cycle process	231	28.7	4.0E-80	3.9	7.7E 77
Up	GOTERM_BP_FAT	GO:1903047~mitotic cell cycle process	188	23.4	1.0E-79	4.9	2.0E 76
Up	GOTERM_BP_FAT	GO:0007049~cell cycle	255	31.7	4.9E-79	3.5	9.5E 76
Up	GOTERM_BP_FAT	GO:0000278~mitotic cell cycle	194	24.1	2.5E-78	4.6	4.9E 75
Up	GOTERM_BP_FAT	GO:0000278~mitotic nuclear division	111	13.8	1.2E-54	5.9	2.4E 51
Up	GOTERM_MF_FAT	GO:0017111~nucleoside-triphosphatase activity	79	9.8	3.7E-13	2.4	6.0E 10
Up	GOTERM_MF_FAT	GO:0016818~hydrolase activity, acting on acid anhydrides, in phosphorus-containing anhydrides	82	10.2	3.8E-13	2.4	6.2E 10
Up	GOTERM_MF_FAT	GO:0016817~hydrolase activity, acting on acid anhydrides	82	10.2	4.3E-13	2.4	7.0E 10
Up	GOTERM_MF_FAT	GO:0016462~pyrophosphatase activity	81	10.1	9.2E-13	2.4	1.5E 9
Up	GOTERM_MF_FAT	GO:0008094~DNA-dependent ATPase activity	21	2.6	8.0E-11	6.2	1.3E 7
Up	GOTERM_CC_FAT	GO:0005694~chromosome	156	19.4	2.3E-47	3.6	3.4E 44
Up	GOTERM_CC_FAT	GO:0044427~chromosomal part	141	17.5	1.0E-43	3.7	1.6E 40
Up	GOTERM_CC_FAT	GO:0098687~chromosomal region	87	10.8	1.2E-39	5.4	1.8E 36
Up	GOTERM_CC_FAT	GO:0000793~condensed chromosome	63	7.8	2.4E-33	6.5	3.6E 30
Down	GOTERM_BP_FAT	GO:0009887~organ morphogenesis	68	9.7	8.6E-7	1.9	1.7E 3
Down	GOTERM_BP_FAT	GO:0048468~cell development	111	15.9	3.2E-6	1.5	6.3E 3
Down	GOTERM_BP_FAT	GO:0000904~cell morphogenesis involved in differentiation	54	7.7	4.0E-6	2.0	7.7E 3
Down	GOTERM_BP_FAT	GO:0060485~mesenchyme development	25	3.6	5.6E-6	2.9	1.1E 2
Down	GOTERM_BP_FAT	GO:0034644~cellular response to UV	13	1.9	5.8E-6	5.2	1.1E 2
Down	GOTERM_MF_FAT	GO:0001540~beta-amyloid binding	8	1.1	1.8E-4	6.5	3.0E 1

Down	GOTERM_MF_FAT	GO:0004090~carbonyl reductase (NADPH) activity	4	0.6	4.5E-4	22.0	7.3E[illegible] 1
Down	GOTERM_MF_FAT	GO:0005518~collagen binding	10	1.4	4.8E-4	4.3	7.7E[illegible] 1
Down	GOTERM_MF_FAT	GO:0033218~amide binding	22	3.1	5.4E-4	2.3	8.8E[illegible] 1
Down	GOTERM_MF_FAT	GO:0005509~calcium ion binding	44	6.3	8.7E-4	1.7	1.4E[illegible]
Down	GOTERM_CC_FAT	GO:0031982~membrane-bounded vesicle	197	28.1	8.3E-9	1.4	1.2E[illegible] 5
Down	GOTERM_CC_FAT	GO:1903561~extracellular vesicle	162	23.1	1.4E-8	1.5	2.1E[illegible] 5
Down	GOTERM_CC_FAT	GO:0043230~extracellular organelle	162	23.1	1.4E-8	1.5	2.1E[illegible] 5
Down	GOTERM_CC_FAT	GO:0044421~extracellular region part	207	29.6	1.7E-8	1.4	2.5E[illegible] 5

Abbreviations: GO, Gene Ontology; FDR, False Discovery Rate; BP, Biological Process; MF, Molecular Function; CC, Cell Component.

Supplementary Table 4. KEGG pathway analysis of target genes associated with ovarian cancer.

Regulation	Category	Term	Count	%	p-value	Fold enrichment	FDR
Up	KEGG_PATHWAY	hsa03030:DNA replication	23	2.9	1.2E-21	14.6	1.5E-18
Up	KEGG_PATHWAY	hsa04110:Cell cycle	32	4.0	6.5E-16	5.9	8.5E-13
Up	KEGG_PATHWAY	hsa03420:Nucleotide excision repair	13	1.6	5.0E-7	6.3	6.5E-4
Up	KEGG_PATHWAY	hsa03460:Fanconi anemia pathway	13	1.6	2.0E-6	5.6	2.6E-3
Up	KEGG_PATHWAY	hsa03430:Mismatch repair	9	1.1	3.3E-6	8.9	4.2E-3
Down	KEGG_PATHWAY	hsa04115:p53 signaling pathway	17	2.4	6.4E-9	6.2	8.3E-6
Down	KEGG_PATHWAY	hsa04512:ECM-receptor interaction	12	1.7	7.4E-4	3.4	9.6E-1
Down	KEGG_PATHWAY	hsa04151:PI3K-Akt signaling pathway	28	4.0	7.6E-4	2.0	9.9E-1
Down	KEGG_PATHWAY	hsa00330:Arginine and Proline metabolism	8	1.1	3.8E-3	3.9	4.8E0
Down	KEGG_PATHWAY	hsa05200:Pathways in cancer	27	3.9	9.2E-3	1.7	1.1E1

Abbreviations: GO, Gene Ontology; FDR, False Discovery Rate; KEGG, Kyoto Encyclopedia of Genes and Genomes.
